# Supplementary material for: Photoredox-catalyzed oxo-amination of aryl cyclopropanes
Source: Nat Commun. 2019 Sep 25;10:4367. doi: 10.1038/s41467-019-12403-2 (PMC6761154; doi:10.1038/s41467-019-12403-2)
Supplement: Supplementary file 1 — Supplementary Information [file 41467_2019_12403_MOESM1_ESM.pdf]

Supporting Information for

Photoredox-Catalyzed Oxo-Amination of Aryl Cyclopropanes

Ge et al.

Table of Contents

Supplementary Methods 2
Substrates Preparation..3
Reaction Optimization 21
General Procedure for Oxo-Amination of Aryl Cyclopropanes...27
General Procedure for One-pot Aminoacylation of Olefins...27
Supplementary Discussion..28
Cyclic Voltammetry 28
Emission Quenching Experiments (Stern-Volmer Studies) 29
Light On/Off Experiment..31
Determination of the Reaction Quantum Yield (Φ) 32
Radical Inhibition Experiment with TEMPO...37
Evidence of Alkyl Hydroperoxide Intermediate 38
Ruling Out the Intermediacy of 1,2-Dioxolane..38
The Examination of Reactions Between Pyrazole and **1ak**, **1ba** and **1bb**..............................39
The Examination of Aza-Nucleophiles with Different Oxidation Potentials..........................40
Experiment with Enantiometically Enriched Diphenyl Cyclopropane 41
Regioselectivity Determination for Oxo-amination of Unsymmetrical Diaryl Cyclopropanes 47
Characterization of Structurally Novel Compounds 53
1H, and 13C NMR Spectra of Structurally Novel Compounds 78
Supplementary Reference 186

Supplementary Methods

General Information

Unless otherwise noted, all reactions were carried out under oxygen atmosphere. All commercially available reagents were used directly without further purification unless noted. All solvents were dried by passing through a column of neutral alumina under nitrogen prior to use. Organic solutions were concentrated under reduced pressure on an IKA RV 10 rotary evaporator. Chromatography was performed using silica gel with distilled solvents. Thin-layer chromatography (TLC) was performed on Silicycle 250 μm silica gel plates visualized under UV light (254 nm) and dyed with cerous molybdate solution by heating.

HRMS spectra were recorded on a Xevo G2-XS QTof (Waters Corporation). ^{1}H NMR and ^{13}C NMR spectra were recorded using Bruker Avance 400 MHz spectrometers. Chemical shifts for ^{1}H NMR spectra are reported as δ in units of parts per million (ppm) downfield from $SiMe_4$ (δ 0.0) and relative to the signal of chloroform-d (δ 7.26, singlet). Multiplicities were given as: s (singlet); d (doublet); t (triplet); q (quartet); dd (doublet of doublets); dt (doublet of triplets); m (multiplet), etc. Coupling constants are reported as a *J* value in Hz. Carbon nuclear magnetic resonance spectra (^{13}C NMR) are reported as δ in units of parts per million (ppm) downfield from $SiMe_4$ (δ 0.0) and relative to the signal of chloroform-d (δ 77.00, triplet).

The photocatalyst $[Ir(dF(CF_3)ppy)_2(4,4'\text{-}dCF_3bpy)]PF_6$ (PC-III)[2], $Ir(dF(CF_3)ppy)_2(5,5'\text{-}dCF_3bpy)]PF_6$ (PC-IV)[2] and (PC-II)[3] were prepared following literature procedures. Acr-Mes-$Me^{+}BF_4^{-}$ (PC-I)[1], PC-IX, and PC-XIII were purchased from Energy.

Substrates Preparation

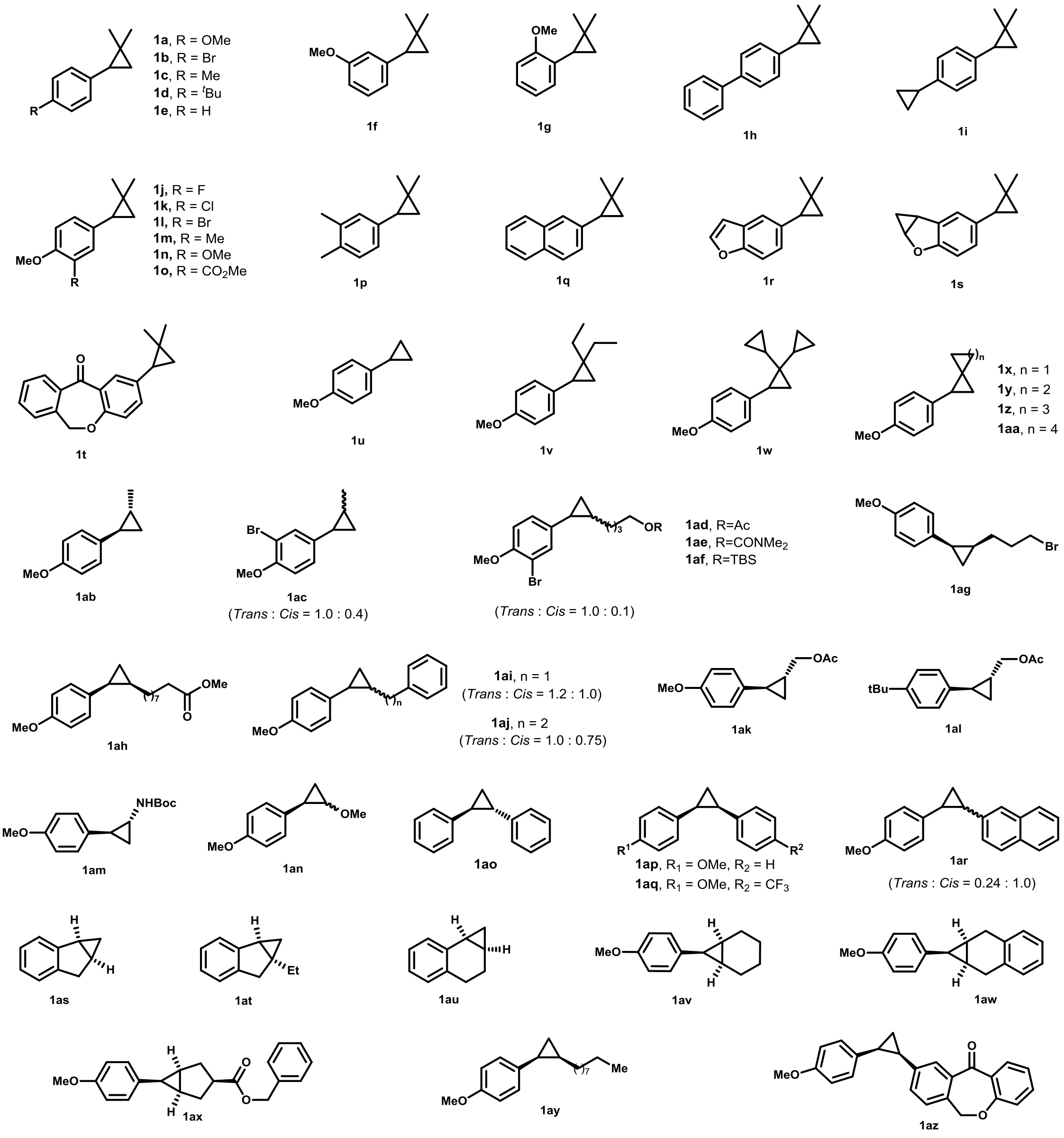

Compounds **1a, 1c**, **1e, 1f, 1g, 1n, 1u, 1x, 1y, 1ab, 1ak, 1am, 1ao-1aq, 1as, 1au** and **1av** were prepared according to the literature procedures, and all the spectroscopic data are in agreement with the literature reports.

General Procedure A for Aryl Cyclopropanes Synthesis[4]

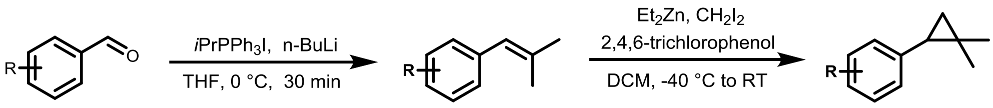

a) To a 150 mL oven-dried round-bottom flask equiped with a stir bar, the isopropyltriphenylphosphonium iodide (6.0 g, 13.88 mmol, 1.2 equiv) and anhydrous

THF (70 mL, 0.2 M) was added. The reaction flask was capped with rubber septum and charged with N_2 balloon and then the reaction mixture was cooled to 0 ℃. *n*-BuLi (2.5 M, 5.6 mL, 14 mmol, 1.2 equiv) was added dropwise by syringe and the reaction mixture was stirred at this temperature for 30 min. The corresponding solution of aldehyde (11.6 mmol, 1.0 equiv) in THF (20 mL) was added by syringe and the reaction mixture was allowed to warm to room temperature, and then stirred for 16 h. After the reaction reached completion according to the TLC analysis, the reaction mixture was quenched by sat. NH_4Cl (30 mL) and extracted with EtOAc (100mL) for 3 times. The combined organic layers were washed with H_2O_2 (10 wt% in water, 10 mL) and brine (20 mL), dried over Na_2SO_4 (20 g), and filtered. After the volatile materials were removed under reduced pressure, the crude residue was purified by column chromatography (PE : EtOAc = 50 : 1 to 10 : 1) to afford the desired alkene.

b) In a 150 mL oven-dried round-bottom flask with a stir bar, was added 2,4,6-trichlorophenol (1.18 g, 6.0 mmol, 2.5 equiv) under nitrogen atmosphere. DCM (60 mL, 0.1 M) was added into the flask and the reaction mixture was cooled to -40 ℃. $ZnEt_2$ (1.0 M, 6.0 mL, 6.0 mmol, 2.5 equiv) was added slowly into the flask by syringe and the reaction mixture was stirred at this temperature for 15 min. CH_2I_2 (2.57 g, 9.6 mmol, 4.0 equiv) was added slowly by syringe and the reaction mixture was stirred at this temperature for another 15 min. Next, the corresponding solution of alkene (2.4 mmol, 1.0 equiv) in DCM (10 mL) was added by syringe and the reaction mixture was allowed to warm to room temperature and stirred for 16 h. After the reaction reached completion (as judged by ^{1}H-NMR of an alloquat removed from the reaction vessel and worked up by evaporation), the reaction mixture was quenched with sat. NH_4Cl (30 mL) and extracted with DCM (100mL) for 3 times. The combined organic layers were washed with aq. NaOH (1.0 M, 30 mL) and brine (20 mL), dried over Na_2SO_4 (20 g) and filtered. After the volatile materials were removed under reduced pressure, the crude residue was purified by column chromatography (PE : EtOAc = 50 : 1 to 10 : 1) to afford the desired compound.

Compounds **1a**, **1c**, **1e**, **1j-1n**, **1p** and **1q** were prepared following the General Procedure A. Overall yield: **1a** (1.36 mmol, 57%) as a colorless liquid; **1c** (1.42 mmol, 59%) as a colorless liquid; **1e** (1.22 mmol, 51%) as a colorless liquid; **1j** (1.34 mmol, 56%) as a colorless liquid; **1q** (1.08 mmol, 45%) as a colorless liquid; **1k** (1.22 mmol, 51%) as a colorless liquid; **1l** (1.13 mmol, 47%) as a colorless liquid; **1m** (1.46 mmol, 61%) as a colorless liquid; **1n** (1.63 mmol, 68%) as a colorless liquid, **1p** (1.32 mmol, 55%) as a white soild.

General Procedure B for Aryl Cyclopropanes Synthesis[5]

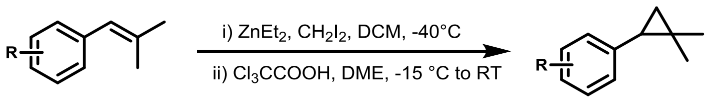

To a 50 mL oven-dried round-bottom flask equipped with a stir bar, was added DCM (3 mL, 0.67 M) under nitrogen atmosphere and was cooled to -40 ℃. $ZnEt_2$ (2.0 M, 2.5 mL, 5.0 mmol, 2.5 equiv) was added followed by slow addition of a solution of CH_2I_2 (2.68 g, 10.0 mmol, 5.0 equiv) in DCM (1 mL) by syringe. The reaction mixture was stirred at this temperature for 1 h followed by warming to -10 ℃. Next, a the solution of trichloroacetic acid (60 mg, 0.4 mmol, 0.2 equiv) and DME (224 mg, 2.4 mmol, 1.2 equiv) in DCM (1 mL) was added dropwise into and the reaction mixture by syringe and the resulting solution was allowed to stir at -10 ℃ for another 1 h. A solution of corresponding alkene (2.0 mmol, 1.0 equiv) in DCM (1 mL) was then added by syringe and the reaction mixture was allowed to warmed to room temperature and stirred for 16 h. After the reaction reached completion (as judged by ^{1}H-NMR of an alloquat removed from the reaction vessel and worked up by evaporation), the reaction mixture was quenched with sat. NH_4Cl (30 mL) and extracted with DCM (20 mL) for 3 times. The combined organic layers were washed with aq. NaOH (1.0 M, 30 mL) and brine (20 mL), dried over Na_2SO_4 (20 g) and filtered. After the volatile materials were removed under reduced pressure, the crude residue was purified by column chromatography (PE : EtOAc = 50 : 1 to 10 : 1) to afford the corresponding title compound.

Compounds **1b**, **1d**, **1h** and **1o** were prepared following the General Procedure B. Over yield: **1b** (1.20 mmol, 60 %) as a colorless liquid; **1d** (1.28 mmol, 64%) as a colorless liquid; **1h** (0.94 mmol, 47%) as a colorless liquid; **1o** (0.82 mmol, 41%).

General Procedure C for Diaryl Cyclopropanes Synthesis

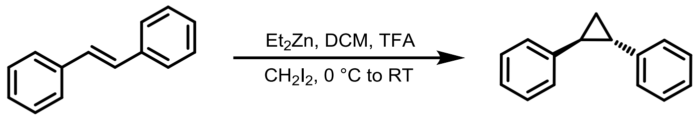

Following the literature method,[6] to a 50 mL oven-dried round-bottom flask with a stir bar was added DCM (3 mL, 0.67 M) under a nitrogen atmosphere. The flask was cooled to 0 ℃. $ZnEt_2$ (2.0 M, 2.8 mL, 5.5 mmol, 5.5 equiv) was added dropwise and followed by dropwise addition of a solution of TFA (0.5 mL, 6.0 mmol, 6.0 equiv) in DCM (1 mL) by syringe over 15 min. The solution was then stirred at this temperature for 15 min. A solution of CH_2I_2 (1.61 g, 6.0 mmol, 6.0 equiv) in DCM (1 mL) was added by syringe and the reaction mixture was stirred at this temperature for 1 h. Then a solution of the corresponding alkene (1 mmol, 1.0 equiv) in DCM (1 mL) was added by syringe and the reaction mixture was allowed to warm to room temperature and stirred for 20 h. After the reaction reached completion (as judged by ^{1}H-NMR of an alloquat removed from the reaction vessel and worked up by evoperation), the reaction mixture was quenched with sat. NH_4Cl (30mL) and extracted with DCM (50 mL) for 3 times. The combined organic layers were washed with sat. $NaHCO_3$ (50 mL) and brine (20 mL), dried over Na_2SO_4 (20 g) and filtered. After the volatile materials were removed under reduced pressure, the crude residue was purified by column chromatography (PE : EtOAc = 50 : 1 to 10 : 1) to afford the desired compound.

Compounds **1ao** was prepared following the General Procedure C. Overall yield: **1ao** (0.40 mmol, 40 %) as a colorless liquid.

General Procedure D for Aryl Cyclopropanes Synthesis[8]

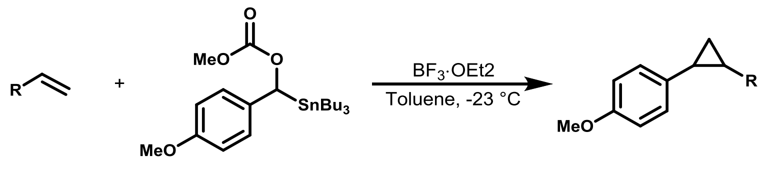

To a 50 mL oven-dried round-bottom flask equipped with a stir bar was added the solution of (4-methoxyphenyl)(tributylstannyl)methyl methyl carbonate[8] (486 mg, 1.0 mmol, 1.0 equiv) and the corresponding alkene (1.1 mmol, 1.0 equiv) in toluene (3.5 mL) under nitrogen atmosphere at room temperature. The reaction vessel was cooled to -23 ℃. $BF_3 \cdot OEt_2$ (156 mg, 1.1 mmol, 1.1 equiv) was added by syringe and stirred at this temperature for 2 h. After the reaction reached completion according to the TLC analysis, the reaction mixture was quenched with sat. $NaHCO_3$ (10 mL) and extracted with EtOAc (30 mL) for 3 times. The combined organic layers were washed with brine (20 mL), dried over Na_2SO_4 (20 g) and filtered. After the volatile materials were removed under reduced pressure, the crude residue was purified by column chromatography (PE : EtOAc = 50 : 1 to 10 : 1) to afford the target compound.

Compounds **1ag**, **1ah, 1ap**, **1aq**, **1ar**, **1av**, **1aw**, **1ax**, **1ay** and **1az** were prepared following the General Procedure D.

Yields of **1ag** (*Cis*, 0.22 mmol, 22%) as a colorless liquid; **1ap** (*Cis*, 0.40 mmol, 40%) as a colorless liquid; **1aq** (*Cis*, 0.64 mol, 64%) as a colorless liquid; **1ar** (*Trans* : *Cis* = 1.0 : 0.1, 0.71 mmol, 71%) as a white solid; **1av** (*Cis,* 0.67 mmol, 67%) as a colorless liquid; **1aw** (*Cis*, 0.44 mmol, 44%) as a colorless liquid; **1ax** (*Cis*, 0.54 mmol, 54%) as a white solid; **1az** (*Cis*, 0.68 mmol, 68%) as a white solid; **1ay** (reaction solvent was DCE, *Cis* product, directly used for one-pot reaction).

Synthesis of 1ak[7]

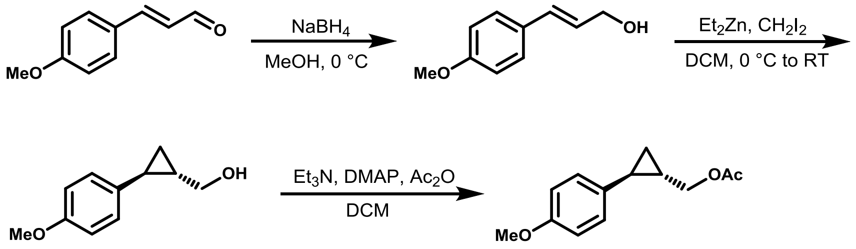

1ak

a) To a solution of (*E*)-3-(4-methoxyphenyl)acrylaldehyde (1.62 g, 10.0 mmol, 1.0 equiv) in MeOH (30 mL, 0.33 M) at 0 ℃ was added $NaBH_4$ (454 mg, 12 mmol, 1.2 equiv) and the reaction mixture was stirred at the same temperature for 2 h. The reaction mixture was then quenched with sat. aq. NH_4Cl (20 mL) and extracted with DCM (50 mL × 3). The combined organic layers were washed with brine (20 mL), dried over

Na_2SO_4(20 g) , filtered and concentrated under reduced pressure. The residue was purified by column chromatography (PE : EtOAc = 4 : 1 to 2 : 1) to provide (*E*)-3-(4-methoxyphenyl)prop-2-en-1-ol 1410 mg (8.60 mmol).

b) To a 50 mL oven-dried round-bottom flask equipped with a stir bar was added the solution of CH_2I_2 (510 μL, 6.4 mmol, 2.0 equiv) in DCM (20 mL) under nitrogen atmosphere. The flask was cooled to 0 ℃. Next, $ZnEt_2$ (2.0 M, 2.1 mL, 4.2 mmol, 1.3 equiv) was added by syringe and the reaction was stirred at this temperature for 30 min. At the same time, to separate 50 mL oven-dried round-bottom flask was added a solution of (*E*)-3-(4-methoxyphenyl)prop-2-en-1-ol (520 mg, 3.2 mmol, 1.0 equiv) in DCM (10 mL) under a nitrogen atmosphere at 0 ℃. Next, $ZnEt_2$ (2.0 M, 2.1 mL, 4.2 mmol, 1.3 equiv) was added dropwise by syringe over 10 min and then stirred at 0 ℃ for 30 min. The resultant solution was added to the first flask by syringe and the resulting reaction mixture was stirred at 0 ℃ for another 30 min. The reaction mixture was then allowed to warm to room temperature and stirred for 24 h. After the reaction reached completion according to the TLC analysis, the reaction mixture was quenched with sat. NH_4Cl (10mL) and extracted with DCM (30 mL) for 3 times. The combined organic layers were washed with sat. $NaHCO_3$ (20 mL) and brine (20 mL), dried over Na_2SO_4 (20 g) and filtered. After the volatile materials were removed under reduced pressure, the crude residue was purified by column chromatography (PE : EtOAc = 4 : 1 to 2 : 1) to afford (2-(4-methoxyphenyl)cyclopropyl)methanol 490 mg (2.75 mmol, 86%) and its NMR spectra match the literature data.[7]

c) (2-(4-Methoxyphenyl)cyclopropyl)methanol (140 mg, 0.786 mmol, 1.0 equiv) was dissolved in DCM (3.0 mL) followed by addion of DMAP (12 mg, 0.08 equiv), TEA (150 μL, 1.9 mmol, 2.4 equiv) and Ac_2O (80 μL, 1.57 mmol, 2.0 equiv) at room temperature. The reaction mixture was stirred at room temperature for 16 h. After reached completion according to the TLC analysis, the reaction mixture was quenched with sat. NH_4Cl (10mL) and extracted with DCM (20 mL) for 3 times. The combined organic layers were washed with sat. $NaHCO_3$ (20 mL) and brine (20 mL), dried over Na_2SO_4 (20 g) and filtered. After the volatile materials were removed under reduced

pressure, the crude residue was purified by column chromatography (PE : EtOAc = 10 : 1 to 4 : 1) to give compound **1ak** (132 mg, 0.60 mmol, 76% yield) as a colorless liquid and its NMR spectra match the literature data.

Synthetic Procedure for 1ab

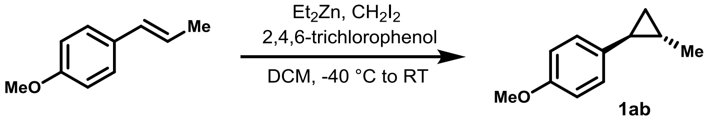

Compound **1ab** was prepared from (*E*)-1-methoxy-4-(prop-1-en-1-yl)benzene following the cyclopropanation method in General Procedure A and obtained in 73% yield as a colorless liquid.

Synthetic Procedure for 1aa

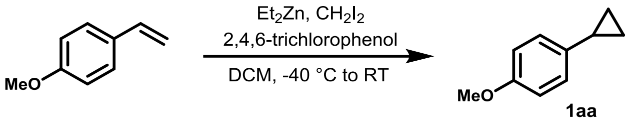

The compound **1aa** was prepared from 1-methoxy-4-vinylbenzene following the cyclopropanation method in General Procedure A and obtained in 77% yield as a colorless liquid.

Synthetic Procedure for 1v

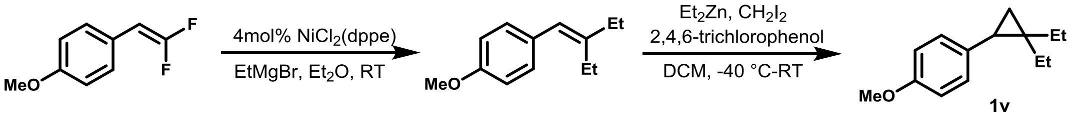

a) To an oven-dried 50 mL round-bottom flask equipped with a stir bar, 1-(2,2-difluorovinyl)-4-methoxybenzene (340 mg, 2.0 mmol, 1.0 equiv), $NiCl_2$(dppe) (42 mg, 4 mol%) and Et_2O (15 mL) was added under nitrogen atmosphere at room temperature. To this stirring solution at room temperature, EtMgBr (1.0 M in THF, 4.8 mL, 4.8 mmol, 2.4 equiv) was added slowly by syringe and the resulting mixture was stirred for an additional 2 h. Once the reaction was judged to be complete by TLC analysis, the reaction mixture was quenched with sat. NH_4Cl (20 mL) and extracted with EtOAc (30 mL) for 3 times. The combined organic layers were washed with sat. $NaHCO_3$ (30 mL) and brine (20 mL), dried over Na_2SO_4 (20 g) and filtered. After the volatile materials

were removed under reduced pressure, the crude residue was purified by column chromatography (PE : EtOAc = 50 : 1 to 10 : 1) to afford 1-(2-ethylbut-1-en-1-yl)-4-methoxybenzene as colorless oil (294 mg, 1.54 mmol, yield 77%).

b) Compound **1v** was prepared from 1-(2-ethylbut-1-en-1-yl)-4-methoxybenzene following the cyclopropanation method in General Procedure A and purified by column chromatography (PE : EtOAc = 50 : 1 to 10 : 1) and was obtained in 67% yield as a colorless liquid.

Synthetic Procedure for 1x

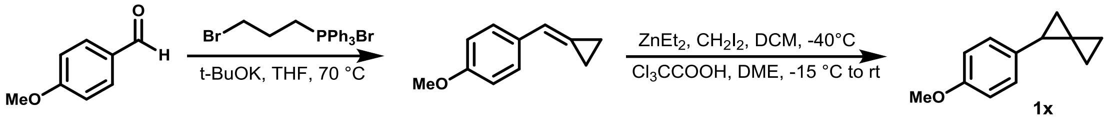

a) To a 150 mL oven-dried round-bottom flask equipped with a stir bar was added bromo(3-bromopropyl)triphenylphosphorane (7.0 g, 15.0 mmol, 1.2 equiv) and anhydrous THF (30 mL, 0.5 M) under a nitrogen atmosphere at room temperature. After a solution of *t*-BuOK (3.4 g, 30 mmol, 3.0 equiv) in THF (25 mL) was added by syringe and the resulting reaction mixture was then heated to 70 ºC and stirred at 70 ℃ for 1 h. Next, 4-methoxybenzaldehyde (1.36 g, 10.0 mmol, 1.0 equiv) was added by syringe and the reaction mixture was stirred at 70 ℃ for an additional 3 h. After the reaction reached completion according to the TLC analysis, the reaction was cooled down to room temperature and quenched with sat.NH_4Cl (50 mL), extracted with EtOAc (50 mL) for 3 times. The combined organic layers were washed with H_2O (50 mL) and brine (20 mL), dried over Na_2SO_4 (20 g) and filtered. After the volatile materials were removed under reduced pressure the crude residue was purified by column chromatography (PE : EtOAc = 50 : 1 to 10 : 1) to afford 1-(cyclopropylidenemethyl)-4-methoxybenzene as colorless oil (244 mg, 1.52 mmol, 15% yield).

b) Compound **1x** was prepared from 1-(cyclopropylidenemethyl)-4-methoxybenzene following the General Procedure B, purified by column chromatography (PE : EtOAc = 50 : 1 to 10 : 1) and was obtained in 77% yield as a colorless liquid.

Synthetic Procedure for 1z[9]

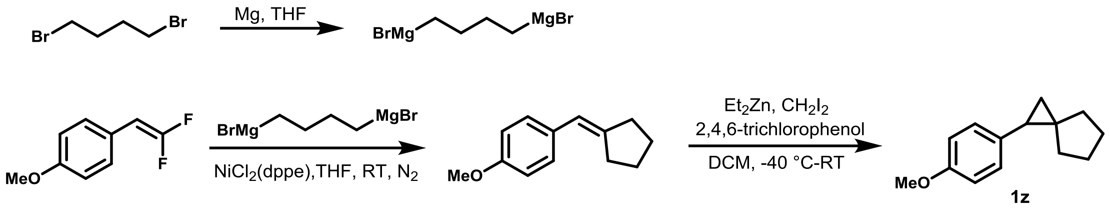

a) To an oven-dried 100 mL 3-necked round-bottom flask equipped with a stir bar, magnesium turnings (2.7 g, 110 mmol, 2.2 equiv) were added followed by anhydrous THF (15 mL) under nitrogen atmosphere. Then a solution of 1,4-dibromobutane (10.75 g, 50.0 mmol, 1.0 equiv) in anhydrous THF (5 mL) was added dropwise by syringe at room temperature and the reaction mixture was further stirred at room temperature for 2 h to give the Grignard reagent (1.0 M). To a separate 50 mL oven-dried 3-necked round-bottom flask equipped with a stir bar, was added 1-(2,2-difluorovinyl)-4-methoxybenzene (340 mg, 2.0 mmol, 1.0 equiv), $NiCl_2$(dppe) (43 mg, 4 mol%) and THF (10 mL) under nitrogen atmosphere. Then the freshly prepared Grignard reagent (1.0 M, 2.0 mL, 2.0 mmol, 1.0 equiv) was added by syringe and the reaction mixture was stirred at RT for 1 h. After the reaction reached completion according to the TLC analysis, the reaction mixture was quenched with sat. NH_4Cl (20 mL) and extracted with EtOAc (50 mL) for 3 times. The combined organic layers were washed with H_2O (50 mL) and brine (20 mL), dried over Na_2SO_4 (20 g) and filtered. After the volatile materials were removed under reduced pressure, the crude residue was purified by column chromatography (PE : EtOAc = 50 : 1 to 10 : 1) to afford the 1-(cyclopentylidenemethyl)-4-methoxybenzene as colorless oil (155 mg, 0.82 mmol, yield 41%).

b) Compound **1z** was prepared from 1-(cyclopentylidenemethyl)-4-methoxybenzene following the cyclopropanation method in General Procedure A, purified by column chromatography (PE : EtOAc = 50 : 1 to 10 : 1) and was obtained in 71% yield as a colorless liquid.

Synthetic Procedure for 1aa

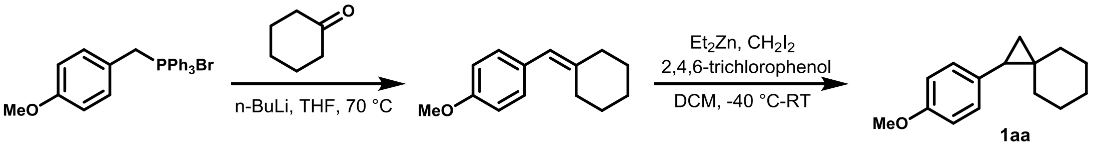

To an oven-dried 50 mL round-bottom flask with a stir bar, (4-methoxybenzyl) tris (phenyl) phosphonium bromide (2.0 g, 4.3 mmol, 1.0 equiv) was added followed by anhydrous THF (10 mL, 0.4 M) under nitrogen atmosphere at room temperature. Next, *n*-BuLi (2.5 M in THF solvent, 2.1 mL , 5.2 mmol, 1.2 equiv) was added by syringe and the reaction mixture was stirred at room temperature for 1 h. To the reaction mixture, cyclohexanone (510 mg, 5.20 mmol, 1.2 equiv) was added by syringe and the reaction mixture was stirred under reflux for 4 h. After the reaction reached completion according to the TLC analysis, the reaction mixture was cooled down to room temperature, quenched with sat.NH_4Cl (10 mL) with and extracted with EtOAc (50 mL) for 3 times. The combined organic layers were washed with H_2O_2 (10 wt% in water, 10 mL) and brine (20 mL), dried over Na_2SO_4 (20 g) and filtered. After the volatile materials were removed under reduced pressure, the crude residue was purified by column chromatography (PE : EtOAc = 50 : 1 to 10 : 1) to afford the 1-(cyclohexylidenemethyl)-4-methoxybenzene as colorless oil (500 mg, 2.47 mmol, yield 57.3%).

Compound **1aa** was prepared from 1-(cyclohexylidenemethyl)-4-methoxybenzene by following the cyclopropanation method in General Procedure A, purified by column chromatography (PE : EtOAc = 50 : 1 to 10 : 1) and was obtained in 71% yield as a colorless liquid.

Synthetic Procedure for 1w

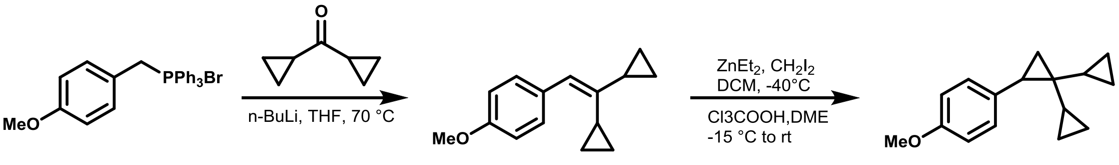

To an oven-dried 50 mL round-bottom flask with a stir bar, (4-methoxybenzyl) tris (phenyl) phosphonium bromide (1.0 g, 2.15 mmol, 1.0 equiv) was added followed by

anhydrous THF (5 mL, 0.4 M) under nitrogen atmosphere at room temperature. Next, *n*-BuLi (2.5 M in THF solvent, 1.1 mL, 2.75 mmol, 1.2 equiv) was added by syringe and the reaction mixture was stirred at room temperature for 1 h. To the reaction mixture, dicyclopropylmethanone (260 mg, 2.36 mmol, 1.1 equiv) was added by syringe and the reaction mixture was stirred under reflux for 4 h. After the reaction reached completion according to the TLC analysis, the reaction mixture was cooled down to room temperatue, quenched with sat.NH_4Cl (10 mL) and extracted with EtOAc (50 mL) for 3 times. The combined organic layers were washed with H_2O_2 (10 wt% in water, 10 mL) and brine (20 mL), dried over Na_2SO_4 (20 g) and filtered. After the volatile materials were removed under reduced pressure, the crude residue was purified by column chromatography (PE : EtOAc = 50 : 1 to 10 : 1) to afford the 1-(2,2-dicyclopropylvinyl)-4-methoxybenzene as a yellow oil (310 mg, 1.44 mmol, yield 67.3%).

Compound **1w** was prepared from 1-(2,2-dicyclopropylvinyl)-4-methoxybenzene following the General Procedure B, purified by column chromatography (PE : EtOAc = 50 : 1 to 10 : 1) and was obtained in 27% yield as a colorless liquid.

Synthetic Procedure for 1ai and 1aj

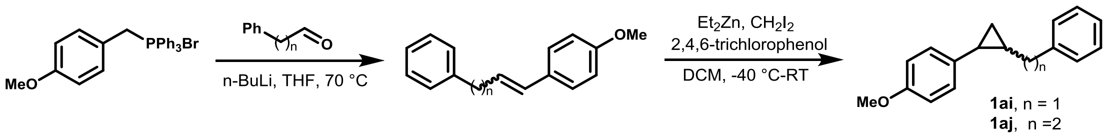

To an oven-dried 50 mL round-bottom flask with a stir bar, (4-methoxybenzyl) tris (phenyl) phosphonium bromide (1.0 g, 2.15 mmol, 1.0 equiv) was added followed by anhydrous THF (5 mL, 0.4 M) under nitrogen atmosphere at room temperature. Next, *n*-BuLi (2.5 M in THF solvent, 1.1 mL, 2.75 mmol, 1.2 equiv) was added by syringe and the reaction mixture was stirred at room temperature for 1 h. To the reaction mixture, 2-phenylacetaldehyde (280 mg, 2.36 mmol, 1.1 equiv) was added by syringe and the reaction mixture was stirred under reflux for 4 h. After the reaction reached completion according to the TLC analysis, the reaction mixture was cooled down to room temperatue, quenched with sat.NH_4Cl (10 mL) and extracted with EtOAc (50 mL) for 3 times. The combined organic layers were washed with H_2O_2 (10 wt% in water, 10

mL) and brine (20 mL), dried over Na_2SO_4 (20 g) and filtered. After the volatile materials were removed under reduced pressure, the crude residue was purified by column chromatography (PE : EtOAc = 50 : 1 to 10 : 1) to afford 1-methoxy-4-(3-phenylprop-1-en-1-yl)benzene as a yellow oil (*E*- and *Z*-isomer mixture as one spot on TLC, 292 mg, 1.30 mmol, yield 60.5%).

1-methoxy-4-(4-phenylbut-1-en-1-yl)benzene was obtained from 3-phenylpropanal by the same reaction and was purified by column chromatography (PE : EtOAc = 50 : 1 to 10 : 1) to afford a yellow oil in 55% yield (*E- and Z-isomer* mixture).

Compounds **1ai** was prepared from 1-methoxy-4-(3-phenylprop-1-en-1-yl)benzene by following the cyclopropanation method in General Procedure A, purified by column chromatography (PE : EtOAc = 50 : 1 to 10 : 1) and was obtained in overall yields of 23% as a colorless oil (*Trans* : *Cis* = 1.2 : 1.0 mixture as one spot on TLC).

Compounds **1aj** was prepared from 1-methoxy-4-(4-phenylbut-1-en-1-yl)benzene by following the cyclopropanation method in General Procedure A, purified by column chromatography (PE : EtOAc = 50 : 1 to 10 : 1) and was obtained in overall yields of 31% as a yellow oil (*Trans* : *Cis* = 1.0 : 0.75 mixture as one spot on TLC).

Synthetic Procedure for 1am[10]

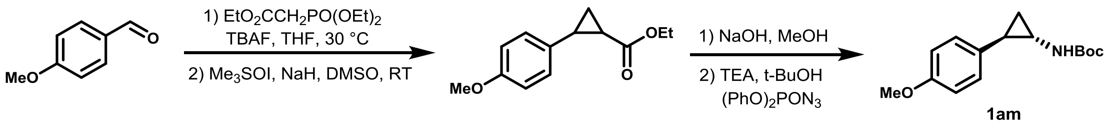

Compound **1am** was prepared according to the literature procedure[10] and obtained with an overall yield of 39% as a white solid.

Synthetic Procedure for 1ad-1af

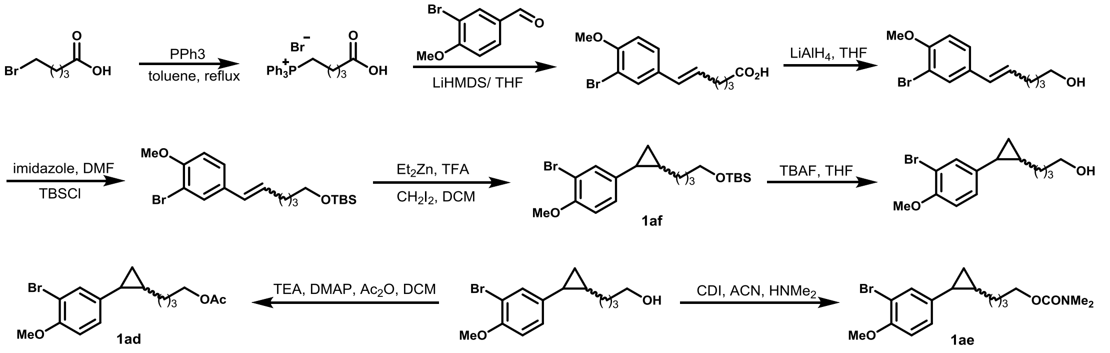

a) To an oven-dried 150 mL round-bottom flask equipped with a stir bar, (4-

carboxybutyl)triphenylphosphonium bromide (5.32 g, 12 mmol, 1.2 equiv) was added followed by anhydrous THF (25 mL) under a nitrogen atmosphere at 0 ℃. Then LiHMDS (1.0 M, 24 mL, 24 mmol, 2.4 equiv) was added dropwise by syringe and the reaction mixture was stirred at 0 ℃ for 1 h. A solution of 3-bromo-4-methoxybenzaldehyde (2.15 g, 10 mmol, 1.0 equiv) in THF (6.0 mL) was added by syringe and the reaction mixture was stirred at rt for 16 h. After the reaction was completed according to the TLC analysis, the reaction mixture was quenched with sat.NH_4Cl (50 mL) and extracted with EtOAc (50 mL) for 3 times. The combined organic layers were washed with H_2O (50 mL) and brine (20 mL), dried over Na_2SO_4 (20 g) and filtered. After removal of the volatile materials under reduced pressure, the crude residue was purified by column chromatography (PE : EtOAc = 10 : 1 to 3 : 1) to afford the 6-(3-bromo-4-methoxyphenyl)hex-5-enoic acid as a white solid 2.37g, (7.9 mmol, 79%).

b) To an oven-dried 500 mL round-bottom flask equipped with a stir bar, 6-(3-bromo-4-methoxyphenyl)hex-5-enoic acid (3.0 g, 10 mmol, 1.0 equiv) was added followed by anhydrous THF (100 mL) under a nitrogen atmosphere at 0 ℃. Then $LiAlH_4$ (380 mg, 10 mmol, 1.0 equiv) was added portionwise and the reaction mixture was stirred at 0 ℃ for 1 h. After the reaction was completed according to the TLC analysis, the reaction mixture was quenched with H_2O (380 μL) followed by the addition of aq. NaOH (15%, 380 μL) and H_2O (1.14 mL). The mixture was filtered and the filtrate was extracted with EtOAc. The combined organic layers were washed with H_2O (50 mL) and brine (20 mL), dried over Na_2SO_4 (20 g) and filtered. After volatile materials were removed under reduced pressure, the crude residue was purified by column chromatography (PE : EtOAc = 10 : 1 to 3 : 1) to afford the 6-(3-bromo-4-methoxyphenyl)hex-5-en-1-ol as a colorless oil 2.0g, (7.0 mmol, 70%).

c) To an oven-dried 50 mL round-bottom flask equipped with a stir bar, 6-(3-bromo-4-methoxyphenyl)hex-5-en-1-ol (670 mg, 2.4 mmol, 1.0 equiv) was added followed by DMF (5 mL) under a nitrogen at 0 ℃. Then imidazole (180 mg, 2.64 mmol, 1.1 equiv) was added followed by TBSCl (400 mg, 2.64 mmol, 1.1 equiv). The mixture was stirred

at room temperature for 2 h. After the reaction was completed according to the TLC analysis, the reaction mixture was quenched with H_2O (380 μL) and extracted with EtOAc. The combined organic layers were washed with sat. NH_4Cl (30 mL), H_2O (30 mL), and brine (20 mL) then dried over Na_2SO_4 (10 g) and filtered. After the volatile materials were removed under reduced pressure, the crude residue was purified by column chromatography (PE : EtOAc = 10 : 1 to 5 : 1) to afford ((6-(3-bromo-4-methoxyphenyl)hex-5-en-1-yl)oxy)(tert-butyl)dimethylsilane as a colorless oil 650 mg, (1.75 mmol, 73%).

d) **1af** was prepared from ((6-(3-bromo-4-methoxyphenyl)hex-5-en-1-yl)oxy) (*tert*-butyl)dimethylsilane (3.0 mmol) by following the General Procedure C purified by column chromatography (PE : EtOAc = 10 : 1 to 5 : 1), and was obtained in 73% yield (2.2 mmol) as a white solid.

e) **1af** (1.0 mmol) was added into a solution of TBAF (1.0 M in THF, 4.0 equiv) and the mixture was stirred at RT for 16 h. After the completion of reaction as judged by TLC analysis, the solution was concentrated under reduced pressure and further diluted with EtOAc. The combined organic layers were washed with H_2O and brine, dried over Na_2SO_4 and filtered. After removing volatile materials under reduced pressure, the crude product 4-(2-(3-bromo-4-methoxyphenyl) cyclopropyl)butan-1-ol was directly used without further purification.

f) **1ad** was prepared from 4-(2-(3-bromo-4-methoxyphenyl)cyclopropyl)butan-1-ol by following the acylation procedure for **1aj** synthesis and obtained (0.76 mmol) in 76% yield as a white solid.

g) To an oven-dried 50 mL round-bottom flask with a stir bar, 4-(2-(3-bromo-4-methoxyphenyl)cyclopropyl)butan-1-ol (120 mg, 0.4 mmol, 1.0 equiv) was added followed by MeCN (1 mL) under a nitrogen atmosphere at 0 ℃. Then CDI (1,1'-carbonyldiimidazole) (100 mg, 0.6 mmol, 1.5 equiv) was added and the mixture was stirred at rt for 1 h. After the reaction was completed according to the TLC analysis, $HNMe_2$ (2.0 M in THF, 0.5 mL, 2.5 equiv) was added and the mixture was stirred at rt for 16 h. After the reaction was completed according to the TLC analysis, the reaction

mixture was quenched with H_2O (380 μL) and extracted with EtOAc (20 mL) for 3 times. The combined organic layers were washed with sat. NH_4Cl, H_2O and brine then dried over Na_2SO_4 and filtered. After removal of the volatile materials under reduced pressure, the crude residue was purified by column chromatography (PE : EtOAc = 10 : 1 to 5 : 1) to afford **1ae** in 74% yield as a white solid.

Synthetic Procedure for 1al

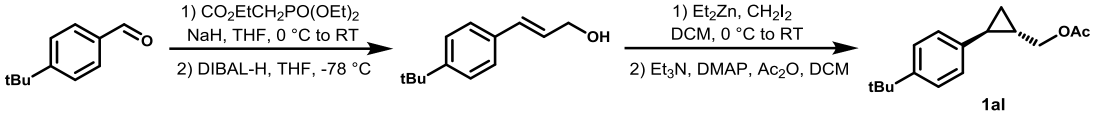

1al

1al (390 mg, 1.59 mmol) was prepared from (*E*)-3-(4-(*tert*-butyl)phenyl)prop-2-en-1-ol[11] (840 mg, 4.42 mmol) by following the cyclopropanation and acylation procedures for **1ak** synthesis and obtained in an overall yield of 36% as a colorless liquid.

Synthetic Procedure for 1r and 1s

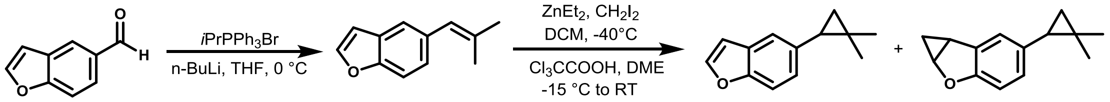

1r 1s

5-(2-Methylprop-1-en-1-yl)benzofuran (228 mg, 1.32 mmol) was subjected to General Procedure B with slight modifications by using Et_2Zn (2.0M, 2.0 mL, 4.0 mmol, 3.0 equiv), CH_2I_2 (2.15 g, 8.0 mmol, 6.0 equiv), Cl_3CCOOH (60 mg, 0.4 mmol, 0.3 equiv) and DME (180 mg, 2.0 mmol, 1.5 equiv). After work up, the residue of the reaction was purified by column chromatography (PE : EtOAc = 50 : 1 to 20 : 1) to afford **1r (**PE : EtOAc = 50 : 1, Rf = 0.6**)** 82 mg, 0.44mmol, 33% and **1s (**PE : EtOAc = 50 : 1, Rf = 0.4**)** 74 mg, 0.37mmol, 28% as colorless liquids, respectively.

Synthetic Procedure for 1i

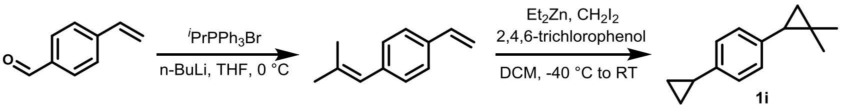

1i

Following the General Procedure A but doubling the stochiometries of respective reagents employed: 2,4,6-trichlorophenol (1.95 g, 10.0 mmol, 5.0 equiv); $ZnEt_2$ (2.0 M, 5.0 mL, 10.0 mmol, 5.0 equiv); CH_2I_2 (4.2 g, 12.8 mmol, 8.0 equiv), from 1-(2-

methylprop-1-en-1-yl)-4-vinylbenzene （316 mg, 2.0mmol）, **1i** (212 mg, 1.14 mmol) was preparedin 57% yield as a colorless liquid.

Synthetic Procedure for 1t[13]

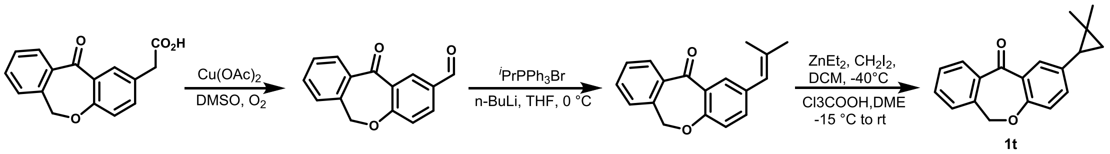

1t

To a three-necked flask was charged with isoxepac (1.34 g, 5.0 mmol), $Cu(OAc)_2$ (90 mg, 0.5 mmol), and DMSO (10 mL). After purging with O_2, the reaction flask was sealed with rubber septum and then heated to 120 ℃. After the reaction reached completion according to TLC analysis, the reaction mixture was diluted with ethyl acetate (50 mL) and brine (50 mL). The phases were separated and the aqueous layer was extracted with EtOAc (50 mL ×2). The combine organic layers were dried over anhydrous Na_2SO_4, filtered and concentrated under reduced pressure. The residue was purified by column chromatography on silica gel (PE : EtOAc = 10 : 1 to 5 : 1) to afford 11-oxo-6,11-dihydrodibenzo[b,e]oxepine-2-carbaldehyde 715 mg, (3.0 mmol, 60%). The following protocol for the synthesis of compound **1t** follows the General Procedure B, which allows the generation of desired product in 55% yield as a white solid.

Synthetic Procedure for 1az[6]

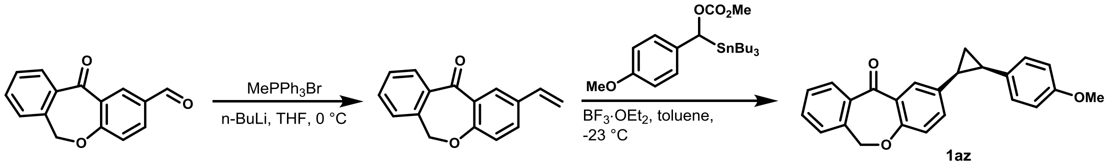

1az

1az was prepared from 2-vinyldibenzo[b,e]oxepin-11(6H)-one according to the General Procedure D in 70% yield as a white solid.

Synthetic Procedure for 1an

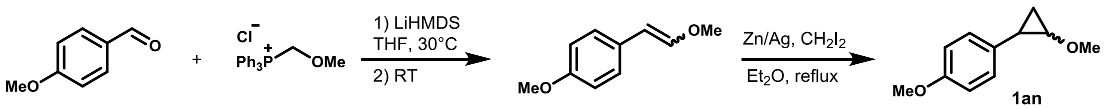

1an

a) To an oven-dried 150 mL round-bottom flask with a stir bar, (methoxymethyl)triphenylphosphonium chloride (4.1 g, 12.0 mmol, 1.2 equiv) was added followed by anhydrous THF (40 mL) under nitrogen atmosphere at room

temperature then the reaction solution was cooled to 0 ℃. Next, LiHMDS (1.0 M in THF solvent, 13.0 mL, 13.0 mmol, 1.3 equiv) was added by syringe and the reaction mixture was stirred at 0 ℃ for 30 min. To the reaction mixture, 4-methoxybenzaldehyde (1.36 g, 10.0 mmol, 1.0 equiv) in THF (10 mL) solution was added by syringe and the reaction mixture was stirred at room temperature for 16 h. After the reaction reached completion according to the TLC analysis, the reaction mixture was quenched with sat.NH_4Cl (10 mL) and extracted with EtOAc (50 mL) for 3 times. The combined organic layers were washed with H_2O_2 (10 wt% in water, 10 mL) and brine (20 mL), dried over Na_2SO_4 (20 g) and filtered. After the volatile materials were removed under reduced pressure, the crude residue was purified by column chromatography (PE : EtOAc = 50 : 1 to 10 : 1) to afford the 1-methoxy-4-(2-methoxyvinyl)benzene as a yellow oil (1.19 g, 7.25 mmol, yield 72.5%).

b) To an oven-dried 50 mL round-bottom flask with a stir bar, AcOH (10 mL) was warm ed 80 ℃, then AgAcO (10 mg) was added with stirring soon it became a clear solution. Then Zn powder (2.0 g) was added into the solution and stirred at 80 ℃ for 30 seconds. The resultant mixture was cooled down to room temperature and then solvent was decant, the residue solids were washed with ether (10 mL) for 5 times. To the residue solids was added 1-methoxy-4-(2-methoxyvinyl)benzene (820 mg, 5.0 mmol, 1.0 equiv) followed by anhydrous ether (10 mL) under nitrogen atmosphere at room temperature, then CH_2I_2 (2.0 g, 7.5 mmole, 1.5 equiv) was dropped into with stirring and then the reaction solution was stirred under reflux for 16 h. After the reaction reached completion according to the TLC analysis, the reaction mixture was quenched with sat.NH_4Cl (10 mL) and extracted with EtOAc (50 mL) for 3 times. The combined organic layers were washed with H_2O (50 mL) and brine (20 mL), dried over Na_2SO_4 (20 g) and filtered. After the volatile materials were removed under reduced pressure, the crude residue was purified by column chromatography (PE : EtOAc = 50 : 1 to 10 : 1) to afford the **1an** as a colorless oil (580 mg, 3.25 mmol, yield 65.0%, D,R = 1: 0.8).

Synthesis of (1R,2R)-1,2-diphenylcyclopropane

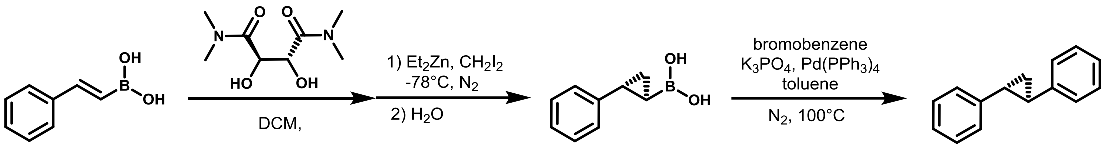

(1R, 2R)-1ao

a) To an oven-dried 100 mL round-bottom flask equipped with a stir bar, (E)-styrylboronic acid (0.75 g, 5 mmol, 1.0 equiv), +TMTA (1.05 g, 5 mmol, 1.0equiv) and dry DCM (20 mL) was added at room temperature. The reaction mixture was stirred for 2h and then cooled to -78 ℃. In a separate 100 mL flask, Et_2Zn (1.0M, 15 mL, 15 mmol, 3.0 equiv) was dissolved in DCM (20 mL), cooled to –78 ℃ and treated dropwise with CH_2I_2 (1.0 ml, 12 mmol, 4.8 equiv), then stirred vigorously for 10 min to generate the carbenoid (ineffective stirring due to precipitation of zinc salt or CH_2I_2 did not affect the reaction). The pre-chilled -78 ℃ solution was then quickly added *via syringe* over 2 min. The mixture was stirred at -78 ℃ for 8 h. 20 mL of saturated aqueous NH_4Cl solution was carefully added to quench the reaction. After addition of NH_4Cl, the mixture was stirred at -78 ℃ for 5 min, taken out of the cooling bath and warmed to ambient temperature. After phase separation, 1M HCl was added just to dissolve precipitate in the aqueous phase (pH was 5-6 at this point). The aqueous phase was extracted with 50 mL of DCM three times. The combined organic phases were dried with $MgSO_4$, filtered and concentrated and pumped to afford crude **((1R,2R)-2-phenylcyclopropyl)boronic acid** (directly used for the next step).

b) To an oven-dried 50 mL round-bottom flask equipped with a stir bar, crude **((1R,2R)-2-phenylcyclopropyl)boronic acid**, bromobenzene (720 mg, 4.5 mmol, 0.9 equiv), K_3PO_4 (3.2 g, 15 mmol, 3.3 equiv), $Pd(PPh_3)_4$ (156 mg, 0.03 quiv) and toluene (20 mL) was added under nitrogen atmosphere. The reaction mixture was stirred at 100 ℃ for 16 h. Once the reaction was judged to be complete by TLC analysis, the reaction mixture was filtered to remove the solids and the volatile materials of the reaction mixture were removed under reduced pressure, the crude residue was purified by column chromatography (PE 100%) to **(1S,2S)-1,2-diphenylcyclopropane** as a colorless oil (306 mg, 1.57 mmol, overall yield 35%, ee 90%). See Fig S3.

Synthetic procedure for 2e[14]

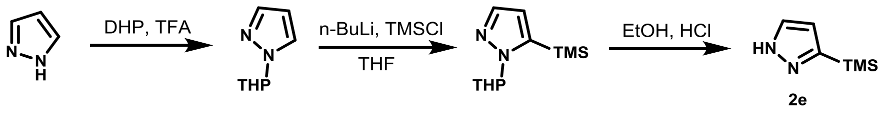

Compound **2e** was prepared according to the literature[14] with an overall yield of 68% as a white solid.

Reaction Optimization

Supplementary Table 1: Photocatalyst screening[a]

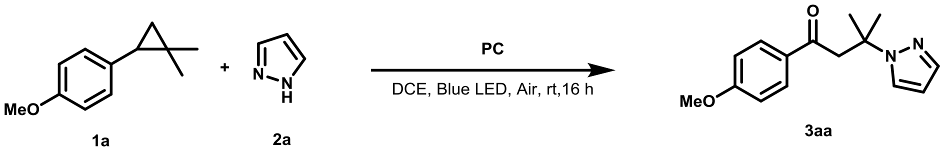

Entry	PC	Yield of 3aa (%)	Conversion (%)
1	PC-I	64	100
2	PC-II	NDP	100
3	PC-III	68	100
4	PC-IV	65	100
5	PC-V	47	70
6	PC-VI	57	100
7	PC-VII	trace	10
8	PC-VIII	trace	10
9	PC-IX	NDP	10
10	PC-X	trace	10
11	PC-XI	trace	10
12	PC--XII	NR	0
13	PC-XIII	NDP	100
14	PC-XIV	NDP	60
15	PC-XV	NDP	50
16 [b]	PC-III	NR	0
17 [c]	-	NR	0

[a] Experiments were performed with **1a** (0.1 mmol), **2a** (0.3 mmol), photocatalyst (2 mol%) in DCE (0.5 mL), irradiating with 15 W blue LEDs under air atmosphere at room temperature for 16 h. Yield

and conversion were determined by ^{1}H NMR using 1,1,2,2-tetrachloroethane as internal standard. NR, no reaction. NDP, no desired product. [b] Without irradiation. [c] Without photocatalyst.

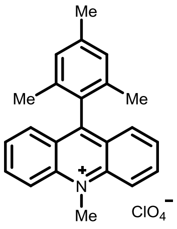

PC-I

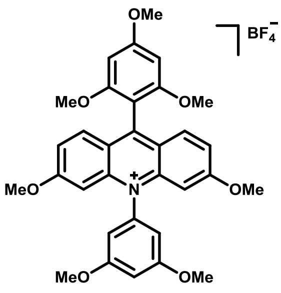

PC-II

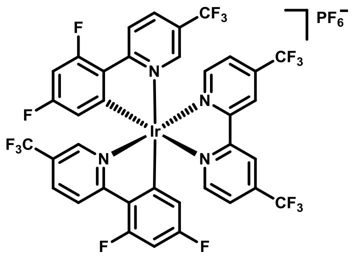

PC-III

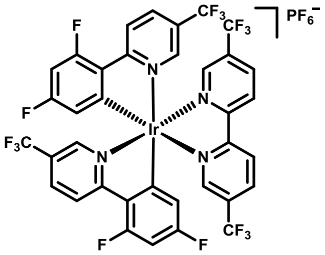

PC-IV

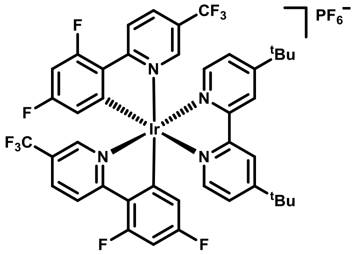

PC-V

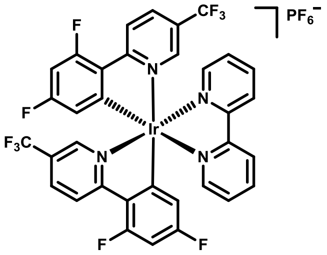

PC-VI

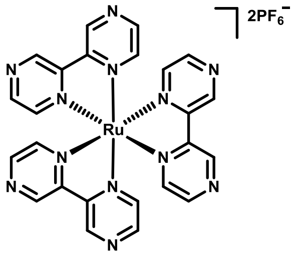

PC-VII

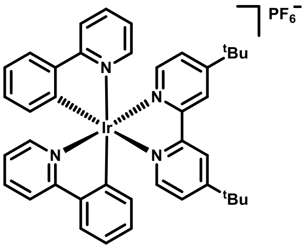

PC-VIII

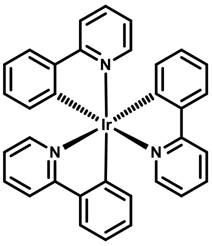

PC-IX

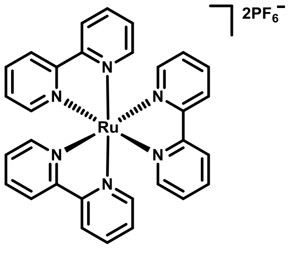

PC-X

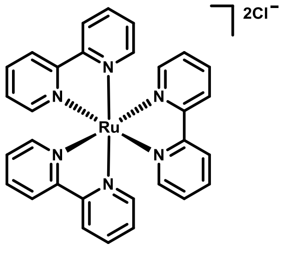

PC-XI

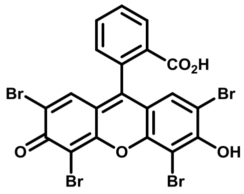

PC-XII

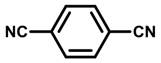

PC-XIII

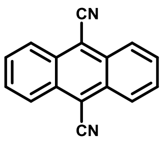

PC-XIV

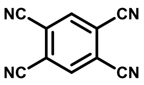

PC-XV

Supplementary Table 2: Solvent screening[a]

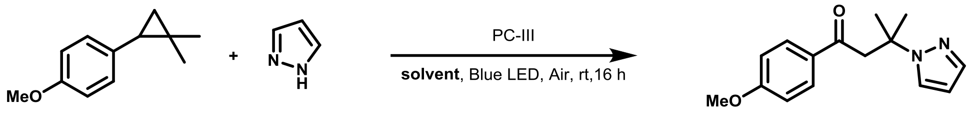

Entry	Solvent	Yield of 3aa(%)	Conversion(%)
1	DCE	70	100
2	DME	38	60
3	MeCN	53	65
4	DMF	0	100
5	DCM	66	100
6	MeOH	0	100
7	Chloroform	33	55
8	NMP	NR	0
9	MTBE	11	30
10	Et_2O	68	100
11	THF	16	40
12	DMAC	NR	0
13	1,4-dioxane	45	100
14	cyclohexane	trace	5
15	n-hexane	trace	5
16	toluene	0	100
17	$MeNO_2$	22	100

[a] Experiments were performed with **1a** (0.1 mmol), **2a** (0.2 mmol), photocatalyst (2 mol %) in solvent (0.5 mL), irradiating with 15 W blue LEDs under air atmosphere at room temperature for 16 h. Yield and conversion were determined by ^{1}H-NMR using 1,1,2,2-tetrachloroethane as internal standard.

Supplementary Table 3: Light source screening[a]

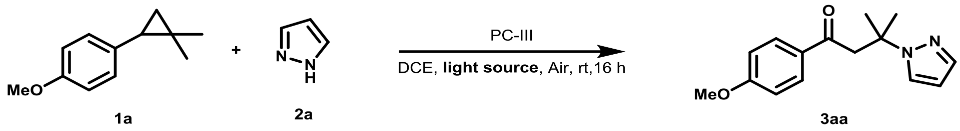

Entry	Light source	Yield of 3a(%)	Conversion(%)
1	Blue-LEDs	65	100
2	Purple-LEDs	51	100
3	white	35	55
4	black	NR	0

[a] Experiments were performed with **1a** (0.1 mmol), **2a** (0.2 mmol), photocatalyst (2 mol %) in DCE (0.5 mL), irradiating with or without light source under air atmosphere at room temperature for 16 h. Yield and conversion were determined by ^{1}H-NMR using 1,1,2,2-tetrachloroethane as internal standard. NR, no reaction.

Supplementary Table 4: Screening of equivalents of pyrazole[a]

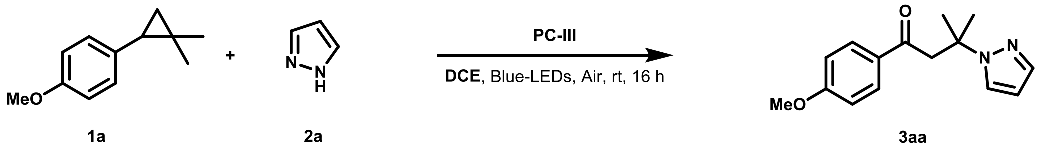

Entry	Equivalents of 2a	Yield of 3a(%)	Conversion(%)
1	1.0	53	70
2	2.0	65	100
3	4.0	66	100

[a] Experiments were performed with **1a** (0.1 mmol), **2a**, 4Å MS (50 mg), photocatalyst (2 mol%) in DCE (0.5 mL), irradiating with blue-LEDs under air atmosphere at room temperature for 16 h. Yield and conversion were determined by ^{1}H-NMR using 1,1,2,2-tetrachloroethane as internal standard.

Supplementary Table 5: Screening of reaction atmosphere[a]

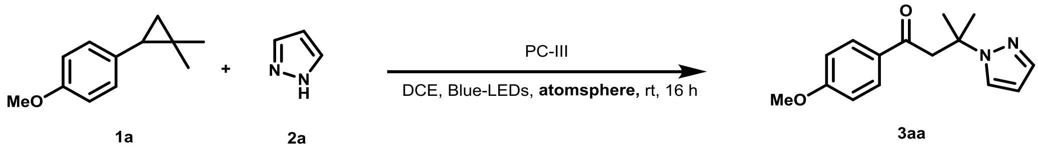

Entry	Atmosphere	Yield of 3aa(%)	Conversion(%)

1	N_2	0	0
2	air	65	100
3	O_2	74	100

[a] Experiments were performed with **1a** (0.1 mmol), **2a** (0.2 mmol), photocatalyst (2 mol%) in DCE (0.5 mL), irradiating with Blue-LEDs under atmosphere at room temperature for 16 h. Yield and conversion were determined by ^{1}H-NMR using 1,1,2,2-tetrachloroethane as internal standard.

Supplementary Table 6: Screening of additives[a]

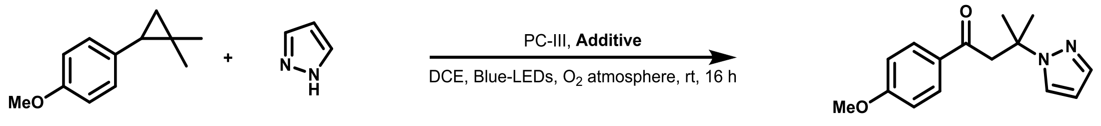

1a 2a 3aa

Entry	Additive	Yield of 3aa(%)	Conversion(%)
1	4Å-MS	85	100
2	3Å-MS	78	100
3	5Å-MS	80	100
4	Activated Al_2O_3	36	77
5	NaOH	NR	0
6	$CaCl_2$	43	73
7	Na_2SO_4	62	95
8	$MgSO_4$	75	100
9	Silica gel	50	90
10	K-CATALYST	75	100

[a] Experiments were performed with **1a** (0.1 mmol), **2a** (0.2 mmol), additive (50 mg), photocatalyst (2 mol%) in DCE (0.5 mL), irradiating with blue-LEDs under O_2 atmosphere at room temperature for 16 h. Yield and conversion were determined by ^{1}H-NMR using 1,1,2,2-tetrachloroethane as internal standard. MS, molecular sieve.

Supplementary Table 7: Screening photocatalyst loading[a]

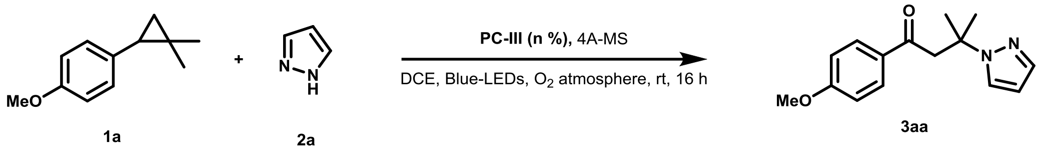

Entry	mol%	Yield of 3a(%)	Conversion (%)
1	2.0	85	100
2	1.0	86	100
3	0.5	83	100
4	0.2	85	100
5	0.1	81	100

[a] Experiments were performed with **1a** (0.1 mmol), **2a** (0.2 mmol), 4 Å MS (50 mg), photocatalyst (n mol %) in DCE (0.5 mL), irradiating with 15 W blue LEDs under O_2 atmosphere at room temperature for 16 h. Yield and conversion were determined by ^{1}H-NMR using 1,1,2,2-tetrachloroethane as internal standard.

Supplementary Table 8: Optimization of reaction concentration and time[a]

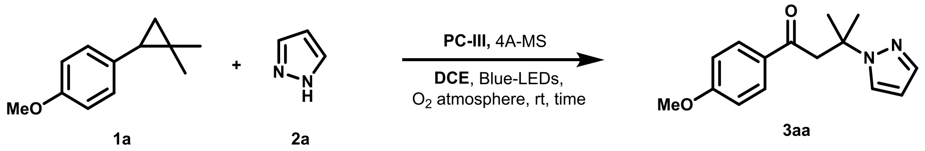

Entry	Concentration(mol/L)	Time(h)	Yield of 3a(%)	Conversion(%)
1	0.1	16	78	100
2	0.2	16	85	100
3	0.5	16	72	90
4	0.2	4	35	55
5	0.2	8	52	70
6	0.2	12	77	92

[a] Experiments were performed with **1a** (0.1 mmol), **2a** (0.2 mmol), 4 Å MS (50 mg), photocatalyst (0.2 mol %) in DCE (n M), irradiating with 15 W blue LEDs under O_2 atmosphere at room temperature for certain h. Yield and conversion were determined by ^{1}H-NMR using 1,1,2,2-tetrachloroethane as internal standard.

General Procedure E for Oxo-Amination of Aryl Cyclopropanes

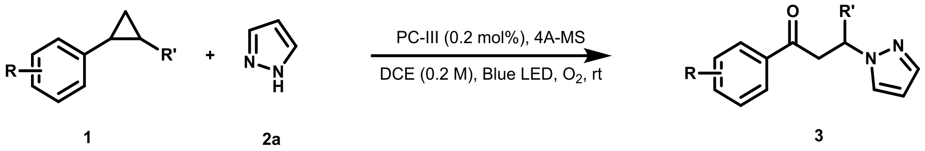

To an oven-dried 10 mL tube equipped with a stir bar, cyclopropanes **1** (0.2 mmol), pyrazole **2a** (0.4 mmol), photocatalyst [Ir(dF(CF_3)ppy)$_2$(4,4'-bpy)](PF_6) (PC-III) (0.5 mg, 0.2 mol%), 4 Å-MS (100 mg) and anhydrous DCE (1.0 mL, 0.2 M) were added and then the reaction tube was capped and charged with O_2 using a balloon, and the resulting mixture was irradiated under 15W blue LEDs at room temperature. When the reaction was determined to be completed by TLC analysis, the mixture was passed through a short pad of celite and rinsed with DCM (20 mL). The filtrate was evaporated to dryness under reduced pressure and the crude residue was purified by column chromatography on silica gel (PE : EtOAc = 9 : 1 to 4 : 1) to afford the desired product **3**.

General Procedure F for One-pot Aminoacylation of Olefins

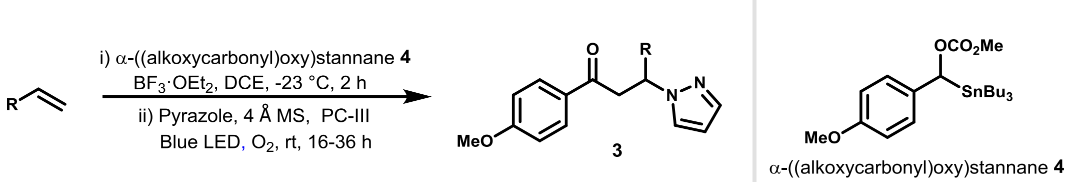

To an oven-dried 50 mL round-bottom flask equipped with a stir bar was added a solution of (4-methoxyphenyl)(tributylstannyl)methyl methyl carbonate (486 mg, 1.0 mmol, 1.0 equiv), alkene substrate (1.1 mmol, 1.1 equiv) and DCE (5.0 mL) under nitrogen atmosphere. The reaction mixture was cooled to -23 ℃ and BF_3 OEt_2 (156 mg, 1.1 mmol, 1.1 equiv) was added by syringe and the resulting solution was stirred at this temperature for an additional 2 h. After the reaction reached completion, as judged by TLC, the reaction mixture was warmed to room temperature, and then pyrazole **2a** (2 mmol, 2.0 equiv), photocatalyst [Ir(dF(CF_3)ppy)$_2$(4,4'-bpy)](PF_6) (PC-III) (2.5 mg, 0.2 mol%), 4 Å-MS (0.5 g) were added. The reaction flask was capped with rubber septum and charged with O_2, then irradiated with 15W blue LEDs at room temperature. When the reaction was determined to be completed by TLC analysis, the mixture was passed through a short pad of celite and rinsed with DCM (20 mL). The

filtrate was evaporated to dryness under reduced pressure and the crude residue was purified by column chromatography on silica gel (PE : EtOAc = 9 : 1 to 4 : 1) to afford the desired product **3**.

Supplementary Discussion

Cyclic Voltammetry

Cyclic voltammograms were taken on a CH Instruments 600E potentiostat using a glassy carbon working electrode, a saturated calomel (SCE) reference electrode, and a Pt mesh counter electrode. The pH was not adjusted and voltammograms were taken at room temperature in a 100 mM MeCN solution of tetrabutylammonium hexafluorophosphate containing 10 mM of the 1-(2,2-dimethylcyclopropyl)-4-methoxybenzene (**1a**). The scan rate was 100 mV/s.

Supplementary Figure 1. Cyclic voltammograms of 1-(2,2-dimethylcyclopropyl)-4-methoxybenzene.

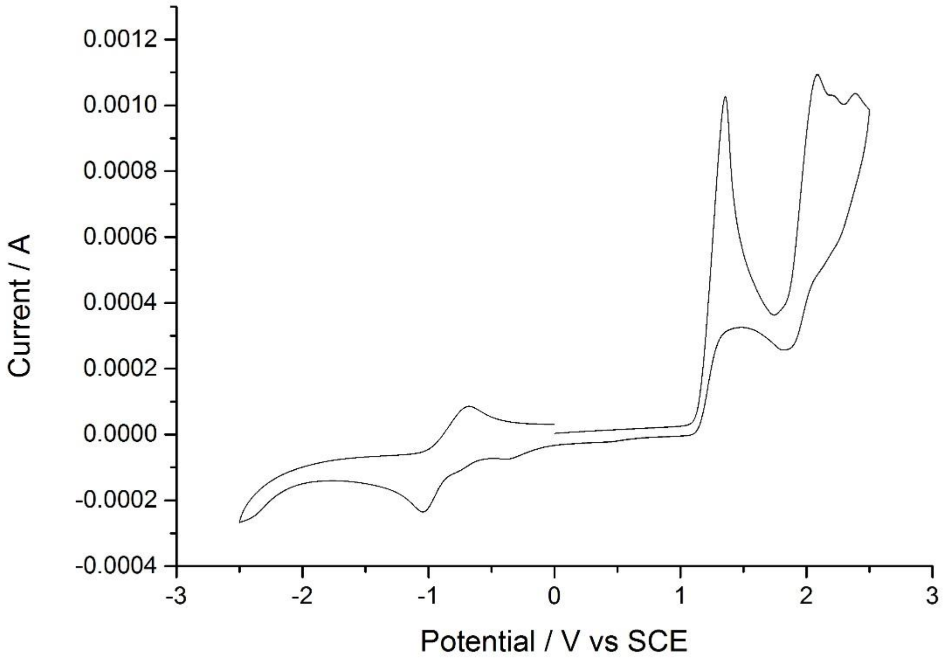

$E_{1/2}{}^{ox}$ (**1a**) = +1.30 V vs. SCE in CH_3CN.

Emission Quenching Experiments (Stern-Volmer Studies)

Emission intensities were recorded using PerkinElemer LS 55 Fluorescence Spectrometer for all experiments. All $[Ir(dF(CF_3)ppy)_2(4,4'\text{-}bpy)](PF_6)$ solutions (0.01 mM) were excited at 389 nm and the emission intensity at 575 nm was collected at room temperature under an **N_2 atmosphere**. Samples were prepared by adding solutions of photocatalyst, quencher, and DCE to obtain a total volume of 3.0 mL under air atmosphere. The sample was shaken for 1 min and then the emission of the sample was collected. The data show that 1-(2,2-dimethylcyclopropyl)-4-methoxybenzene **1a** is competent at quenching the excited state of the photocatalyst, while Parazole **2a** is shown to be unable to quench this excited state.

Constant Iridium, Varied 1a.

Species			Concentration (mM)		
$[Ir(dF(CF_3)ppy)_2(4,4'\text{-}(dCF_3)bpy)](PF_6)$			0.005 mM		
**1a**			Varied		
[**1b**] (mM)	Scan 1	Scan 2	Scan 3	Average	I_0/I
0	658.63	655.76	657.15	657.18	1.00
1	218.99	215.43	216.27	216.90	3.03
2	136.55	134.68	135.33	135.52	4.85
3	110.37	112.01	110.03	110.80	5.93
6	61.60	62.58	62.79	62.32	10.54
9	48.84	46.16	47.69	47.56	13.82
12	35.37	35.59	35.27	35.41	18.56

Constant Iridium, Varied 2a.

Species			Concentration (mM)		
$[Ir(dF(CF_3)ppy)_2(4,4'\text{-}(dCF_3)bpy)](PF_6)$			0.005 mM		
**2a**			Varied		
[**2a**] (mM)	Scan 1	Scan 2	Scan 3	Average	I_0/I
0	658.63	655.76	657.15	657.18	1.000
3	643.68	642.77	643.75	643.4	1.021
6	670.7	667.31	660.04	666.02	0.987
9	659.88	662.74	660.04	660.89	0.994
12	630.19	631.04	628.01	629.74	1.043

Supplementary Figure 2. Emission intensities record under N_2 atmosphere.

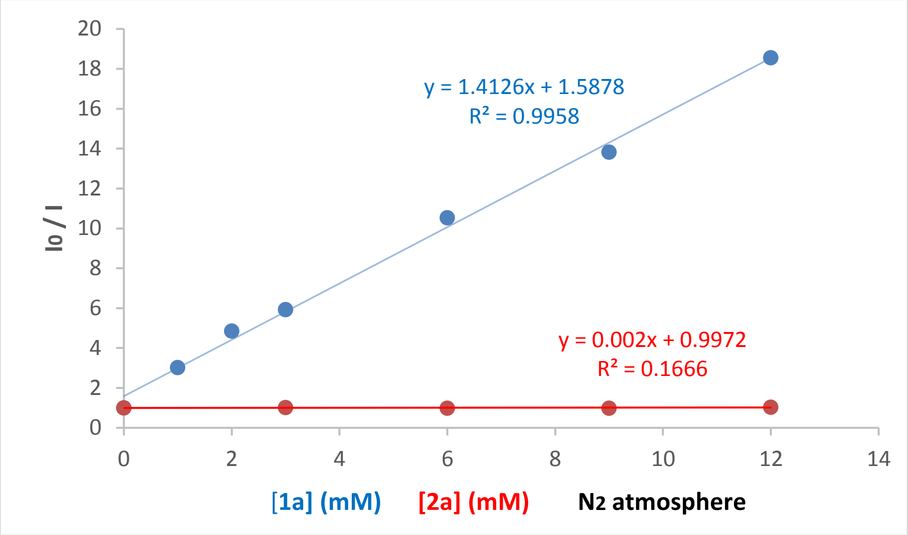

Emission intensities were recorded using PerkinElemer LS 55 Fluorescence Spectrometer for all experiments. All [Ir(dF(CF_3)ppy)$_2$(4,4'-bpy)](PF_6) solutions (0.01 mM) were excited at 389 nm and the emission intensity at 575 nm was collected at room temperature under an **air atmosphere**. Samples were prepared by adding solutions of photocatalyst, quencher, and DCE to obtain a total volume of 3.0 mL under air atmosphere. The sample was shaken for 1 min and then the emission of the sample was collected. The data show that 1-(2,2-dimethylcyclopropyl)-4-methoxybenzene **1a** is competent at quenching the excited state of the photocatalyst, while Parazole **2a** is shown to be unable to quench this excited state.

Constant Iridium, Varied 1a.

Species	Concentration (mM)
[Ir(dF(CF_3)ppy)$_2$(4,4'-(dCF_3)bpy)](PF_6)	0.005 mM
**1a**	Varied

[**1b**] (mM)	Scan 1	Scan 2	Scan 3	Average	I_0/I
0	526.41	528.58	524.1	526.36	1.00
1	212.68	212.22	212.33	212.41	2.48
2	123.24	123.79	122.7	123.24	4.27
3	102.38	104.33	102.19	102.97	5.11
6	53.73	57.17	55.34	55.41	9.50
9	43.86	44.38	43.55	43.93	11.99
12	30.68	30.33	30.61	30.54	17.23

Constant Iridium, Varied 2a.

Species	Concentration (mM)
$[Ir(dF(CF_3)ppy)_2(4,4'-(dCF_3)bpy)](PF_6)$	0.005 mM
**2a**	Varied

[**2a**] (mM)	Scan 1	Scan 2	Scan 3	Average	I_0/I
0	634.85	631.77	638.29	634.97	1.000
3	646.42	635.24	637.89	639.85	0.992
6	601.63	593.72	593.89	596.41	1.064
9	609.18	607.83	605.89	607.63	1.045
12	630.19	631.04	628.01	629.75	1.008

Supplementary Figure 3. Emission intensities record under air atmosphere.

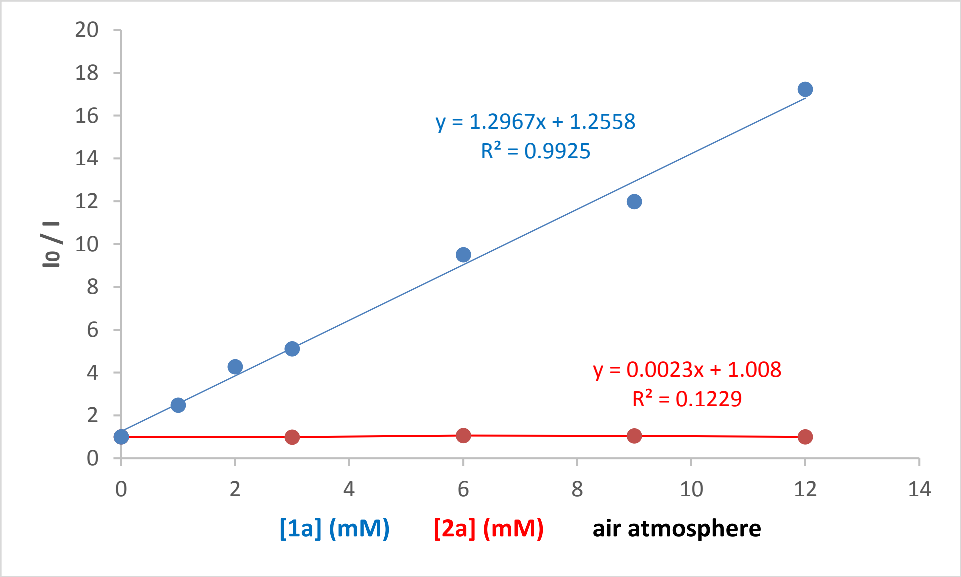

Light On/Off Experiment

To an over dried 5 mm NMR tube equipped with a stir bar, were added **1b** (0.2 mmol), **2a** (0.4 mmol), $[Ir(dF(CF_3)ppy)_2(4,4'-bpy)](PF_6)$ (PC-III) (0.5 mg, 0.2 mol%) and anhydrous DCE (1.0 mL, 0.2 M) in air. The NMR tube was capped and charged with O_2 using a balloon. The resulting mixture was subjected to alternating intervals of irradiation with blue light and dark. The reaction profile is shown below and the yield of product **3ba** as a function of time was determined by ^{1}H-NMR using 1,1,2,2-tetrachloroethane as an internal standard. These results indicated that continuous irradiation with light was essential for promoting the reaction.

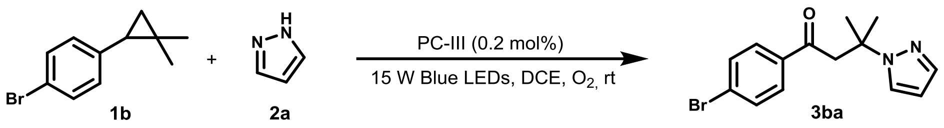

Supplementary Figure 4. Light on/off experiment of reaction.

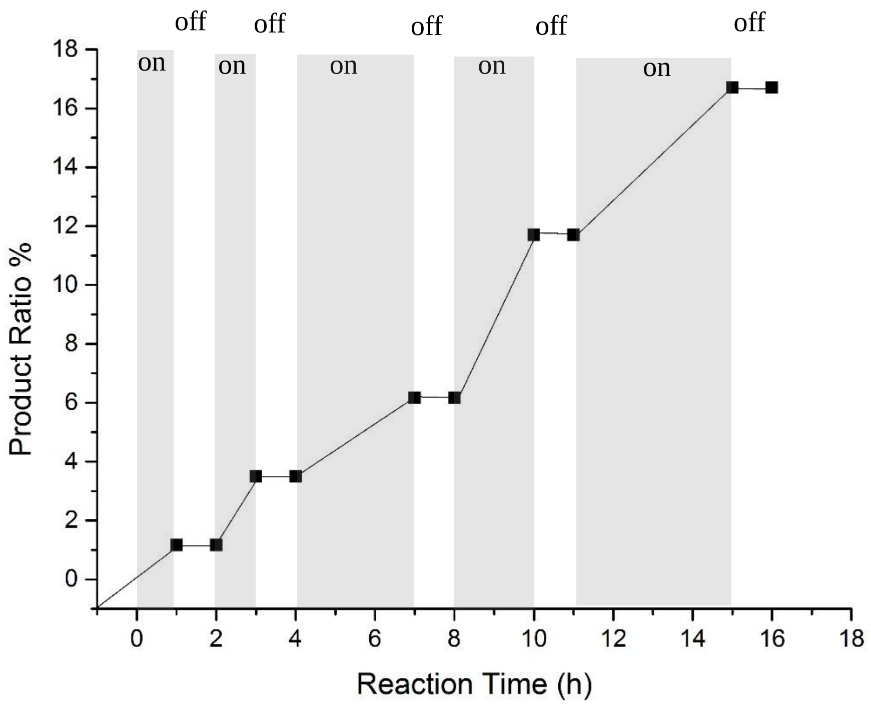

Determination of the Reaction Quantum Yield (Φ)

Supplementary Figure 5. Emission spectrum of blue LED used for quantum yield experiments.

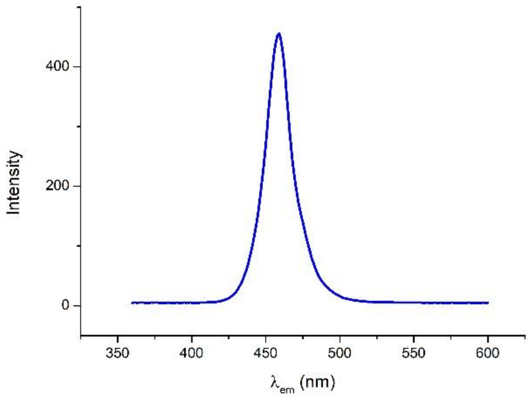

Recorded using a F-4600 FL Spectrophotometer (λ_{max}= 459 nm).

Determination of the Light Intensity at 459 nm

Following a modified procedure reported by Melchiorre and co-workers,[16] an aq. ferrioxalate actinometer solution was prepared and stored in the dark. The actinometer

solution measures the photodecomposition of ferric oxalate anions to ferrous oxalate anions, which are then reacted with 1,10-phenanthroline to form $Fe(Phen)_3^{2+}$. Its concentration is then estimated by UV/Vis absorbance at 510 nm. The number of moles of $Fe(Phen)_3^{2+}$ complex formed is related to the numbers of photons absorbed by the actinometer solution. Preparation of the solutions used for the studies:

1. Potassium ferrioxalate solution: Potassium ferrioxalate trihydrate (118 mg) and 95-98% H_2SO_4 (56 μL) were added to a 20 mL volumetric flask and filled to the mark with distilled water.

2. Buffer solution: Sodium acetate (0.988 g) and 95-98% H_2SO_4 (0.2 mL) were added to a 20 mL volumetric flask and filled to the mark with distilled water.

The actinometry measurements:

a) 1 mL of the actinometer solution was taken in a quartz cuvette (l = 1 cm). Both the cuvettes of actinometer solution and reaction solution were placed next to each other at a distance of 5 cm away from a 15 W blue LED (λmax = 459 nm) and irradiated for 30 s. The same process was repeated for different time intervals: 60 and 90 s.

b) After irradiation, the actinometer solution was transferred to a 10 mL volumetric flask containing 1.0 mg of 1,10-phenanthroline in 2 ml of buffer solution. The flask was filled to the mark with distilled water. In a similar manner, a blank solution (10 mL) was also prepared using the actinometer solution stored in dark.

c) Absorbance of the actinometer solution after complexation with 1,10-phenanthroline at λ = 510 nm was measured by UV/Vis spectrophotometry.

d) According to Beer's law, the number of moles of Fe^{2+} formed (x) for each sample was determined by Supplementary Equation 1:

$$\text{mol Fe}^{2+} = \frac{\text{v1}\cdot\text{v3}\cdot\Delta\text{A}(510\text{nm})}{1000\cdot\text{v2}\cdot\text{l}\cdot\varepsilon(510\text{nm})} \quad (1)$$

Where:

v1 = Irradiated volume (1 mL).

v2 = The aliquot of the irradiated solution taken for the estimation of Fe+ ions (1 mL).

v3 = Final volume of the solution after complexation with 1,10-phenanthroline (10 mL).

ε(510 nm) =Molar extinction coefficient of $[Fe(Phen)_3]^{2+}$complex (11100 L $mol^{-1}cm^{-1}$).

l = Optical path-length of the cuvette (1 cm).

ΔA(510 nm) = Difference in absorbance between the irradiated solution and the solution stored in dark (blank).

e) The number of moles of Fe^{2+} formed (x) was plotted as a function of time (t). The slope (dx/dt) of the line is equal to the number of moles of Fe^{2+} formed per unit time.

f) This slope (dx/dt) was correlated to the number of moles of incident photons per unit time (F = photon flux) by using Supplementary Equation 2:

$$\Phi(\lambda) = \frac{\frac{dx}{dt}}{F \cdot (1 - 10^{-A(\lambda)})} \qquad (2)$$

Φ(λ) = The quantum yield for Fe^{2+} formation at 450 nm is 0.9.[17]

g) A(λ) = Absorbance of the ferrioxalate actinometer solution at a wavelength of 459 nm, which was measured placing 1 mL of the solution in a cuvette of pathlength 1 cm by UV/Vis spectrophotometry.

Sample calculation according to Supplementary Equation 1:

$A^0 = 0.023$, $A^1_{30S} = 0.772$, $A^1_{60S} = 1.319$, $A^1_{90S} = 1.918$

$\Delta A^1_{30S} =$ 0.749 $\Delta A^1_{60S} =$ 1.296 $\Delta A^1_{90S} =$ 1.895

mol Fe^{2+} (30 s) = (1 mL ×10 mL ×0.749)/(1000 ×1 mL ×1 cm ×111100 L mol^{-1} cm^{-1})

= 6.748×10^{-7} mol

mol Fe^{2+} (60 s) = (1 mL ×10 mL ×1.296)/(1000 ×1 mL ×1 cm ×111100 L mol^{-1} cm^{-1})

= 1.1676×10^{-6} mol

mol Fe^{2+} (90 s) = (1 mL ×10 mL ×1.895)/(1000 ×1 mL ×1 cm ×111100 L mol^{-1} cm^{-1})

= 1.7072×10^{-6} mol

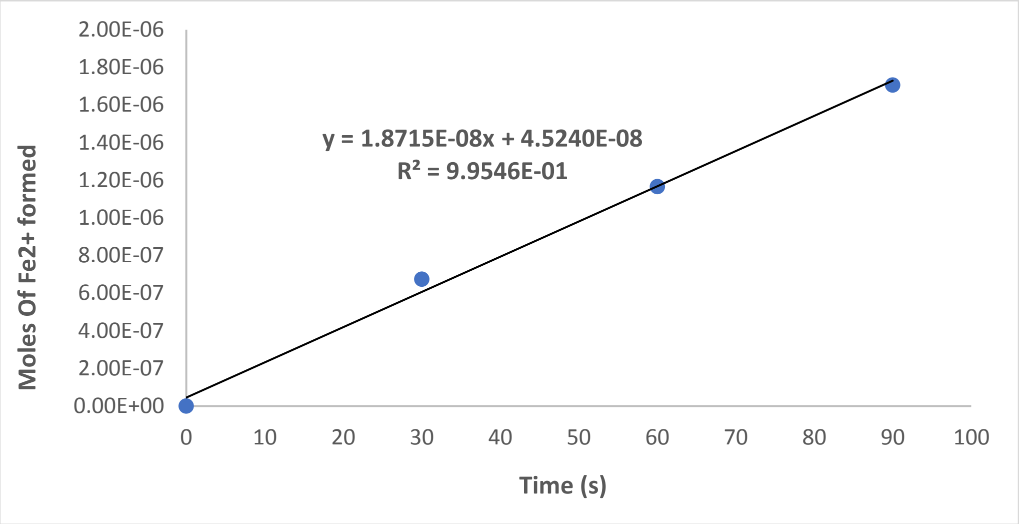

Supplementary Figure 6. Moles of Fe^{2+} formed being plotted as a function of time.

Moles of $[Fe(Phen)_3]^{2+}$ per unit of time formed due to decomposition of the actinometer solution at 459 nm blue Led irradiation as shown in Supplementary Equation 3:

$$F = \frac{\frac{dx}{dt}}{\Phi(\lambda)\cdot(1-10^{-A(\lambda)})} \qquad (3)$$

$A(\lambda)_{30S} = 0.649$, $A(\lambda)_{60S} = 1.134$, $A(\lambda)_{90S} = 1.638$

$F_{30s} = (1.8715\times10^{-8})/(0.9\times(1-10^{-0.649})) = 2.681\times10^{-8}$

$F_{60s} = (1.8715\times10^{-8})/(0.9\times(1-10^{-1.134})) = 2.244\times10^{-8}$

$F_{90s} = (1.8715\times10^{-8})/(0.9\times(1-10^{-1.638})) = 2.128\times10^{-8}$

$F_{everage} = (F_{30s} + F_{60s} + F_{90s})/3 = 2.351\times10^{-8}$

h) The determined incident photons per unit time (F) is 2.351×10^{-8} einsteins/s.

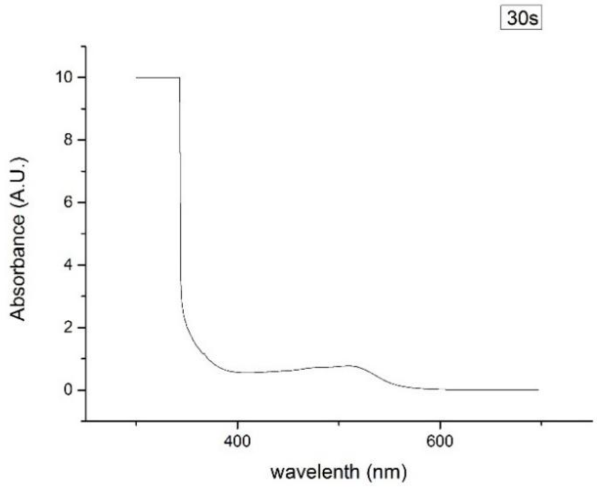

Supplementary Figure 7. Absorbance of the 30 seconds irradiated ferrioxalate actinometer solution.

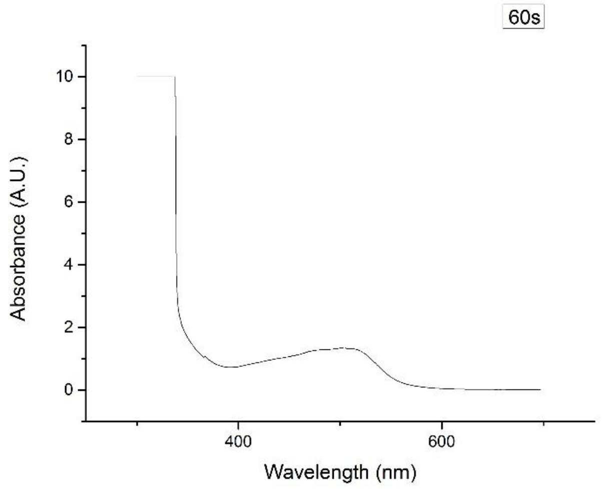

Supplementary Figure 8. Absorbance of the 60 seconds irradiated ferrioxalate actinometer solution.

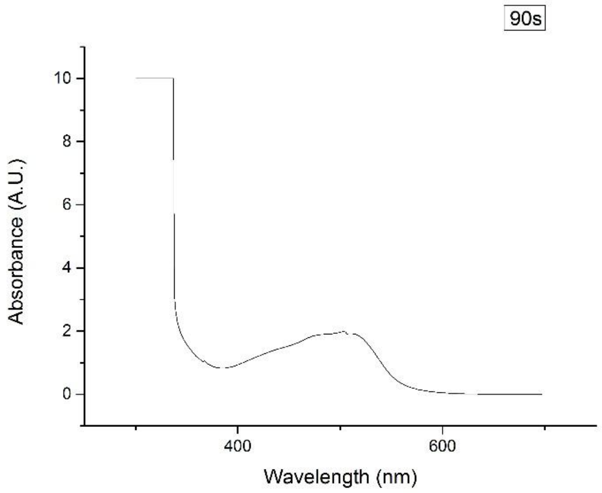

Supplementary Figure 9. Absorbance of the 90 seconds irradiated ferrioxalate actinometer solution.

Determination of the Reaction Quantum Yield

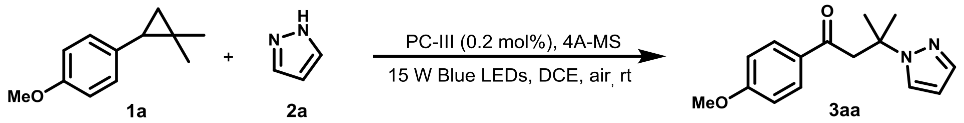

To a 3 mL quartz cuvette with two sides taped over with electrical tape, **1a** (35 mg, 0.2 mmol, 1 equiv), **2a** (27 mg, 0.4 mmol, 2 equiv), [Ir(dF(CF_3)ppy)$_2$(4,4'-bpy)](PF_6) (PC-III) (1.0 mg, 0.2 mol%) and anhydrous DCE (1.0 mL, 0.2 M), 4Å-MS (100 mg) and a small stir bar were added and then the quartz cuvette was capped and charged with O_2 using a balloon. The sample was stirred and irradiated for 10800 s (3.0 h) at λ_{max} = 459 nm at rt. After irradiation, the yield of product **3aa** was determined to be 16.6% (3.32

× 10^{-5} mol of **3aa**) by ^{1}H NMR integration against an internal standard. The reaction quantum yield (Φ) was determined using the Supplementary Equation 4 where the photon flux is 2.35×10^{-8} einstein s^{-1} (described above), t is the reaction time (10800 s) and f is the fraction of incident light absorbed by the reaction mixture. An absorbance of the reaction mixture at 459 nm was measured to be 1.426

$$\Phi = \frac{\text{mol of product formed}}{\text{photon flux} \cdot \text{t} \cdot \text{f}} \qquad (4)$$

Sample quantum yield calculation accoeding to Supplementary Equation 4:

$f = 1\text{-}10^{(-1.426)} = 0.9625$

$\Phi = 3.32 \times 10^{-5}$ mol/(2.351×10^{-8} einstein s^{-1} ×10800 s ×0.9625) = 0.14

The reaction quantum yield (Φ) was thus determined to be 0.14.

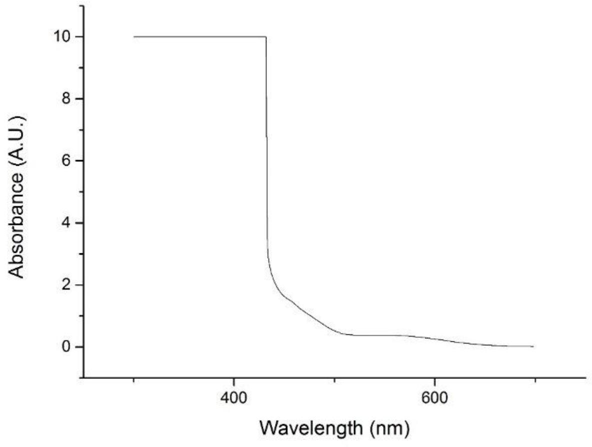

Supplementary Figure 10. Absorbance of the reaction mixture solution.

Radical Inhibition Experiment with TEMPO

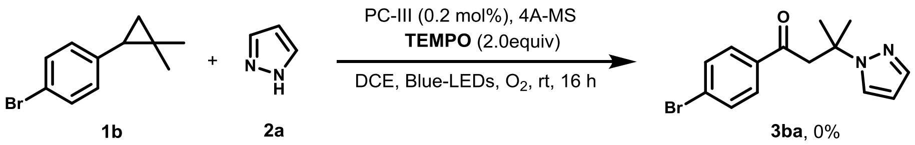

To an oven-dried 10 mL Schlenk tube equipped with a stir bar, **1b** (0.2 mmol), **2a** (0.4 mmol), $[Ir(dF(CF_3)ppy)_2(4,4'\text{-bpy})](PF_6)$ (PC-III) (1.0 mg), 4Å-MS (100 mg), TEMPO (0.4 mmol) and dry DCE (1.0 mL) were added under air. The reaction flask was capped with a rubber septum, charged with O_2, and the resulting mixture was irradiated under 15W blue LEDs for 16 h. The reaction solution was monitored by TLC, which showed no desired product formation.

Evidence of Alkyl Hydroperoxide Intermediate

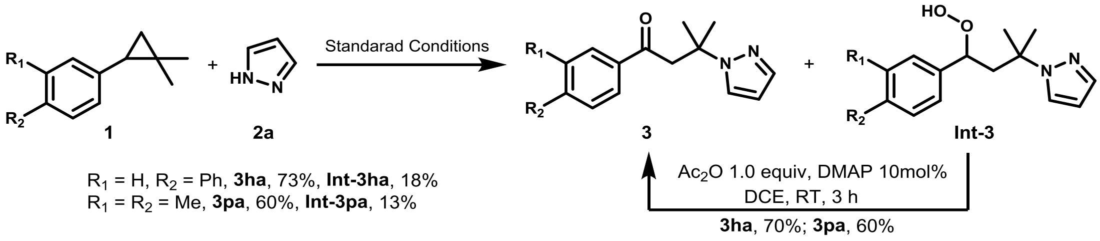

To an oven-dried 10 mL Schlenk tube equipped with a stir bar was added **1** (0.2 mmol), **2a** (0.4 mmol), [Ir(dF(CF$_3$)ppy)$_2$(4,4'-bpy)](PF$_6$) (PC-III) (0.5 mg), 4Å-MS (100 mg) and dry DCE (1.0 mL) under air at room temperature. The reaction flask was capped with a rubber septum, charged with O_2 using a balloon, and the resulting mixture was irradiated under 15 W blue LEDs for 24 h. Upon completion of the reaction, the mixture was passed through a short pad of celite. The filtrate was concentrated and purified by column chromatography on silica gel (PE : EtOAc = 9 : 1 to 4 : 1). In addition to the desired products (PE : EtOAc = 4 : 1, $Rf^{\mathbf{3ha}}$ = 0.55, $Rf^{\mathbf{3pa}}$ = 0.40), the hydroperoxide intermediates **Int-3ha** (PE : EtOAc = 4 : 1, $Rf^{\mathbf{Int\text{-}3ha}}$ = 0.35, 18% isolated yield) and **Int-3pa** (PE : EtOAc = 4 : 1, $Rf^{\mathbf{Int\text{-}3pa}}$ = 0.25,13% isolated yield) were isolated. To a flask with a stir bar, **Int-3ha**/**Int-3pa** was added dimethylaminopyridine (DMAP) (10 mol%), Ac_2O (1.0 equiv) and DCE (0.5 mL). The reaction mixture was stirred at rt for 3 h after which TLC showed all of the starting material was consumed. The reaction mixture was diluted with ethyl acetate (20 mL) and brine (20 mL). The phases were separated and the aqueous layer was extracted with EtOAc (20 mL ×2). The combine organic layers were dried over anhydrous Na_2SO_4, filtered and concentrated under reduced pressure. The product was separated from the residue by column chromatography on silica gel ((PE : EtOAc = 4 : 1 , Rf3ha = 0.55, 70% yield; Rf3pa = 0.40, 60% yield), and the structure of the obtained compound was confirmed to be identical to **3ha**/**3pa** by NMR.

Ruling Out the Intermediacy of 1,2-Dioxolane

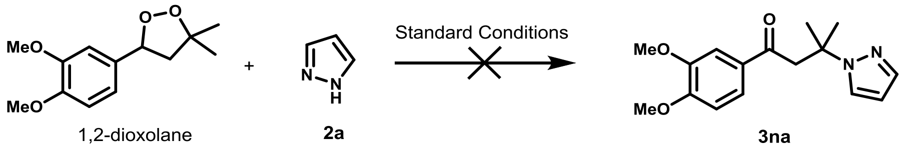

To an oven-dried 10 mL tube equipped with a stir bar, 1,2-dioxolane[15] (10 mg, 0.04 mmol), pyrazole **2a** (6.8 mg, 0.08 mmol), photocatalyst $[Ir(dF(CF_3)ppy)_2(4,4'$-bpy)](PF_6) (PC-III) (0.1 mg, 0.2 mol%), 4Å-MS (50 mg) and anhydrous DCE (0.5 mL, 0.1 M) were added. The reaction tube was capped and charged with O_2, and the resulting mixture was irradiated under 15W blue LEDs at room temperature for 16 h. The reaction was monitored by TLC. TLC analysis indicated that all 1,2-dioxolane was consumed but no product **3na** was detected.

The Examination of Reactions Between Pyrazole and 1ak, 1ba and 1bb

Following the General Procedure E, **3aka** and **5aka** was obtained from **1ak** in 22%, 35% yields, respectively, **6baa** was obtained from **1ba** in 30% yield and **5bba** was obtained from **1bb** in 50% yield.

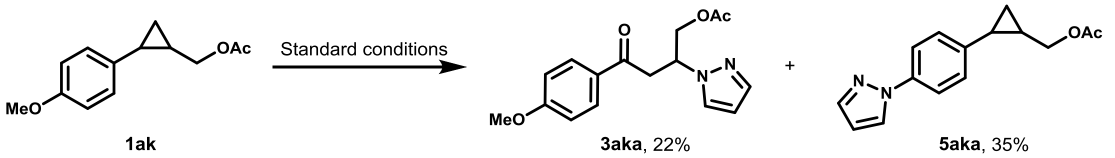

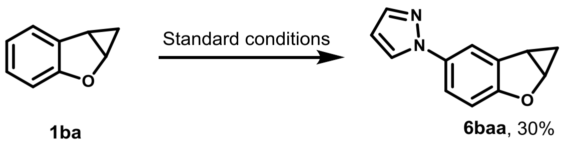

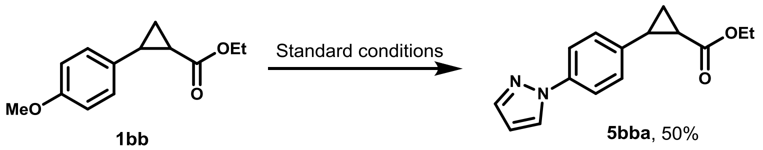

As we can obtain the desired products of to **3aka**, the SET oxidation of respective substrates for the generation of radical cation intermediates therefore is accomplished. The present reaction predominately proceeded through a concerted nucleophilic attack/ring-opening manifold, and the positive charge is believed to be delocalized over the whole molecule, not only the aryl moiety but also the cyclopropyl ring system. Therefore, the installation of electronically negative substituent proximal to the cyclopropyl ring would inevitably affect the charge distribution, thus making the carbon atom of cyclopropane proximal to those electron-withdrawing functionalities less positively charged and in turn more reluctant to undergo nucleophilic attack by azaarenes. Furthermore, in addition to the formation of desired product **3aka**, the

demethoxyamination product **5aka** was also obtained in 35% yield in the reaction of **1ak**. In this case, the nucleophilic aromatic substitution by azaarene compares favorably with ring-opening functionalization of cyclopropane ring because of the proximity of –OAc functionality.

Furthermore, when **1ba** (1a,6b-dihydro-1H-cyclopropa[b]benzofuran) was subjected to the standard reaction conditions, only homolytic aromatic substitution product **6baa** was obtained in 30% yield with no any ring-opening oxo-amination being observed. This phenomenon is consistent with the observation of Nicewicz that in the case of photoredox catalyzed hemolytic aromatic substitution of electron-rich arene derivatives, the nucleophile tends to attack the para-position of arenes with electron-donating substituents (*J. Am. Chem. Soc.* **2017**, *139*, 11288). Therefore, the experimental result from **1ba** indicates that after one electron oxidation the positive charge reside mainly on the aryl ring, with the cyclopropyl ring embed in oxo-bicyclic system poorly participated in charge delocalization, probably because of poor orbit overlap.

In the case of **1bb**, the reaction selectively underwent nucleophilic aromatic substitution to afford **5bba** in 50% yield.

The Examination of Aza-Nucleophiles with Different Oxidation Potentials

The prerequisite for the success of this reaction is the efficient and selective SET oxidation of aryl cyclopropane substrates, therefore, the selected aza-nucleophile should tolerate the oxidation potentials that enable smooth SET oxidation of cyclopropane derivatives. On the other hand, the success of ring opening of cyclopropane radical cation intermediate is also directly affected by the nucleophilicity of azaarenes. This issue we faced is that nucleophiles with high nucleophilicity are always more easily undergo SET oxidation. We have tested a set of aza-nucleophiles with varying oxidation potentials and found that nucleophiles with oxidation potentails lower than aryl cyclopropane substrate are not viable in the present reaction.

Supplementary Table 9: Reaction results of different Aza-nucleophiles.

Aza-nucleophiles	Oxidation potentials vs. SCE	Reaction results
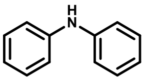	+0.92 V	No reaction
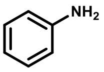	+0.95 V	No reaction
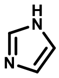	+1.15 V	No reaction
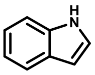	+1.16 V	No reaction
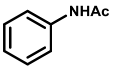	+1.70V	No desired product
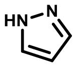	+2.21 V	Reaction succeeded
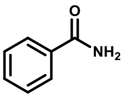	+2.30V	No desired product
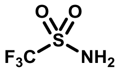	> +2.5 V	Reaction succeeded
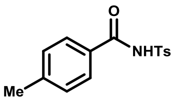	> +2.5 V	No desired product
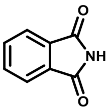	> +2.5 V	No desired product

Experiment with Enantiometically Enriched Diphenyl Cyclopropane

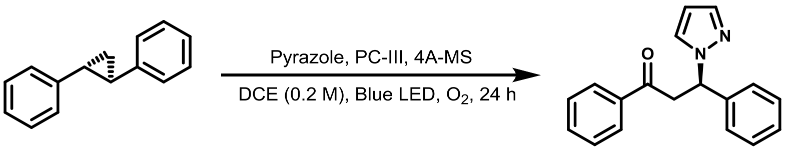

1ao (1R, 2R) 90% ee　　**3aoa** (R), 65% yield, 60% ee

The reaction of **1ao** with 90% ee led to the generation of the product **3aoa** in 65% yield with 60% ee after 24 h. This result indicates that the oxo-amination maninly proceed through a concerted nucleophilic attack/ring-opening fashion (S_N2-like process), while the erosion of stereochemistry could potentially derive from minor contribution of ring-opening followed by nucleophilic attack manifold (S_N1-like process). However, the possibility of racemization of enantiomerically enriched diphenyl cyclopropane substrate through a cascade of sensitization via triplet energy transfer, homolytic ring cleavage and radical recombination-based ring closure should also be considered. To

unravel this possibility, the reaction of **(1R,2R)-1ao** was monitored, which clearly demonstrated that cyclopropane substrate underwent racemization under the present reaction conditions.

Supplementary Figure 11. The Evolvement of ee Values of Substrate **(1R,2R)-1ao** and Product **(R)-3aoa** as the Reaction Progresses.

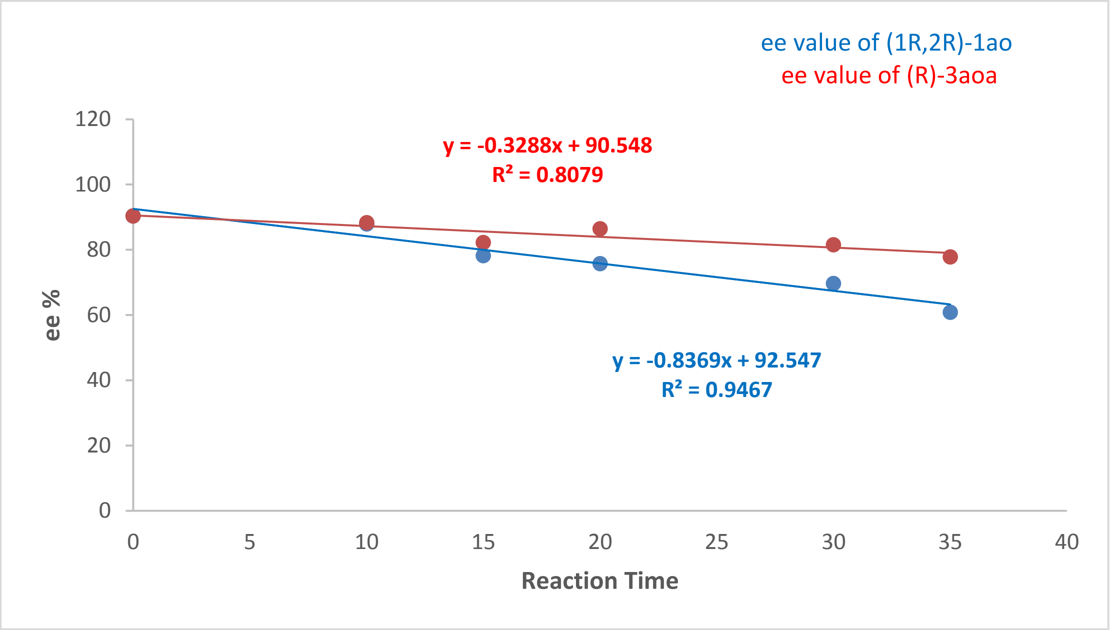

HPLC Chromatography of the Starting Materials and Products

Supplementary Figure 12. HPLC Chromatography of Racemic 1,2-diphenylcyclopropane (**1ao**) (Chiralpak AD-H 250*4.6 mm/5 um column, 5% isopropanol in hxane, 1.0 mL/min, 210 nm)

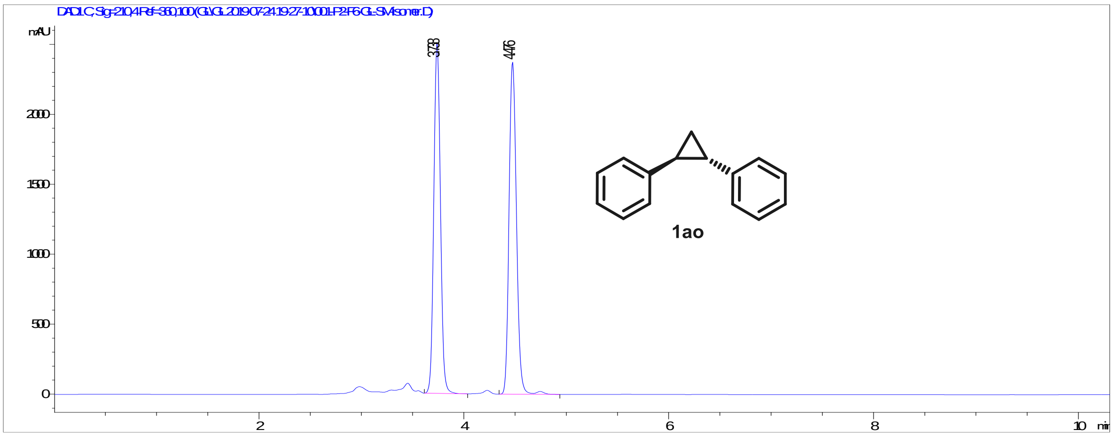

Peak #	RetTime [min]	Type	Width [min]	Area [mAU*s]	Height [mAU]	Area %
1	3.738	BB	0.0662	11193.6	2503.8	48.325
2	4.476	BV R	0.0731	11969.7	2376.9	51.675
Total				7108.9	1574.3	100%

Supplementary Figure 13. HPLC Chromatography of (1R,2S)-1,2-diphenylcyclopropane (**cis-1ao**) (Chiralpak AD-H 250*4.6 mm/5 um column, 5% isopropanol in hxane, 1.0 mL/min, 210 nm)

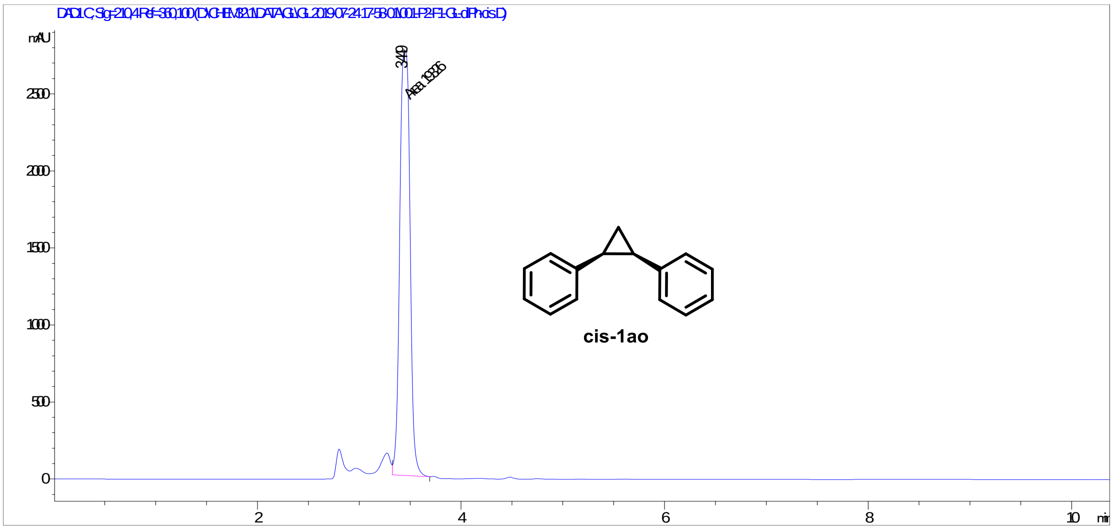

Peak #	RetTime [min]	Type	Width [min]	Area [mAU*s]	Height [mAU]	Area %
1	3.449	FM	0.1169	19326	2754.3	100.00
Total				19326	2754.3	100%

Supplementary Figure 14. HPLC Chromatography of (1R,2R)-1,2-diphenylcyclopropane (**(1R,2R)-1ao**).

(Chiralpak AD-H 250*4.6 mm/5 um column, 5% isopropanol in hxane, 1.0 mL/min, 210 nm)

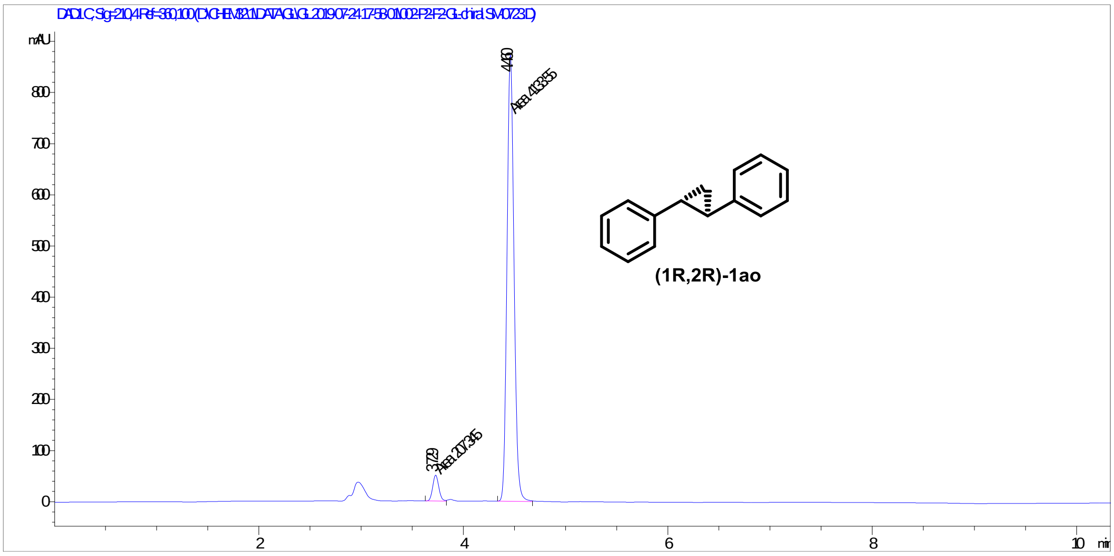

Peak #	RetTime [min]	Type	Width [min]	Area [mAU*s]	Height [mAU]	Area %
1	3.729	MF	0.0681	207.3	50.8	4.777
2	4.46	MF	0.0786	4133.5	876.8	95.223
Total				4340.8	927.6	100%
						(ee 90.446%)

Supplementary Figure 15. HPLC Chromatography of Racemic 1,3-diphenyl-3-(1H-pyrazol-1-yl)propan-1-one (**3aoa**)

(Chiralpak AD-H 250*4.6 mm/5 um column, 5% isopropanol in hxane, 1.0 mL/min, 210 nm)

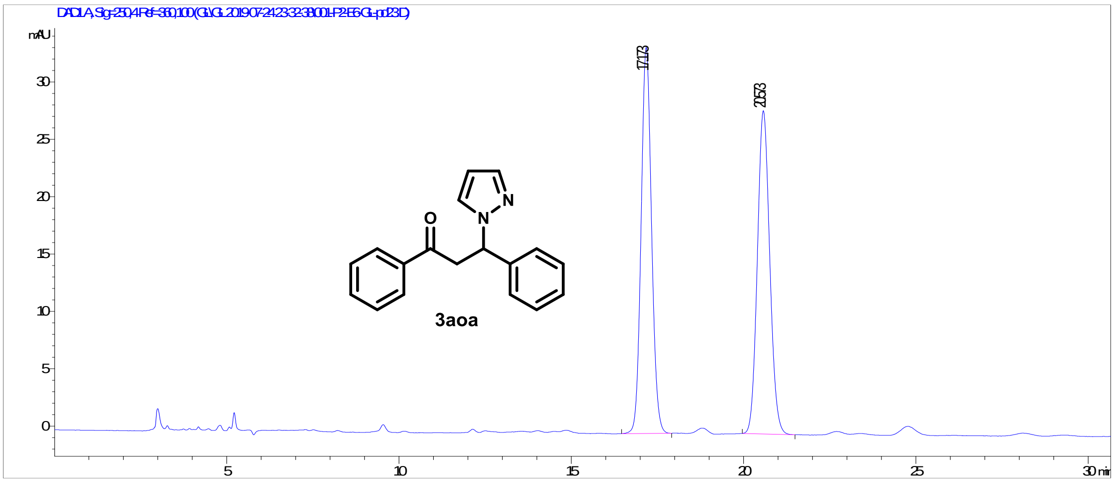

Peak #	RetTime [min]	Type	Width [min]	Area [mAU*s]	Height [mAU]	Area %
1	17.173	BB	0.3237	708.7	33.7	50.191
2	20.573	BB	0.3832	703.3	28.2	49.809
Total				1412	61.9	100%

Supplementary Figure 16. HPLC Chromatography of Reaction solution of 10 min.

(Chiralpak AD-H 250*4.6 mm/5 um column, 5% isopropanol in hxane, 1.0 mL/min, 210 nm)

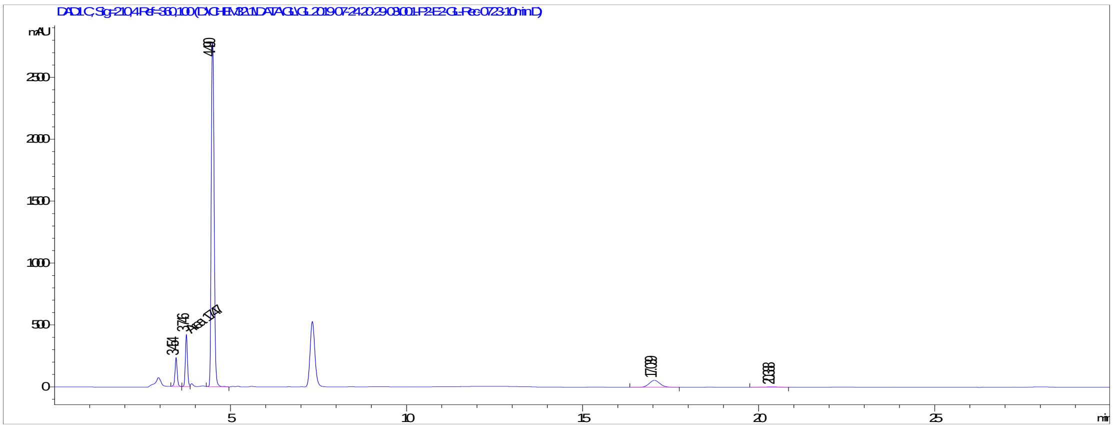

Peak #	RetTime [min]	Type	Width [min]	Area [mAU*s]	Height [mAU]	Area %
1	3.454	BB	0.0666	1014.4	233.9	5.098
2	3.746	MF	0.0689	1747	422.8	8.779
3	4.49	VV R	0.0906	15887.7	2781.7	79.838
4	17.039	BB	0.3239	1178.1	56.3	5.920
5	20.388	BB	0.3855	72.8	2.8	0.366
Total				19900	3497.5	100%

((**1R,2R)-1ao** ee 87.99%)

((**R)-3aoa** ee 88.36%)

Supplementary Figure 17. HPLC Chromatography of Reaction solution of 15 min.
(Chiralpak AD-H 250*4.6 mm/5 um column, 5% isopropanol in hxane, 1.0 mL/min, 210 nm)

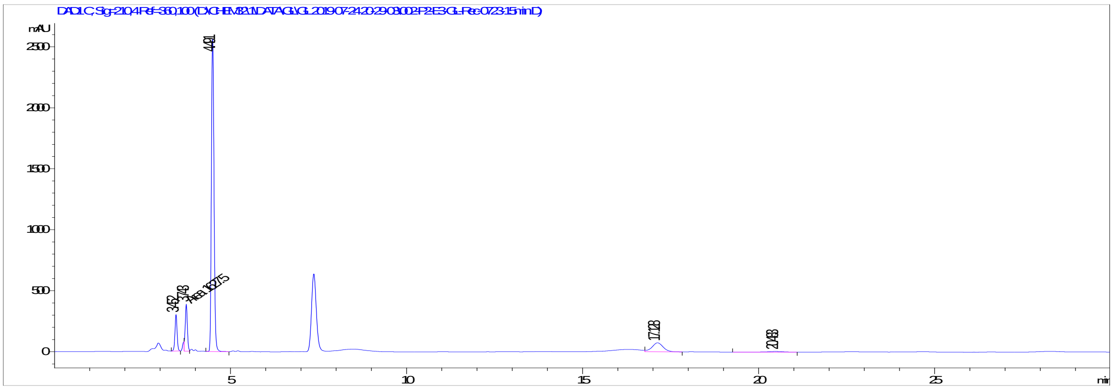

Peak #	RetTime [min]	Type	Width [min]	Area [mAU*s]	Height [mAU]	Area %
1	3.452	BB	0.0649	1258.4	300.5	6.968
2	3.743	MF	0.0704	1627.5	385.3	9.012
3	4.491	VV R	0.0826	13373	2573.2	74.049
4	17.128	BB	0.3367	1641.1	74	9.087
5	20.483	BB	0.4442	159.8	5.1	0.885
Total				18059.8	3338.1	100%

((**1R,2R)-1ao** ee 78.30%)

((**R)-3aoa** ee 82.25%)

Supplementary Figure 18. HPLC Chromatography of Reaction solution of 20 min.
(Chiralpak AD-H 250*4.6 mm/5 um column, 5% isopropanol in hxane, 1.0 mL/min, 210 nm)

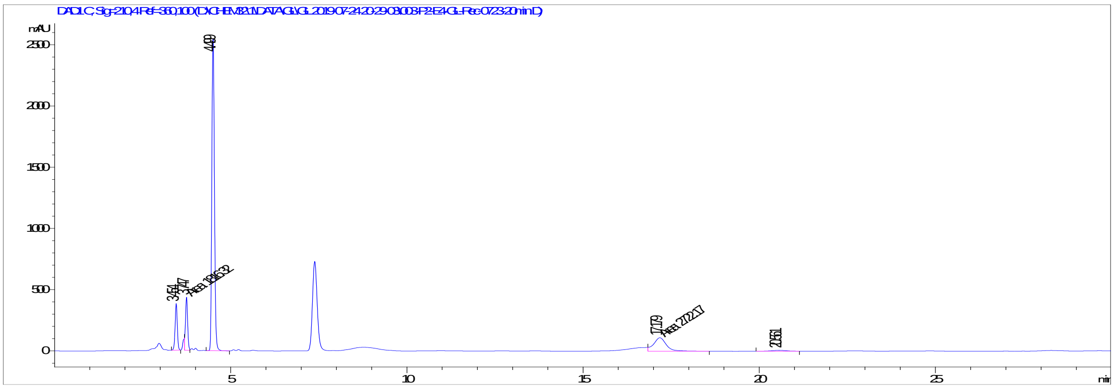

Peak #	RetTime [min]	Type	Width [min]	Area [mAU*s]	Height [mAU]	Area %
1	3.454	BB	0.0665	1649.7	381.1	8.414
2	3.747	FM	0.0697	1816.3	434.5	9.263
3	4.499	VV R	0.0823	13223.4	2557.6	67.439
4	17.179	FM	0.4143	2722.2	109.5	13.883
5	20.561	BB	0.4114	196.2	7.2	1.001
Total				19607.8	3489.9	100%

((**1R,2R)-1ao** ee 75.84%)

((**R)-3aoa** ee 86.55%)

Supplementary Figure 19. HPLC Chromatography of Reaction solution of 30 min.
(Chiralpak AD-H 250*4.6 mm/5 um column, 5% isopropanol in hxane, 1.0 mL/min, 210 nm)

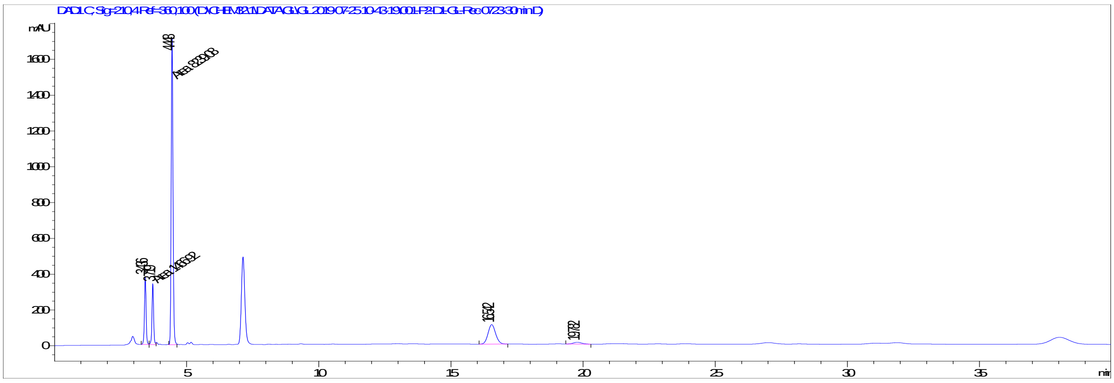

Peak #	RetTime [min]	Type	Width [min]	Area [mAU*s]	Height [mAU]	Area %
1	3.436	BV	0.0663	1632.2	378.6	11.894
2	3.719	MF	0.0717	1466.9	340.9	10.689
3	4.448	MF	0.0797	8239.1	1721.9	60.038
4	16.542	BB	0.3083	2165.8	108.8	15.782
5	19.782	BB	0.3551	219.1	9.7	1.596
Total				13723.1	2559.9	100%

((**1R,2R)-1ao** ee 69.77%)
((**R)-3aoa** ee 81.63%)

Supplementary Figure 20. HPLC Chromatography of Recovered **(1R,2R)-1ao** after 35 min
(Chiralpak AD-H 250*4.6 mm/5 um column, 5% isopropanol in hxane, 1.0 mL/min, 210 nm)

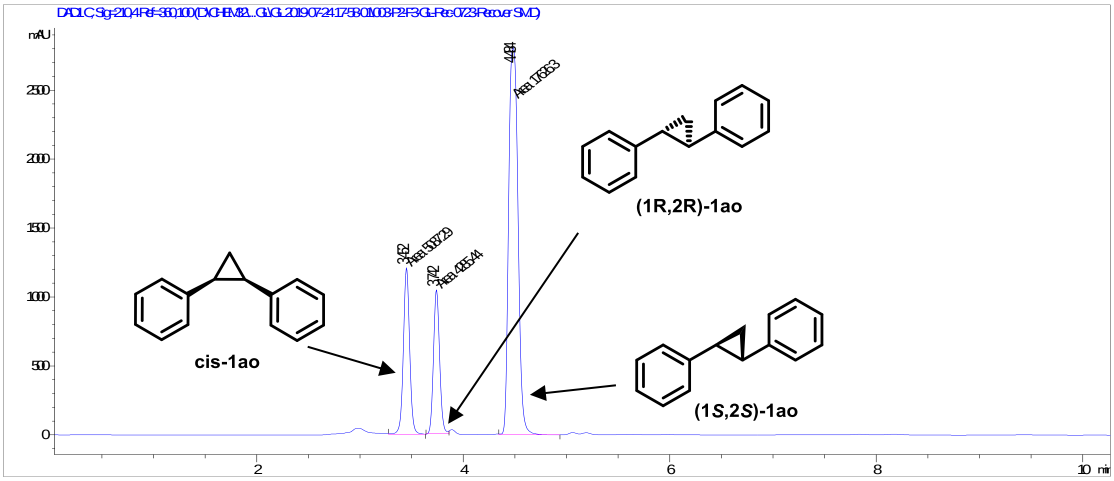

Peak #	RetTime [min]	Type	Width [min]	Area [mAU*s]	Height [mAU]	Area %
1	3.452	FM	0.0699	5087.3	1212.6	18.842
2	3.742	MM	0.0682	4285.4	1047.3	15.873
3	4.484	FM	0.1045	17626.3	2810.4	65.285
Total				26999	5070.3	100%

(ee 60.88%)

Supplementary Figure 21. HPLC Chromatography of isolated **(R)-3aoa** after 35 min (Chiralpak AD-H 250*4.6 mm/5 um column, 5% isopropanol in hxane, 1.0 mL/min, 210 nm)

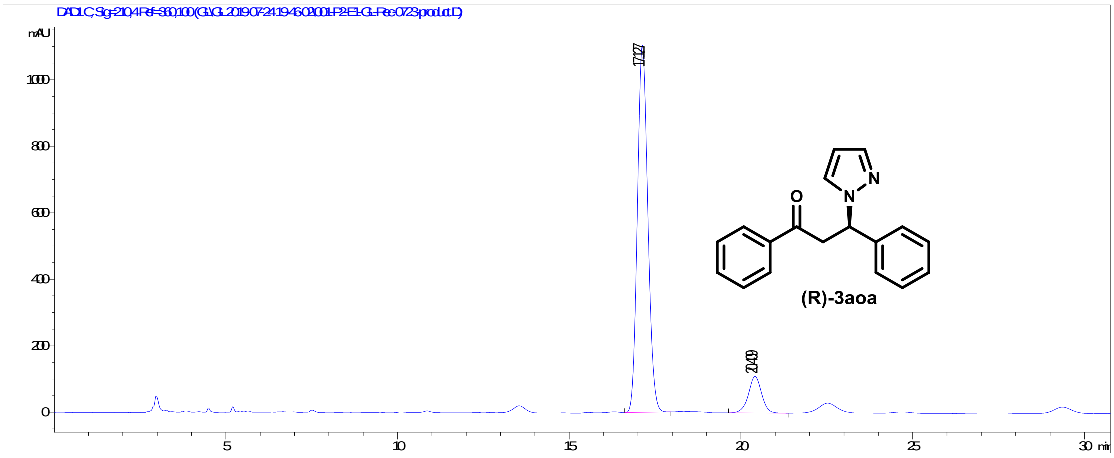

Peak #	RetTime [min]	Type	Width [min]	Area [mAU*s]	Height [mAU]	Area %
1	17.127	BB	0.3288	23361.7	1104.5	88.943
2	20.409	BB	0.4039	2904.1	110	11.057
Total				26265.8	1214.5	100%

(ee 77.886%)

Supplementary Figure 22. HPLC Chromatography of isolated **(R)-3aoa** after 24 h (Chiralpak AD-H 250*4.6 mm/5 um column, 5% isopropanol in hxane, 1.0 mL/min, 210 nm)

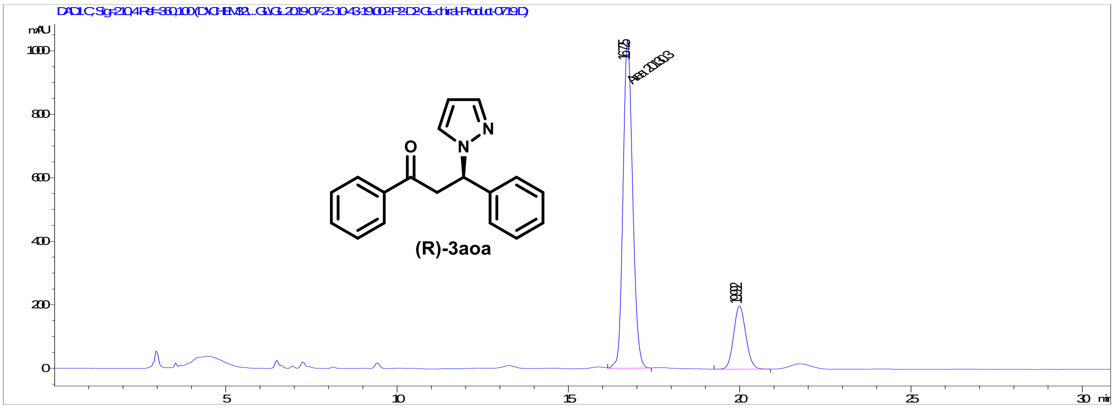

Peak #	RetTime [min]	Type	Width [min]	Area [mAU*s]	Height [mAU]	Area %
1	16.725	FM	0.3251	20130.3	1031.9	80.392
2	19.992	BB	0.3834	4909.9	199.3	19.608
Total				25040.2	1231.2	100%

(ee 60.78%)

Regioselectivity Determination for Oxo-amination of Unsymmetrical Diaryl Cyclopropanes

The regioselectivities for reactions of unsymmetric diaryl cyclopropanes were determined by HPLC.

Supplementary Figure 23. HPLC Chromatography of 3-(4-methoxyphenyl)-1-phenyl-3-(1H-pyrazol-1-yl)propan-1-one (**3apa**)

(Chiralpak OD-H 250*4.6 mm/5 um column, 15% isopropanol in hxane, 1.0 mL/min, 210 nm)

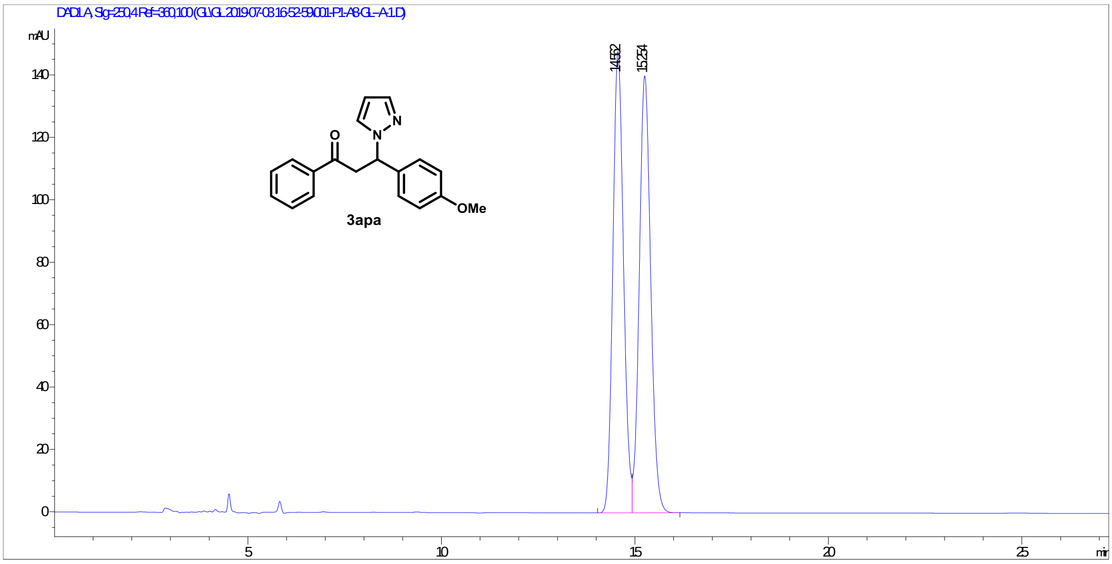

Peak #	RetTime [min]	Type	Width [min]	Area [mAU*s]	Height [mAU]	Area %
1	14.562	BV	0.2978	2834.9	147.8	49.652
2	15.254	VB	0.3175	2874.7	140	50.348
Total				5709.6	287.8	100%

Supplementary Figure 24. HPLC Chromatography of 1-(4-methoxyphenyl)-3-phenyl-3-(1H-pyrazol-1-yl)propan-1-one (**3apa'**)

(Chiralpak OD-H 250*4.6 mm/5 um column, 15% isopropanol in hxane, 1.0 mL/min, 210 nm)

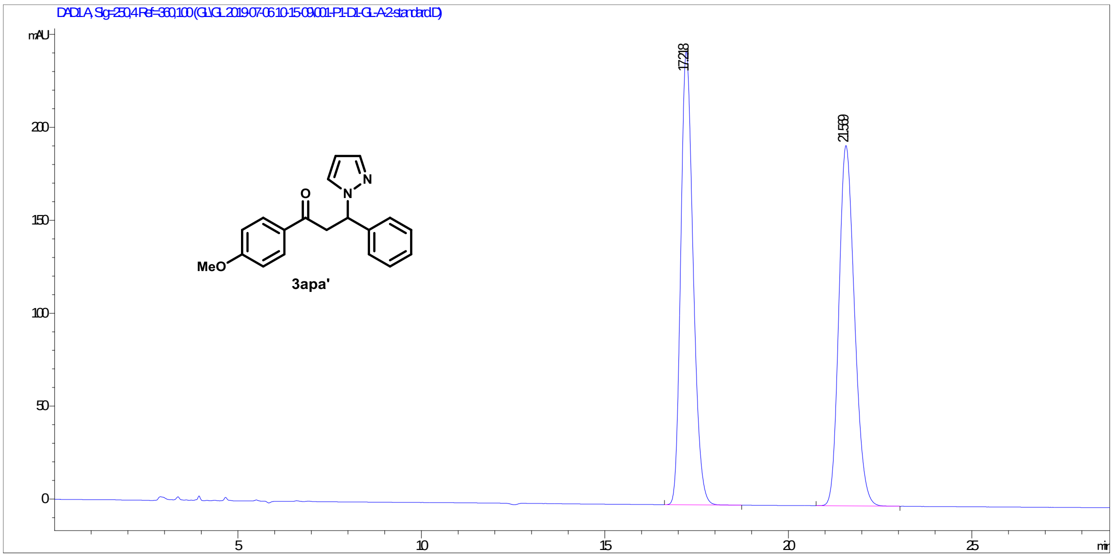

Peak #	RetTime [min]	Type	Width [min]	Area [mAU*s]	Height [mAU]	Area %
1	17.218	BB	0.3619	5728.5	244	49.928
2	21.569	BB	0.4597	5744.9	193.9	50.072
Total				11473.4	437.9	100%

Supplementary Figure 25. HPLC Chromatography of reaction solution of **1ap**

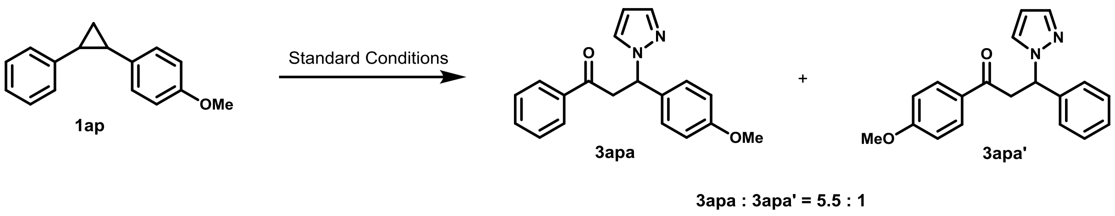

(Chiralpak OD-H 250*4.6 mm/5 um column, 15% isopropanol in hxane, 1.0 mL/min, 210 nm)

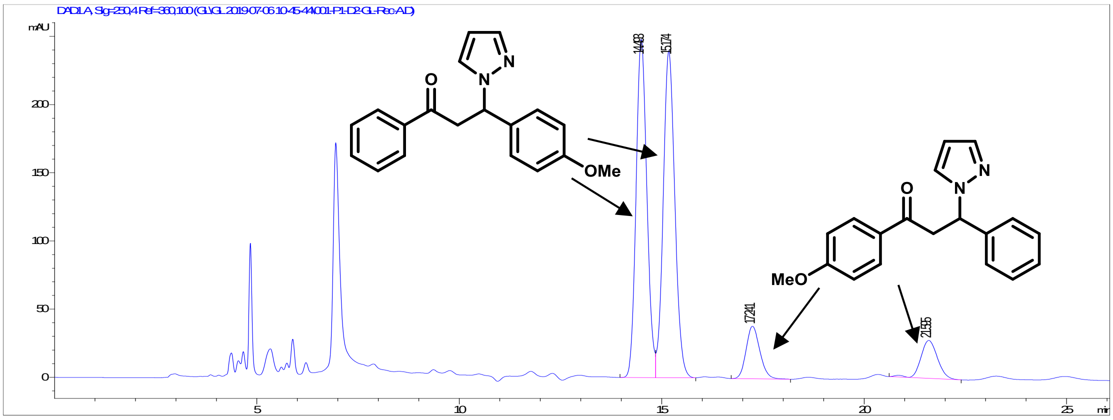

Peak	RetTime	Type	Width	Area	Height	Area
#	[min]		[min]	[mAU*s]	[mAU]	%
1	14.493	VB	0.2892	4617.8	248.1	41.536
2	15.174	BV	0.3091	4789.7	239.8	43.083
3	17.241	BB	0.3651	908.4	38.5	8.171
4	21.595	VB R	0.4372	801.5	27.9	7.210
Total				11117.4	554.3	100%

Supplementary Figure 26. HPLC Chromatography of 1-(4-methoxyphenyl)-3-(naphthalen-2-yl)-3-(1H-pyrazol-1-yl)propan-1-one (**3ara'**)

(Chiralpak OD-H 250*4.6 mm/5 um column, 15% isopropanol in hxane, 1.0 mL/min, 210 nm)

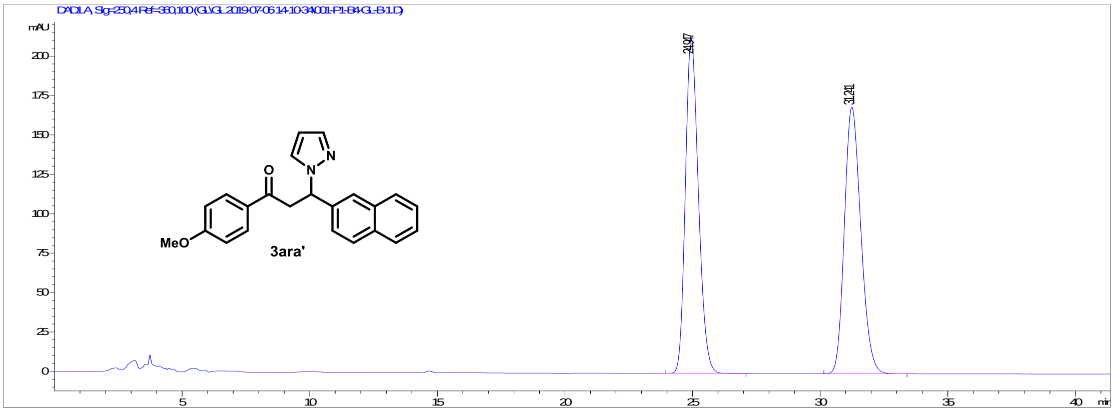

Peak	RetTime	Type	Width	Area	Height	Area
#	[min]		[min]	[mAU*s]	[mAU]	%
1	24.947	BB	0.5517	7564.1	212.3	49.951
2	31.241	BB	0.6987	7578.9	169.2	50.049
Total				15143	381.5	100%

Supplementary Figure 27. HPLC Chromatography of 3-(4-methoxyphenyl)-1-(naphthalen-2-yl)-3-(1H-pyrazol-1-yl)propan-1-one (**3ara**)

(Chiralpak OD-H 250*4.6 mm/5 um column, 15% isopropanol in hxane, 1.0 mL/min, 210 nm)

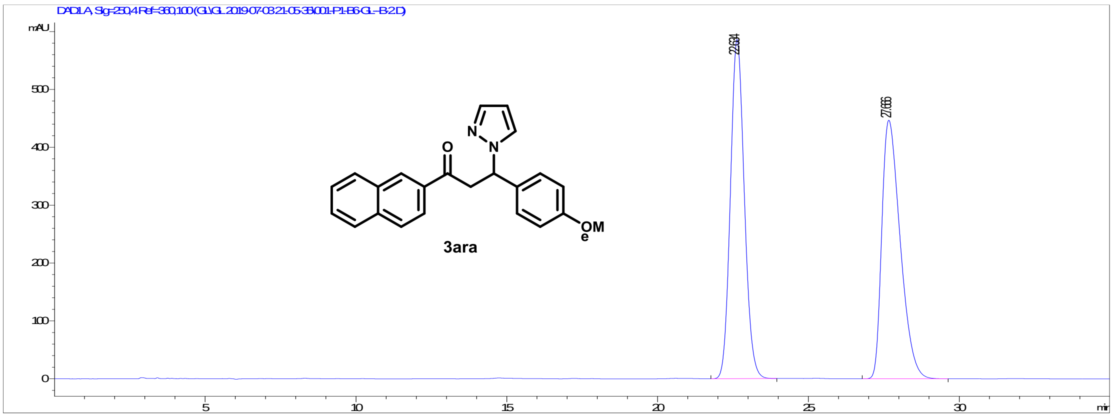

Peak #	RetTime [min]	Type	Width [min]	Area [mAU*s]	Height [mAU]	Area %
1	22.634	BB	0.4991	18840.5	586	49.889
2	27.666	BB	0.6561	18924.2	446.8	50.111
Total				37764.7	1032.8	100%

Supplementary Figure 28. HPLC Chromatography of reaction solution of **1ar**

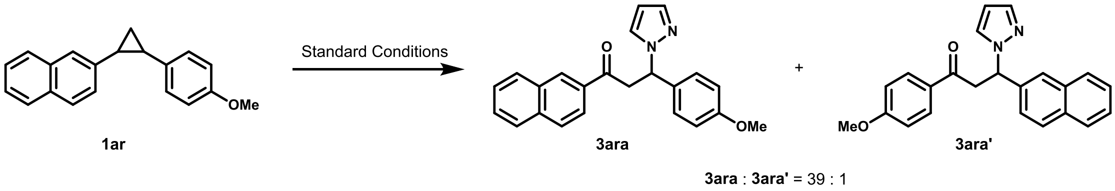

(Chiralpak OD-H 250*4.6 mm/5 um column, 15% isopropanol in hxane, 1.0 mL/min, 210 nm)

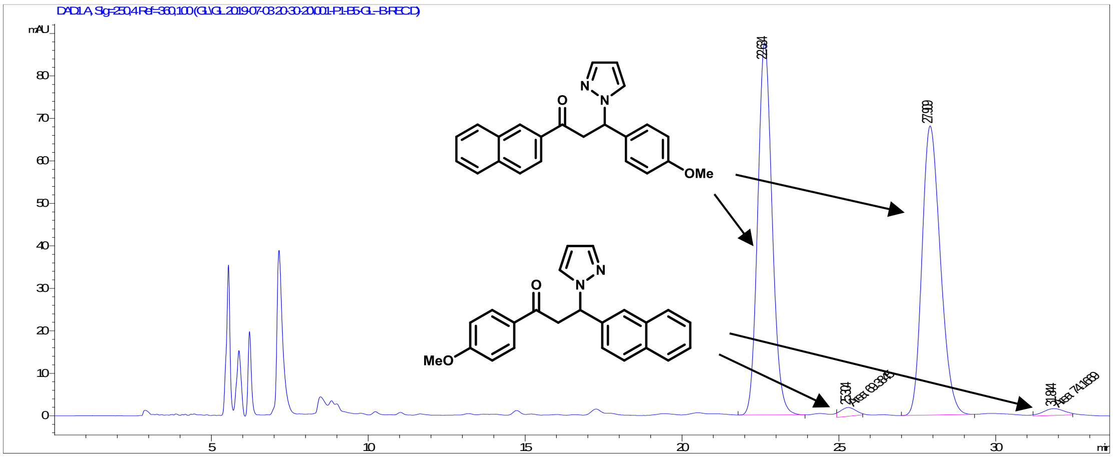

Peak #	RetTime [min]	Type	Width [min]	Area [mAU*s]	Height [mAU]	Area %
1	22.634	BB	0.2892	2804.4	87.7	49.014
2	25.304	MM	0.3091	69.4	2	1.213
3	27.909	BB	0.3651	2773.7	68.1	48.478
4	31.844	MM	0.4372	74.2	1.6	1.296
Total				5721.7	159.4	100%

Supplementary Figure 29. HPLC Chromatography of 3-(4-methoxyphenyl)-3-(1H-pyrazol-1-yl)-1-(4-(trifluoromethyl)phenyl)propan-1-one (**3aqa**)

(Chiralpak OD-H 250*4.6 mm/5 um column, 15% isopropanol in hxane, 1.0 mL/min, 210 nm)

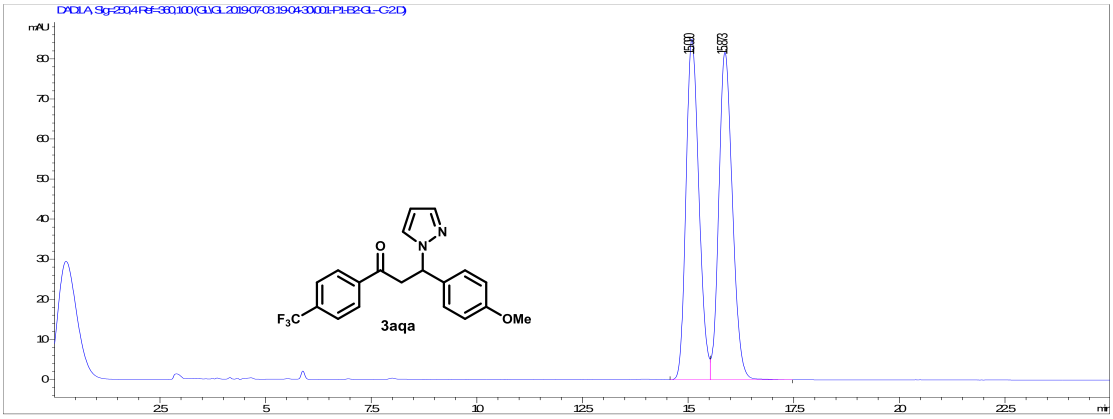

Peak #	RetTime [min]	Type	Width [min]	Area [mAU*s]	Height [mAU]	Area %
1	15.09	BV	0.3308	1814.2	85.1	49.629
2	15.873	VB	0.3487	1841.4	81.8	50.371
Total				3655.6	166.9	100%

Supplementary Figure 30. HPLC Chromatography of 1-(4-methoxyphenyl)-3-(1H-pyrazol-1-yl)-3-(4-(trifluoromethyl)phenyl)propan-1-one (**3aqa'**)

(Chiralpak OD-H 250*4.6 mm/5 um column, 15% isopropanol in hxane, 1.0 mL/min, 210 nm)

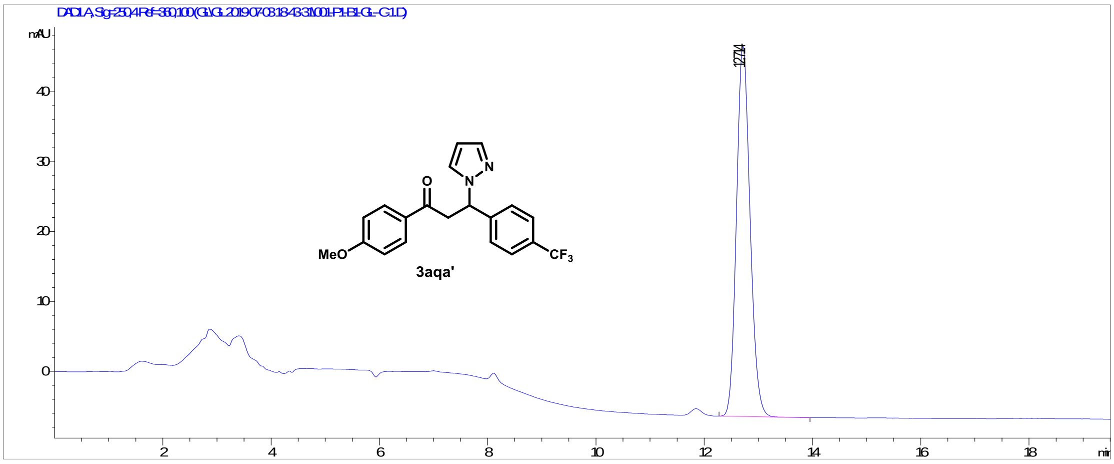

Peak #	RetTime [min]	Type	Width [min]	Area [mAU*s]	Height [mAU]	Area %
1	12.714	BB	0.2692	923.3	53	100.00
Total				923.3	53	100%

Supplementary Figure 31. HPLC Chromatography of reaction solution of **1aq**

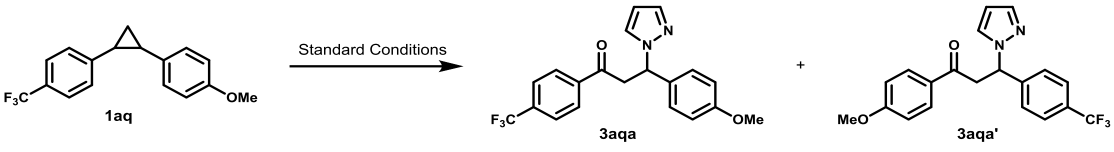

(Chiralpak OD-H 250*4.6 mm/5 um column, 15% isopropanol in hxane, 1.0 mL/min, 210 nm)

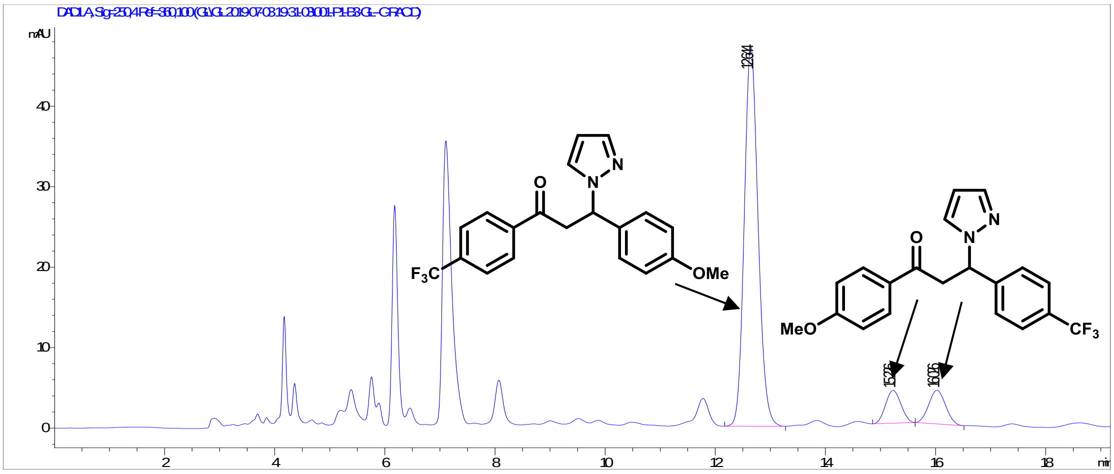

Peak #	RetTime [min]	Type	Width [min]	Area [mAU*s]	Height [mAU]	Area %
1	12.644	BB	0.2698	824.4	47.2	83.085
2	15.226	BB	0.305	79.1	4.1	7.976
3	16.026	BB	0.3339	88.7	4.2	8.939
Total				992.2	55.5	100%

Characterization of Structurally Novel Compounds

1-Bromo-4-(2,2-dimethylcyclopropyl)benzene (1b)

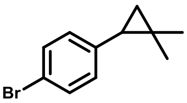

1b

^{1}H NMR (400 MHz, Chloroform-*d*): δ 7.38 (d, *J* = 8.4 Hz, 2H), 7.03 (d, *J* = 8.1 Hz, 2H), 1.82 (dd, *J* = 8.4, 6.2 Hz, 1H), 1.22 (s, 3H), 0.79 (s, 3H), 0.83 – 0.75 (m, 2H) ppm; **^{13}C NMR (100 MHz, Chloroform-*d*):** δ 139.54, 130.98, 130.75, 119.29, 29.27, 27.41, 20.42, 19.26, 18.64 ppm; **HRMS (ESI, m/z):** calculated for [M+H]$^+$: 225.0279, found: 225.0254.

1-(*tert*-butyl)-4-(2,2-dimethylcyclopropyl)benzene (1d)

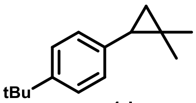

1d

^{1}H NMR (400 MHz, Chloroform-*d*): δ 7.30 (d, *J* = 8.3 Hz, 2H), 7.11 (d, *J* = 8.3 Hz, 2H), 1.88 – 1.83 (m, 1H), 1.34 (d, *J* = 1.8 Hz, 9H), 1.24 (s, 3H), 0.84 (s, 3H), 0.81 – 0.73 (m, 2H) ppm; **^{13}C NMR (100 MHz, Chloroform-*d*):** δ 148.29, 137.35, 128.65, 124.83, 34.44, 31.55, 29.44, 27.62, 20.52, 18.98, 18.56 ppm; **HRMS (ESI, m/z):** calculated for [M+H]$^+$: 203.1800, found: 203.1793.

4-(2,2-dimethylcyclopropyl)-1,1'-biphenyl (1h)

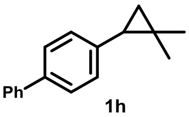

1h

^{1}H NMR (400 MHz, Chloroform-*d*): δ 7.57 (d, *J* = 7.6 Hz, 2H), 7.49 (d, *J* = 7.8 Hz, 2H), 7.39 (t, *J* = 7.6 Hz, 2H), 7.29 (t, *J* = 7.4 Hz, 1H), 7.21 (d, *J* = 7.8 Hz, 2H), 1.89 (t, *J* = 7.1 Hz, 1H), 1.23 (s, 3H), 0.83 (s, 3H), 0.82 – 0.78 (m, 1H) ppm; **^{13}C NMR (100 MHz, Chloroform-*d*):** δ 141.19, 139.64, 138.39, 129.38, 128.79, 127.03, 126.66, 29.61, 27.59, 20.49, 19.36, 18.67 ppm; **HRMS (ESI, m/z):** calculated for [M+H]+: 223.1487, found: 223.1479.

1-cyclopropyl-4-(2,2-dimethylcyclopropyl)benzene (1i)

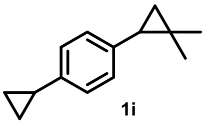

1i

^{1}H NMR (400 MHz, Chloroform-*d*): δ 7.06 (d, *J* = 8.1 Hz, 2H), 6.98 (d, *J* = 8.2 Hz, 2H), 1.91 – 1.85 (m, 1H), 1.84 (dd, *J* = 7.4, 5.8 Hz, 1H), 1.22 (s, 3H), 0.97 – 0.91 (m, 2H), 0.80 (s, 3H), 0.76 (d, *J* = 1.3 Hz, 1H), 0.75 (d, *J* = 3.6 Hz, 1H), 0.70 – 0.65 (m, 2H) ppm; **^{13}C NMR (100 MHz, Chloroform-*d*):** δ 141.06, 137.45, 128.94, 125.25, 29.46, 27.54, 20.50, 18.92, 18.46, 15.14, 9.17, 9.14 ppm; **HRMS (ESI, m/z):** calculated for [M+H]$^+$: 187.487, found: 187.1484.

4-(2,2-dimethylcyclopropyl)-2-fluoro-1-methoxybenzene (1j)

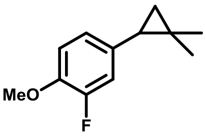

1j

^{1}H NMR (400 MHz, Chloroform-*d*): δ 6.92 – 6.84 (m, 1H), 3.86 (s, 1H), 1.80 (dd, *J* = 8.4, 5.9 Hz, 1H), 1.20 (s, 1H), 0.79 (s, 1H), 0.75 (dd, *J* = 8.5, 4.8 Hz, 1H), 0.70 (t, *J* = 5.3 Hz, 1H) ppm; **^{13}C NMR (100 MHz, Chloroform-*d*):** δ 151.97 (d, *J* = 244.5 Hz), 145.46 (d, *J* = 10.8 Hz), 133.65 (d, *J* = 6.3 Hz), 124.40 (d, *J* = 3.3 Hz), 116.64 (d, *J* = 17.8 Hz), 112.93 (d, *J* = 2.3 Hz), 56.27, 28.81 (d, *J* = 1.6 Hz), 27.22, 20.38, 18.89, 18.55 ppm; **HRMS (ESI, m/z):** calculated for [M+H]$^+$: 195.185, found: 195.1179.

2-chloro-4-(2,2-dimethylcyclopropyl)-1-methoxybenzene (1k)

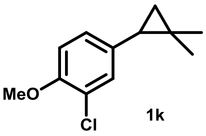

1k

^{1}H NMR (400 MHz, Chloroform-*d*): δ 7.13 (d, *J* = 2.1 Hz, 1H), 6.95 (dd, *J* = 8.4, 2.1 Hz, 1H), 6.77 (d, *J* = 8.4 Hz, 1H), 3.81 (s, 3H), 1.74 (dd, *J* = 8.4, 5.9 Hz, 1H), 1.15 (s, 3H), 0.74 (s, 3H), 0.70 (dd, *J* = 8.5, 4.8 Hz, 1H), 0.66 (t, *J* = 5.3 Hz, 1H) ppm; **^{13}C NMR (100 MHz, Chloroform-*d*):** δ 153.07, 133.79, 130.83, 128.18, 121.81, 111.73, 56.20, 28.74,

27.31, 20.56, 18.91, 18.61 ppm; **HRMS (ESI, m/z):** calculated for $[M+H]^+$: 211.0890, found: 211.0884.

2-bromo-4-(2,2-dimethylcyclopropyl)-1-methoxybenzene (1l)

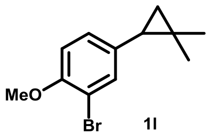

1l

^{1}H NMR (400 MHz, Chloroform-*d*): δ 7.35 (d, *J* = 0.7 Hz, 1H), 7.05 (dd, *J* = 8.4, 0.8 Hz, 1H), 6.80 (d, *J* = 8.4 Hz, 1H), 3.87 (s, 3H), 1.84 – 1.74 (m, 1H), 1.20 (s, 3H), 0.78 (s, 3H), 0.75 (dd, *J* = 8.4, 4.8 Hz, 1H), 0.70 (t, *J* = 5.2 Hz, 1H) ppm; **^{13}C NMR (100 MHz, Chloroform-*d*):** δ 153.82, 134.18, 133.78, 128.83, 111.40, 110.98, 56.24, 28.53, 27.21, 20.49, 18.87, 18.52 ppm; **HRMS (ESI, m/z):** calculated for $[M+H]^+$: 255.0385, found: 255.0379.

4-(2,2-dimethylcyclopropyl)-1-methoxy-2-methylbenzene (1m)

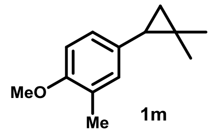

1m

^{1}H NMR (400 MHz, Chloroform-*d*): δ 6.97 (dd, *J* = 2.0, 1.2 Hz, 1H), 6.96 – 6.93 (m, 1H), 6.74 (d, *J* = 8.1 Hz, 1H), 3.82 (s, 3H), 2.22 (s, 3H), 1.80 (t, *J* = 7.1 Hz, 1H), 1.22 (s, 3H), 0.81 (s, 3H), 0.75 – 0.70 (m, 2H) ppm; **^{13}C NMR (100 MHz, Chloroform-*d*):** δ 155.77, 131.92, 131.54, 126.83, 125.80, 109.41, 55.32, 28.93, 27.38, 20.49, 18.48, 18.31, 16.31 ppm; **HRMS (ESI, m/z):** calculated for $[M+H]^+$: 191.1436, found: 191.1435.

Methyl 5-(2,2-dimethylcyclopropyl)-2-methoxybenzoate (1o)

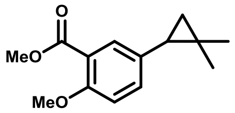

1o

^{1}H NMR (400 MHz, Chloroform-*d*): δ 7.58 (d, *J* = 2.3 Hz, 1H), 7.26 – 7.23 (m, 1H), 6.88 (d, *J* = 8.5 Hz, 1H), 3.88 (d, *J* = 3.4 Hz, 6H), 1.81 (dd, *J* = 8.8, 6.6 Hz, 1H), 1.20 (s, 3H), 0.76 (s, 3H), 0.75 – 0.73 (m, 1H) ppm; **^{13}C NMR (100 MHz, Chloroform-*d*):** δ 167.06, 157.30, 134.00, 132.26, 132.14, 119.48, 111.79, 56.19, 52.09, 28.68, 27.30, 20.56, 18.82, 18.54 ppm; **HRMS (ESI, m/z):** calculated for $[M+H]^+$: 235.1334, found: 235.1327.

4-(2,2-dimethylcyclopropyl)-1,2-dimethylbenzene (1p)

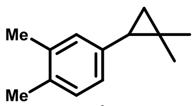

1p

^{1}H NMR (400 MHz, Chloroform-*d*): δ 6.94 (d, *J* = 8.7 Hz, 2H), 6.73 (d, *J* = 8.1 Hz, 1H), 3.81 (s, 3H), 2.21 (s, 3H), 1.87 – 1.68 (m, 1H), 1.21 (s, 3H), 0.80 (s, 3H), 0.72 (d, *J* = 1.3 Hz, 1H), 0.70 (s, 1H) ppm; **^{13}C NMR (100 MHz, Chloroform-*d*):** δ 137.67, 135.86, 133.53, 130.41, 129.14, 126.16, 29.38, 20.42, 19.84, 19.35, 18.73, 18.27 ppm; **HRMS (ESI, m/z):** calculated for $[M+H]^+$: 175.1487, found: 175.1482.

2-(2,2-dimethylcyclopropyl)naphthalene (1q)

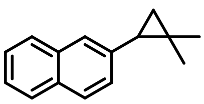
1q

^{1}H NMR (400 MHz, Chloroform-*d*): δ 7.85 – 7.79 (m, 2H), 7.78 (d, *J* = 8.5 Hz, 1H), 7.59 (s, 1H), 7.50 – 7.41 (m, 2H), 7.39 (dd, *J* = 8.4, 1.7 Hz, 1H), 2.07 (dd, *J* = 8.3, 5.9 Hz, 1H), 1.31 (s, 3H), 1.02 – 0.96 (m, 1H), 0.89 (dd, *J* = 8.4, 4.7 Hz, 1H), 0.85 (s, 3H) ppm; **^{13}C NMR (100 MHz, Chloroform-*d*):** δ 138.14, 133.46, 131.95, 128.41, 127.59, 127.47, 127.26, 126.48, 125.82, 125.00, 30.06, 27.53, 20.42, 19.40, 18.51 ppm; **HRMS (ESI, m/z):** calculated for $[M+Na]^+$: 219.1150, found: 219.1139.

5-(2,2-dimethylcyclopropyl)benzofuran (1r)

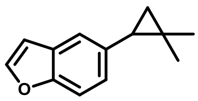

1r

^{1}H NMR (400 MHz, Chloroform-*d*): δ 7.58 (d, *J* = 2.2 Hz, 1H), 7.40 (d, *J* = 8.5 Hz, 1H), 7.37 – 7.36 (m, 1H), 7.13 (dd, *J* = 8.5, 1.8 Hz, 1H), 6.72 – 6.69 (m, 1H), 1.97 (dd, *J* = 8.3, 6.0 Hz, 1H), 1.25 (s, 3H), 0.82 (d, *J* = 2.1 Hz, 1H), 0.79 (s, 3H) ppm; **^{13}C NMR (100 MHz, Chloroform-*d*):** δ 153.60, 145.05, 134.90, 127.28, 126.02, 120.95, 110.62, 106.52, 29.68, 27.47, 20.64, 18.71, 18.66 ppm; **HRMS (ESI, m/z):** calculated for $[M+H]^+$: 187.1123, found: 187.1118.

5-(2,2-dimethylcyclopropyl)-1a,6b-dihydro-1H-cyclopropa[b]benzofuran (1s)

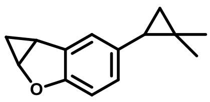

1s

^{1}H NMR (400 MHz, Chloroform-*d*): δ 7.13 (dd, *J* = 8.3, 1.8 Hz, 1H), 6.87 (dddd, *J* = 10.1, 8.2, 1.9, 0.7 Hz, 1H), 6.71 (dd, *J* = 8.2, 2.2 Hz, 1H), 4.77 (tt, *J* = 5.4, 2.0 Hz, 1H), 2.57 (dddd, *J* = 9.1, 5.6, 4.0, 1.8 Hz, 1H), 1.81 (ddd, *J* = 8.2, 6.0, 1.9 Hz, 1H), 1.20 (s, 3H), 0.96 (dddd, *J* = 8.8, 6.4, 5.5, 1.0 Hz, 1H), 0.79 (s, 3H), 0.73 (td, *J* = 4.2, 3.6, 1.6 Hz, 1H), 0.70 (dd, *J* = 5.8, 2.3 Hz, 1H), 0.31 (ddt, *J* = 6.3, 4.1, 2.1 Hz, 1H) ppm; **^{13}C NMR (100 MHz, Chloroform-*d*):** δ 157.52 (d, *J* = 9.4 Hz), 132.54 (d, *J* = 2.0 Hz), 130.90 (d, *J* = 3.4 Hz), 127.60 (d, *J* = 31.8 Hz), 124.54 (d, *J* = 29.0 Hz), 109.55 (d, *J* = 5.2 Hz), 61.73 (d, *J* = 11.2 Hz), 29.33, 27.43, 20.62 (d, *J* = 1.1 Hz), 19.86 (d, *J* = 5.0 Hz), 18.59 (d, *J* = 1.0 Hz), 10.35 ppm; **HRMS (ESI, m/z):** calculated for $[M+H]^+$: 201.1279, found: 201.1270.

2-(2,2-dimethylcyclopropyl)dibenzo[b,e]oxepin-11(6H)-one (1t)

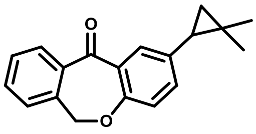

1t

^{1}H NMR (400 MHz, Chloroform-*d*): δ 8.00 (d, *J* = 2.3 Hz, 1H), 7.90 (dd, *J* = 7.7, 1.3 Hz, 1H), 7.55 (td, *J* = 7.4, 1.5 Hz, 1H), 7.47 (td, *J* = 7.6, 1.3 Hz, 1H), 7.36 (dd, *J* = 7.5, 1.3 Hz, 1H), 7.29 (dd, *J* = 8.4, 2.4 Hz, 1H), 6.96 (d, *J* = 8.4 Hz, 1H), 5.17 (s, 2H), 1.92 – 1.80 (m, 1H), 1.23 (s, 3H), 0.86 – 0.75 (m, 2H), 0.80 (s, 4H) ppm; **^{13}C NMR (100 MHz, Chloroform-*d*):** δ 191.39, 159.45, 140.65, 136.34, 135.67, 134.21, 132.63, 131.50, 129.46, 129.18, 127.73, 124.74, 120.11, 73.58, 28.77, 27.25, 20.44, 18.94, 18.54 ppm; **HRMS (ESI, m/z):** calculated for $[M+H]^+$: 279.1385, found: 279.1379.

1-(2,2-diethylcyclopropyl)-4-methoxybenzene (1v)

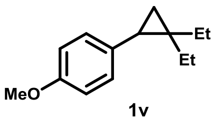

1v

^{1}H NMR (400 MHz, Chloroform-*d*): δ 7.10 (d, *J* = 8.6 Hz, 2H), 6.82 (d, *J* = 8.6 Hz, 2H), 3.79 (s, 3H), 1.85 (dd, *J* = 8.3, 6.1 Hz, 1H), 1.61 (dd, *J* = 14.0, 7.1 Hz, 1H), 1.24 – 1.13 (m, 2H), 0.99 (t, *J* = 7.4 Hz, 3H), 0.82 – 0.74 (m, 4H), 0.73 – 0.66 (m, 2H) ppm; **^{13}C NMR (100 MHz, Chloroform-*d*):** δ 157.52, 132.12, 130.00, 113.22, 55.22, 29.42, 28.68, 28.54, 22.82, 16.65, 10.85, 10.52 ppm; **HRMS (ESI, m/z):** calculated for $[M+H]^+$: 205.1592, found: 205.1590.

2'-(4-methoxyphenyl)-1,1':1',1''-tercyclopropane (1w)

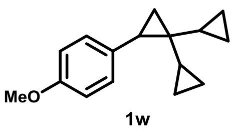

1w

^{1}H NMR (400 MHz, Chloroform-*d*): δ 7.10 (d, *J* = 8.5 Hz, 2H), 6.80 (d, *J* = 8.7 Hz, 2H), 3.79 (s, 3H), 1.78 (dd, *J* = 8.9, 6.0 Hz, 1H), 1.08 (tt, *J* = 8.3, 5.3 Hz, 1H), 0.88 (t, *J* = 6.7 Hz, 1H), 0.56 (t, *J* = 5.5 Hz, 1H), 0.54 – 0.46 (m, 2H), 0.39 – 0.34 (m, 2H), 0.30 (ddd, *J* = 14.6, 5.8, 2.9 Hz, 2H), 0.11 – 0.07 (m, 2H), 0.06 – 0.02 (m, 1H) ppm; **^{13}C**

NMR (100 MHz, Chloroform-*d*): δ 157.56, 132.03, 130.31, 113.25, 55.30, 29.81, 27.25, 25.70, 15.97, 14.43, 12.04, 3.32, 2.18 (d, *J* = 4.0 Hz), 1.64 ppm; **HRMS (ESI, m/z):** calculated for $[M+H]^+$: 229.1592, found: 229.1584.

1-(4-methoxyphenyl)spiro[2.4]heptane (1z)

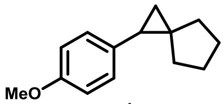

1z

^{1}H NMR (400 MHz, Chloroform-*d*): δ 7.02 (d, *J* = 8.6 Hz, 2H), 6.82 (d, *J* = 8.7 Hz, 2H), 3.79 (s, 3H), 1.93 (dd, *J* = 8.7, 6.0 Hz, 1H), 1.65 (dtt, *J* = 12.0, 8.3, 3.7 Hz, 4H), 1.61 – 1.53 (m, 2H), 1.38 – 1.28 (m, 1H), 1.27 – 1.18 (m, 1H), 1.00 (dd, *J* = 8.7, 4.8 Hz, 1H), 0.95 – 0.85 (m, 1H) ppm; **^{13}C NMR (100 MHz, Chloroform-*d*):** δ 157.54, 133.00, 129.07, 113.49, 55.34, 38.04, 30.67, 30.11, 29.11, 26.52, 26.48, 18.22 ppm; **HRMS (ESI, m/z):** calculated for $[M+H]^+$: 203.1436, found: 203.1432.

1-(4-methoxyphenyl)spiro[2.5]octane (1aa)

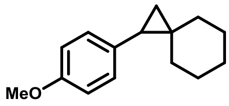

1aa

^{1}H NMR (400 MHz, Chloroform-*d*): δ 7.12 (d, *J* = 8.8 Hz, 2H), 6.82 (d, *J* = 8.7 Hz, 2H), 3.80 (s, 3H), 1.82 (dd, *J* = 8.2, 5.9 Hz, 1H), 1.59 (q, *J* = 7.1, 6.6 Hz, 2H), 1.45 (dq, *J* = 13.8, 5.8 Hz, 4H), 1.30 (dd, *J* = 10.2, 5.4 Hz, 2H), 1.12 – 1.01 (m, 2H), 0.76 (t, *J* = 5.2 Hz, 1H), 0.69 (dd, *J* = 8.4, 4.7 Hz, 1H) ppm; **^{13}C NMR (100 MHz, Chloroform-*d*):** δ 157.60, 131.98, 129.89, 113.28, 55.31, 38.02, 30.58, 28.71, 26.41, 26.29, 25.96, 25.13, 16.75 ppm; **HRMS (ESI, m/z):** calculated for $[M+H]^+$: 217.1592, found: 217.1586.

2-bromo-1-methoxy-4-(2-methylcyclopropyl)benzene (1ac)

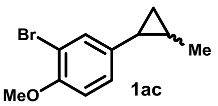

(***Trans*** : ***Cis*** = 1.0 : 0.4)

Trans **isomer ^{1}H NMR (400 MHz, Chloroform-*d*):** δ 7.21 (d, *J* = 2.2 Hz, 1H), 6.95 (dd, *J* = 8.4, 2.2 Hz, 1H), 6.78 (d, *J* = 8.5 Hz, 1H), 3.85 (s, 3H), 1.50 (dt, *J* = 9.0, 4.7 Hz, 1H), 1.16 (d, *J* = 5.9 Hz, 3H), 0.98 – 0.92 (m, 1H), 0.83 – 0.79 (m, 1H), 0.69 (dt, *J* = 8.5, 5.2 Hz, 1H) ppm; **^{13}C NMR (100 MHz, Chloroform-*d*):** δ 153.63, 134.08, 130.49, 125.68, 111.87, 111.42, 56.35, 23.17, 19.01, 17.54, 17.10 ppm.

Cis **isomer ^{1}H NMR (400 MHz, Chloroform-*d*):** δ 7.38 (d, *J* = 2.1 Hz, 1H), 7.10 – 7.07 (m, 1H), 6.81 (d, *J* = 8.4 Hz, 1H), 3.87 (s, 3H), 1.99 (td, *J* = 8.6, 5.8 Hz, 1H), 1.09 (dtd, *J* = 14.7, 6.0, 2.6 Hz, 1H), 1.00 – 0.98 (m, 1H), 0.78 (d, *J* = 6.2 Hz, 3H), 0.48 (q, *J* = 5.5 Hz, 1H) ppm; **^{13}C NMR (100 MHz, Chloroform-*d*):** δ 153.91, 137.76, 133.33, 129.21, 111.46, 110.99, 56.25, 19.96, 13.77, 12.46, 11.05 ppm; **HRMS (ESI, m/z):** calculated for $[M+H]^+$: 241.0228, found: 241.0220.

4-(2-(3-bromo-4-methoxyphenyl)cyclopropyl)butyl acetate (1ad)

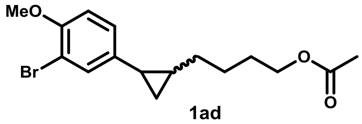

1ad

(***Trans*** : ***Cis*** = 1.0 : 0.1)

Only spectra of the major isomer is provided.

Trans **isomer ^{1}H NMR (400 MHz, Chloroform-*d*):** δ 7.21 (d, *J* = 2.2 Hz, 1H), 6.96 (dd, *J* = 8.4, 2.2 Hz, 1H), 6.78 (d, *J* = 8.4 Hz, 1H), 4.06 (t, *J* = 6.7 Hz, 2H), 3.85 (s, 3H), 2.05 (s, 3H), 1.66 (dt, *J* = 14.5, 6.8 Hz, 2H), 1.54 (dd, *J* = 8.7, 4.5 Hz, 1H), 1.54 – 1.45 (m, 2H), 1.44 – 1.33 (m, 2H), 0.98 – 0.89 (m, 1H), 0.81 (dt, *J* = 8.4, 4.9 Hz, 1H), 0.72 (dt, *J* = 8.6, 5.2 Hz, 1H) ppm; **^{13}C NMR (100 MHz, Chloroform-*d*):** δ 171.29, 153.68, 137.52, 130.51, 125.80, 111.85, 111.47, 64.54, 56.35, 33.87, 28.38, 25.76, 23.17, 22.07, 21.07, 15.72 ppm; **HRMS (ESI, m/z):** calculated for $[M+Na]^+$: 363.0572, found 363.0557.

4-(2-(3-bromo-4-methoxyphenyl)cyclopropyl)butyl dimethylcarbamate (1ae)

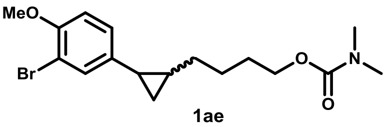

1ae

(*Trans* : *Cis* = 1.0 : 0.1)

Only spectra of the major isomer is provided. ***Trans* isomer ^{1}H NMR (400 MHz, Chloroform-*d*):** δ 7.20 (d, *J* = 2.2 Hz, 1H), 6.95 (dd, *J* = 8.5, 2.2 Hz, 1H), 6.78 (d, *J* = 8.4 Hz, 1H), 4.06 (t, *J* = 6.6 Hz, 2H), 3.85 (s, 3H), 2.91 (s, 3H), 2.89 (s, 3H), 1.70 – 1.61 (m, 2H), 1.54 (t, *J* = 4.5 Hz, 1H), 1.53 – 1.44 (m, 2H), 1.42 – 1.36 (m, 2H), 0.98 – 0.89 (m, 1H), 0.81 (dt, *J* = 8.4, 4.9 Hz, 1H), 0.75 – 0.68 (m, 1H) ppm; **^{13}C NMR (100 MHz, Chloroform-d):** δ 156.83, 153.65, 137.59, 130.50, 125.78, 111.85, 111.45, 65.33, 56.35, 33.90, 28.87, 25.78, 23.28, 22.09, 15.72 ppm; **HRMS (ESI, m/z):** calculated for [M+H]$^+$: 370.1018, found 370.1009.

(4-(2-(3-bromo-4-methoxyphenyl)cyclopropyl)butoxy)(tert-butyl)dimethylsilane (1af)

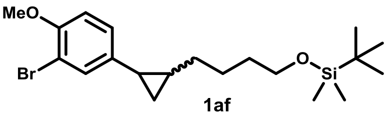

1af

(*Trans* : *Cis* = 1.0 : 0.1)

Only spectra of the major isomer is provided. ***Trans* isomer ^{1}H NMR (400 MHz, Chloroform-*d*):** δ 7.21 (d, *J* = 2.2 Hz, 1H), 6.96 (dd, *J* = 8.4, 2.2 Hz, 1H), 6.78 (d, *J* = 8.4 Hz, 1H), 3.85 (s, 3H), 3.61 (t, *J* = 6.4 Hz, 2H), 1.56 (d, *J* = 8.6 Hz, 1H), 1.55 – 1.50 (m, 2H), 1.47 (ddd, *J* = 13.8, 7.0, 4.3 Hz, 2H), 1.41 – 1.34 (m, 2H), 0.89 (s, 9H), 0.80 (dt, *J* = 8.5, 4.9 Hz, 1H), 0.71 (dt, *J* = 8.6, 5.2 Hz, 1H), 0.05 (s, 6H) ppm; **^{13}C NMR (100 MHz, Chloroform-d):** δ 152.58, 136.70, 129.50, 124.76, 110.81, 110.41, 62.19, 55.31, 33.05, 31.60, 24.96, 24.59, 22.38, 21.05, 14.66, -0.00, -6.28 ppm; **HRMS (ESI, m/z):** calculated for [M+H]$^+$: 413.1511, found 413.1506.

1-(2-(3-bromopropyl)cyclopropyl)-4-methoxybenzene (1ag)

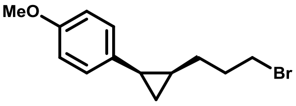

1ag

^{1}H NMR (400 MHz, Chloroform-*d*): δ 7.10 (d, *J* = 8.5 Hz, 2H), 6.82 (d, *J* = 8.7 Hz, 2H), 3.79 (s, 3H), 3.38 – 3.16 (m, 2H), 2.08 (q, *J* = 8.3 Hz, 1H), 1.90 – 1.78 (m, 2H), 1.31 – 1.20 (m, 1H), 1.10 – 1.00 (m, 2H), 0.97 – 0.89 (m, 1H), 0.61 (q, *J* = 5.1 Hz, 1H) ppm; **^{13}C NMR (100 MHz, Chloroform-*d*):** δ 153.07, 133.79, 130.83, 128.18, 121.81, 111.73, 56.20, 28.74, 27.31, 20.56, 18.91, 18.61 ppm; **HRMS (ESI, m/z):** calculated for [M+H]$^+$: 268.0463, found: 268.0456.

methyl 9-(2-(4-methoxyphenyl)cyclopropyl)nonanoate (1ah)

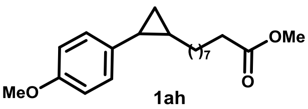

1ah

Only spectra of the major isomer is provided. ***Cis* isomer ^{1}H NMR (400 MHz, Chloroform-d):** δ 7.10 (d, *J* = 8.3 Hz, 2H), 6.81 (d, *J* = 8.7 Hz, 2H), 3.78 (s, 3H), 3.66 (s, 3H), 2.31 – 2.25 (m, 2H), 2.03 (td, *J* = 8.5, 5.9 Hz, 1H), 1.58 (p, *J* = 7.5 Hz, 2H), 1.31 – 1.12 (m, 11H), 1.04 – 0.96 (m, 1H), 0.93 – 0.90 (m, 1H), 0.87 – 0.81 (m, 1H), 0.54 (q, *J* = 5.6 Hz, 1H) ppm; **^{13}C NMR (100 MHz, Chloroform-*d*):** δ 174.43 , 157.66 , 131.74 , 130.05 , 113.31 , 55.28 , 51.52 , 34.19 , 29.42 , 29.23 , 29.20 , 28.66 , 25.03 , 20.18 , 18.80 , 9.72 ppm; **HRMS (ESI, m/z):** calculated for [M+H]+: 319.2268, found: 319.2263.

1-(2-benzylcyclopropyl)-4-methoxybenzene (1ai)

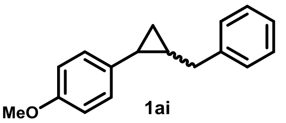

1ai

(*Trans* : *Cis* = 1.2 : 1.0)

***Cis* isomer ^{1}H NMR (400 MHz, Chloroform-*d*):** (resolved signals only) δ 7.33 – 7.15 (m, 5H), 6.99 – 6.95 (m, 2H), 6.81 – 6.76 (m, 2H), 3.76 (s, 3H), 2.77 (dd, *J* = 14.8, 6.7 Hz, 1H), 2.67 (dd, *J* = 14.8, 6.9 Hz, 1H), 1.75 (dt, *J* = 8.6, 4.9 Hz, 1H), 1.31 – 1.20 (m, 1H), 0.89 (ddt, *J* = 16.2, 8.5, 5.0 Hz, 2H) ppm; **^{13}C NMR (100 MHz, Chloroform-*d*):** δ 157.60, 141.50, 135.35, 130.15, 128.38, 126.85, 125.98, 113.80, 55.34, 40.02, 23.59, 22.61, 15.48 ppm.

***Trans* isomer ^{1}H NMR (400 MHz, Chloroform-*d*):** (resolved signals only) δ 7.33 – 7.15 (m, 5H), 7.09 (dd, *J* = 7.7, 1.2 Hz, 2H), 6.85 – 6.81 (m, 2H), 3.76 (s, 3H), 2.50 (dd, *J* = 15.0, 6.3 Hz, 1H), 2.23 – 2.10 (m, 2H), 1.35 (qt, *J* = 8.6, 6.1 Hz, 1H), 1.05 (td, *J* = 8.4, 5.1 Hz, 1H), 0.77 (q, *J* = 5.6 Hz, 1H) ppm; **^{13}C NMR (100 MHz, Chloroform-*d*):** δ 157.87, 142.20, 131.13, 128.37, 128.35, 128.17, 125.68, 113.43, 55.28, 34.61, 20.52, 19.60, 9.95 ppm; **HRMS (ESI, m/z):** calculated for $[M+H]^+$: 239.1436, found: 239.1435.

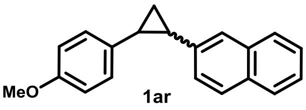

1ar

(*Trans* : *Cis* = 0.24 : 1.0)

1-methoxy-4-(2-phenethylcyclopropyl)benzene (1aj)

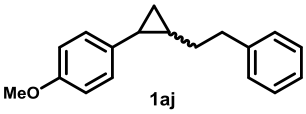

1aj

(*Trans* : *Cis* = 1.0 : 0.75)

***Cis* isomer ^{1}H NMR (400 MHz, Chloroform-*d*):** (resolved signals only) δ 7.30 – 7.13 (m, 5H), 7.07 – 7.00 (m, 2H), 6.83 (d, *J* = 8.7 Hz, 2H), 3.80 (s, 3H), 2.56 (ddd, *J* = 8.8, 6.8, 1.9 Hz, 2H), 2.10 (td, *J* = 8.6, 5.8 Hz, 1H), 1.41 (ddd, *J* = 13.9, 8.6, 7.0 Hz, 1H), 1.26 (ddd, *J* = 14.1, 6.9, 1.5 Hz, 1H), 1.14 – 1.03 (m, 1H), 0.95 (td, *J* = 8.4, 4.9 Hz, 1H), 0.61 (q, *J* = 5.5 Hz, 1H) ppm; **^{13}C NMR (100 MHz, Chloroform-*d*):** δ 157.73, 142.61, 131.31, 130.01, 128.44, 128.18, 125.57, 113.39, 55.29, 35.68, 30.96, 22.82, 20.24, 9.63 ppm.

***Trans* isomer ^{1}H NMR (400 MHz, Chloroform-*d*):** (resolved signals only) δ 7.30 – 7.13 (m, 5H), 6.96 (d, *J* = 8.7 Hz, 2H), 6.81 (d, *J* = 8.7 Hz, 2H), 3.78 (s, 3H), 2.77 (dd, *J* = 8.7, 6.6 Hz, 2H), 1.74 – 1.70 (m, 1H), 1.70 – 1.66 (m, 1H), 1.60 (dt, *J* = 9.1, 4.8 Hz, 1H), 1.02 (ddd, *J* = 12.0, 5.2, 2.4 Hz, 1H), 0.83 (dt, *J* = 8.4, 4.9 Hz, 1H), 0.73 (ddd, *J* = 8.6, 5.6, 4.7 Hz, 1H) ppm; **^{13}C NMR (100 MHz, Chloroform-*d*):** δ 157.50, 142.37, 135.72, 128.51, 128.31, 126.78, 125.71, 113.72, 55.34, 36.46, 35.85, 22.62, 18.34, 15.58 ppm; **HRMS (ESI, m/z):** calculated for [M+H]+: 253.1592, found: 253.1585.

(2-(4-(tert-butyl)phenyl)cyclopropyl)methyl acetate (1al)

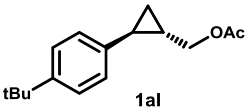

1al

^{1}H NMR (400 MHz, Chloroform-*d*): δ 7.30 (d, *J* = 8.4 Hz, 2H), 7.02 (d, *J* = 8.3 Hz, 2H), 4.05 (qd, *J* = 11.5, 7.2 Hz, 2H), 2.08 (s, 3H), 1.87 (dt, *J* = 9.4, 4.9 Hz, 1H), 1.51 – 1.43 (m, 1H), 1.31 (s, 9H), 0.97 (ddt, *J* = 17.8, 8.8, 5.2 Hz, 2H) ppm; **^{13}C NMR (100 MHz, Chloroform-*d*):** δ 171.27, 148.71, 138.97, 125.58, 125.28, 68.14, 34.38, 31.39, 21.40, 21.22, 21.10, 13.94 ppm; **HRMS (ESI, m/z):** calculated for [M+H]+: 247.1698, found 247.1695.

1-methoxy-4-(2-methoxycyclopropyl)benzene (1an)

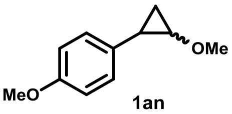

1an

***Cis* isomer ^{1}H NMR (400 MHz, Chloroform-*d*)**: δ 7.17 (d, *J* = 8.6 Hz, 2H), 6.83 (d, *J* = 7.3 Hz, 2H), 3.78 (s, 3H), 3.40 – 3.37 (m, 1H), 3.14 (s, 3H), 1.94 (dt, *J* = 9.6, 6.8 Hz, 1H), 1.08 (dt, *J* = 9.6, 6.4 Hz, 1H), 0.99 (ddd, *J* = 7.2, 6.4, 3.7 Hz, 1H) ppm; **^{13}C NMR**

(100 MHz, Chloroform-*d*): δ 157.94, 133.23, 127.27, 113.90, 63.14, 58.18, 55.38 , 22.86, 15.45 ppm;

***Trans* isomer ^{1}H NMR (400 MHz, Chloroform-d)**: δ 6.98 (d, *J* = 8.6 Hz, 2H), 6.81 (d, *J* = 7.3 Hz, 2H), 3.77 (s, 3H), 3.41 (s, 3H), 3.25 (ddd, *J* = 6.3, 3.5, 2.6 Hz, 1H), 2.05 (ddd, *J* = 10.2, 6.4, 2.5 Hz, 1H), 1.20 (ddd, *J* = 10.3, 6.1, 3.5 Hz, 1H), 0.94 (q, *J* = 6.3 Hz, 1H) ppm; **^{13}C NMR (100 MHz, Chloroform-*d*):** δ 157.86, 129.74 , 129.00, 113.52, 59.77, 58.21, 55.31 , 21.93 , 12.45 ppm; **HRMS (ESI, m/z):** calculated for [M+H]+: 179.2385, found: 179.2379.

2-(2-(4-methoxyphenyl)cyclopropyl)naphthalene (1ar)

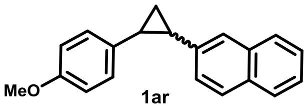

(***Trans*** : ***Cis*** = 0.24 : 1.0)

***Cis* isomer ^{1}H NMR (400 MHz, Chloroform-*d*):** (resolved signals only) δ 7.73 – 7.66 (m, 2H), 7.56 (d, *J* = 8.5 Hz, 1H), 7.48 – 7.46 (m, 1H), 7.42 – 7.34 (m, 2H), 7.02 (dd, *J* = 8.5, 1.7 Hz, 1H), 6.93 (d, *J* = 8.5 Hz, 2H), 6.62 (d, *J* = 8.7 Hz, 2H), 3.67 (s, 3H), 2.55 (dtd, *J* = 24.3, 9.0, 6.5 Hz, 2H), 1.57 – 1.43 (m, 2H) ppm; **^{13}C NMR (100 MHz, Chloroform-*d*):** δ 157.57, 136.51, 133.22, 131.82, 130.06, 127.57, 127.50, 127.43, 127.11, 127.04, 126.95, 125.62, 124.95, 113.21, 55.09, 24.22, 23.94, 11.67 ppm.

***Trans* isomer ^{1}H NMR (400 MHz, Chloroform-*d*):** (resolved signals only) δ 7.83 – 7.78 (m, 2H), 7.77 (d, *J* = 2.1 Hz, 1H), 7.59 (s, 1H), 7.48 (d, *J* = 1.4 Hz, 1H), 7.44 (dd, *J* = 3.9, 1.6 Hz, 1H), 7.28 (dd, *J* = 8.5, 1.8 Hz, 1H), 7.13 (d, *J* = 8.7 Hz, 2H), 6.88 (d, *J* = 8.7 Hz, 2H), 3.81 (s, 3H), 2.26 (ddd, *J* = 8.6, 6.0, 4.0 Hz, 2H), 1.57 – 1.43 (m, 2H) ppm; **^{13}C NMR (100 MHz, Chloroform-*d*):** δ 157.89, 140.23, 134.48, 133.54, 131.97, 130.20, 128.00, 127.65, 127.32, 126.13, 125.07, 124.68, 123.75, 113.91, 55.38, 27.87, 27.53, 17.85 ppm; **HRMS (ESI, m/z):** calculated for $[M+H]^+$: 275.1436, found 275.1440.

6a-ethyl-1,1a,6,6a-tetrahydrocyclopropa[a]indene (1at)

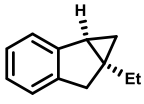

^{1}H NMR (400 MHz, Chloroform-*d*): δ 7.25 (d, *J* = 7.9 Hz, 1H), 7.14 (d, *J* = 7.1 Hz, 1H), 7.12 – 7.03 (m, 2H), 3.01 (s, 2H), 2.14 (dd, *J* = 7.9, 2.8 Hz, 1H), 1.75 (dq, *J* = 14.5, 7.4 Hz, 1H), 1.54 (dq, *J* = 14.7, 7.5 Hz, 1H), 1.03 (t, *J* = 7.4 Hz, 3H), 1.01 – 0.98 (m, 1H), 0.24 (t, *J* = 3.6 Hz, 1H) ppm; **^{13}C NMR (100 MHz, Chloroform-*d*):** δ 147.78, 142.53, 125.84, 125.28, 125.05, 123.02, 39.29, 29.81, 29.56, 28.74, 22.84, 11.81 ppm; **HRMS (ESI, m/z):** calculated for $[M+Na]^+$: 181.0993, found: 181.0995.

1-(4-methoxyphenyl)-1a,2,7,7a-tetrahydro-1H-cyclopropa[b]naphthalene (1aw)

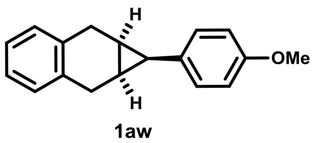

^{1}H NMR (400 MHz, Chloroform-*d*): δ 7.09 – 7.01 (m, 2H), 6.91 – 6.80 (m, 4H), 6.63 (d, *J* = 8.7 Hz, 2H), 3.69 (s, 3H), 3.16 (ddd, *J* = 16.0, 4.3, 2.5 Hz, 2H), 2.70 (dd, *J* = 17.6, 2.0 Hz, 2H), 2.16 (t, *J* = 8.7 Hz, 1H), 1.68 – 1.58 (m, 2H) ppm; **^{13}C NMR (100 MHz, Chloroform-*d*):** δ 157.43 , 137.18 , 131.31 , 129.20 , 127.77 , 125.32 , 113.34 , 55.20 , 26.81 , 23.19 , 15.92 ppm; **HRMS (ESI, m/z):** calculated for $[M+Na]^+$: 273.3308, found: 273.3303.

benzyl 6-(4-methoxyphenyl)bicyclo[3.1.0]hexane-3-carboxylate (1ax)

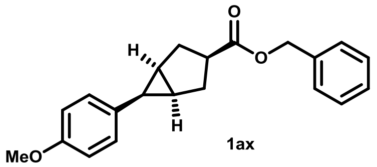

1ax

^{1}H NMR (400 MHz, Chloroform-*d*): δ 7.36 – 7.32 (m, 1H), 7.32 – 7.29 (m, 2H), 7.26 (dd, *J* = 7.6, 1.8 Hz, 2H), 7.17 – 7.13 (m, 2H), 6.84 (d, *J* = 8.6 Hz, 2H), 4.98 (s, 2H), 3.79 (s, 3H), 2.19 – 2.10 (m, 2H), 2.04 (dd, *J* = 13.3, 8.6 Hz, 2H), 1.93 (t, *J* = 8.3 Hz, 1H), 1.75 – 1.63 (m, 2H), 1.50 – 1.35 (m, 1H) ppm; **^{13}C NMR (100 MHz, Chloroform-*d*):** δ 175.20 , 158.08 , 136.23 , 130.25 , 129.45 , 128.49 , 128.08 , 128.06 , 114.11 , 66.02 , 55.21 , 41.55 , 29.68 , 22.41 (d, *J* = 11.0 Hz) ppm; **HRMS (ESI, m/z):** calculated for [M+Na]$^{+}$: 354.3938, found: 354.3944.

1-(2-decylcyclopropyl)-4-methoxybenzene (1ay)

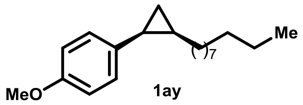

1ay

^{1}H NMR (400 MHz, Chloroform-*d*): δ 7.11 (d, *J* = 8.4 Hz, 2H), 6.82 (d, *J* = 8.7 Hz, 2H), 3.79 (s, 3H), 2.08 – 1.99 (m, 1H), 1.34 – 1.09 (m, 18H), 1.04 – 0.98 (m, 1H), 0.93 (dd, *J* = 8.4, 4.7 Hz, 1H), 0.89 (t, *J* = 6.9 Hz, 3H), 0.55 (q, *J* = 5.5 Hz, 1H) ppm; **^{13}C NMR (100 MHz, Chloroform-*d*):** δ 157.71, 131.86, 130.14, 113.36, 55.36, 32.10, 29.80, 29.78, 29.75, 29.62, 29.58, 29.53, 28.78, 22.87, 20.25, 18.90, 14.32, 9.81 ppm; **HRMS (ESI, m/z):** calculated for [M+H]$^{+}$: 288.2453, found: 288.2446.

2-(2-(4-methoxyphenyl)cyclopropyl)dibenzo[b,e]oxepin-11(6H)-one (1az)

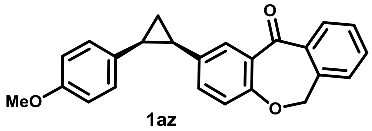

1az

^{1}H NMR (400 MHz, Chloroform-*d*): δ 7.99 (d, *J* = 2.3 Hz, 1H), 7.90 (dd, *J* = 7.7, 1.4 Hz, 1H), 7.56 (td, *J* = 7.4, 1.4 Hz, 1H), 7.47 (td, *J* = 7.6, 1.3 Hz, 1H), 7.36 (d, *J* = 7.4 Hz, 1H), 7.29 (dd, *J* = 8.5, 2.5 Hz, 1H), 7.11 – 7.06 (m, 2H), 6.99 (d, *J* = 8.4 Hz, 1H), 6.85 (d, *J* = 8.7 Hz, 2H), 5.18 (s, 2H), 3.80 (s, 3H), 2.13 (ddq, *J* = 8.9, 6.0, 4.7 Hz, 2H), 1.40 (tdt, *J* = 13.1, 6.9, 3.5 Hz, 2H) ppm; **^{13}C NMR (100 MHz, Chloroform-*d*):** δ 191.16, 159.55, 157.87, 140.49, 136.48, 135.68, 134.29, 133.49, 132.73, 129.50, 129.23, 128.20, 127.77, 126.92, 125.10, 120.71, 113.88, 73.66, 55.36, 27.04, 26.58, 17.67 ppm; **HRMS (ESI, m/z):** calculated for [M+H]$^{+}$: 357.1491, found 357.1482.

1-(3-methoxyphenyl)-3-methyl-3-(1H-pyrazol-1-yl)butan-1-one (3aa)

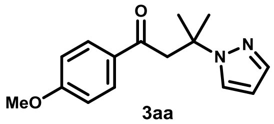

3aa

Following the General Procedure E, **3aa** was obtained in 80% yield as a colorless oil; **^{1}H NMR (400 MHz, Chloroform-*d*):** δ 7.79 (d, *J* = 8.9 Hz, 2H), 7.51 (s, 1H), 7.50 (s, 1H), 6.84 (d, *J* = 8.9 Hz, 2H), 6.15 (t, *J* = 2.1 Hz, 1H), 3.84 (s, 3H), 3.51 (s, 2H), 1.78 (s, 6H) ppm; **^{13}C NMR (100 MHz, Chloroform-*d*):** δ 196.24, 163.44, 138.99, 130.57, 130.43, 126.37, 113.52, 104.58, 59.67, 55.45, 48.76, 27.96 ppm; **HRMS (ESI, m/z):** calculated for [M+H]$^{+}$: 258.1447, found: 259.1441.

1-(4-bromophenyl)-3-methyl-3-(1H-pyrazol-1-yl)butan-1-one (3ba)

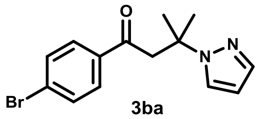

3ba

Following the General Procedure E, **3ba** was obtained in 80% yield as a colorless oil; **^{1}H NMR (400 MHz, Chloroform-*d*):** δ 7.64 (d, *J* = 8.6 Hz, 2H), 7.49 (d, *J* = 5.9 Hz, 2H), 7.47 (s, 1H), 7.47 (d, *J* = 1.7 Hz, 1H), 6.13 (t, *J* = 2.1 Hz, 1H), 3.52 (s, 2H), 1.76 (s, 6H) ppm; **^{13}C NMR (100 MHz, Chloroform-*d*):** δ 196.87, 139.17, 136.13,

131.75, 129.69, 128.34, 126.46, 104.81, 59.61, 49.05, 28.06 ppm; **HRMS (ESI, m/z):** calculated for $[M+H]^+$: 307.0446, found: 307.0441.

3-methyl-3-(1H-pyrazol-1-yl)-1-(p-tolyl)butan-1-one (3ca)

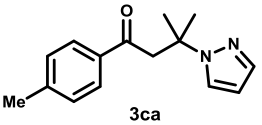

3ca

Following the General Procedure E, **3ca** was obtained in 65% yield as a colorless oil; **^{1}H NMR (400 MHz, Chloroform-*d*):** δ 7.72 (d, *J* = 8.2 Hz, 2H), 7.52 (d, *J* = 2.4 Hz, 1H), 7.50 (d, *J* = 1.7 Hz, 1H), 7.17 (d, *J* = 8.0 Hz, 2H), 6.15 (t, *J* = 2.1 Hz, 1H), 3.54 (s, 2H), 2.36 (s, 3H), 1.78 (s, 6H) ppm; **^{13}C NMR (100 MHz, Chloroform-*d*):** δ 197.41, 143.95, 139.07, 135.04, 129.20, 128.29, 126.46, 104.67, 59.67, 48.95, 28.00, 21.70 ppm; **HRMS (ESI, m/z):** calculated for $[M+H]^+$: 243.1497, found: 243.1493.

1-(4-(tert-butyl)phenyl)-3-methyl-3-(1H-pyrazol-1-yl)butan-1-one (3da)

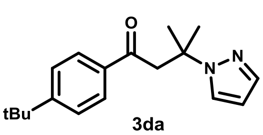

3da

Following the General Procedure E, **3da** was obtained in 77% yield as a white solid, mp 48-50 ℃; **^{1}H NMR (400 MHz, Chloroform-*d*):** δ 7.77 (d, *J* = 8.6 Hz, 2H), 7.53 (dd, *J* = 2.4, 0.7 Hz, 1H), 7.50 (d, *J* = 1.8 Hz, 1H), 7.39 (d, *J* = 8.6 Hz, 2H), 6.16 (t, *J* = 2.1 Hz, 1H), 3.56 (s, 2H), 1.78 (s, 6H), 1.31 (s, 9H) ppm; **^{13}C NMR (100 MHz, Chloroform-*d*):** δ 197.43, 156.86, 139.07, 135.06, 128.15, 126.45, 125.48, 104.66, 59.69, 49.00, 35.16, 31.17, 28.03 ppm; **HRMS (ESI, m/z):** calculated for $[M+H]^+$: 285.1967, found: 285.1965.

3-methyl-1-phenyl-3-(1H-pyrazol-1-yl)butan-1-one (3ea)

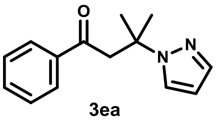

3ea

Following the General Procedure E, **3ea** was obtained in 34% yield as a colorless oil; **^{1}H NMR (400 MHz, Chloroform-*d*):** δ 7.82 (dd, *J* = 8.2, 1.0 Hz, 2H), 7.52 (d, *J* = 2.4 Hz, 1H), 7.48 (dd, *J* = 5.3, 1.4 Hz, 2H), 7.37 (t, *J* = 7.7 Hz, 2H), 6.15 (t, *J* = 2.0 Hz, 1H), 3.58 (s, 2H), 1.78 (s, 6H) ppm; **^{13}C NMR (100 MHz, Chloroform-*d*):** δ 197.81, 139.11, 133.15, 128.53, 128.15, 126.47, 104.71, 59.63, 49.07, 28.03 ppm; **HRMS (ESI, m/z):** calculated for $[M+H]^+$: 229.1341, found: 229.1340.

1-(3-methoxyphenyl)-3-methyl-3-(1H-pyrazol-1-yl)butan-1-one (3fa)

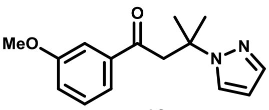

3fa

Following the General Procedure E, **3fa** was obtained in 72% yield as a colorless oil; **^{1}H NMR (400 MHz, Chloroform-*d*):** δ 7.53 (dd, *J* = 2.3, 0.7 Hz, 1H), 7.50 (d, *J* = 1.4 Hz, 1H), 7.41 (dt, *J* = 7.7, 1.3 Hz, 1H), 7.36 (dd, *J* = 2.7, 1.6 Hz, 1H), 7.31 – 7.23 (m, 1H), 7.07 (dd, *J* = 2.7, 1.0 Hz, 1H), 7.05 (dd, *J* = 2.7, 1.0 Hz, 1H), 6.17 (t, *J* = 2.1 Hz, 1H), 3.82 (s, 3H), 3.57 (s, 2H), 1.79 (s, 6H) ppm; **^{13}C NMR (100 MHz, Chloroform-*d*):** δ 197.57, 159.79, 139.11, 138.87, 129.52, 126.46, 120.93, 119.89, 112.06, 104.69, 59.61, 55.50, 49.17, 28.05 ppm; **HRMS (ESI, m/z):** calculated for $[M+H]^+$: 259.1447, found: 259.1442.

1-(3-methoxyphenyl)-3-methyl-3-(1H-pyrazol-1-yl)butan-1-one (3ga)

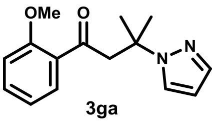

3ga

Following the General Procedure E, **3ga** was obtained in 62% yield as a colorless oil; **^{1}H NMR (400 MHz, Chloroform-*d*):** δ 7.53 (d, *J* = 2.0 Hz, 1H), 7.45 (d, *J* = 1.4 Hz, 1H), 7.43 (dd, *J* = 7.7, 1.8 Hz, 1H), 7.38 (ddd, *J* = 8.4, 7.5, 1.8 Hz, 1H), 6.94 – 6.86 (m, 2H), 6.15 (t, *J* = 2.1 Hz, 1H), 3.86 (s, 3H), 3.61 (s, 2H), 1.75 (s, 6H) ppm; **^{13}C NMR (100 MHz, Chloroform-*d*):** δ 200.36, 158.15, 138.83, 133.30, 130.01, 129.40, 126.48, 120.66,

111.48, 104.47, 59.80, 55.64, 53.95, 28.20 ppm; **HRMS (ESI, m/z):** calculated for $[M+H]^+$: 259.1447, found: 259.1439.

1-([1,1'-biphenyl]-4-yl)-3-methyl-3-(1H-pyrazol-1-yl)butan-1-one (3ha)

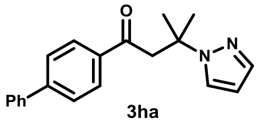

3ha

Following the General Procedure E, **3ha** was obtained in 73% yield as a white soild, mp 108-109 ℃; **^{1}H NMR (400 MHz, Chloroform-*d*):** δ 7.90 (d, *J* = 8.5 Hz, 2H), 7.60 (d, *J* = 8.6 Hz, 4H), 7.54 (d, *J* = 2.3 Hz, 1H), 7.52 (d, *J* = 1.7 Hz, 1H), 7.46 (t, *J* = 7.4 Hz, 2H), 7.39 (t, *J* = 7.3 Hz, 1H), 6.18 – 6.14 (t, *J* = 2.1 Hz, 1H), 3.61 (s, 2H), 1.81 (s, 6H) ppm; **^{13}C NMR (100 MHz, Chloroform-*d*):** δ 197.42, 145.76, 139.92, 139.15, 136.23, 129.04, 128.80, 128.33, 127.36, 127.16, 126.50, 104.76, 59.73, 49.17, 28.09 ppm; **HRMS (ESI, m/z):** calculated for $[M+H]^+$: 305.1654, found: 305.1649.

1-(4-([1,1'-biphenyl]-4-yl)-4-hydroperoxy-2-methylbutan-2-yl)-1H-pyrazole (int-ha)

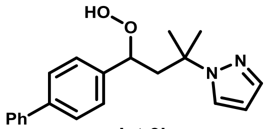

Int-3ha

^{1}H NMR (400 MHz, Chloroform-*d*): δ 7.62 (d, *J* = 2.1 Hz, 2H), 7.59 (d, *J* = 1.5 Hz, 1H), 7.57 (d, *J* = 2.0 Hz, 1H), 7.56 (d, *J* = 8.2 Hz, 2H), 7.45 – 7.39 (m, 4H), 7.34 (t, *J* = 7.3 Hz, 1H), 6.31 (t, *J* = 2.1 Hz, 1H), 4.81 (dd, *J* = 9.8, 2.0 Hz, 1H), 2.42 (dd, *J* = 15.1, 9.9 Hz, 1H), 2.15 (dd, *J* = 15.1, 2.2 Hz, 1H), 1.75 (s, 3H), 1.73 (s, 3H) ppm; **^{13}C NMR (100 MHz, Chloroform-*d*):** δ 144.78, 141.13, 140.27, 139.16, 128.89, 127.33, 127.31, 127.25, 127.07, 126.26, 105.44, 70.53, 60.84, 52.28, 30.03, 28.22 ppm; **HRMS (ESI, m/z):** calculated for $[M+Na]^+$: 345.1579, found: 345.1587.

1-(4-cyclopropylphenyl)-3-methyl-3-(1H-pyrazol-1-yl)butan-1-one (3ia)

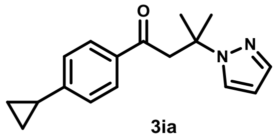

3ia

Following the General Procedure E, **3ia** was obtained in 56% yield as a colorless oil; **^{1}H NMR (400 MHz, Chloroform-*d*):** δ 7.71 (d, *J* = 8.3 Hz, 2H), 7.52 (d, *J* = 2.4 Hz, 1H), 7.50 (d, *J* = 1.6 Hz, 1H), 7.03 (d, *J* = 8.4 Hz, 2H), 6.16 (t, *J* = 2.0 Hz, 1H), 3.53 (s, 2H), 1.90 (ddd, *J* = 13.4, 8.3, 4.9 Hz, 1H), 1.77 (s, 6H), 1.09 – 0.98 (m, 2H), 0.77 – 0.72 (m, 2H) ppm; **^{13}C NMR (100 MHz, Chloroform-*d*):** δ 197.23, 150.44, 139.08, 134.93, 128.38, 126.46, 125.41, 104.68, 59.72, 48.94, 28.03, 15.79, 10.50 ppm; **HRMS (ESI, m/z):** calculated for $[M+H]^+$: 269.1654, found: 269.1649.

1-(3-fluoro-4-methoxyphenyl)-3-methyl-3-(1H-pyrazol-1-yl)butan-1-one (3ja)

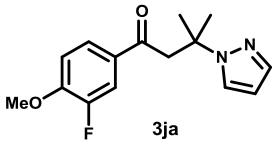

3ja

Following the General Procedure E, **3ja** was obtained in 74% yield as a colorless oil; **^{1}H NMR (400 MHz, Chloroform-*d*):** δ 7.58 – 7.48 (m, 2H), 7.47 (d, *J* = 2.1 Hz, 2H), 6.86 (t, *J* = 8.3 Hz, 1H), 6.12 (t, *J* = 2.1 Hz, 1H), 3.88 (s, 3H), 3.47 (s, 2H), 1.75 (s, 6H) ppm; **^{13}C NMR (100 MHz, Chloroform-*d*)**: δ 195.53 (d, *J* = 1.9 Hz), 152.55 (d, *J* = 115.3 Hz), 151.26 (d, *J* = 121.4 Hz), 139.12 , 130.80 (d, *J* = 4.9 Hz), 126.49 , 125.61 (d, *J* = 3.3 Hz), 115.73 (d, *J* = 19.1 Hz), 112.12 (d, *J* = 1.8 Hz), 104.75 , 59.68 , 56.31 , 48.85 , 28.05 . ppm; **HRMS (ESI, m/z):** calculated for $[M+H]^+$: 277.1352, found: 277.1349.

1-(3-chloro-4-methoxyphenyl)-3-methyl-3-(1H-pyrazol-1-yl)butan-1-one (3ka)

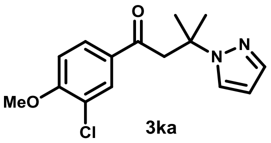

3ka

Following the General Procedure E, **3ka** was obtained in 71% yield as a white solid, mp 65-68 ℃; **^{1}H NMR (400 MHz, Chloroform-*d*):** δ 7.80 (d, *J* = 2.2 Hz, 1H), 7.69 (dd, *J* = 8.7, 2.2 Hz, 1H), 7.49 (d, *J* = 1.8 Hz, 1H), 7.47 (d, *J* = 2.3 Hz, 1H), 6.84 (d, *J* = 8.6 Hz, 1H), 6.12 (t, *J* = 2.1 Hz, 1H), 3.92 (s, 3H), 3.48 (s, 2H), 1.76 (s, 6H) ppm; **^{13}C NMR (100 MHz, Chloroform-*d*):** δ 195.51, 158.80, 139.19, 131.08, 130.52, 128.71, 126.52, 122.77, 111.10, 104.78, 59.71, 56.44, 48.98, 28.08 ppm; **HRMS (ESI, m/z):** calculated for $[M+H]^+$: 293.1057, found: 293.1059.

1-(3-bromo-4-methoxyphenyl)-3-methyl-3-(1H-pyrazol-1-yl)butan-1-one (3la)

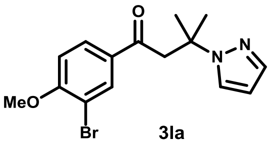

3la

Following the General Procedure E, **3la** was obtained in 82% yield as a white solid, mp 84-91 ℃; **^{1}H NMR (400 MHz, Chloroform-*d*):** δ 7.35 (d, *J* = 0.7 Hz, 1H), 7.05 (dd, *J* = 8.4, 0.8 Hz, 1H), 6.80 (d, *J* = 8.4 Hz, 2H), 3.87 (s, 5H), 1.84 – 1.74 (m, 3H), 1.20 (s, 3H), 0.78 (s, 3H), 0.75 (dd, *J* = 8.4, 4.8 Hz, 2H), 0.70 (t, *J* = 5.2 Hz, 2H) ppm; **^{13}C NMR (100 MHz, Chloroform-*d*):** δ 195.39, 159.63, 139.20, 133.68, 131.50, 129.43, 126.52, 111.83, 110.94, 104.79, 59.70, 56.54, 48.98, 28.08 ppm; **HRMS (ESI, m/z):** calculated for $[M+H]^+$: 337.0552, found: 337.0546.

1-(4-methoxy-3-methylphenyl)-3-methyl-3-(1H-pyrazol-1-yl)butan-1-one (3ma)

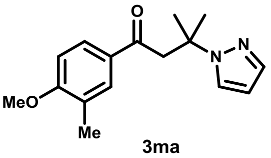

3ma

Following the General Procedure E, **3ma** was obtained in 48% yield as a colorless oil; **^{1}H NMR (400 MHz, Chloroform-*d*):** δ 7.67 (dd, *J* = 8.6, 2.3 Hz, 1H), 7.59 (d, *J* = 2.5 Hz, 1H), 7.50 (dd, *J* = 7.7, 2.1 Hz, 2H), 6.74 (d, *J* = 8.6 Hz, 1H), 6.14 (t, *J* = 2.1 Hz, 1H), 3.85 (s, 3H), 3.50 (s, 2H), 2.17 (s, 3H), 1.77 (s, 6H) ppm; **^{13}C NMR (100 MHz, Chloroform-*d*):** δ 196.66, 161.84, 139.03, 130.82, 130.10, 128.39, 126.54, 109.10, 104.63, 59.78, 55.57, 48.90, 28.03, 16.26 ppm; **HRMS (ESI, m/z):** calculated for $[M+H]^+$: 273.1603, found: 273.1604.

1-(3,4-dimethoxyphenyl)-3-methyl-3-(1H-pyrazol-1-yl)butan-1-one (3na)

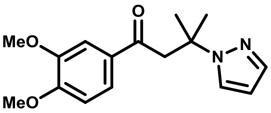

3na

Following the General Procedure E, **3na** was obtained in 42% yield as a colorless oil; **^{1}H NMR (400 MHz, Chloroform-*d*):** δ 7.50 (d, *J* = 2.3 Hz, 2H), 7.42 (dd, *J* = 8.4, 2.1 Hz, 1H), 7.38 (d, *J* = 2.0 Hz, 1H), 6.77 (d, *J* = 8.3 Hz, 1H), 6.14 (t, *J* = 2.1 Hz, 1H), 3.90 (s, 3H), 3.88 (s, 3H), 3.52 (s, 2H), 1.77 (s, 6H) ppm; **^{13}C NMR (100 MHz, Chloroform-*d*):** δ 196.38, 153.38, 148.92, 139.10, 130.77, 126.55, 123.11, 110.10, 109.87, 59.75, 56.00, 48.68, 28.11 ppm; **HRMS (ESI, m/z):** calculated for $[M+H]^+$: 289.1552, found: 289.1551.

Methyl 2-methoxy-5-(3-methyl-3-(1H-pyrazol-1-yl)butanoyl)benzoate (3oa)

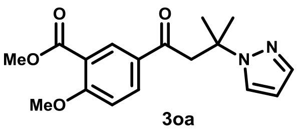

3oa

Following the General Procedure E, **3oa** was obtained in 71% yield as a white solid, mp 55-59 ℃; **^{1}H NMR (400 MHz, Chloroform-*d*):** δ 8.23 (d, *J* = 2.4 Hz, 1H), 7.93 (dd, *J* = 8.8, 2.4 Hz, 1H), 7.48 (d, *J* = 2.4 Hz, 1H), 7.47 (d, *J* = 1.7 Hz, 1H), 6.92 (d, *J* = 8.9 Hz, 1H), 6.12 (t, *J* = 2.1 Hz, 1H), 3.94 (s, 3H), 3.89 (s, 3H), 3.53 (s, 2H), 1.77 (s, 6H) ppm; **^{13}C NMR (100 MHz, Chloroform-*d*):** δ 195.72, 165.84, 162.61, 139.12, 133.78, 132.35, 129.79, 126.51, 119.80, 111.57, 104.71, 59.65, 56.37,

52.31, 48.89, 28.07 ppm; **HRMS (ESI, m/z):** calculated for $[M+H]^+$: 317.1501, found: 317.1495.

1-(3,4-dimethylphenyl)-3-methyl-3-(1H-pyrazol-1-yl)butan-1-one (3pa)

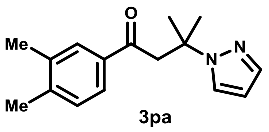

3pa

Following the General Procedure E, **3pa** was obtained in 60% yield as a colorless oil; **^{1}H NMR (400 MHz, Chloroform-*d*):** δ 7.56 (d, *J* = 6.4 Hz, 2H), 7.51 (d, *J* = 2.3 Hz, 2H), 7.12 (d, *J* = 8.5 Hz, 1H), 6.15 (t, *J* = 2.1 Hz, 1H), 3.54 (s, 2H), 2.27 (s, 3H), 2.25 (s, 3H), 1.78 (s, 6H) ppm; **^{13}C NMR (100 MHz, Chloroform-*d*):** δ 197.68, 142.72, 139.06, 136.84, 135.43, 129.76, 129.31, 126.51, 125.92, 104.66, 59.71, 49.08, 28.02, 20.12, 19.82 ppm; **HRMS (ESI, m/z):** calculated for $[M+H]^+$: 257.1654, found: 257.1651.

1-(4-(3,4-dimethylphenyl)-4-hydroperoxy-2-methylbutan-2-yl)-1H-pyrazole (Int-3pa)

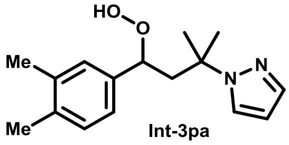

Int-3pa

^{1}H NMR (400 MHz, Chloroform-*d*): δ 8.44 (s, 1H), 7.57 (d, *J* = 2.4 Hz, 1H), 7.56 (d, *J* = 1.5 Hz, 1H), 7.09 (d, *J* = 7.5 Hz, 1H), 7.00 (s, 1H), 6.97 (d, *J* = 7.6 Hz, 1H), 6.27 (t, *J* = 2.1 Hz, 1H), 4.57 (dd, *J* = 9.0, 3.1 Hz, 1H), 2.28 (d, *J* = 3.0 Hz, 1H), 2.23 (s, 6H), 1.72 (s, 3H), 1.62 (s, 3H). **^{13}C NMR (100 MHz, Chloroform-*d*):** δ 139.19, 138.53, 136.92, 136.61, 129.90, 127.94, 126.88, 124.15, 104.99, 84.51, 60.08, 46.75, 29.03, 28.20, 19.94, 19.61 ppm.

3-methyl-1-(naphthalen-2-yl)-3-(1H-pyrazol-1-yl)butan-1-one (3qa)

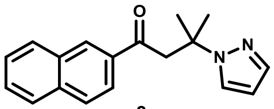

3qa

Following the General Procedure E, **3qa** was obtained in 46% yield as a white solid, mp 55-57 ℃; **^{1}H NMR (400 MHz, Chloroform-*d*):** δ 8.24 (s, 1H), 7.91 (dd, *J* = 8.8, 1.9 Hz, 2H), 7.88 (d, *J* = 7.6 Hz, 2H), 7.81 (t, *J* = 7.7 Hz, 1H), 7.61 – 7.52 (m, 2H), 7.52 – 7.50 (d, 1H), 6.11 (t, *J* = 2.1 Hz, 1H), 3.70 (s, 2H), 1.83 (s, 6H) ppm; **^{13}C NMR (100 MHz, Chloroform-*d*):** δ 197.86, 139.22, 135.61, 134.79, 132.50, 130.19, 129.80, 128.58, 128.35, 127.78, 126.78, 126.59, 123.76, 104.81, 59.85, 49.40, 28.14 ppm; **HRMS (ESI, m/z):** calculated for $[M+H]^+$: 279.1497, found: 279.1493.

1-(benzofuran-5-yl)-3-methyl-3-(1H-pyrazol-1-yl)butan-1-one (3ra)

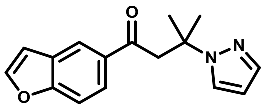

3ra

Following the General Procedure E, **3ra** was obtained in 50% yield as a white solid, mp 47-53 ℃; **^{1}H NMR (400 MHz, Chloroform-*d*):** δ 8.08 (d, *J* = 1.8 Hz, 1H), 7.83 (dd, *J* = 8.8, 1.9 Hz, 1H), 7.65 (d, *J* = 2.2 Hz, 1H), 7.50 (d, *J* = 2.1 Hz, 2H), 7.45 (d, *J* = 8.8 Hz, 1H), 6.80 (dd, *J* = 2.2, 0.9 Hz, 1H), 6.12 (t, *J* = 2.1 Hz, 1H), 3.63 (s, 2H), 1.80 (s, 6H) ppm; **^{13}C NMR (100 MHz, Chloroform-*d*):** δ 197.36, 157.55, 146.40, 139.12, 133.13, 127.46, 126.54, 124.90, 122.64, 111.42, 107.41, 104.73, 59.82, 49.34, 28.09 ppm; **HRMS (ESI, m/z):** calculated for $[M+H]^+$: 269.1290, found: 269.1288.

1-(1a,6b-dihydro-1H-cyclopropa[b]benzofuran-5-yl)-3-methyl-3-(1H-pyrazol-1-yl)butan-1-one (3sa)

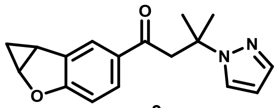

3sa

Following the General Procedure E, **3sa** was obtained in 39% yield as a white solid, mp 47-52 ℃; **^{1}H NMR (400 MHz, Chloroform-*d*):** δ 7.80 (d, *J* = 2.0 Hz, 1H), 7.62 (dd, *J* = 8.5,

2.0 Hz, 1H), 7.50 (d, *J* = 1.4 Hz, 1H), 7.49 (d, *J* = 2.4 Hz, 1H), 6.74 (d, *J* = 8.4 Hz, 1H), 6.14 (t, *J* = 2.1 Hz, 1H), 4.86 (td, *J* = 5.4, 1.8 Hz, 1H), 3.50 (d, *J* = 3.5 Hz, 2H), 2.59 (dt, *J* = 9.2, 4.7 Hz, 1H), 1.77 (s, 6H), 1.06 (ddd, *J* = 8.9, 6.6, 5.5 Hz, 1H), 0.29 (ddd, *J* = 6.4, 4.1, 1.8 Hz, 1H) ppm; **^{13}C NMR (100 MHz, Chloroform-*d*):** δ 196.38, 163.48, 139.04, 131.74, 130.99, 129.03, 126.55, 124.49, 109.92, 104.64, 62.97, 59.78, 49.05, 28.07, 28.01, 19.39, 10.38 ppm; **HRMS (ESI, m/z):** calculated for $[M+H]^+$: 283.147, found: 283.145.

2-(3-methyl-3-(1H-pyrazol-1-yl)butanoyl)dibenzo[b,e]oxepin-11(6H)-one (3ta)

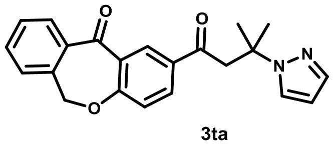

3ta

Following the General Procedure E, **3ta** was obtained in 74% yield as a white solid, mp 116-121 ℃; **^{1}H NMR (400 MHz, Chloroform-*d*):** δ 8.68 (d, *J* = 2.4 Hz, 1H), 7.95 (dd, *J* = 8.7, 2.4 Hz, 1H), 7.85 (dd, *J* = 7.7, 1.4 Hz, 1H), 7.58 (td, *J* = 7.4, 1.4 Hz, 1H), 7.53 (d, *J* = 2.4 Hz, 1H), 7.50 (dd, *J* = 7.6, 1.3 Hz, 1H), 7.47 (dd, *J* = 5.9, 1.5 Hz, 1H), 7.38 (d, *J* = 7.4 Hz, 1H), 7.02 (d, *J* = 8.7 Hz, 1H), 6.13 (t, *J* = 2.1 Hz, 1H), 5.22 (s, 2H), 3.62 (s, 2H), 1.79 (s, 6H) ppm; **^{13}C NMR (100 MHz, Chloroform-*d*):** δ 195.80, 190.54, 164.46, 140.50, 138.96, 134.58, 134.32, 133.35, 133.04, 131.53, 129.65, 129.26, 128.05, 126.38, 124.25, 121.21, 104.57, 73.45, 59.49, 48.77, 28.05 ppm; **HRMS (ESI, m/z):** calculated for $[M+H]^+$: 361.1552, found: 361.1545.

1-(4-methoxyphenyl)-3-(1H-pyrazol-1-yl)propan-1-one (3ua)

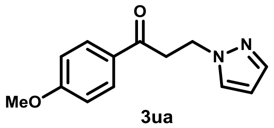

3ua

Following the General Procedure E, **3ua** was obtained in 60% yield as a colorless oil; **^{1}H NMR (400 MHz, Chloroform-*d*):** δ 7.89 (d, *J* = 8.9 Hz, 2H), 7.48 (d, *J* = 2.1 Hz, 2H), 6.89 (d, *J* = 8.9 Hz, 2H), 6.18 (t, *J* = 2.1 Hz, 1H), 4.57 (t, *J* = 6.6 Hz, 2H), 3.83 (s, 3H), 3.51 (t, *J* = 6.6 Hz, 2H) ppm;

^{13}C NMR (100 MHz, Chloroform-*d*): δ 196.03 , 163.86 , 139.64 , 130.45 , 130.22 , 129.57 , 113.90 , 105.34 , 55.59 , 46.87 , 38.57 . ppm; **HRMS (ESI, m/z):** calculated for [M+H]+: 231.1134, found: 231.1131.

3-ethyl-1-(4-methoxyphenyl)-3-(1H-pyrazol-1-yl)pentan-1-one (3va)

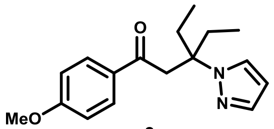

3va

Following the General Procedure E, **3va** was obtained in 44% yield as a colorless oil; **^{1}H NMR (400 MHz, Chloroform-*d*):** δ 7.81 (d, *J* = 9.0 Hz, 2H), 7.50 – 7.49 (dd, *J* = 2.4 Hz, 1H), 7.48 (d, *J* = 1.8 Hz, 1H), 6.83 (d, *J* = 9.0 Hz, 2H), 6.16 – 6.10 (m, 1H), 3.83 (s, 3H), 3.58 (s, 2H), 2.26 – 2.13 (m, 4H), 0.76 (t, *J* = 7.4 Hz, 6H) ppm; **^{13}C NMR (100 MHz, Chloroform-*d*):** δ 196.42, 163.47, 138.65, 130.80, 130.41, 127.47, 113.57, 104.46, 66.06, 55.53, 43.41, 29.06, 7.84 ppm; **HRMS (ESI, m/z):** calculated for $[M+H]^+$: 287.1760, found: 287.1758.

3,3-dicyclopropyl-1-(4-methoxyphenyl)-3-(1H-pyrazol-1-yl)propan-1-one (3wa)

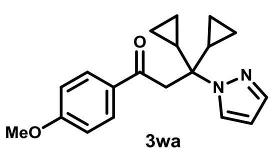

3wa

Following the General Procedure E, **3wa** was obtained in 69% yield as a white solid, mp 77-83 ℃; **^{1}H NMR (400 MHz, Chloroform-*d*):** δ 7.95 (d, *J* = 8.9 Hz, 2H), 7.93 (d, *J* = 0.7 Hz, 1H), 7.38 (d, *J* = 1.2 Hz, 1H), 6.89 (d, *J* = 8.9 Hz, 2H), 6.21 (t, *J* = 2.1 Hz, 1H), 3.86 (s, 2H), 3.84 (s, 3H), 1.45 – 1.34 (m, 2H), 0.78 (td, *J* = 10.0, 5.7 Hz, 2H), 0.59 – 0.47 (m, 2H), 0.49 – 0.37 (m, 2H), 0.16 (dq, *J* = 10.5, 5.5 Hz, 2H) ppm;

^{13}C NMR (100 MHz, Chloroform-*d*): δ 196.01, 163.31, 137.93, 131.16, 130.49, 128.79, 113.58, 104.34, 63.35, 55.52, 46.77, 17.54, 2.44, 1.69 ppm; **HRMS (ESI, m/z):** calculated for $[M+H]^+$: 311.1760, found: 311.1753.

2-(1-(1H-pyrazol-1-yl)cyclopropyl)-1-(4-methoxyphenyl)ethan-1-one (3xa)

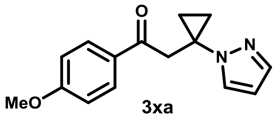

3xa

Following the General Procedure E, **3xa** was obtained in 42% yield as a white soild, mp 84-88 ℃; **^{1}H NMR (400 MHz, Chloroform-*d*):** δ 7.75 (d, *J* = 8.9 Hz, 2H), 7.43 (d, *J* = 1.9 Hz, 1H), 7.22 (d, *J* = 2.3 Hz, 1H), 6.90 (d, *J* = 8.9 Hz, 2H), 6.14 (t, *J* = 2.1 Hz, 1H), 4.51 (s, 2H), 3.85 (s, 3H), 1.33 – 1.26 (m, 2H), 1.13 – 1.05 (m, 2H) ppm; **^{13}C NMR (100 MHz, Chloroform-*d*):** δ 199.57, 163.09, 139.47, 130.83, 129.26, 113.84, 105.73, 57.38, 55.54, 30.91, 12.08 ppm; **HRMS (ESI, m/z):** calculated for $[M+H]^+$: 257.1290, found: 257.1288.

2-(1-(1H-pyrazol-1-yl)cyclobutyl)-1-(4-methoxyphenyl)ethan-1-one (3ya)

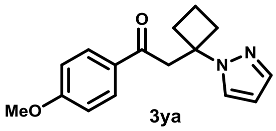

3ya

Following the General Procedure E, **3ya** was obtained in 47% yield as a white soild, mp 84-86 ℃; **^{1}H NMR (400 MHz, Chloroform-*d*):** δ 7.74 (d, *J* = 8.9 Hz, 2H), 7.53 (d, *J* = 2.3 Hz, 1H), 7.46 – 7.44 (d, *J* = 1.3 Hz, 1H), 6.82 (d, *J* = 8.9 Hz, 2H), 6.09 (t, *J* = 2.0 Hz, 1H), 3.83 (s, 3H), 3.65 (s, 2H), 2.78 – 2.68 (m, 2H), 2.64 (td, *J* = 8.2, 4.2 Hz, 2H), 2.01 (ddd, *J* = 11.9, 9.0, 4.9 Hz, 2H) ppm; **^{13}C NMR (100 MHz, Chloroform-*d*):** δ 196.43, 163.62, 139.30, 130.35, 128.00, 113.63, 104.59, 62.73, 55.54, 46.00, 33.31, 15.04 ppm (only 12 carbon resonances were observed do to overlapping resonances); **HRMS (ESI, m/z):** calculated for $[M+H]^+$: 271.1447, found: 271.1442.

2-(1-(1H-pyrazol-1-yl)cyclopentyl)-1-(4-methoxyphenyl)ethan-1-one (3za)

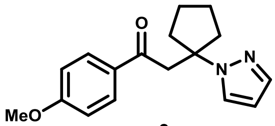

3za

Following the General Procedure E, **3za** was obtained in 47% yield as a white soild, mp 89-96 ℃; **^{1}H NMR (400 MHz, Chloroform-*d*):** δ 7.71 (d, *J* = 8.9 Hz, 2H), 7.47 (d, *J* = 2.4 Hz, 1H), 7.46 (d, *J* = 1.5 Hz, 1H), 6.80 (d, *J* = 8.9 Hz, 2H), 6.06 (t, *J* = 2.1 Hz, 1H), 3.82 (s, 3H), 3.54 (s, 2H), 2.59 (ddd, *J* = 12.2, 5.8, 2.0 Hz, 2H), 2.14 (dt, *J* = 16.0, 8.3 Hz, 2H), 1.83 – 1.76 (m, 2H), 1.69 – 1.62 (m, 2H) ppm; **^{13}C NMR (100 MHz, Chloroform-*d*):** δ 196.83, 163.51, 139.26, 130.46, 128.03, 113.55, 104.50, 70.63, 55.52, 47.00, 38.03, 22.75 ppm (only 12 carbon resonances were observed do to overlapping resonances); **HRMS (ESI, m/z):** calculated for $[M+H]^+$: 285.1603, found: 285.1599.

2-(1-(1H-pyrazol-1-yl)cyclohexyl)-1-(4-methoxyphenyl)ethan-1-one (3aaa)

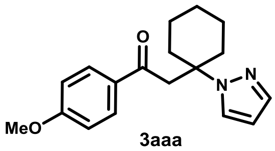

3aaa

Following the General Procedure E, **3aaa** was obtained in 49% yield as a white soild, mp 88-97 ℃; **^{1}H NMR (400 MHz, Chloroform-*d*):** δ 7.63 (d, *J* = 9.0 Hz, 2H), 7.47 (d, *J* = 1.6 Hz, 1H), 7.42 (d, *J* = 2.2 Hz, 1H), 6.76 (d, *J* = 8.9 Hz, 2H), 6.06 (t, *J* = 2.1 Hz, 1H), 3.81 (s, 3H), 3.30 (s, 2H), 2.52 (d, *J* = 15.3 Hz, 2H), 2.05 (dt, *J* = 13.5, 7.0 Hz, 2H), 1.63 (dd, *J* = 10.5, 5.0 Hz, 2H), 1.52 – 1.41 (m, 2H), 1.42 – 1.33 (m, 2H) ppm; **^{13}C NMR (100 MHz, Chloroform-*d*):** δ 196.97, 163.48, 139.01, 130.87, 130.54, 127.89, 113.46, 104.73, 62.37, 55.50, 48.82, 35.75, 25.29, 21.89 ppm; **HRMS (ESI, m/z):** calculated for $[M+H]^+$: 299.1760, found: 299.1755.

1-(4-methoxyphenyl)-3-(1H-pyrazol-1-yl)butan-1-one (3aba)

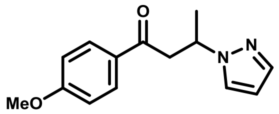

3aba

Following the General Procedure E, **3aba** was obtained in 47% yield as a colorless oil; **^{1}H NMR (400 MHz, Chloroform-*d*):** δ 7.89 (d, *J* = 9.0 Hz, 2H), 7.49 (s, 1H), 7.47 (dd, *J* = 2.2, 0.8 Hz, 1H), 6.89 (d, *J* = 9.0 Hz, 2H), 6.17 – 6.15 (m, 1H), 5.04 (q, *J* = 6.7 Hz, 1H), 3.84 (s, 3H), 3.71 (dd, *J* = 17.0, 6.7 Hz, 1H), 3.27 (dd, *J* = 17.1, 6.4 Hz, 1H), 1.60 (d, *J* = 6.8 Hz, 3H) ppm; **^{13}C NMR (100 MHz, Chloroform-*d*):** δ 196.02, 163.79, 139.43, 130.51, 129.80, 128.76, 113.84, 104.79, 55.59, 53.83, 45.02, 21.47 ppm; **HRMS (ESI, m/z):** calculated for [M+H]$^+$: 245.1288, found: 245.1286.

1-(3-bromo-4-methoxyphenyl)-3-(1H-pyrazol-1-yl)butan-1-one (3aca)

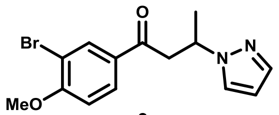

3aca

Following the General Procedure E, **3aca** was obtained in 65% yield as a white solid, mp 83-90℃; **^{1}H NMR (400 MHz, Chloroform-*d*):** δ 8.12 (d, *J* = 2.2 Hz, 1H), 7.87 (dd, *J* = 8.6, 2.2 Hz, 1H), 7.50 (d, *J* = 1.6 Hz, 1H), 7.46 (d, *J* = 2.2 Hz, 1H), 6.89 (d, *J* = 8.7 Hz, 1H), 6.17 (t, *J* = 2.0 Hz, 1H), 5.03 (q, *J* = 6.7 Hz, 1H), 3.95 (s, 3H), 3.72 (dd, *J* = 17.1, 6.8 Hz, 1H), 3.24 (dd, *J* = 17.1, 6.2 Hz, 1H), 1.61 (d, *J* = 6.8 Hz, 3H) ppm; **^{13}C NMR (100 MHz, Chloroform-*d*):** δ 195.05, 159.91, 139.53, 133.73, 130.75, 129.43, 128.77, 112.10, 111.15, 104.88, 56.61, 53.76, 45.00, 21.48 ppm; **HRMS (ESI, m/z):** calculated for [M+H]$^+$: 323.0395, found: 323.0391.

7-(3-bromo-4-methoxyphenyl)-7-oxo-5-(1H-pyrazol-1-yl)heptyl acetate (3ada)

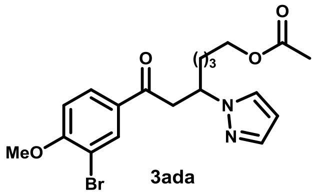

3ada

Following the General Procedure E, **3ada** was obtained in 62% yield as a white solid, mp 122-127 ℃; **^{1}H NMR (400 MHz, Chloroform-*d*):** δ 8.10 (d, *J* = 2.1 Hz, 1H), 7.84 (dd, *J* = 8.6, 2.2 Hz, 1H), 7.49 (d, *J* = 1.8 Hz, 1H), 7.44 (d, *J* = 2.1 Hz, 1H), 6.87 (d, *J* = 8.7 Hz, 1H), 6.14 (t, *J* = 2.1 Hz, 1H), 4.81 (ddt, *J* = 9.9, 7.3, 4.9 Hz, 1H), 3.98 (td, *J* = 6.6, 4.9 Hz, 2H), 3.93 (s, 3H), 3.72 (dd, *J* = 17.2, 7.4 Hz, 1H), 3.24 (dd, *J* = 17.2, 5.4 Hz, 1H), 2.14 – 2.05 (m, 1H), 2.00 (s, 3H), 1.82 (ddt, *J* = 14.1, 10.2, 5.1 Hz, 1H), 1.65 – 1.52 (m, 2H), 1.25 (ddt, *J* = 11.5, 5.6, 2.4 Hz, 1H), 1.07 (ddt, *J* = 13.3, 9.8, 5.1 Hz, 1H) ppm; **^{13}C NMR (100 MHz, Chloroform-*d*):** δ 194.98, 171.16, 159.81, 139.78, 133.61, 130.55, 130.18, 129.34, 111.98, 111.04, 104.50, 64.11, 57.98, 56.52, 43.68, 34.89, 28.03, 22.56, 20.99 ppm; **HRMS (ESI, m/z):** calculated for [M+H]$^+$: 423.0919, found: 423.0913.

7-(3-bromo-4-methoxyphenyl)-7-oxo-5-(1H-pyrazol-1-yl)heptyl dimethylcarbamate (3aea)

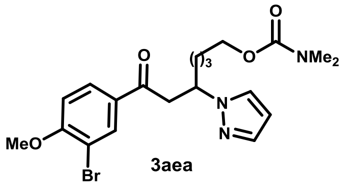

3aea

Following the General Procedure E, **3aea** was obtained in 64% yield as a white solid, mp 119-125 ℃ ; **^{1}H NMR (400 MHz, Chloroform-*d*):** δ 8.09 (d, *J* = 2.2 Hz, 1H), 7.84 (dd, *J* = 8.6, 2.2 Hz, 1H), 7.48 (d, *J* = 1.8 Hz, 1H), 7.43 (d, *J* = 2.3 Hz, 1H), 6.87 (d, *J* = 8.7 Hz, 1H), 6.12 (t, *J* = 2.0 Hz, 1H), 4.81 (ddt, *J* = 9.9, 7.5, 4.9 Hz, 1H), 3.97 (td, *J* = 6.5, 4.5 Hz, 2H), 3.92 (s, 3H), 3.72 (dd, *J* = 17.2, 7.6 Hz, 1H), 3.23 (dd, *J* = 17.2, 5.3 Hz, 1H), 2.84 (d, *J* = 21.6 Hz, 6H), 2.09 (ddd, *J* = 19.4, 10.3, 5.1 Hz, 1H), 1.83 (dt, *J* = 9.8, 5.7 Hz, 1H), 1.59 (dddt, *J* = 29.3, 13.7, 9.3, 4.9 Hz, 2H), 1.30 – 1.16 (m, 1H), 1.08 (ddt, *J* = 18.4, 10.0, 5.2 Hz, 1H) ppm; **^{13}C NMR (100 MHz, Chloroform-*d*):** δ 195.00, 159.79, 156.65,

139.71, 133.60, 130.56, 130.16, 129.34, 111.96, 111.03, 104.44, 64.87, 57.99, 56.51, 43.66, 36.35, 35.81, 34.93, 28.52, 22.54 ppm; **HRMS (ESI, m/z):** calculated for $[M+H]^+$: 452.1185, found: 452.1182.

1-(3-bromo-4-methoxyphenyl)-7-((tert-butyldimethylsilyl)oxy)-3-(1H-pyrazol-1-yl)heptan-1-one (3afa)

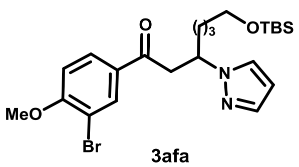

3afa

Following the General Procedure E, **3afa** was obtained in 62% yield as a white solid, mp 121-128 ℃; **^{1}H NMR (400 MHz, Chloroform-*d*):** δ 8.11 (d, *J* = 2.2 Hz, 1H), 7.85 (dd, *J* = 8.7, 2.2 Hz, 1H), 7.49 (d, *J* = 1.8 Hz, 1H), 7.44 (d, *J* = 1.9 Hz, 1H), 6.87 (d, *J* = 8.7 Hz, 1H), 6.13 (t, *J* = 2.1 Hz, 1H), 4.81 (ddt, *J* = 9.9, 7.6, 4.9 Hz, 1H), 3.93 (s, 3H), 3.74 (dd, *J* = 17.1, 7.7 Hz, 1H), 3.53 (td, *J* = 6.5, 2.7 Hz, 2H), 3.23 (dd, *J* = 17.1, 5.2 Hz, 1H), 2.06 (ddt, *J* = 18.7, 10.1, 5.0 Hz, 1H), 1.87 – 1.78 (m, 1H), 1.48 (dddd, *J* = 25.6, 15.3, 7.9, 4.8 Hz, 2H), 1.24 (dddd, *J* = 13.4, 9.7, 6.7, 3.9 Hz, 1H), 1.13 – 1.03 (m, 1H), 0.85 (s, 9H), -0.00 (s, 6H) ppm; **^{13}C NMR (100 MHz, Chloroform-*d*):** δ 195.11, 159.76, 139.68, 133.63, 130.64, 130.19, 129.35, 111.96, 111.01, 104.38, 62.82, 58.14, 56.50, 43.63, 35.19, 32.23, 25.96, 22.50, 18.33, -5.29 ppm; **HRMS (ESI, m/z):** calculated for [M+H]+: 495.1679, found: 495.1675.

6-bromo-1-(4-methoxyphenyl)-3-(1H-pyrazol-1-yl)hexan-1-one (3aga)

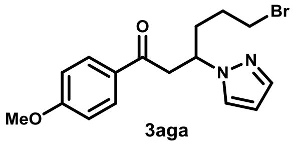

3aga

Following the General Procedure E, **3aga** was obtained in 50% yield as a white solid, mp 129-137 ℃; **^{1}H NMR (400 MHz, Chloroform-*d*):** δ 7.87 (d, *J* = 8.9 Hz, 2H), 7.50 (d, *J* = 1.8 Hz, 1H), 7.46 (d, *J* = 2.2 Hz, 1H), 6.88 (d, *J* = 8.9 Hz, 2H), 6.15 (t, *J* = 2.1 Hz, 1H), 4.91 – 4.81 (m, 1H), 3.83 (s, 3H), 3.73 (dd, *J* = 17.3, 7.3 Hz, 1H), 3.33 – 3.29 (m, 2H), 3.30 – 3.24 (m, 1H), 2.18 (ddt, *J* = 15.2, 10.4, 5.2 Hz, 1H), 2.01 (dp, *J* = 14.0, 5.2 Hz, 1H), 1.82 – 1.69 (m, 1H), 1.63 – 1.49 (m, 1H) ppm; **^{13}C NMR (100 MHz, Chloroform-*d*):** δ 195.63, 163.75, 139.85, 130.41, 130.11, 129.56, 113.76, 104.65, 57.53, 55.51, 43.72, 33.90, 32.92, 29.40 ppm; **HRMS (ESI, m/z):** calculated for $[M+H]^+$: 351.0708, found: 351.0710.

methyl 12-(4-methoxyphenyl)-12-oxo-10-(1H-pyrazol-1-yl)dodecanoate (3aha)

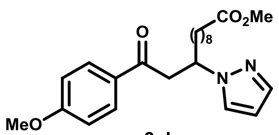

3aha

Following the General Procedure E, **3aha** was obtained in 45% yield as a white solid, mp 65-67 ℃; **^{1}H NMR (400 MHz, Chloroform-*d*) δ** 7.88 (d, *J* = 8.9 Hz, 2H), 7.51 (d, *J* = 1.8 Hz, 1H), 7.46 (d, *J* = 2.2 Hz, 1H), 6.87 (d, *J* = 8.9 Hz, 2H), 6.14 (t, *J* = 2.1 Hz, 1H), 4.88 – 4.73 (m, 1H), 3.83 (s, 3H), 3.75 (dd, *J* = 17.2, 7.6 Hz, 1H), 3.64 (s, 3H), 3.27 (dd, *J* = 17.2, 5.4 Hz, 1H), 2.26 (t, *J* = 7.6 Hz, 2H), 2.12 – 2.00 (m, 1H), 1.79 (ddt, *J* = 14.4, 9.6, 5.0 Hz, 1H), 1.56 (p, *J* = 7.5 Hz, 2H), 1.32 – 1.12 (m, 10H), 1.00 (tt, *J* = 10.2, 4.9 Hz, 1H) ppm; **^{13}C NMR (100 MHz, Chloroform-*d*):** δ 196.14 , 174.33 , 163.73 , 139.55 , 130.46 , 130.13 , 129.81 , 113.76 , 104.34 , 58.33 , 55.51 , 51.48 , 43.75 , 35.39 , 34.11 , 29.24 , 29.12 , 29.09 , 29.05 , 26.13 , 24.93 ppm; **HRMS (ESI, m/z):** calculated for [M+H]+: 402.2468, found: 402.2471.

1-(4-methoxyphenyl)-4-phenyl-3-(1H-pyrazol-1-yl)butan-1-one (3aia)

Following the General Procedure E, **3aia** was obtained in 42% yield as a white solid, mp 72-77 ℃; **^{1}H NMR (400 MHz, Chloroform-*d*):** δ 7.91 (d, *J* = 9.0 Hz, 2H), 7.52 (d, *J* = 1.8 Hz, 1H), 7.24 – 7.16 (m, 3H), 7.12 (d, *J* = 2.0 Hz, 1H), 6.98 (d, *J* = 1.7 Hz, 1H), 6.97 (s, 1H), 6.90 (d, *J* = 8.9 Hz, 2H), 6.02 (t, *J* = 2.1 Hz, 1H), 5.03 (ddt, *J* = 10.6, 7.2, 5.4 Hz, 1H), 3.85 (s, 3H), 3.85 (dd, *J* = 17.3, 7.5 Hz, 1H), 3.41 (dd, *J* = 17.3, 5.5 Hz, 1H), 3.29 (dd, *J* = 13.5, 9.4 Hz, 1H), 3.16 (dd, *J* = 13.5, 5.2 Hz, 1H) ppm; **^{13}C NMR (100 MHz, Chloroform-*d*):** δ 195.97, 163.85, 139.75, 137.99, 130.59, 130.54, 129.82, 129.13, 128.49, 126.72, 113.86, 104.28, 60.08, 55.59, 42.89, 42.02 ppm; **HRMS (ESI, m/z):** calculated for $[M+H]^+$: 321.1603, found: 321.1596.

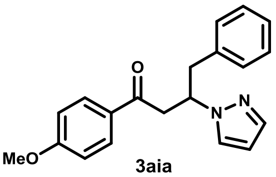

3aia

1-(4-methoxyphenyl)-5-phenyl-3-(1H-pyrazol-1-yl)pentan-1-one (3aja)

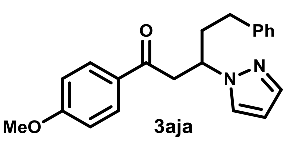

3aja

Following the General Procedure E, **3aja** was obtained in 45% yield as a white solid, mp 76-82 ℃; **^{1}H NMR (400 MHz, Chloroform-*d*):** δ 7.87 (d, *J* = 8.9 Hz, 2H), 7.55 (d, *J* = 1.8 Hz, 1H), 7.45 (d, *J* = 1.6 Hz, 1H), 7.30 – 7.23 (m, 2H), 7.18 (t, *J* = 7.3 Hz, 1H), 7.13 (d, *J* = 1.3 Hz, 1H), 7.11 (s, 1H), 6.88 (d, *J* = 8.9 Hz, 2H), 6.18 (t, *J* = 2.0 Hz, 1H), 4.85 (ddd, *J* = 10.5, 7.4, 5.5 Hz, 1H), 3.84 (s, 3H), 3.75 (dd, *J* = 17.2, 7.5 Hz, 1H), 3.28 (dd, *J* = 17.2, 5.4 Hz, 1H), 2.43 (tdd, *J* = 11.2, 8.9, 5.0 Hz, 3H), 2.16 (ddd, *J* = 11.1, 8.9, 5.1 Hz, 1H) ppm; **^{13}C NMR (100 MHz, Chloroform-*d*):** δ 196.00, 163.81, 141.00, 139.92, 130.56, 130.50, 129.81, 128.53, 126.14, 113.83, 104.48, 57.61, 55.58, 43.86, 36.79, 32.30 ppm; **HRMS (ESI, m/z):** calculated for $[M+H]^+$: 335.1760, found: 335.1755.

4-(4-methoxyphenyl)-4-oxo-2-(1H-pyrazol-1-yl)butyl acetate (3aka)

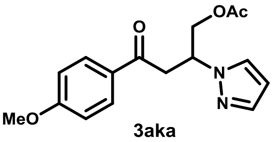

3aka

Following the General Procedure E, **3aka** was obtained in 22% yield as a colorless oil; **^{1}H NMR (400 MHz, Chloroform-*d*):** δ 7.91 (d, *J* = 8.8 Hz, 2H), 7.52 (d, *J* = 2.6 Hz, 1H), 7.51 (s, 1H), 6.91 (d, *J* = 8.9 Hz, 2H), 6.19 (t, *J* = 2.1 Hz, 1H), 5.18 (tt, *J* = 7.4, 5.3 Hz, 1H), 4.55 – 4.36 (m, 2H), 3.86 (s, 3H), 3.82 (dd, *J* = 17.4, 7.5 Hz, 1H), 3.38 (dd, *J* = 17.5, 5.5 Hz, 1H), 2.00 (s, 3H) ppm; **^{13}C NMR (100 MHz, Chloroform-*d*):** δ 194.77, 170.51, 163.98, 140.08, 130.56, 130.50, 129.50, 113.93, 105.10, 65.76, 56.41, 55.62, 39.73, 20.82 ppm; **HRMS (ESI, m/z):** calculated for $[M+H]^+$: 303.1345, found: 303.1340.

4-(4-(tert-butyl)phenyl)-4-oxo-2-(1H-pyrazol-1-yl)butyl acetate (3ala)

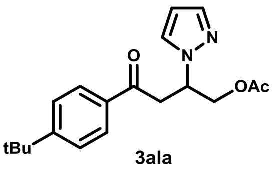

3ala

Following the General Procedure E, **3ala** was obtained in 20% yield as a white solid, mp 68-75 ℃; **^{1}H NMR (400 MHz, Chloroform-*d*):** δ 7.88 (d, *J* = 8.6 Hz, 2H), 7.54 (d, *J* = 2.0 Hz, 1H), 7.52 (d, *J* = 1.8 Hz, 1H), 7.46 (d, *J* = 8.5 Hz, 2H), 6.20 (t, *J* = 2.1 Hz, 1H), 5.20 (tt, *J* = 7.5, 5.2 Hz, 1H), 4.50 (dd, *J* = 11.2, 8.0 Hz, 1H), 4.43 (dd, *J* = 11.2, 5.2 Hz, 1H), 3.86 (dd, *J* = 17.8, 7.5 Hz, 1H), 3.41 (dd, *J* = 17.8, 5.4 Hz, 1H), 2.00 (s, 3H), 1.32 (s, 9H) ppm; **^{13}C NMR (100 MHz, Chloroform-*d*):** δ 195.80, 170.43, 157.48, 140.00, 133.70, 130.43, 128.10, 125.66,

105.02, 65.63, 56.14, 39.88, 35.18, 31.05, 20.74 ppm; **HRMS (ESI, m/z):** calculated for [M+H]+: 329.1865, found: 329.1862.

tert-butyl (3-(4-methoxyphenyl)-3-oxo-1-(1H-pyrazol-1-yl)propyl)carbamate (3ama)

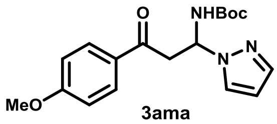

3ama

Following the General Procedure E, **3ama** was obtained in 65% yield as a white solid, mp 57-62 ℃; **^{1}H NMR (400 MHz, Chloroform-*d*):** δ 7.86 (d, *J* = 8.9 Hz, 2H), 7.69 (bs, 1H), 7.46 (d, *J* = 1.7 Hz, 1H), 6.88 (d, *J* = 8.9 Hz, 2H), 6.45 (s, 1H), 6.33 (d, *J* = 9.4 Hz, 1H), 6.17 (t, *J* = 2.1 Hz, 1H), 3.85 – 3.82 (m, 3H), 3.81 – 3.63 (m, 2H), 1.40 (s, 9H) ppm; **^{13}C NMR (100 MHz, Chloroform-*d*):** δ 194.47, 163.87, 154.58, 139.81, 130.48, 129.76, 129.41, 113.81, 105.09, 80.42, 63.71, 55.50, 41.90, 29.70, 28.24 ppm; ; HRMS (ESI, m/z): calculated for [M+H]+: 346.1767, found: 346.1762.

3-methoxy-1-(4-methoxyphenyl)-3-(1H-pyrazol-1-yl)propan-1-one (3ana)

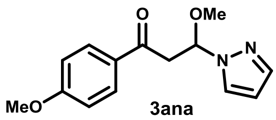

3ana

Following the General Procedure E, **3ana** was obtained in 75% yield as a colorless oil; **^{1}H NMR (400 MHz, Chloroform-d)**: δ 7.90 (d, *J* = 9.0 Hz, 2H), 7.63 (dd, *J* = 2.4, 0.6 Hz, 1H), 7.57 (d, *J* = 1.7 Hz, 1H), 6.88 (d, *J* = 9.0 Hz, 2H), 6.29 – 6.25 (m, 1H), 5.85 (dd, *J* = 6.8, 5.1 Hz, 1H), 3.81 (s, 3H), 3.76 (dd, *J* = 16.9, 6.8 Hz, 1H), 3.59 (dd, *J* = 16.9, 5.0 Hz, 1H), 3.22 (s, 3H).ppm; **^{13}C NMR (100 MHz, Chloroform-*d*)**: δ 194.33 , 163.90 , 140.49 , 130.64 , 129.63 , 129.13 , 113.88 , 105.88 , 88.37 , 56.31 , 55.55 , 43.75 ppm; **HRMS (ESI, m/z):** calculated for [M+H]+: 261.1234, found: 261.1232.

1,3-diphenyl-3-(1H-pyrazol-1-yl)propan-1-one (3aoa)

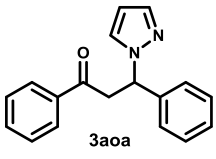

3aoa

Following the General Procedure E, **3aoa** was obtained in 67% yield as a colorless oil; **^{1}H NMR (400 MHz, Chloroform-*d*):** δ 7.98 (dd, *J* = 8.3, 1.1 Hz, 1H), 7.58 – 7.53 (m, 1H), 7.52 (d, *J* = 1.5 Hz, 1H), 7.50 (d, *J* = 2.3 Hz, 1H), 7.44 (t, *J* = 7.6 Hz, 2H), 7.34 (d, *J* = 4.4 Hz, 4H), 7.29 (dd, *J* = 8.1, 4.2 Hz, 1H), 6.24 (t, *J* = 2.1 Hz, 1H), 6.12 (dd, *J* = 8.4, 5.2 Hz, 1H), 4.50 (dd, *J* = 17.6, 8.4 Hz, 1H), 3.65 (dd, *J* = 17.6, 5.2 Hz, 1H) ppm; **^{13}C NMR (100 MHz, Chloroform-*d*):** δ 196.72, 140.81, 139.39, 136.59, 133.50, 129.89, 128.90, 128.72, 128.31, 128.11, 126.79, 105.69, 60.88, 44.27 ppm; **HRMS (ESI, m/z):** calculated for [M+H]$^{+}$: 277.1341, found: 277.1337.

1-(4-methoxyphenyl)-3-phenyl-3-(1H-pyrazol-1-yl)propan-1-one (3apa)

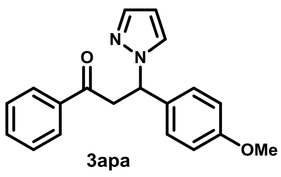

3apa

Following the General Procedure E, **3apa** was obtained in 44% yield; Following the one-pot General Procedure F, **3apa** was obtained in 30% total yield as a colorless oil; **^{1}H NMR (400 MHz, Chloroform-*d*):** δ 7.97 (d, *J* = 7.1 Hz, 2H), 7.60 – 7.51 (m, 1H), 7.50 (d, *J* = 1.8 Hz, 1H), 7.47 (d, *J* = 2.2 Hz, 1H), 7.44 (dd, *J* = 8.4, 7.1 Hz, 2H), 7.29 (d, *J* = 8.7 Hz, 2H), 6.85 (d, *J* = 8.7 Hz, 2H), 6.21 (t, *J* = 2.1 Hz, 1H), 6.05 (dd, *J* = 8.1, 5.6 Hz, 1H), 4.43 (dd, *J* = 17.5, 8.1 Hz, 1H), 3.77 (s, 3H), 3.65 (dd, *J* = 17.5, 5.6 Hz, 1H) ppm; **^{13}C NMR (100 MHz, Chloroform-*d*):** δ 196.74, 159.26, 139.28, 136.53, 133.40, 132.67, 129.57, 128.64, 128.22, 128.07, 114.12, 105.48, 60.34, 55.29, 44.23 ppm; **HRMS (ESI, m/z):** calculated for [M+H]$^{+}$: 307.1447, found: 307.1444.

1-(4-methoxyphenyl)-3-phenyl-3-(1H-pyrazol-1-yl)propan-1-one (3apa')

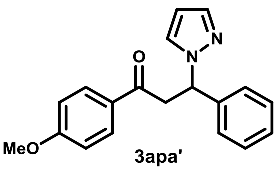

3apa'

^{1}H NMR (400 MHz, Chloroform-d): δ 7.96 (d, *J* = 9.1 Hz, 2H), 7.51 (d, *J* = 1.8 Hz, 1H), 7.49 (dd, *J* = 2.3, 0.6 Hz, 1H), 7.32 (d, *J* = 4.5 Hz, 4H), 6.91 (d, *J* = 9.0 Hz, 2H), 6.22 (dd, *J* = 1.9 Hz, 1H), 6.11 (dd, *J* = 8.4, 5.3 Hz, 1H), 4.42 (dd, *J* = 17.4, 8.4 Hz, 1H), 3.85 (s, 3H), 3.60 (dd, *J* = 17.4, 5.3 Hz, 1H) ppm; **^{13}C NMR (100 MHz, Chloroform-d)**: δ 195.16 , 163.81 , 140.93 , 139.37 , 130.62 , 129.86 , 129.71 , 128.85 , 128.01 , 126.78 , 113.84 , 105.59 , 61.00 , 55.58 , 43.88 ppm; **HRMS (ESI, m/z)**: calculated for [M+H]+: 307.1447, found: 307.1443.

1-(4-methoxyphenyl)-3-(1H-pyrazol-1-yl)-3-(4-(trifluoromethyl)phenyl)propan-1-one (3aqa)

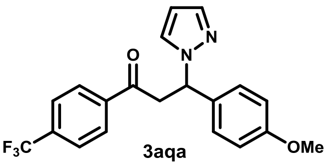

3aqa

Following the one-pot General Procedure F, **3aqa** was obtained in 56% total yield as a white solid, mp 63-65 ℃; **^{1}H NMR (400 MHz, Chloroform-*d*):** δ 8.07 (d, *J* = 8.1 Hz, 2H), 7.71 (d, *J* = 8.2 Hz, 2H), 7.49 (d, *J* = 1.8 Hz, 1H), 7.45 (d, *J* = 2.3 Hz, 1H), 7.28 (d, *J* = 8.8 Hz, 2H), 6.86 (d, *J* = 8.7 Hz, 2H), 6.22 (t, *J* = 2.1 Hz, 1H), 6.03 (dd, *J* = 8.4, 5.3 Hz, 1H), 4.49 (dd, *J* = 17.5, 8.4 Hz, 1H), 3.78 (s, 3H), 3.59 (dd, *J* = 17.5, 5.3 Hz, 1H) ppm; **^{13}C NMR (100 MHz, Chloroform-*d*):** δ 196.03, 159.39, 139.25, 134.57 (q, *J* = 32.6 Hz), 132.39, 129.60, 128.57, 127.98, 125.70 (q, *J* = 3.7 Hz), 114.19, 105.69, 60.28, 55.30, 44.52 ppm; **HRMS (ESI, m/z):** calculated for [M+Na]+: 397.1140, found: 397.1132.

1-(4-methoxyphenyl)-3-(1H-pyrazol-1-yl)-3-(4-(trifluoromethyl)phenyl)propan-1-one (3aqa')

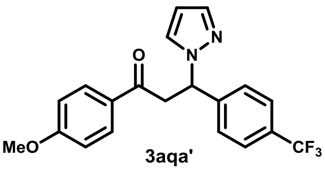

3aqa'

^{1}H NMR (400 MHz, Chloroform-d): δ 7.95 (d, *J* = 9.0 Hz, 2H), 7.57 (d, *J* = 8.2 Hz, 2H), 7.54 (d, *J* = 1.8 Hz, 1H), 7.52 (d, *J* = 2.3 Hz, 1H), 7.45 (d, *J* = 8.4 Hz, 2H), 6.90 (d, *J* = 9.0 Hz, 2H), 6.25 – 6.23 (m, 1H), 6.17 (dd, *J* = 8.0, 5.6 Hz, 1H), 4.40 (dd, *J* = 17.5, 8.0 Hz, 1H), 3.83 (s, 3H), 3.64 (dd, *J* = 17.5, 5.6 Hz, 1H) ppm; **^{13}C NMR (100 MHz, Chloroform-d)**: δ 194.65 , 163.99 , 144.95 , 139.79 , 130.63 , 130.02 , 129.45 , 127.23 , 125.81 (d, *J* = 3.7 Hz), 113.93 , 105.95 , 60.51 , 55.56 , 43.78 ppm; **HRMS (ESI, m/z):** calculated for [M+Na]+: 397.1140, found: 397.1137.

3-(4-methoxyphenyl)-1-(naphthalen-2-yl)-3-(1H-pyrazol-1-yl)propan-1-one (3ara)

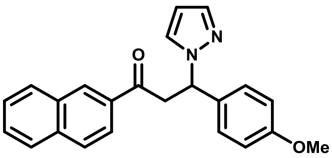

3ara

Following the General Procedure E, **3ara** was obtained in 63% yield; Following the one-pot General Procedure F, **3ara** was obtained in 43% total yield as a white solid, mp 80-92℃; **^{1}H NMR (400 MHz, Chloroform-*d*):** δ 8.53 (s, 1H), 8.01 (dd, *J* = 8.7, 1.8 Hz, 1H), 7.94 (d, *J* = 8.0 Hz, 1H), 7.87 (d, *J* = 4.3 Hz, 1H), 7.85 (d, *J* = 3.6 Hz, 1H), 7.62 – 7.57 (m, 1H), 7.56 – 7.54 (m, 1H), 7.53 (d, *J* = 1.9 Hz, 1H), 7.51 (d, *J* = 2.3 Hz, 1H), 7.34 (d, *J* = 8.7 Hz, 2H), 6.87 (d, *J* = 8.7 Hz, 2H), 6.23 (t, *J* = 2.1 Hz, 1H), 6.12 (dd, *J* = 8.0, 5.7 Hz, 1H), 4.58 (dd, *J* = 17.5, 8.0 Hz, 1H), 3.81 (dd, *J* = 17.4, 5.8 Hz, 1H), 3.78 (s, 3H) ppm; **^{13}C NMR (100 MHz, Chloroform-*d*):** δ 196.66, 159.29, 139.35, 135.71, 133.85, 132.69, 132.46,

130.19, 129.66, 128.64, 128.48, 128.10, 127.77, 126.84, 123.75, 114.14, 105.53, 60.49, 55.30, 44.26 ppm; **HRMS (ESI, m/z):** calculated for [M+H]+: 357.1603, found: 357.1595.

1-(4-methoxyphenyl)-3-(naphthalen-2-yl)-3-(1H-pyrazol-1-yl)propan-1-one (3ara')

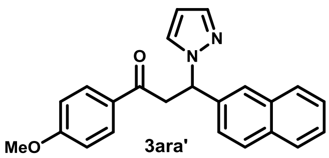

^{1}H NMR (400 MHz, Chloroform-*d*): δ 7.98 (d, *J* = 8.4 Hz, 2H), 7.84 – 7.73 (m, 4H), 7.54 (dd, *J* = 5.7, 1.5 Hz, 2H), 7.51 – 7.43 (m, 3H), 6.91 (d, *J* = 9.0 Hz, 2H), 6.28 (dd, *J* = 8.2, 5.4 Hz, 1H), 6.24 (t, *J* = 1.6 Hz, 1H), 4.51 (dd, *J* = 17.4, 8.2 Hz, 1H), 3.85 (s, 3H), 3.71 (dd, *J* = 17.4, 5.4 Hz, 1H) ppm; **^{13}C NMR (100 MHz, Chloroform-d):** δ 195.13 , 163.84 , 139.44 , 138.26 , 133.32 , 133.01 , 130.65 , 129.94 , 129.71 , 128.76 , 128.20 , 127.71 , 126.42 , 126.31 , 125.80 , 124.69 , 113.86 , 105.71 , 61.16 , 55.58 , 43.80 ppm; **HRMS (ESI, m/z):** calculated for [M+H]+: 357.1603, found: 357.1599.

3-(1H-pyrazol-1-yl)-3,4-dihydronaphthalen-1(2H)-one (3asa)

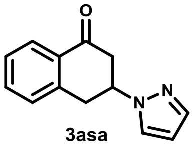

Following the General Procedure E, **3asa** was obtained in 30% yield as a colorless oil; **^{1}H NMR (400 MHz, Chloroform-*d*):** δ 7.75 (d, *J* = 7.7 Hz, 1H), 7.57 (td, *J* = 7.5, 1.3 Hz, 1H), 7.46 (d, *J* = 1.8 Hz, 1H), 7.43 – 7.39 (m, 2H), 7.36 (t, *J* = 7.5 Hz, 1H), 6.19 (t, *J* = 2.1 Hz, 1H), 4.66 (dd, *J* = 14.0, 4.5 Hz, 1H), 4.45 (dd, *J* = 14.0, 7.2 Hz, 1H), 3.28 (dd, *J* = 16.8, 7.9 Hz, 1H), 3.22 – 3.15 (m, 1H), 3.09 (dd, *J* = 16.7, 4.1 Hz, 1H) ppm; **^{13}C NMR (100 MHz, Chloroform-*d*):** δ 205.67, 153.78, 139.55, 136.28, 135.27, 129.76, 127.64, 126.73, 124.13, 105.88, 51.91, 48.75, 30.67 ppm; **HRMS (ESI, m/z):** calculated for [M+H]$^+$: 213.1028, found: 213.1026.

3-ethyl-3-(1H-pyrazol-1-yl)-3,4-dihydronaphthalen-1(2H)-one (3ata)

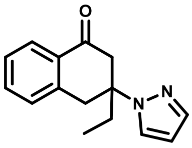

Following the General Procedure E, **3ata** was obtained in 40% yield as a white solid, mp 42-47 ℃ ; **^{1}H NMR (400 MHz, Chloroform-*d*):** δ 7.97 (d, *J* = 7.6 Hz, 1H), 7.48 (t, *J* = 7.1 Hz, 1H), 7.44 (d, *J* = 1.7 Hz, 1H), 7.41 (d, *J* = 2.2 Hz, 1H), 7.29 (d, *J* = 4.9 Hz, 2H), 6.13 (t, *J* = 2.1 Hz, 1H), 3.80 (d, *J* = 16.5 Hz, 1H), 3.50 (dd, *J* = 16.9, 2.2 Hz, 1H), 3.45 (d, *J* = 16.4 Hz, 1H), 3.01 (d, *J* = 16.8 Hz, 1H), 2.08 (dq, *J* = 14.8, 7.4 Hz, 1H), 1.96 (dq, *J* = 14.6, 7.4 Hz, 1H), 0.68 (t, *J* = 7.4 Hz, 3H) ppm; **^{13}C NMR (100 MHz, Chloroform-*d*):** δ 195.30, 140.15, 139.14, 134.27, 131.61, 129.18, 127.37 , 127.15, 126.86, 105.22, 64.79, 47.69, 40.21, 34.23, 7.41 ppm; **HRMS (ESI, m/z):** calculated for [M+H]$^+$: 241.1341, found: 241.1340.

7-(1H-pyrazol-1-yl)-6,7,8,9-tetrahydro-5H-benzo[7]annulen-5-one (3aua)

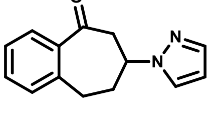

Following the General Procedure E, **3aua** was obtained in 68% yield as a yellow oil; **^{1}H NMR (400 MHz, Chloroform-*d*):** δ 8.03 (d, *J* = 7.9 Hz, 1H), 7.49 (dd, *J* = 4.7, 1.9 Hz, 2H), 7.45 (dd, *J* = 7.5, 1.4 Hz, 1H), 7.30 (t, *J* = 7.6 Hz, 1H), 7.21 (d, *J* = 7.7 Hz, 1H), 6.23 (t, *J* = 2.1 Hz, 1H), 4.77 (dd, *J* = 14.1, 4.9 Hz, 1H), 4.39 (dd, *J* = 14.1, 7.1 Hz, 1H), 3.07 (ddt, *J* = 13.5, 7.2, 4.9 Hz, 1H), 3.00 (dd, *J* = 11.9, 4.4 Hz, 1H), 2.97 – 2.90 (m, 1H), 2.11 (dq, *J* = 12.6, 4.2 Hz, 1H), 1.78 (qd, *J* = 13.2, 4.9 Hz, 1H) ppm; **^{13}C NMR (100 MHz, Chloroform-*d*):** δ 197.81, 144.15, 139.45, 133.77, 132.24, 130.45,

128.92, 127.51, 126.83, 105.58, 51.72, 48.85, 28.89, 27.07 ppm; **HRMS (ESI, m/z):** calculated for $[M+H]^+$: 227.1184, found: 227.1181.

(2-(1H-pyrazol-1-yl)cyclohexyl)(4-methoxyphenyl)methanone (3ava)

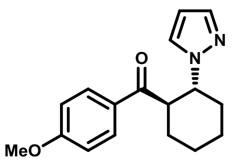
3ava

Following the General Procedure E, **3ava** was obtained in 52% yield as a white solid, mp 62-67 ℃; **^{1}H NMR (400 MHz, Chloroform-*d*):** δ 7.83 (d, *J* = 8.9 Hz, 2H), 7.36 (d, *J* = 1.8 Hz, 1H), 7.34 (d, *J* = 2.3 Hz, 1H), 6.84 (d, *J* = 8.9 Hz, 2H), 6.00 (t, *J* = 2.0 Hz, 1H), 4.57 (dt, *J* = 10.9, 5.4 Hz, 1H), 4.08 (td, *J* = 11.0, 3.5 Hz, 1H), 3.82 (s, 3H), 2.14 (dt, *J* = 17.0, 9.2 Hz, 2H), 2.06 – 1.88 (m, 2H), 1.88 – 1.81 (m, 1H), 1.71 (s, 1H), 1.54 – 1.46 (m, 2H) ppm; **^{13}C NMR (100 MHz, Chloroform-*d*):** δ 200.81, 163.64, 139.24, 130.70, 129.81, 129.30, 113.71, 104.28, 61.41, 55.53, 49.98, 32.75, 30.60, 25.17 ppm; **HRMS (ESI, m/z):** calculated for [M+H]+: 285.1603, found: 285.1598.

(3-(1H-pyrazol-1-yl)-1,2,3,4-tetrahydronaphthalen-2-yl)(4-methoxyphenyl)-methanone (3awa)

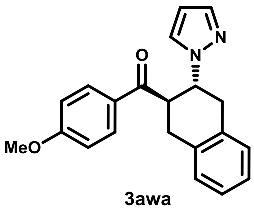
3awa

Following the General Procedure E, **3awa** was obtained in 38% yield as a white solid, mp 77-83 ℃; **^{1}H NMR (400 MHz, Chloroform-*d*):** δ 7.90 (d, *J* = 8.9 Hz, 2H), 7.43 (d, *J* = 2.2 Hz, 1H), 7.40 (d, *J* = 1.8 Hz, 1H), 7.18 (dt, *J* = 7.6, 4.2 Hz, 3H), 7.12 – 7.09 (m, 1H), 6.87 (d, *J* = 8.9 Hz, 2H), 6.07 (t, *J* = 2.1 Hz, 1H), 4.97 (td, *J* = 10.9, 5.6 Hz, 1H), 4.57 – 4.48 (m, 1H), 3.84 (s, 3H), 3.72 (dd, *J* = 16.7, 11.2 Hz, 1H), 3.29 (dd, *J* = 16.7, 5.6 Hz, 1H), 3.14 (d, *J* = 8.4 Hz, 2H) ppm; **^{13}C NMR (100 MHz, Chloroform-*d*):** δ 200.03, 163.85, 139.65, 134.20, 134.02, 130.89, 130.28, 129.27, 128.92, 128.42, 126.55, 126.49, 113.81, 104.70, 59.18, 55.57, 46.51, 35.76, 33.98 ppm; **HRMS (ESI, m/z):** calculated for [M+H]+: 333.1603, found: 333.1607.

benzyl 3-(4-methoxybenzoyl)-4-(1H-pyrazol-1-yl)cyclopentane-1-carboxylate (3axa)

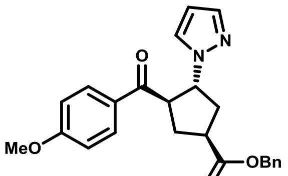
3axa

Following the General Procedure E, **3axa** was obtained in 45% yield as a white solid, mp 69-77 ℃; **^{1}H NMR (400 MHz, Chloroform-*d*):** δ 7.81 (d, *J* = 8.9 Hz, 2H), 7.51 (d, *J* = 1.8 Hz, 1H), 7.39 (dd, *J* = 2.3, 0.6 Hz, 1H), 7.40 – 7.29 (m, 5H), 6.86 (d, *J* = 8.9 Hz, 2H), 6.14 (t, *J* = 2.1 Hz, 1H), 5.22 – 5.11 (m, 1H), 5.17 (s, 2H), 4.36 (dt, *J* = 10.1, 7.4 Hz, 1H), 3.84 (s, 3H), 3.10 (dt, *J* = 17.0, 7.9 Hz, 1H), 2.76 – 2.57 (m, 3H), 2.17 (ddd, *J* = 13.3, 8.8, 7.1 Hz, 1H) ppm; **^{13}C NMR (100 MHz, Chloroform-*d*):** δ 198.48, 174.22, 163.94, 139.94, 135.90, 131.07, 129.66, 128.97, 128.71, 128.41, 128.33, 113.93, 105.18, 66.81, 63.49, 55.60, 51.24, 41.58, 36.26, 32.73 ppm. **HRMS (ESI, m/z):** calculated for [M+H]+: 405.1814, found: 405.1807.

1-(4-methoxyphenyl)-3-(1H-pyrazol-1-yl)tridecan-1-one (3aya)

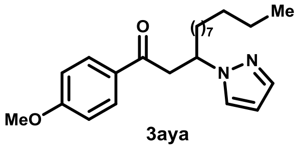
3aya

Following the one-pot General Procedure F, **3aya** was obtained in 39% yield as a white solid, mp 59-62 ℃ ; **^{1}H NMR (400 MHz, Chloroform-*d*):** δ 7.88 (d, *J* = 8.9 Hz, 2H), 7.49 (d, *J* = 1.7 Hz, 1H), 7.44 (d, *J* = 2.2 Hz, 1H), 6.87 (d, *J*

= 8.9 Hz, 2H), 6.13 (t, J = 2.1 Hz, 1H), 4.83 (ddt, J = 10.1, 7.4, 5.0 Hz, 1H), 3.82 (s, 3H), 3.72 (dd, J = 17.1, 7.4 Hz, 1H), 3.26 (dd, J = 17.1, 5.4 Hz, 1H), 2.04 (dtd, J = 14.3, 9.6, 4.8 Hz, 1H), 1.79 (ddt, J = 14.3, 9.5, 4.8 Hz, 1H), 1.31 – 1.12 (m, 16H), 0.86 (t, J = 6.8 Hz, 3H) ppm; **^{13}C NMR (100 MHz, Chloroform-*d*):** δ 196.11, 163.65, 139.51, 130.41, 130.06, 129.77, 113.69, 104.24, 58.28, 55.46, 43.71, 35.40, 31.90, 29.55, 29.52, 29.44, 29.31, 29.12, 26.15, 22.68, 14.14 ppm; **HRMS (ESI, m/z):** calculated for $[M+H]^+$: 371.2699, found: 371.2695.

2-(3-(4-methoxyphenyl)-3-(1H-pyrazol-1-yl)propanoyl)dibenzo[b,e]oxepin-11(6H)-one (3aza)

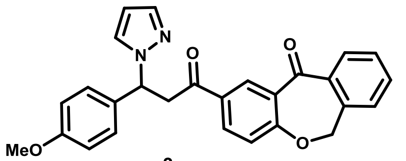

3aza

Following the one-pot General Procedure F, **3aza** was obtained in 54% total yield as a white solid, mp 146-149 ℃; **^{1}H NMR (400 MHz, Chloroform-*d*):** δ 8.87 (d, J = 2.3 Hz, 1H), 8.08 (dd, J = 8.7, 2.4 Hz, 1H), 7.85 (dd, J = 7.6, 1.4 Hz, 1H), 7.59 (td, J = 7.5, 1.4 Hz, 1H), 7.51 (dd, J = 7.6, 1.3 Hz, 1H), 7.48 (dd, J = 6.7, 2.1 Hz, 2H), 7.39 (d, J = 7.4 Hz, 1H), 7.30 (d, J = 8.7 Hz, 2H), 7.08 (d, J = 8.7 Hz, 1H), 6.87 (d, J = 8.7 Hz, 2H), 6.21 (t, J = 2.1 Hz, 1H), 6.06 (dd, J = 8.3, 5.3 Hz, 1H), 5.24 (s, 2H), 4.48 (dd, J = 17.6, 8.4 Hz, 1H), 3.78 (s, 3H), 3.67 (dd, J = 17.6, 5.4 Hz, 1H) ppm; **^{13}C NMR (100 MHz, Chloroform-*d*):** δ 194.89, 190.54, 164.74, 159.29, 140.46, 139.20, 134.57, 134.43, 133.57, 133.10, 132.58, 130.70, 129.68, 129.45, 129.34, 128.10, 128.07, 124.44, 121.49, 114.15, 105.50, 73.49, 60.33, 55.31, 43.98 ppm; **HRMS (ESI, m/z):** calculated for [M+H]+: 439.1658, found: 439.1659.

2-(3-(4-methoxyphenyl)-3-oxo-1-(1H-pyrazol-1-yl)propyl)dibenzo[b,e]oxepin-11(6H)-one (3aza')

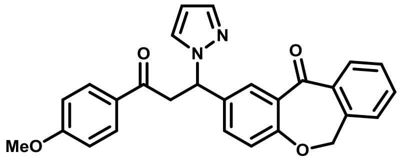

3aza'

Following the one-pot General Procedure F, **3aza'** was obtained in 15% total yield as a white solid, mp 152-159 ℃; **^{1}H NMR (400 MHz, Chloroform-*d*):** δ 8.25 (d, J = 2.4 Hz, 1H), 7.96 (d, J = 8.9 Hz, 2H), 7.87 (dd, J = 7.6, 1.3 Hz, 1H), 7.56 (td, J = 7.4, 1.4 Hz, 1H), 7.53 (d, J = 2.3 Hz, 1H), 7.51 (d, J = 1.9 Hz, 1H), 7.50 – 7.44 (m, 2H), 7.35 (d, J = 7.4 Hz, 1H), 7.02 (d, J = 8.6 Hz, 1H), 6.91 (d, J = 8.9 Hz, 2H), 6.22 (t, J = 2.1 Hz, 1H), 6.13 (dd, J = 8.2, 5.4 Hz, 1H), 5.17 (s, 2H), 4.40 (dd, J = 17.4, 8.3 Hz, 1H), 3.86 (s, 3H), 3.65 (dd, J = 17.4, 5.5 Hz, 1H) ppm; **^{13}C NMR (100 MHz, Chloroform-*d*):** δ 194.85, 190.73, 163.77, 160.92, 140.38, 139.62, 135.39, 134.35, 134.06, 132.85, 130.56, 130.16, 129.69, 129.52, 129.33, 127.84, 125.04, 121.45, 113.78, 105.56, 73.59, 60.18, 55.52, 43.44 ppm; **HRMS (ESI, m/z):** calculated for [M+H]+: 439.1658, found: 439.1656.

3-(4-bromo-1H-pyrazol-1-yl)-1-(4-bromophenyl)-3-methylbutan-1-one (3bb)

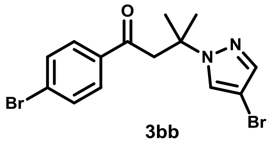

3bb

Following the General Procedure E, **3bb** was obtained in 77% yield as a white solid, mp 65-69 ℃; **^{1}H NMR (400 MHz, Chloroform-*d*):** δ 7.67 (d, J = 8.6 Hz, 2H), 7.53 (d, J = 8.6 Hz, 2H), 7.52 (s, 1H), 7.40 (s, 1H), 3.50 (s, 2H), 1.74 (s, 6H) ppm; **^{13}C NMR (100 MHz, Chloroform-*d*):** δ 196.36, 139.58, 136.03, 131.89, 129.61,

128.54, 127.02, 92.38, 60.52, 48.55, 27.87 ppm; **HRMS (ESI, m/z):** calculated for $[M+H]^+$: 384.9551, found: 384.9546.

1-(4-bromophenyl)-3-(4-chloro-1H-pyrazol-1-yl)-3-methylbutan-1-one (3bc)

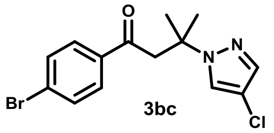

3bc

Following the General Procedure E, **3bc** was obtained in 70% yield as a white solid, mp 61-65 °C; **^{1}H NMR (400 MHz, Chloroform-*d*):** δ 7.68 (d, *J* = 8.7 Hz, 2H), 7.53 (d, *J* = 8.6 Hz, 2H), 7.49 (s, 1H), 7.37 (s, 1H), 3.50 (s, 2H), 1.73 (s, 6H) ppm; **^{13}C NMR (100 MHz, Chloroform-*d*):** δ 196.36, 137.44, 136.03, 131.88, 129.62, 128.53, 124.91, 109.22, 60.47, 48.48, 27.86 ppm; **HRMS (ESI, m/z):** calculated for [M+H]+: 341.0056, found: 341.0050.

1-(4-bromophenyl)-3-methyl-3-(4-methyl-1H-pyrazol-1-yl)butan-1-one (3bd)

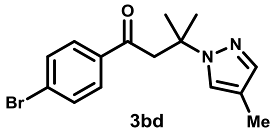

3bd

Following the General Procedure E, **3bd** was obtained in 55% yield as a colorless oil; **^{1}H NMR (400 MHz, Chloroform-*d*):** δ 7.65 (d, *J* = 8.6 Hz, 2H), 7.50 (d, *J* = 8.7 Hz, 2H), 7.26 (s, 1H), 7.23 (s, 1H), 3.49 (s, 2H), 1.98 (s, 3H), 1.73 (s, 6H) ppm; **^{13}C NMR (100 MHz, Chloroform-*d*):** δ 197.02, 139.39, 136.12, 131.61, 129.63, 128.18, 125.30, 115.18, 59.28, 49.09, 27.94, 8.89 ppm; **HRMS (ESI, m/z):** calculated for [M+H]+: 260.0603, found: 260.0595.

1-(4-bromophenyl)-3-methyl-3-(1H-pyrazol-1-yl)butan-1-one (3be)

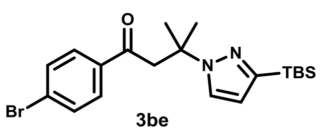

3be

Following the General Procedure E, **3be** was obtained in 53% yield as a white solid, mp 52-59 °C; **^{1}H NMR (400 MHz, Chloroform-*d*):** δ 7.59 (d, *J* = 8.5 Hz, 2H), 7.46 (d, *J* = 8.5 Hz, 2H), 7.44 (d, *J* = 2.2 Hz, 1H), 6.20 (d, *J* = 2.2 Hz, 1H), 3.50 (s, 2H), 1.78 (s, 6H), 0.19 (s, 9H) ppm; **^{13}C NMR (100 MHz, Chloroform-*d*):** δ 197.80, 152.35, 136.53, 131.67, 129.89, 128.14, 126.15, 111.08, 59.97, 49.37, 28.60, -0.82 ppm; **HRMS (ESI, m/z):** calculated for [M+H]+: 421.1311, found: 421.1302.

1-(4-methoxyphenyl)-3-(1H-pyrazol-1-yl)tridecan-1-one (3aye)

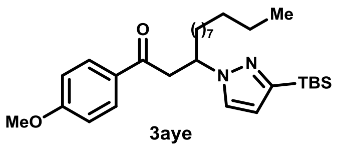

3aye

Following the General Procedure E, **3aye** was obtained in 33% yield as a white solid, mp 68-72 °C; **^{1}H NMR (400 MHz, Chloroform-*d*):** δ 7.87 (d, *J* = 8.9 Hz, 2H), 7.41 (d, *J* = 2.1 Hz, 1H), 6.87 (d, *J* = 8.8 Hz, 2H), 6.24 (d, *J* = 2.2 Hz, 1H), 4.92 – 4.79 (m, 1H), 3.85 (s, 3H), 3.77 (dd, *J* = 16.5, 7.3 Hz, 1H), 3.20 (dd, *J* = 16.5, 5.6 Hz, 1H), 2.12 – 1.97 (m, 1H), 1.90 – 1.75 (m, 1H), 1.32 – 1.15 (m, 16H), 0.87 (t, *J* = 6.9 Hz, 3H), 0.23 (s, 9H) ppm; **^{13}C NMR (100 MHz, Chloroform-*d*):** δ 196.98, 163.67, 152.78, 130.64, 130.18, 129.32, 113.75, 110.83, 58.69, 55.61, 43.94, 35.51, 32.06, 29.72, 29.66, 29.54, 29.48, 29.25, 26.24, 22.85, 14.31, -0.74 ppm; **HRMS (ESI, m/z):** calculated for [M+H]+: 485.3563, found: 485.3580.

1-(4-methoxyphenyl)-3-methyl-3-(1H-1,2,4-triazol-1-yl)butan-1-one (3af)

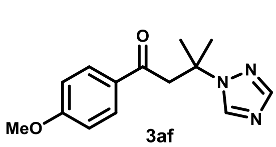

3af

Following the General Procedure E, **3af** was obtained in 44% yield as a colorless oil; **^{1}H NMR (400 MHz, Chloroform-*d*):** δ 8.17 (s, 1H), 7.89 (s, 1H), 7.80 (d, *J* = 8.9 Hz, 2H), 6.86 (d, *J* = 9.0 Hz, 2H), 3.84 (s, 3H), 3.51 (s, 2H), 1.80 (s, 6H) ppm; **^{13}C NMR (100 MHz, Chloroform-*d*):** δ 195.20, 163.77, 151.59, 140.94, 130.36, 130.23,

113.79, 59.56, 55.57, 47.80, 27.70 ppm; **HRMS (ESI, m/z):** calculated for [M+H]+: 260.1399, found: 260.1392.

1-(4-methoxyphenyl)-3-methyl-3-(2H-1,2,3-triazol-2-yl)butan-1-one (3ag)

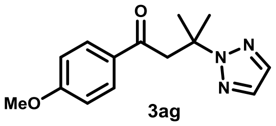
3ag

Following the General Procedure E, **3ag** was obtained in 21% yield as a colorless oil; **^{1}H NMR (400 MHz, Chloroform-*d*):** δ 7.84 (d, *J* = 8.9 Hz, 2H), 7.55 (s, 2H), 6.87 (d, *J* = 8.9 Hz, 2H), 3.85 (s, 3H), 3.62 (s, 2H), 1.85 (s, 6H) ppm; **^{13}C NMR (100 MHz, Chloroform-*d*):** δ 195.41, 163.59, 133.51, 130.64, 130.47, 113.71, 64.09, 55.57, 48.31, 27.84 ppm; **HRMS (ESI, m/z):** calculated for $[M+H]^+$: 260.1399, found: 260.1396.

1-(4-methoxyphenyl)-3-methyl-3-(1H-1,2,3-triazol-1-yl)butan-1-one (3ag')

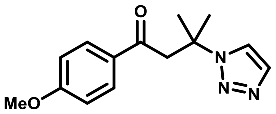
3ag'

Following the General Procedure E, **3ag'** was obtained in 30% yield as a colorless oil; **^{1}H NMR (400 MHz, Chloroform-*d*):** δ 7.80 (d, *J* = 8.9 Hz, 2H), 7.66 (d, *J* = 1.0 Hz, 1H), 7.61 (d, *J* = 1.0 Hz, 1H), 6.86 (d, *J* = 8.9 Hz, 2H), 3.84 (s, 3H), 3.65 (s, 2H), 1.86 (s, 6H) ppm; **^{13}C NMR (100 MHz, Chloroform-*d*):** δ 195.17, 163.80, 132.99, 130.39, 130.23, 121.52, 113.83, 60.32, 55.59, 48.64, 28.38 ppm; **HRMS (ESI, m/z):** calculated for [M+H]+: 260.1399, found: 260.1396.

3-(2H-benzo[d][1,2,3]triazol-2-yl)-1-(4-methoxyphenyl)-3-methylbutan-1-one (3ah)

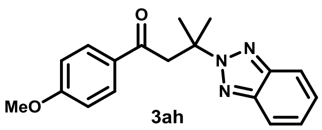
3ah

Following the General Procedure E, **3ah** was obtained in 24% yield as a white solid, mp 84-89 ℃; **^{1}H NMR (400 MHz, Chloroform-*d*):** δ 7.83 (d, *J* = 9.2 Hz, 4H), 7.33 (dd, *J* = 6.6, 3.1 Hz, 2H), 6.82 (d, *J* = 8.9 Hz, 2H), 3.82 (s, 3H), 3.81 (s, 2H), 2.00 (s, 6H) ppm; **^{13}C NMR (100 MHz, Chloroform-*d*):** δ 195.11, 163.58, 143.92, 130.51, 130.38, 126.04, 118.22, 113.65, 66.00, 55.55, 48.79, 28.19 ppm; **HRMS (ESI, m/z):** calculated for [M+H]+: 310.1556, found: 310.1558.

3-(1H-benzo[d][1,2,3]triazol-1-yl)-1-(4-methoxyphenyl)-3-methylbutan-1-one (3ah')

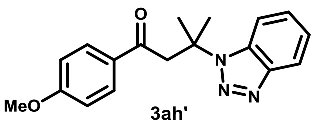
3ah'

Following the General Procedure E, **3ah'** was obtained in 40% yield as a white solid, mp 87-91 ℃; **^{1}H NMR (400 MHz, Chloroform-*d*):** δ 8.03 (d, *J* = 8.3 Hz, 1H), 7.82 (d, *J* = 8.9 Hz, 2H), 7.77 (d, *J* = 8.5 Hz, 1H), 7.45 – 7.39 (m, 1H), 7.34 – 7.29 (m, 1H), 6.84 (d, *J* = 8.9 Hz, 2H), 3.83 (s, 3H), 3.81 (s, 2H), 2.07 (s, 6H) ppm; **^{13}C NMR (100 MHz, Chloroform-*d*):** δ 195.27, 163.70, 146.95, 132.21, 130.43, 126.93, 123.51, 120.41, 113.79, 112.17, 62.43, 55.58, 48.46, 28.18 ppm; **HRMS (ESI, m/z):** calculated for [M+H]+: 310.1556, found: 310.1554.

1,1,1-trifluoro-N-(4-(4-methoxyphenyl)-2-methyl-4-oxobutan-2-yl)methanesulfonamide (3ai)

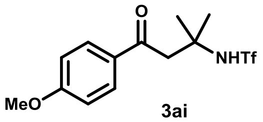
3ai

Following the General Procedure E, **3ai** was obtained in 37% yield as a colorless oil; **^{1}H NMR (400 MHz, Chloroform-d)**: δ 7.92 (d, *J* = 8.9 Hz, 2H), 7.12 (s, 1H), 6.96 (d, *J* = 8.9 Hz, 2H), 3.89 (s, 3H), 3.19 (s, 2H), 1.55 (s, 6H) ppm; **^{19}F NMR (376 MHz, Chloroform-d)**: δ -78.00 ppm; **^{13}C NMR (100 MHz, Chloroform-*d*):** δ 198.13 , 164.39 , 130.61 , 129.66 ,

119.28 (q, *J* = 320.8 Hz), 114.04 , 58.26 , 55.60 , 48.38 , 28.09 ppm; **HRMS (ESI, m/z):** calculated for [M+H]+: 340.0825, found: 340.0828.

(2-(4-(1H-pyrazol-1-yl)phenyl)cyclopropyl)methyl acetate (5aka)

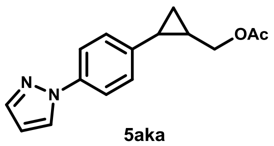

5aka

^{1}H NMR (400 MHz, Chloroform-*d*): δ 7.86 (dd, J = 2.4, 0.5 Hz, 1H), 7.69 (d, J = 1.5 Hz, 1H), 7.57 (d, J = 8.6 Hz, 2H), 7.13 (d, J = 8.5 Hz, 2H), 6.45 – 6.40 (m, 1H), 4.11 – 3.95 (m, 2H), 2.07 (s, 3H), 1.91 (dt, J = 8.7, 5.3 Hz, 1H), 1.53 – 1.43 (m, 1H), 1.05 – 0.95 (m, 2H) ppm; **^{13}C NMR (100 MHz, Chloroform-*d*):** δ 171.30 , 140.96 , 140.47 , 138.35 , 126.99 , 126.74 , 119.36 , 107.50 , 67.92 , 21.60 , 21.48 , 21.13 , 14.05 ppm; **HRMS (ESI, m/z):** calculated for $[M+H]^+$: 257.1290, found: 257.1287.

ethyl 2-(4-(1H-pyrazol-1-yl)phenyl)cyclopropane-1-carboxylate (5bba)

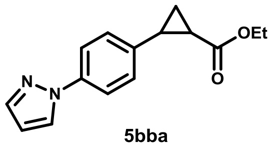

5bba

^{1}H NMR (400 MHz, Chloroform-*d*): δ 7.89 (d, *J* = 2.4 Hz, 1H), 7.71 (d, *J* = 1.6 Hz, 1H), 7.60 (d, *J* = 8.6 Hz, 2H), 7.18 (d, *J* = 8.6 Hz, 2H), 6.47 – 6.44 (m, 1H), 4.31 – 4.03 (m, 2H), 2.55 (ddd, *J* = 9.4, 6.5, 4.2 Hz, 1H), 1.92 (ddd, *J* = 8.5, 5.3, 4.2 Hz, 1H), 1.65 – 1.61 (m, 1H), 1.37 – 1.31 (m, 1H), 1.29 (t, *J* = 7.1 Hz, 3H) ppm; **^{13}C NMR (100 MHz, Chloroform-*d*):** δ 173.33 , 141.09 , 138.53 , 127.29 , 126.75 , 119.35 , 107.64 , 60.90 , 25.72 , 24.28 , 17.14 , 14.35 ppm; **HRMS (ESI, m/z):** calculated for $[M+H]^+$: 257.1290, found: 257.1284.

1-(1a,6b-dihydro-1H-cyclopropa[b]benzofuran-5-yl)-1H-pyrazole (6baa)

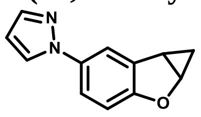

6baa

^{1}H NMR (400 MHz, Chloroform-*d*): δ 7.82 – 7.76 (m, 1H), 7.68 (d, *J* = 1.5 Hz, 1H), 7.66 (d, *J* = 2.4 Hz, 1H), 7.34 (dd, *J* = 8.6, 2.4 Hz, 1H), 6.85 (d, *J* = 8.6 Hz, 1H), 6.54 – 6.27 (m, 1H), 4.87 (td, *J* = 5.4, 1.9 Hz, 1H), 2.67 (ddd, *J* = 9.1, 5.2, 4.1 Hz, 1H), 1.13 – 0.88 (m, 1H), 0.50 – 0.24 (m, 1H) ppm; **^{13}C NMR (100 MHz, Chloroform-*d*):** δ 158.10 , 140.59 , 132.59 , 127.12 , 118.87 , 116.49 , 110.48 , 107.13 , 62.48 , 19.92 , 10.33 ppm; **HRMS (ESI, m/z):** calculated for $[M+H]^+$: 199.0871, found: 199.0867.

^{1}H, and ^{13}C NMR Spectra of Structurally Novel Compounds

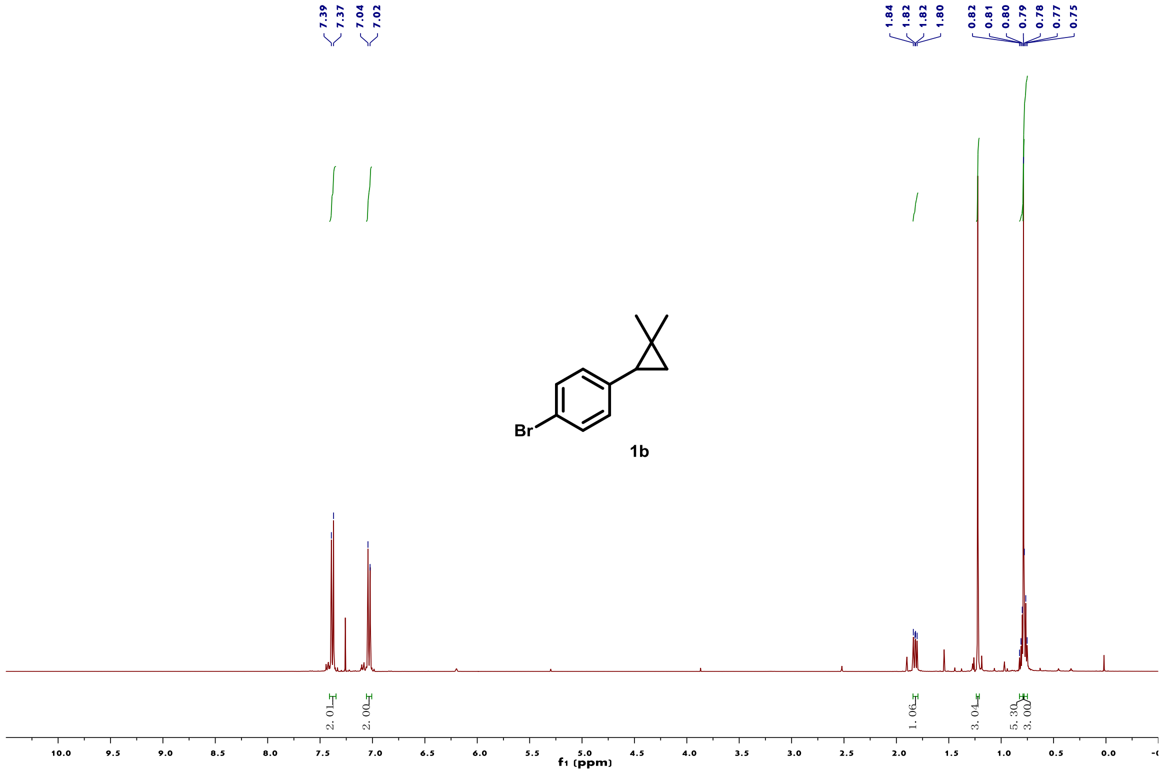

Supplementary Figure 32. ^{1}H NMR (400 MHz, $CDCl_3$) spectrum for 1b

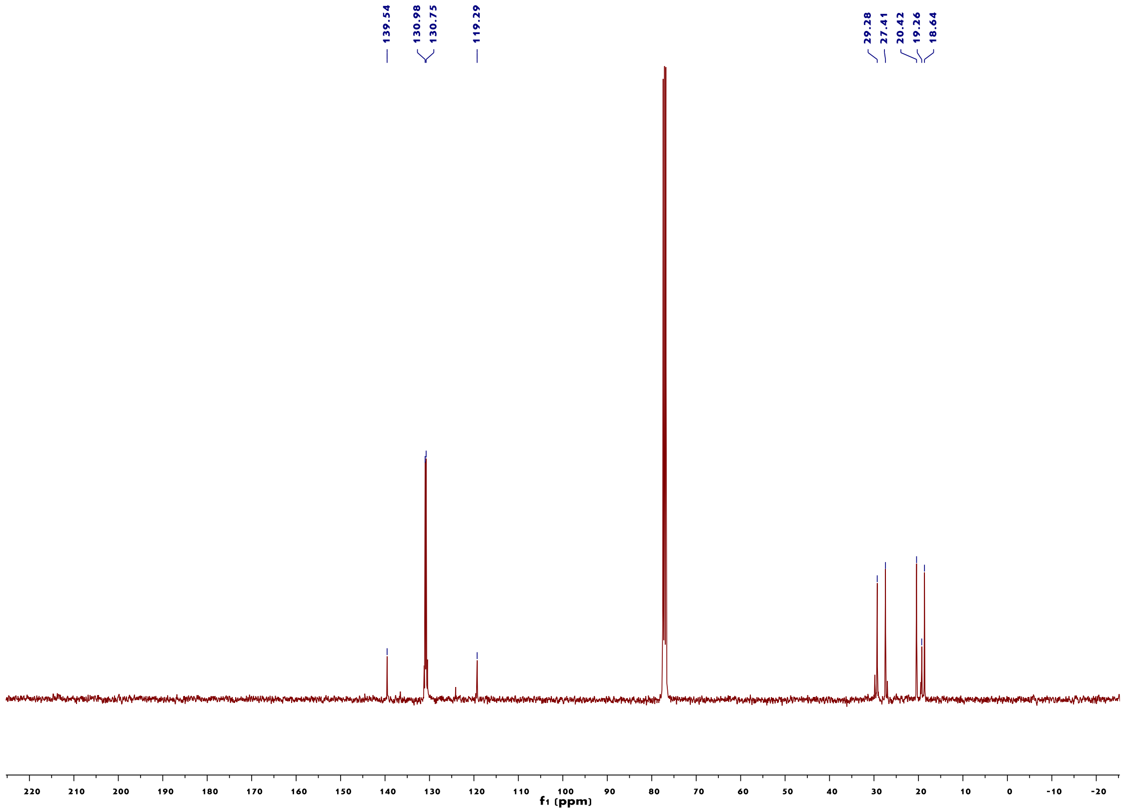

Supplementary Figure 33. ^{13}C NMR (100 MHz, $CDCl_3$) spectrum for 1b

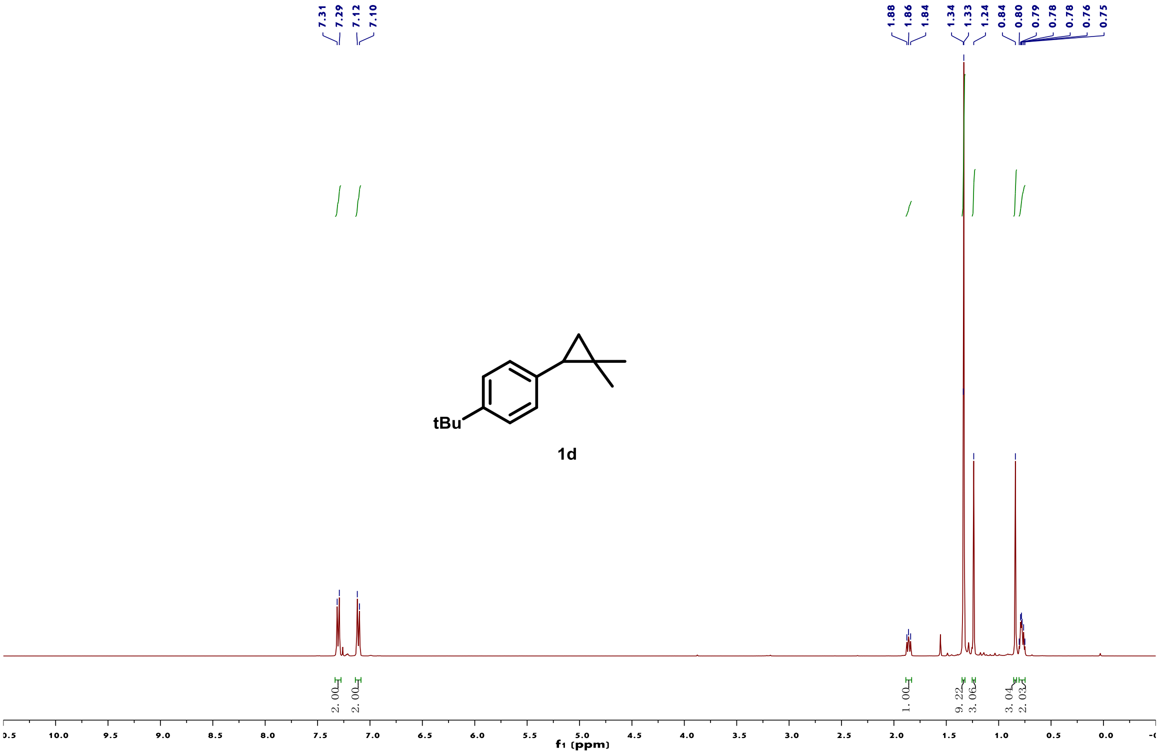

Supplementary Figure 34. ^{1}H NMR (400 MHz, $CDCl_3$) spectrum for 1d

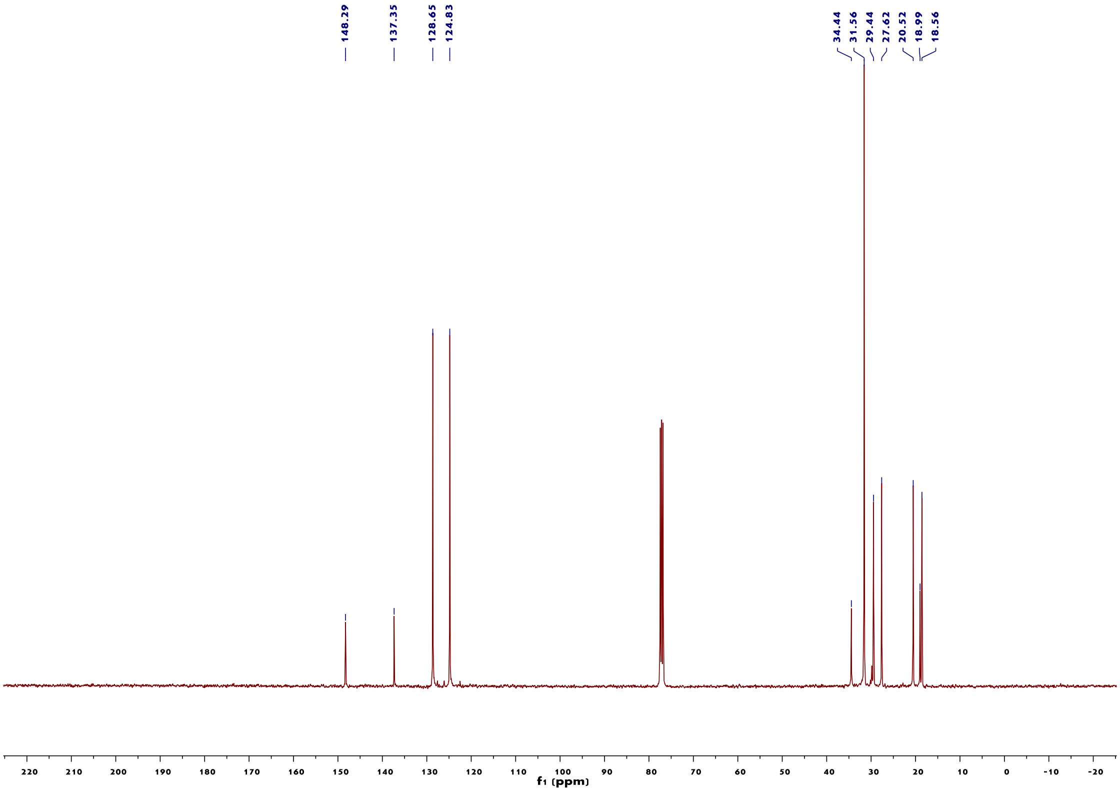

Supplementary Figure 35. ^{13}C NMR (100 MHz, $CDCl_3$) spectrum for 1d

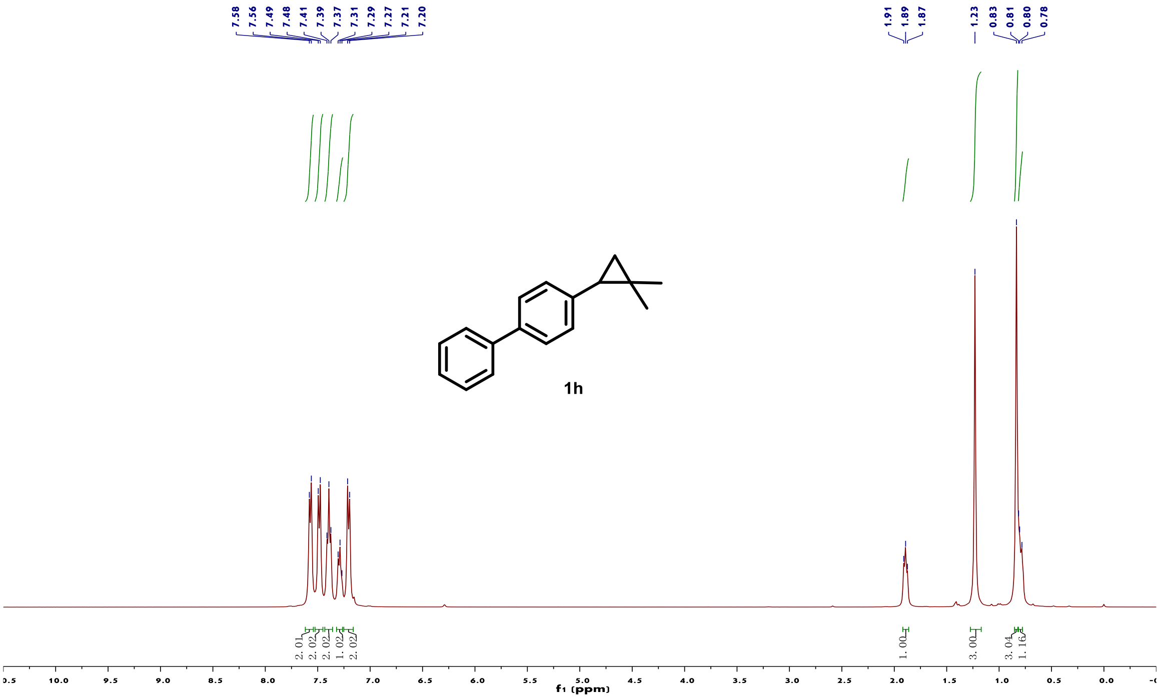

Supplementary Figure 36. ^{1}H NMR (400 MHz, $CDCl_3$) spectrum for 1h

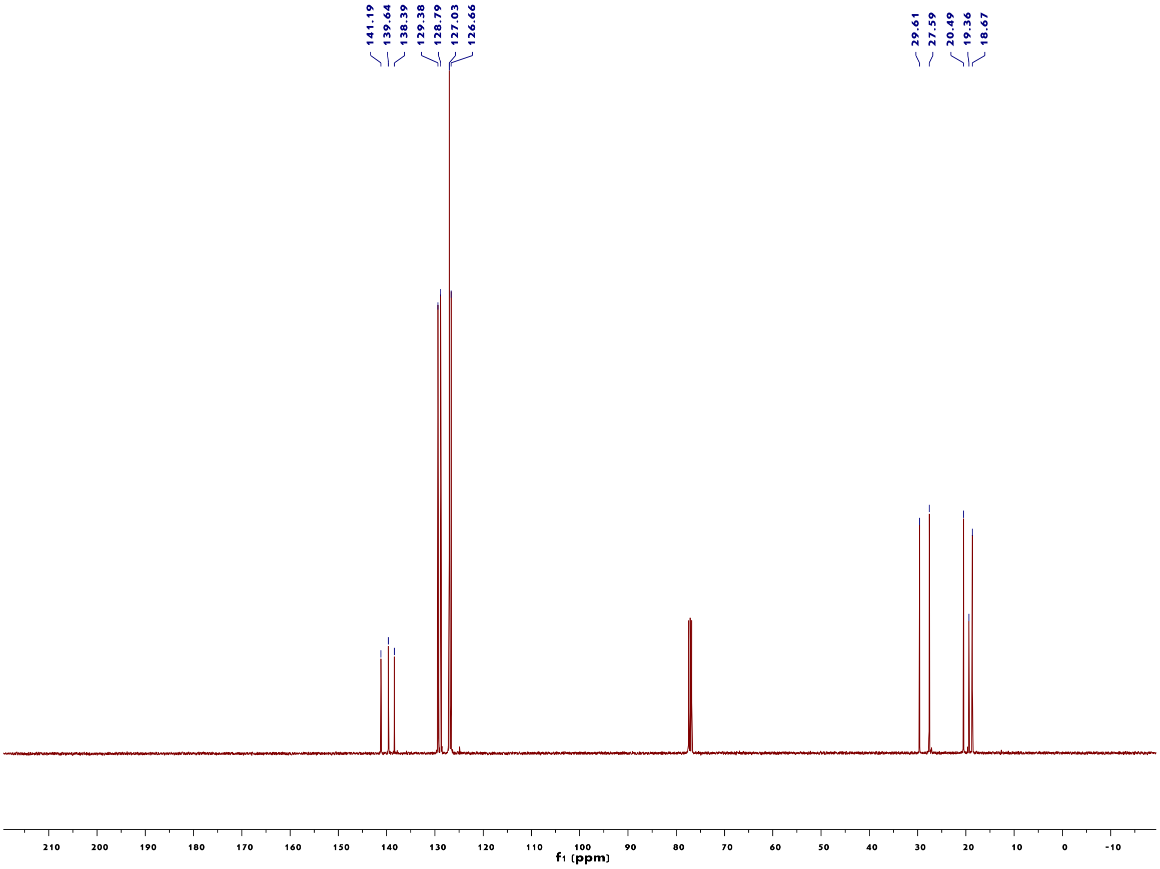

Supplementary Figure 37. ^{13}C NMR (100 MHz, $CDCl_3$) spectrum for 1h

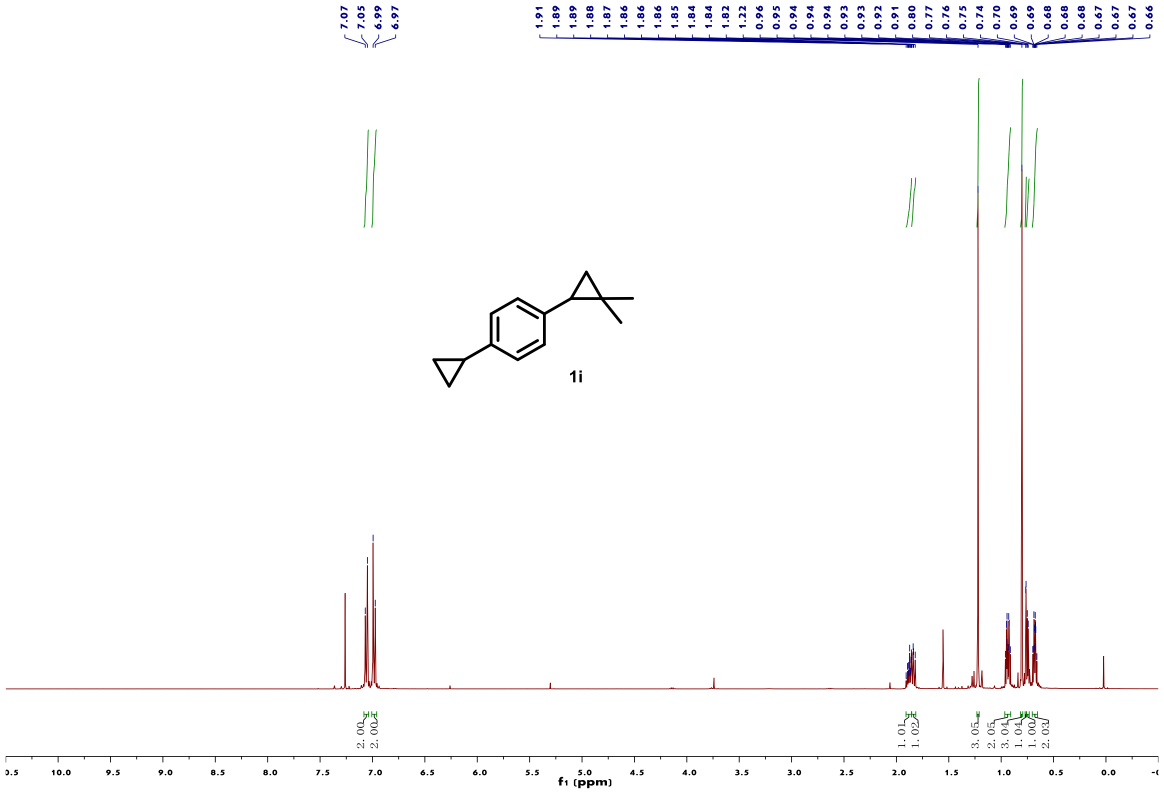

Supplementary Figure 38. ^{1}H NMR (400 MHz, $CDCl_3$) spectrum for 1i

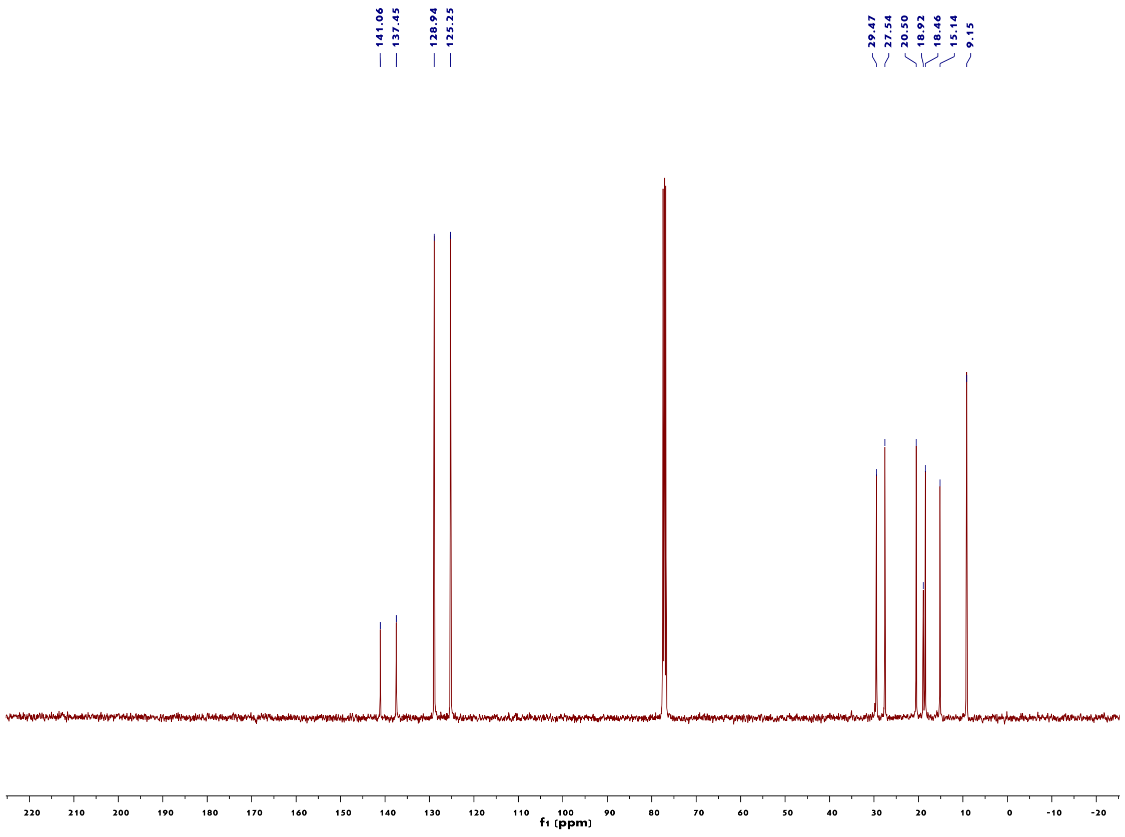

Supplementary Figure 39. ^{13}C NMR (100 MHz, $CDCl_3$) spectrum for 1i

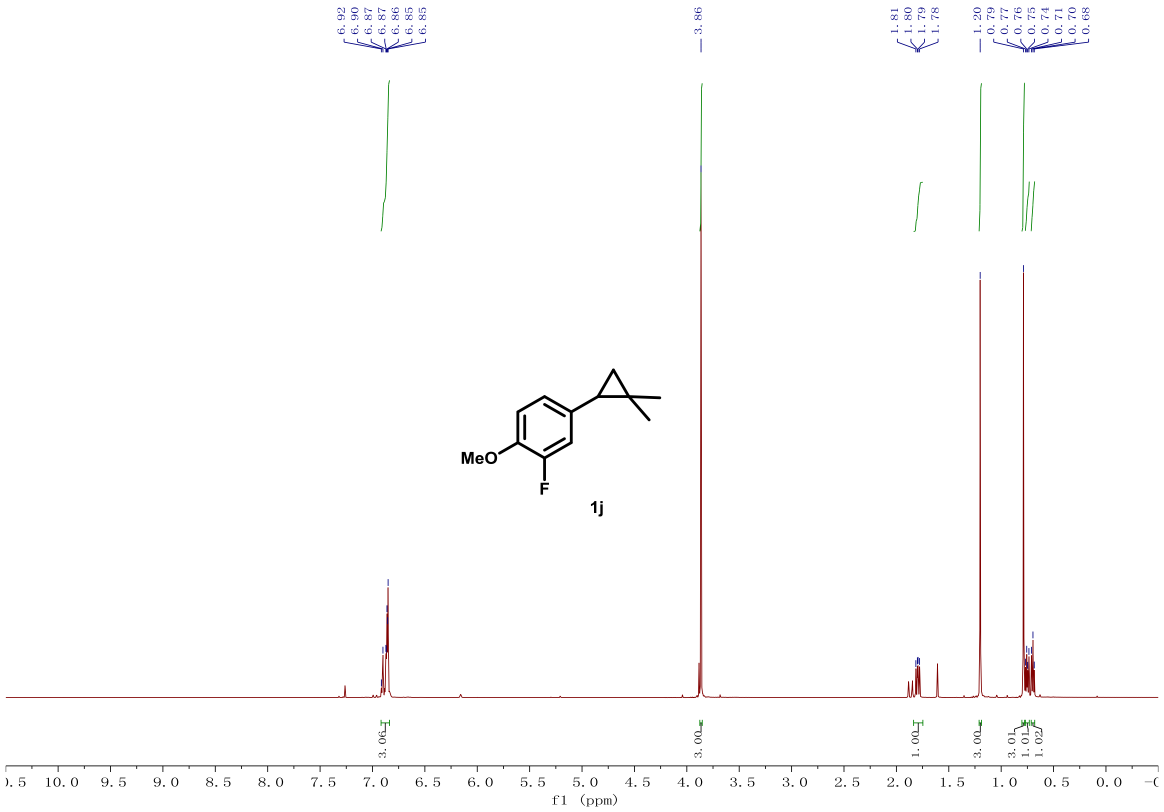

Supplementary Figure 40. ^{1}H NMR (400 MHz, $CDCl_3$) spectrum for 1j

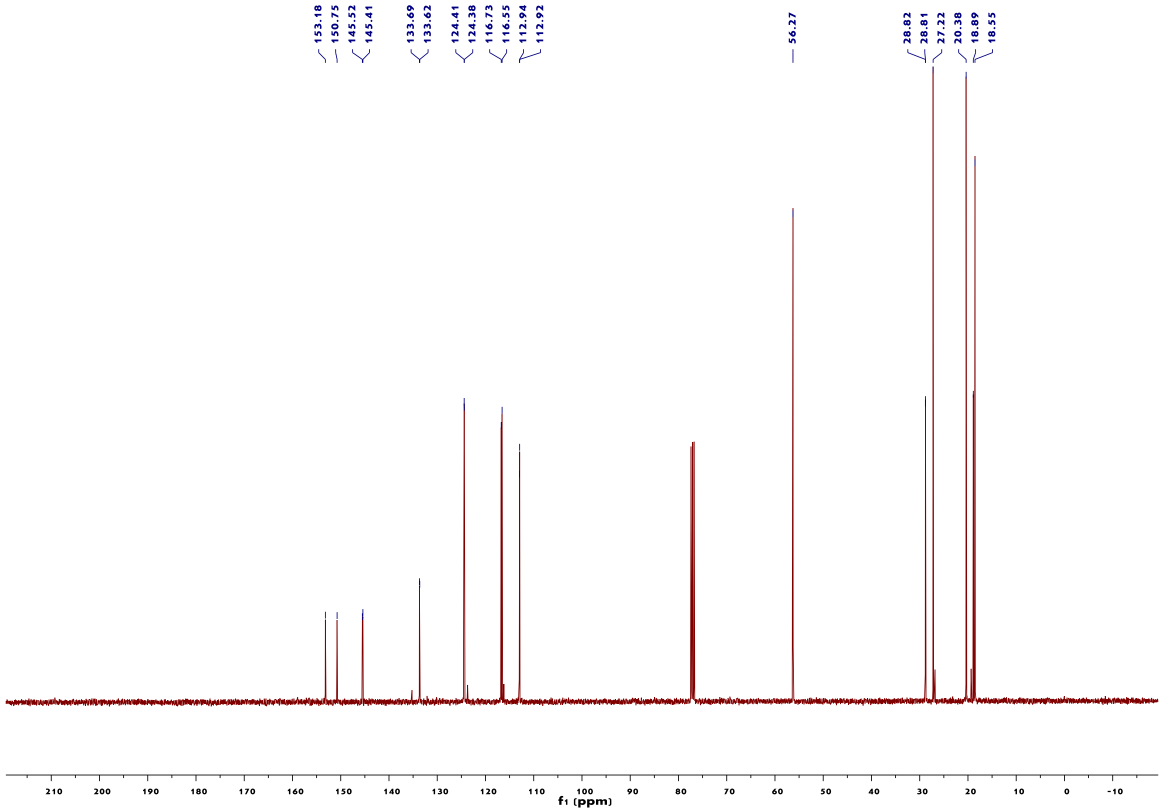

Supplementary Figure 41. ^{13}C NMR (100 MHz, $CDCl_3$) spectrum for 1j

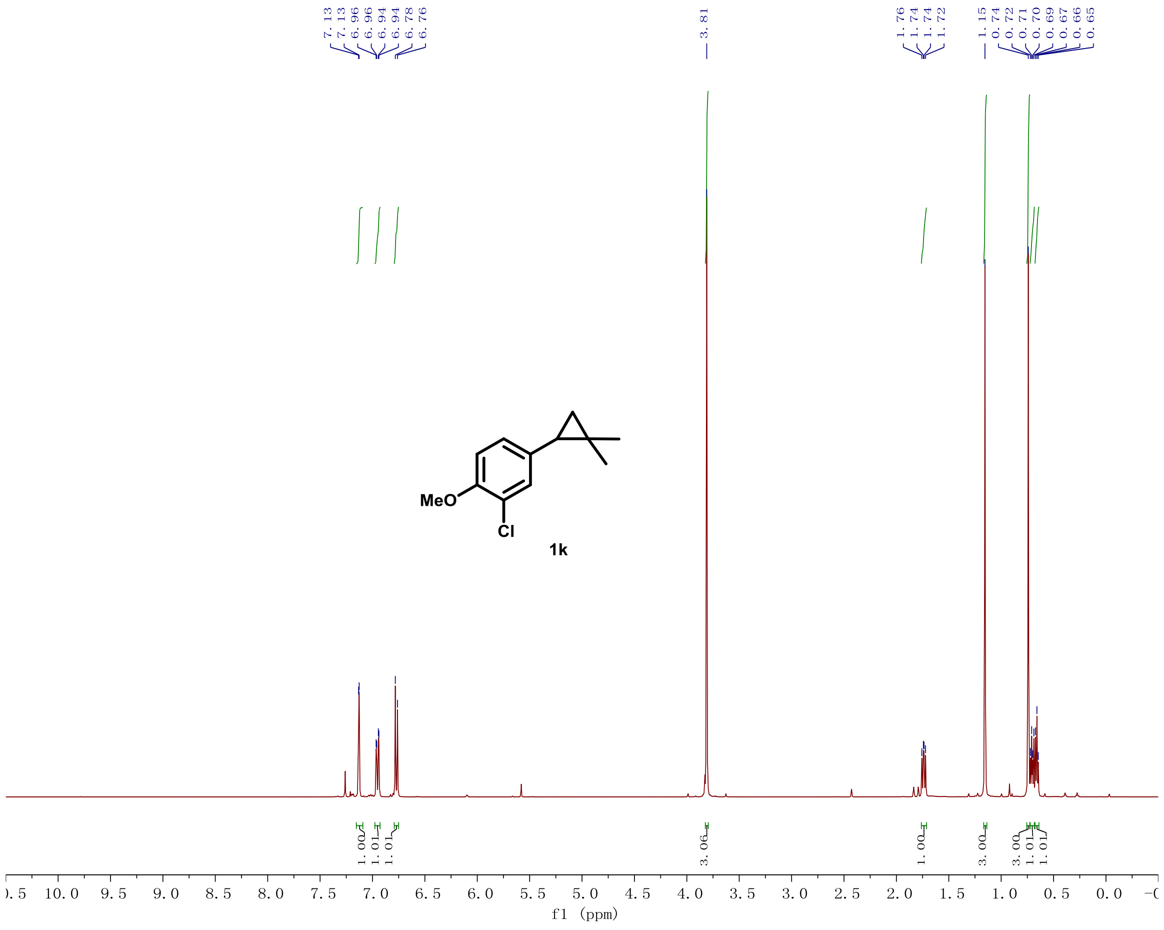

Supplementary Figure 42. ^{1}H NMR (400 MHz, $CDCl_3$) spectrum for 1k

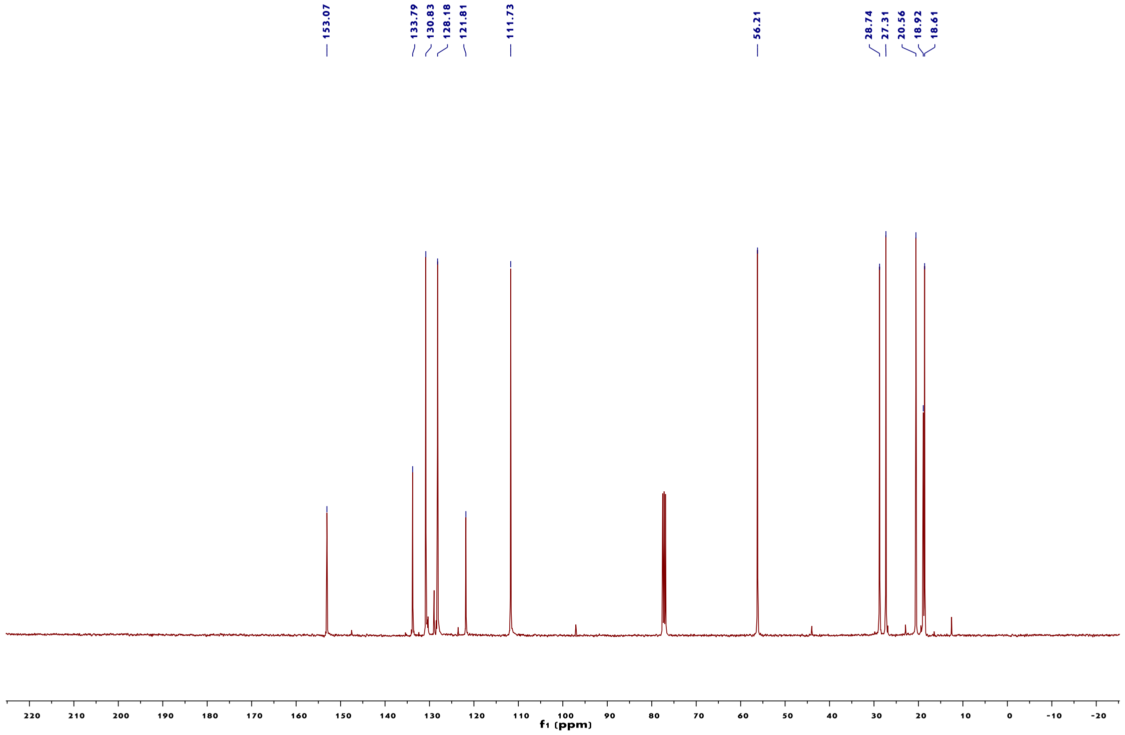

Supplementary Figure 43. ^{13}C NMR (100 MHz, $CDCl_3$) spectrum for 1k

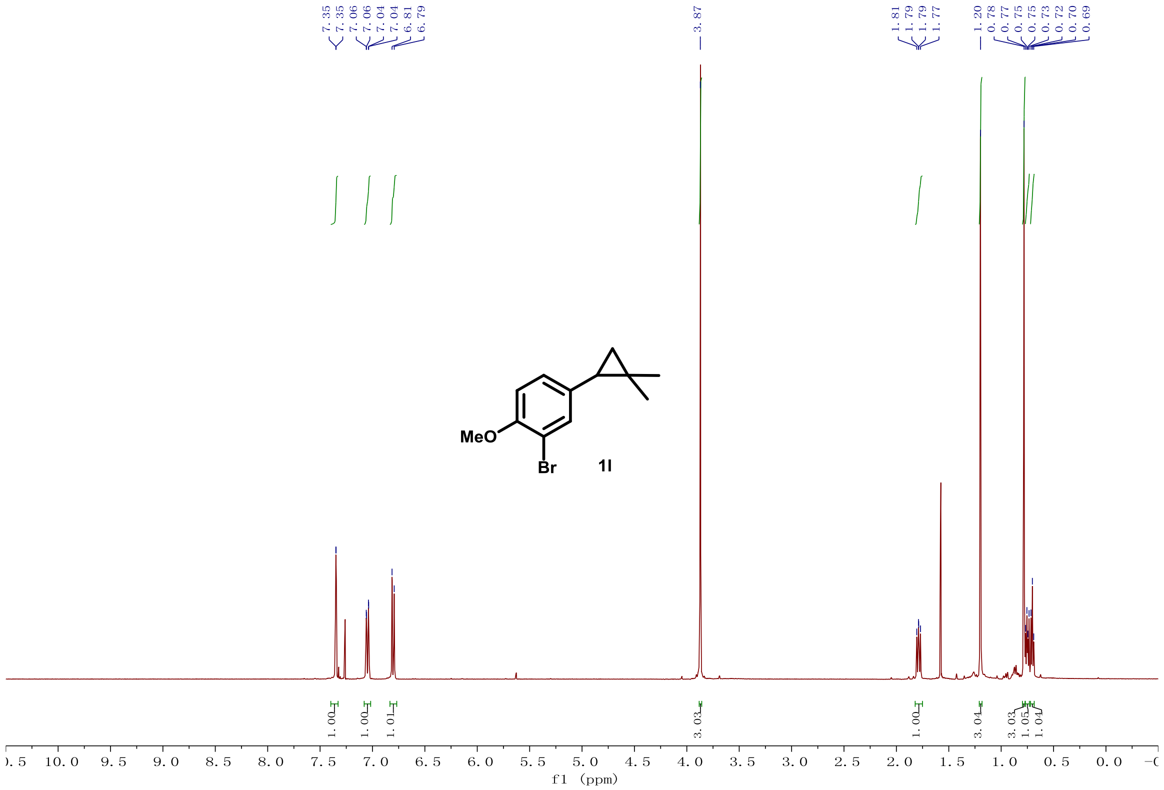

Supplementary Figure 44. ^{1}H NMR (400 MHz, $CDCl_3$) spectrum for 1l

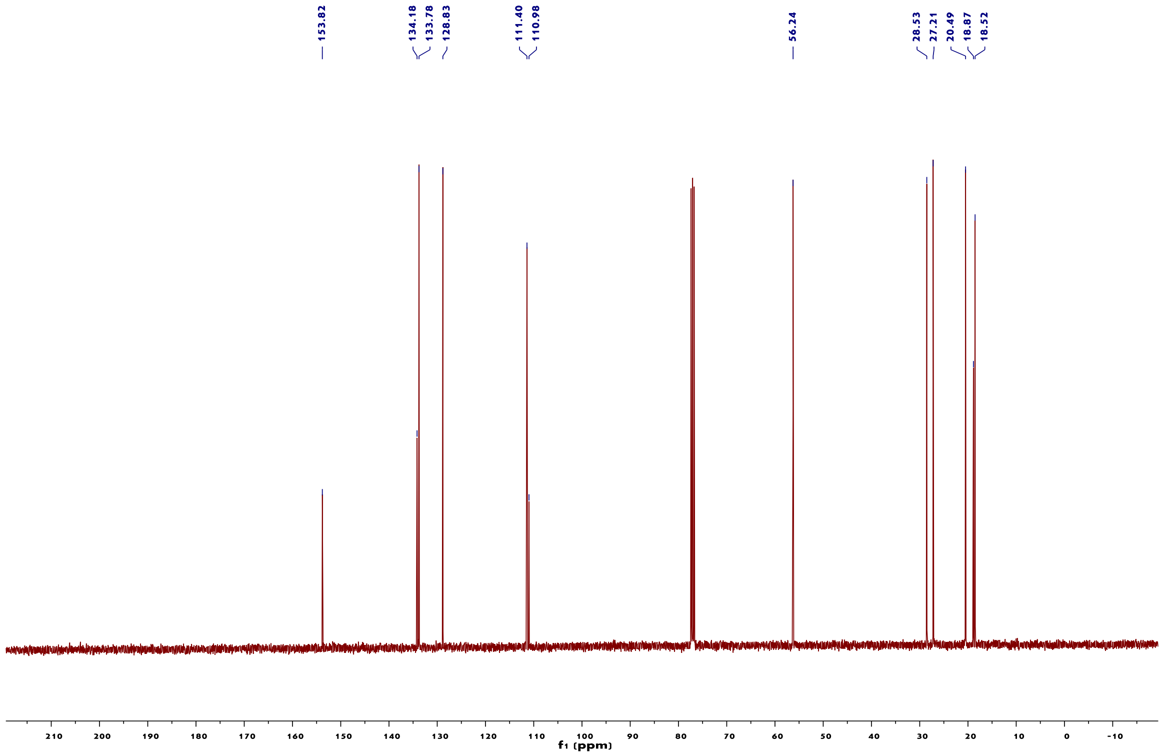

Supplementary Figure 45. ^{13}C NMR (100 MHz, $CDCl_3$) spectrum for 1l

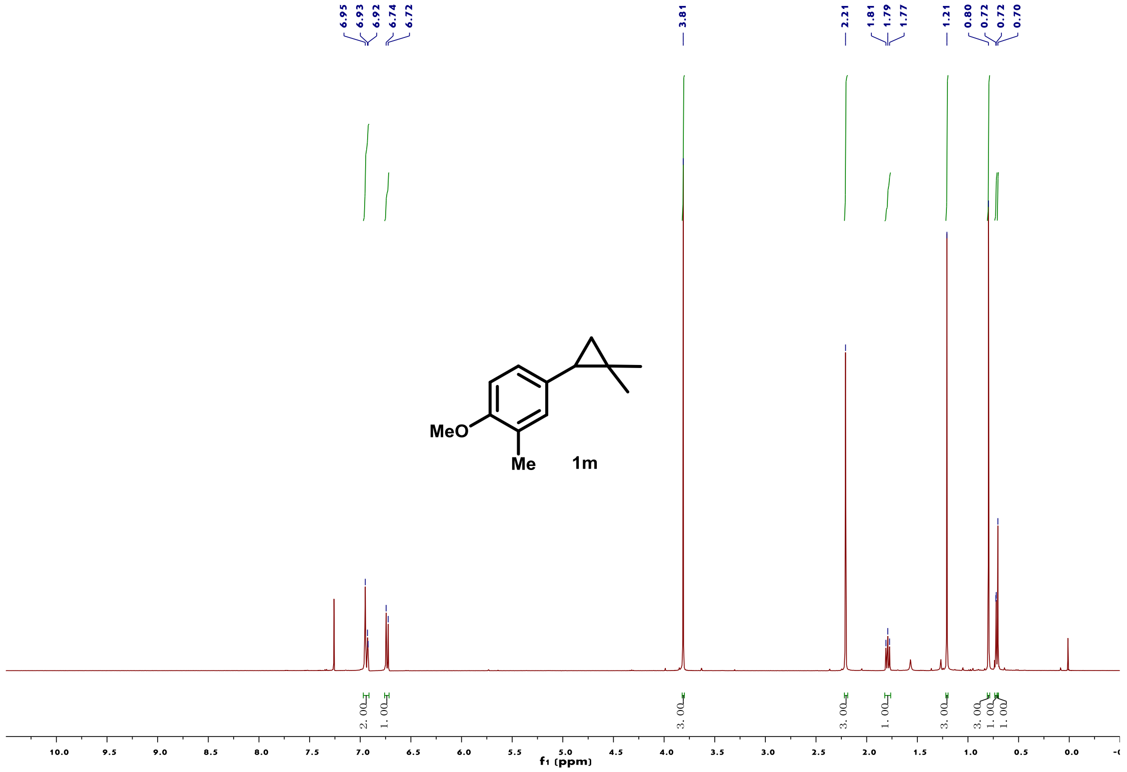

Supplementary Figure 46. ^{1}H NMR (400 MHz, $CDCl_3$) spectrum for 1m

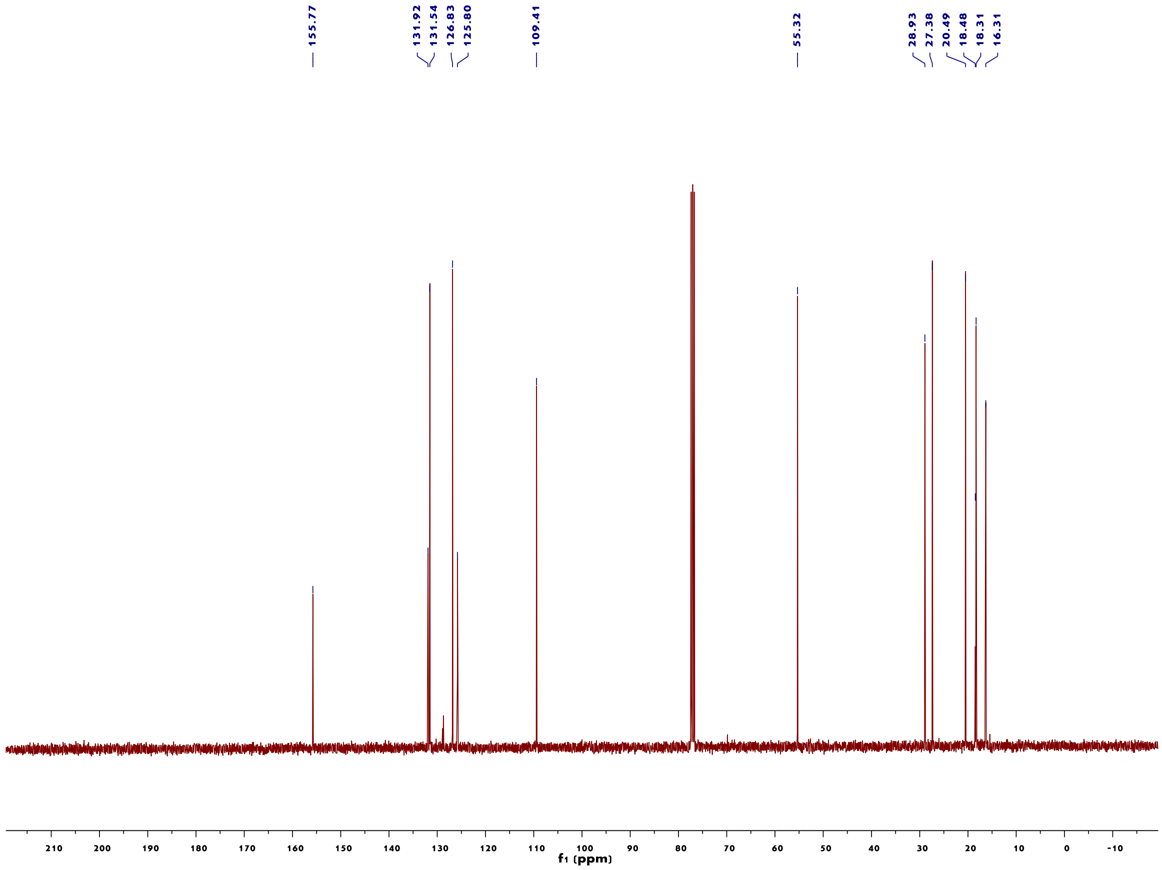

Supplementary Figure 47. ^{13}C NMR (100 MHz, $CDCl_3$) spectrum for 1m

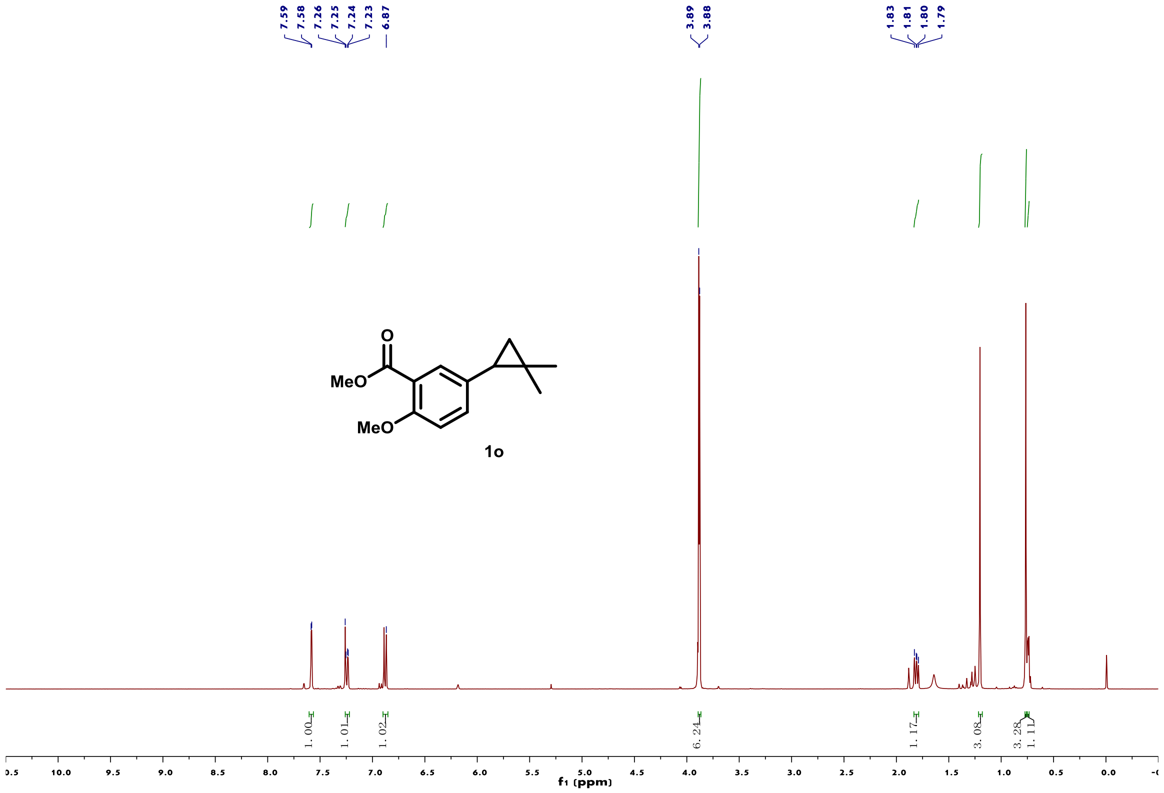

Supplementary Figure 48. ^{1}H NMR (400 MHz, $CDCl_3$) spectrum for 1o

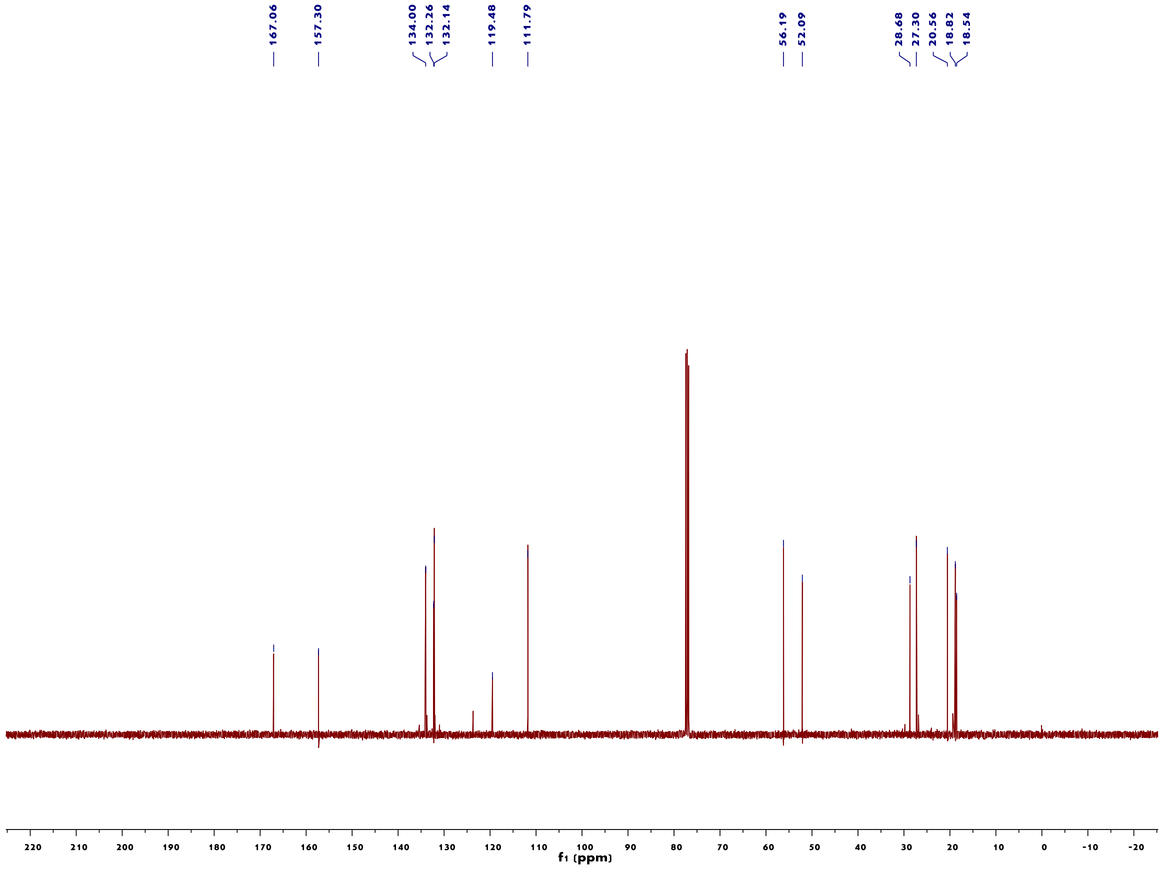

Supplementary Figure 49. ^{13}C NMR (100 MHz, $CDCl_3$) spectrum for 1o

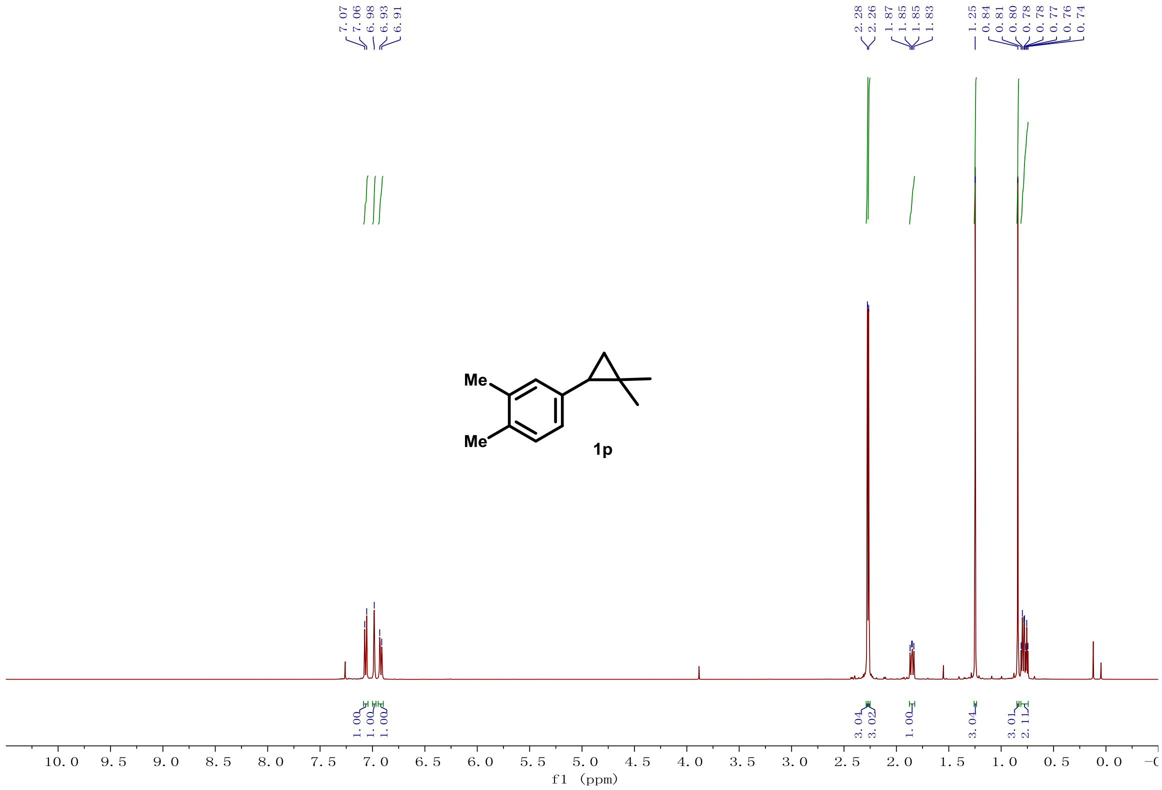

Supplementary Figure 50. ^{1}H NMR (400 MHz, $CDCl_3$) spectrum for 1p

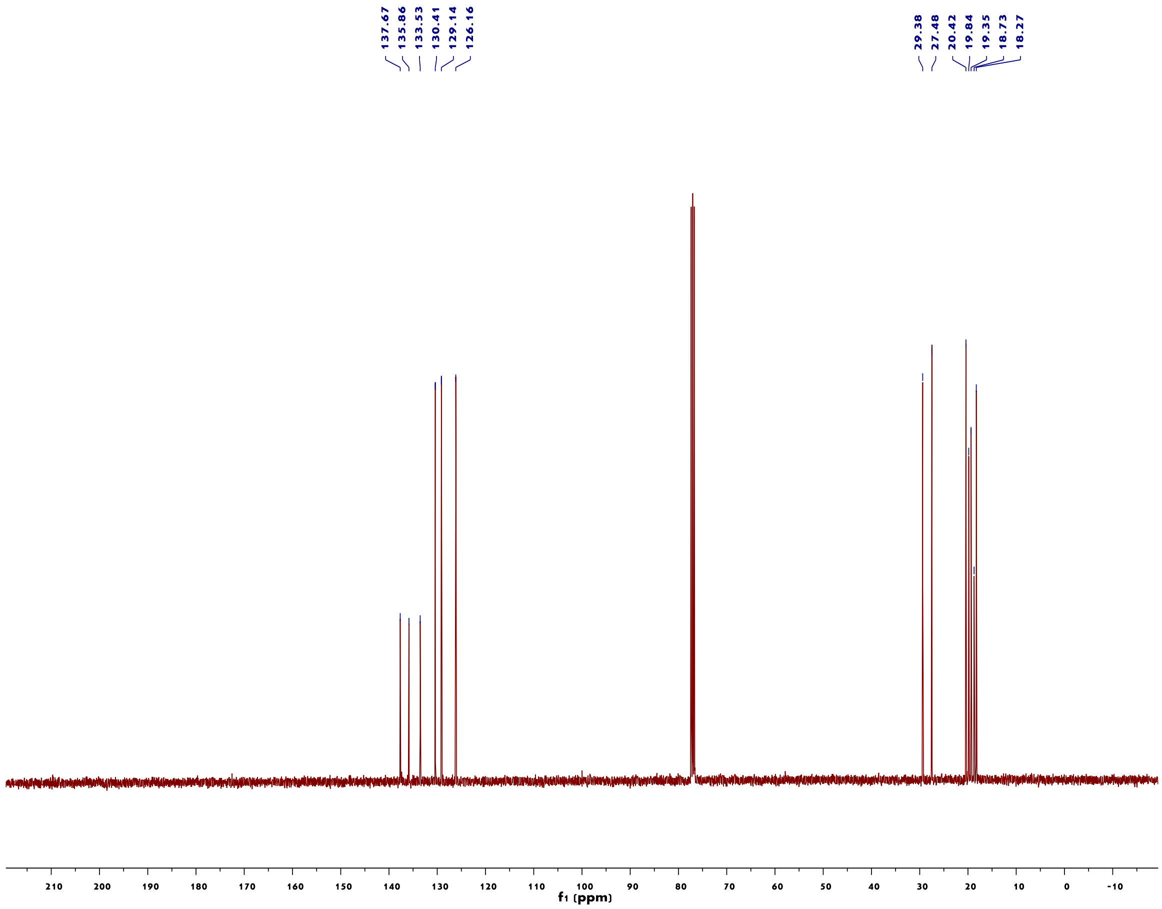

Supplementary Figure 51. ^{13}C NMR (100 MHz, $CDCl_3$) spectrum for 1p

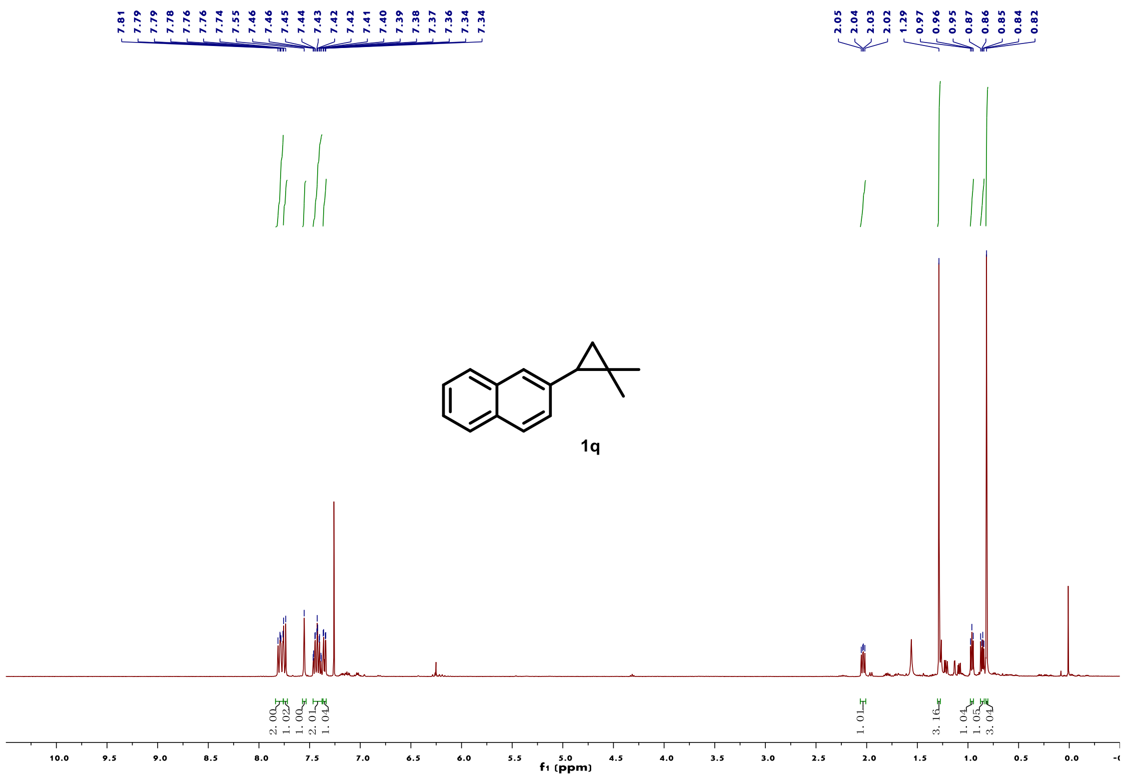

Supplementary Figure 52. ^{1}H NMR (400 MHz, $CDCl_3$) spectrum for 1q

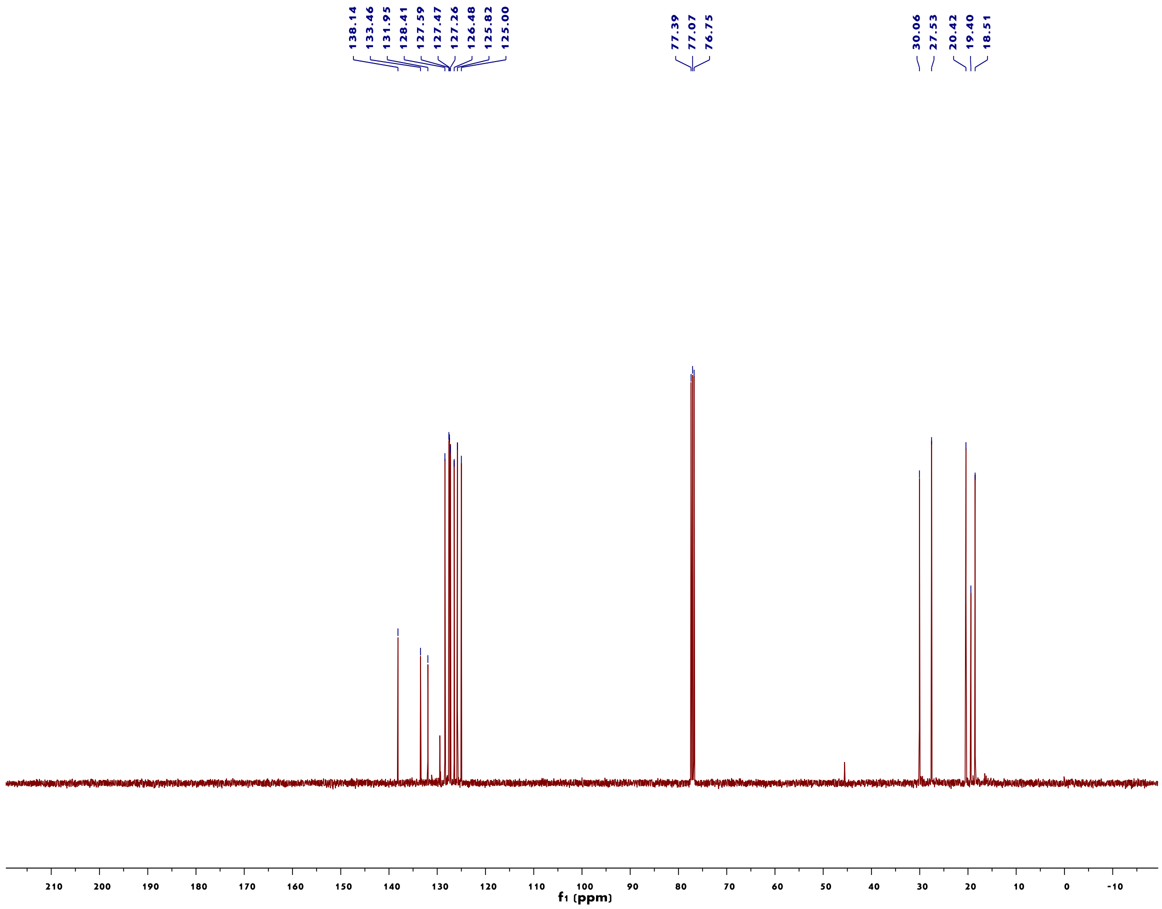

Supplementary Figure 53. ^{13}C NMR (100 MHz, $CDCl_3$) spectrum for 1q

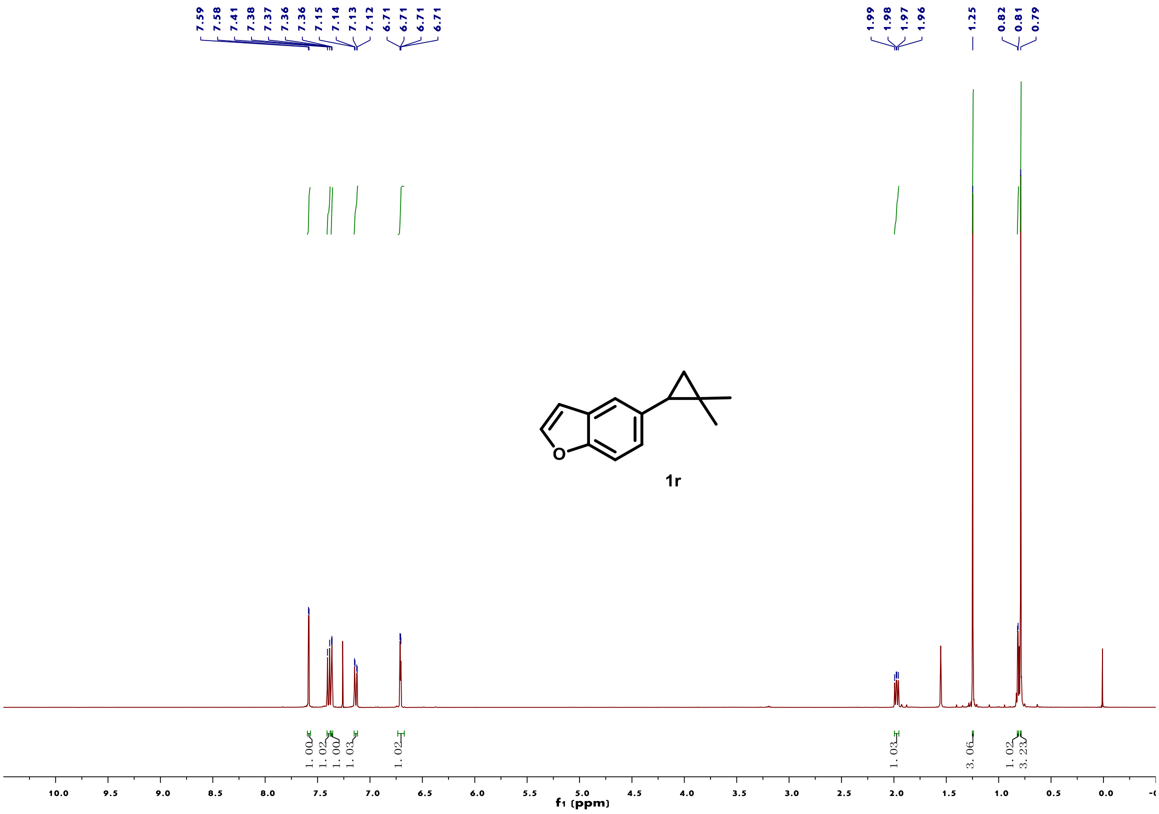

Supplementary Figure 54. ^{1}H NMR (400 MHz, $CDCl_3$) spectrum for 1r

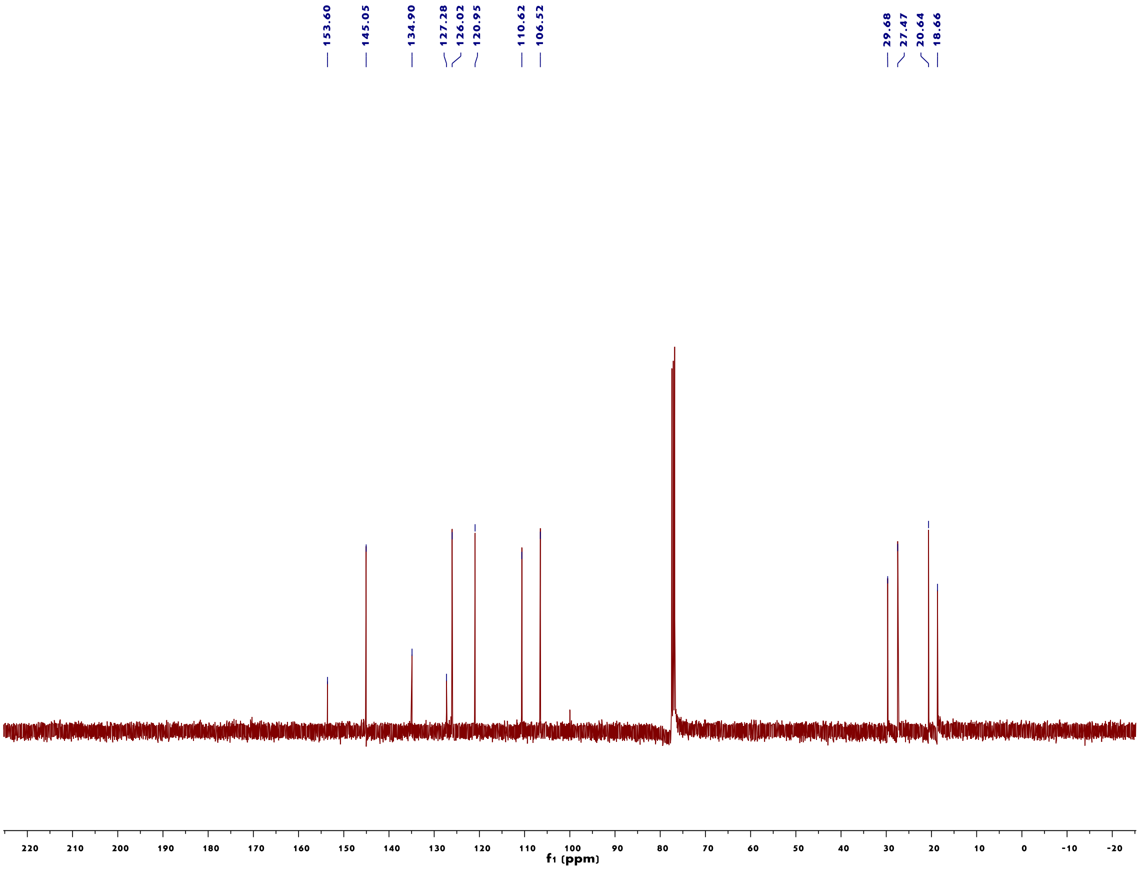

Supplementary Figure 55. ^{13}C NMR (100 MHz, $CDCl_3$) spectrum for 1r

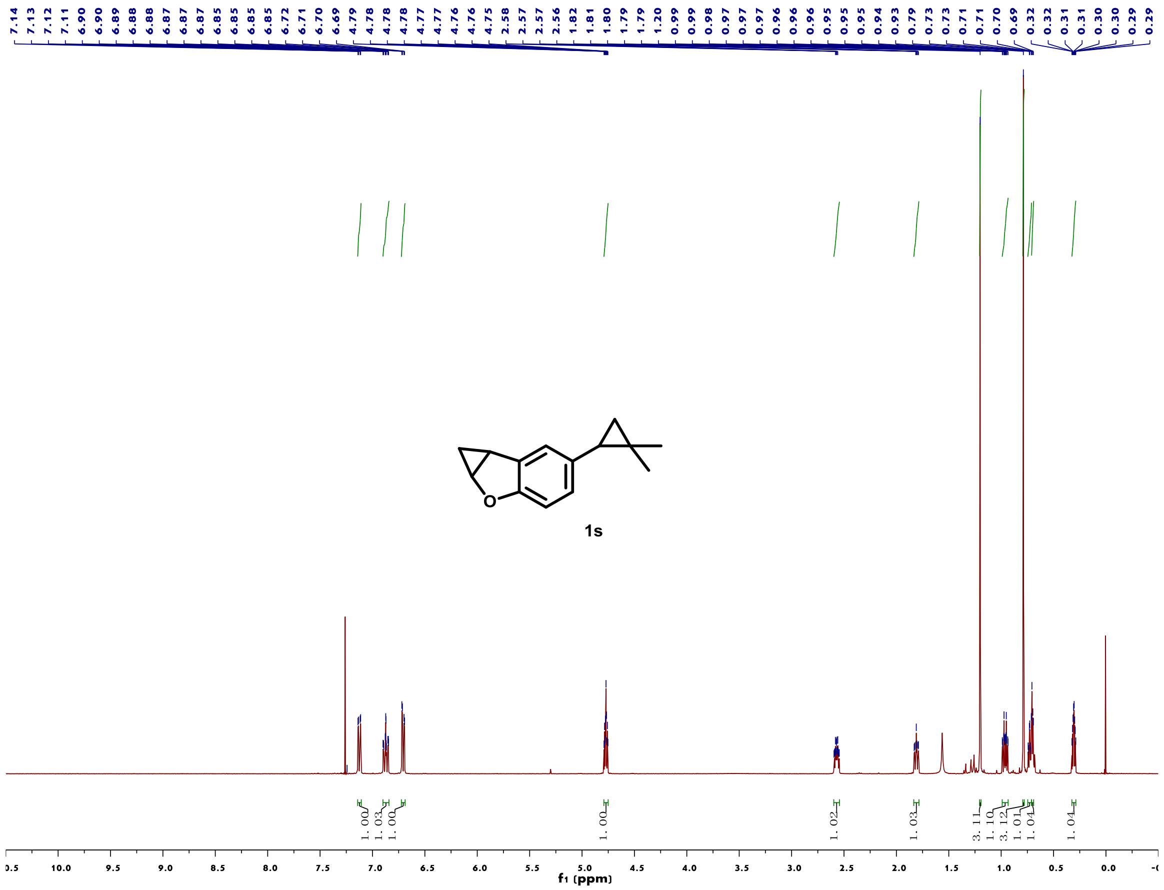

Supplementary Figure 56. ^{1}H NMR (400 MHz, $CDCl_3$) spectrum for 1s

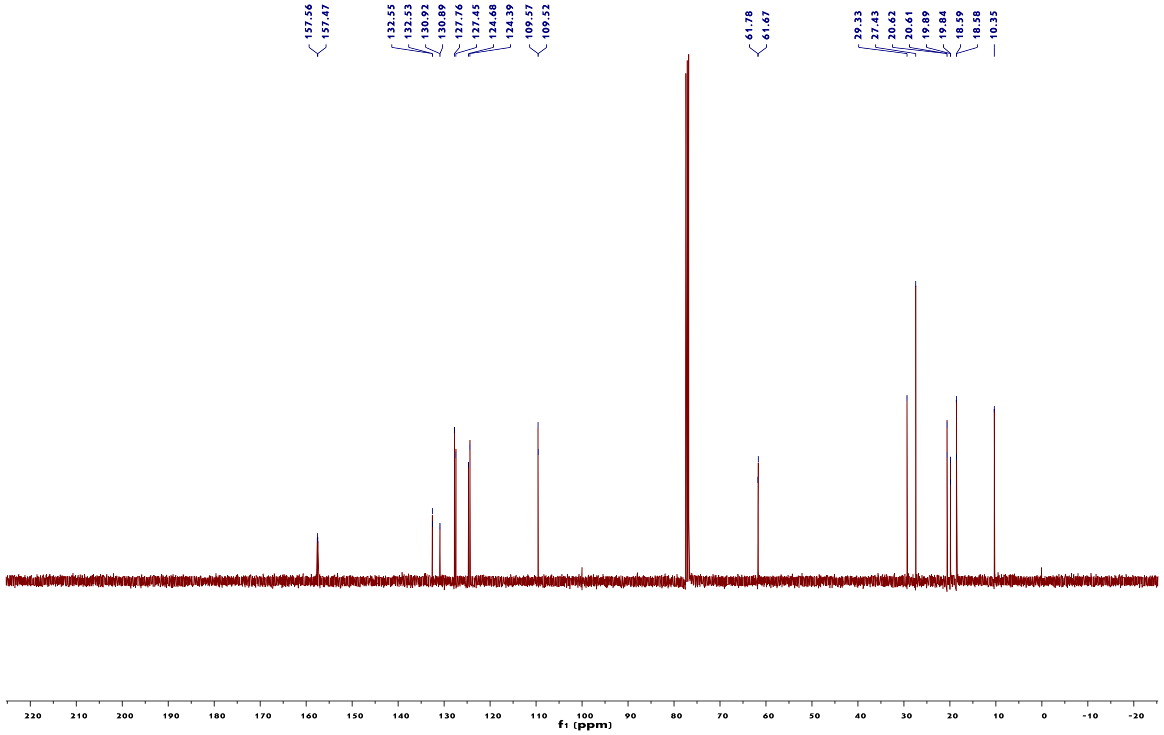

Supplementary Figure 57. ^{13}C NMR (100 MHz, $CDCl_3$) spectrum for 1s

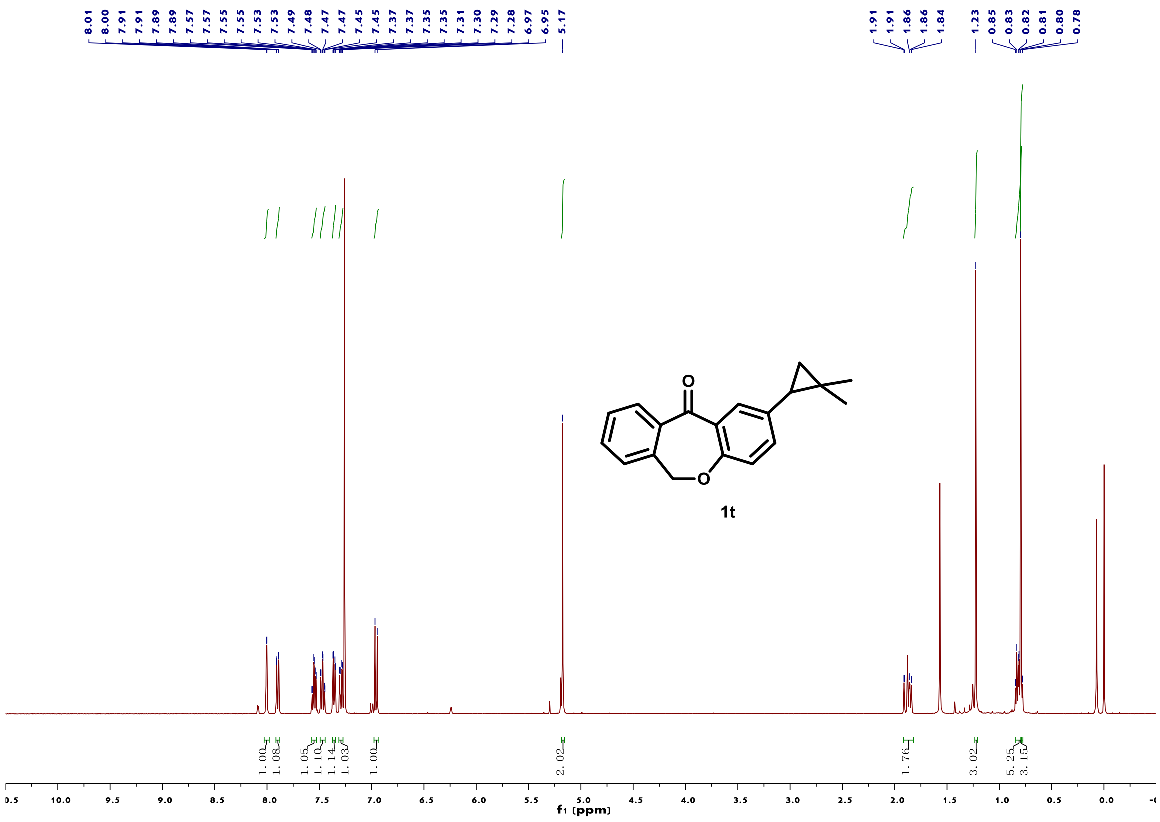

Supplementary Figure 58. ^{1}H NMR (400 MHz, $CDCl_3$) spectrum for 1t

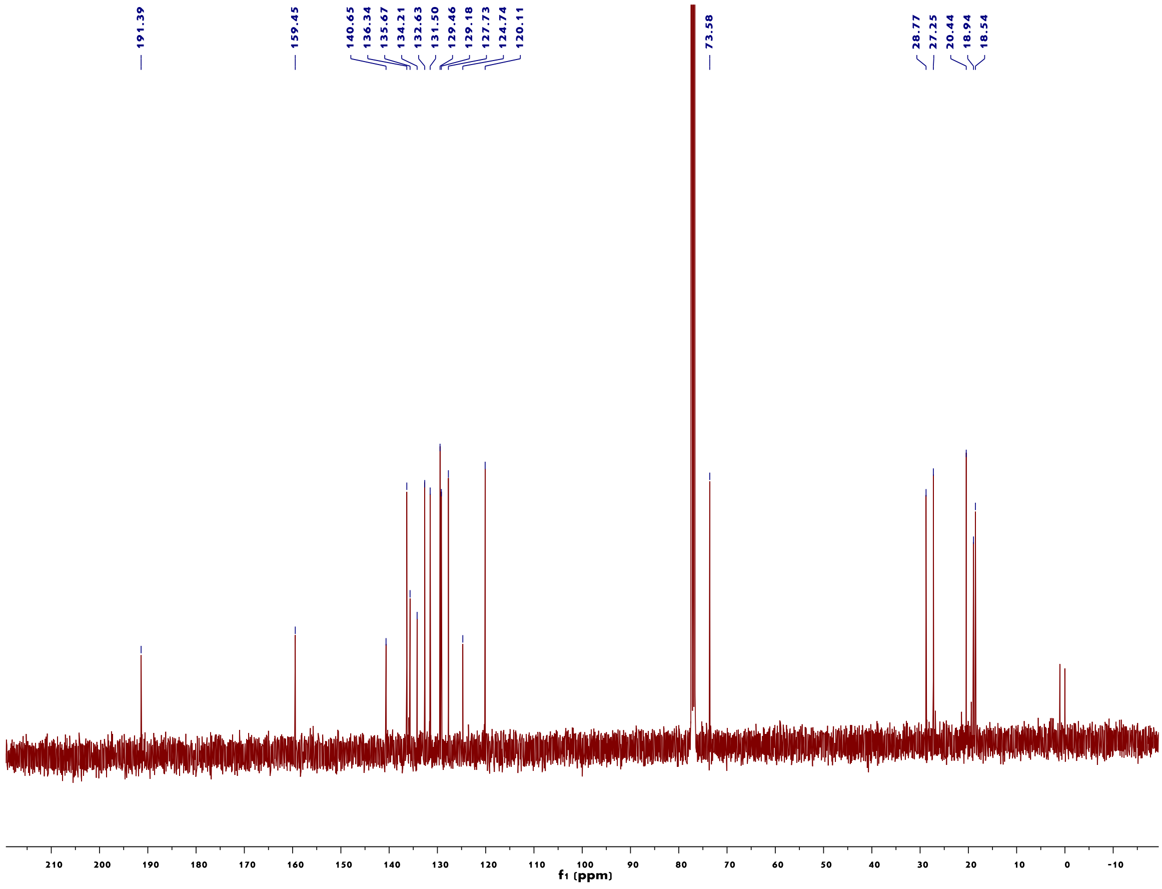

Supplementary Figure 59. ^{13}C NMR (100 MHz, $CDCl_3$) spectrum for 1t

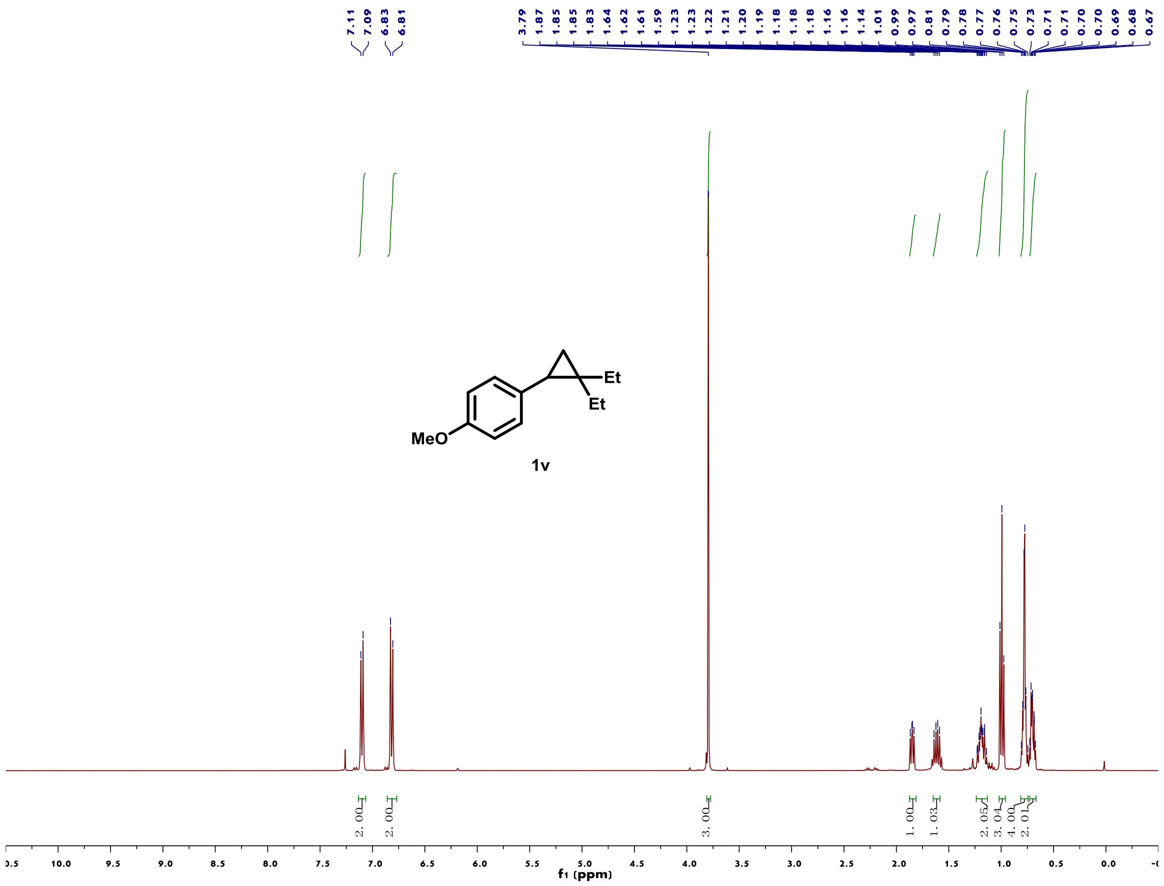

Supplementary Figure 60. ^{1}H NMR (400 MHz, $CDCl_3$) spectrum for 1v

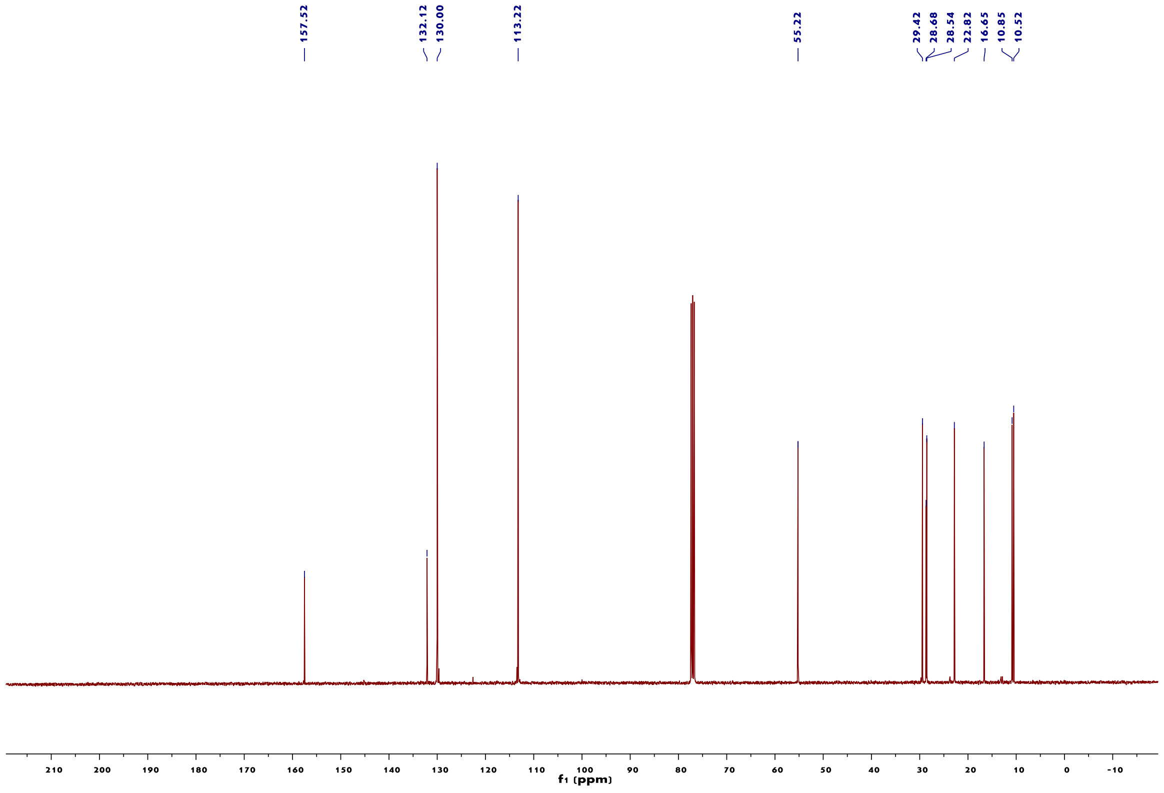

Supplementary Figure 61. ^{13}C NMR (100 MHz, $CDCl_3$) spectrum for 1v

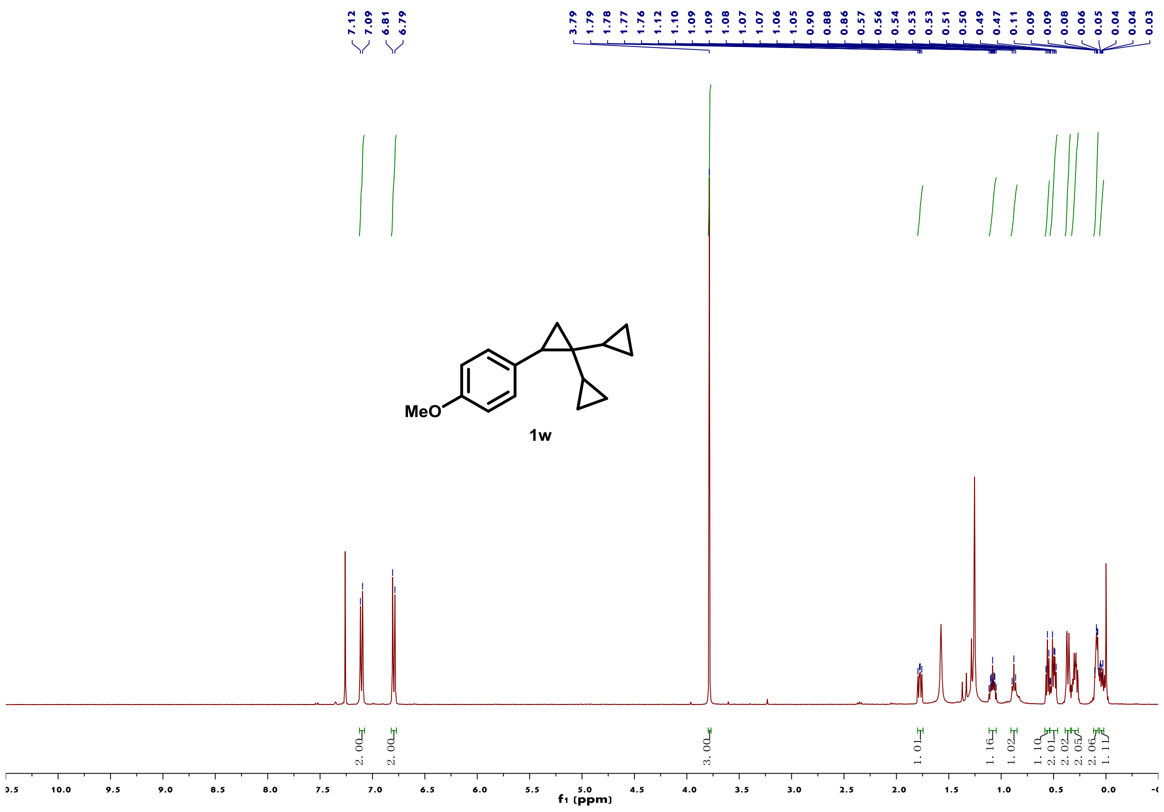

Supplementary Figure 62. ^{1}H NMR (400 MHz, $CDCl_3$) spectrum for 1w

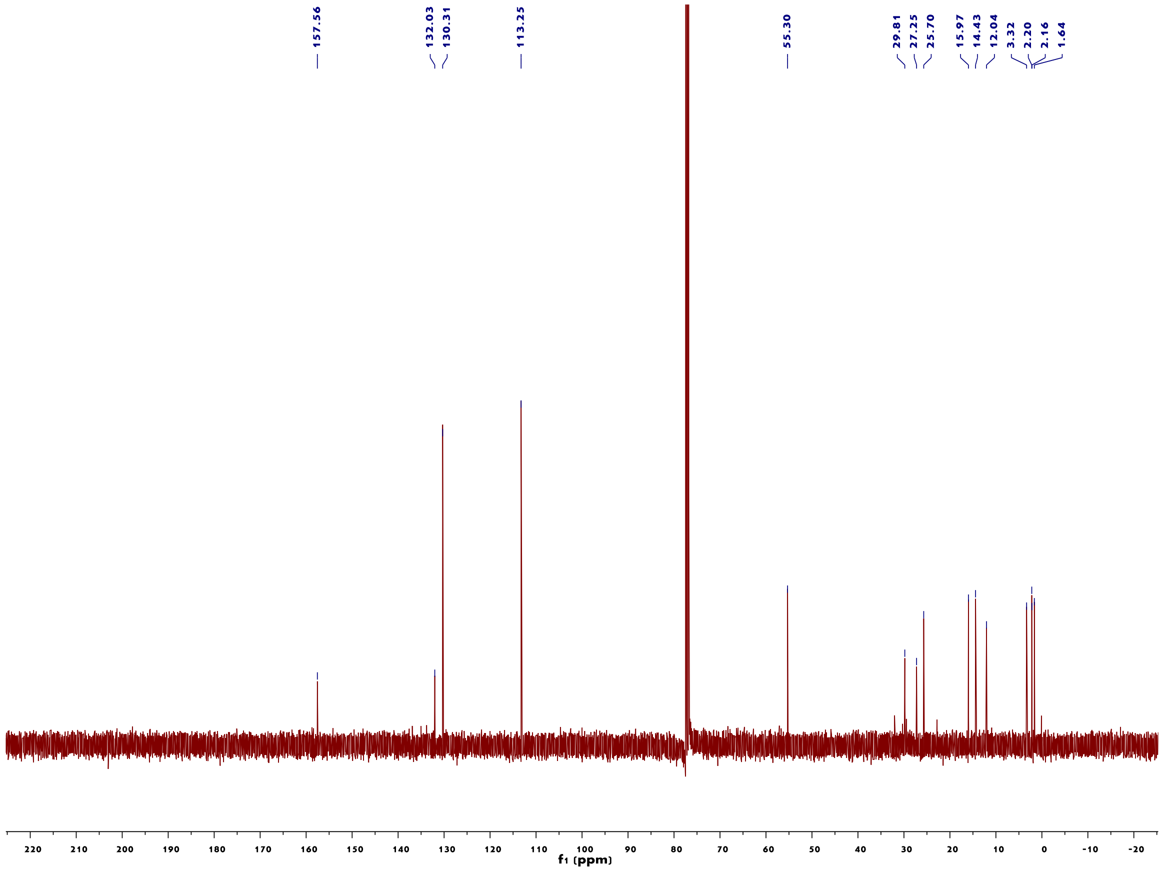

Supplementary Figure 63. ^{13}C NMR (100 MHz, $CDCl_3$) spectrum for 1w

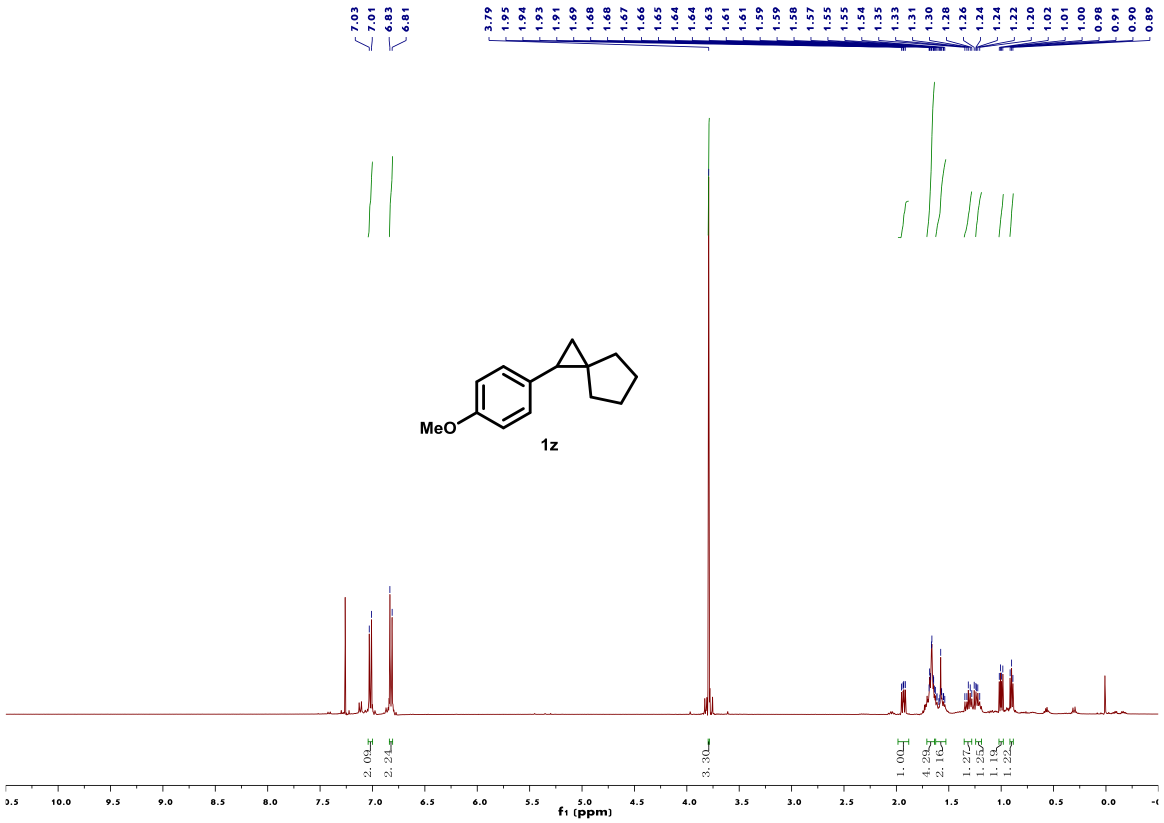

Supplementary Figure 64. ^{1}H NMR (400 MHz, $CDCl_3$) spectrum for 1z

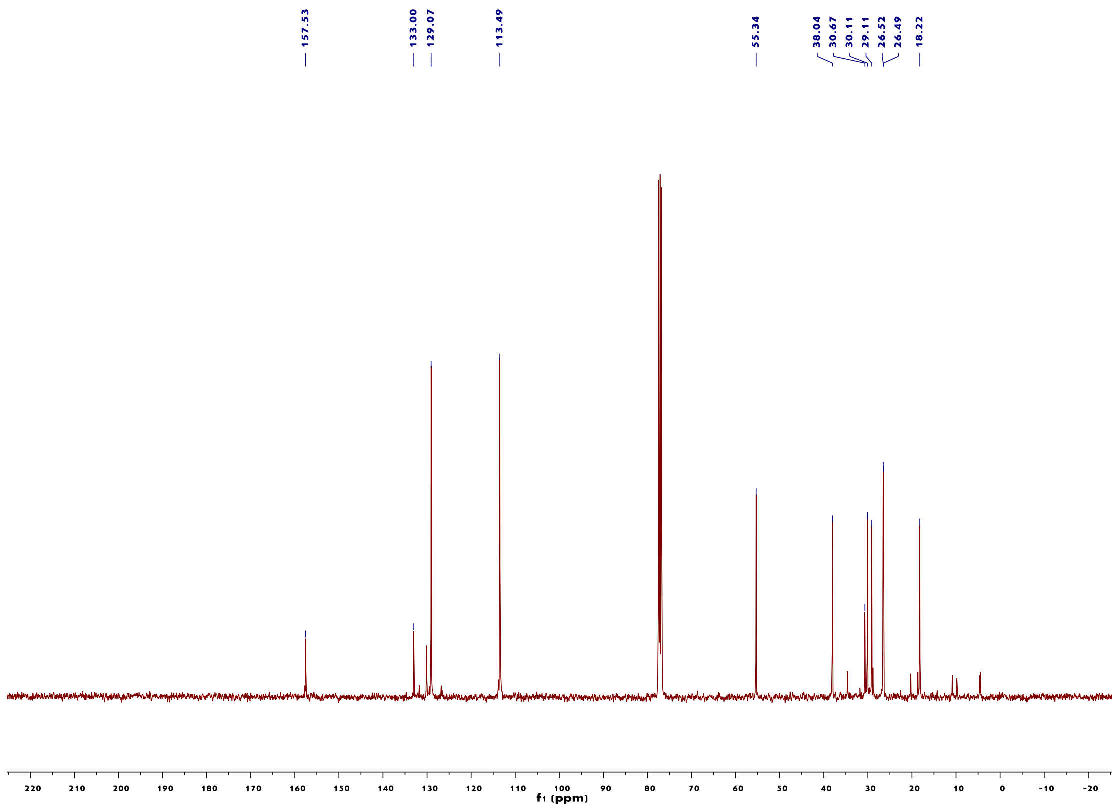

Supplementary Figure 65. ^{13}C NMR (100 MHz, $CDCl_3$) spectrum for 1z

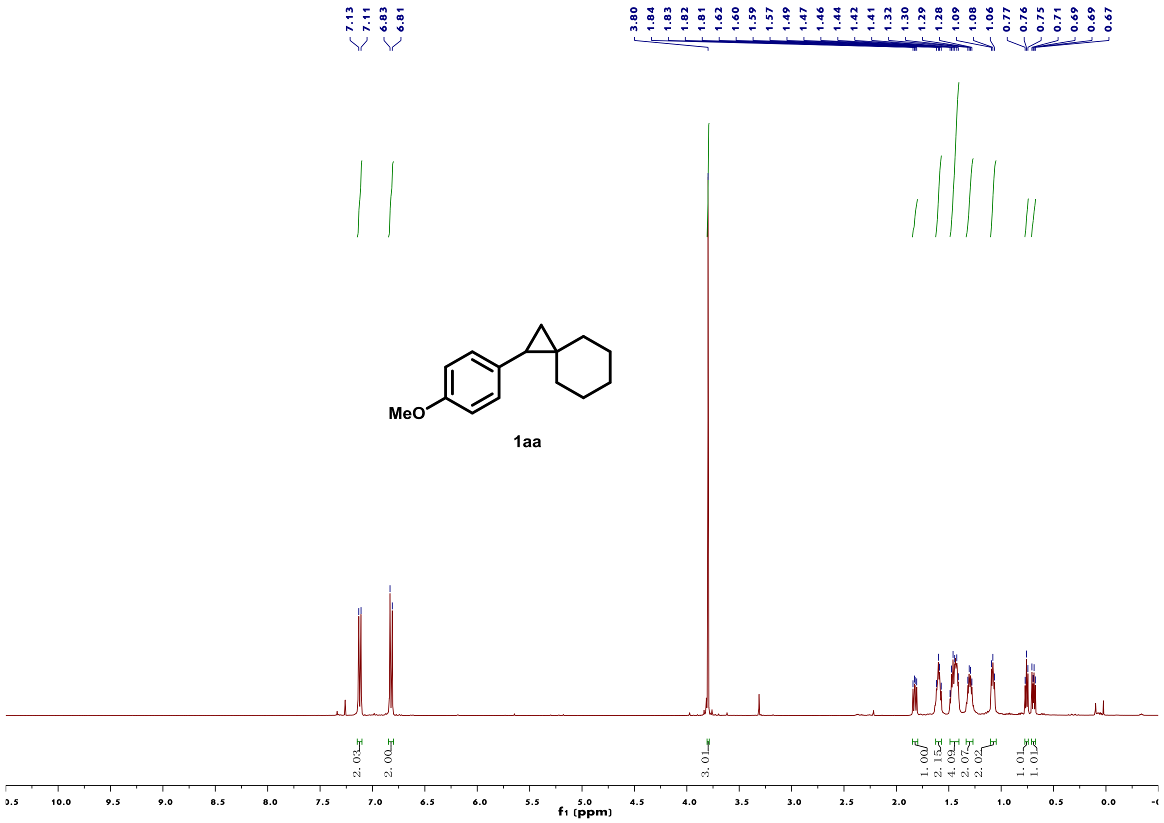

Supplementary Figure 66. ^{1}H NMR (400 MHz, $CDCl_3$) spectrum for 1aa

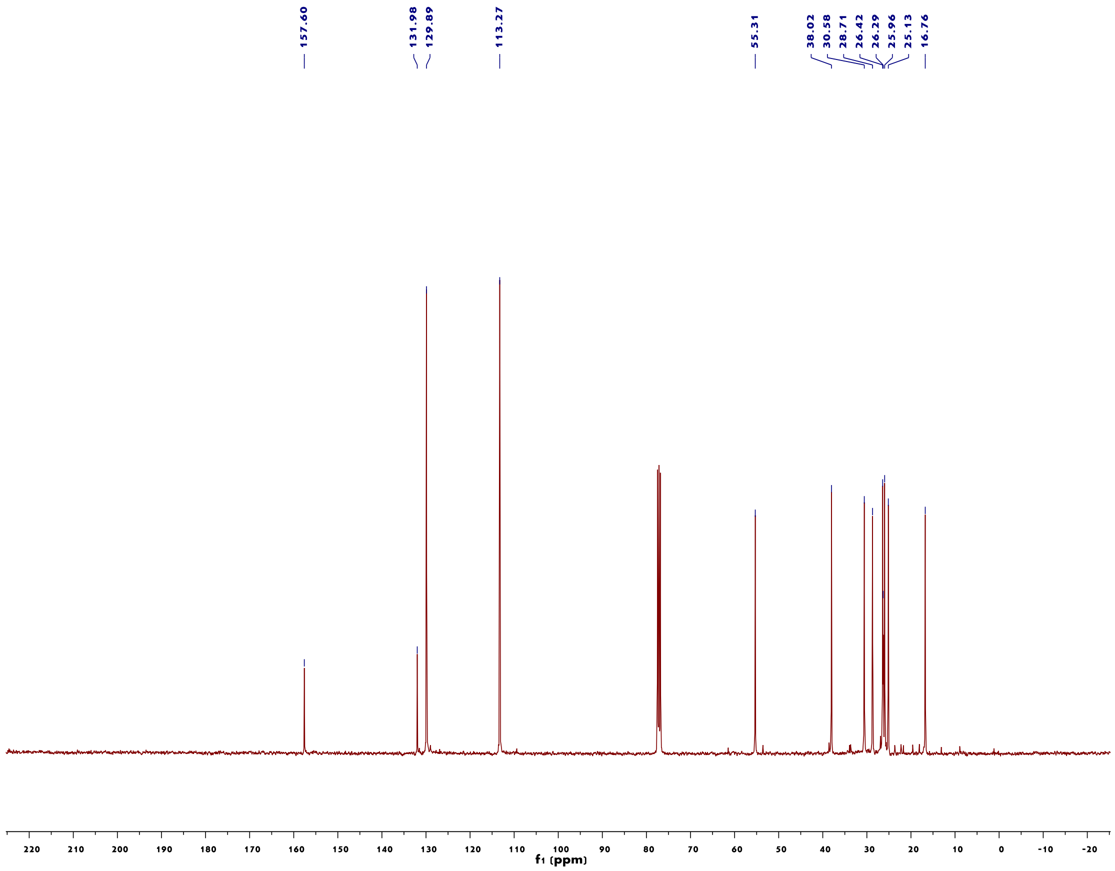

Supplementary Figure 67. ^{13}C NMR (100 MHz, $CDCl_3$) spectrum for 1aa

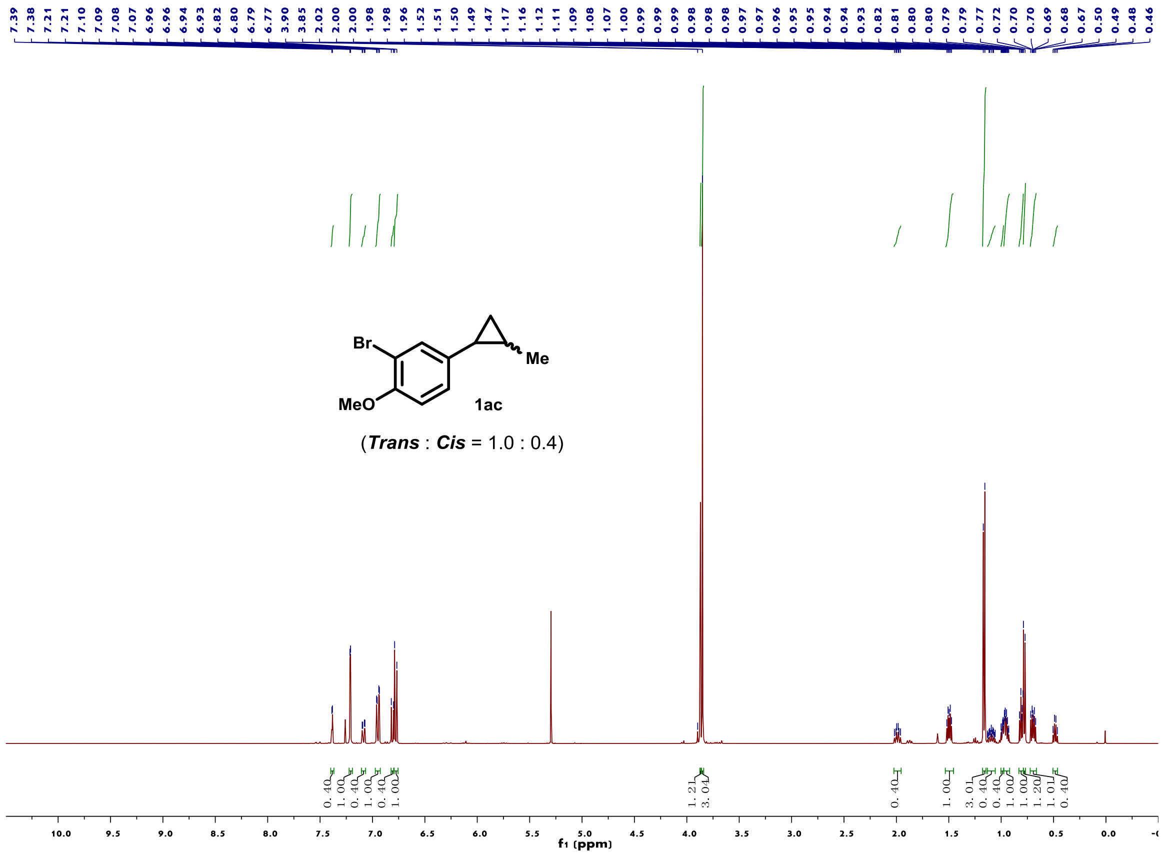

Supplementary Figure 68. ^{1}H NMR (400 MHz, $CDCl_3$) spectrum for 1ac

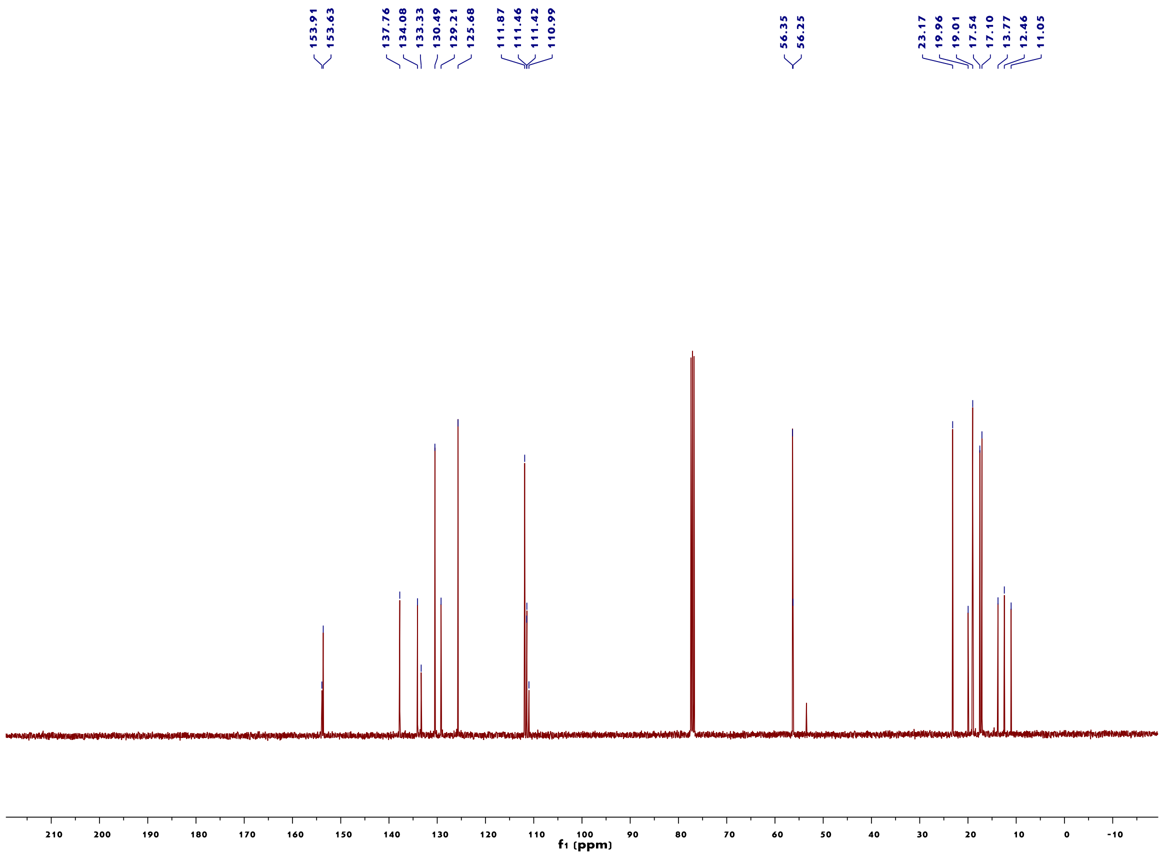

Supplementary Figure 69. ^{13}C NMR (100 MHz, $CDCl_3$) spectrum for 1ac

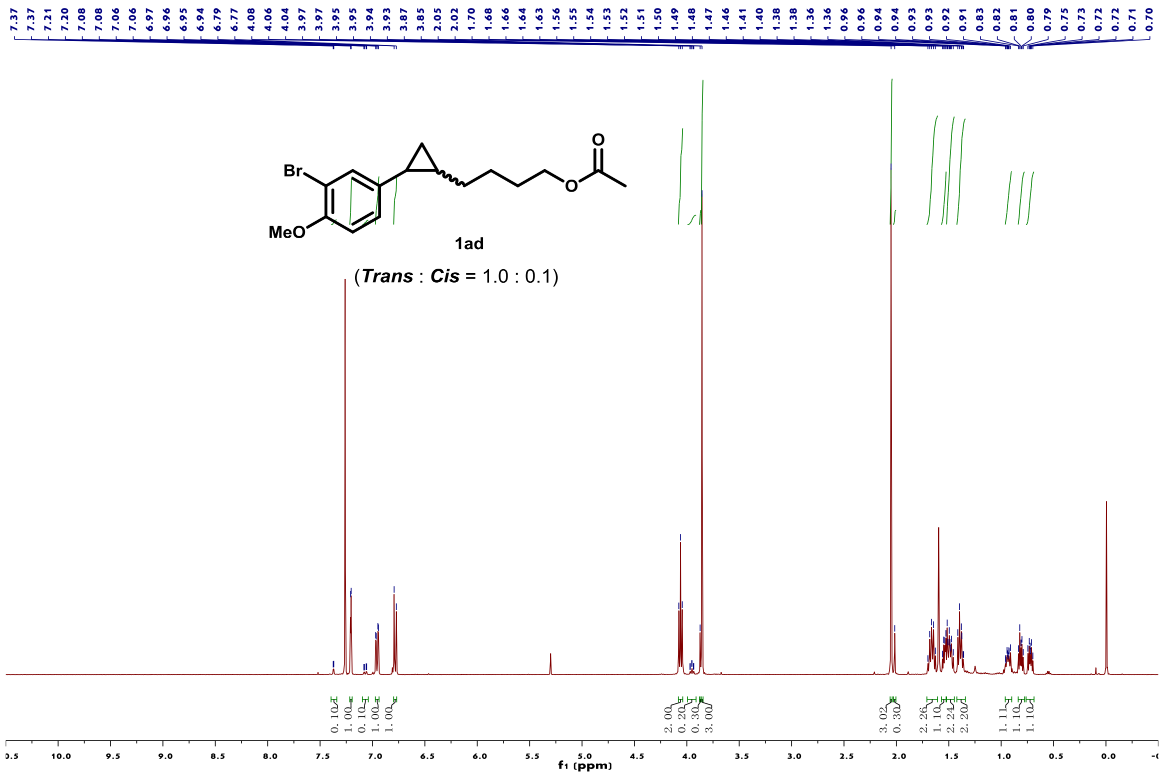

Supplementary Figure 70. ^{1}H NMR (400 MHz, $CDCl_3$) spectrum for 1ad

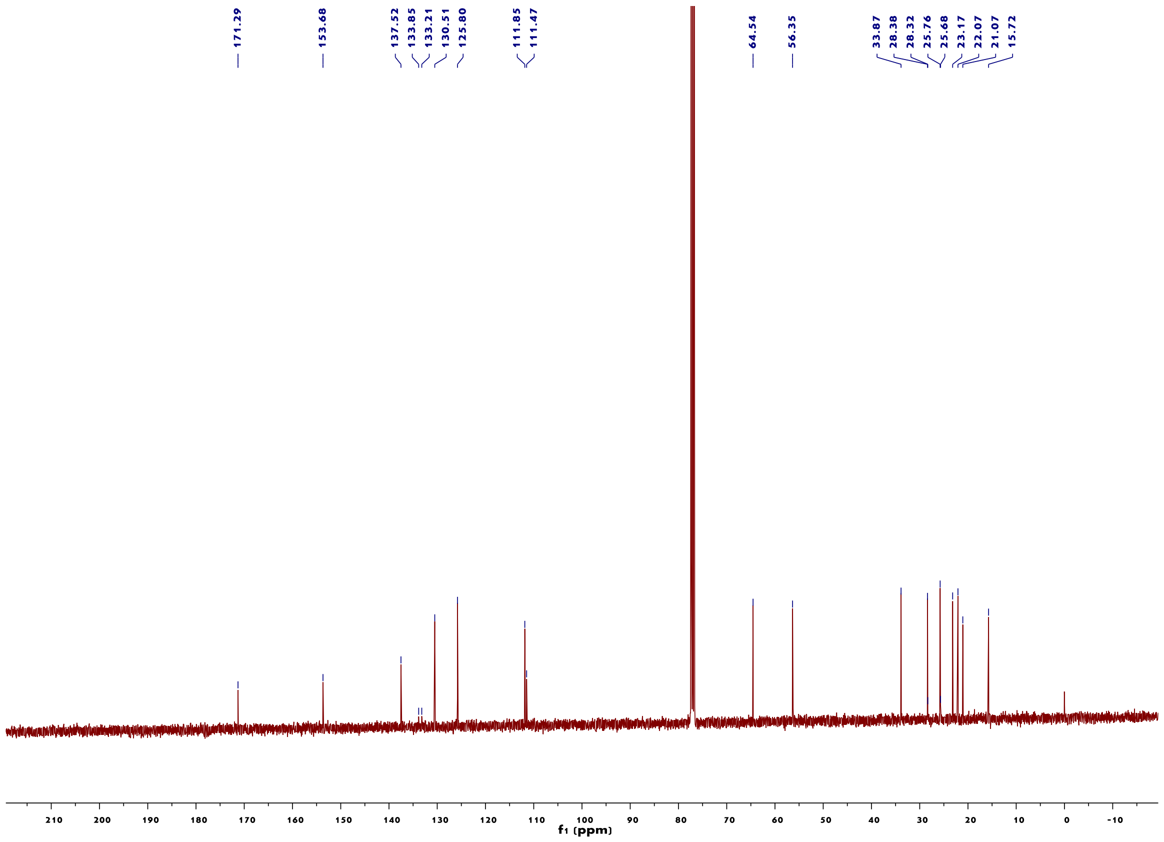

Supplementary Figure 71. ^{13}C NMR (100 MHz, $CDCl_3$) spectrum for 1ad

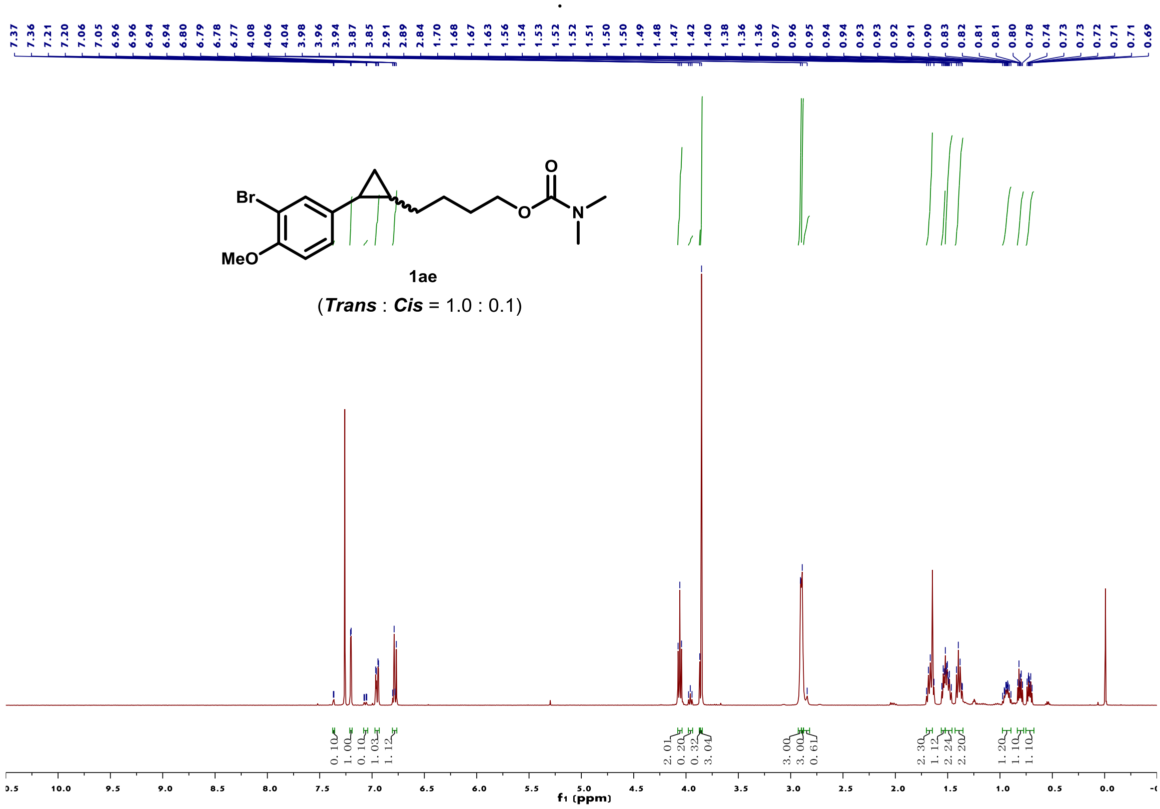

Supplementary Figure 72. ^{1}H NMR (400 MHz, $CDCl_3$) spectrum for 1ae

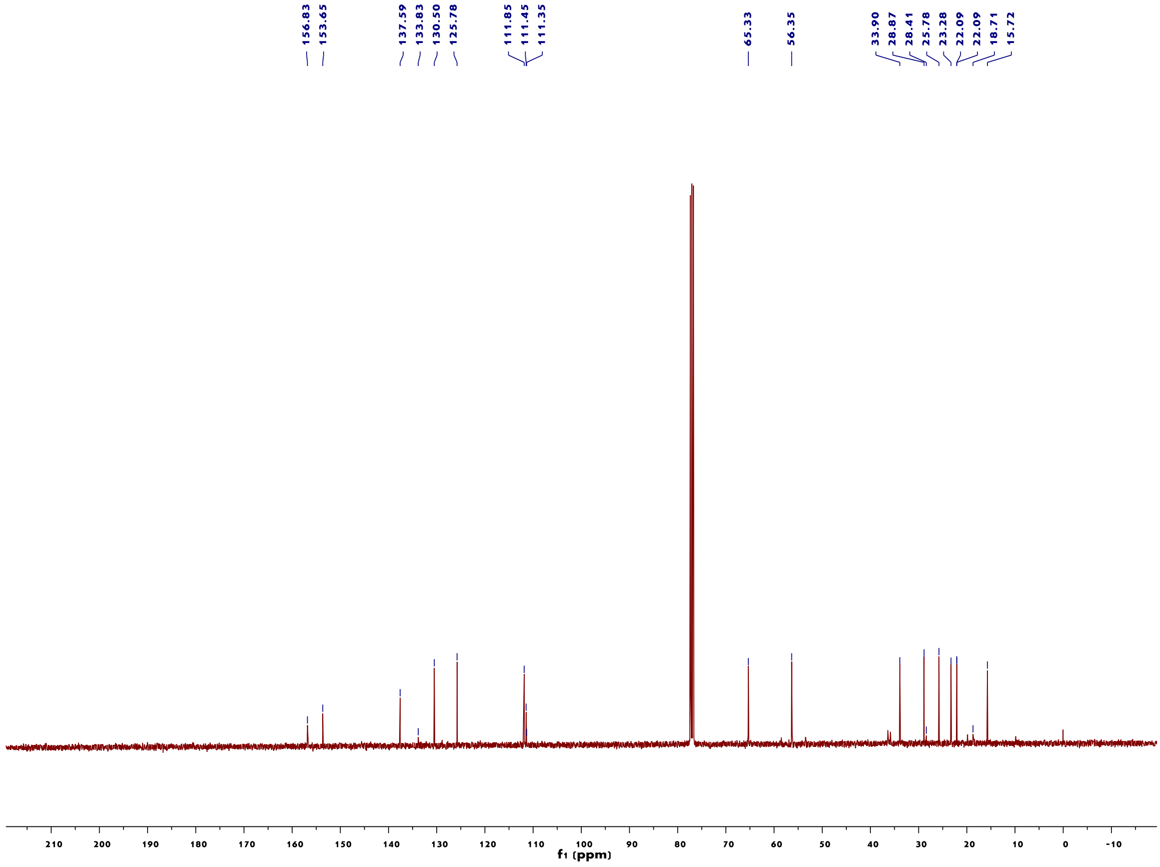

Supplementary Figure 73. ^{13}C NMR (100 MHz, $CDCl_3$) spectrum for 1ae

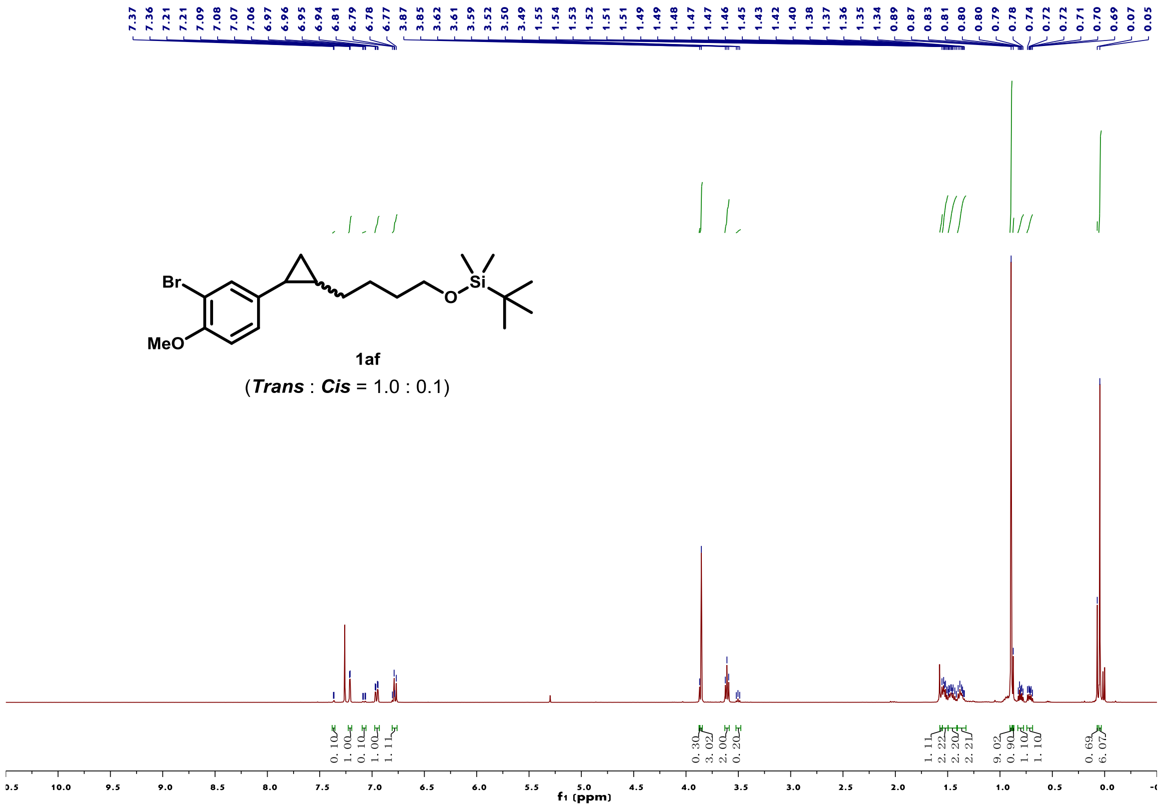

Supplementary Figure 74. ^{1}H NMR (400 MHz, $CDCl_3$) spectrum for 1af

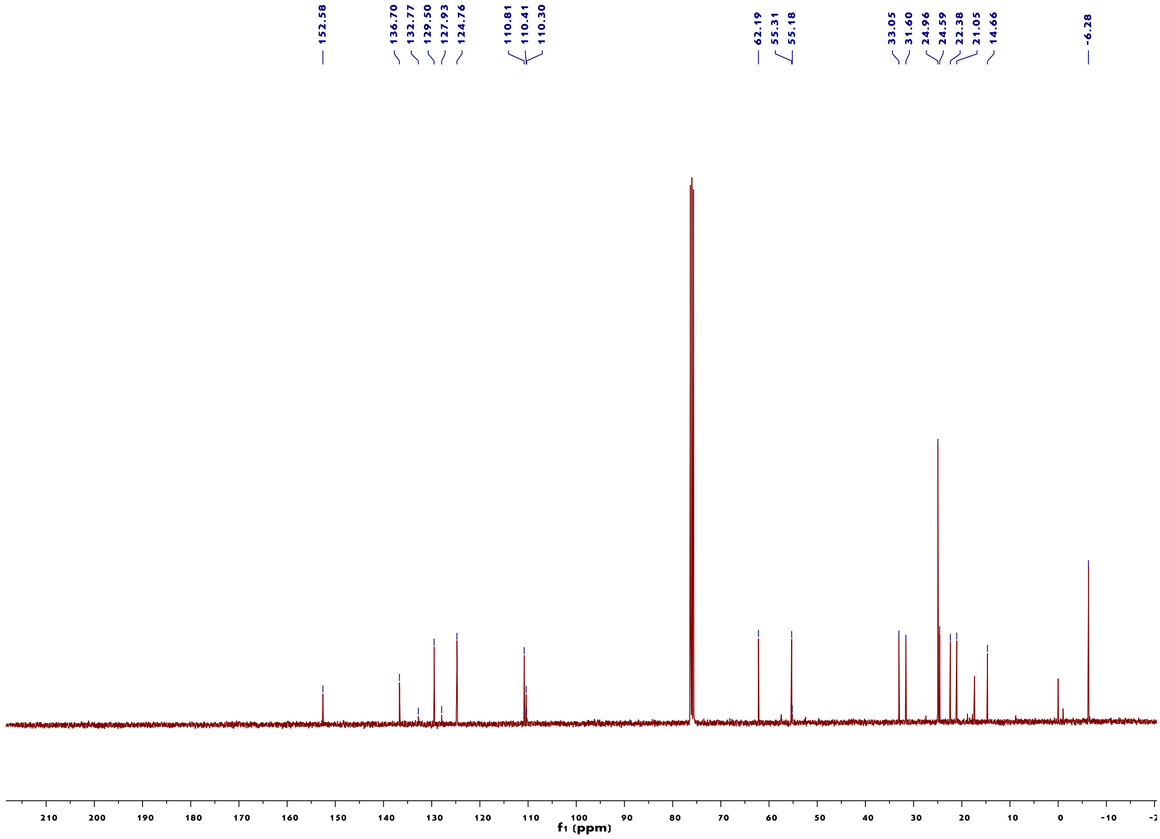

Supplementary Figure 75. ^{13}C NMR (100 MHz, $CDCl_3$) spectrum for 1af

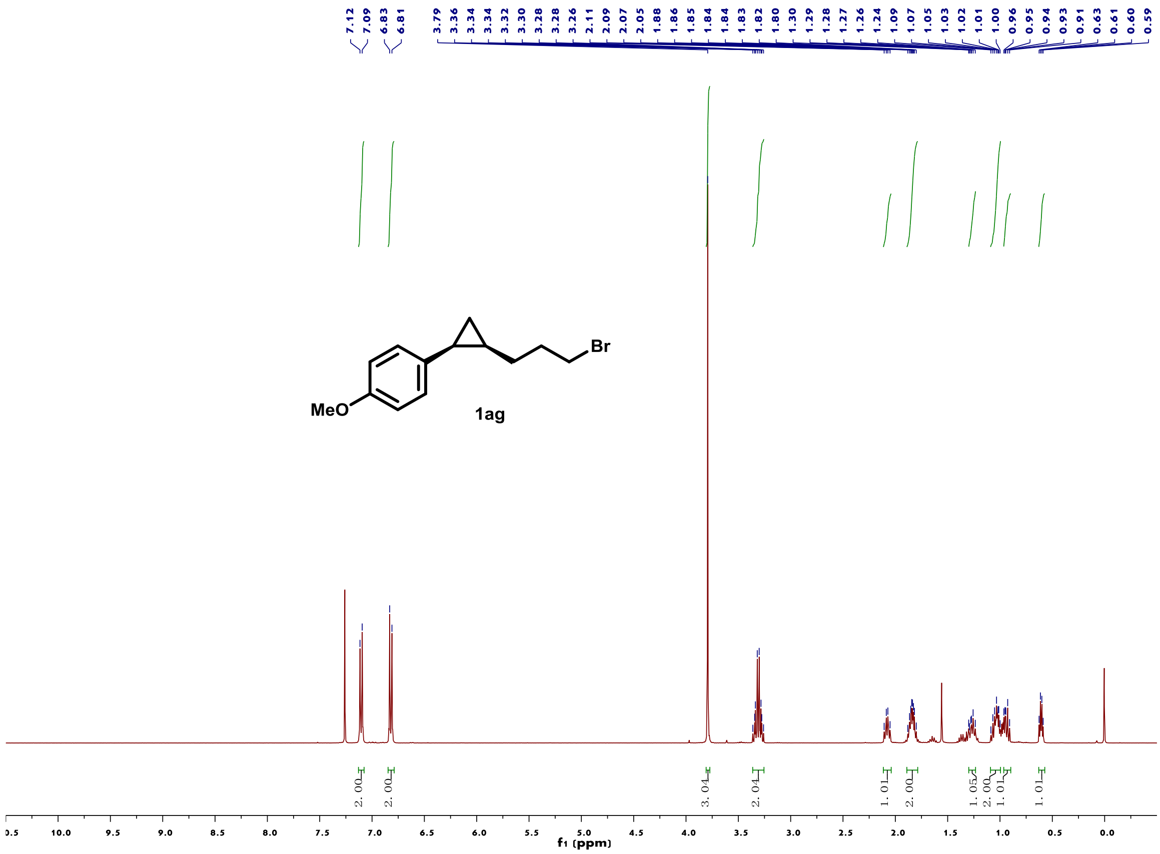

Supplementary Figure 76. ^{1}H NMR (400 MHz, $CDCl_3$) spectrum for 1ag

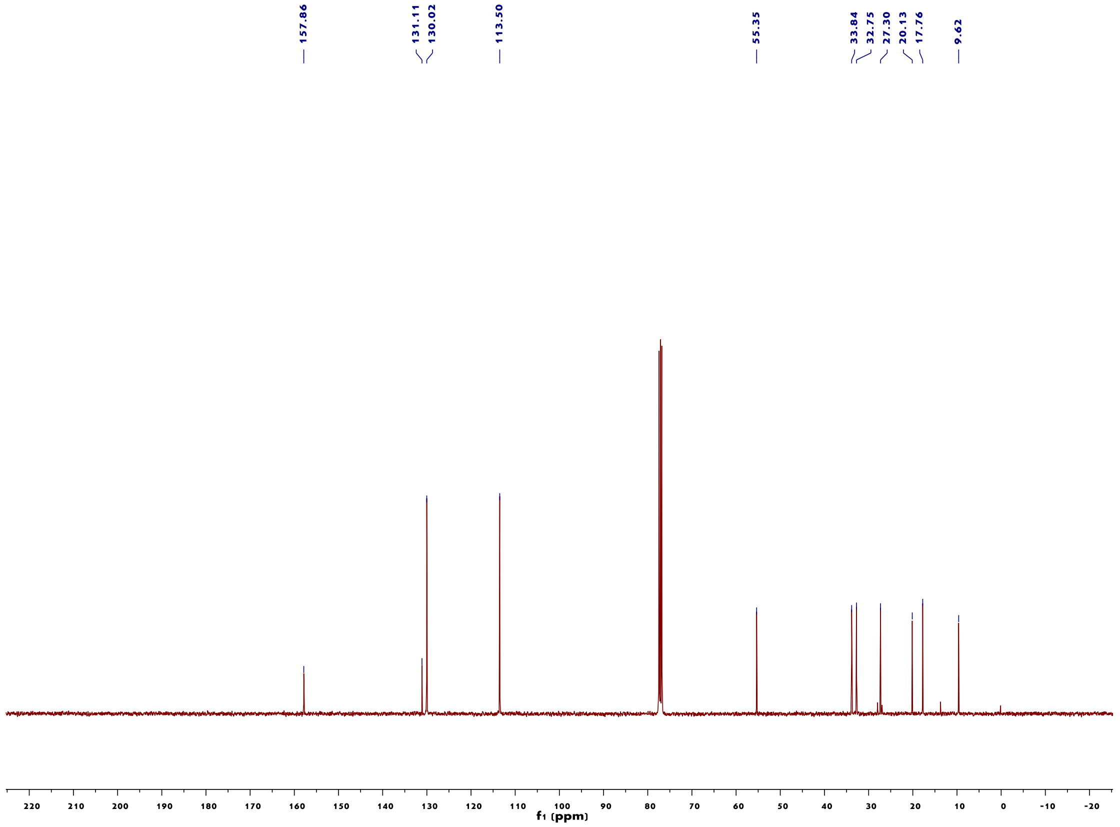

Supplementary Figure 77. ^{13}C NMR (100 MHz, $CDCl_3$) spectrum for 1ag

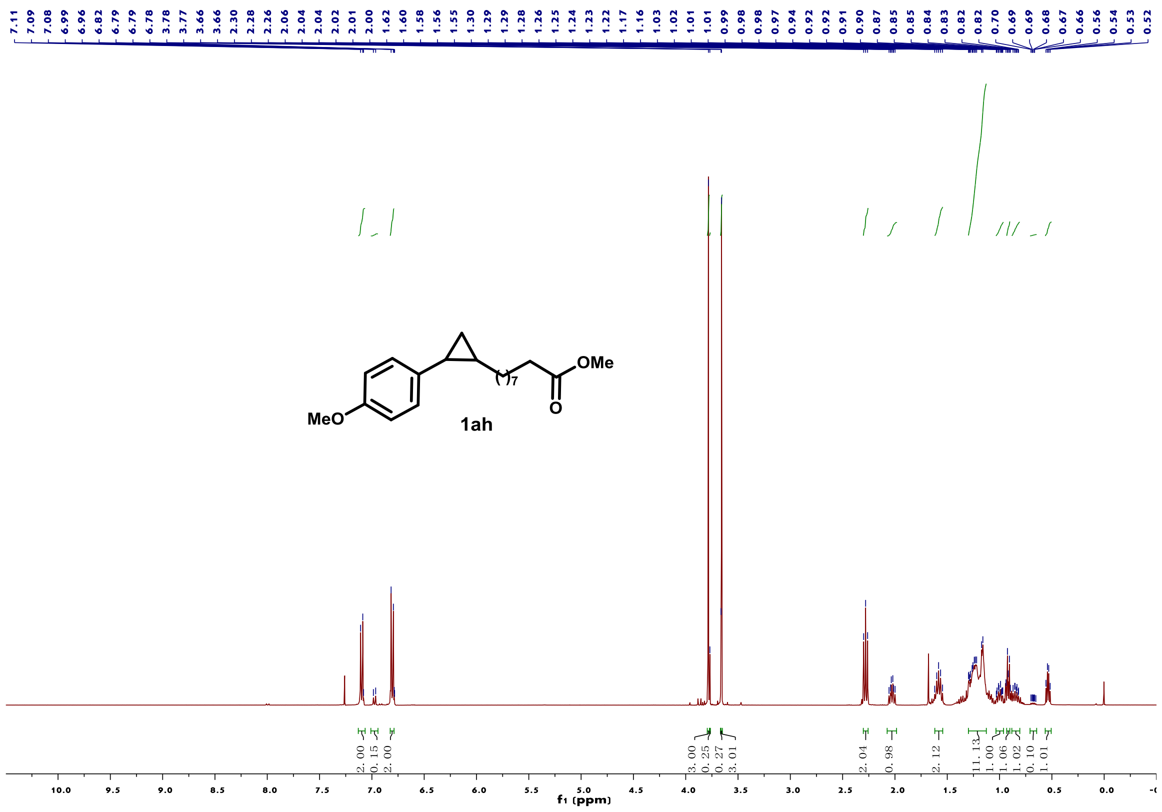

Supplementary Figure 78. ^{1}H NMR (400 MHz, $CDCl_3$) spectrum for 1ah

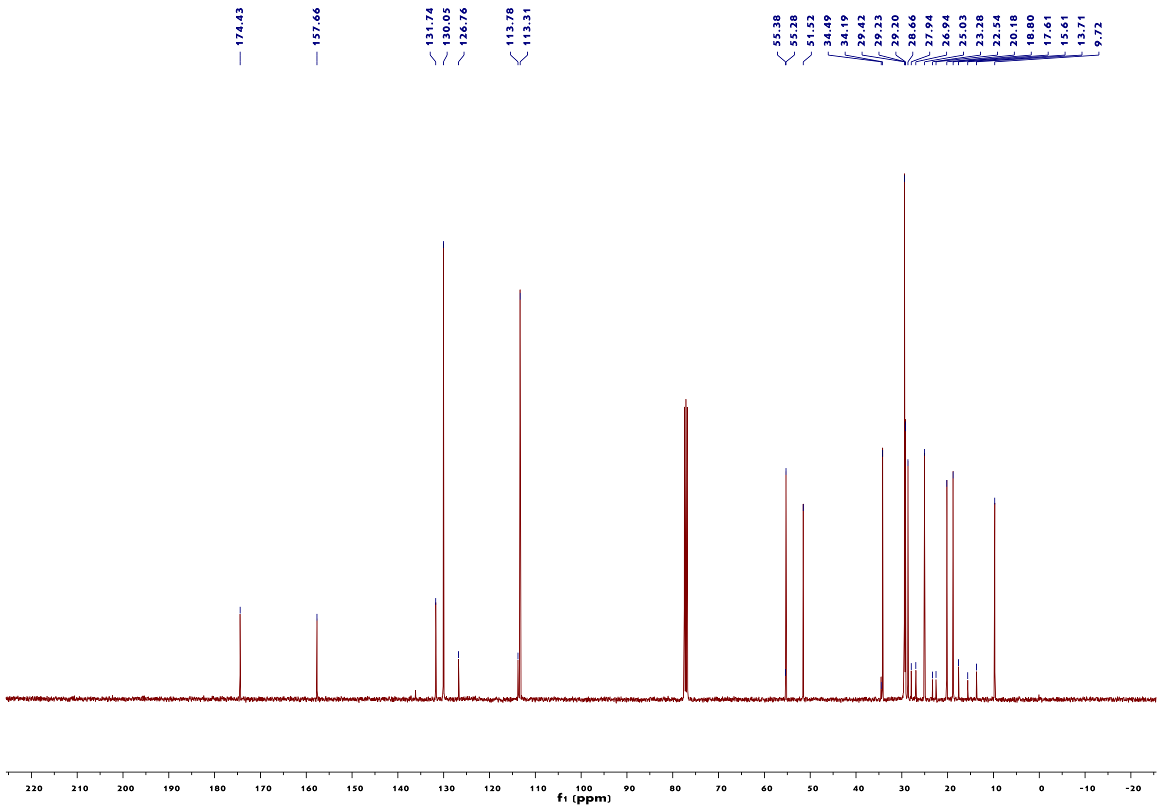

Supplementary Figure 79. ^{13}C NMR (100 MHz, $CDCl_3$) spectrum for 1ah

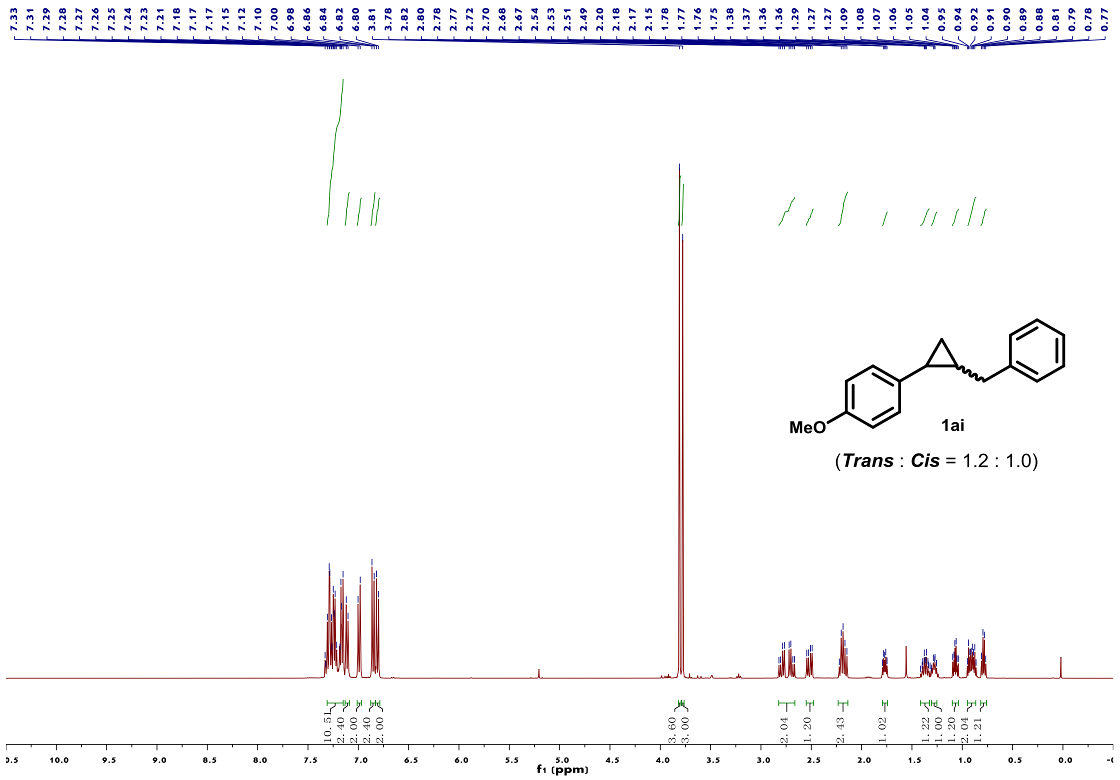

Supplementary Figure 80. ^{1}H **NMR (400 MHz,** $CDCl_3$**) spectrum for 1ai**

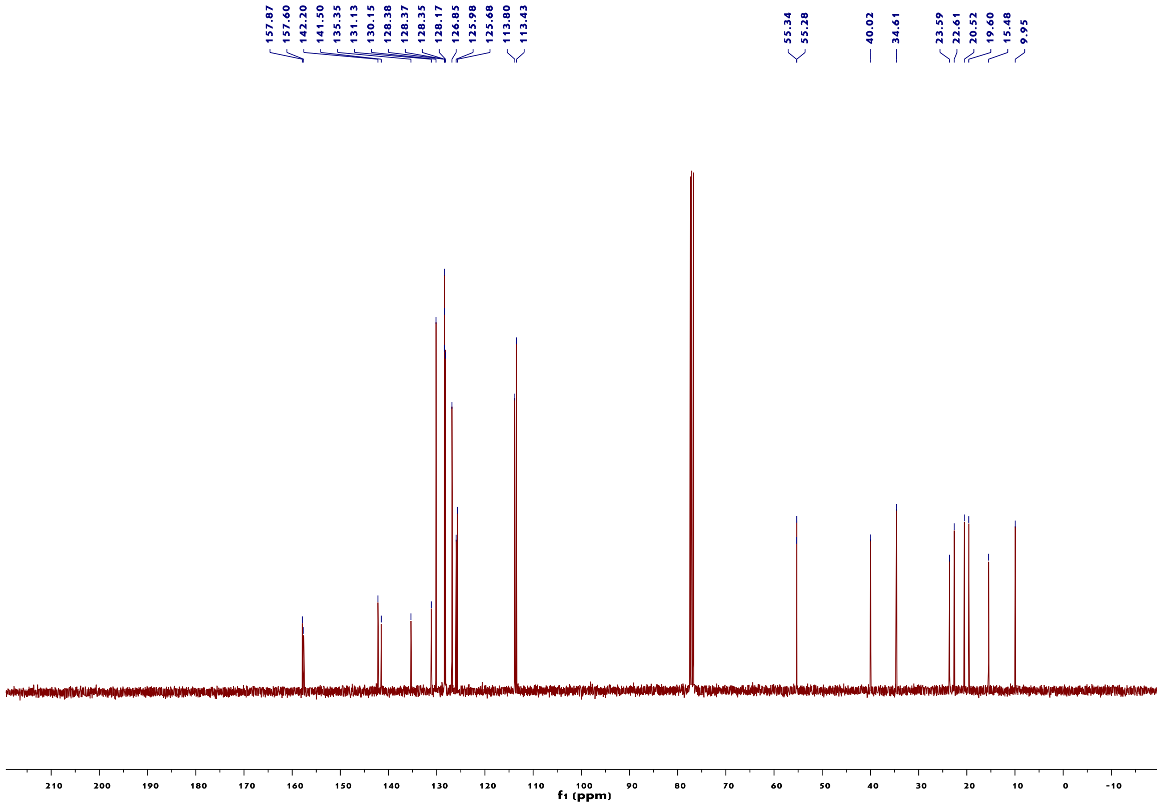

Supplementary Figure 81. ^{13}C **NMR (100 MHz,** $CDCl_3$**) spectrum for 1ai**

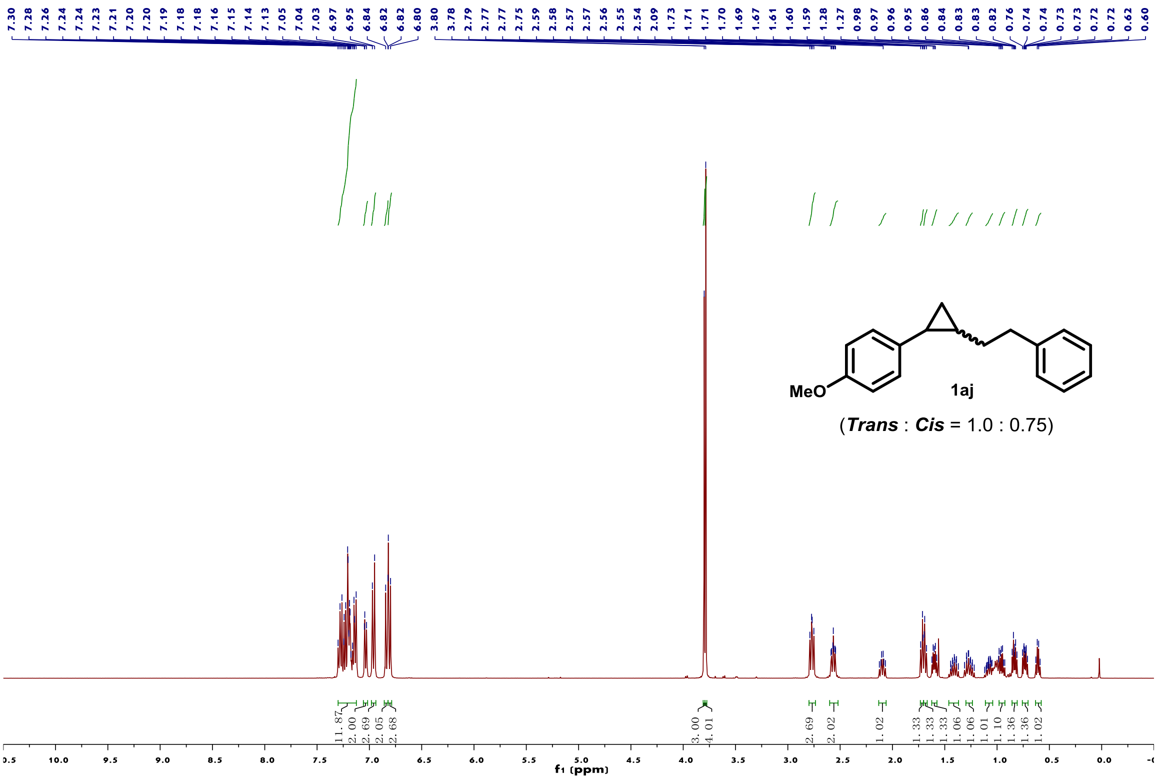

Supplementary Figure 82. ^{1}H NMR (400 MHz, $CDCl_3$) spectrum for 1aj

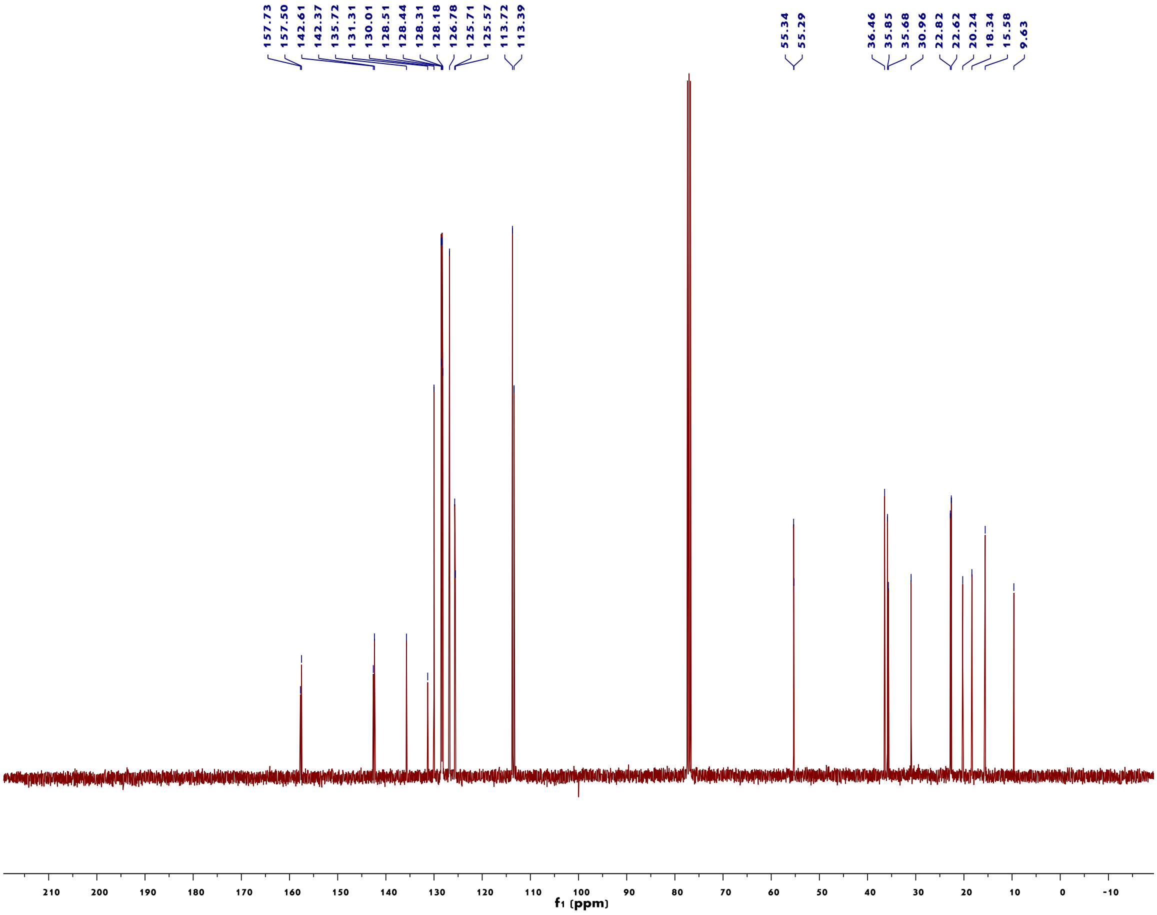

Supplementary Figure 83. ^{13}C NMR (100 MHz, $CDCl_3$) spectrum for 1aj

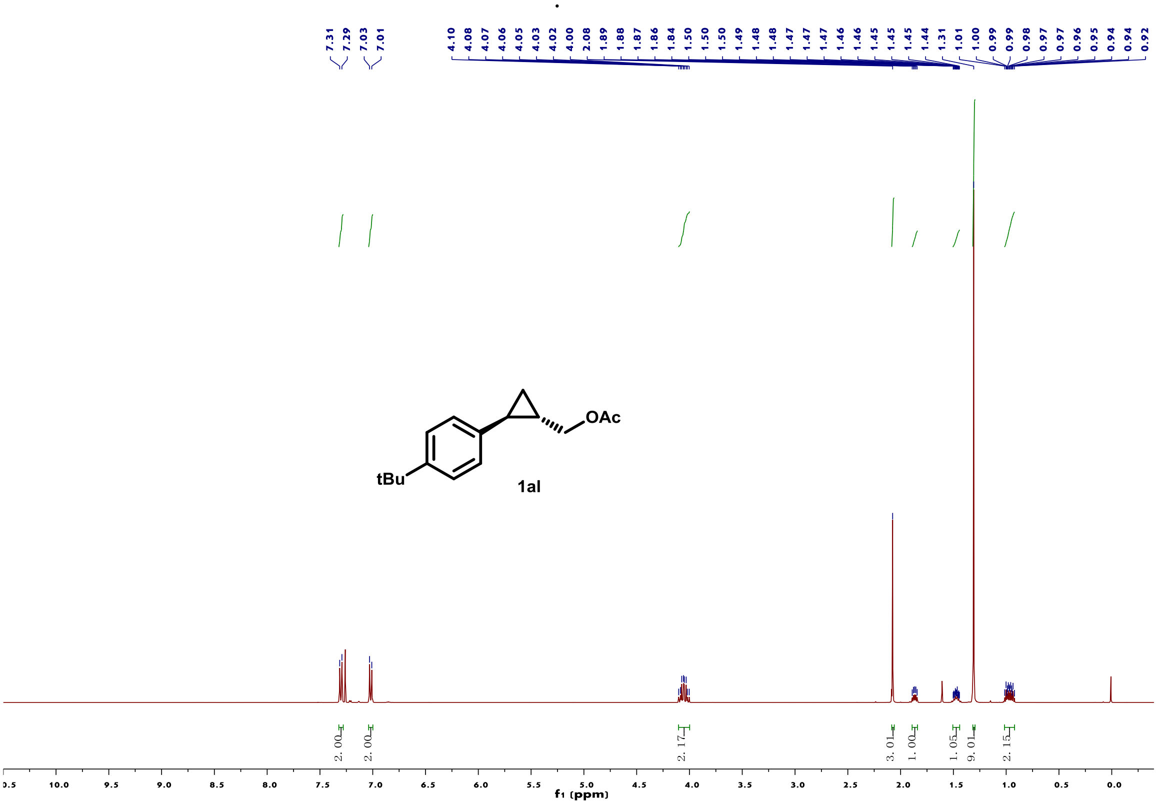

Supplementary Figure 84. ^{1}H **NMR (400 MHz,** $CDCl_3$**) spectrum for 1al**

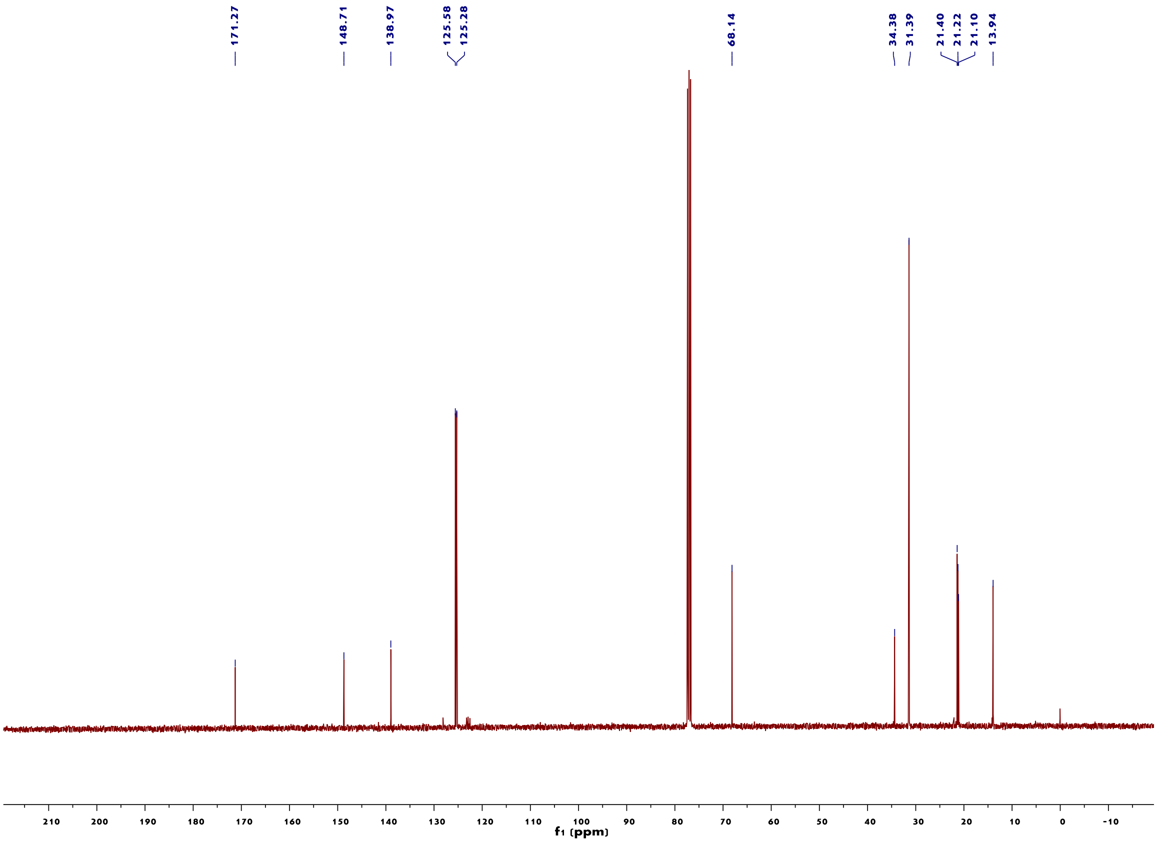

Supplementary Figure 85. ^{13}C **NMR (100 MHz,** $CDCl_3$**) spectrum for 1al**

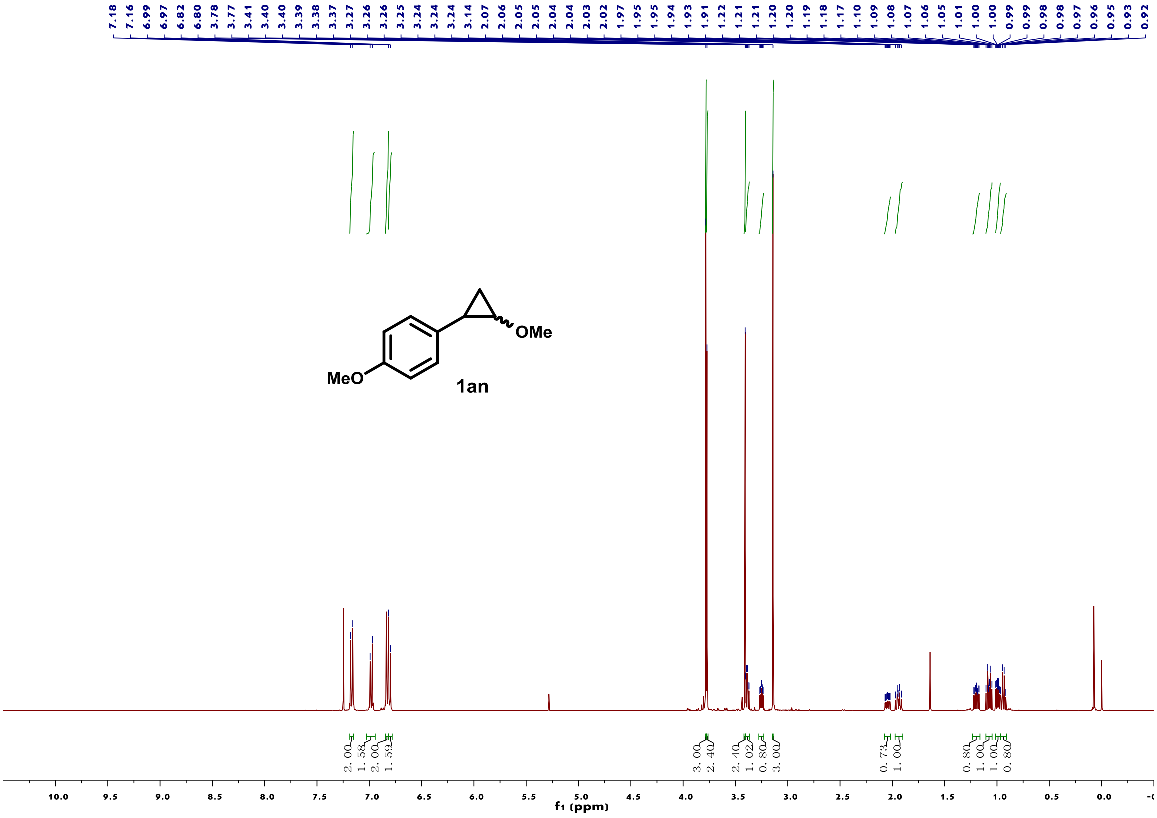

Supplementary Figure 86. ^{1}H NMR (400 MHz, $CDCl_3$) spectrum for 1an

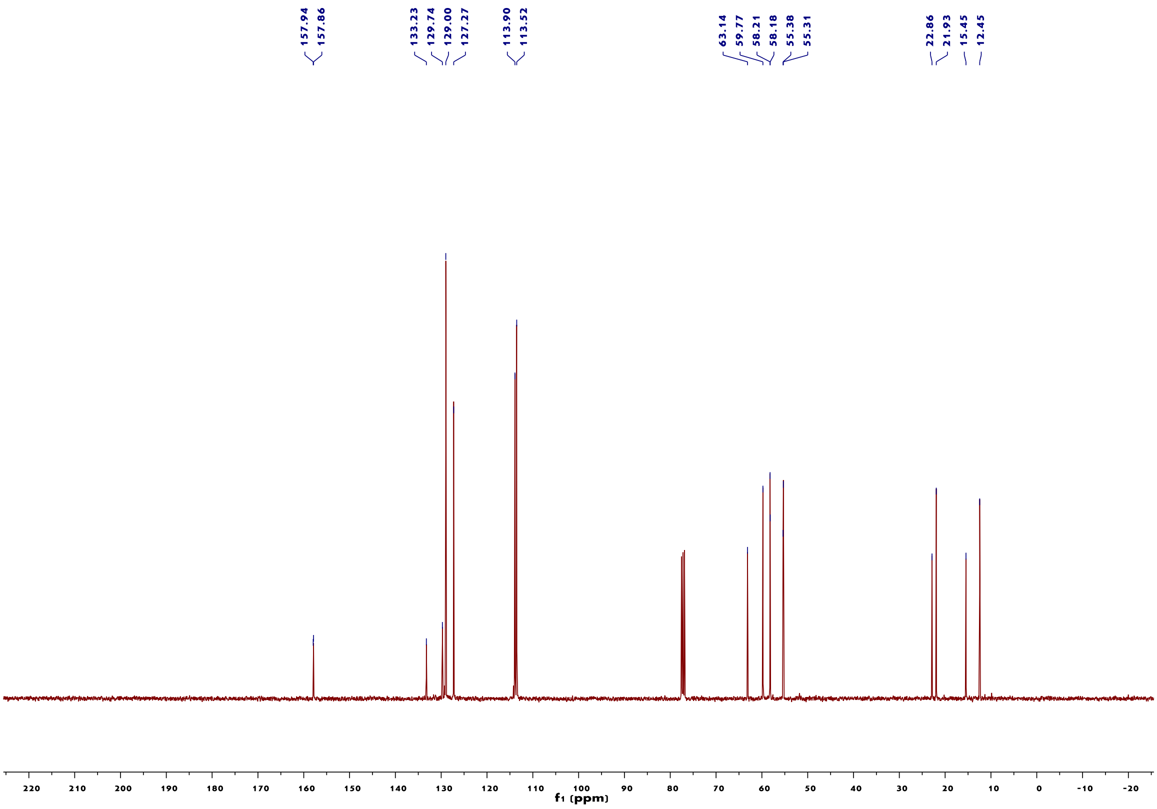

Supplementary Figure 87. ^{13}C NMR (100 MHz, $CDCl_3$) spectrum for 1an

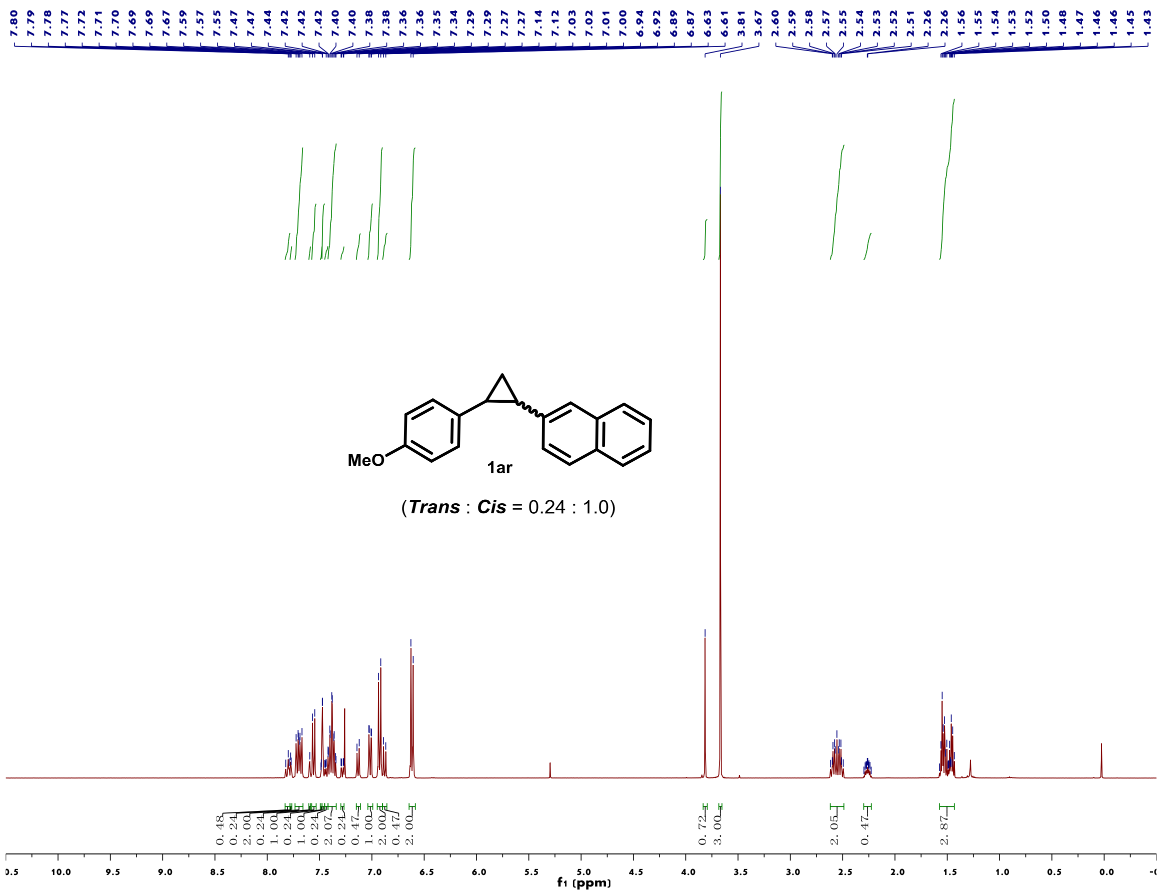

Supplementary Figure 88. ^{1}H NMR (400 MHz, $CDCl_3$) spectrum for 1ar

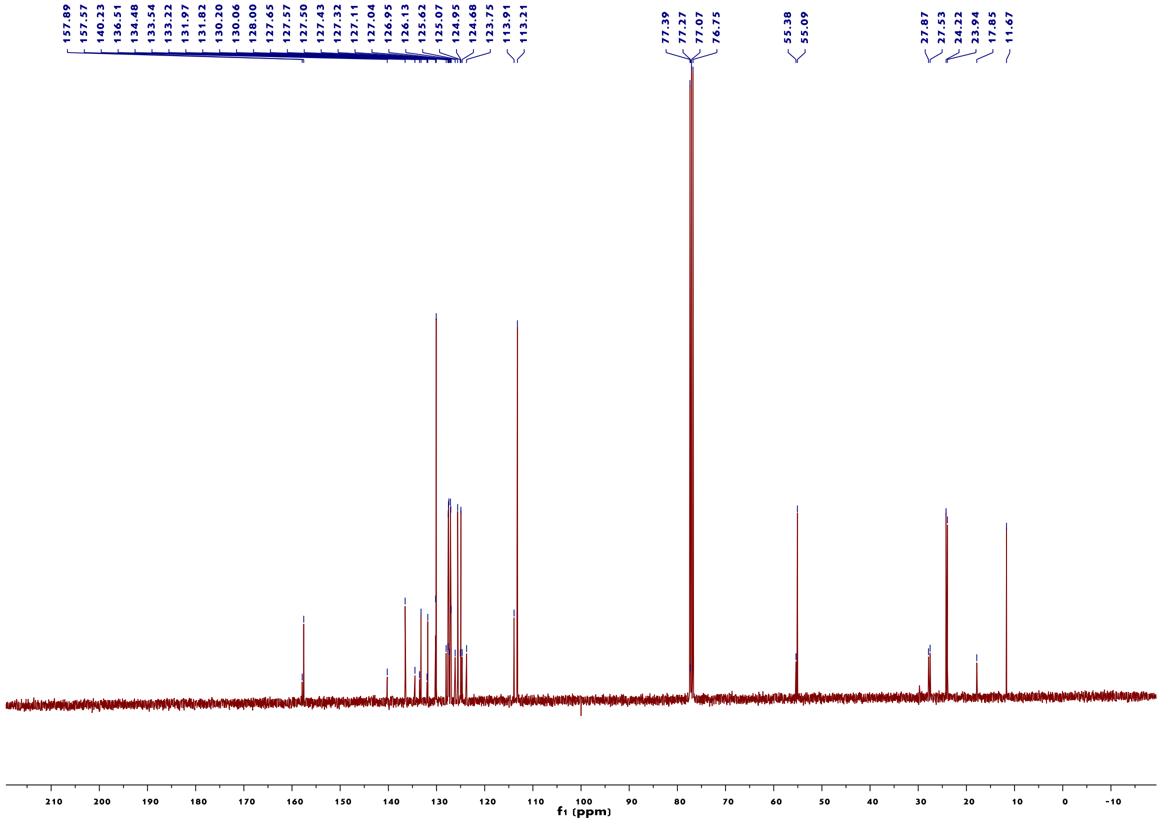

Supplementary Figure 89. ^{13}C NMR (100 MHz, $CDCl_3$) spectrum for 1ar

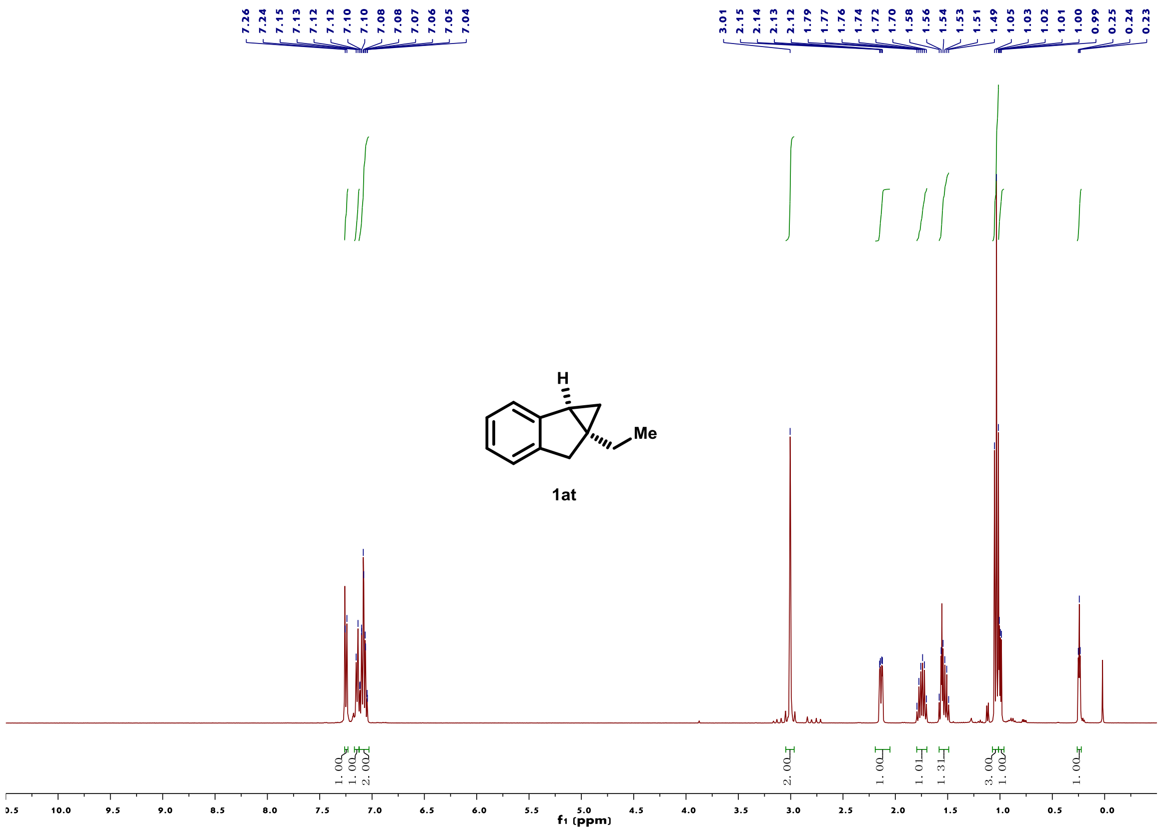

Supplementary Figure 90. ^{1}H NMR (400 MHz, CDCl$_3$) spectrum for 1at

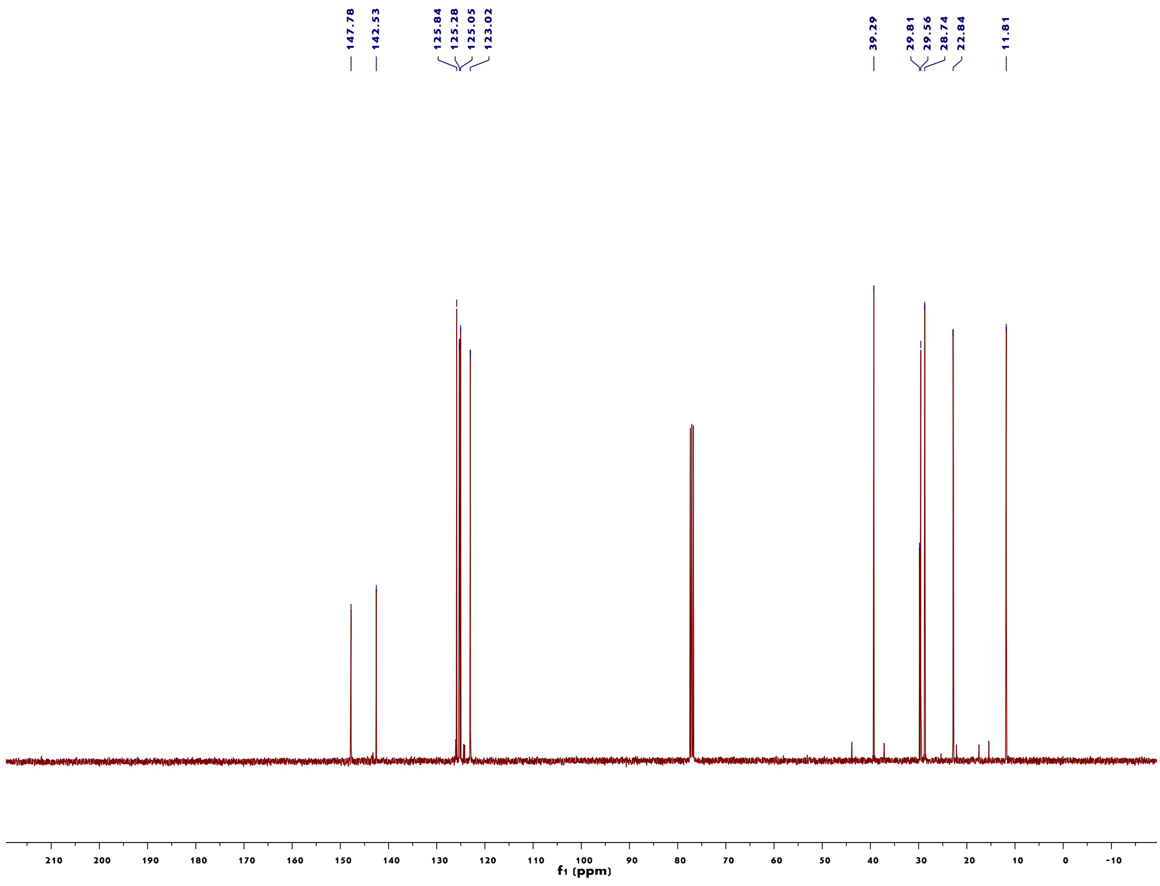

Supplementary Figure 91. ^{13}C NMR (100 MHz, CDCl$_3$) spectrum for 1at

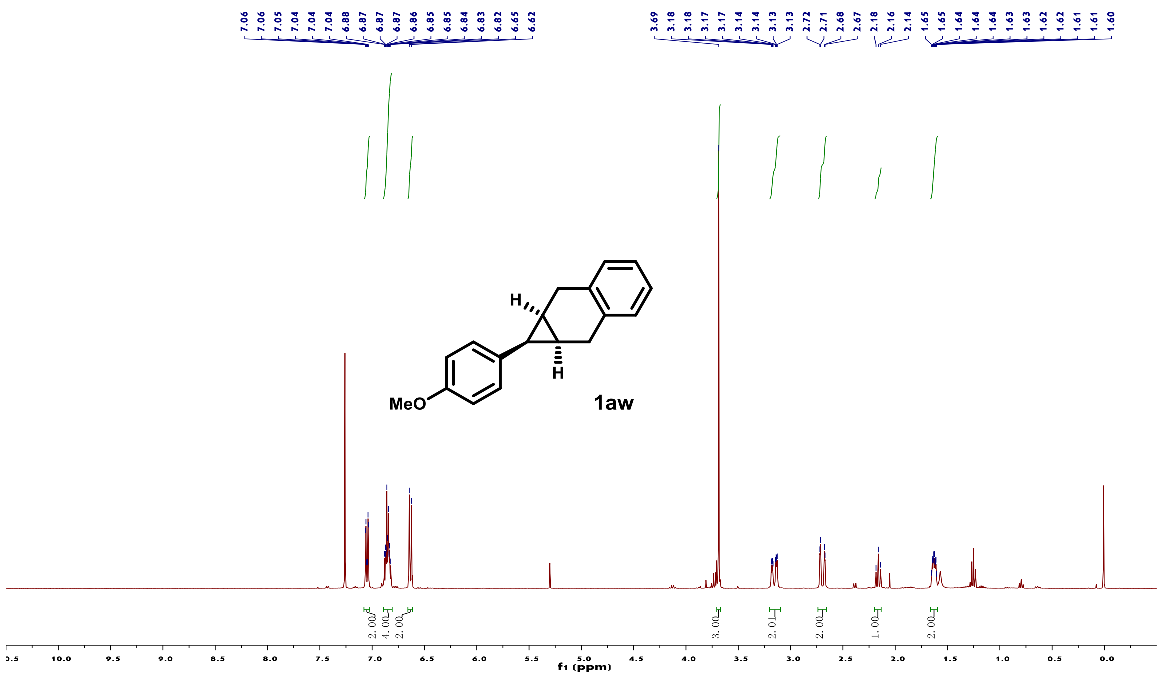

Supplementary Figure 92. ^{1}**H NMR (400 MHz, CDCl$_3$) spectrum for 1aw**

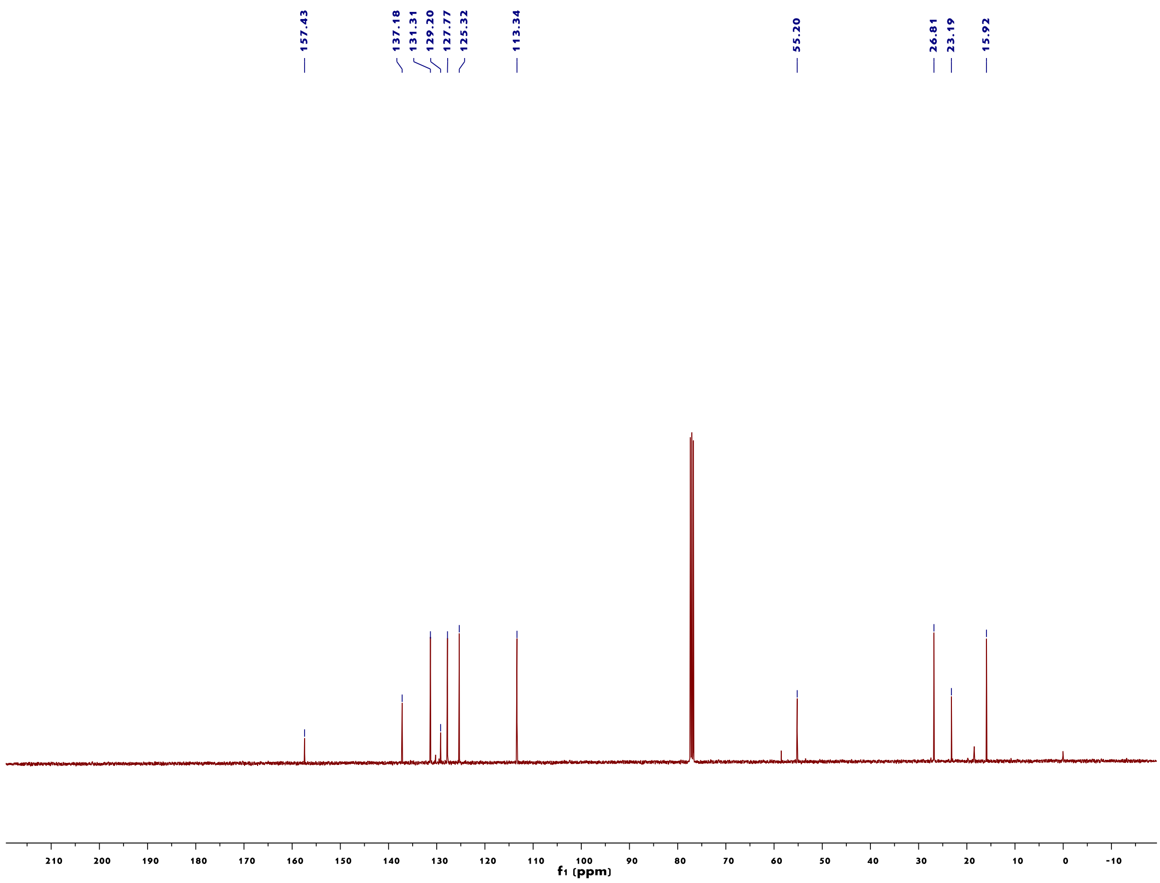

Supplementary Figure 93. ^{13}**C NMR (100 MHz, CDCl$_3$) spectrum for 1aw**

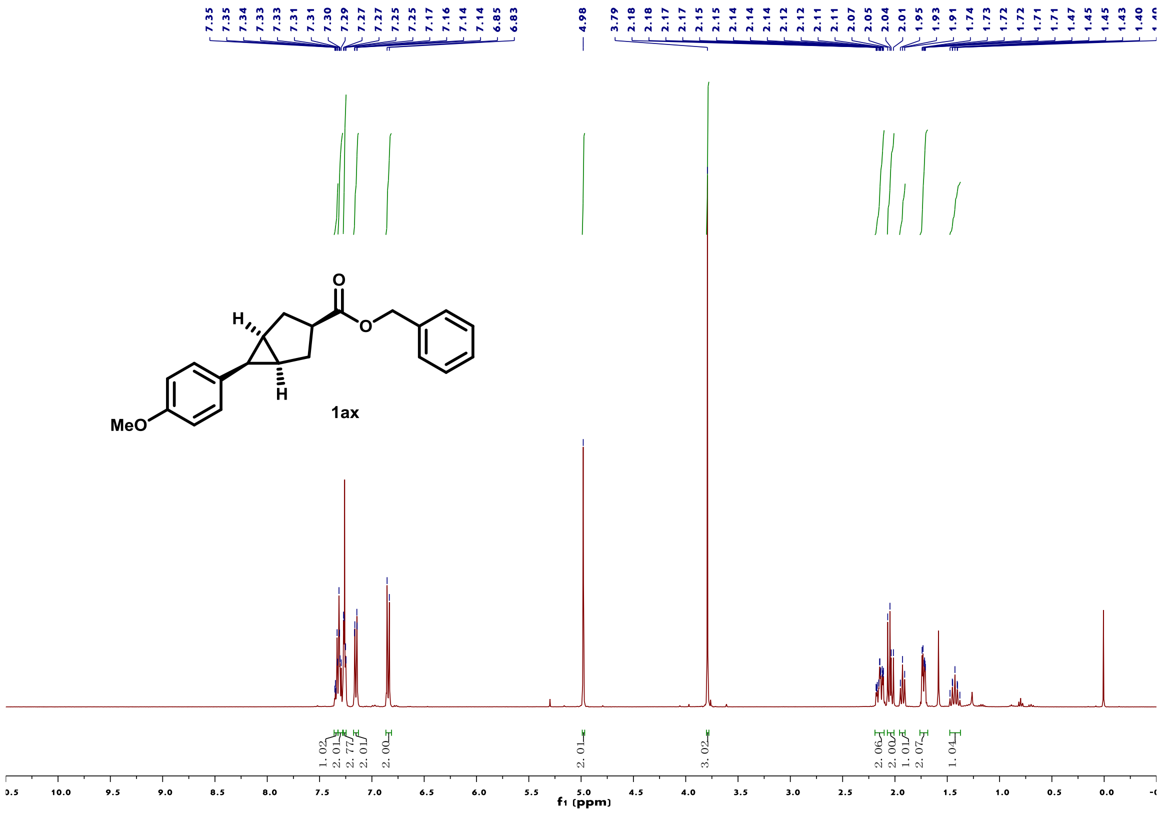

Supplementary Figure 94. **^{1}H NMR (400 MHz, $CDCl_3$) spectrum for 1ax**

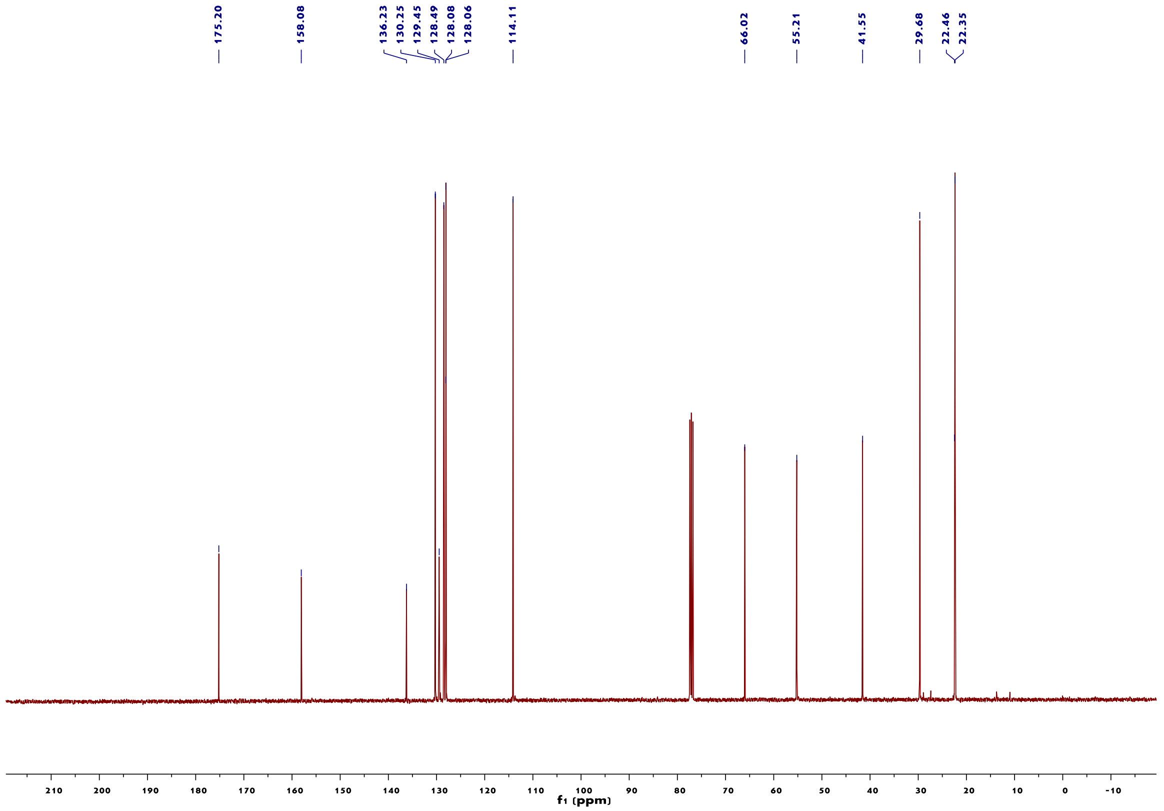

Supplementary Figure 95. **^{13}C NMR (100 MHz, $CDCl_3$) spectrum for 1ax**

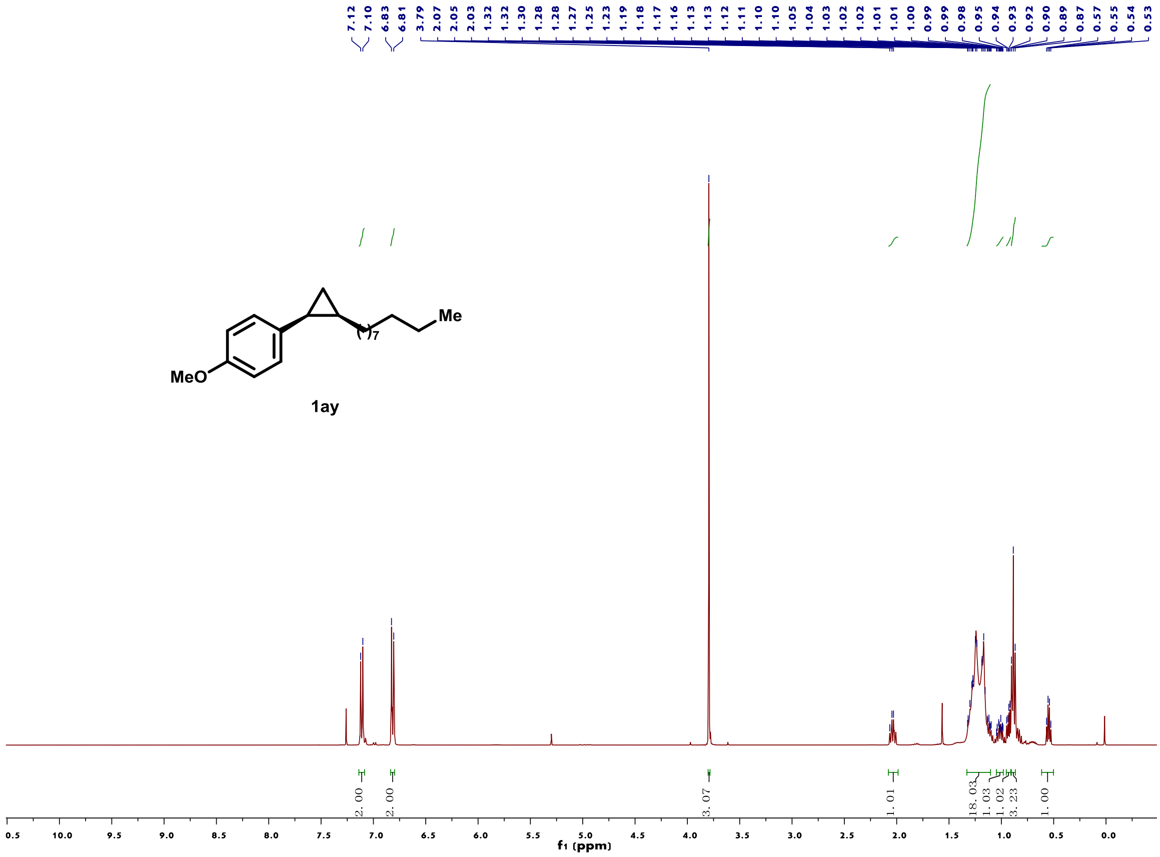

Supplementary Figure 96. ^{1}H NMR (400 MHz, $CDCl_3$) spectrum for 1ay

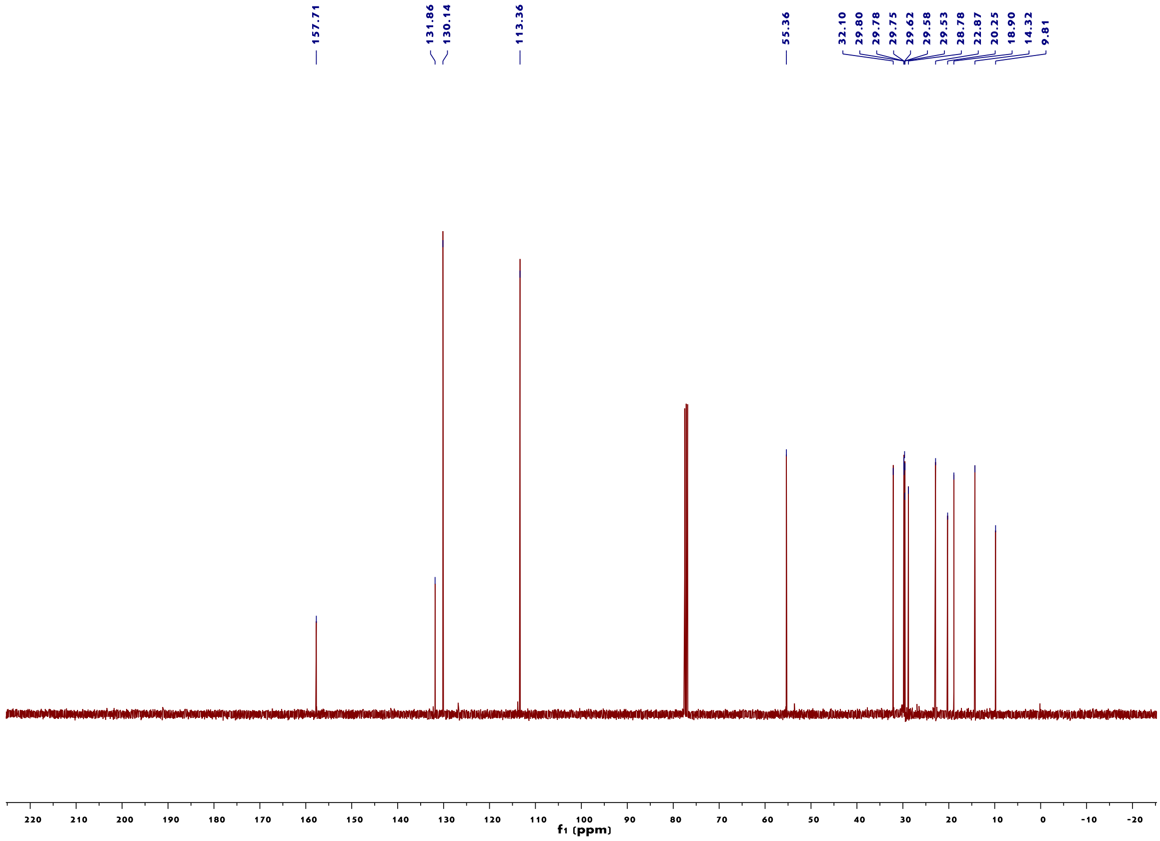

Supplementary Figure 97. ^{13}C NMR (100 MHz, $CDCl_3$) spectrum for 1ay

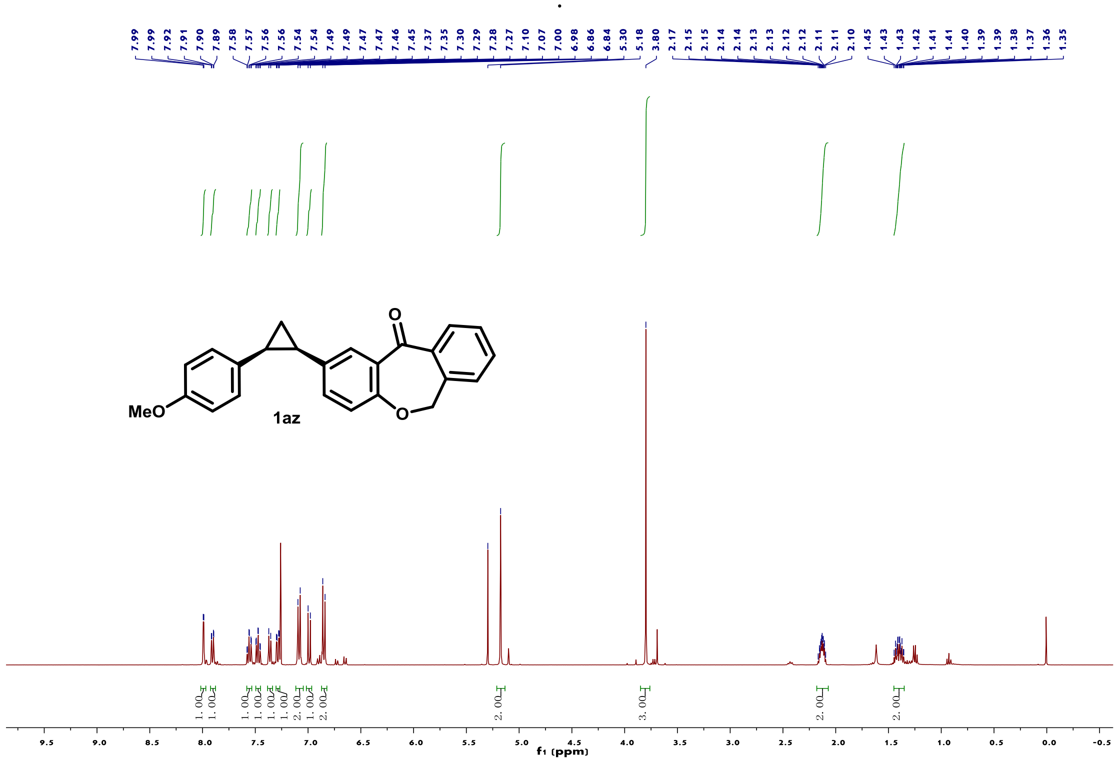

Supplementary Figure 98. ^{1}H NMR (400 MHz, $CDCl_3$) spectrum for 1az

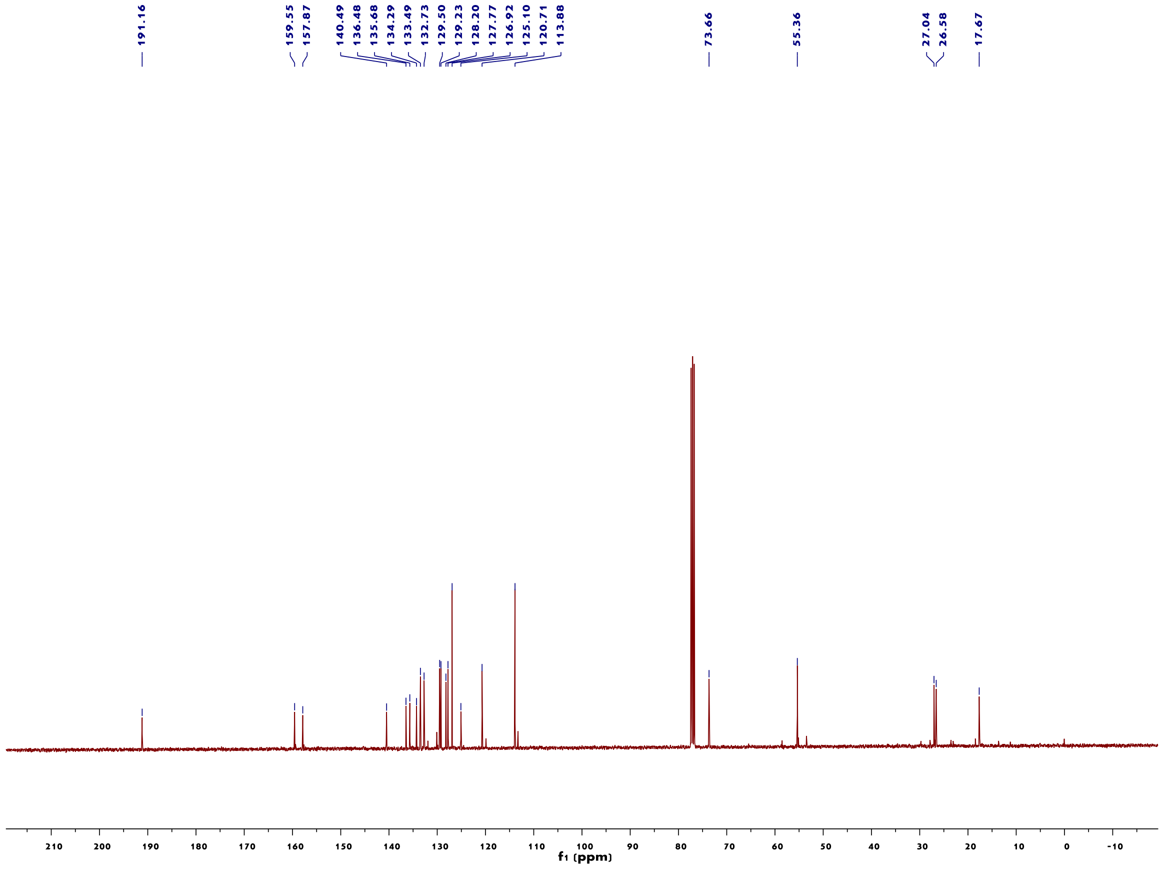

Supplementary Figure 99. ^{13}C NMR (100 MHz, $CDCl_3$) spectrum for 1az

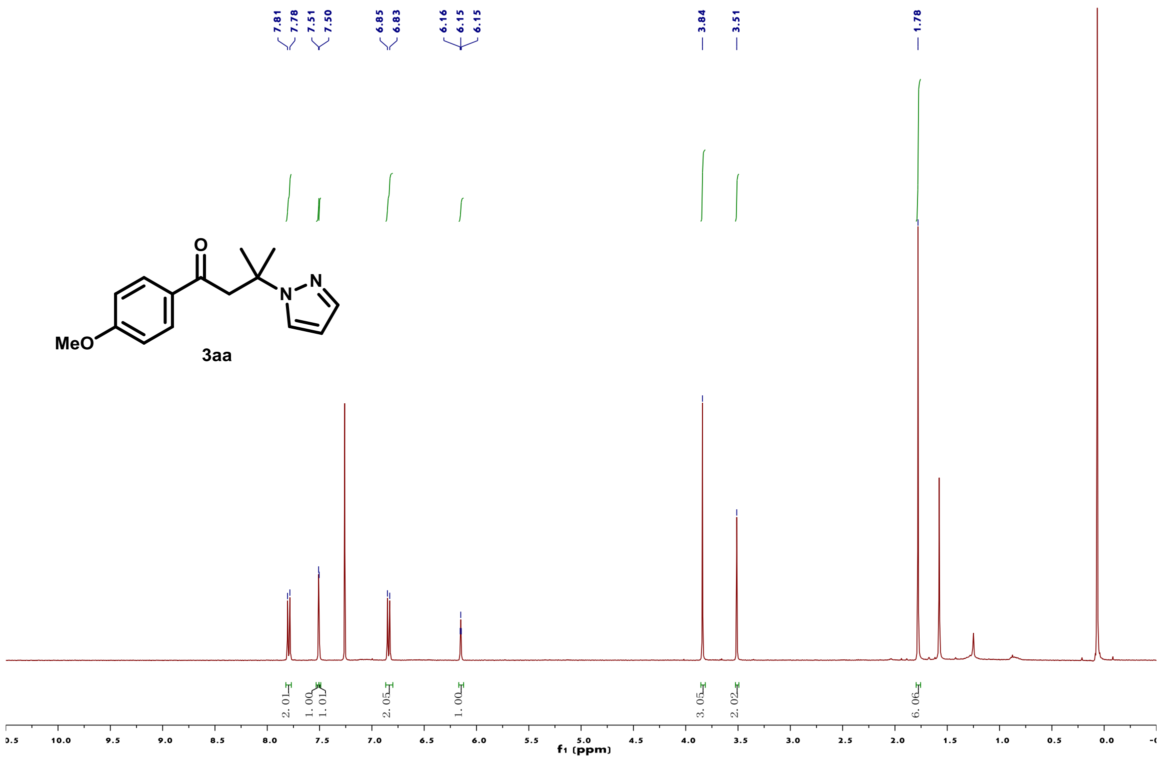

Supplementary Figure 100. ^{1}H **NMR (400 MHz,** $CDCl_3$**) spectrum for 3aa**

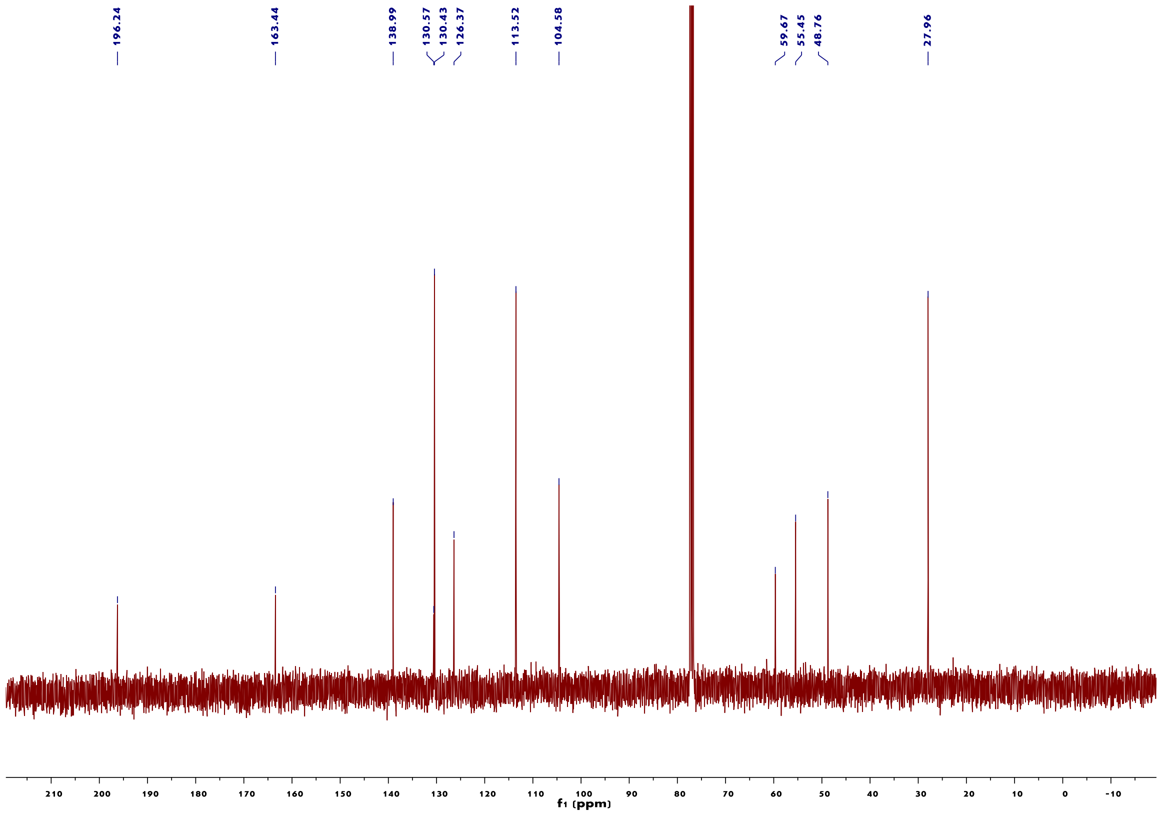

Supplementary Figure 101. ^{13}C **NMR (100 MHz,** $CDCl_3$**) spectrum for 3aa**

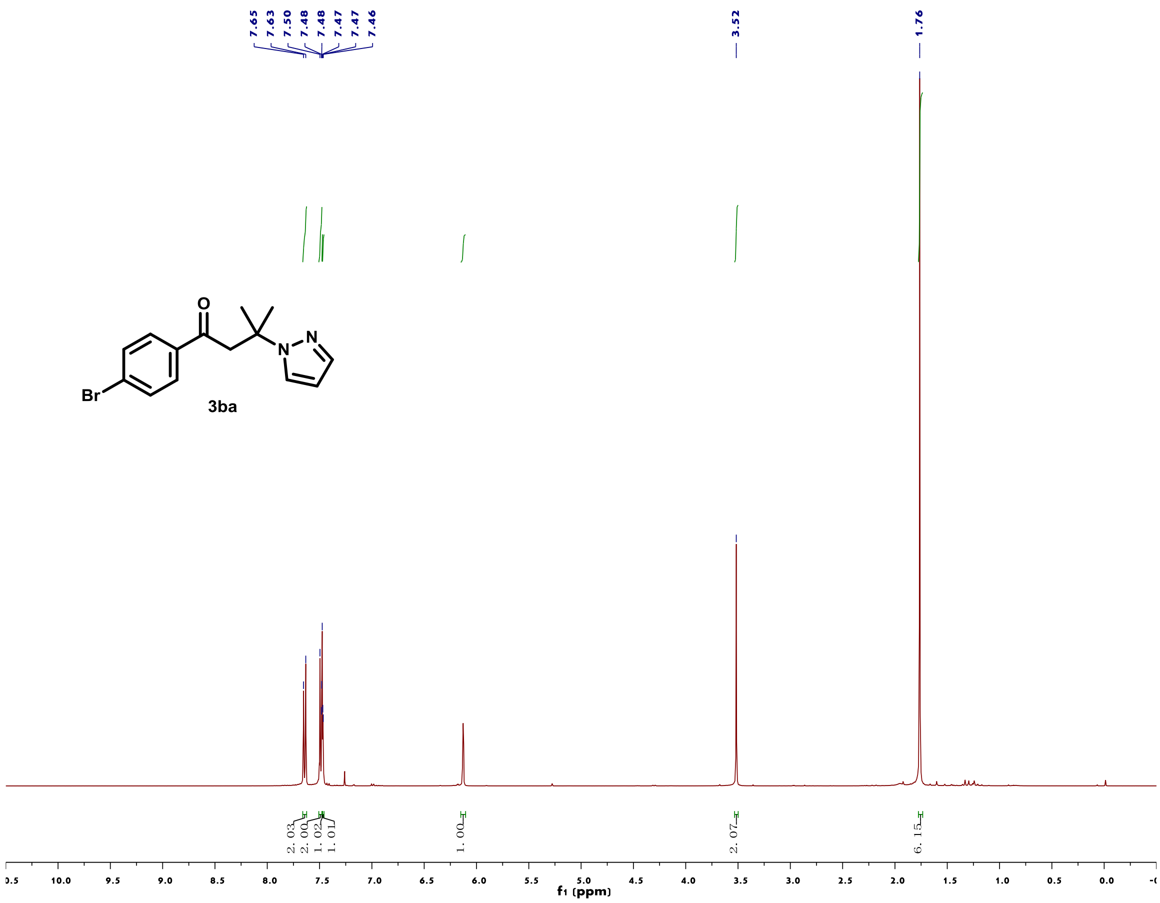

Supplementary Figure 102. ^{1}H NMR (400 MHz, $CDCl_3$) spectrum for 3ba

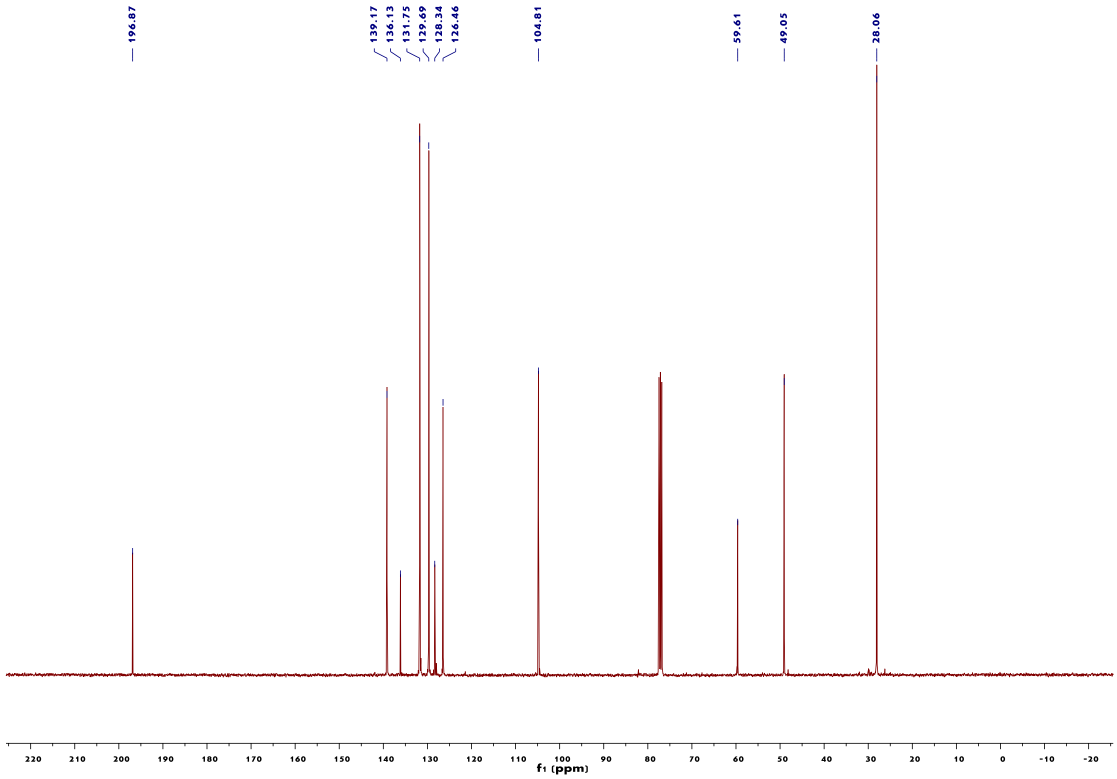

Supplementary Figure 103. ^{13}C NMR (100 MHz, $CDCl_3$) spectrum for 3ba

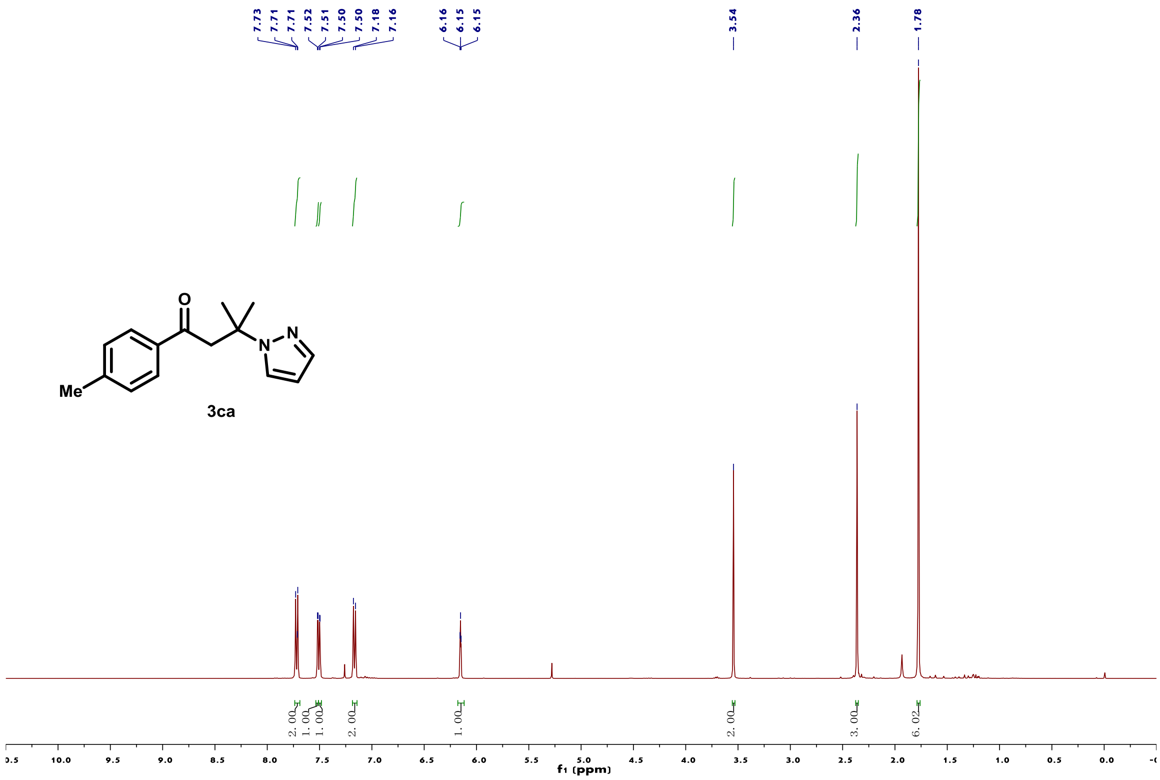

Supplementary Figure 104. ^{1}H NMR (400 MHz, $CDCl_3$) spectrum for 3ca

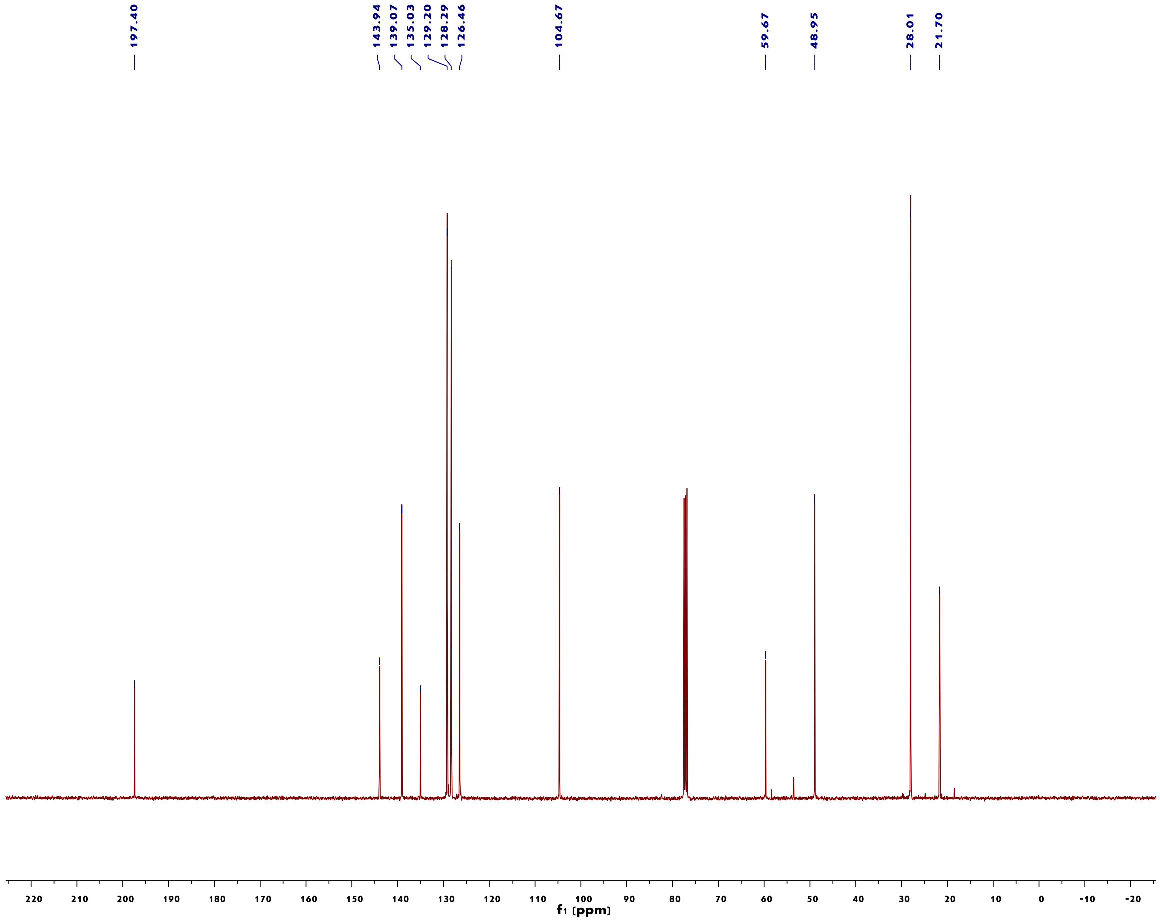

Supplementary Figure 105. ^{13}C NMR (100 MHz, $CDCl_3$) spectrum for 3ca

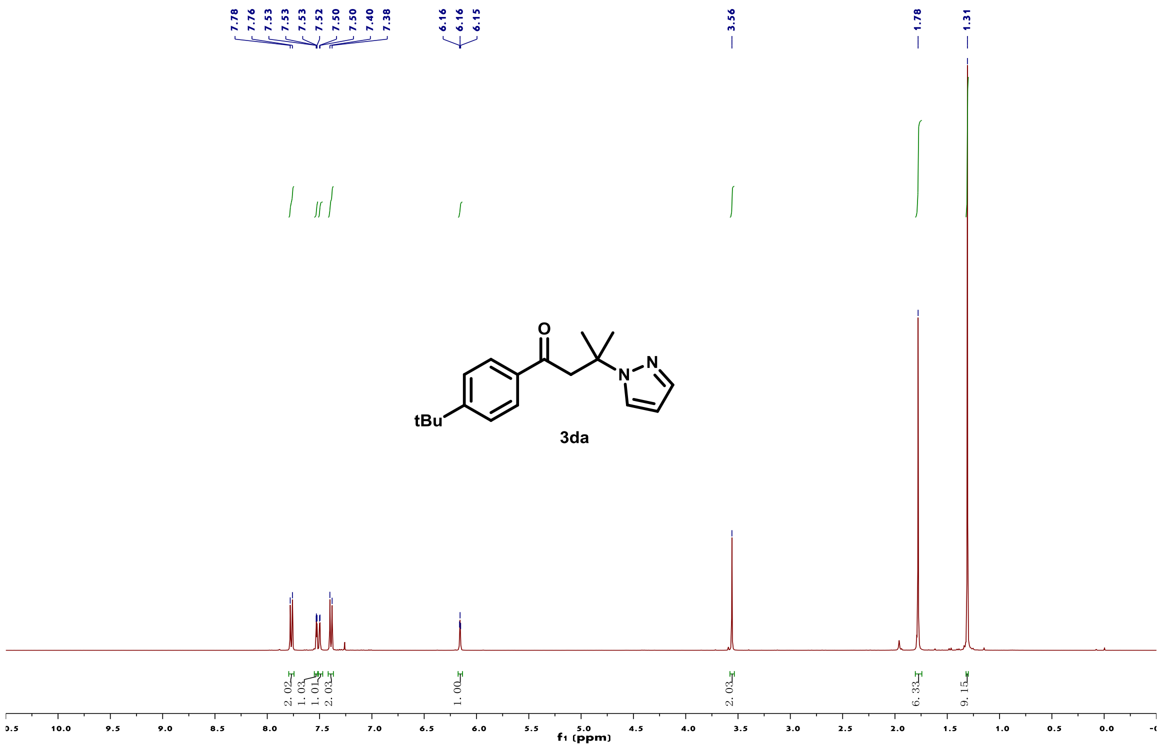

Supplementary Figure 106. ^{1}H NMR (400 MHz, $CDCl_3$) spectrum for 3da

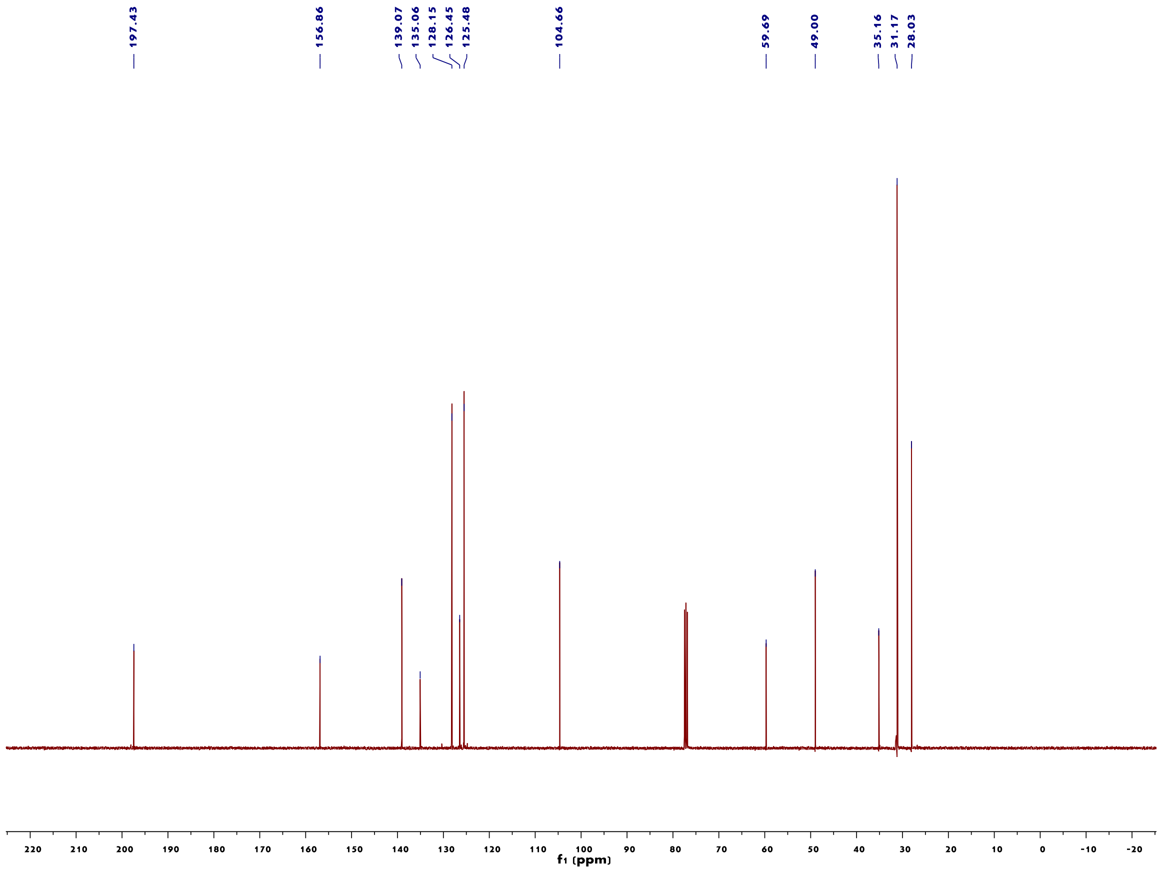

Supplementary Figure 107. ^{13}C NMR (100 MHz, $CDCl_3$) spectrum for 3da

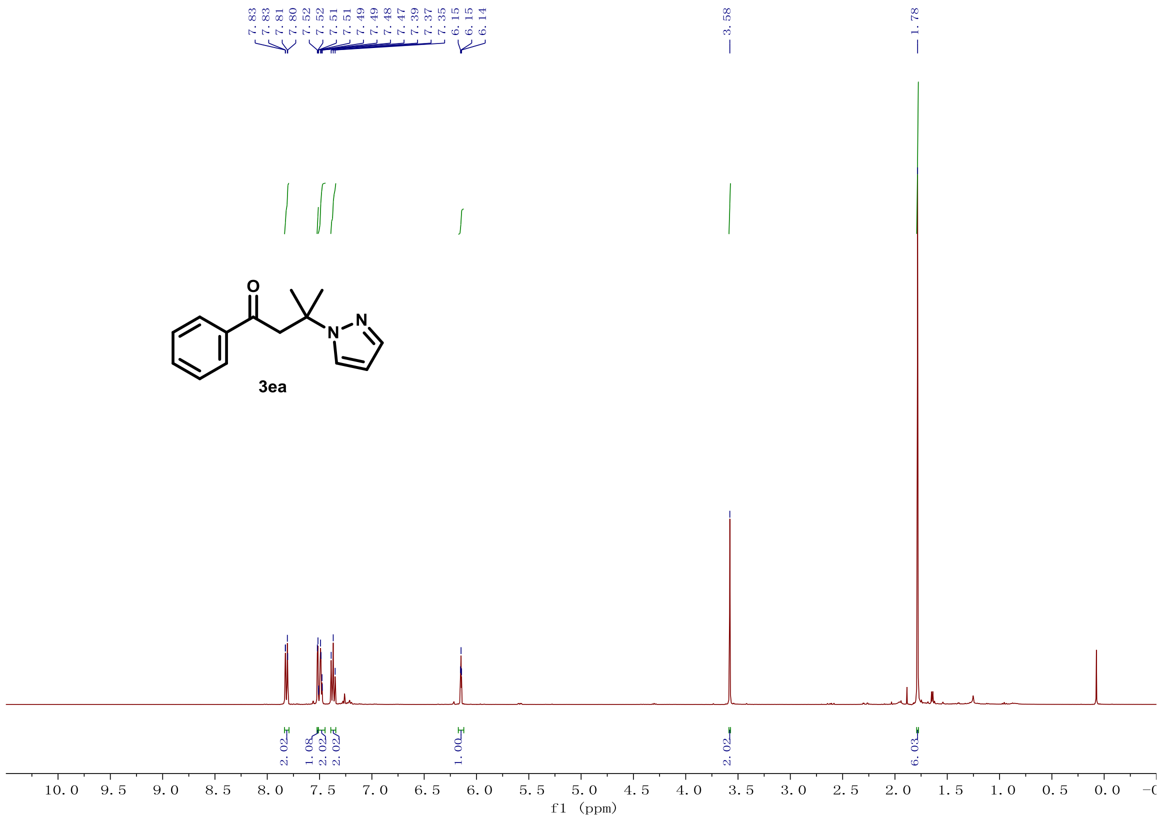

Supplementary Figure 108. **^{1}H NMR (400 MHz, $CDCl_3$) spectrum for 3ea**

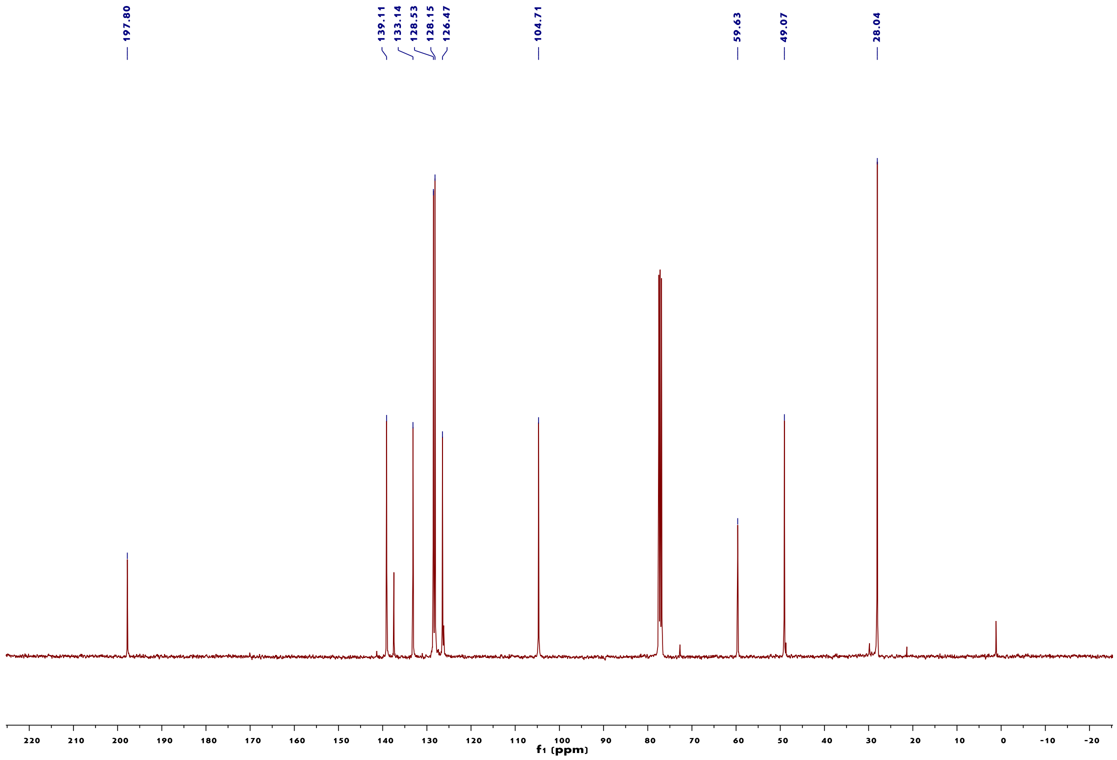

Supplementary Figure 109. **^{13}C NMR (100 MHz, $CDCl_3$) spectrum for 3ea**

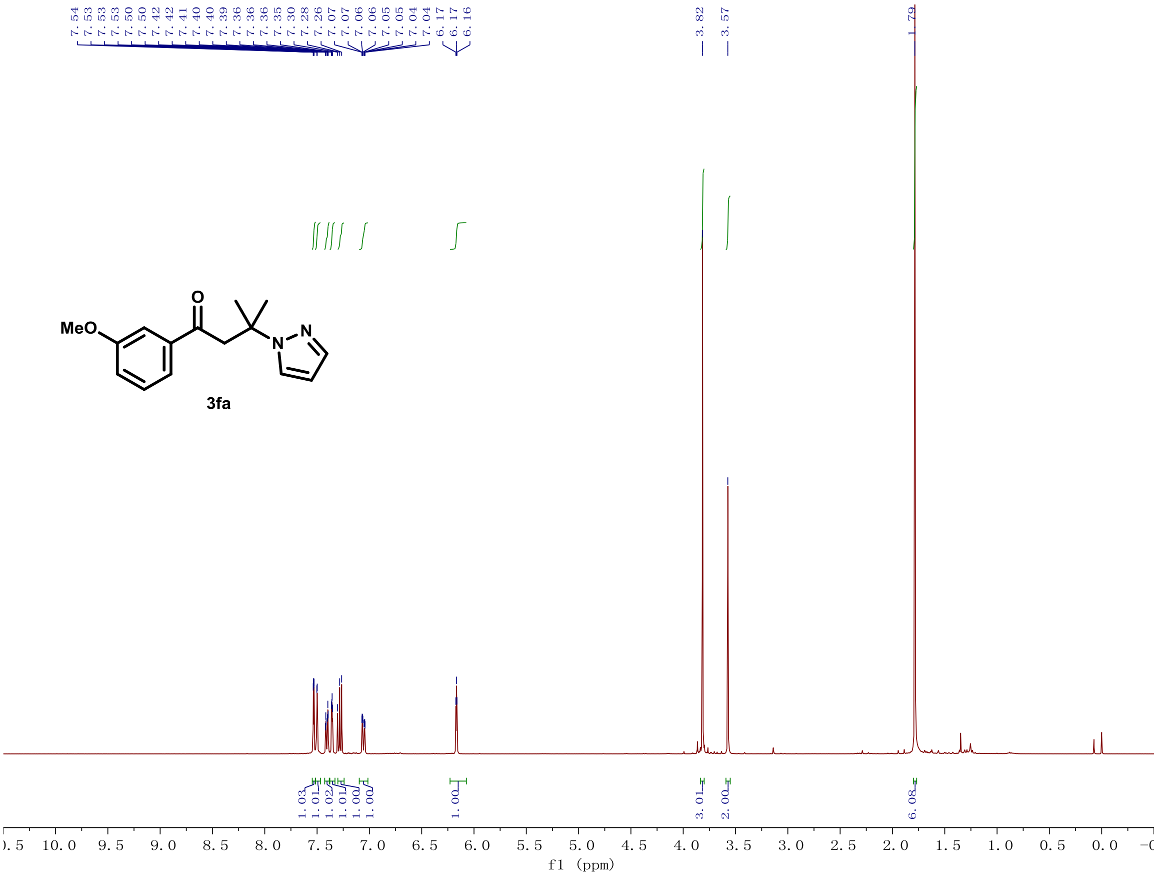

Supplementary Figure 110. ^{1}H NMR (400 MHz, $CDCl_3$) spectrum for 3fa

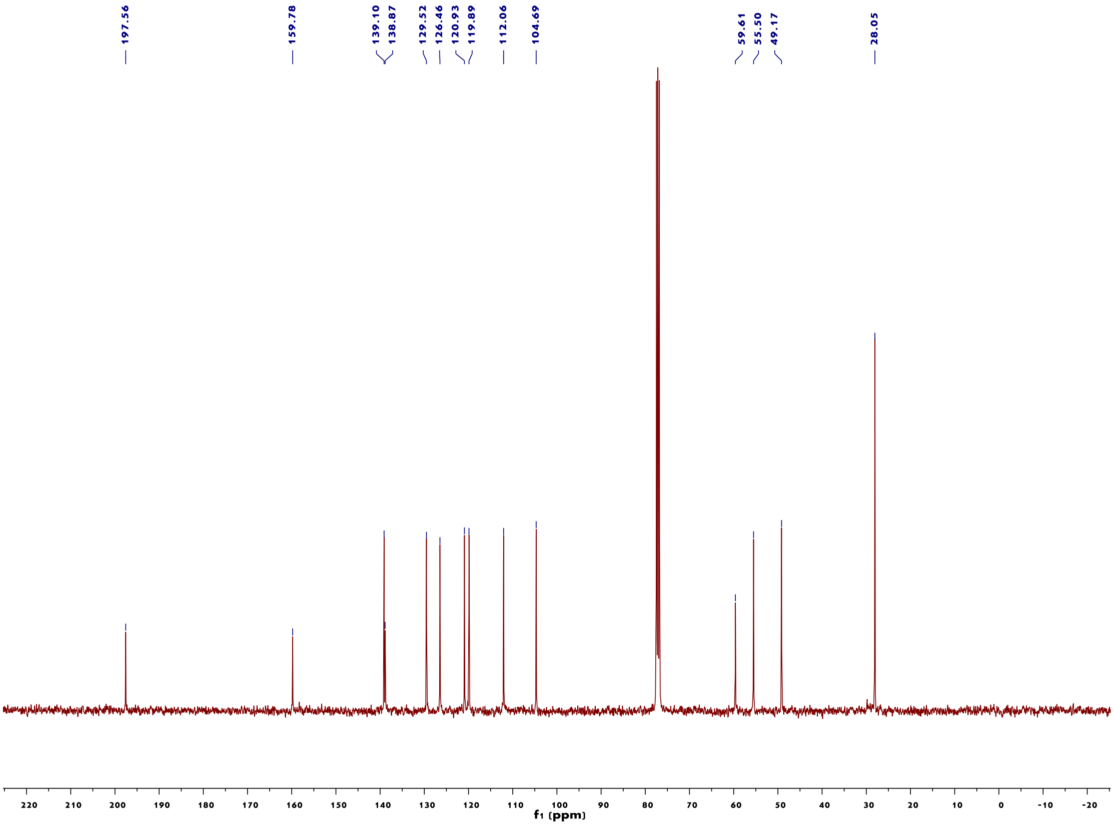

Supplementary Figure 111. ^{13}C NMR (100 MHz, $CDCl_3$) spectrum for 3fa

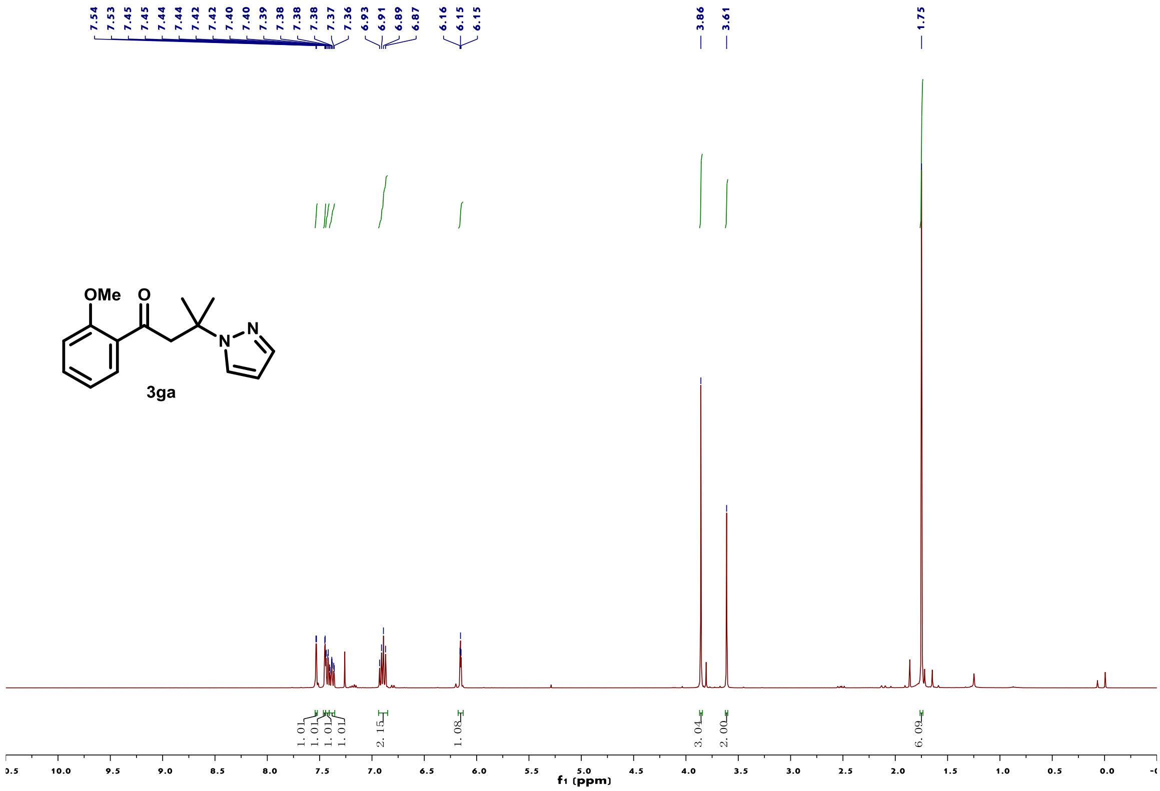

Supplementary Figure 112. ^{1}H NMR (400 MHz, $CDCl_3$) spectrum for 3ga

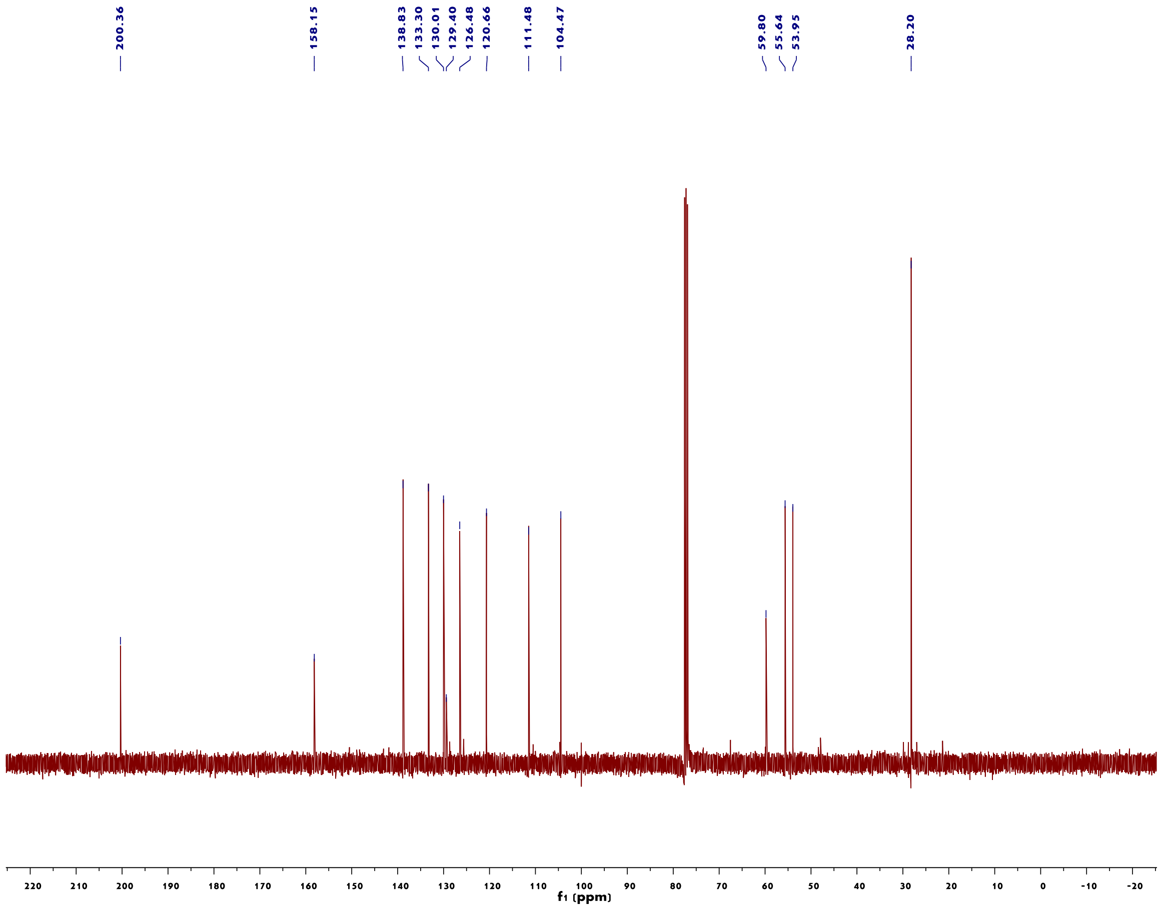

Supplementary Figure 113. ^{13}C NMR (100 MHz, $CDCl_3$) spectrum for 3ga

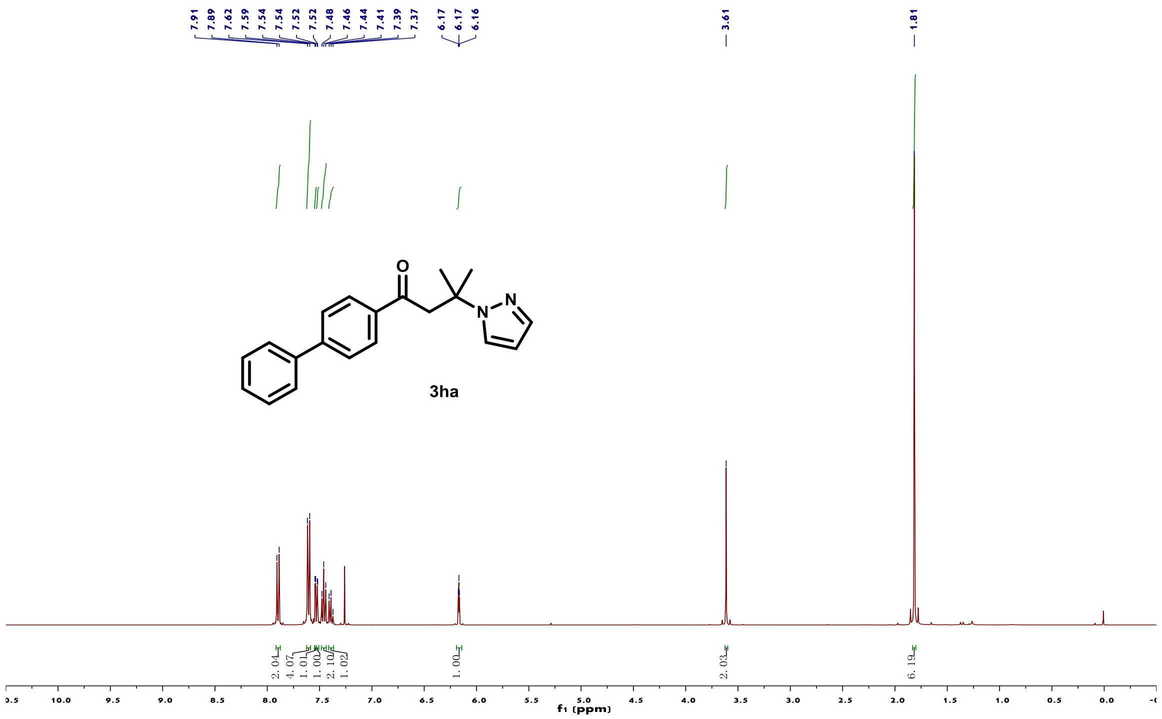

Supplementary Figure 114. ^{1}H NMR (400 MHz, $CDCl_3$) spectrum for 3ha

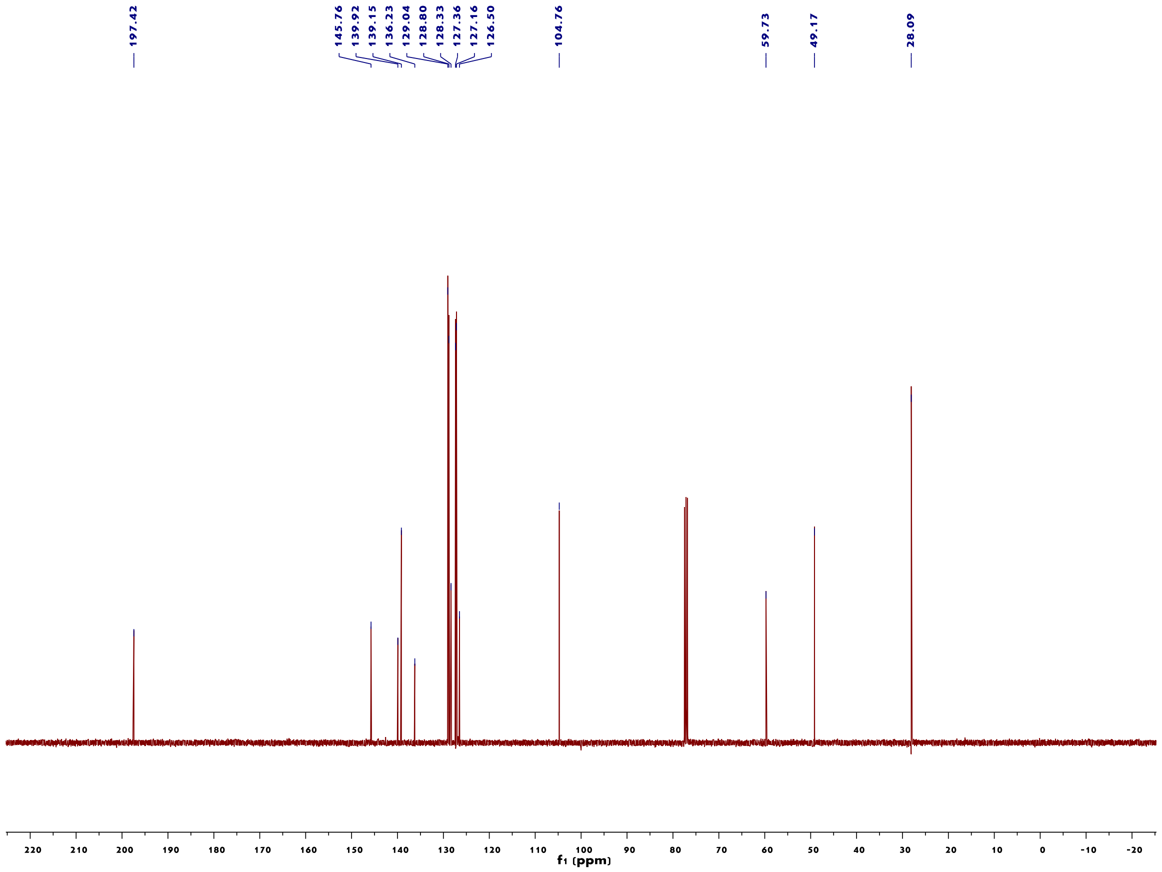

Supplementary Figure 115. ^{13}C NMR (100 MHz, $CDCl_3$) spectrum for 3ha

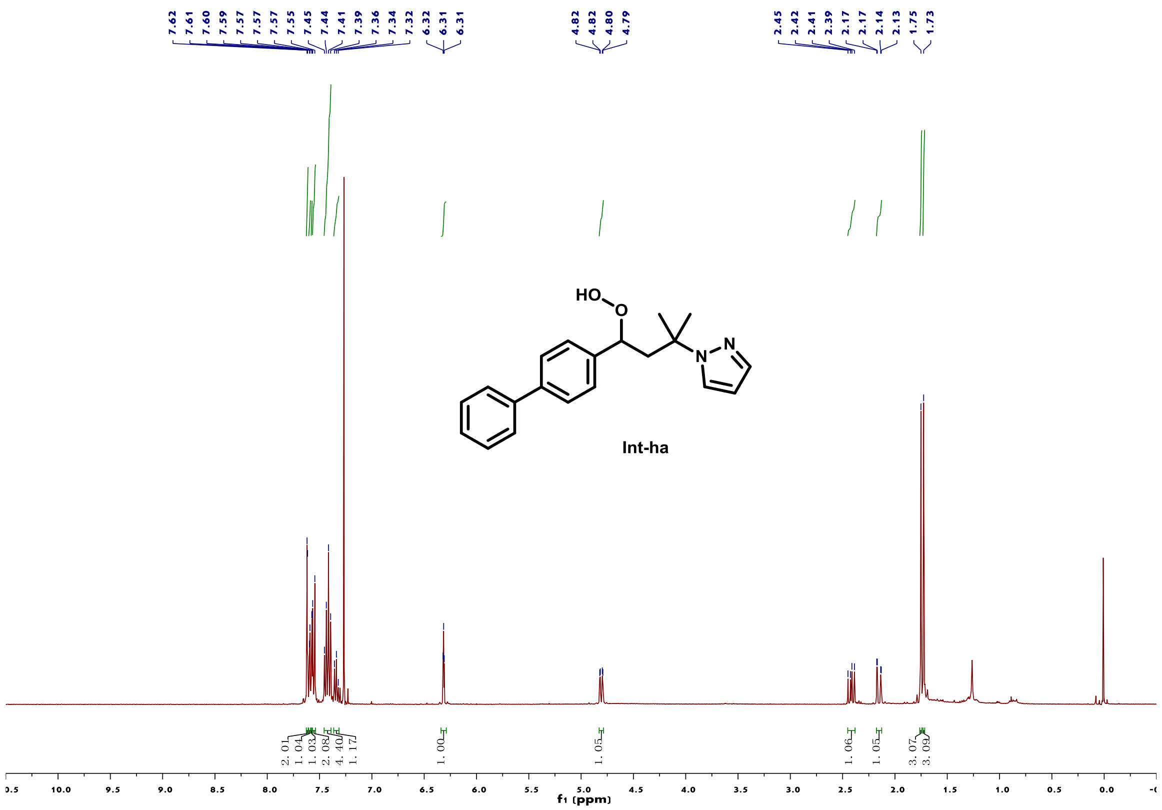

Supplementary Figure 116. ^{1}H NMR (400 MHz, $CDCl_3$) spectrum for Int-ha

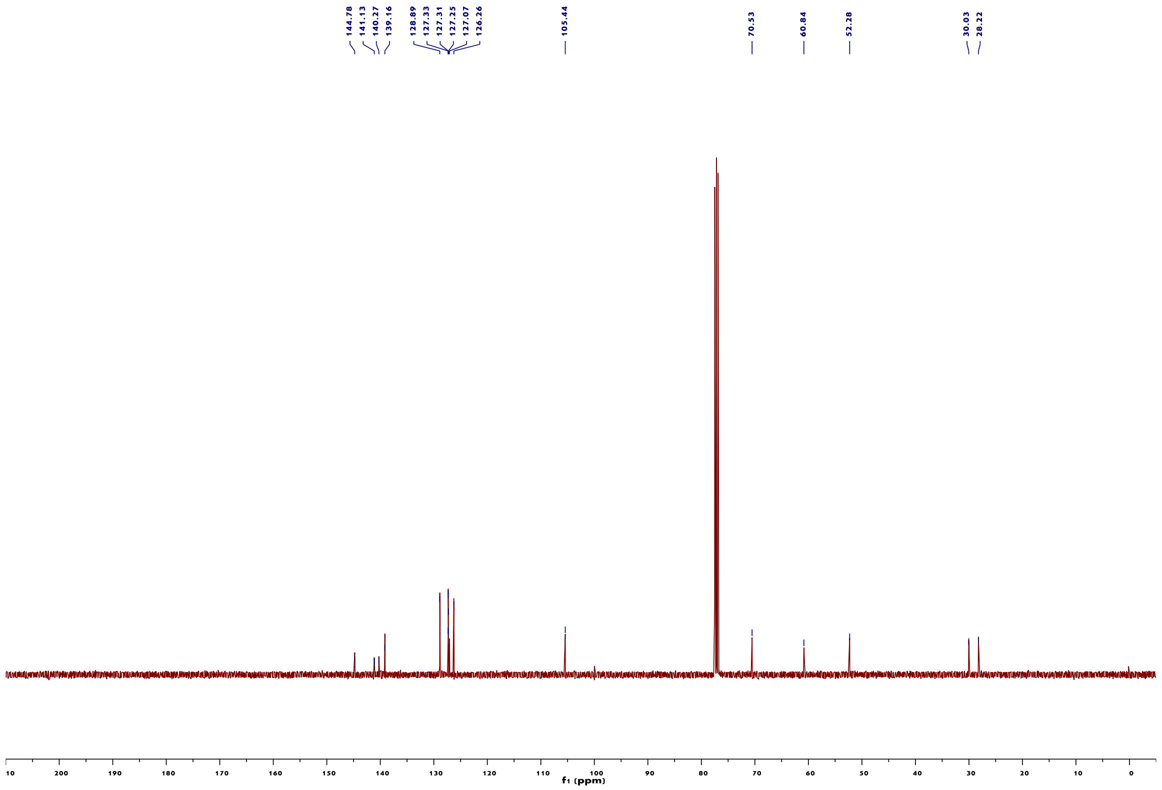

Supplementary Figure 117. ^{13}C NMR (100 MHz, $CDCl_3$) spectrum for Int-ha

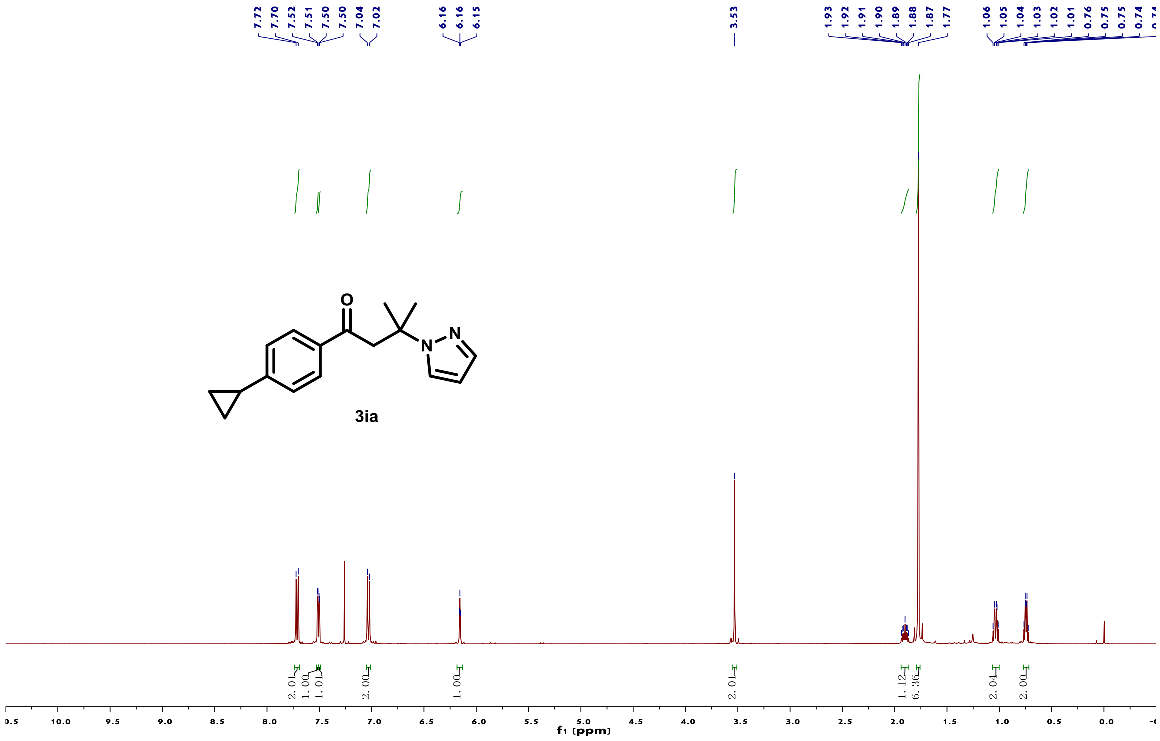

Supplementary Figure 118. ^{1}H NMR (400 MHz, $CDCl_3$) spectrum for 3ia

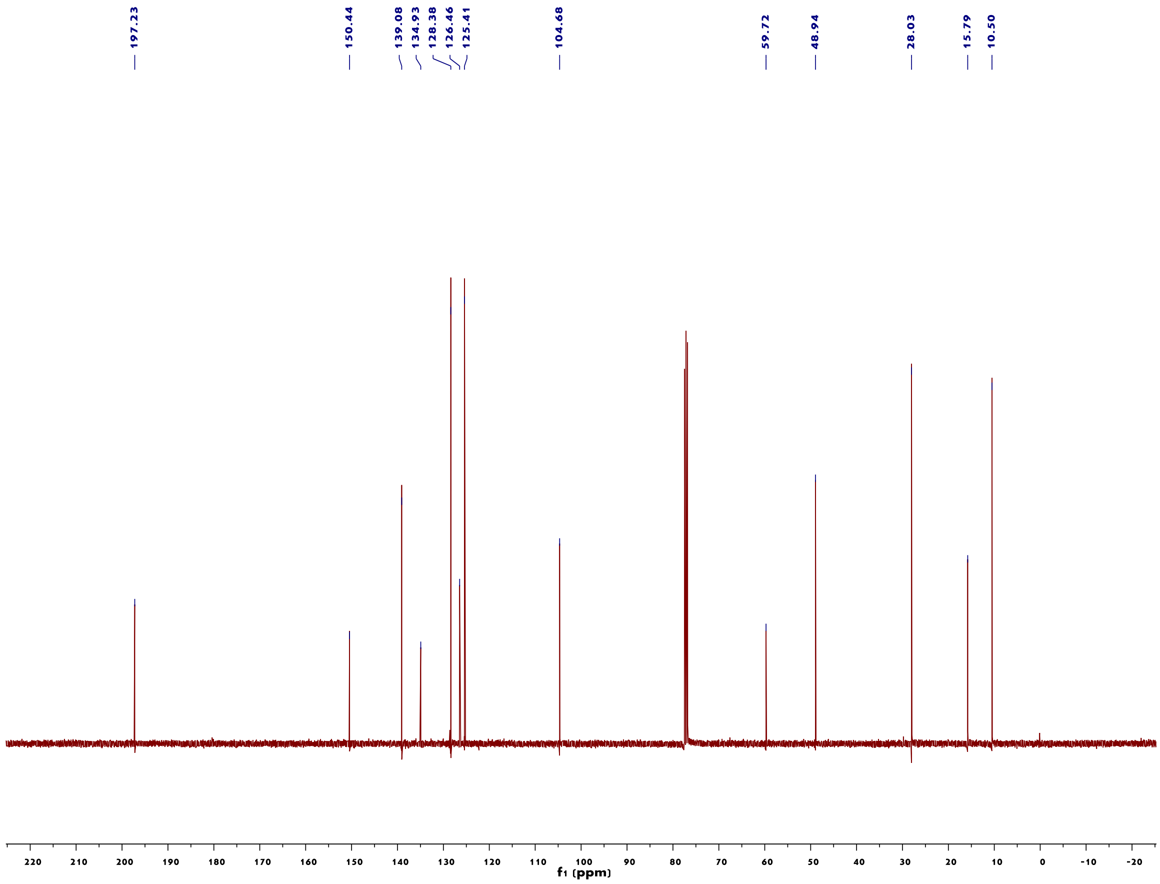

Supplementary Figure 119. ^{13}C NMR (100 MHz, $CDCl_3$) spectrum for 3ia

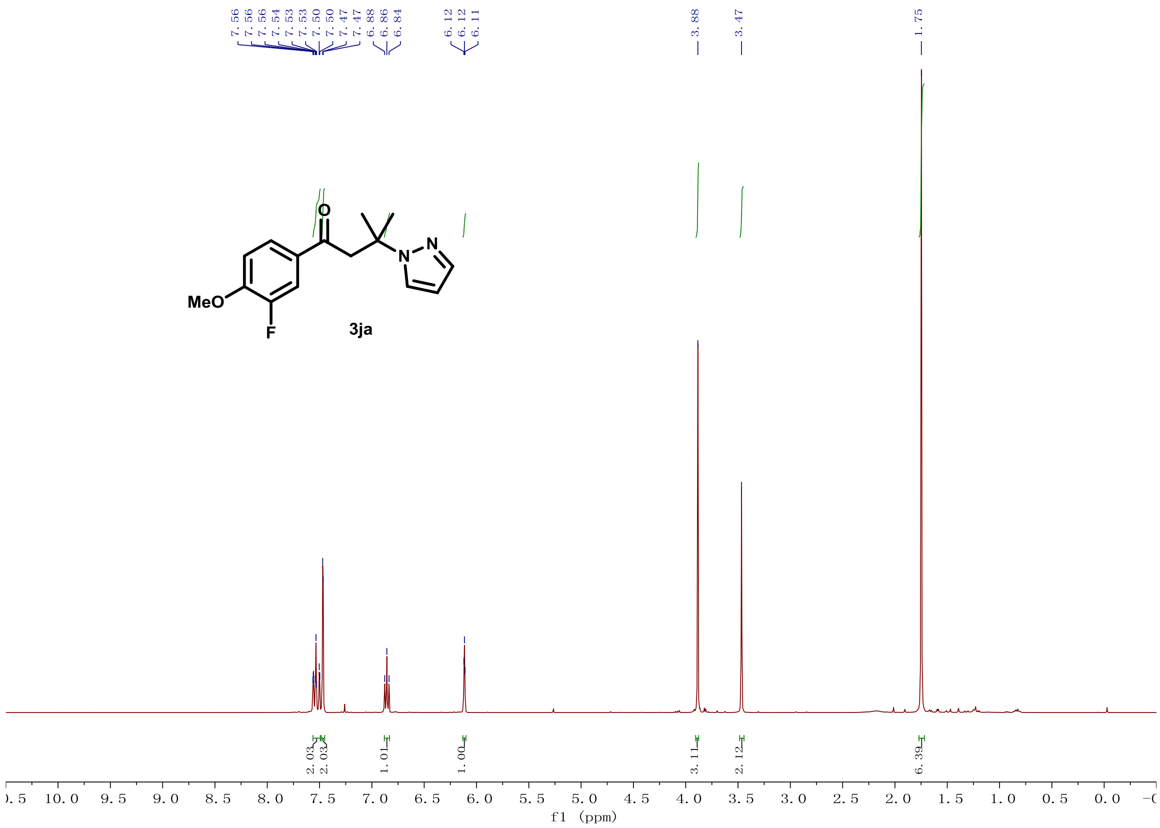

Supplementary Figure 120. ^{1}H NMR (400 MHz, $CDCl_3$) spectrum for 3ja

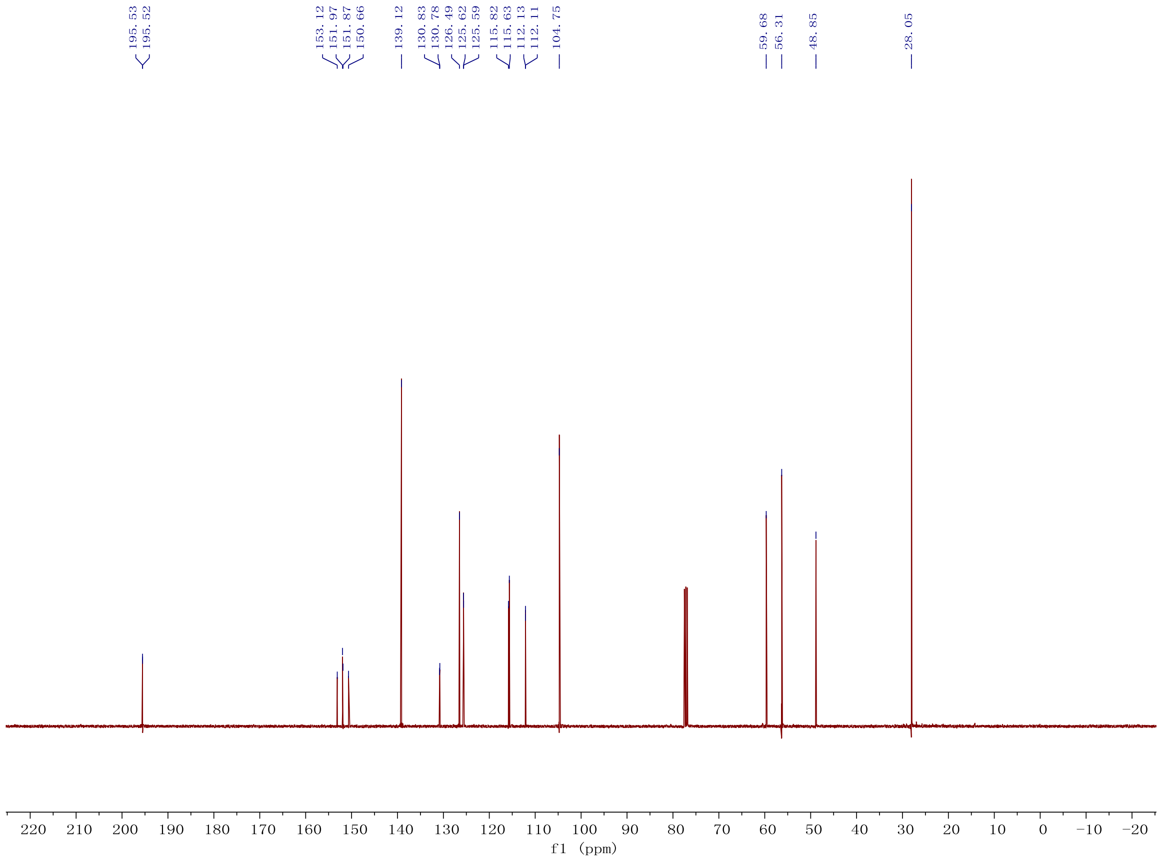

Supplementary Figure 121. ^{13}C NMR (100 MHz, $CDCl_3$) spectrum for 3ja

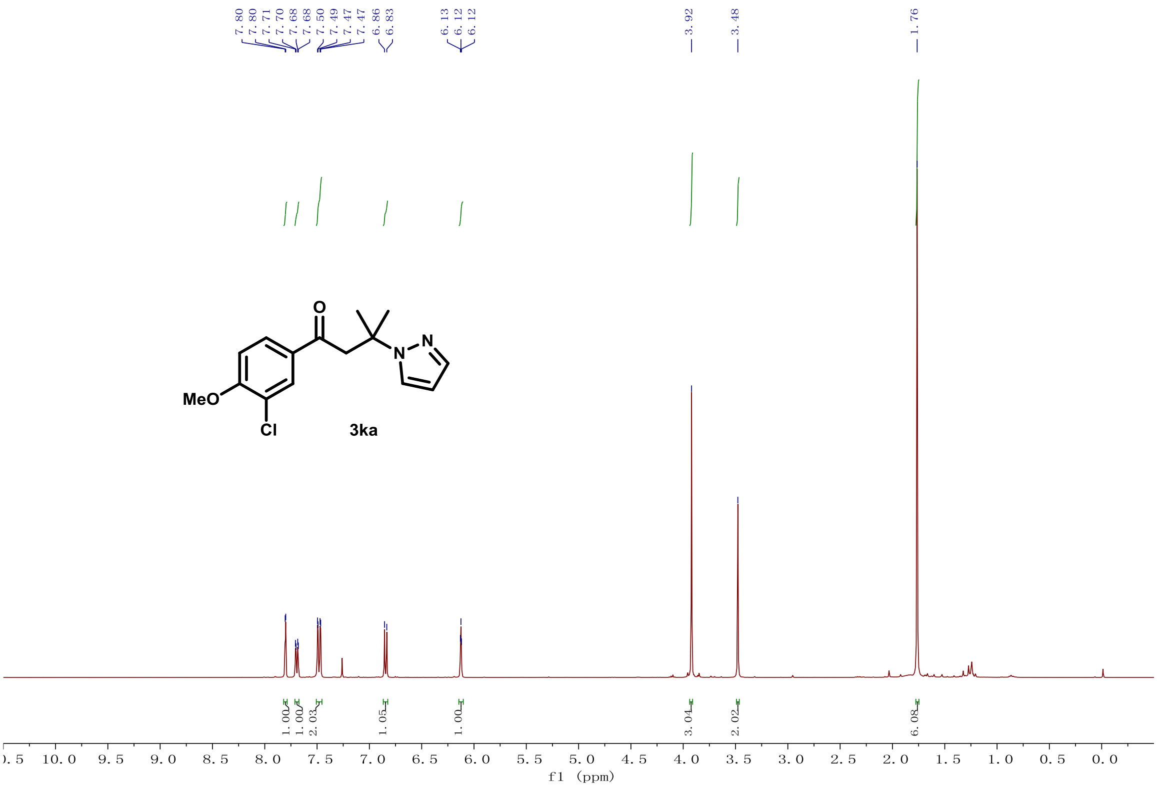

Supplementary Figure 122. ^{1}H **NMR (400 MHz,** $CDCl_3$**) spectrum for 3ka**

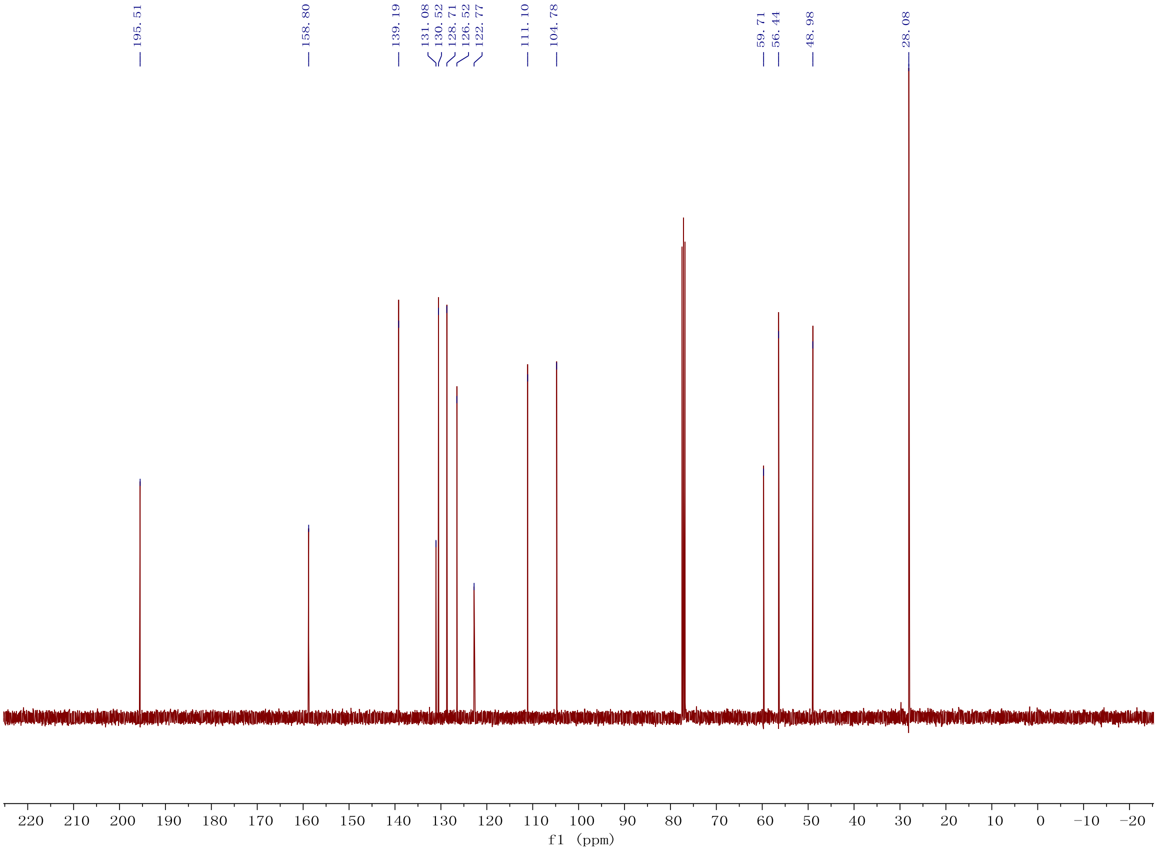

Supplementary Figure 123. ^{13}C **NMR (100 MHz,** $CDCl_3$**) spectrum for 3ka**

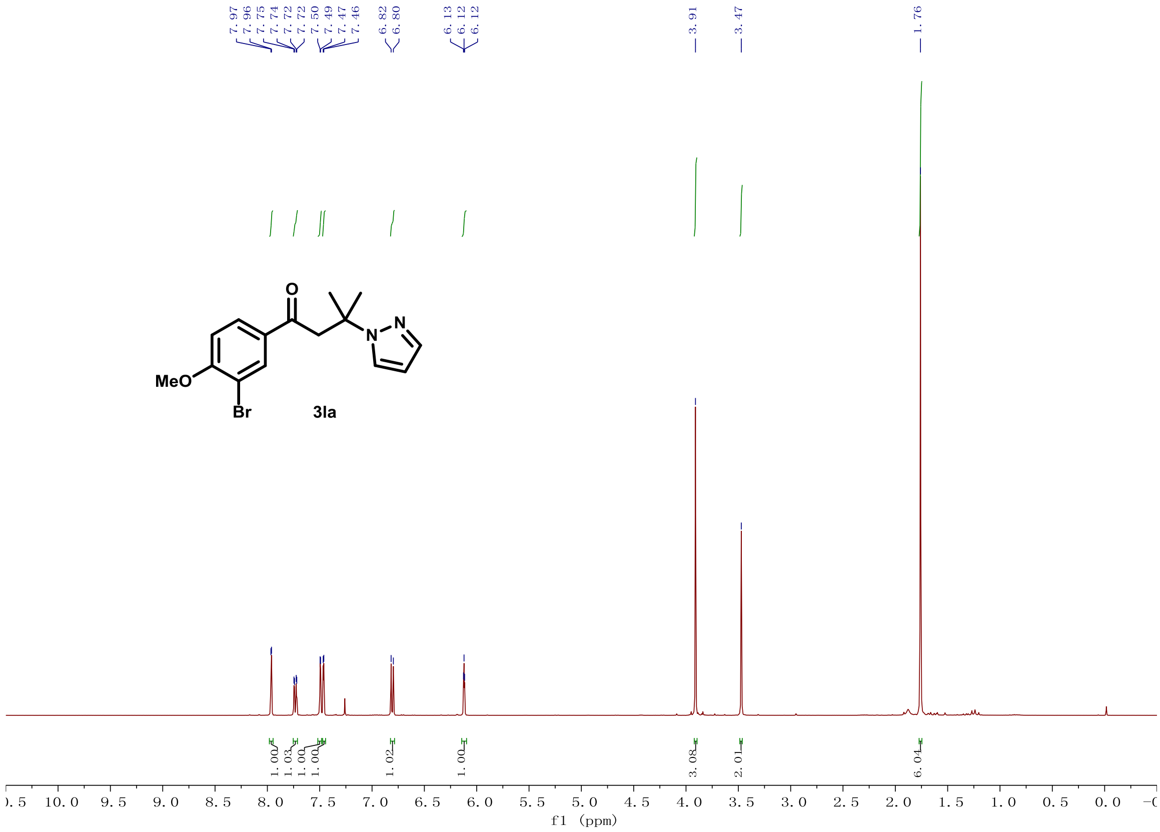

Supplementary Figure 124. ^{1}H NMR (400 MHz, CDCl$_3$) spectrum for 3la

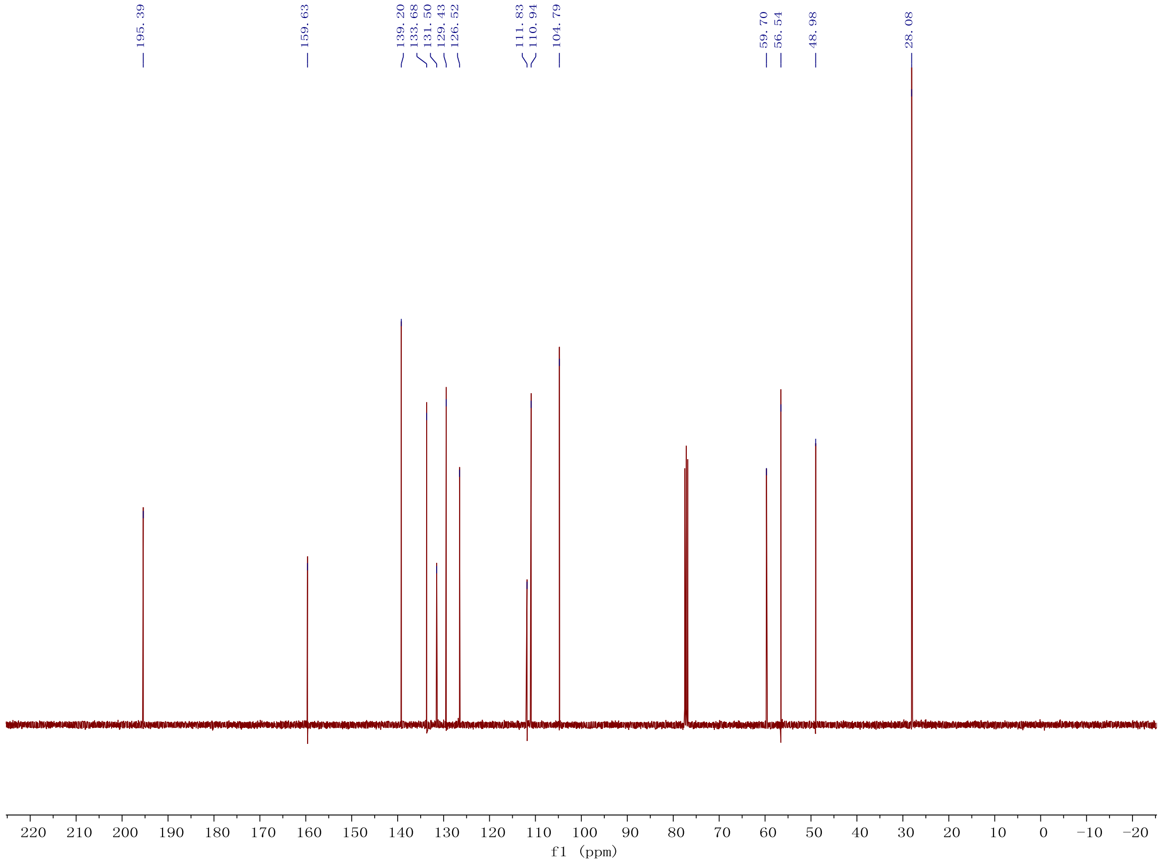

Supplementary Figure 125. ^{13}C NMR (100 MHz, CDCl$_3$) spectrum for 3la

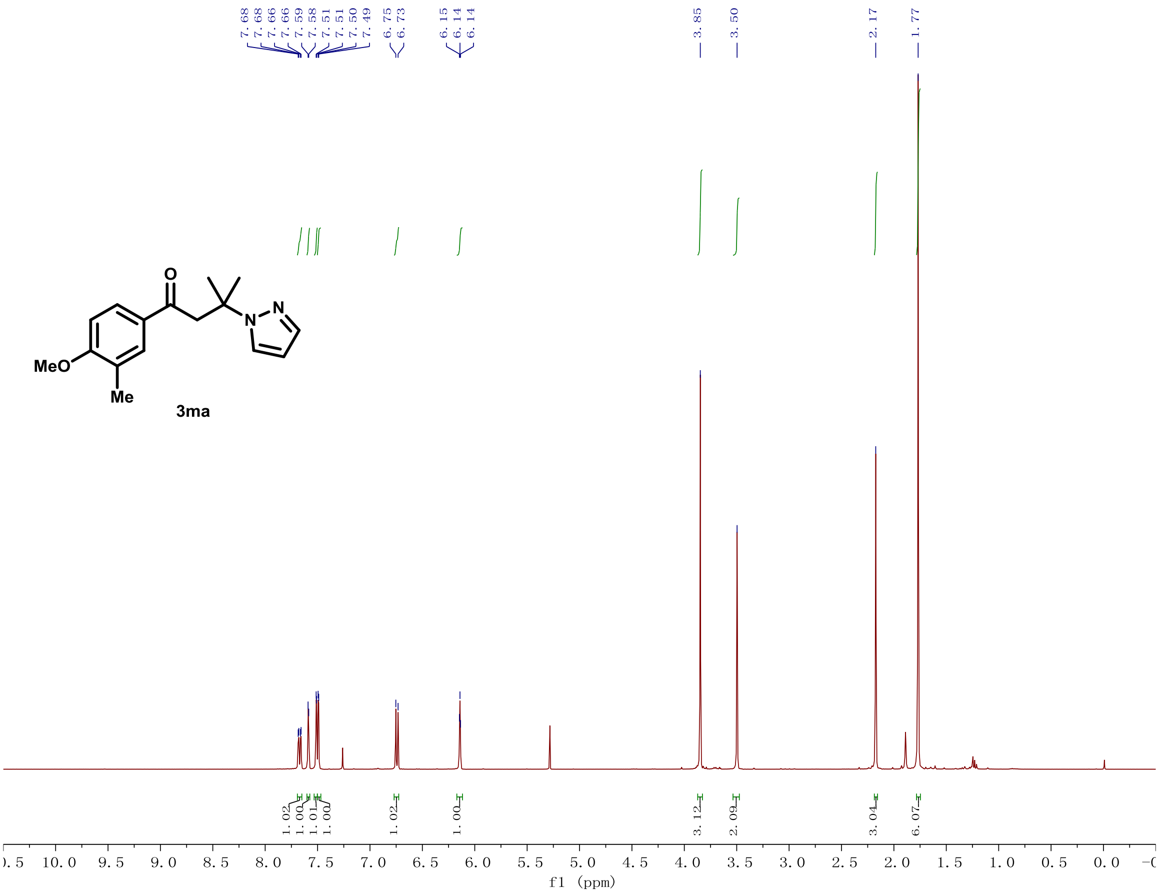

Supplementary Figure 126. ^{1}H NMR (400 MHz, $CDCl_3$) spectrum for 3ma

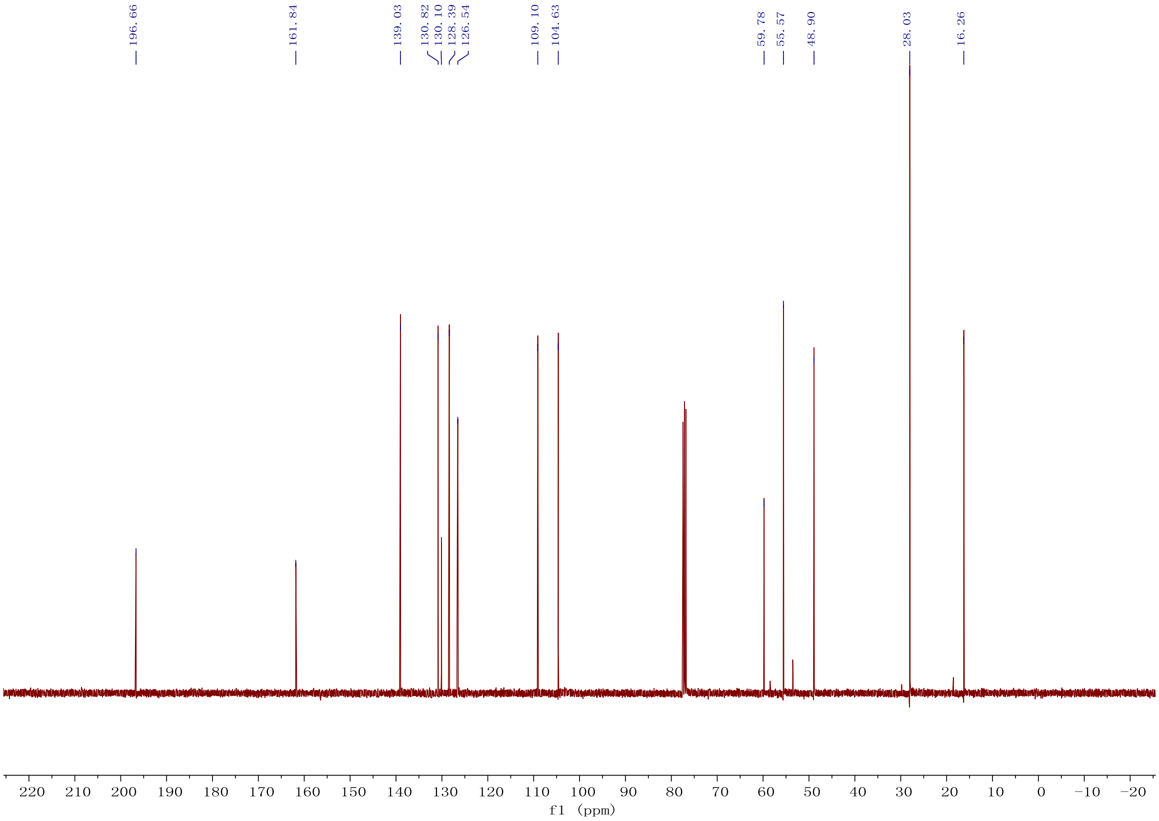

Supplementary Figure 127. ^{13}C NMR (100 MHz, $CDCl_3$) spectrum for 3ma

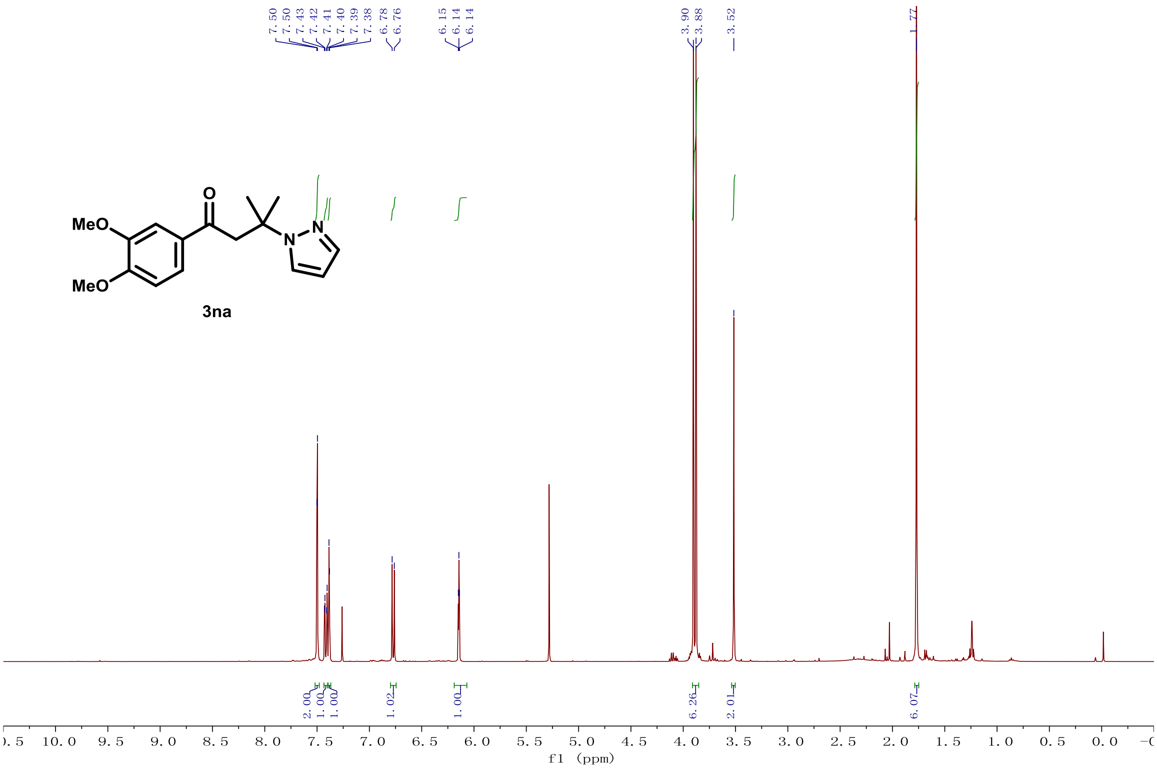

Supplementary Figure 128. **^{1}H NMR (400 MHz, $CDCl_3$) spectrum for 3na**

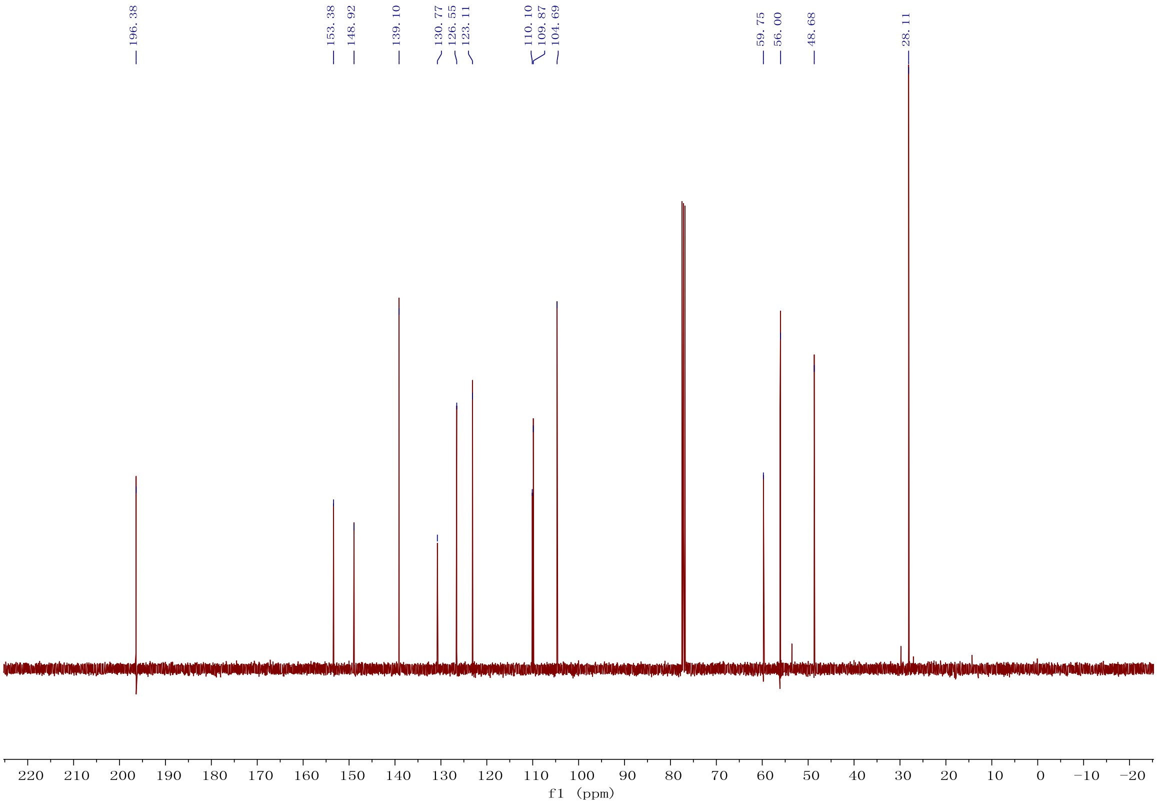

Supplementary Figure 129. **^{13}C NMR (100 MHz, $CDCl_3$) spectrum for 3na**

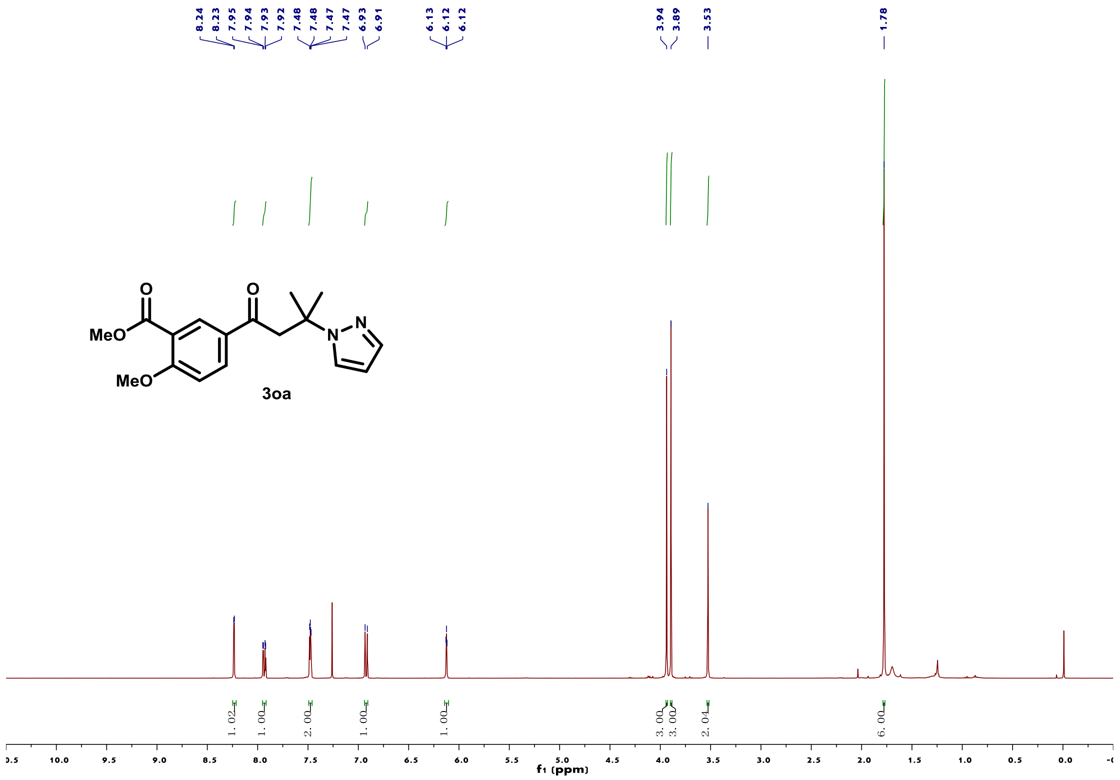

Supplementary Figure 130. ^{1}H NMR (400 MHz, $CDCl_3$) spectrum for 3oa

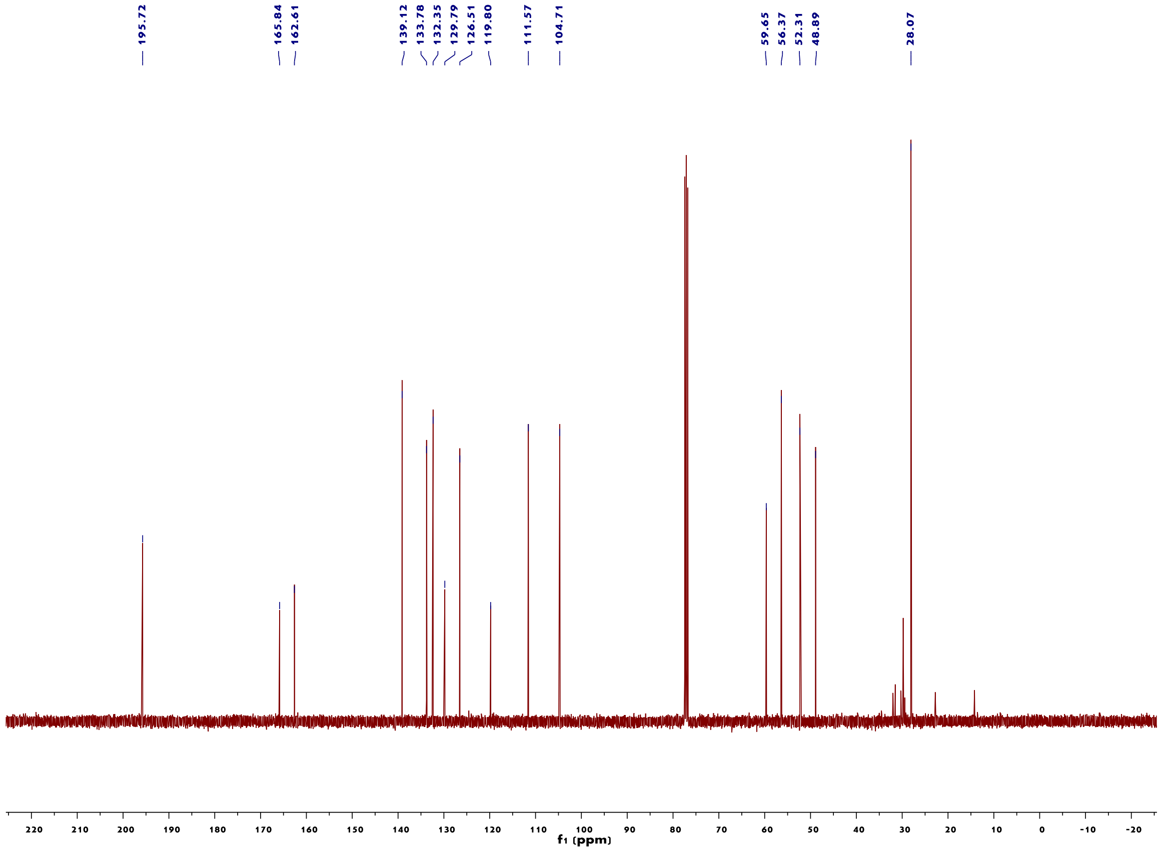

Supplementary Figure 131. ^{13}C NMR (100 MHz, $CDCl_3$) spectrum for 3oa

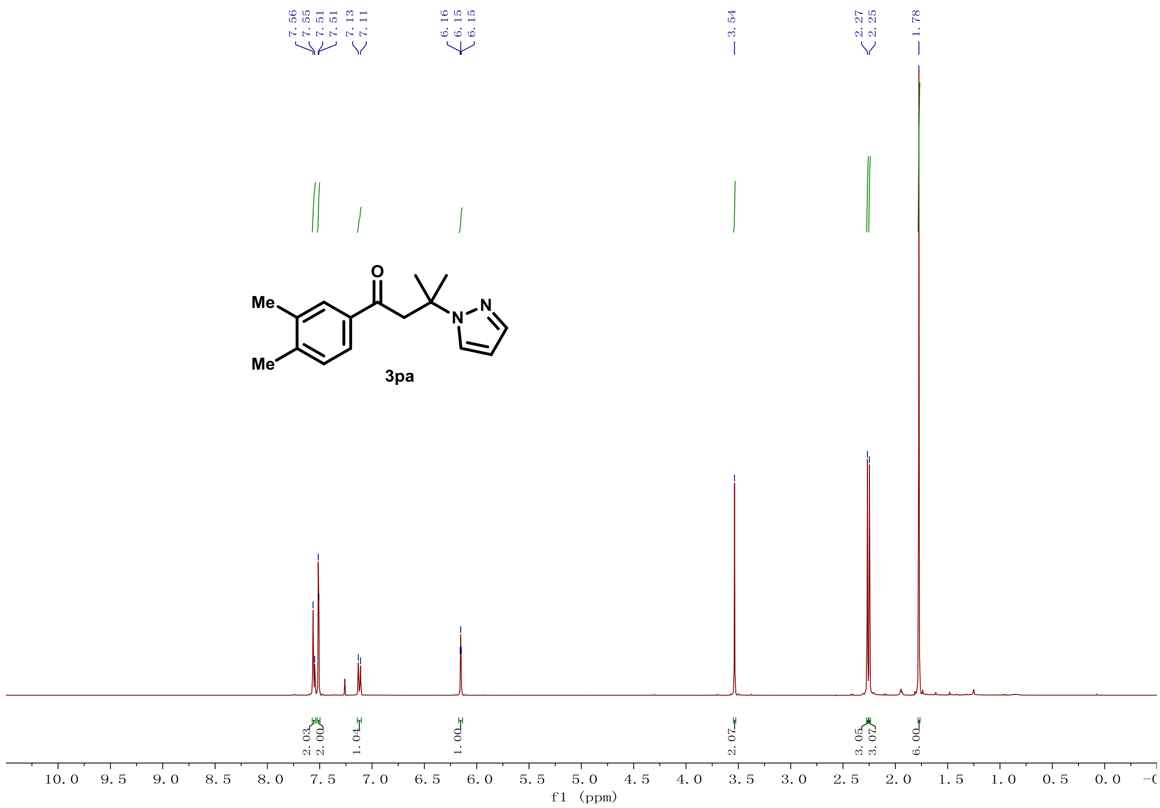

Supplementary Figure 132. ^{1}H NMR (400 MHz, $CDCl_3$) spectrum for 3pa

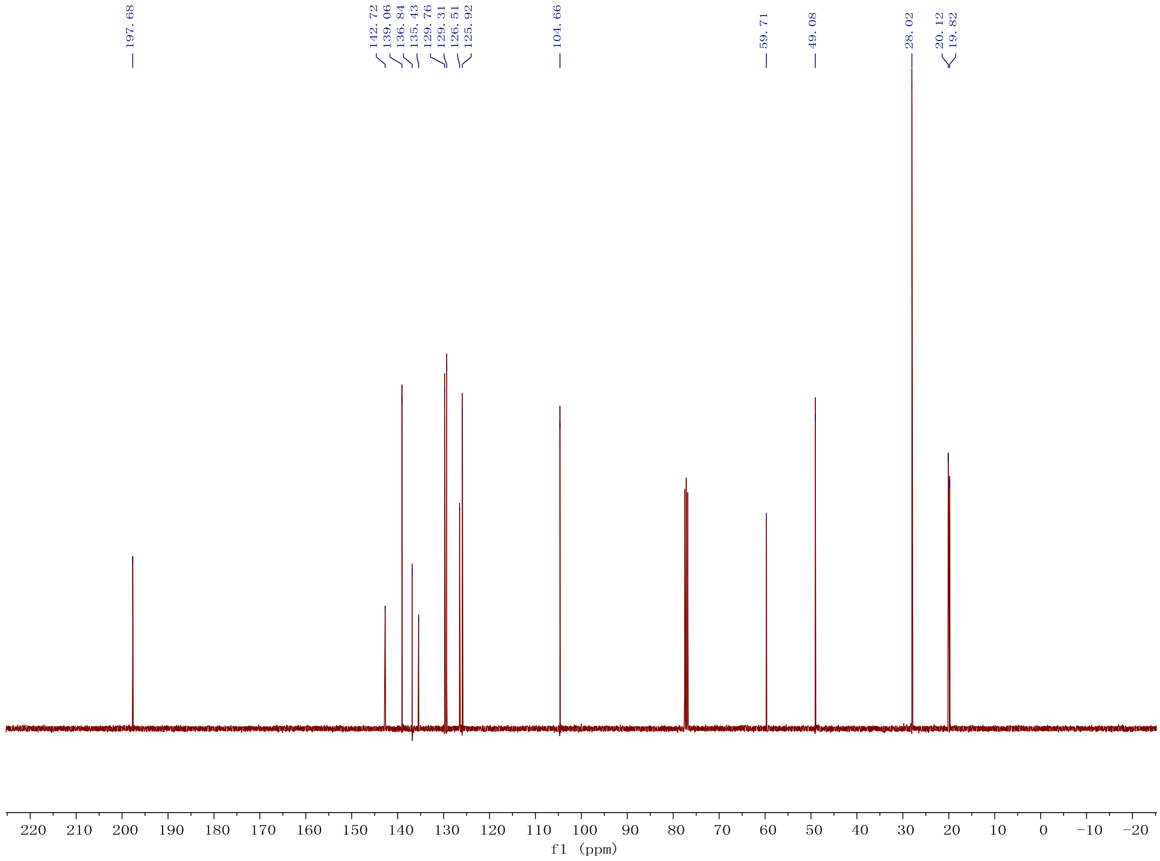

Supplementary Figure 133. ^{13}C NMR (100 MHz, $CDCl_3$) spectrum for 3pa

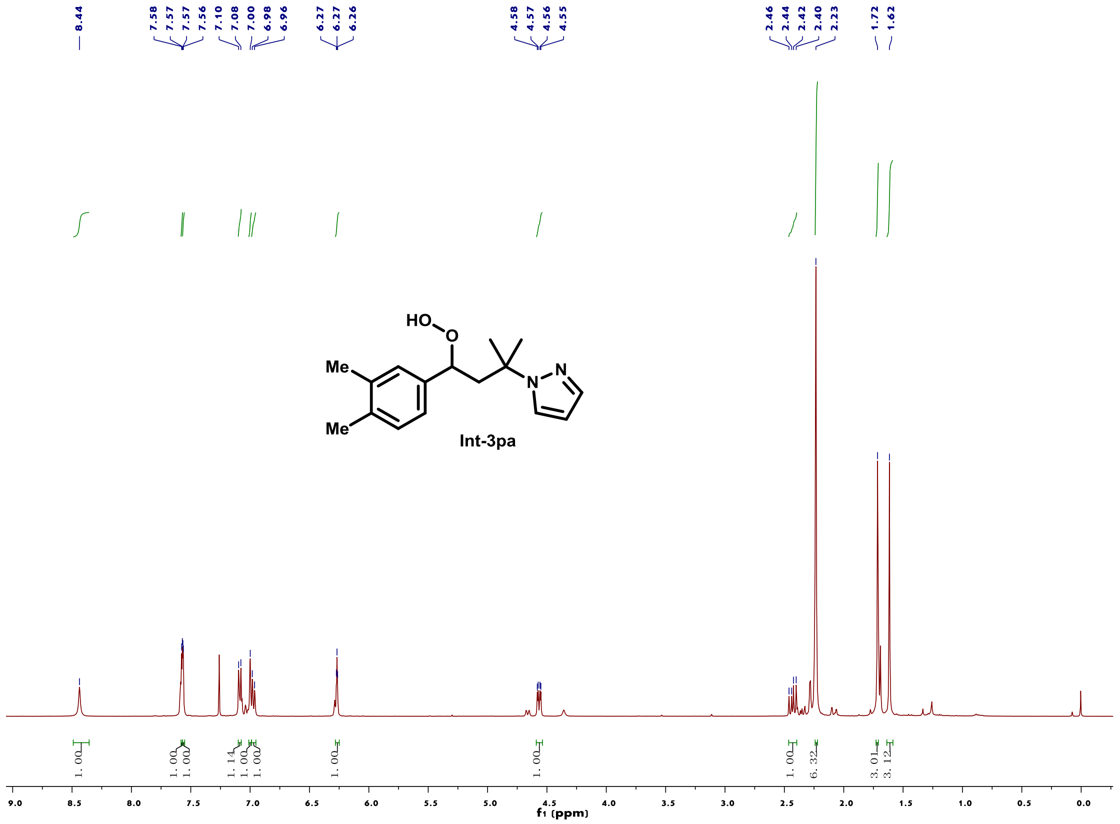

Supplementary Figure 134. ^{1}H NMR (400 MHz, $CDCl_3$) spectrum for Int-3pa

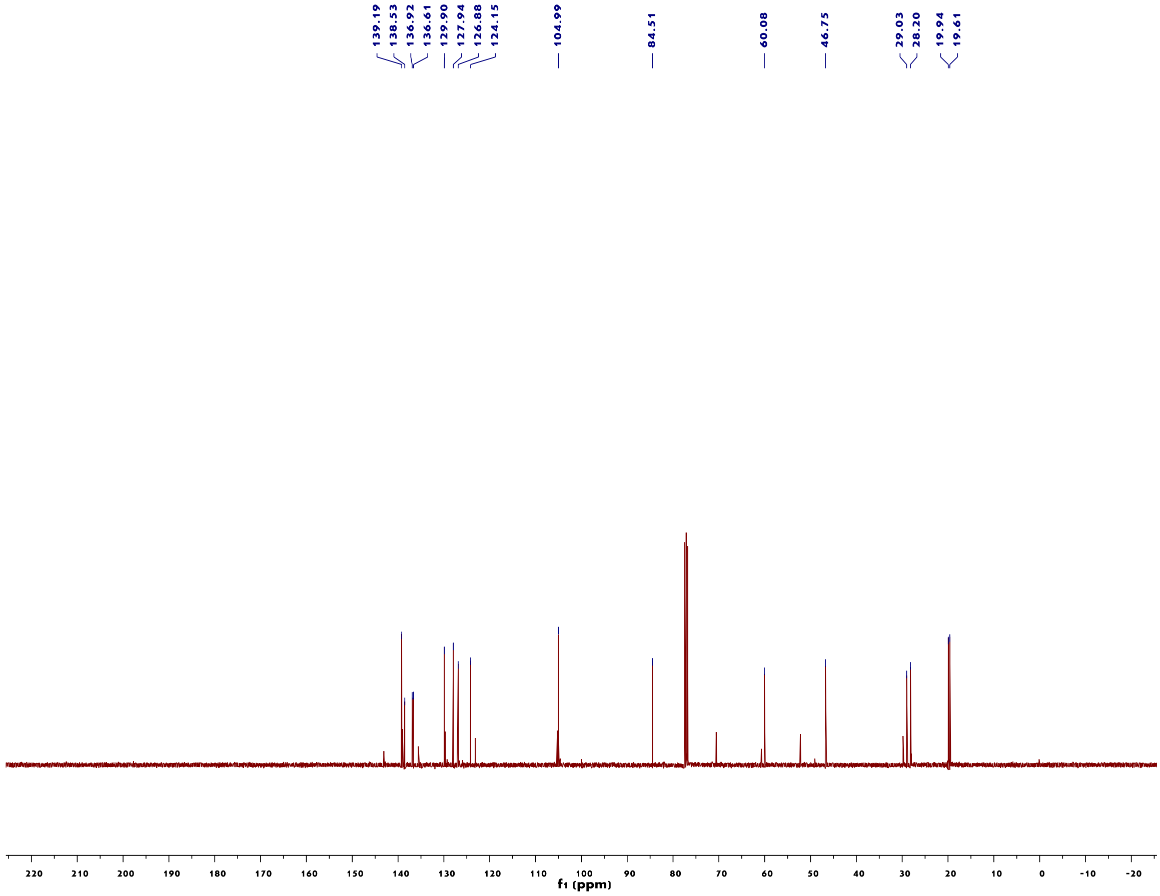

Supplementary Figure 135. ^{13}C NMR (100 MHz, $CDCl_3$) spectrum for Int-3pa

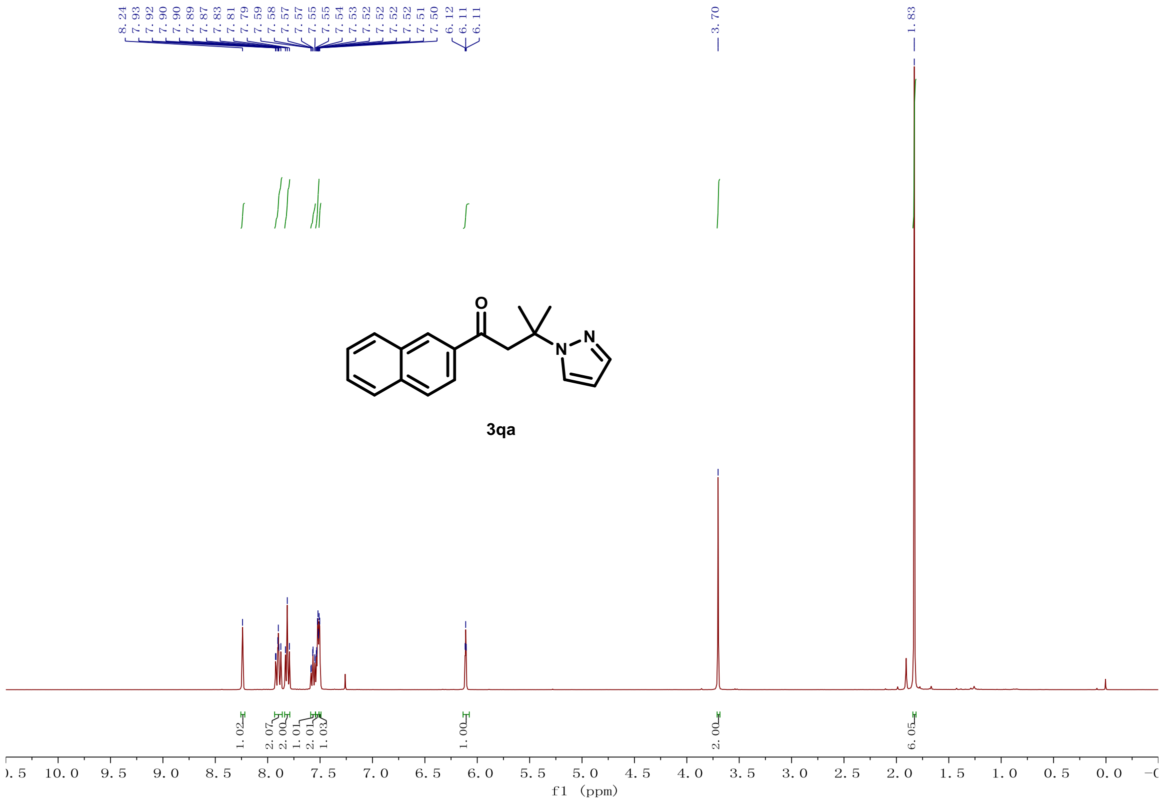

Supplementary Figure 136. ^{1}H NMR (400 MHz, $CDCl_3$) spectrum for 3qa

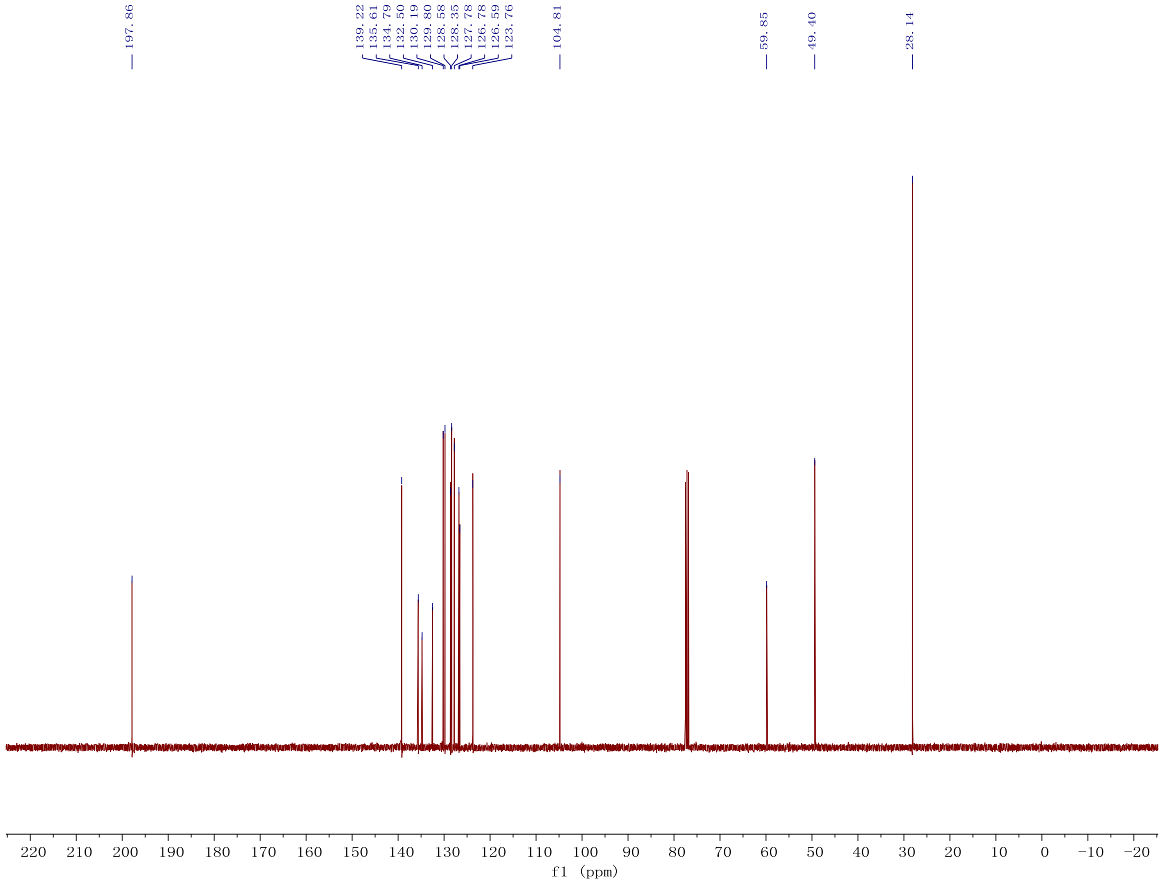

Supplementary Figure 137. ^{13}C NMR (100 MHz, $CDCl_3$) spectrum for 3qa

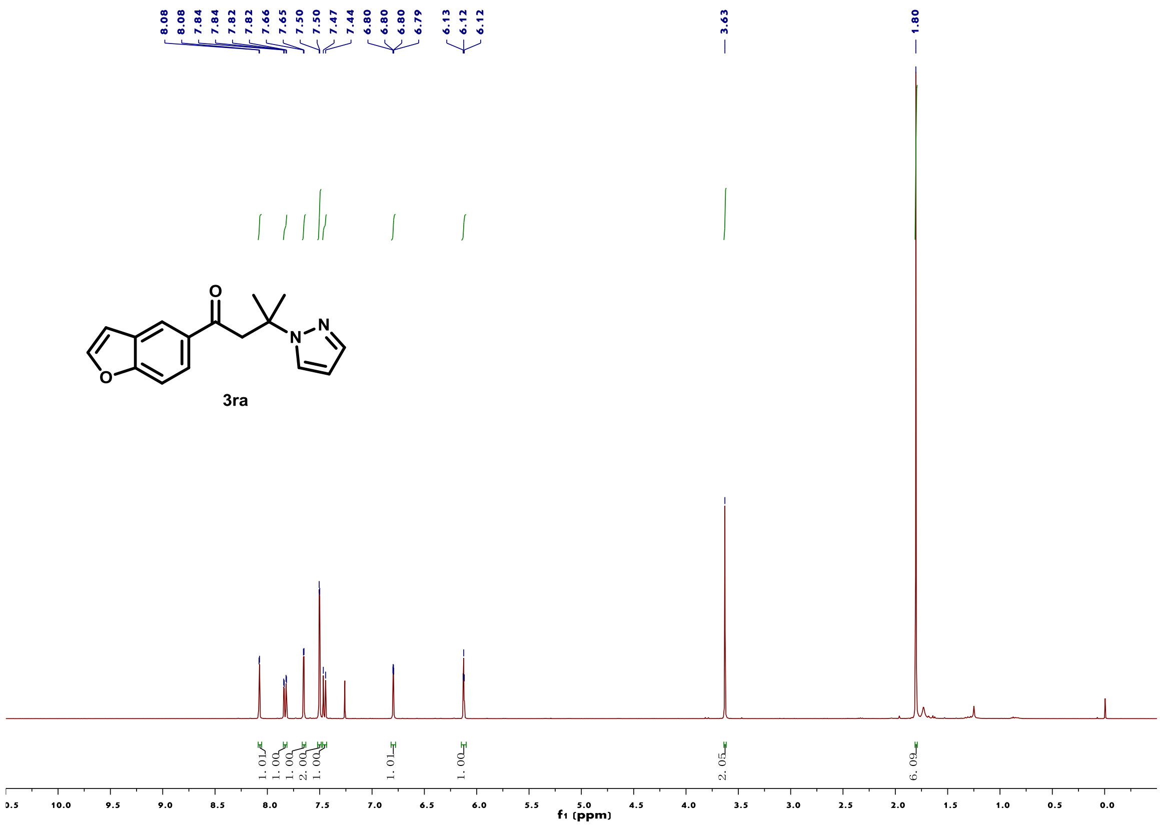

Supplementary Figure 138. ^{1}H NMR (400 MHz, $CDCl_3$) spectrum for 3ra

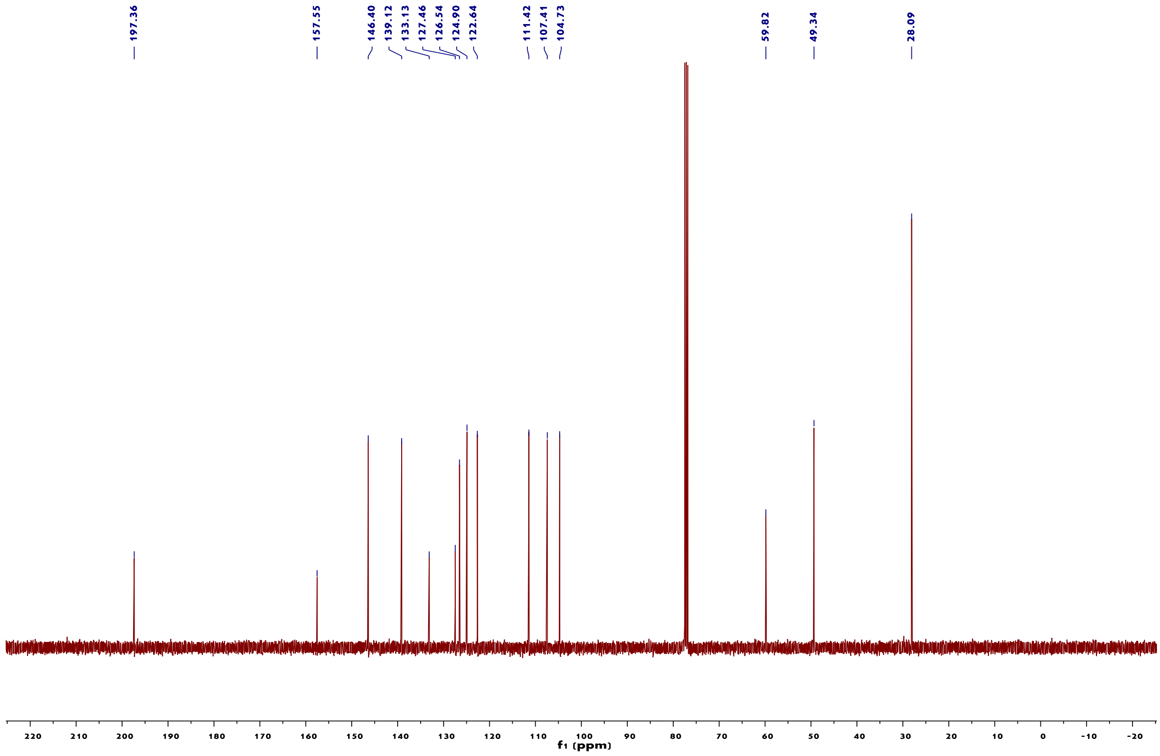

Supplementary Figure 139. ^{13}C NMR (100 MHz, $CDCl_3$) spectrum for 3ra

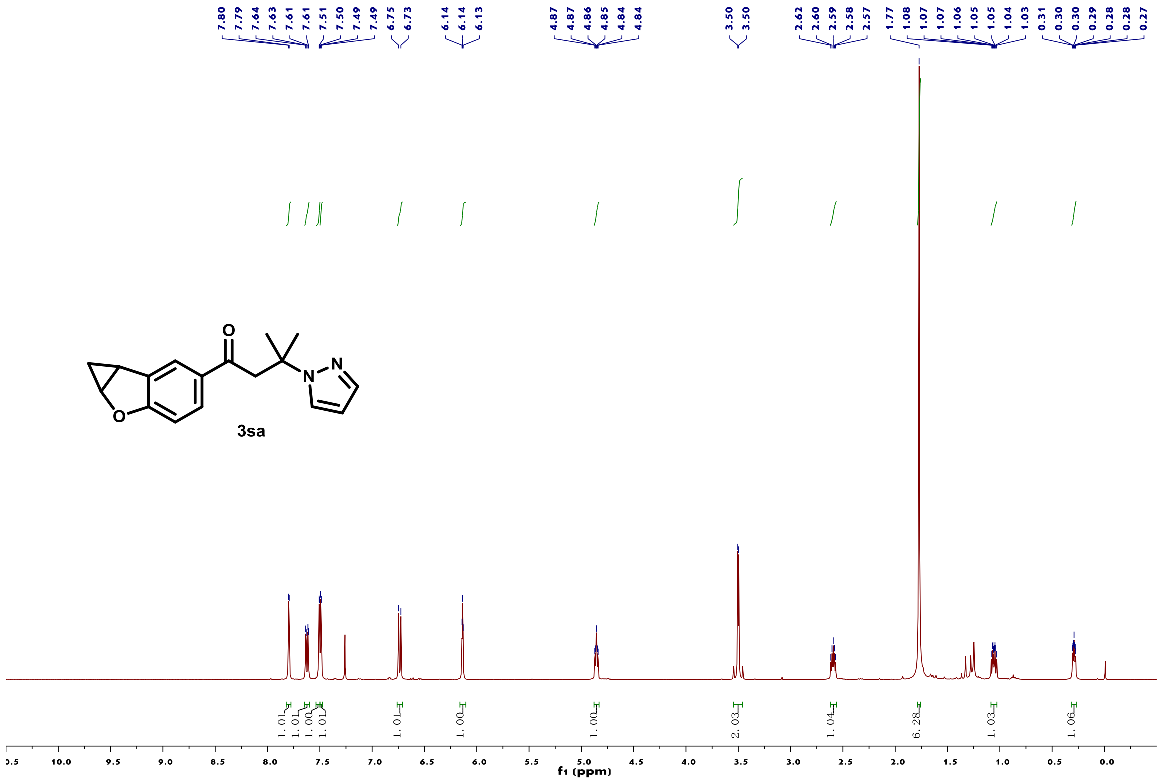

Supplementary Figure 140. ^{1}H NMR (400 MHz, $CDCl_3$) spectrum for 3sa

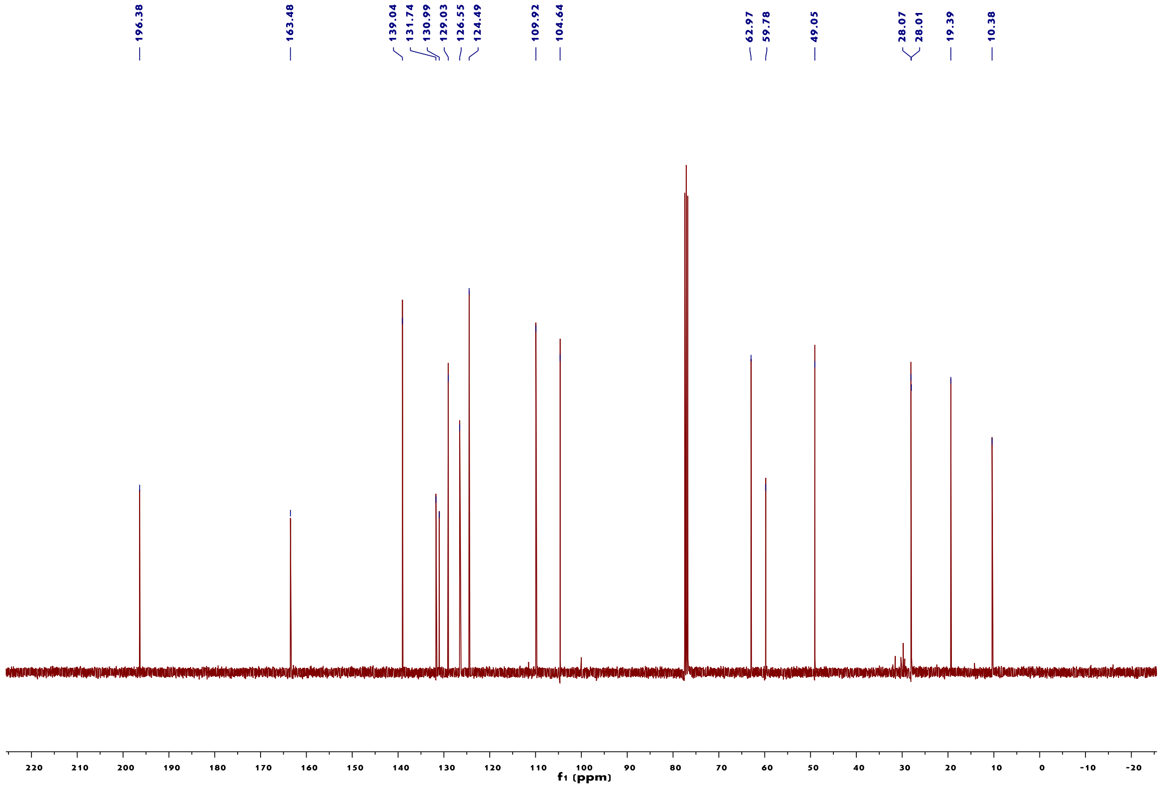

Supplementary Figure 141. ^{13}C NMR (100 MHz, $CDCl_3$) spectrum for 3sa

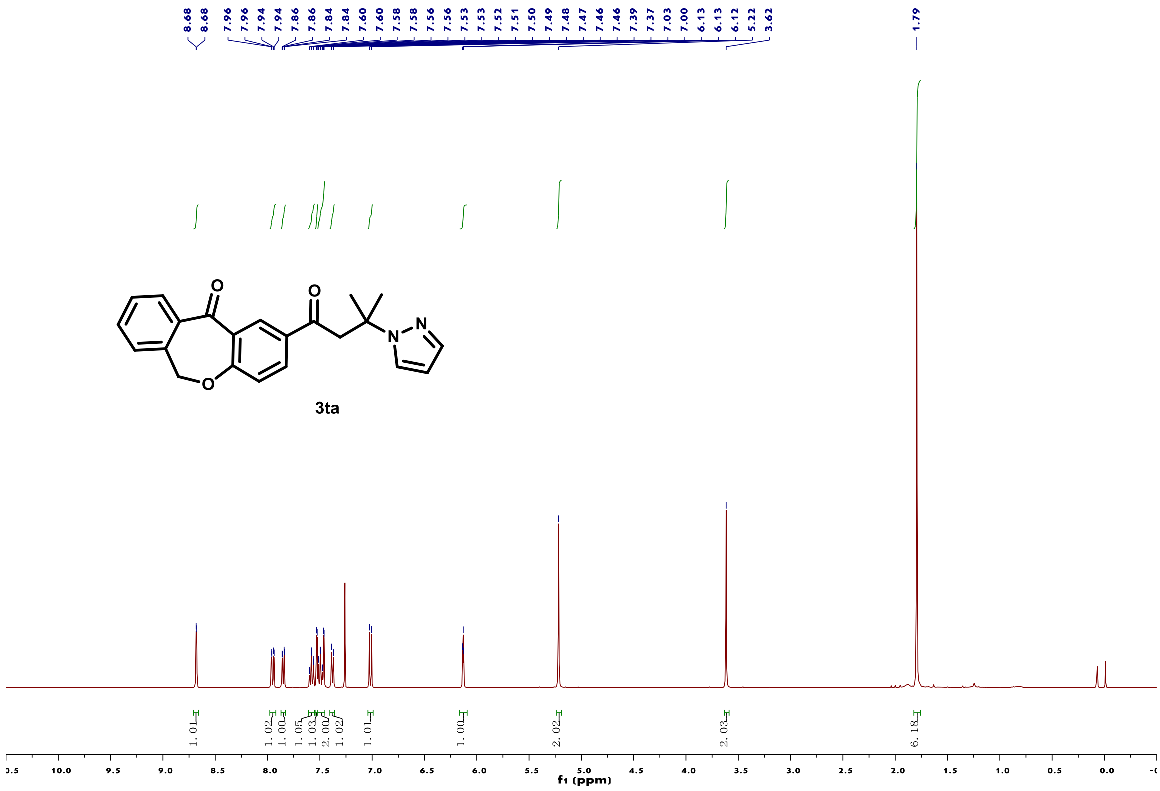

Supplementary Figure 142. ^{1}H NMR (400 MHz, $CDCl_3$) spectrum for 3ta

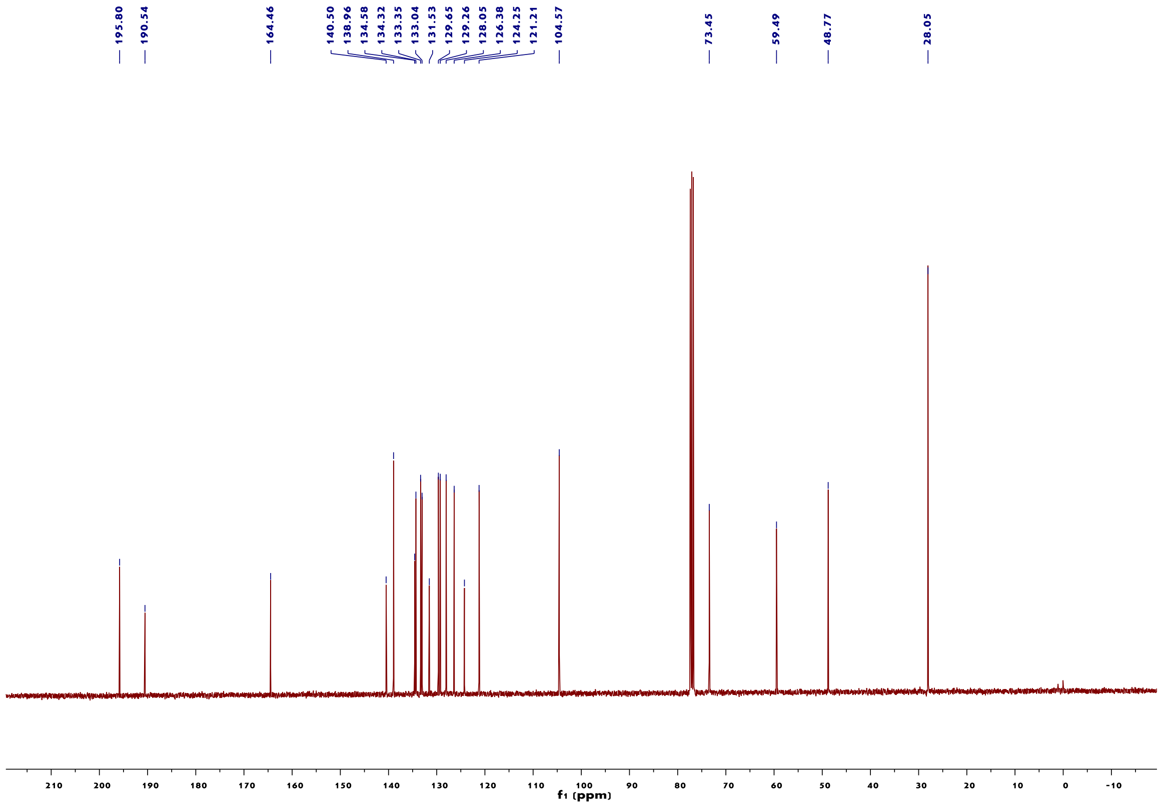

Supplementary Figure 143. ^{13}C NMR (100 MHz, $CDCl_3$) spectrum for 3ta

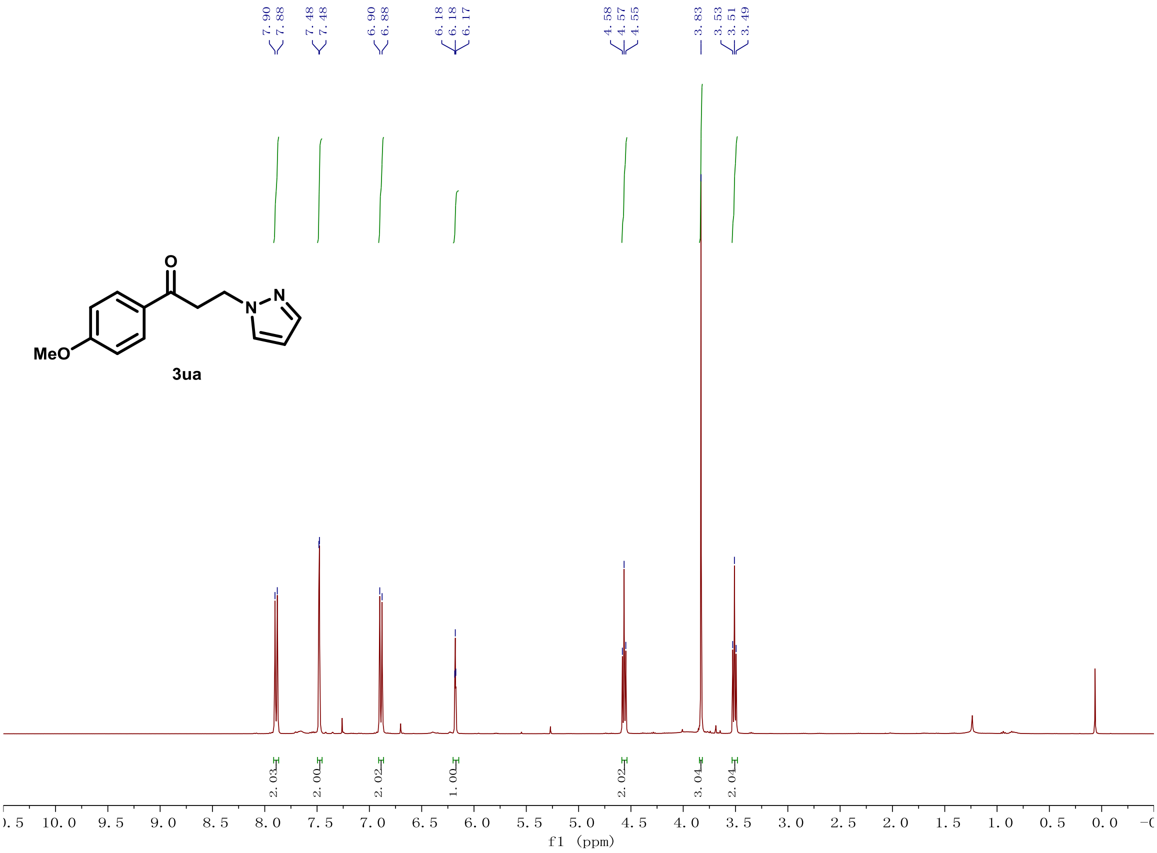

Supplementary Figure 144. ^{1}H NMR (400 MHz, $CDCl_3$) spectrum for 3ua

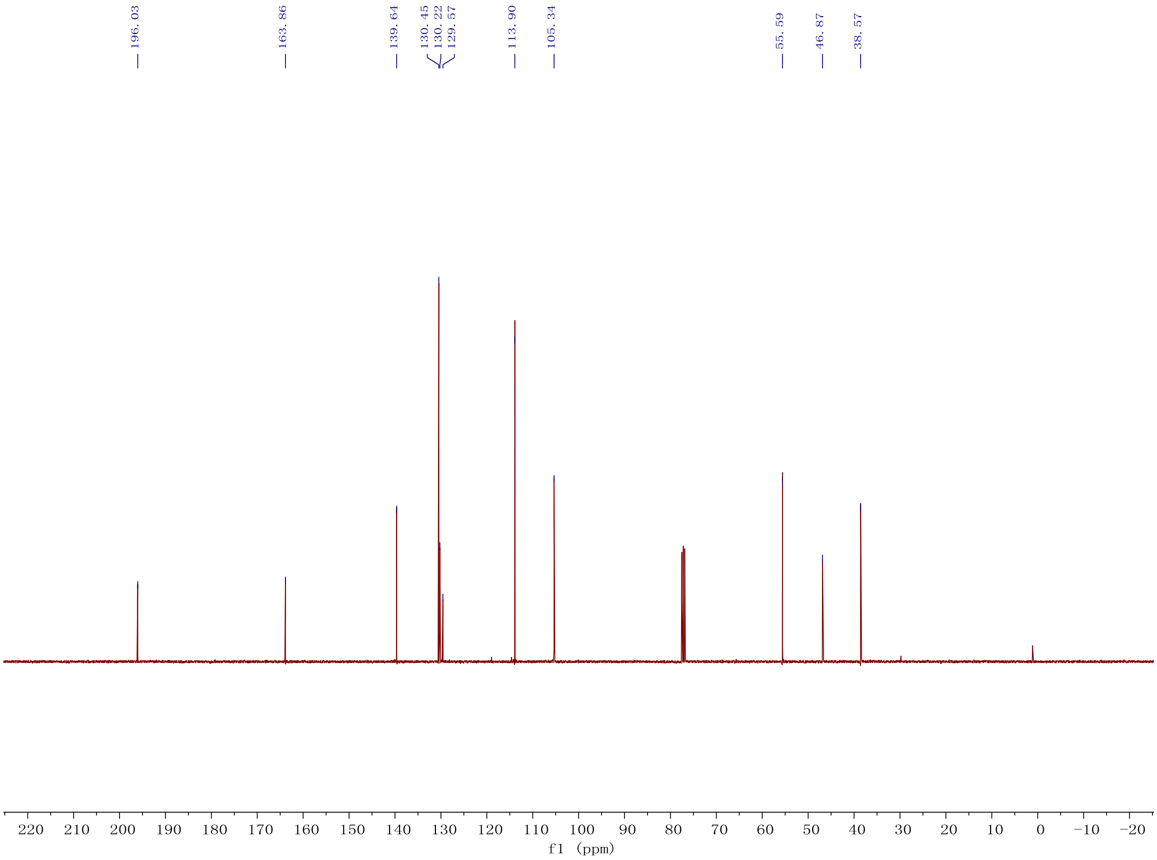

Supplementary Figure 145. ^{13}C NMR (100 MHz, $CDCl_3$) spectrum for 3ua

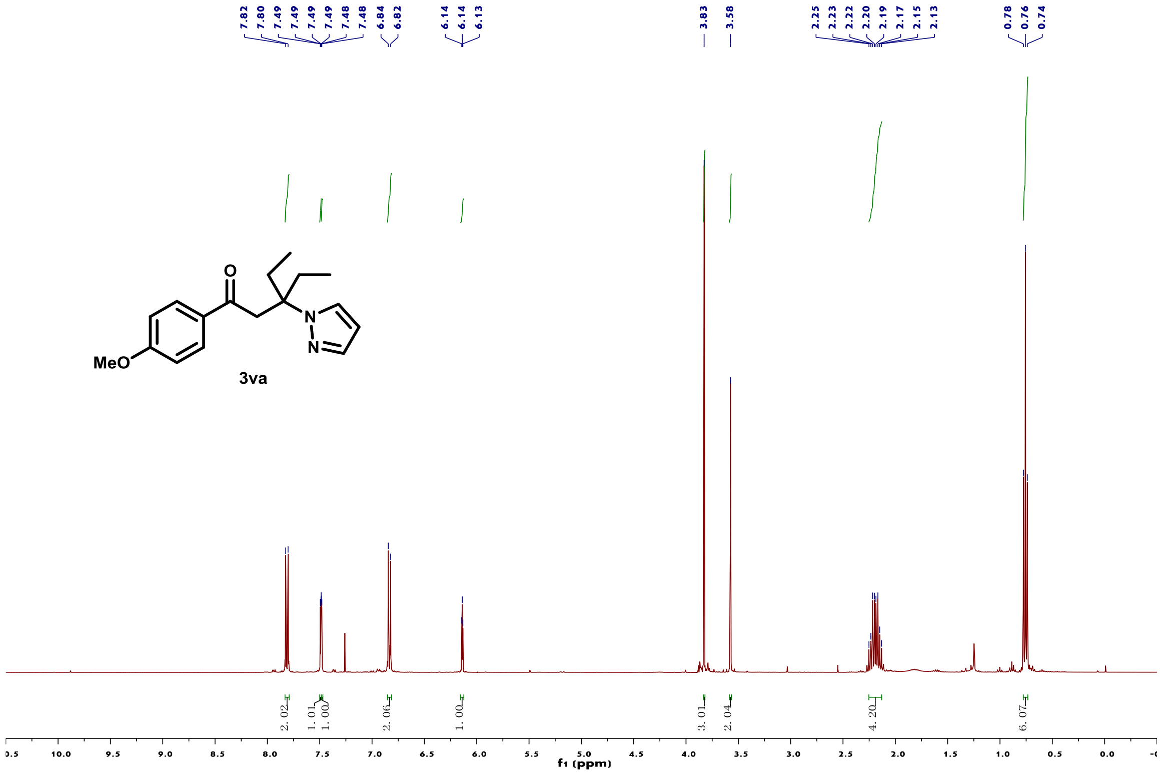

Supplementary Figure 146. ^{1}H **NMR (400 MHz, $CDCl_3$) spectrum for 3va**

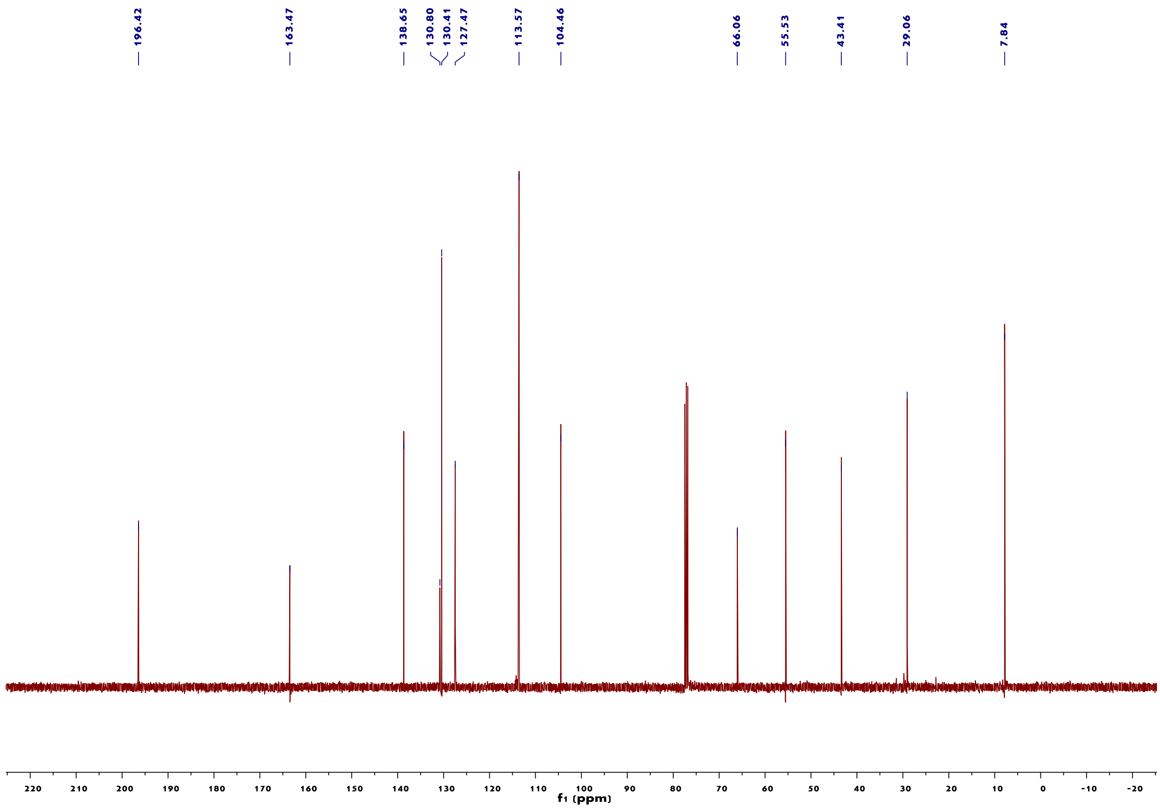

Supplementary Figure 147. ^{13}C **NMR (100 MHz, $CDCl_3$) spectrum for 3va**

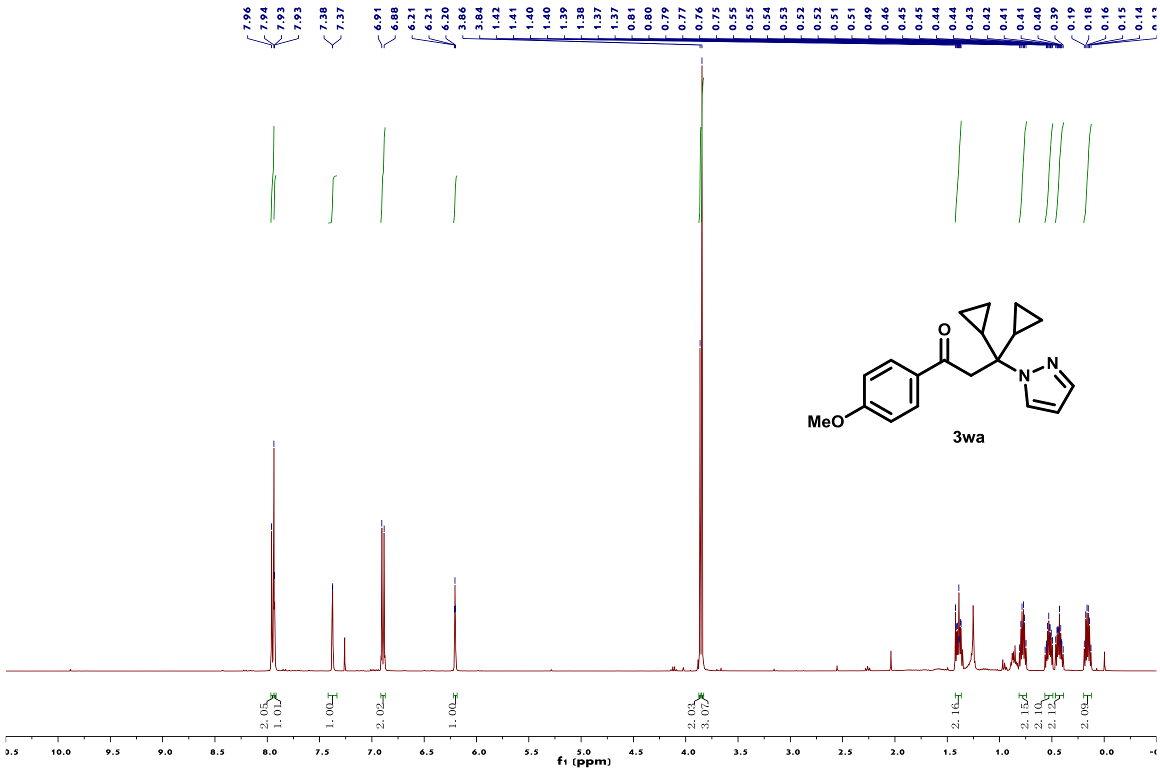

Supplementary Figure 148. ^{1}H NMR (400 MHz, $CDCl_3$) spectrum for 3wa

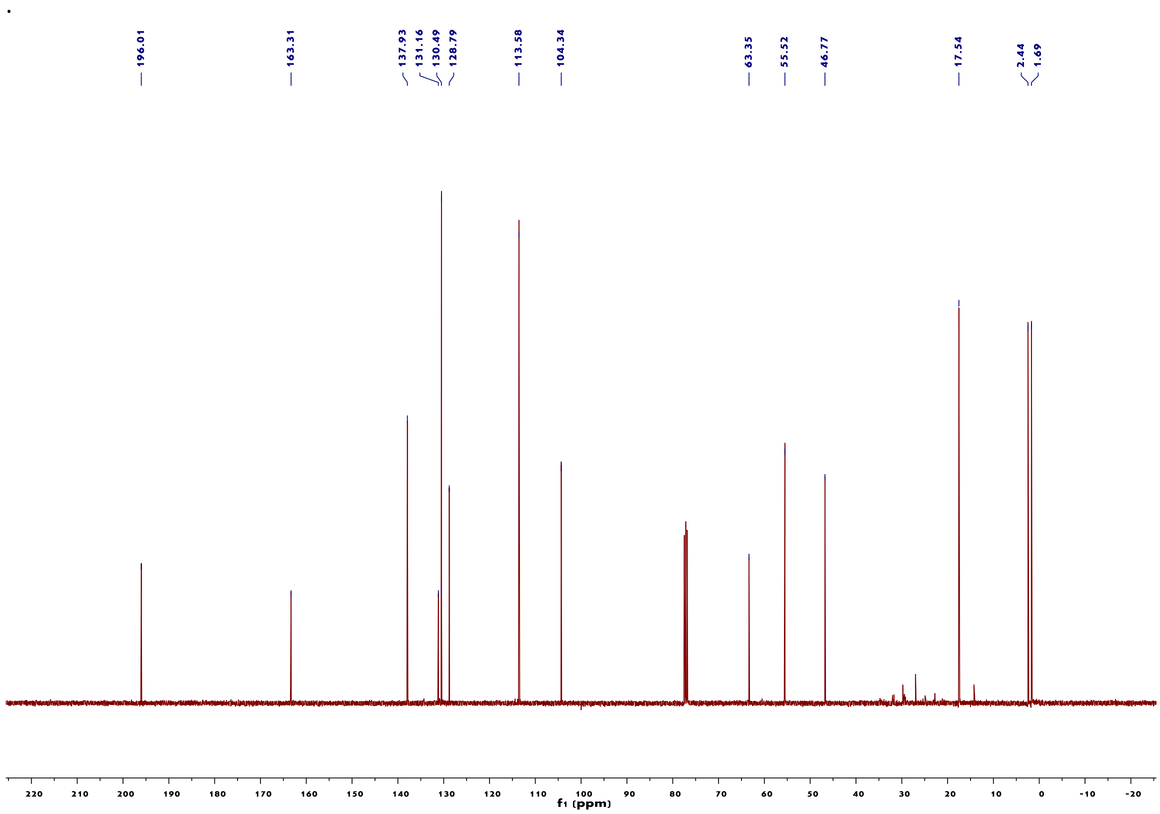

Supplementary Figure 149. ^{13}C NMR (100 MHz, $CDCl_3$) spectrum for 3wa

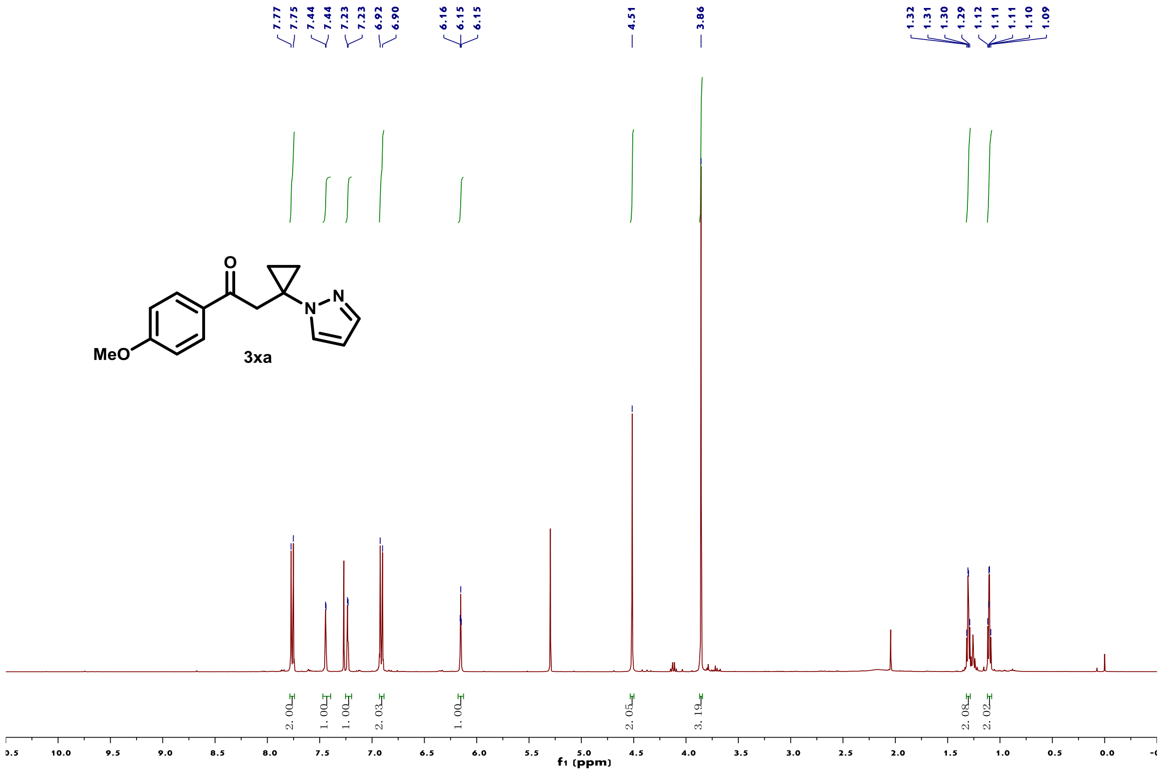

Supplementary Figure 150. ^{1}H NMR (400 MHz, $CDCl_3$) spectrum for 3xa

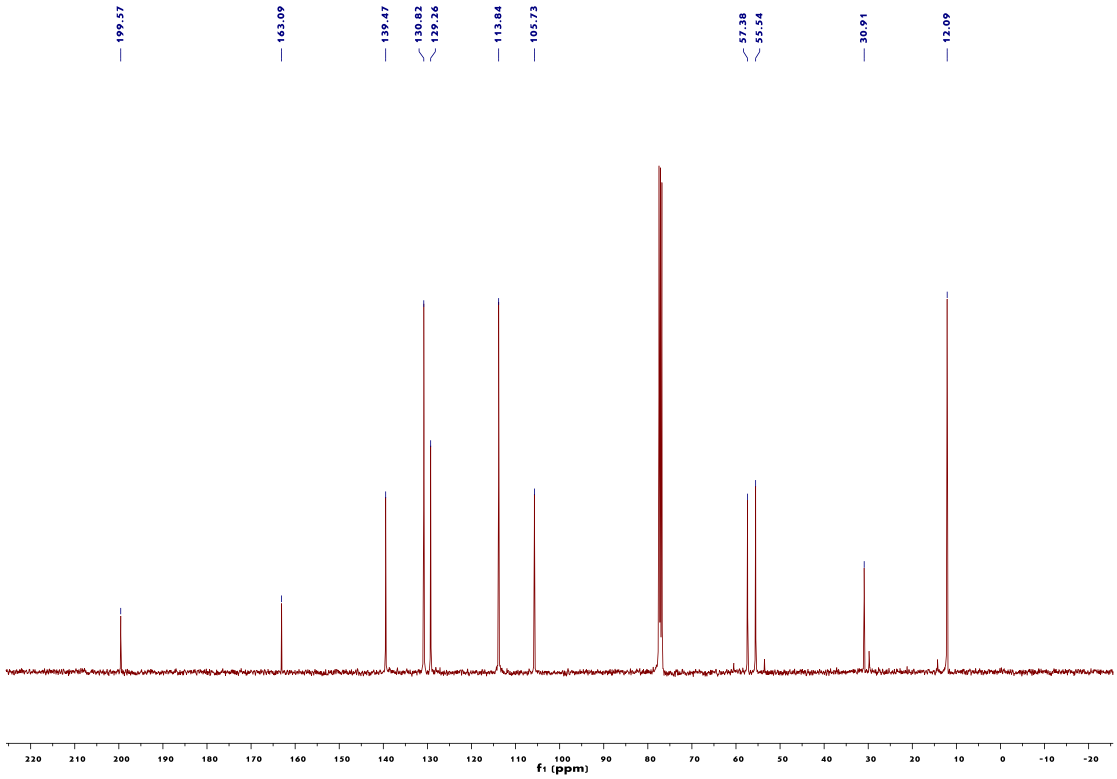

Supplementary Figure 151. ^{13}C NMR (100 MHz, $CDCl_3$) spectrum for 3xa

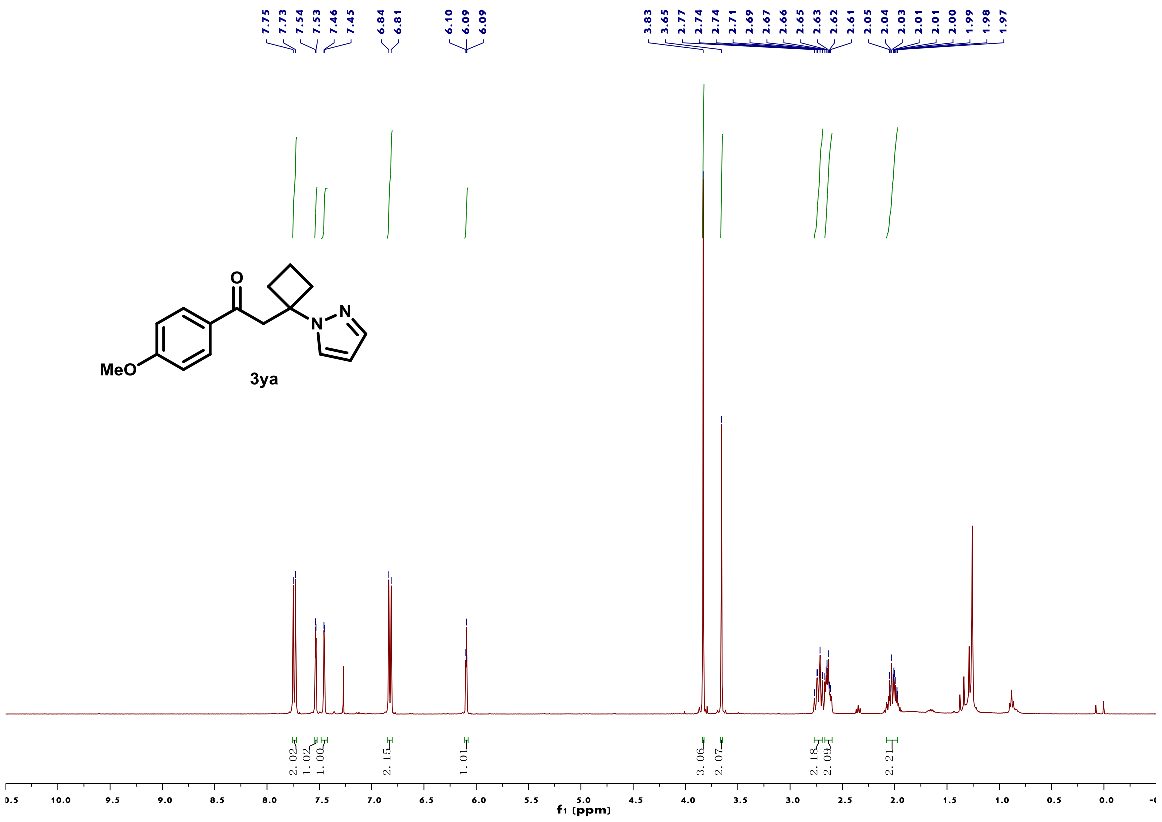

Supplementary Figure 152. ^{1}H NMR (400 MHz, $CDCl_3$) spectrum for 3ya

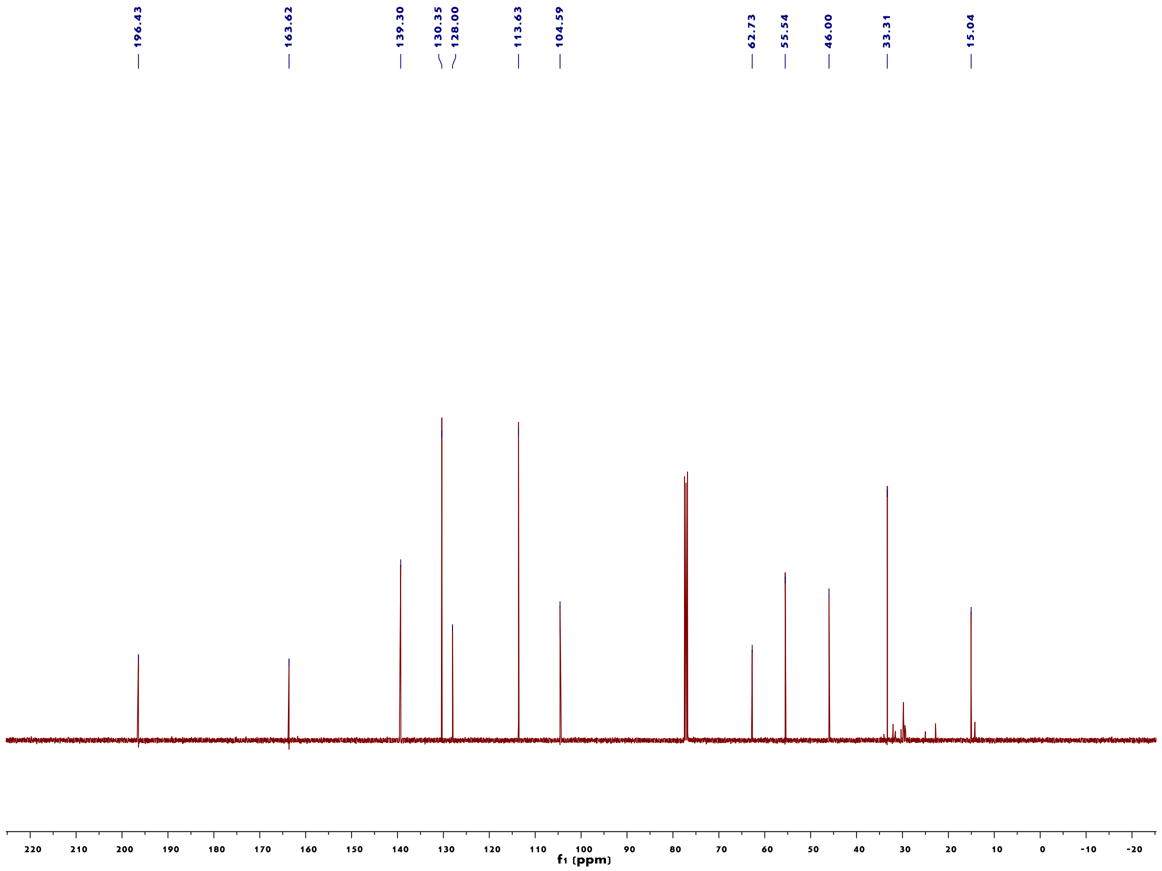

Supplementary Figure 153. ^{13}C NMR (100 MHz, $CDCl_3$) spectrum for 3ya

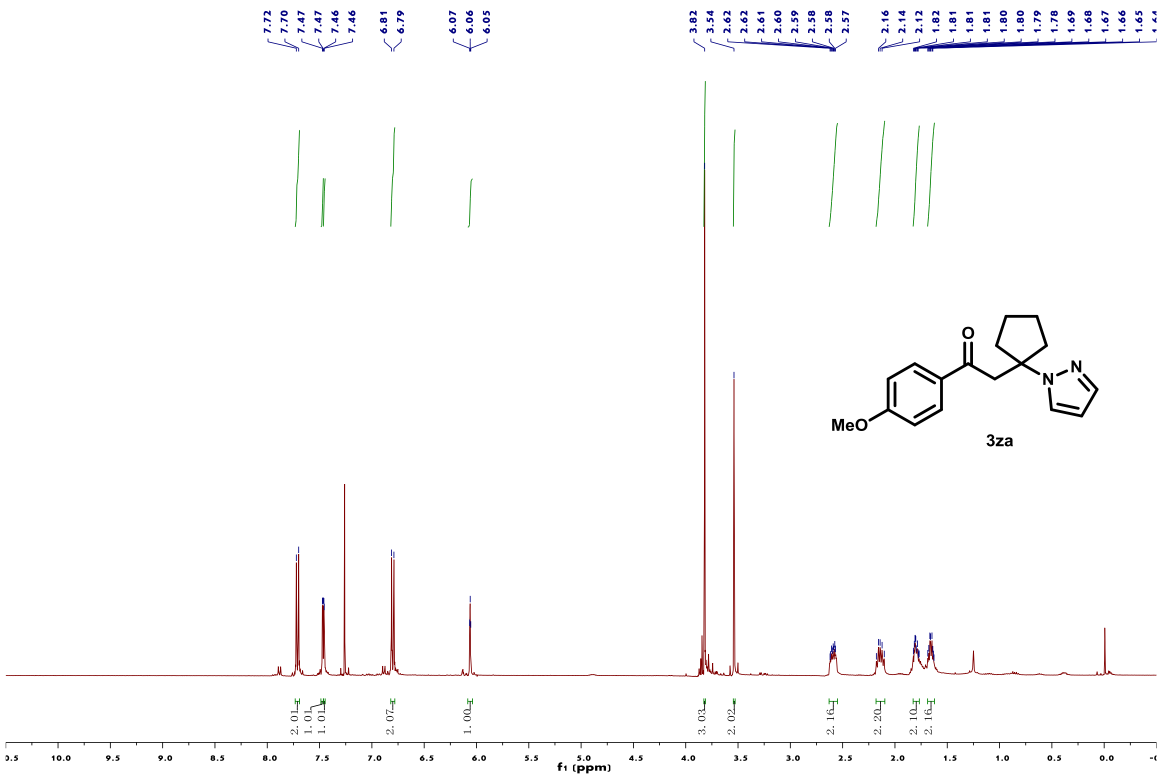

Supplementary Figure 154. ^{1}H NMR (400 MHz, $CDCl_3$) spectrum for 3za

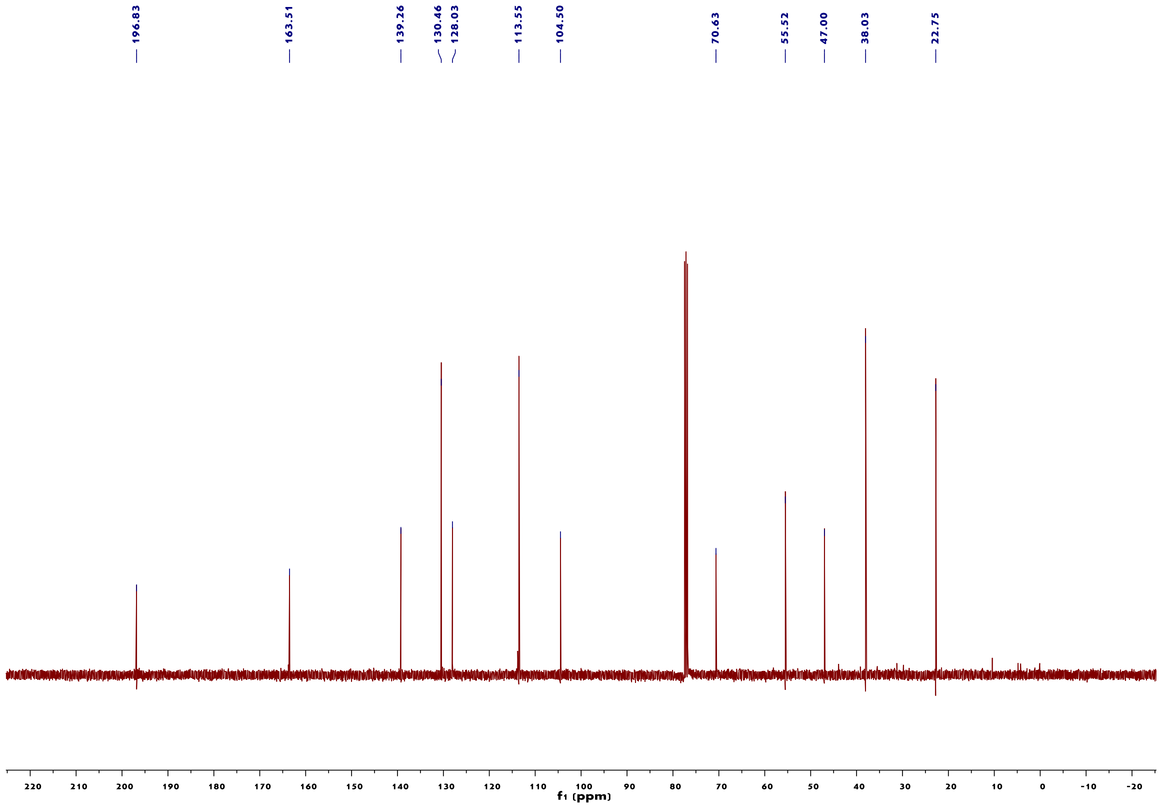

Supplementary Figure 155. ^{13}C NMR (100 MHz, $CDCl_3$) spectrum for 3za

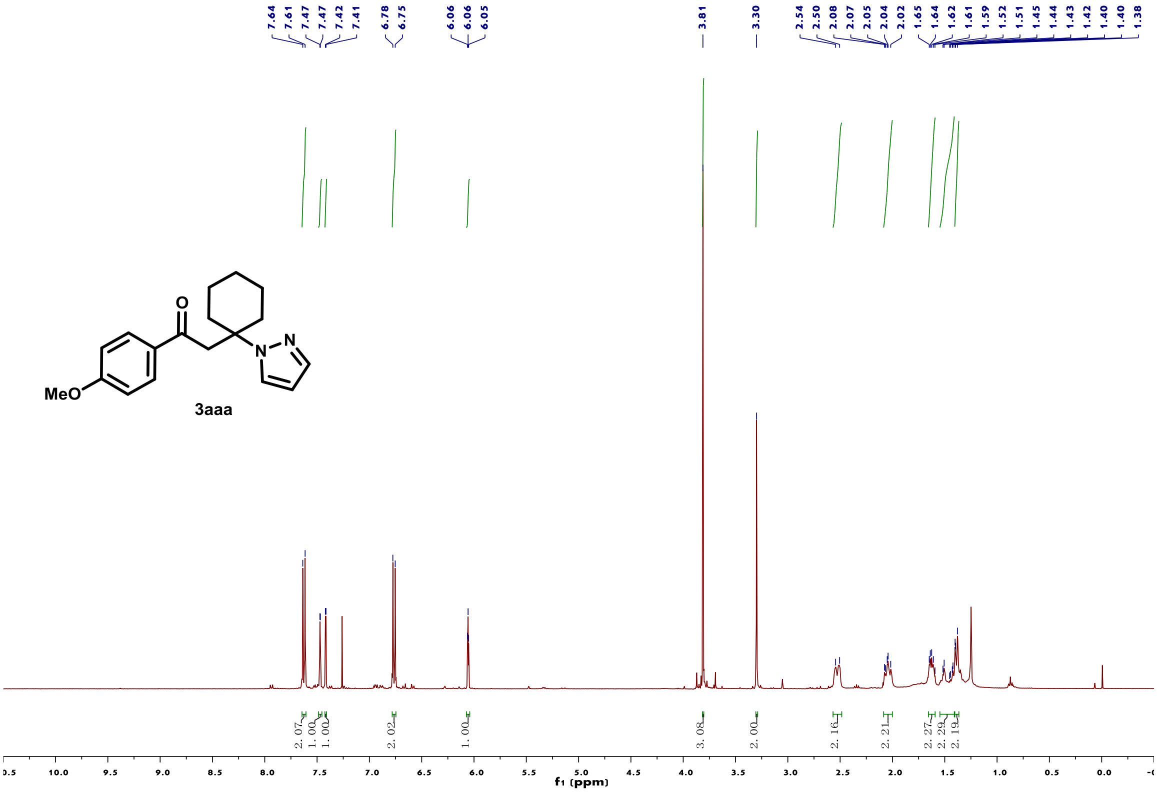

Supplementary Figure 156. ^{1}H **NMR (400 MHz,** $CDCl_3$**) spectrum for 3aaa**

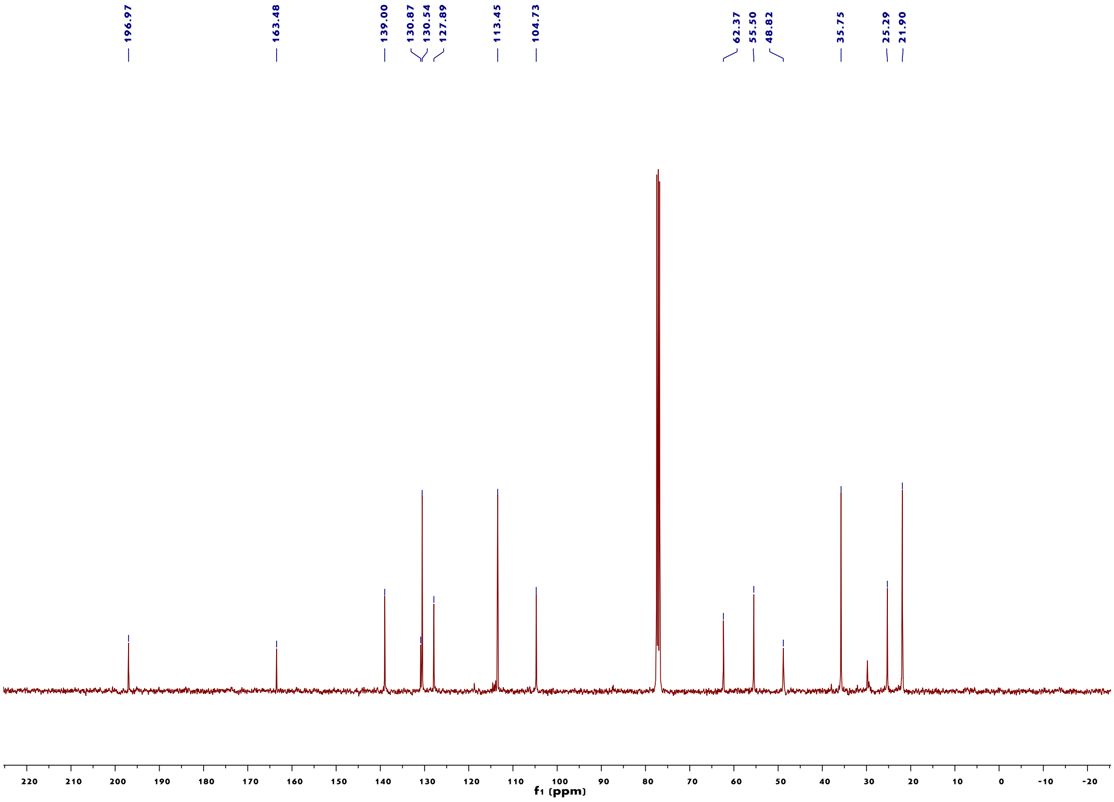

Supplementary Figure 157. ^{13}C **NMR (100 MHz,** $CDCl_3$**) spectrum for 3aaa**

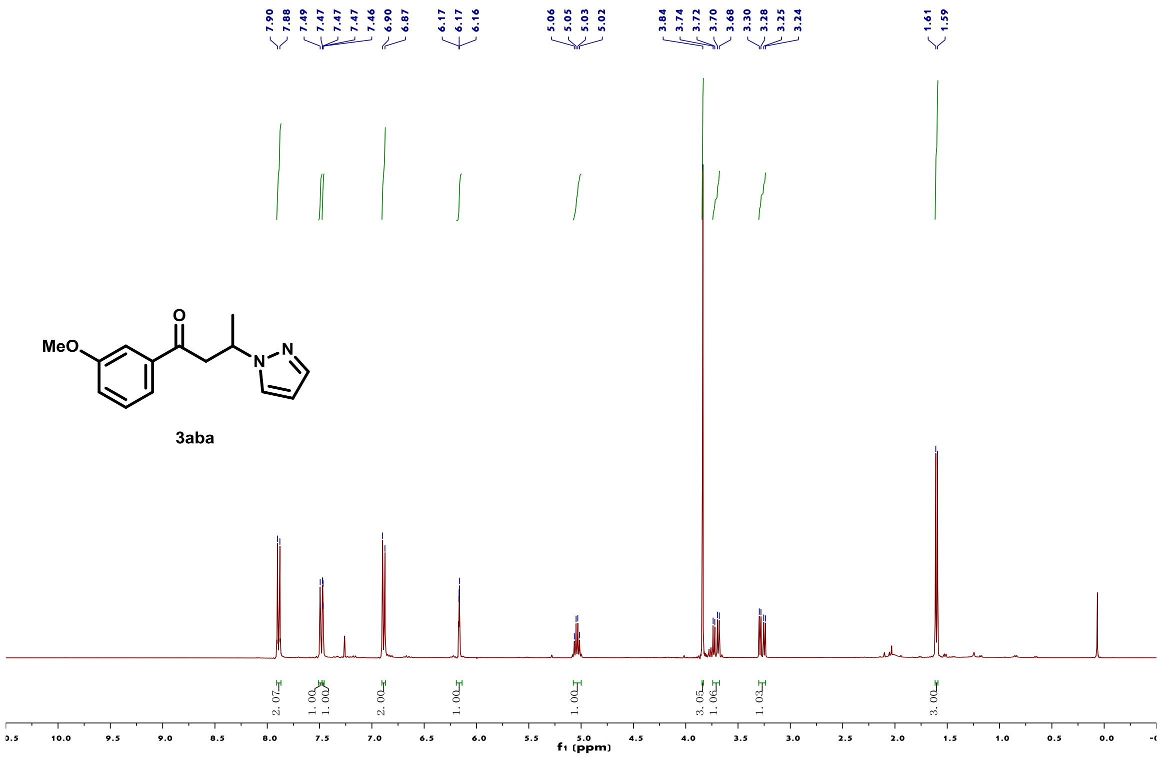

Supplementary Figure 158. ^{1}H NMR (400 MHz, $CDCl_3$) spectrum for 3aba

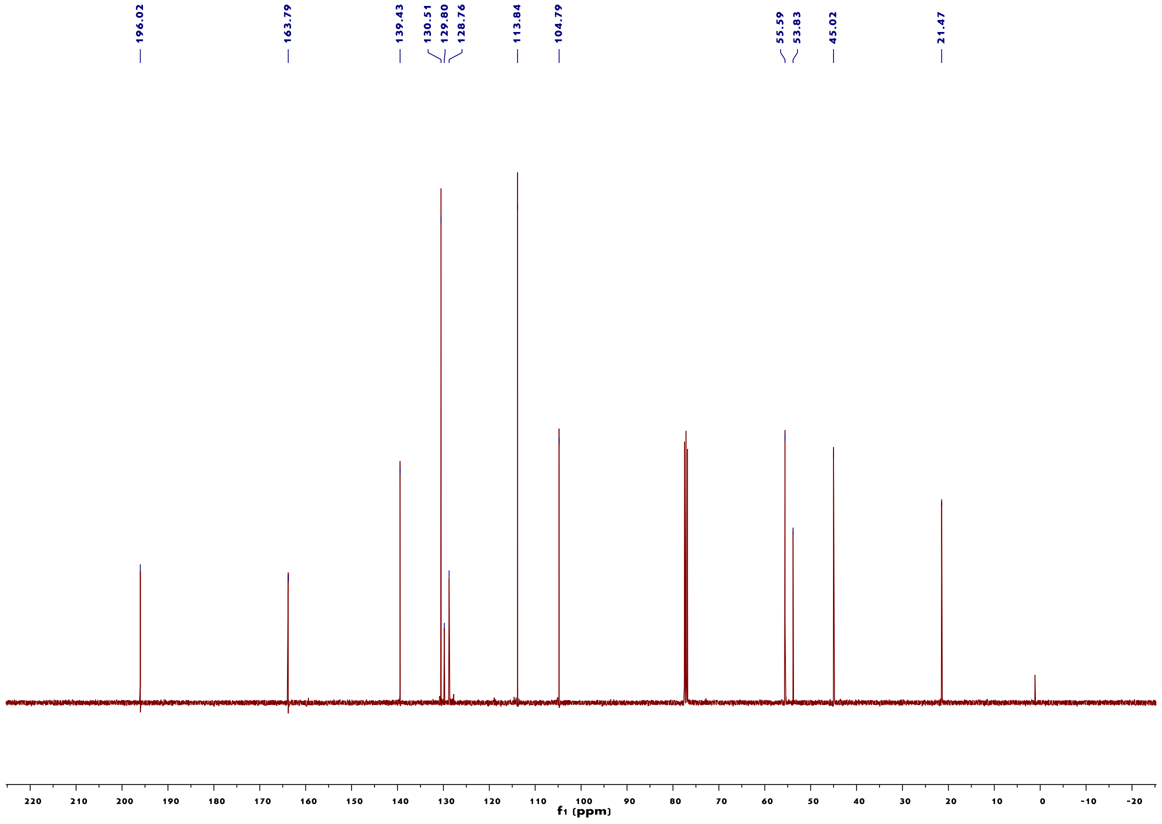

Supplementary Figure 159. ^{13}C NMR (100 MHz, $CDCl_3$) spectrum for 3aba

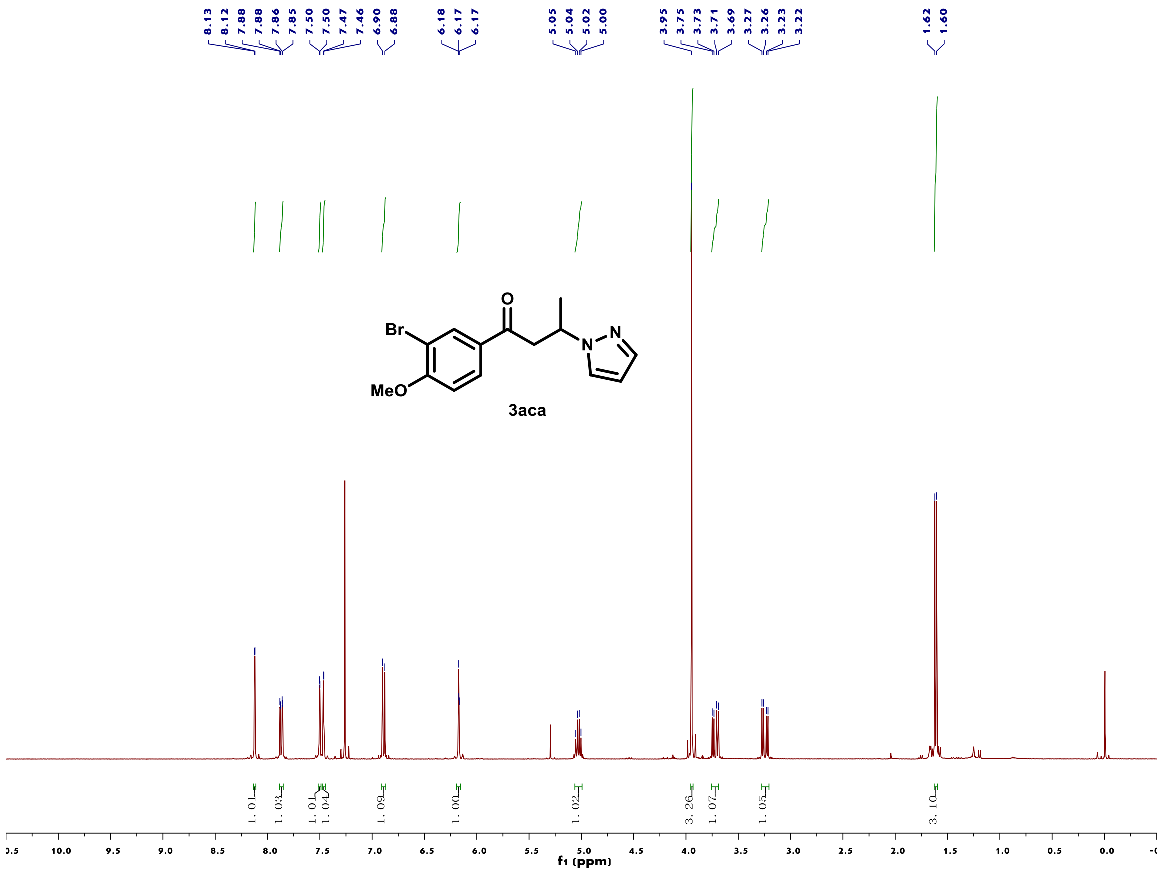

Supplementary Figure 160. ^{1}H NMR (400 MHz, $CDCl_3$) spectrum for 3aca

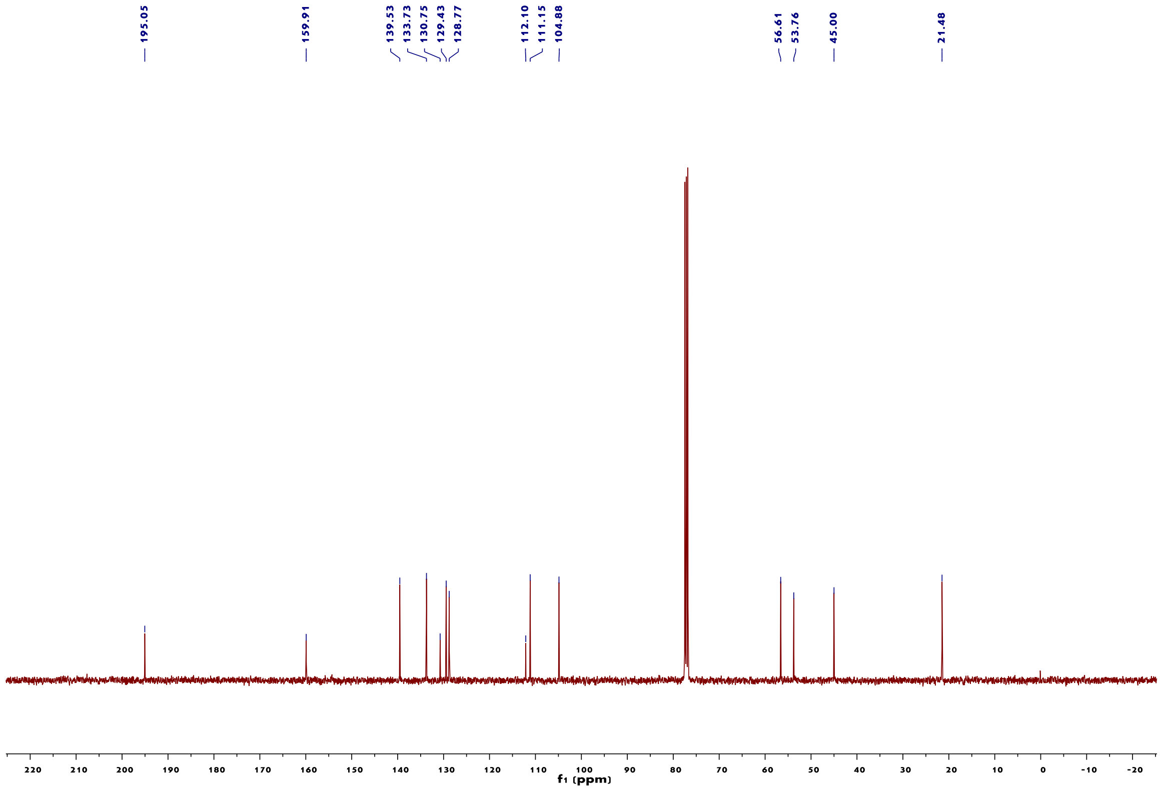

Supplementary Figure 161. ^{13}C NMR (100 MHz, $CDCl_3$) spectrum for 3aca

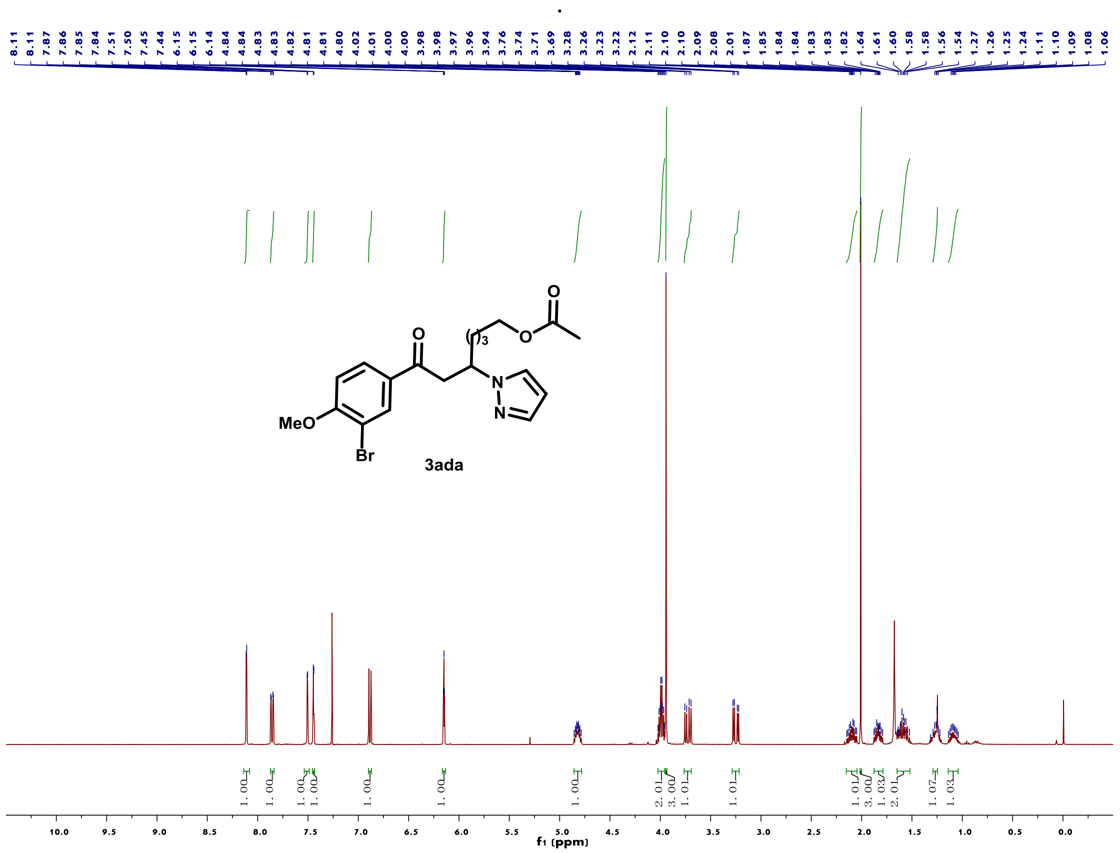

Supplementary Figure 162. ^{1}H NMR (400 MHz, $CDCl_3$) spectrum for 3ada

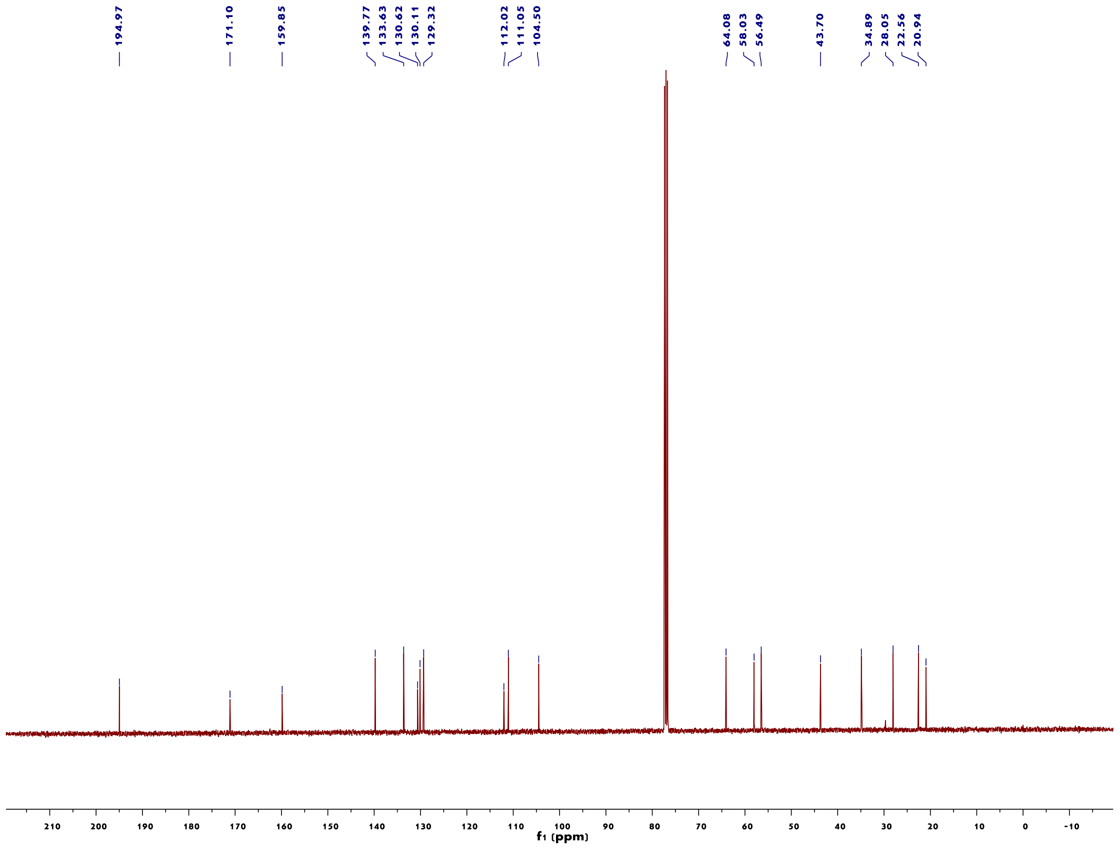

Supplementary Figure 163. ^{13}C NMR (100 MHz, $CDCl_3$) spectrum for 3ada

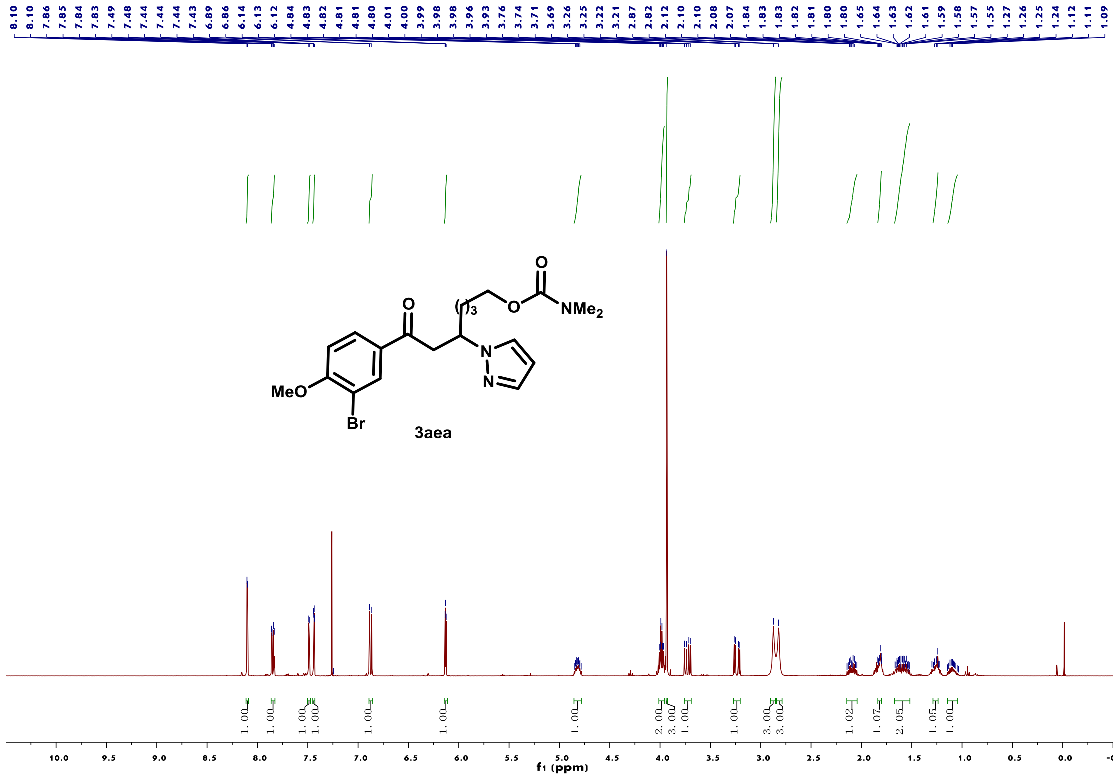

Supplementary Figure 164. ^{1}H **NMR (400 MHz,** $CDCl_3$**) spectrum for 3aea**

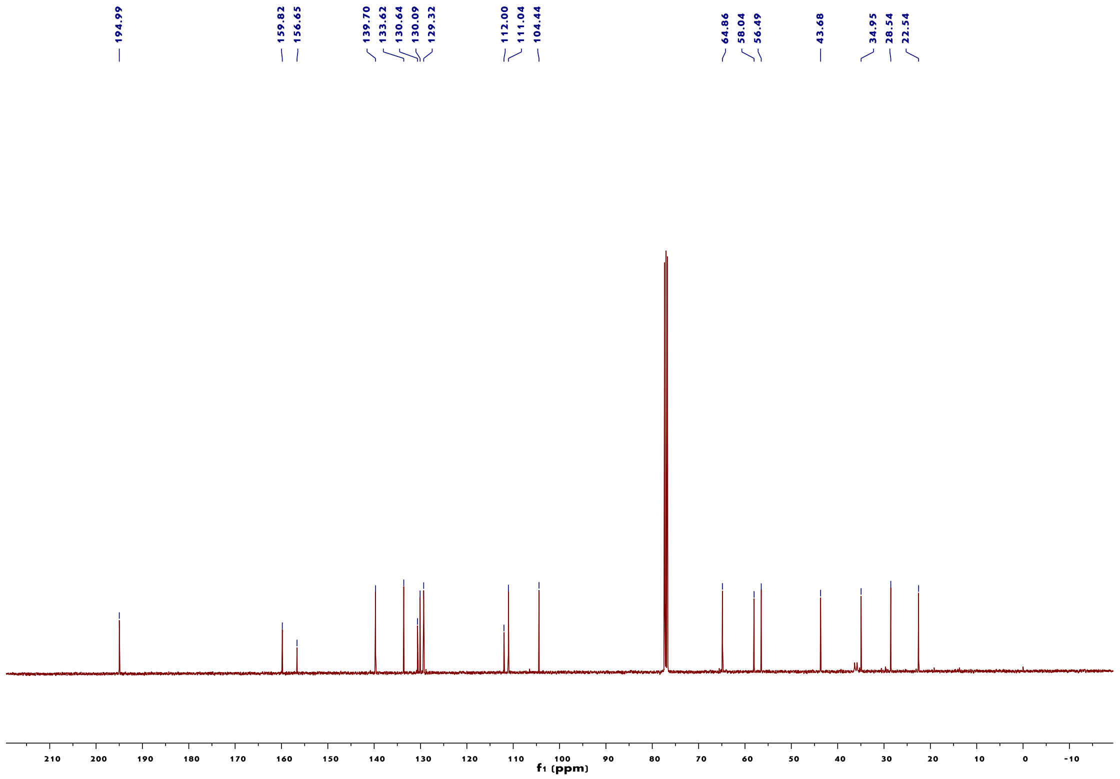

Supplementary Figure 165. ^{13}C **NMR (100 MHz,** $CDCl_3$**) spectrum for 3aea**

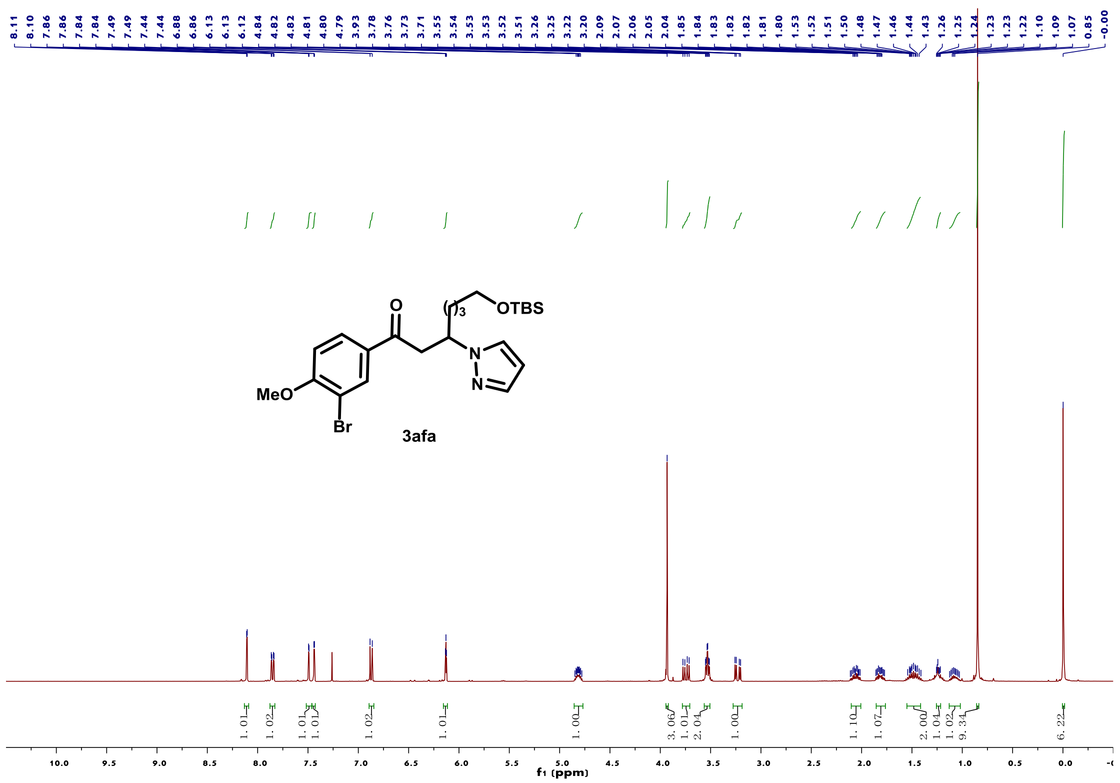

Supplementary Figure 166. ^{1}H NMR (400 MHz, $CDCl_3$) spectrum for 3afa

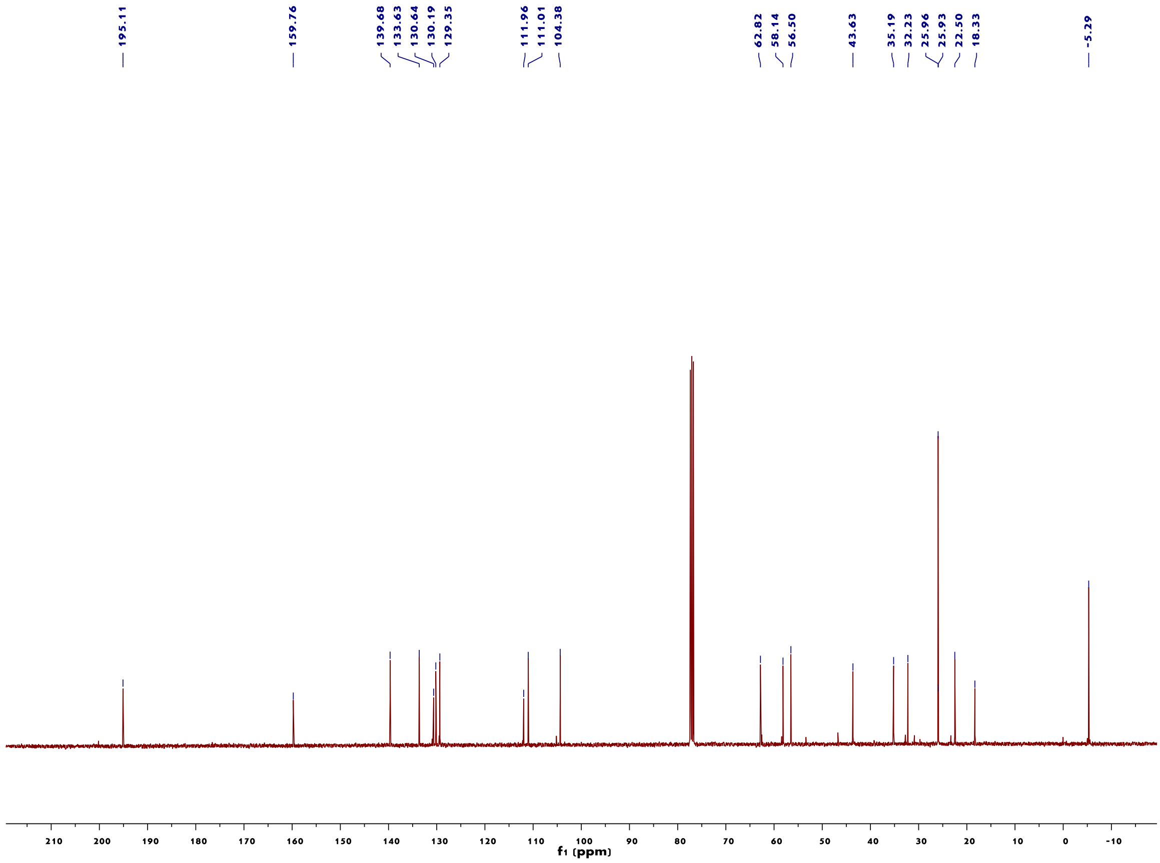

Supplementary Figure 167. ^{13}C NMR (100 MHz, $CDCl_3$) spectrum for 3afa

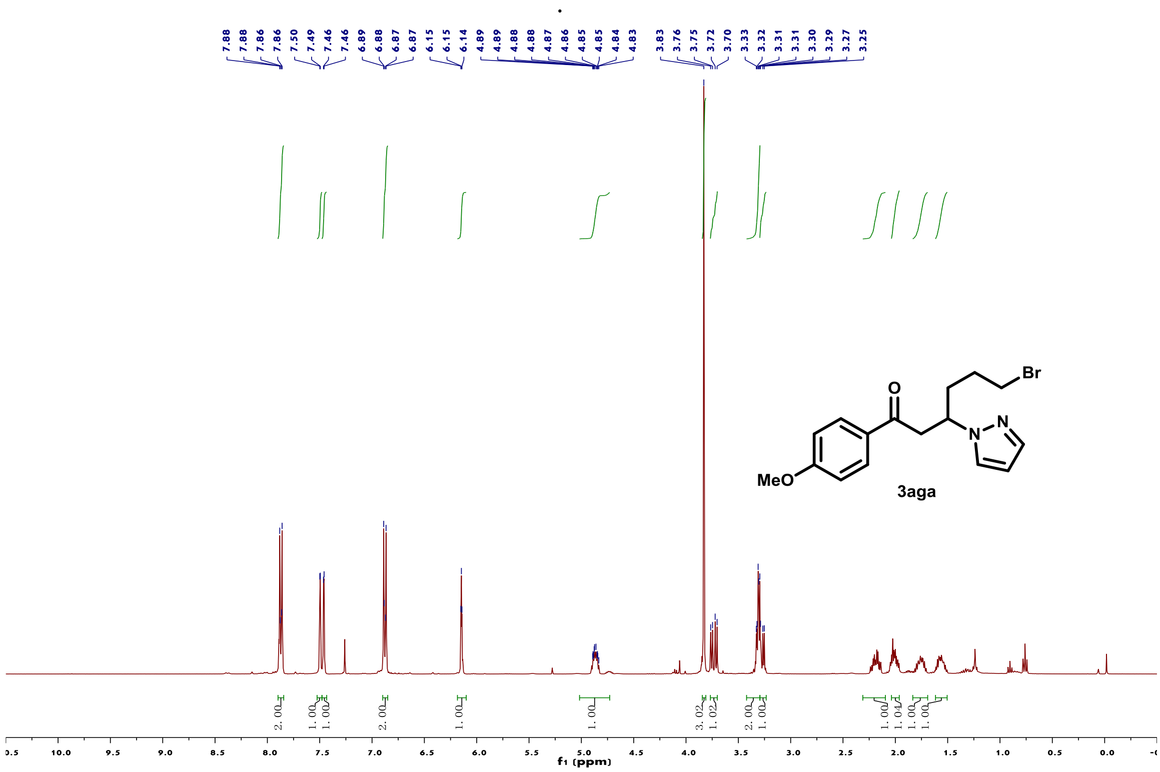

Supplementary Figure 168. ^{1}**H NMR (400 MHz,** $CDCl_3$**) spectrum for 3aga**

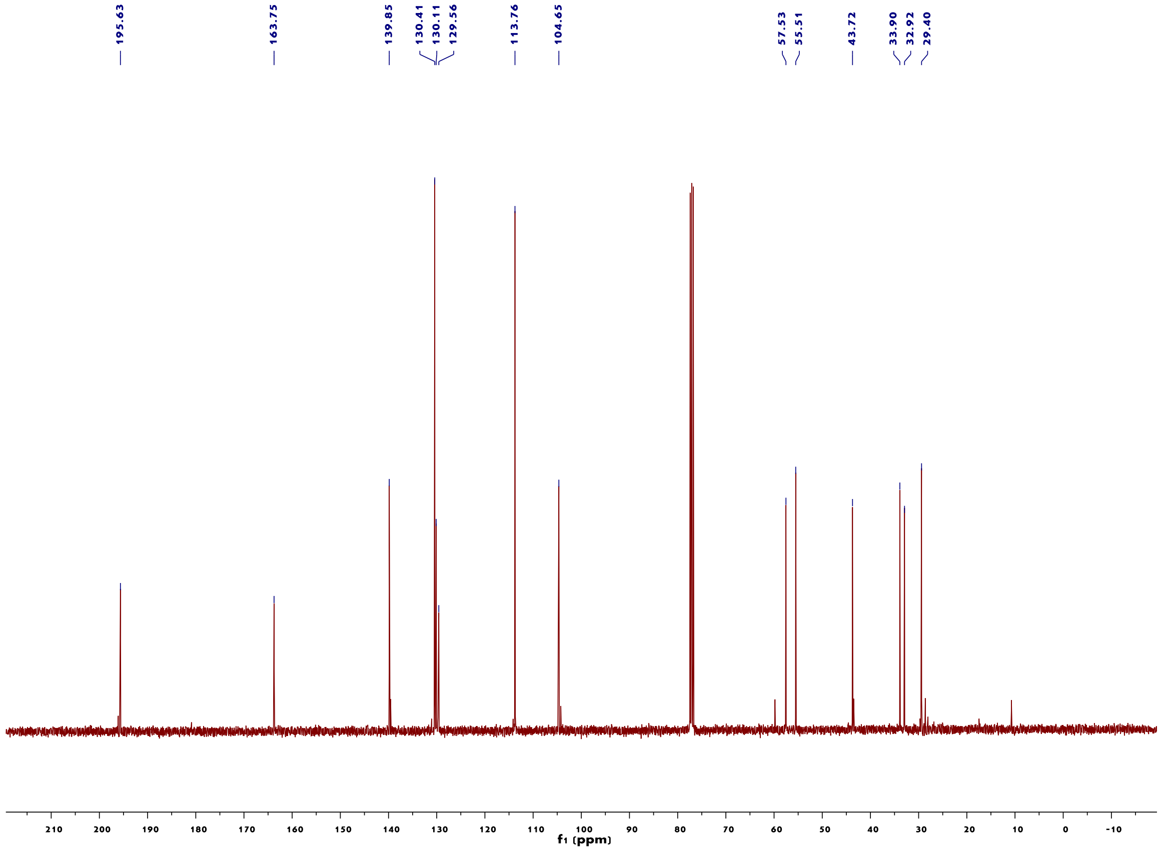

Supplementary Figure 169. ^{13}**C NMR (100 MHz,** $CDCl_3$**) spectrum for 3aga**

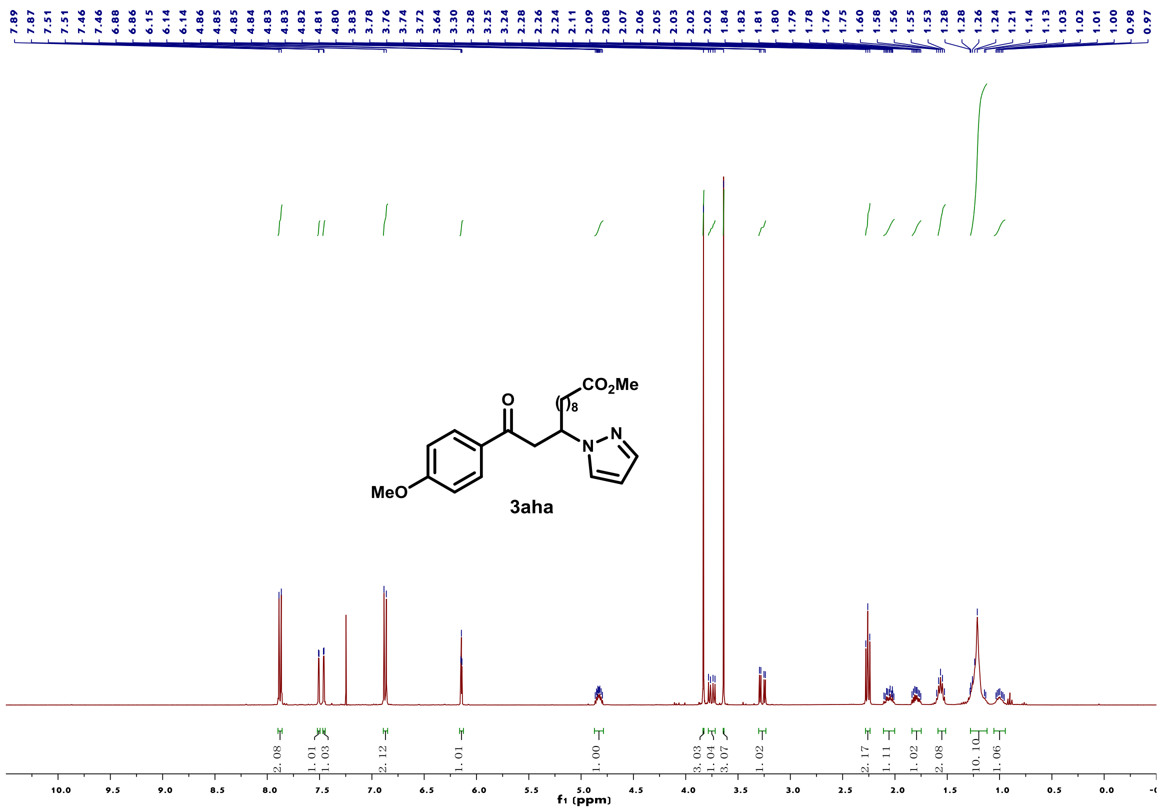

Supplementary Figure 170. ^{1}H NMR (400 MHz, CDCl$_3$) spectrum for 3aha

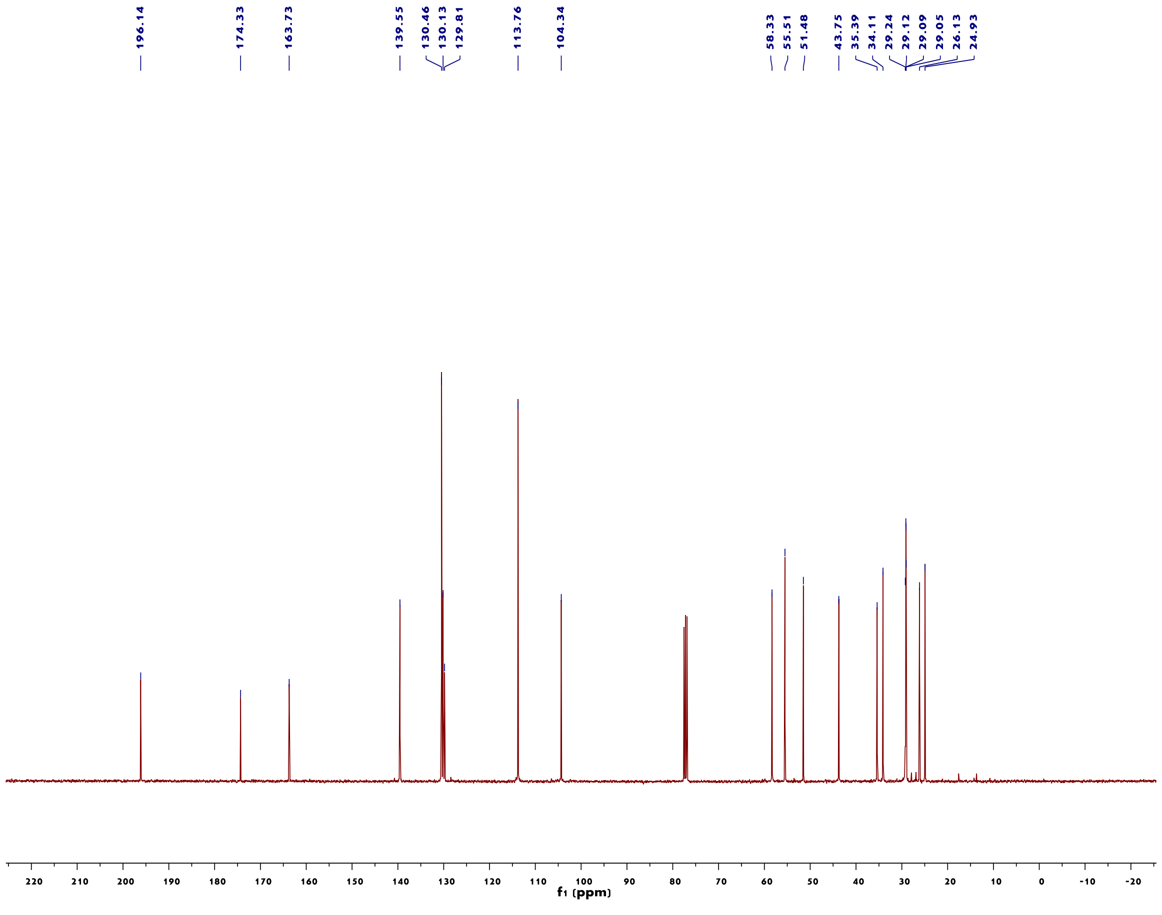

Supplementary Figure 171. ^{13}C NMR (100 MHz, CDCl$_3$) spectrum for 3aha

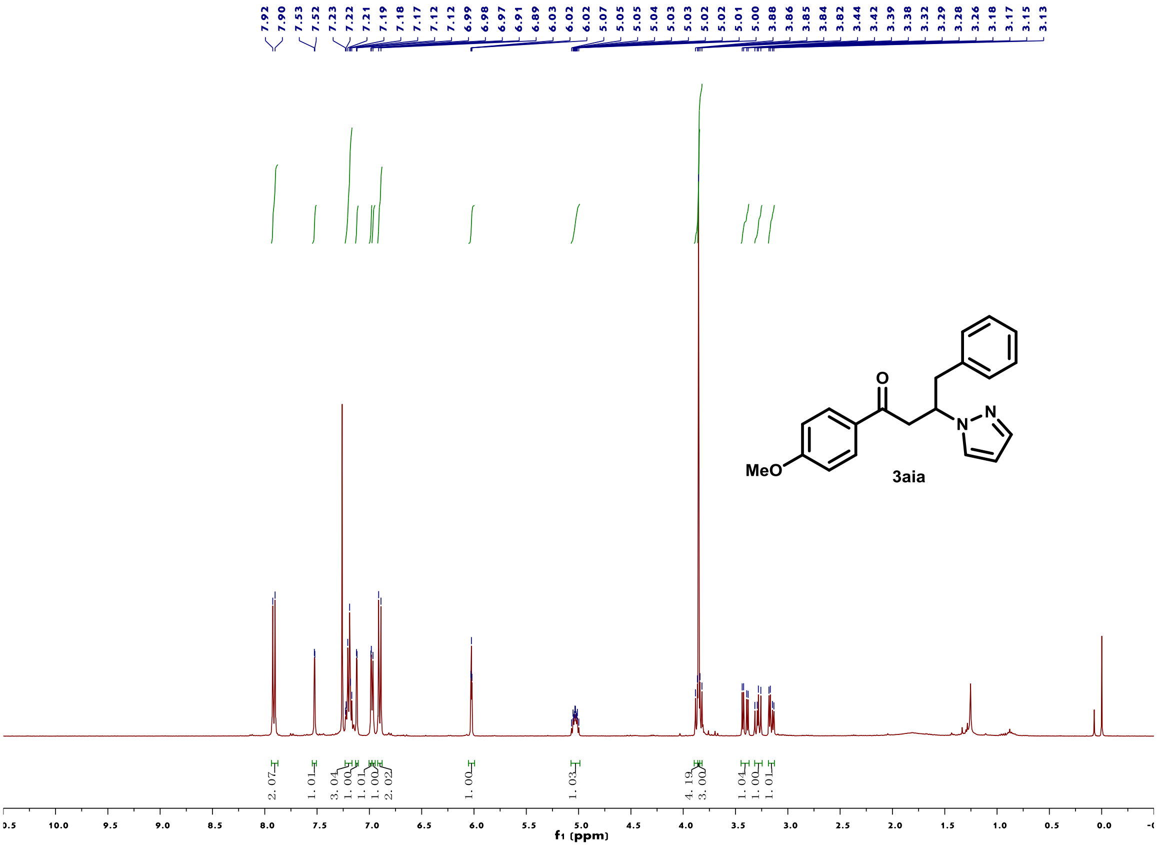

Supplementary Figure 172. ^{1}H NMR (400 MHz, $CDCl_3$) spectrum for 3aia

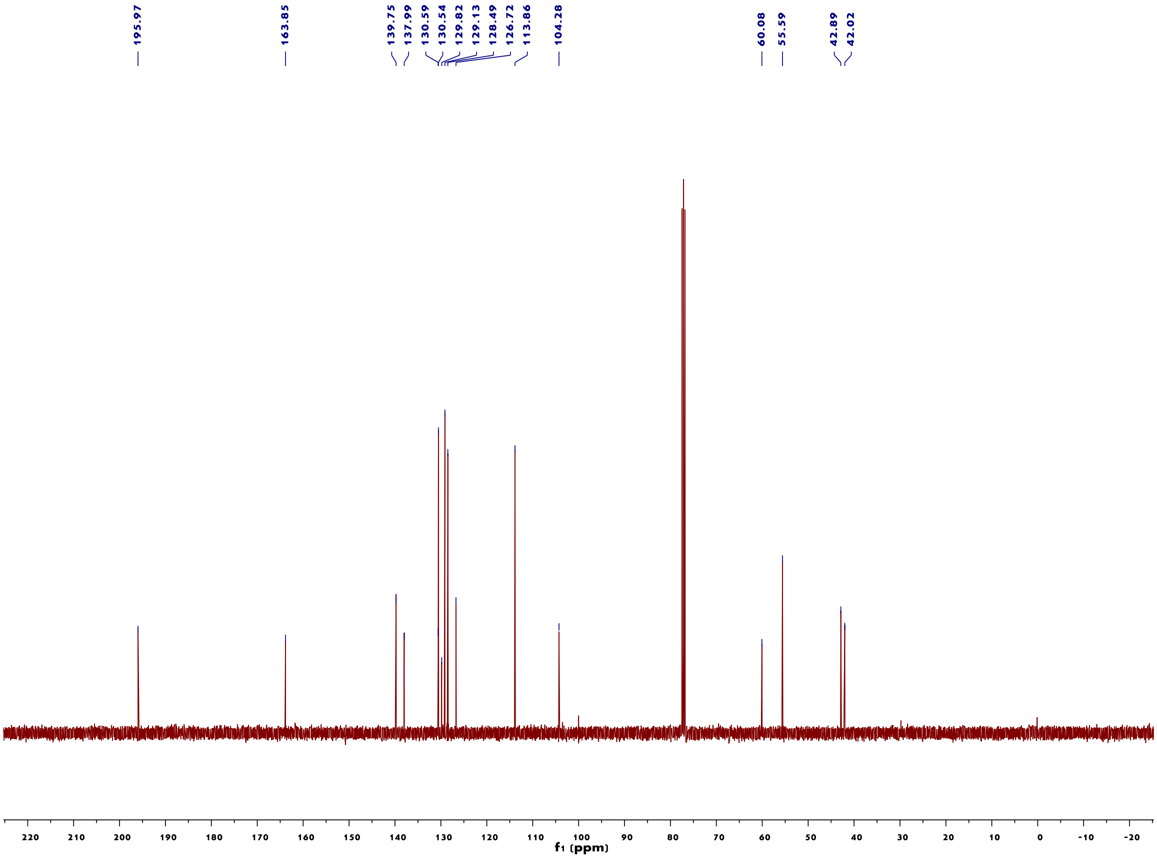

Supplementary Figure 173. ^{13}C NMR (100 MHz, $CDCl_3$) spectrum for 3aia

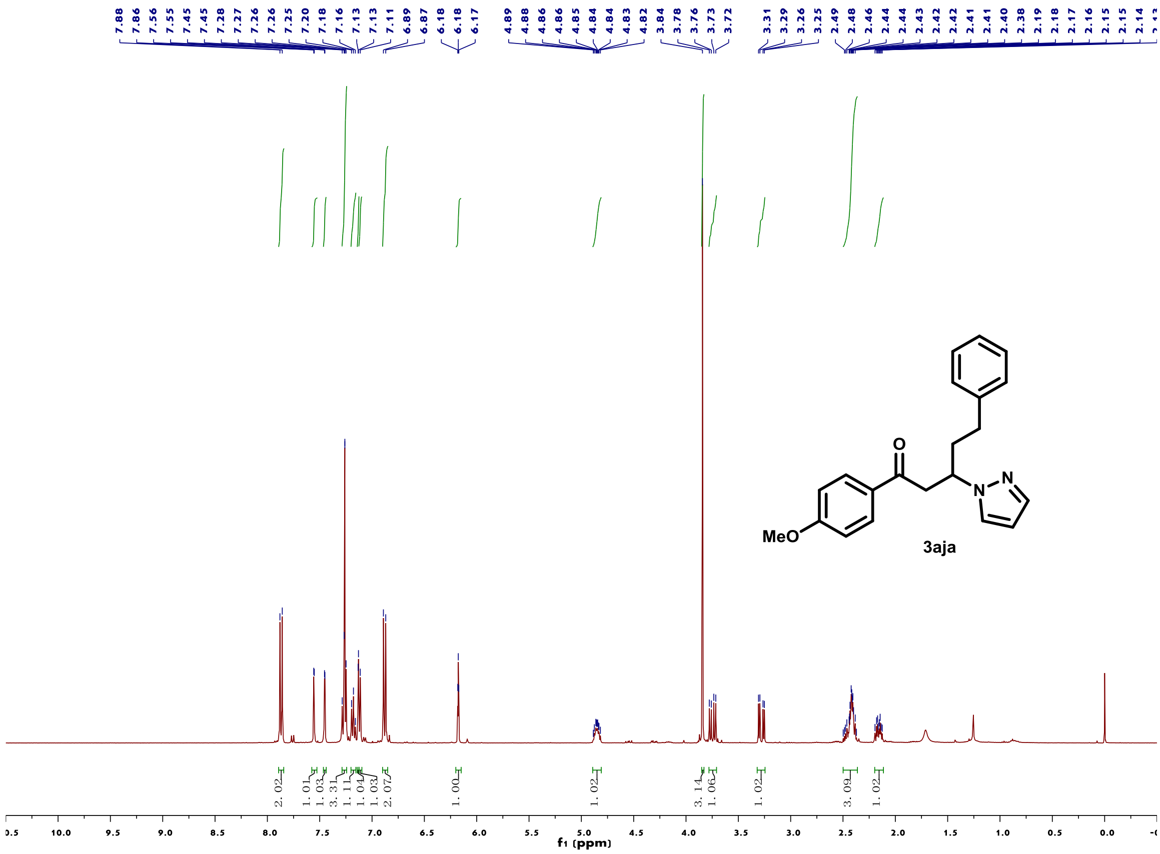

Supplementary Figure 174. ^{1}H NMR (400 MHz, $CDCl_3$) spectrum for 3aja

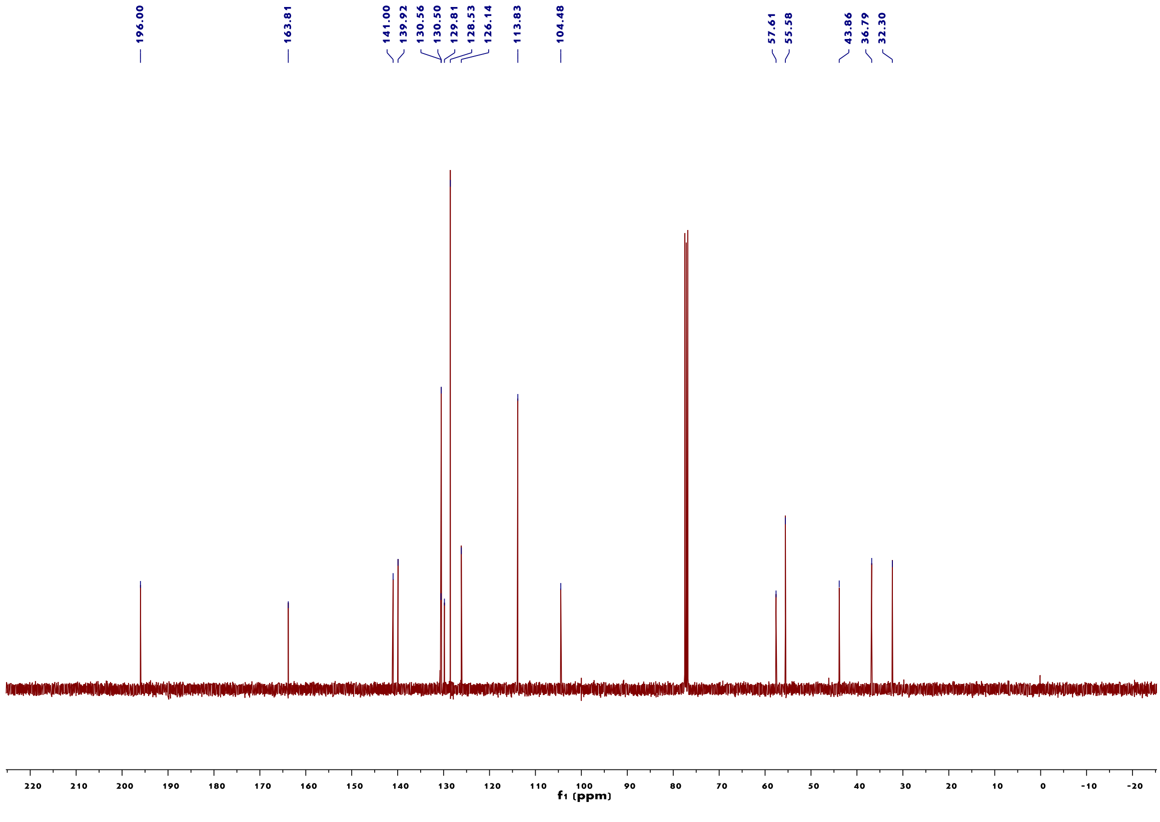

Supplementary Figure 175. ^{13}C NMR (100 MHz, $CDCl_3$) spectrum for 3aja

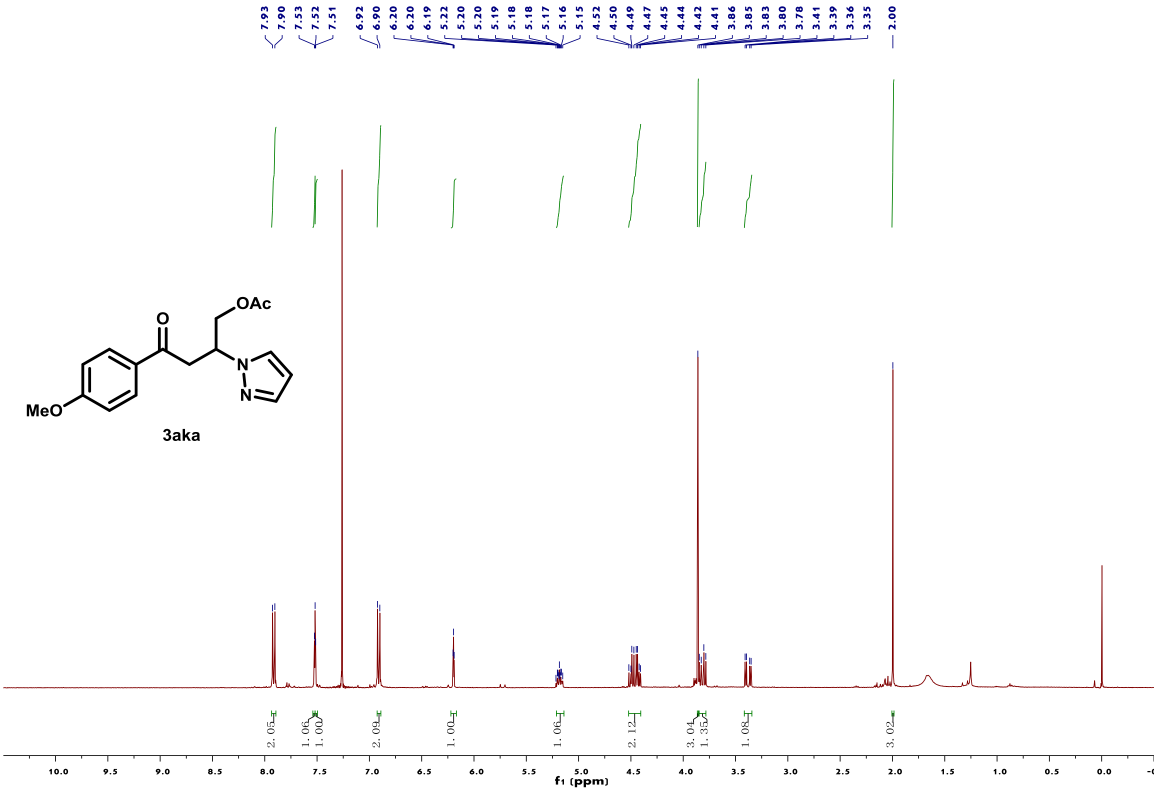

Supplementary Figure 176. ^{1}H NMR (400 MHz, $CDCl_3$) spectrum for 3aka

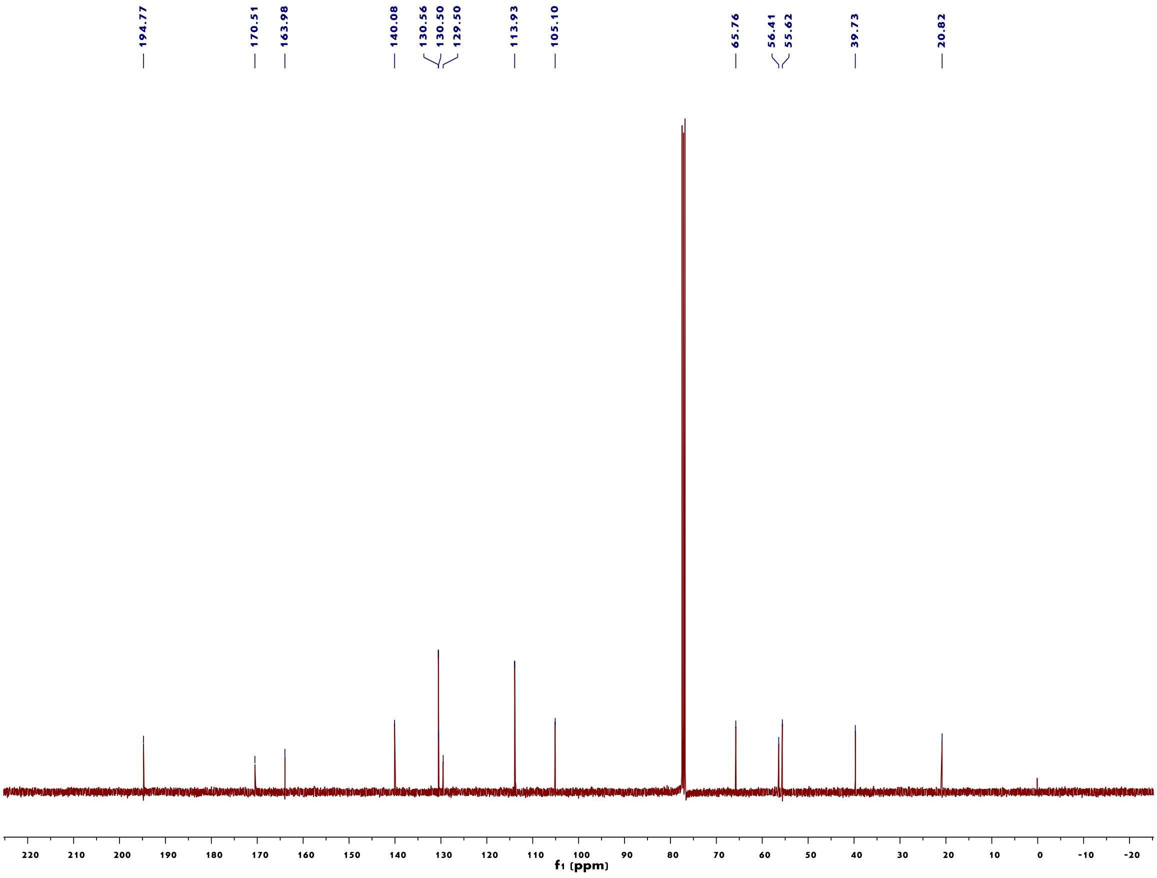

Supplementary Figure 177. ^{13}C NMR (100 MHz, $CDCl_3$) spectrum for 3aka

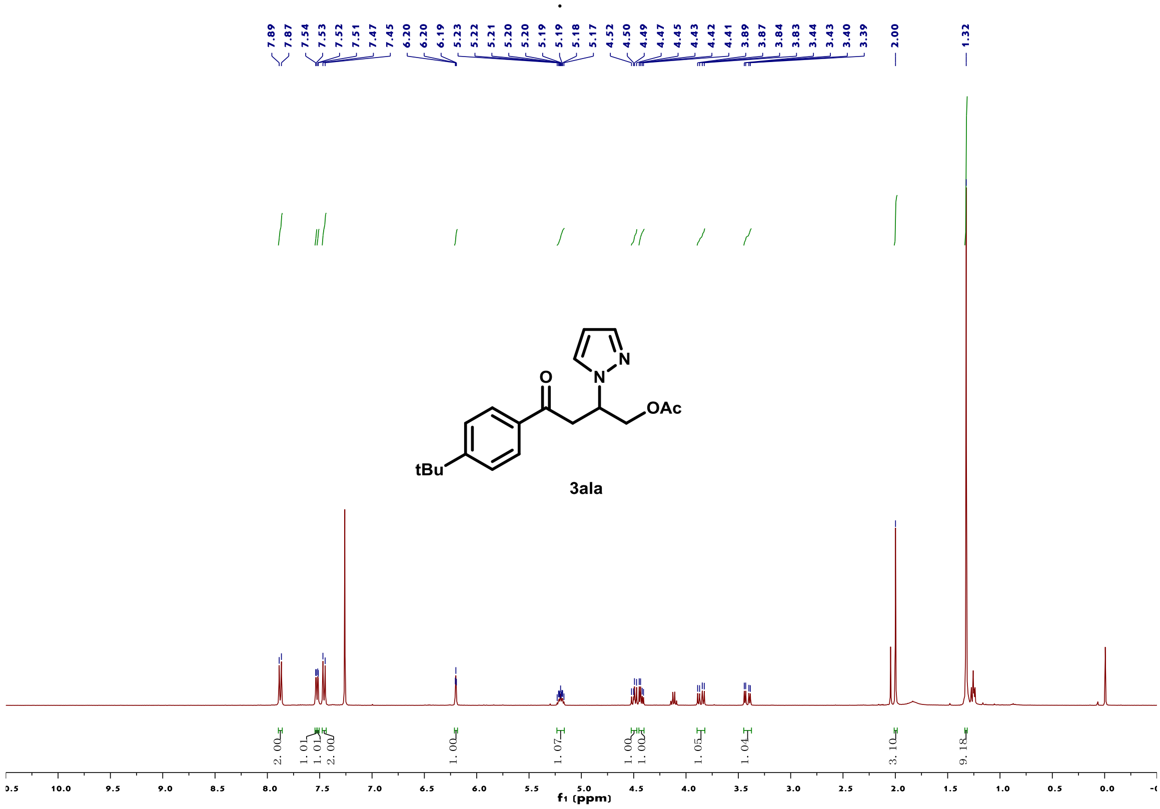

Supplementary Figure 178. ^{1}H NMR (400 MHz, $CDCl_3$) spectrum for 3ala

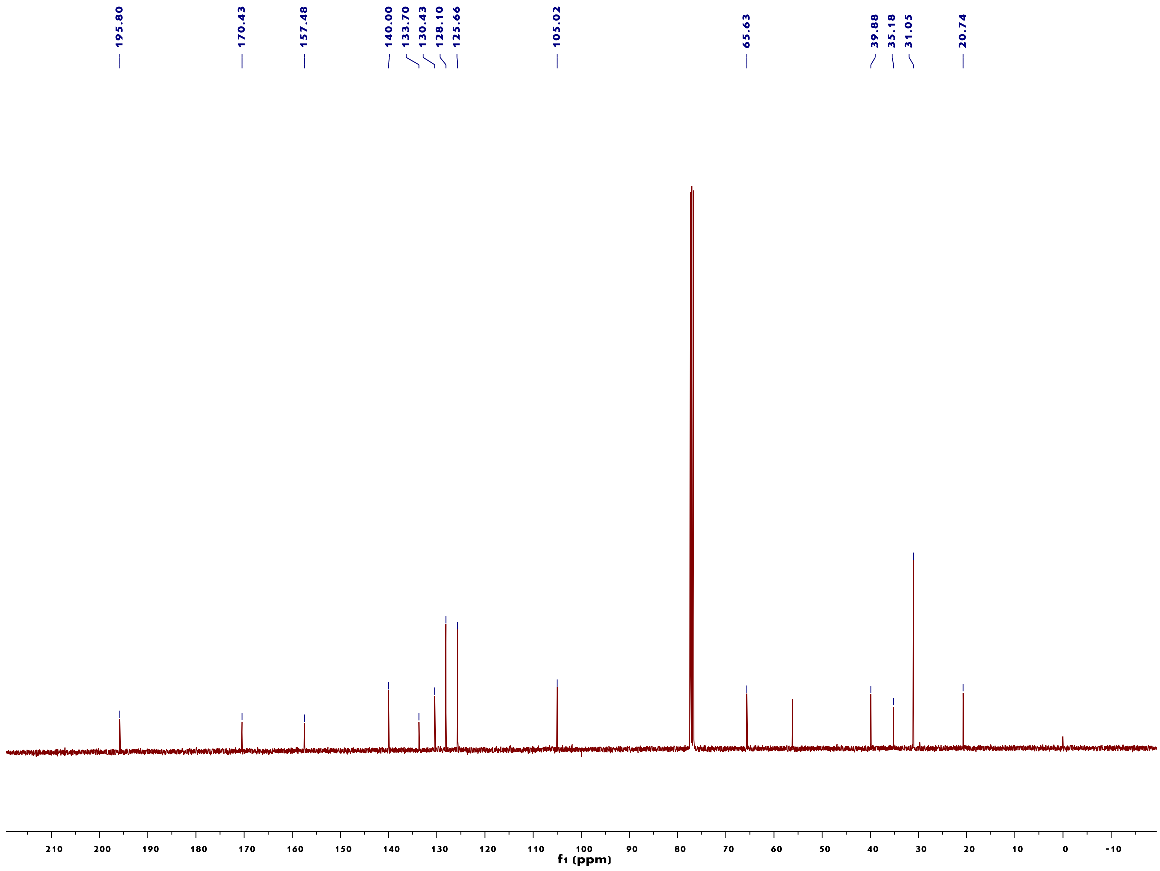

Supplementary Figure 179. ^{13}C NMR (100 MHz, $CDCl_3$) spectrum for 3ala

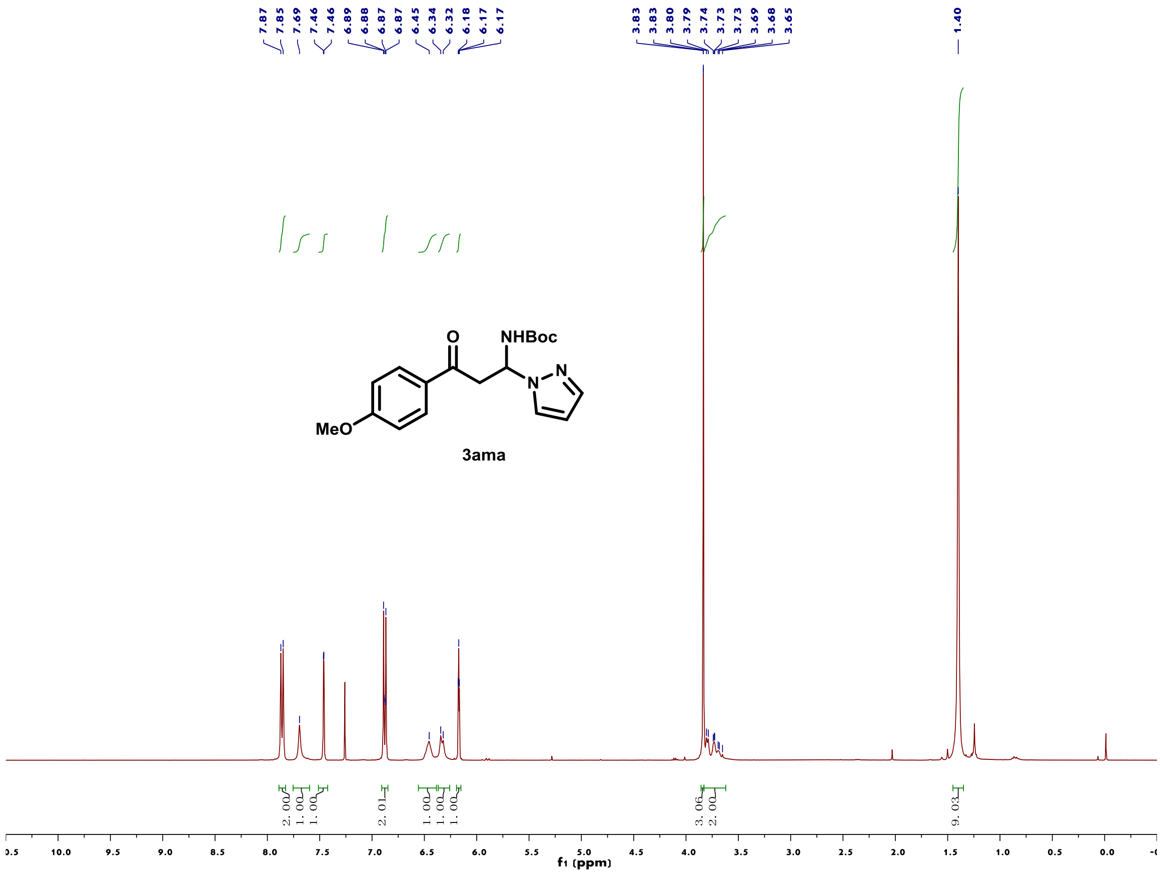

Supplementary Figure 180. ^{1}H NMR (400 MHz, $CDCl_3$) spectrum for 3ama

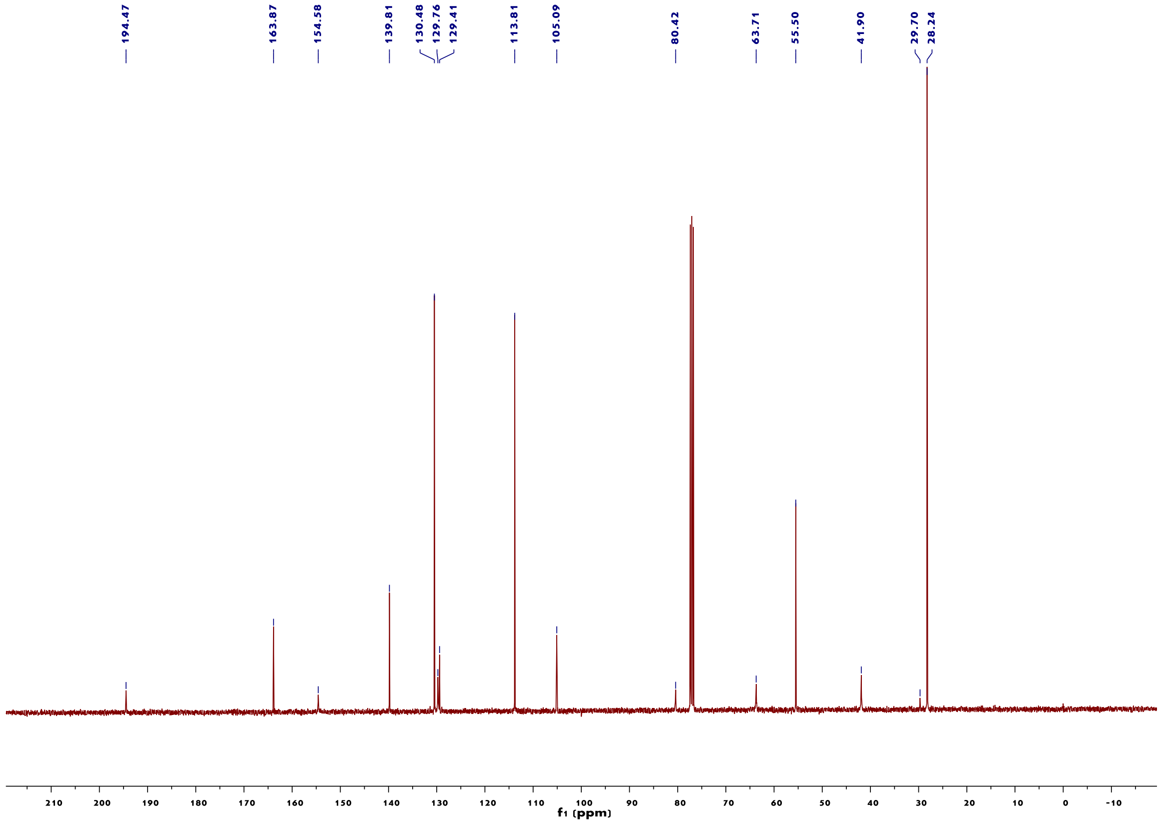

Supplementary Figure 181. ^{13}C NMR (100 MHz, $CDCl_3$) spectrum for 3ama

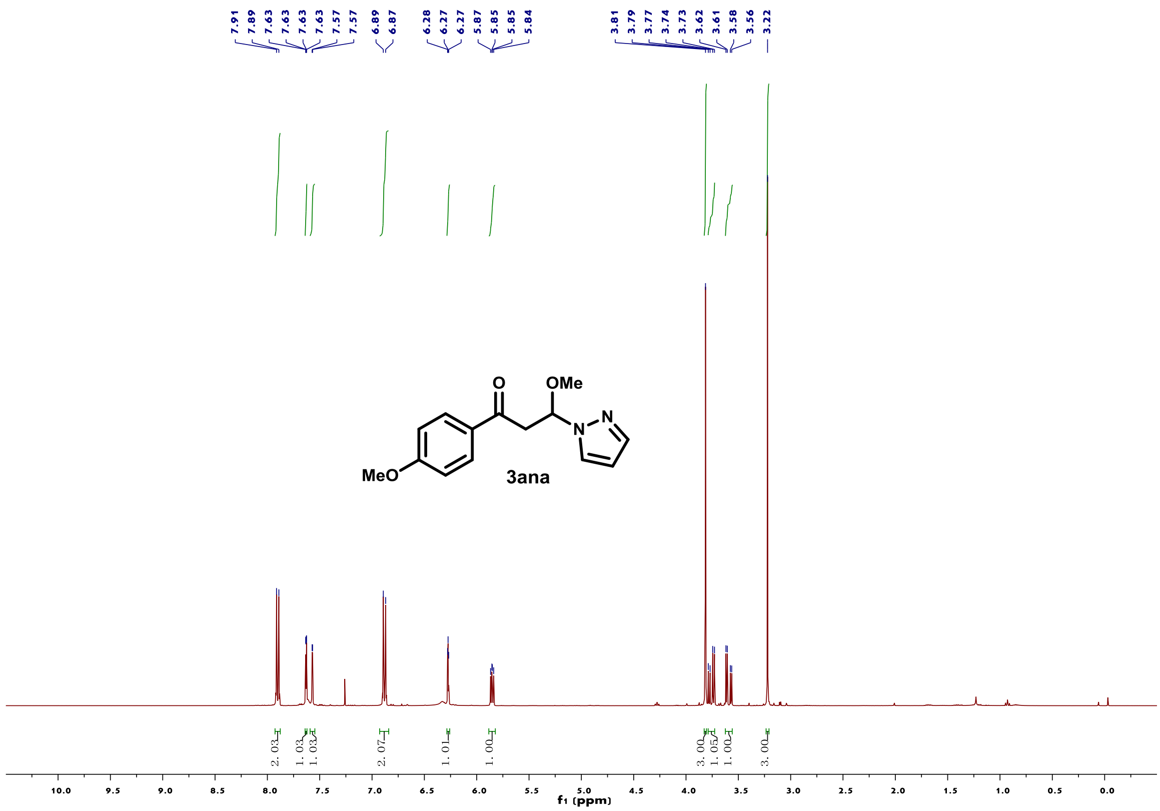

Supplementary Figure 182. ^{1}H NMR (400 MHz, $CDCl_3$) spectrum for 3ana

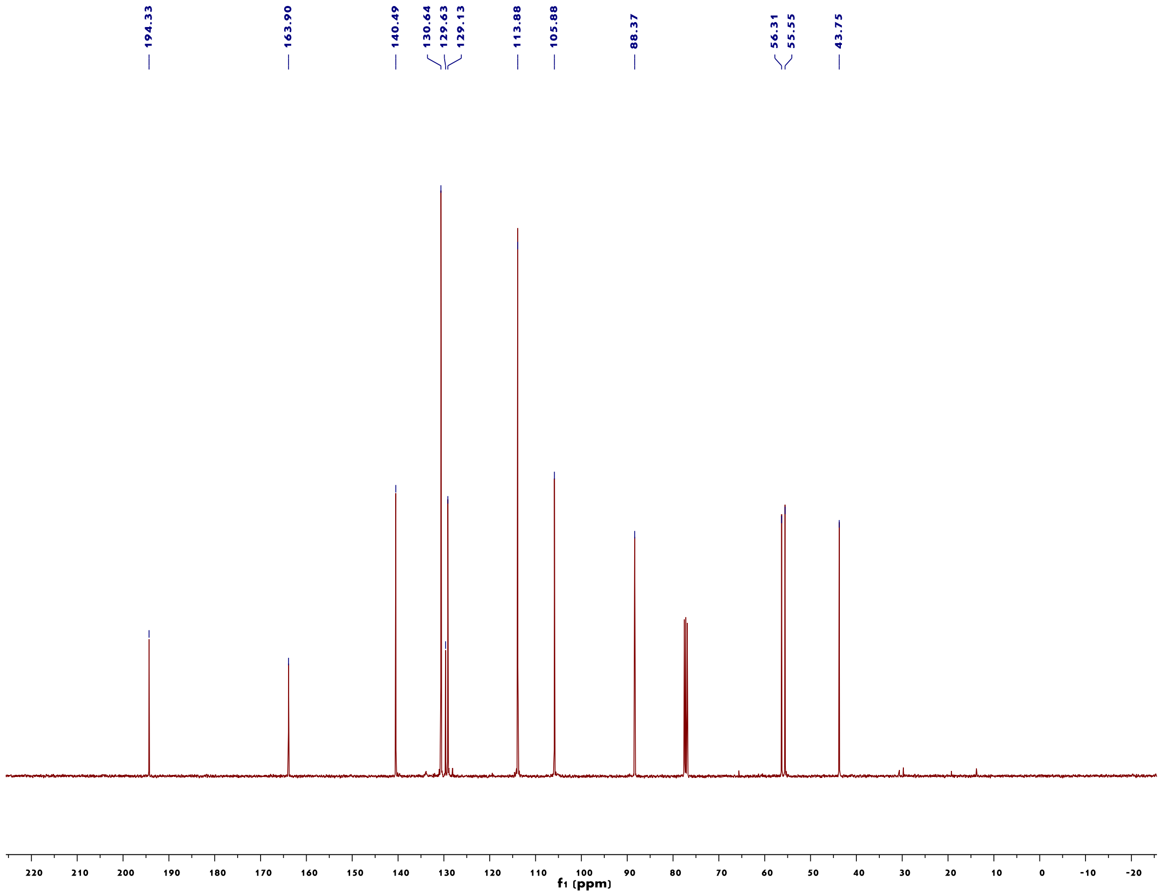

Supplementary Figure 183. ^{13}C NMR (100 MHz, $CDCl_3$) spectrum for 3ana

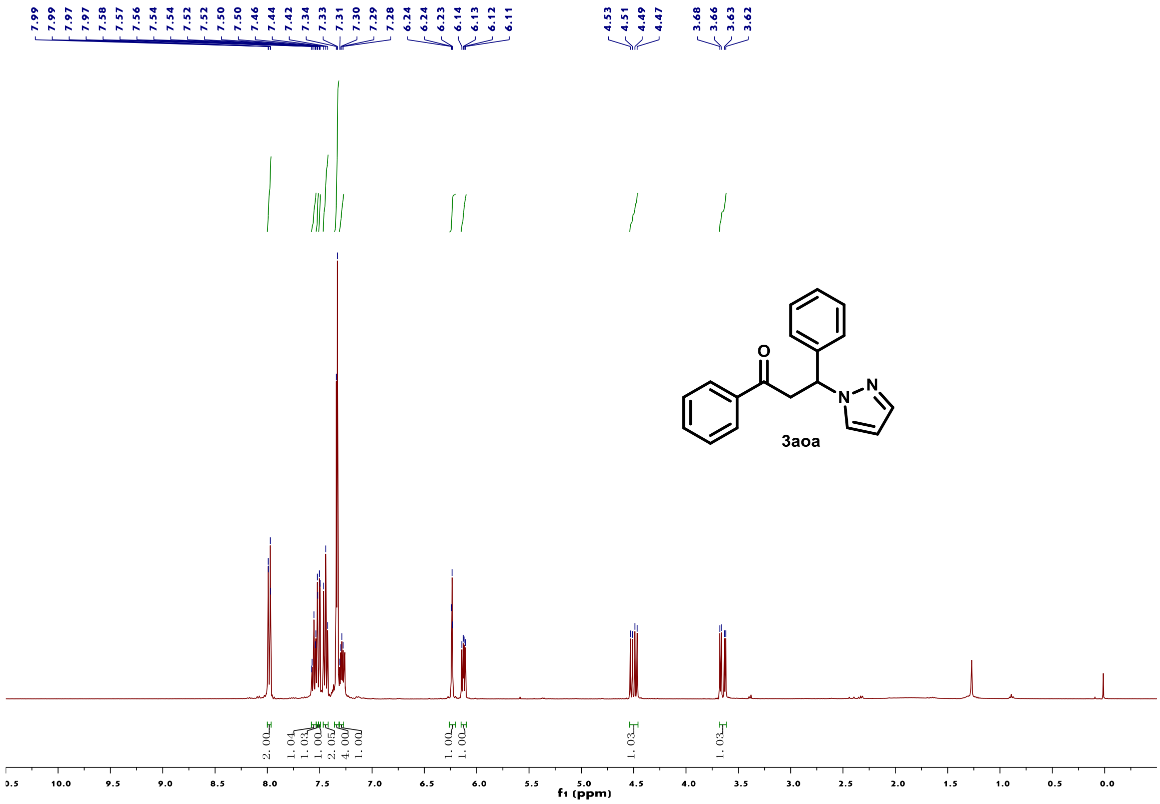

Supplementary Figure 184. ^{1}H NMR (400 MHz, $CDCl_3$) spectrum for 3aoa

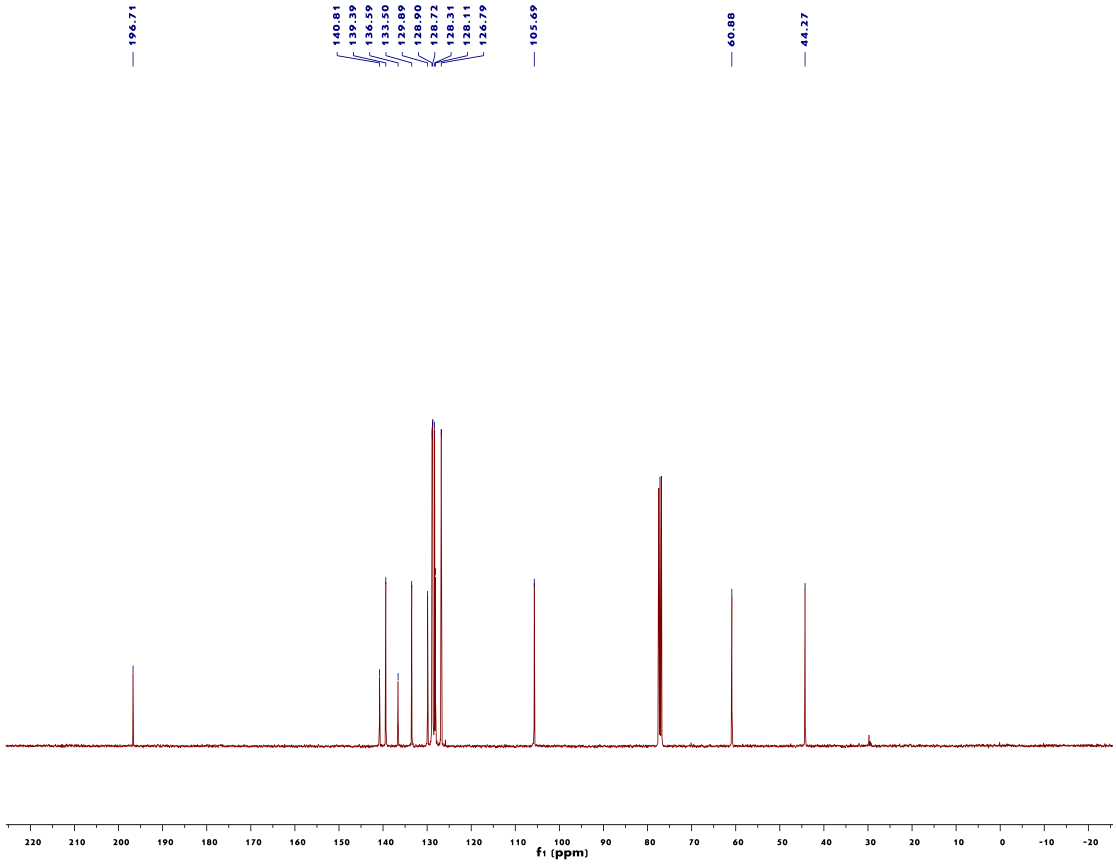

Supplementary Figure 185. ^{13}C NMR (100 MHz, $CDCl_3$) spectrum for 3aoa

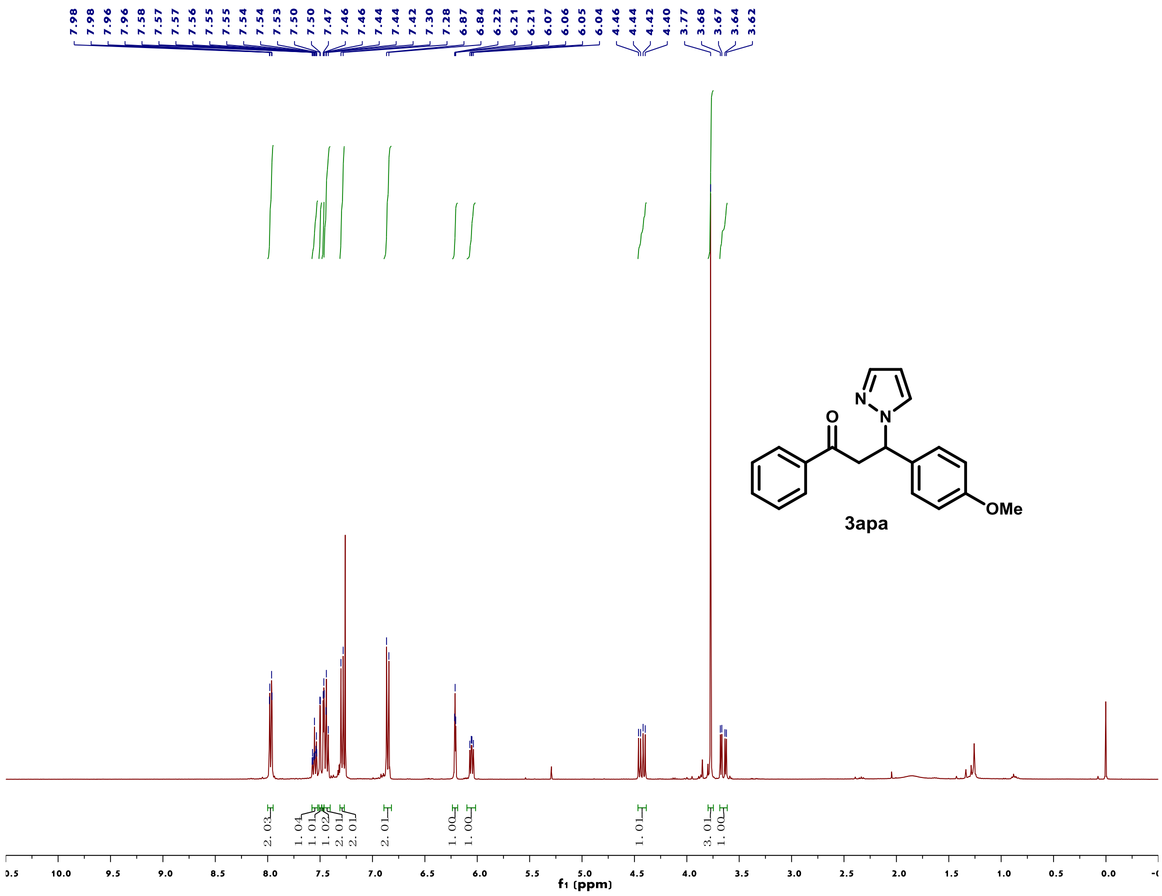

Supplementary Figure 186. **^{1}H NMR (400 MHz, $CDCl_3$) spectrum for 3apa**

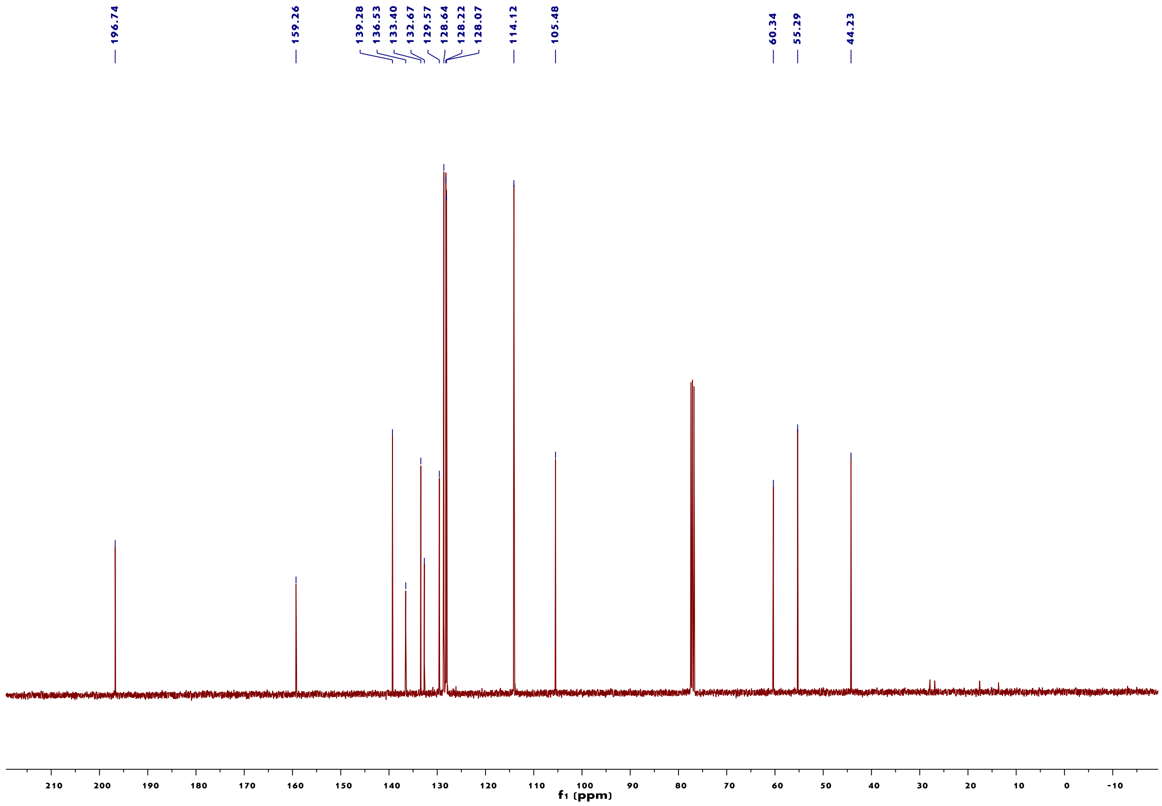

Supplementary Figure 187. **^{13}C NMR (100 MHz, $CDCl_3$) spectrum for 3apa**

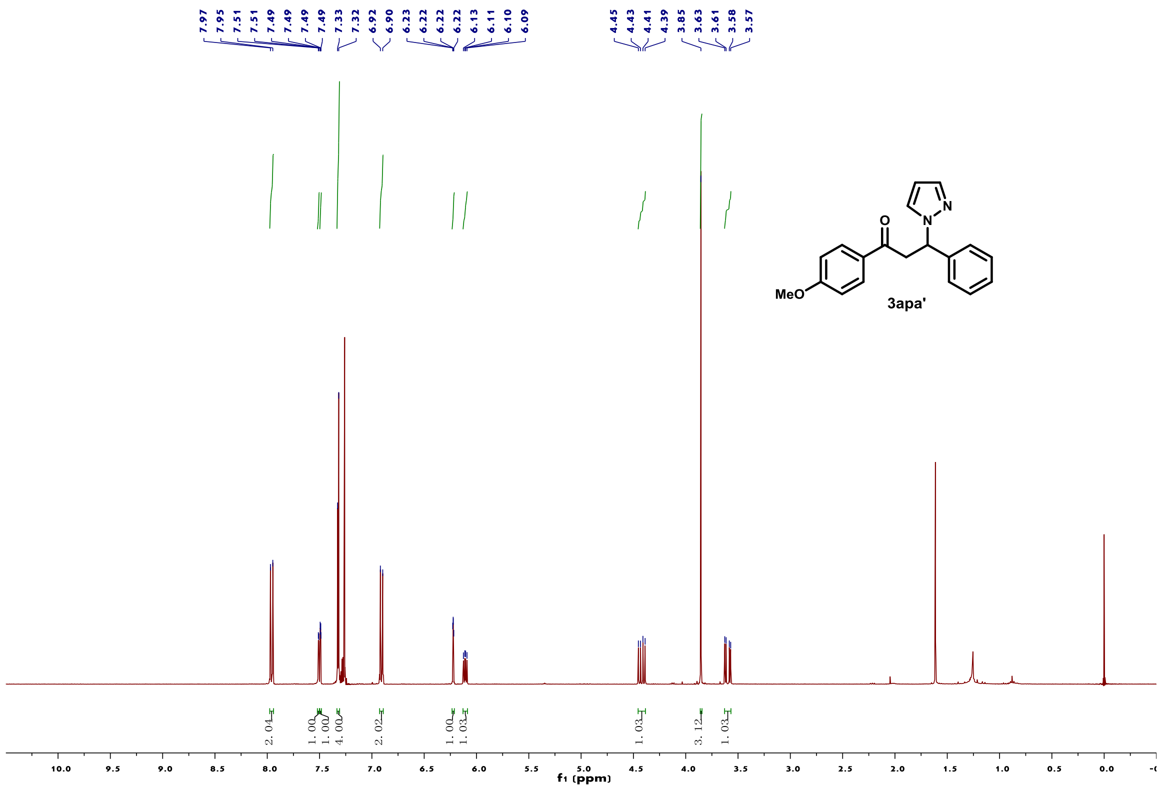

Supplementary Figure 188. ^{1}H NMR (400 MHz, $CDCl_3$) spectrum for 3apa’

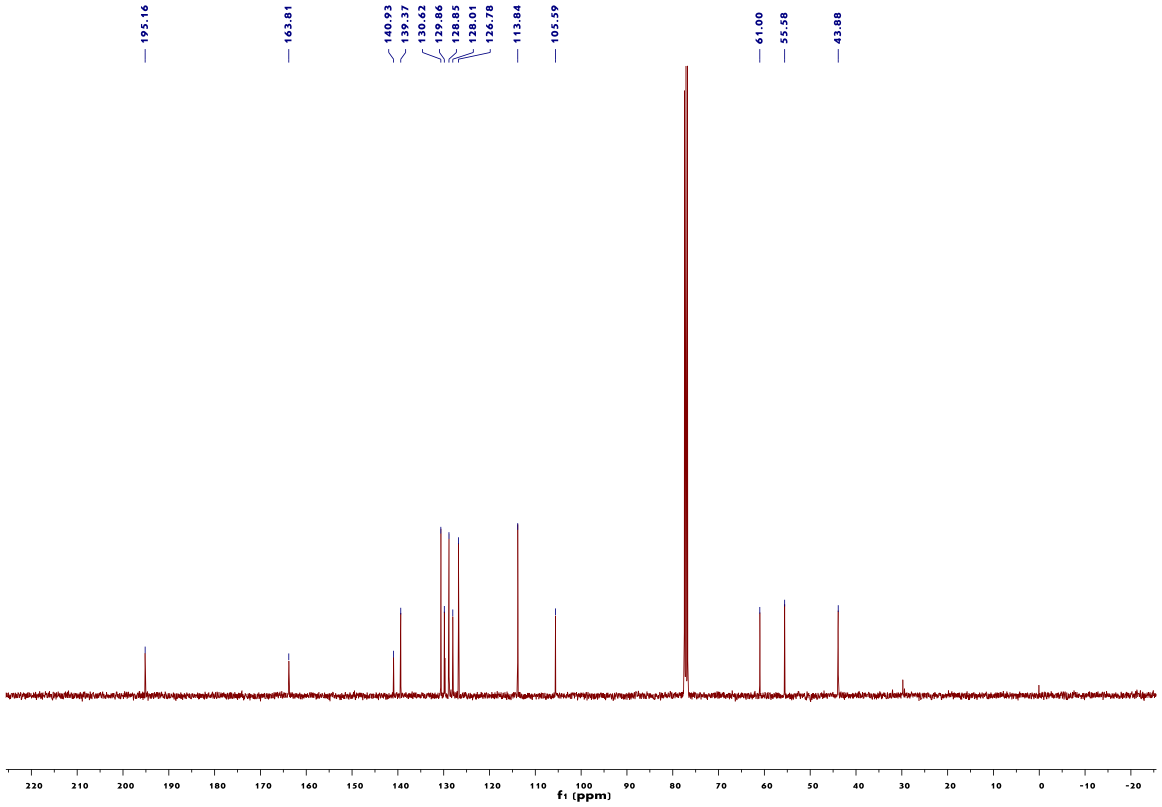

Supplementary Figure 189. ^{13}C NMR (100 MHz, $CDCl_3$) spectrum for 3apa’

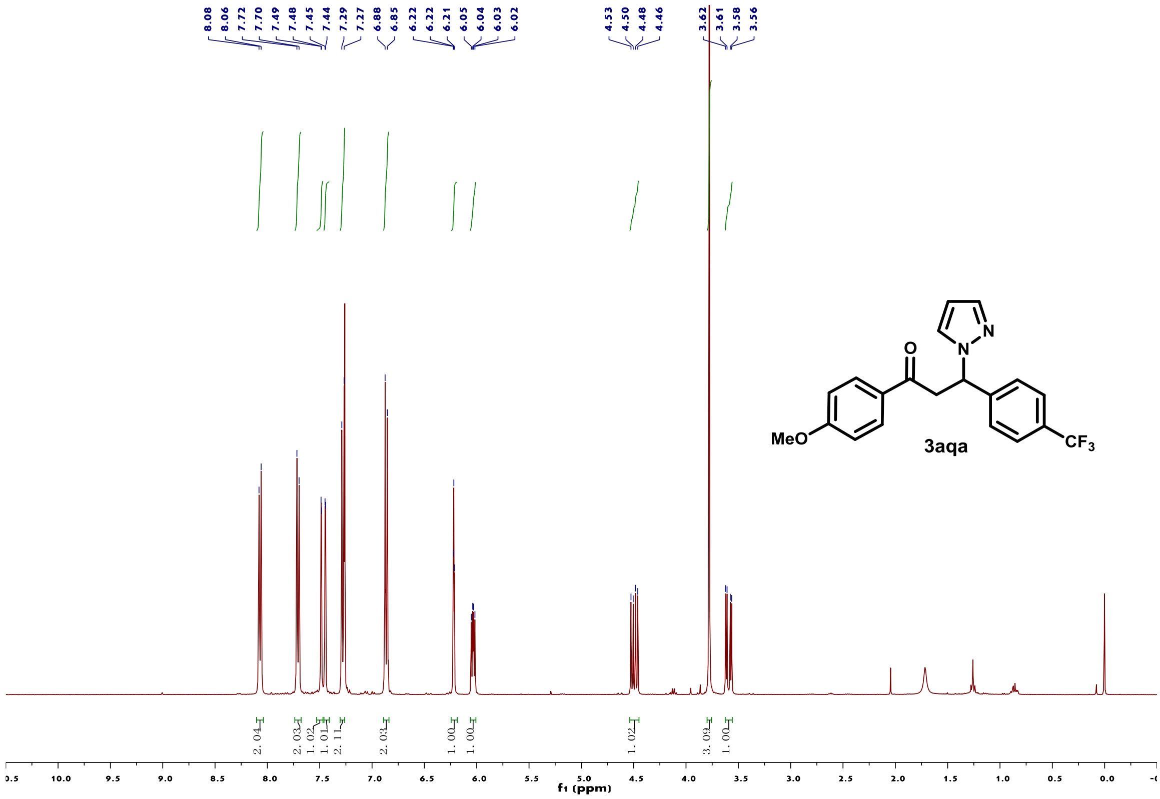

Supplementary Figure 190. ^{1}**H NMR (400 MHz,** $CDCl_3$**) spectrum for 3aqa**

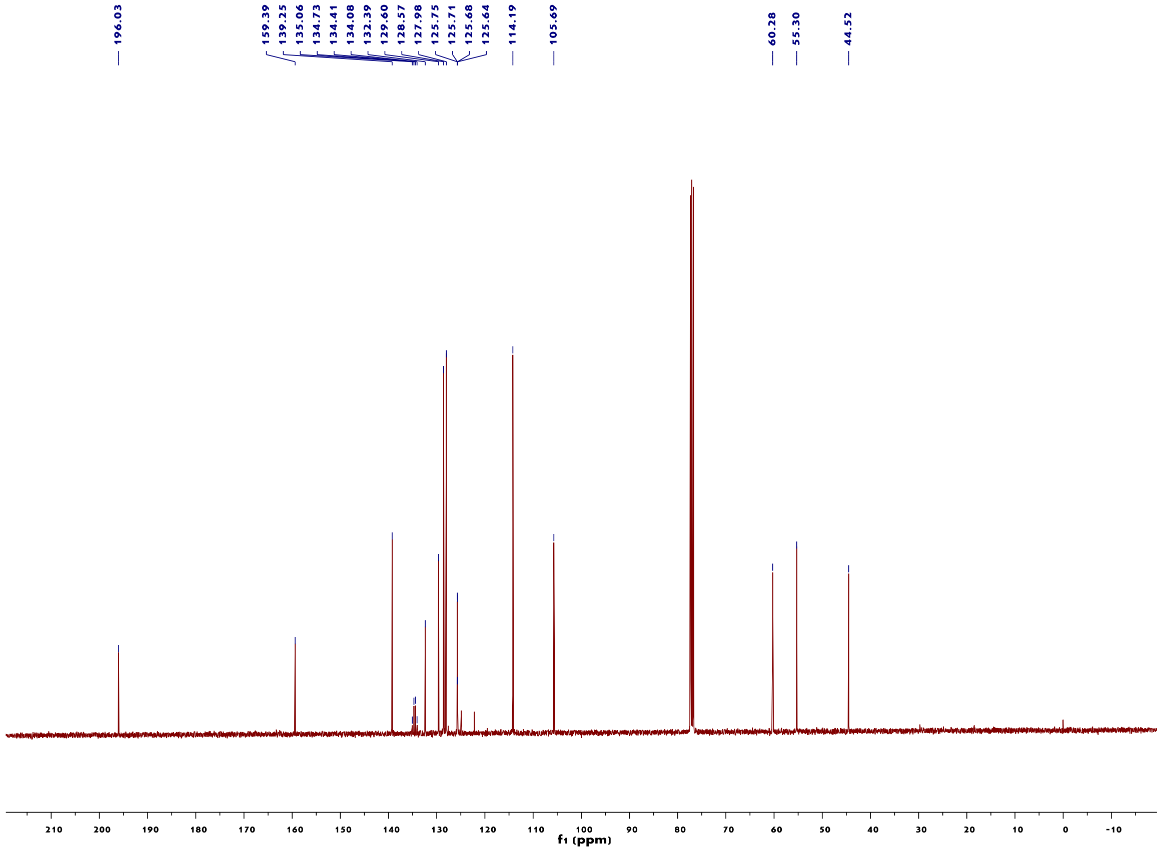

Supplementary Figure 191. ^{13}**C NMR (100 MHz,** $CDCl_3$**) spectrum for 3aqa**

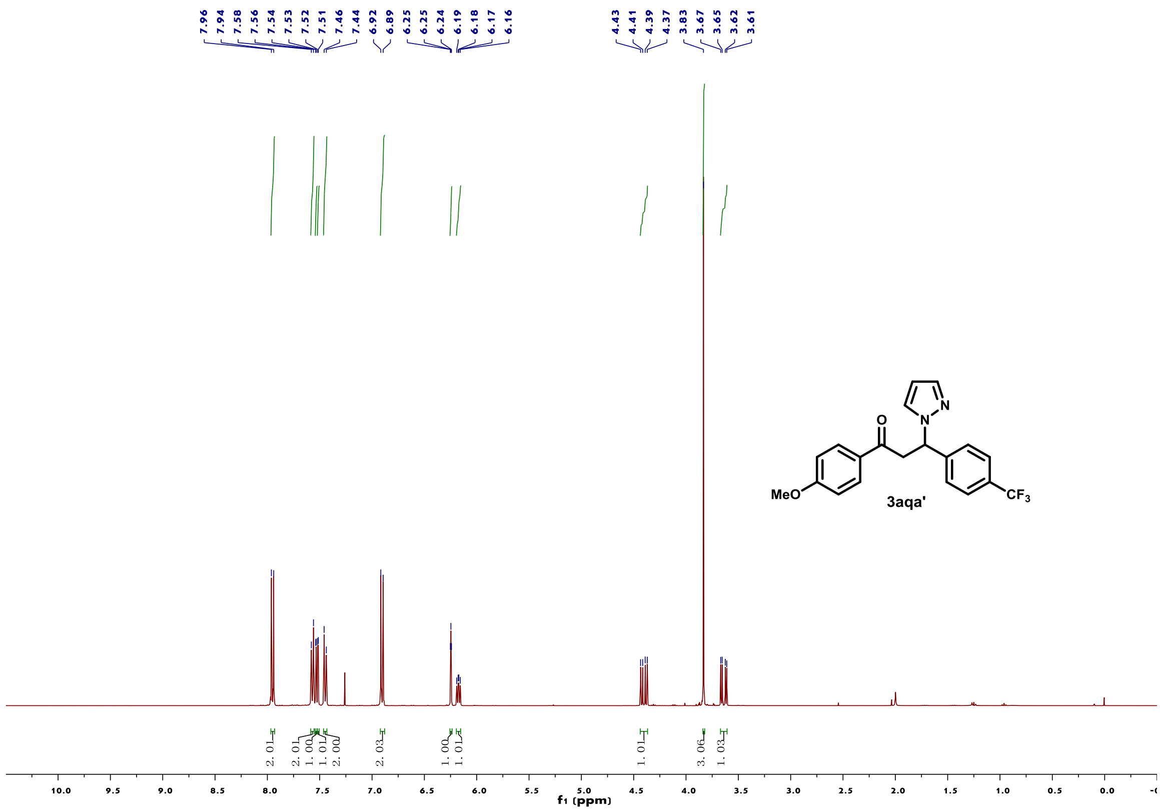

Supplementary Figure 192. ^{1}H NMR (400 MHz, $CDCl_3$) spectrum for 3aqa’

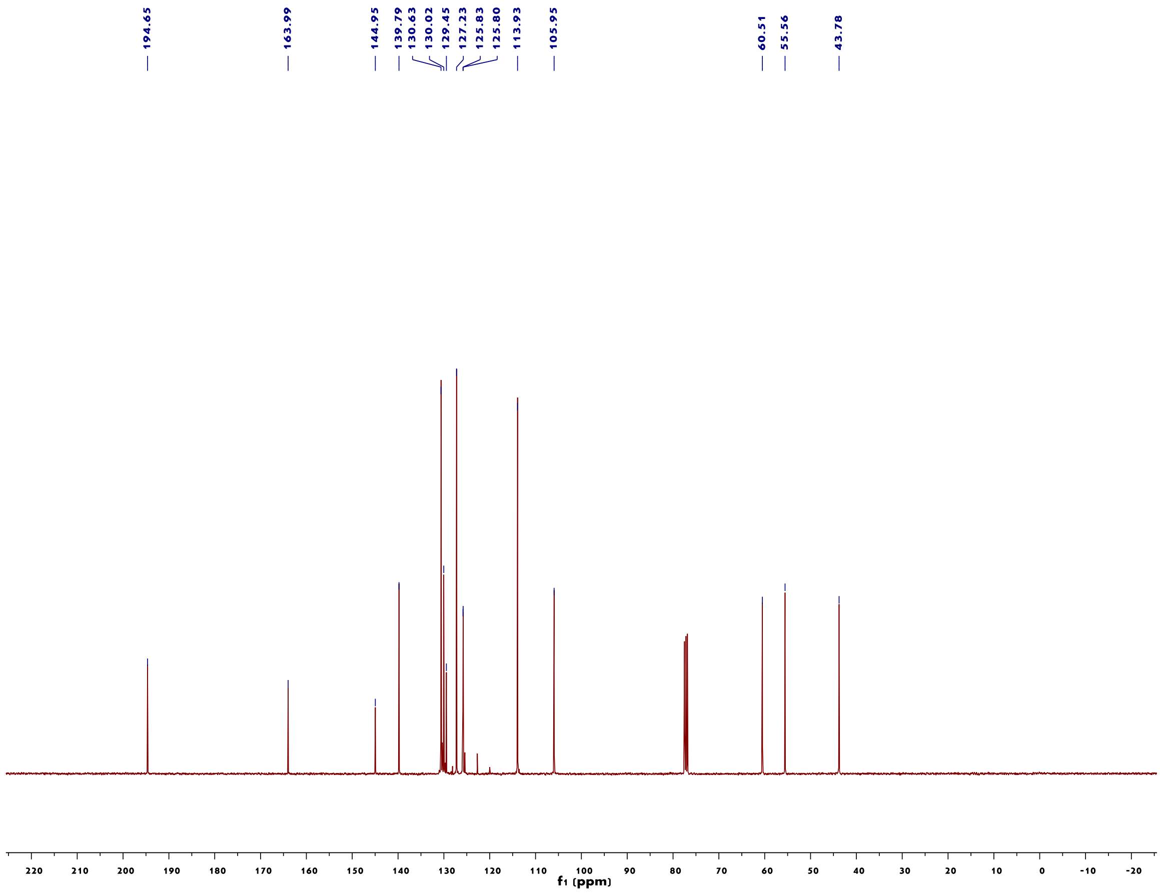

Supplementary Figure 193. ^{13}C NMR (100 MHz, $CDCl_3$) spectrum for 3aqa’

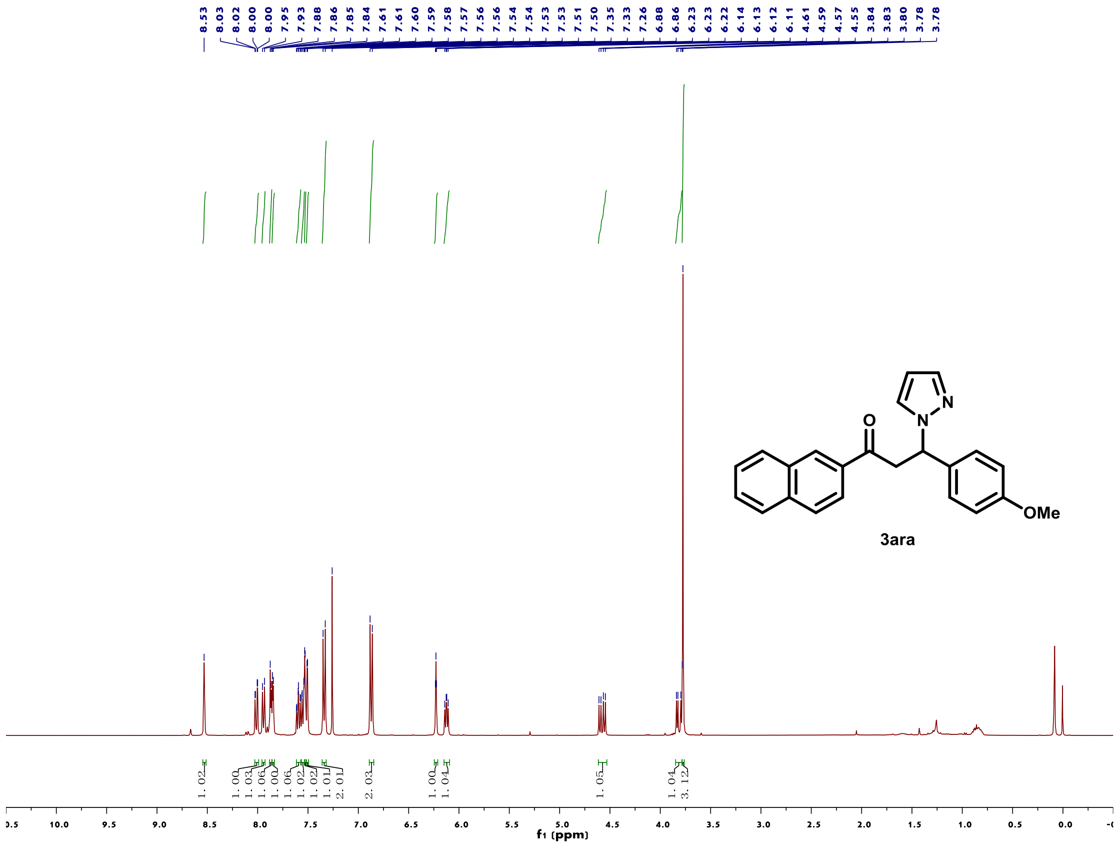

Supplementary Figure 194. ^{1}H NMR (400 MHz, $CDCl_3$) spectrum for 3ara

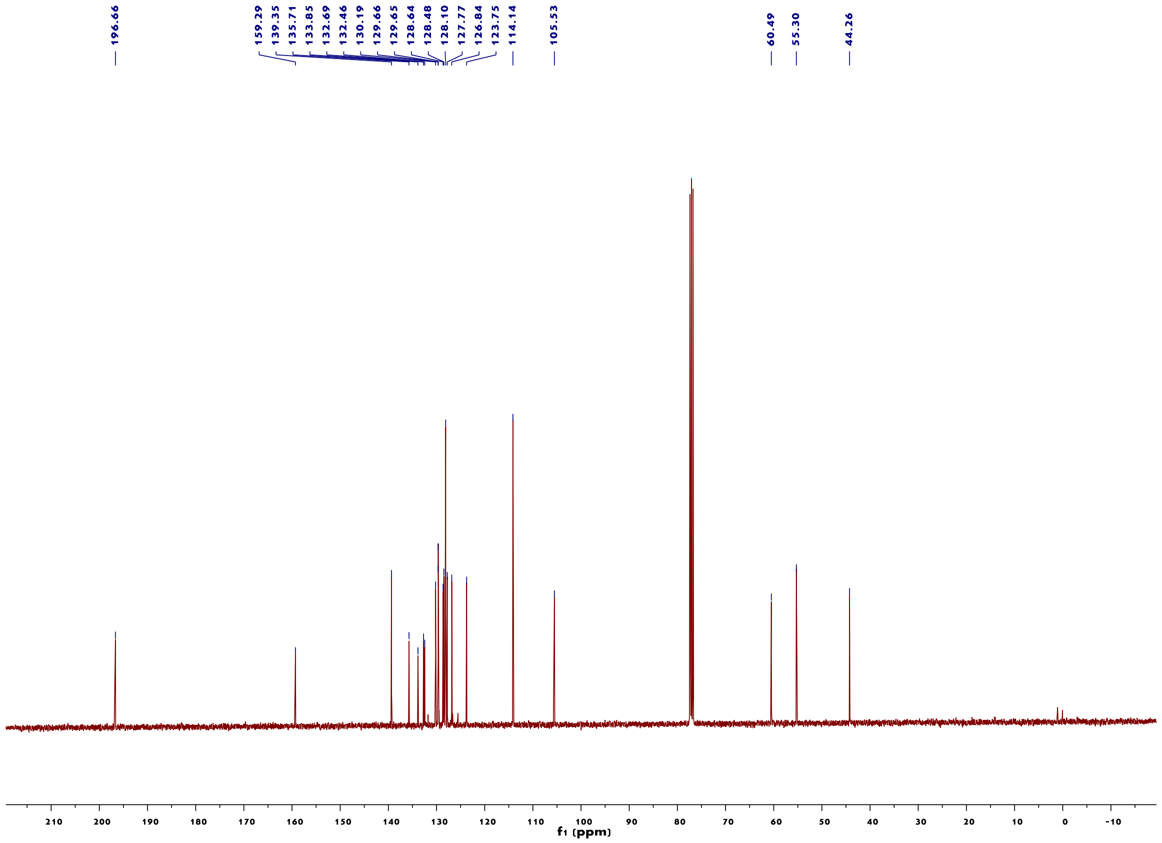

Supplementary Figure 195. ^{13}C NMR (100 MHz, $CDCl_3$) spectrum for 3ara

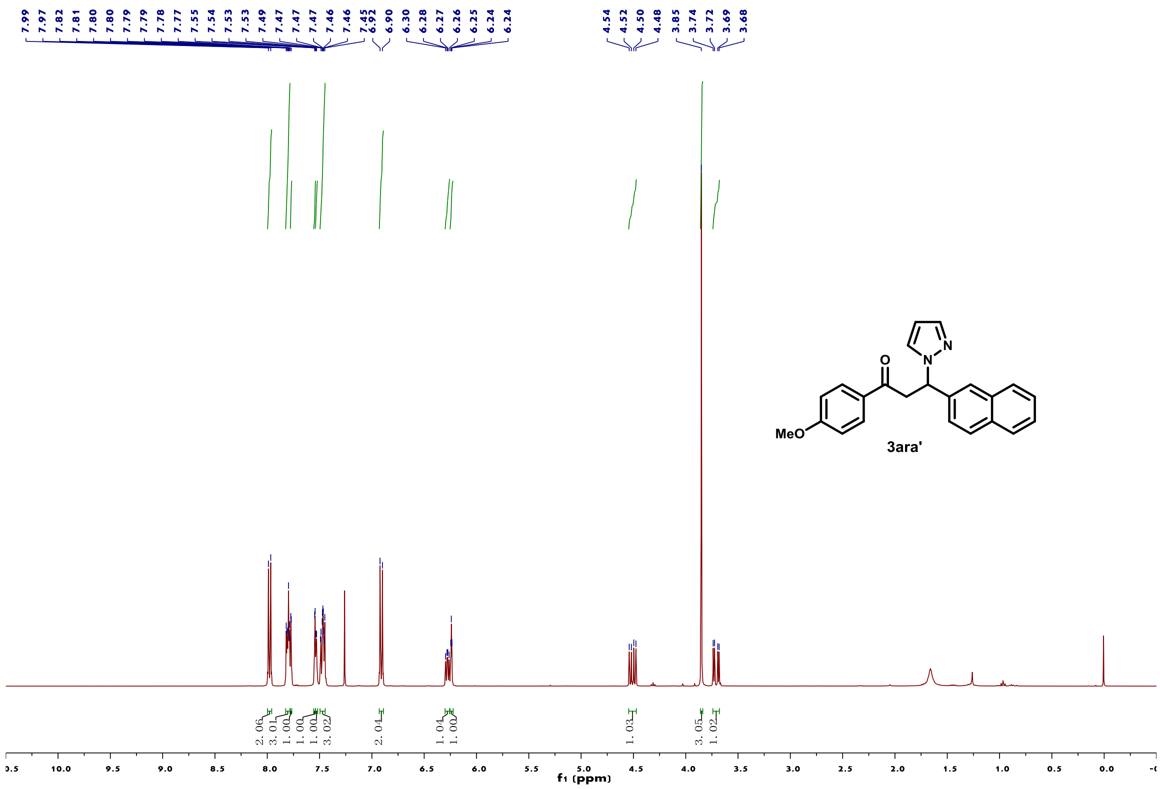

Supplementary Figure 196. ^{1}H **NMR (400 MHz,** $CDCl_3$**) spectrum for 3ara’**

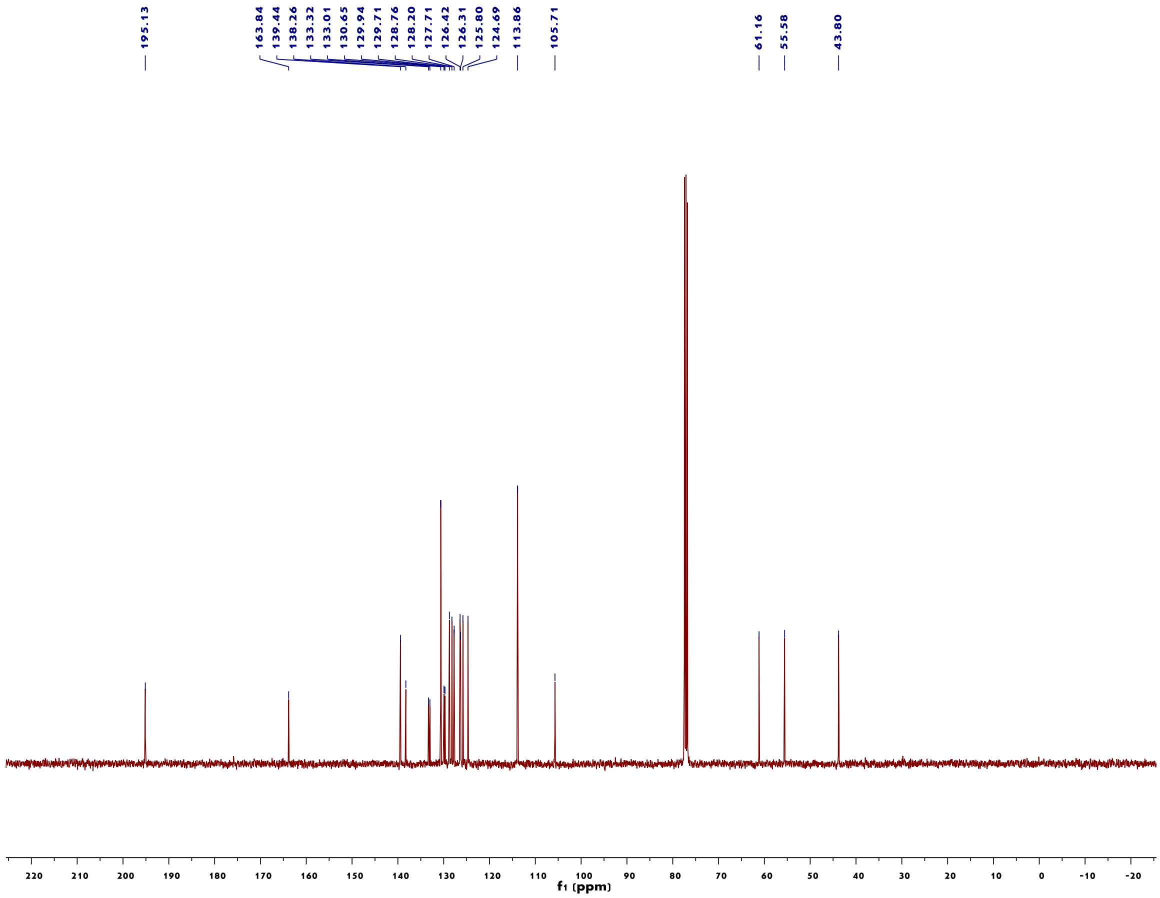

Supplementary Figure 197. ^{13}C **NMR (100 MHz,** $CDCl_3$**) spectrum for 3ara’**

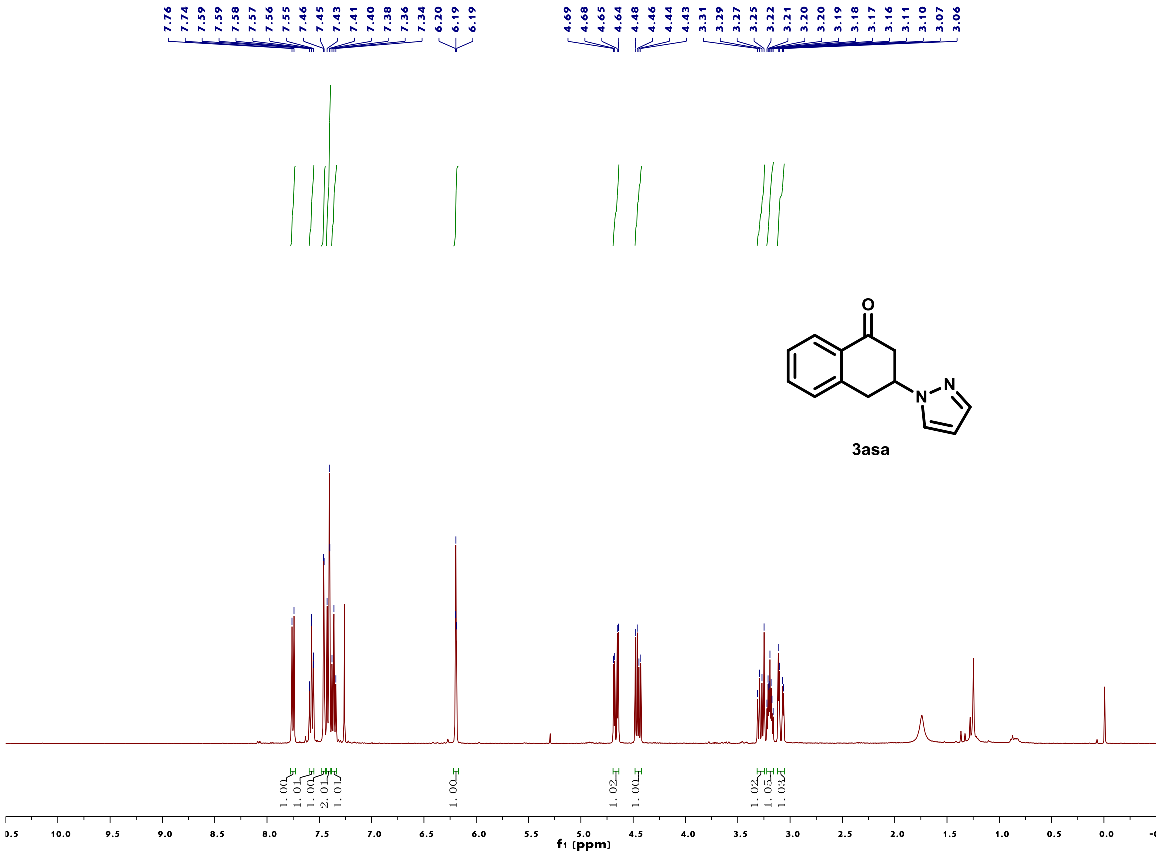

Supplementary Figure 198. ^{1}H **NMR (400 MHz,** $CDCl_3$**) spectrum for 3asa**

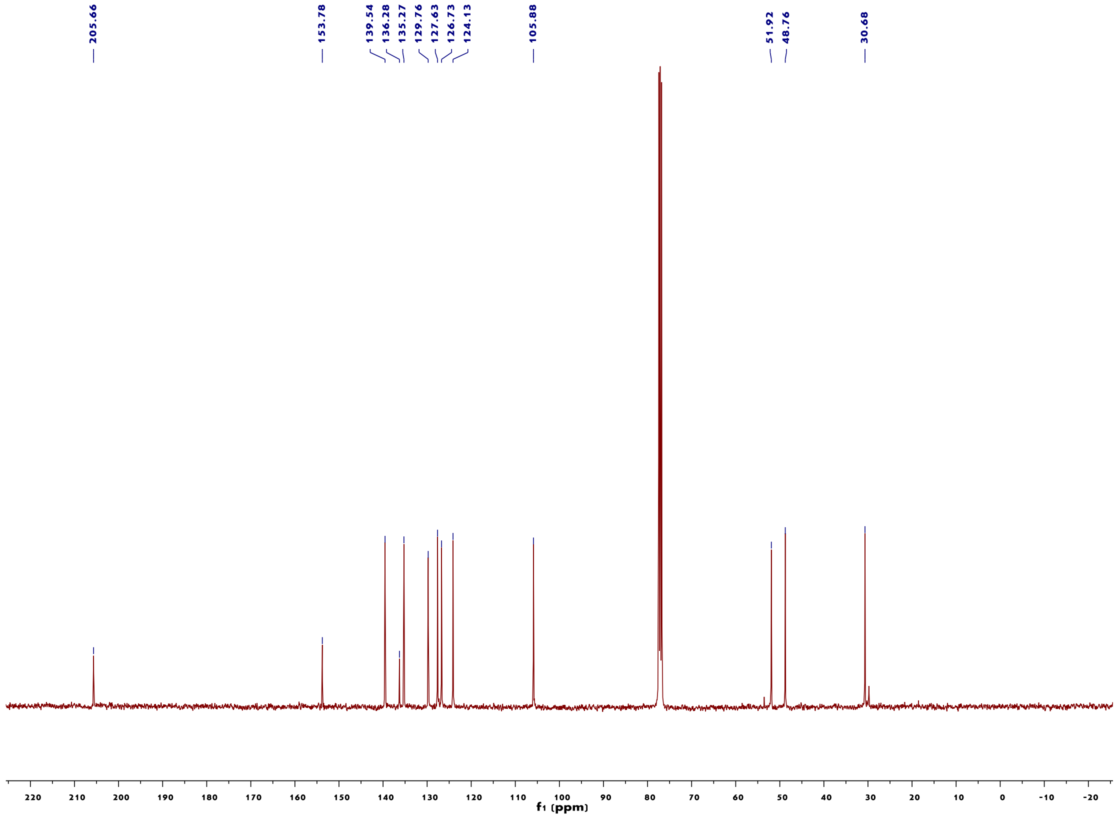

Supplementary Figure 199. ^{13}C **NMR (100 MHz,** $CDCl_3$**) spectrum for 3asa**

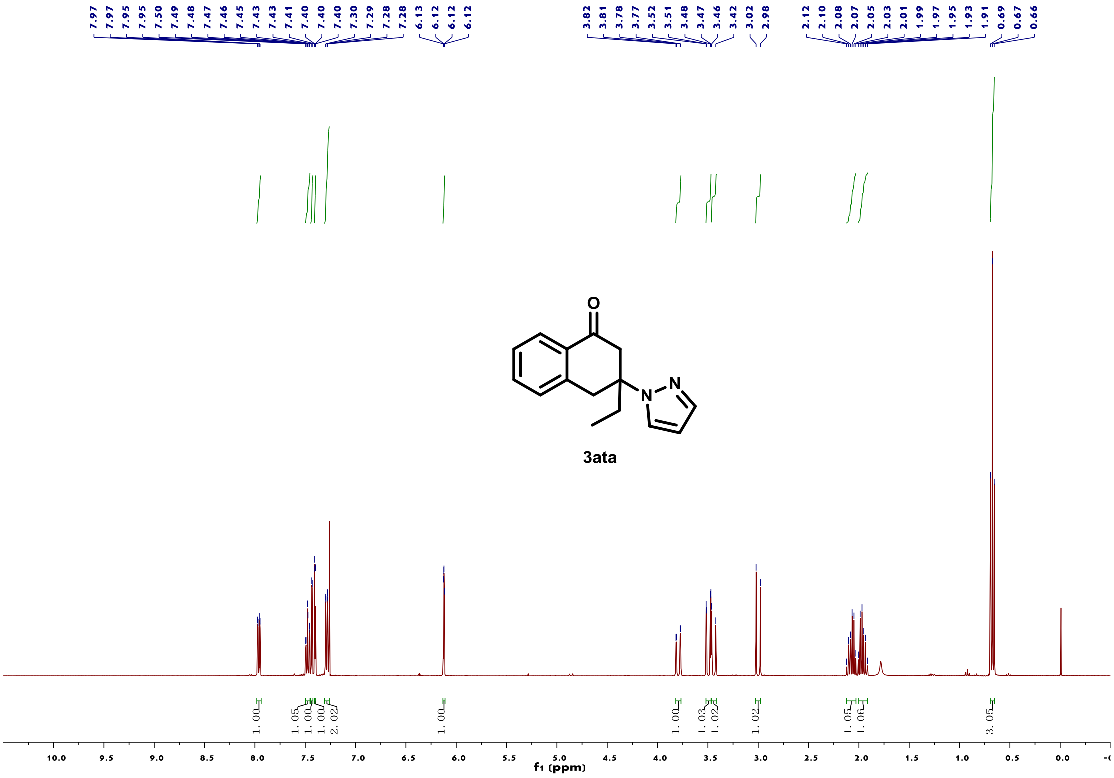

Supplementary Figure 200. ^{1}H NMR (400 MHz, $CDCl_3$) spectrum for 3ata

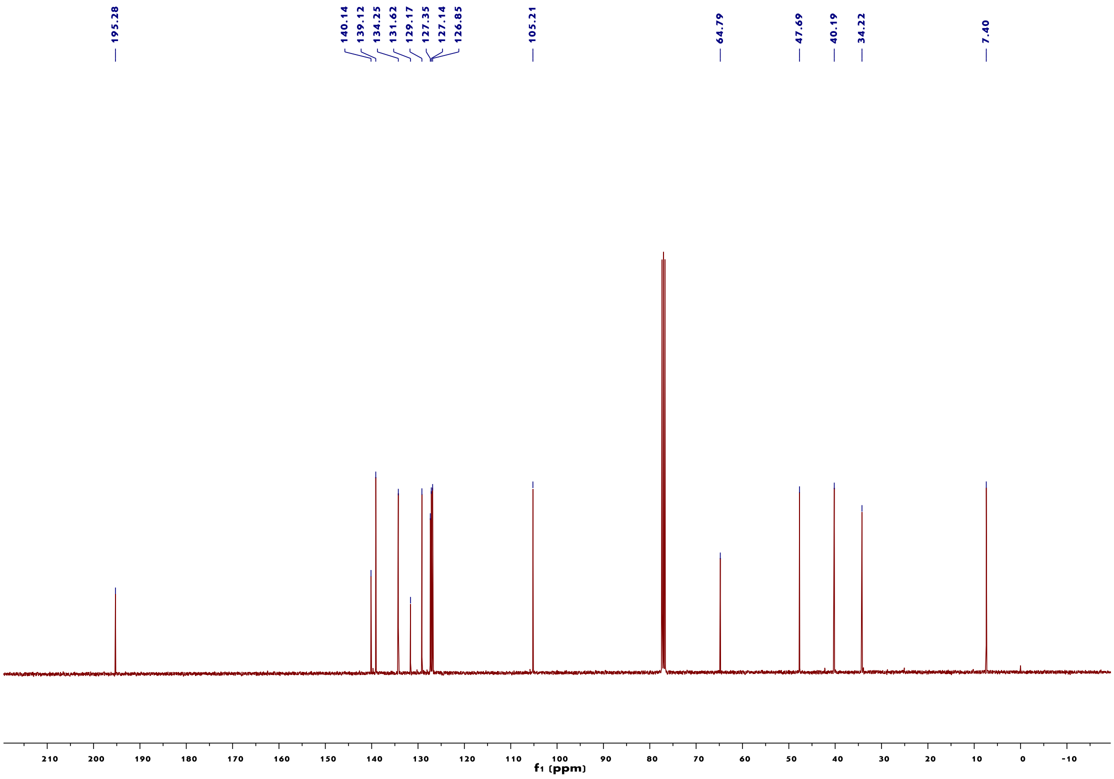

Supplementary Figure 201. ^{13}C NMR (100 MHz, $CDCl_3$) spectrum for 3ata

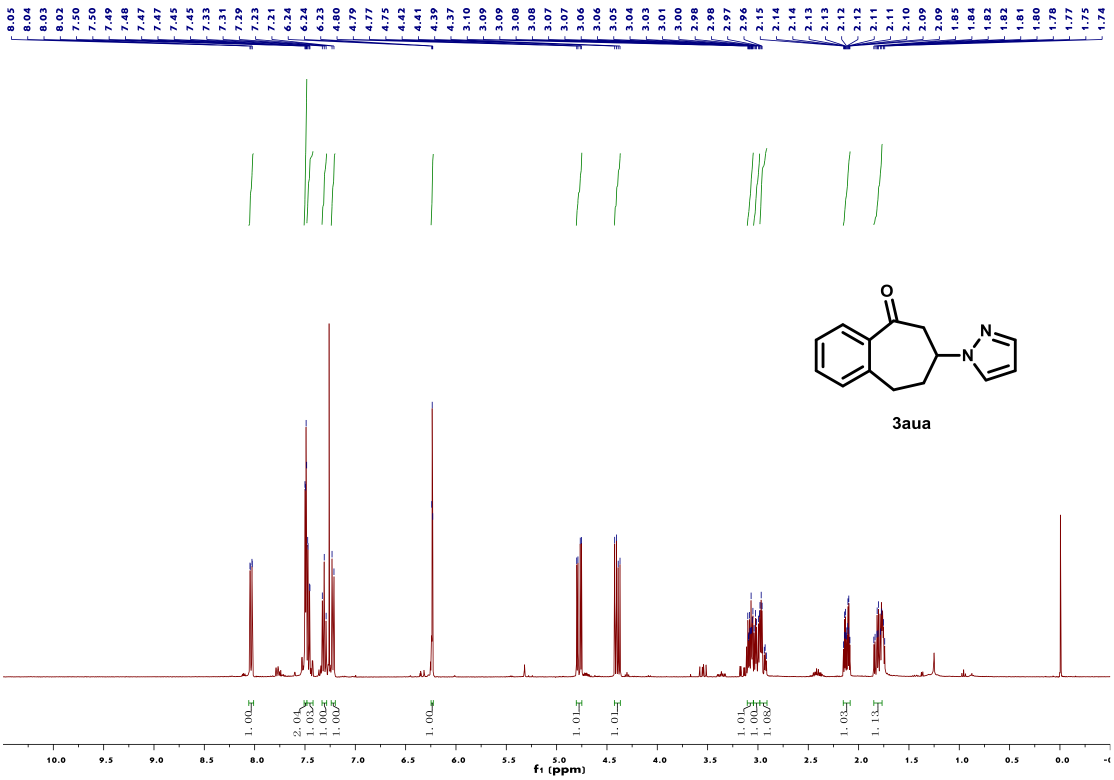

Supplementary Figure 202. ^{1}H NMR (400 MHz, $CDCl_3$) spectrum for 3aua

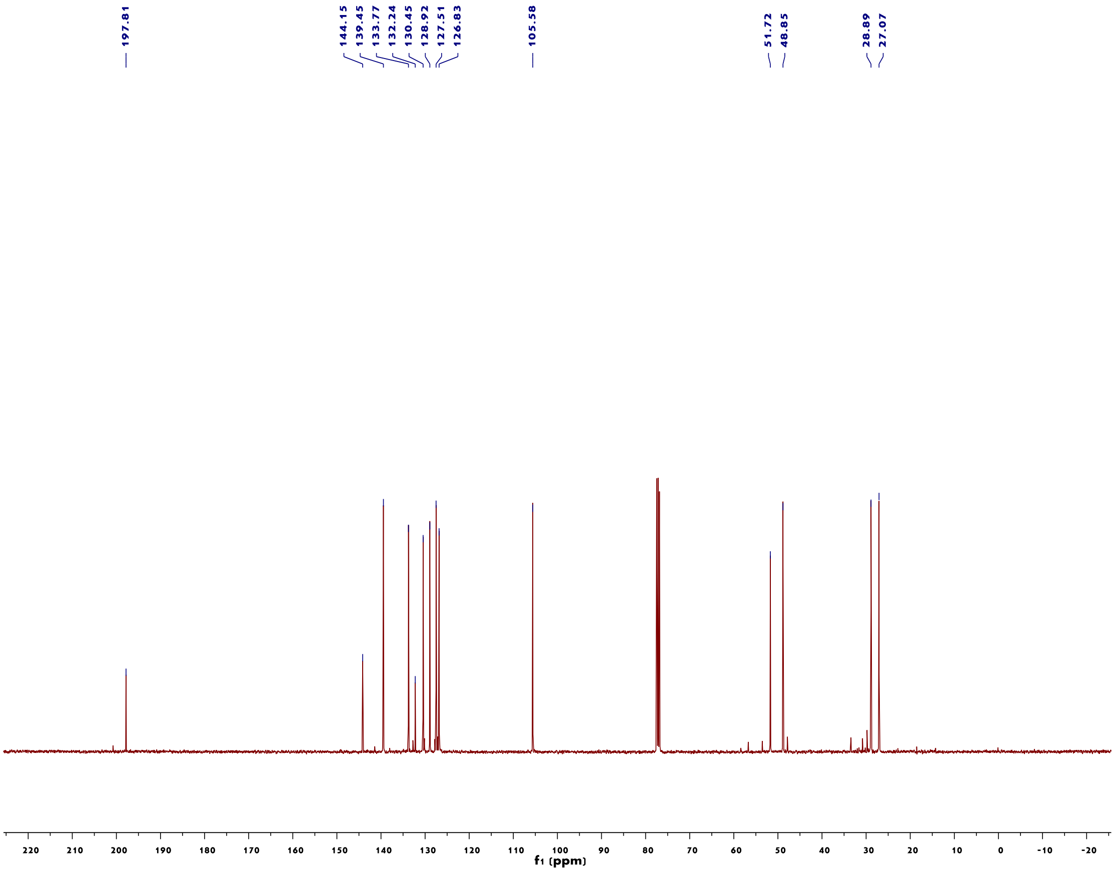

Supplementary Figure 203. ^{13}C NMR (100 MHz, $CDCl_3$) spectrum for 3aua

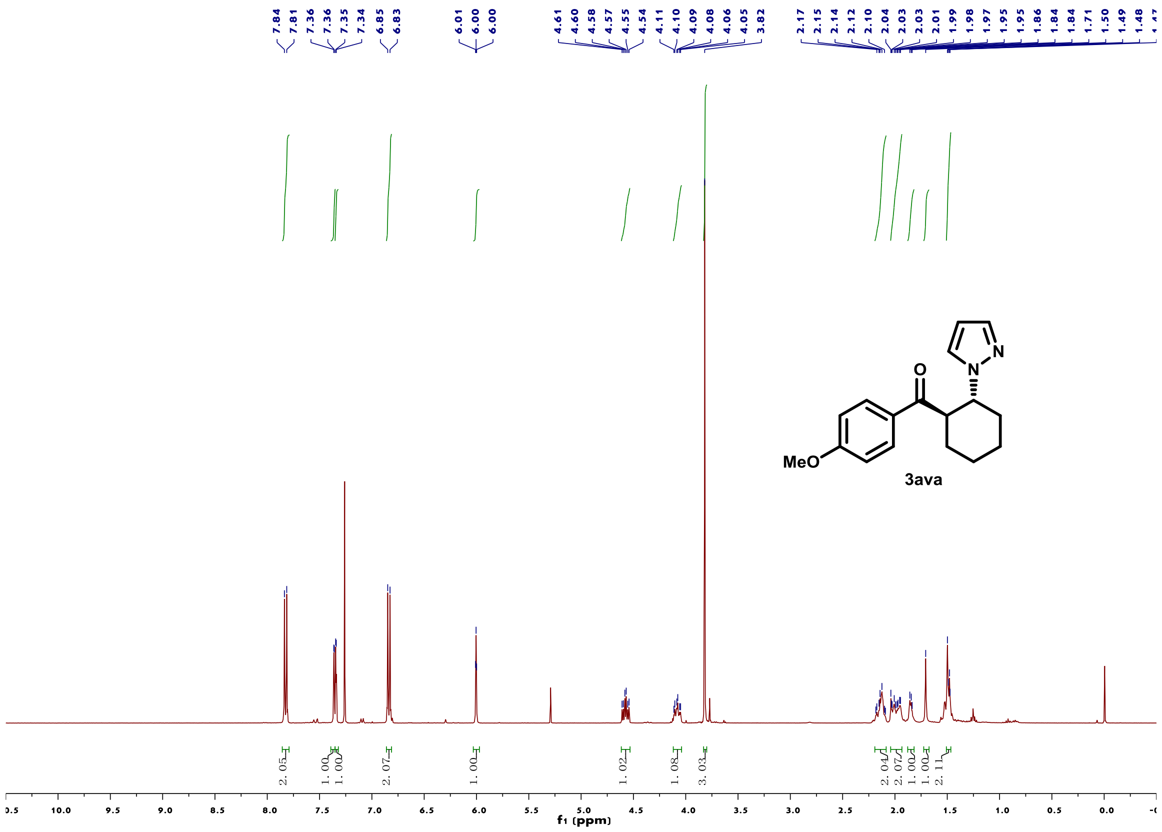

Supplementary Figure 204. ^{1}H NMR (400 MHz, $CDCl_3$) spectrum for 3ava

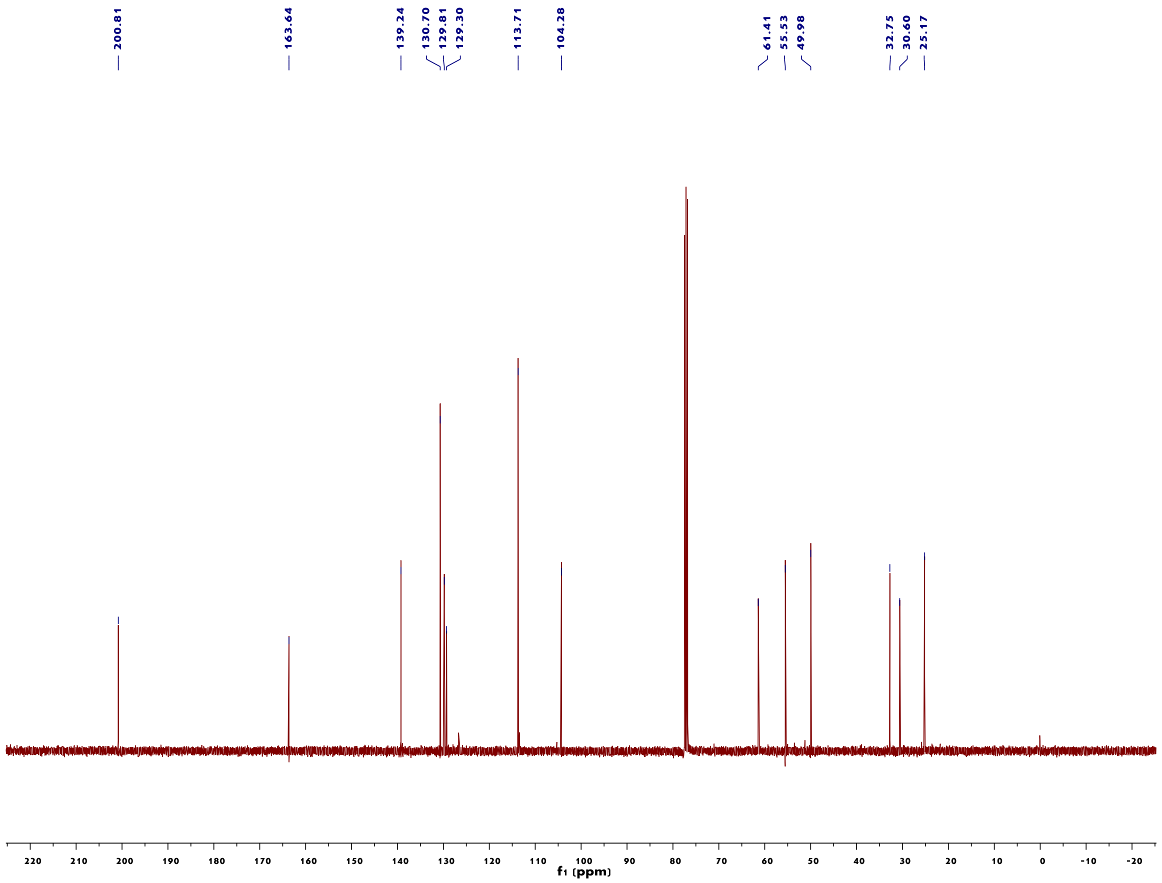

Supplementary Figure 205. ^{13}C NMR (100 MHz, $CDCl_3$) spectrum for 3ava

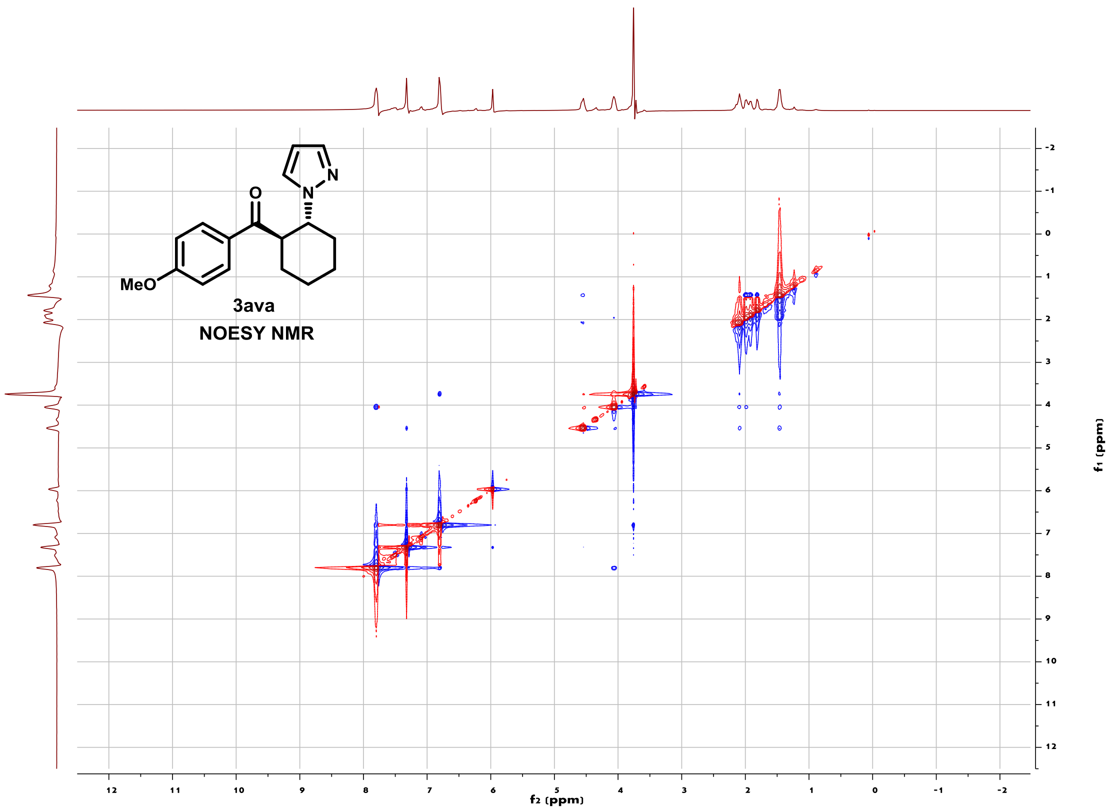

Supplementary Figure 206. NOESY NMR spectrum for 3ava

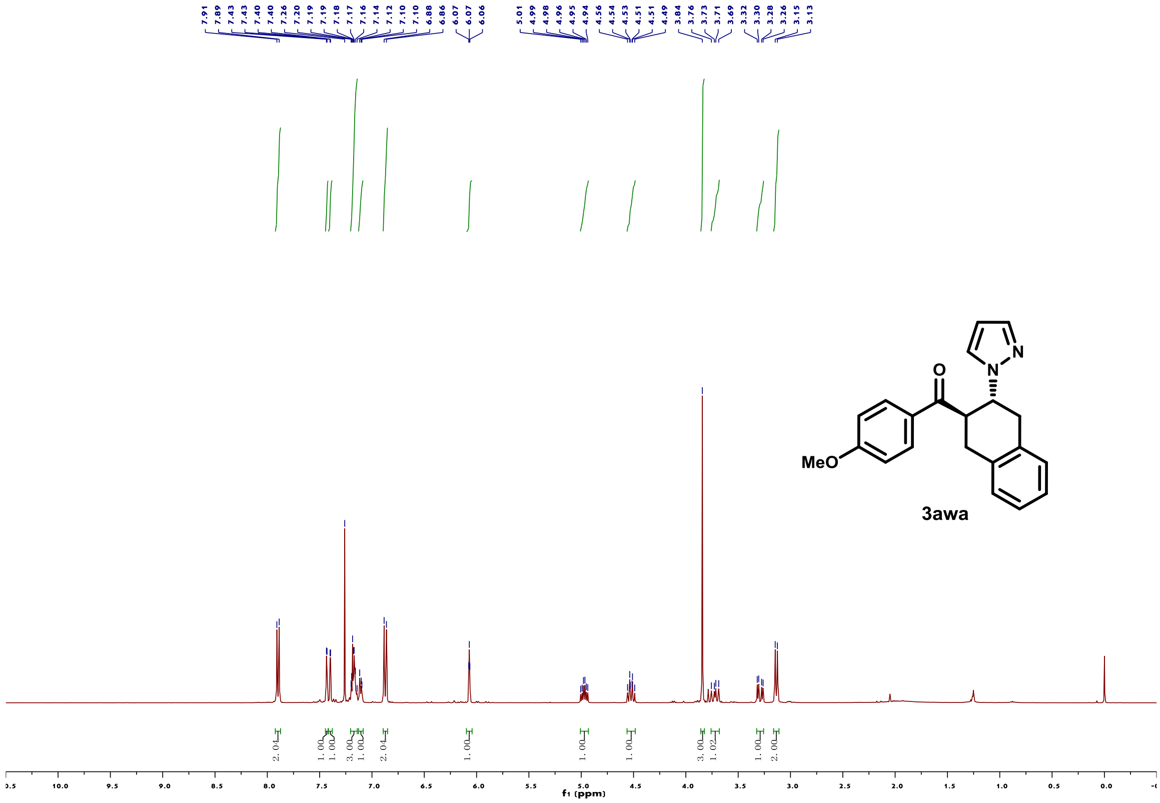

Supplementary Figure 207. ^{1}H NMR (400 MHz, $CDCl_3$) spectrum for 3awa

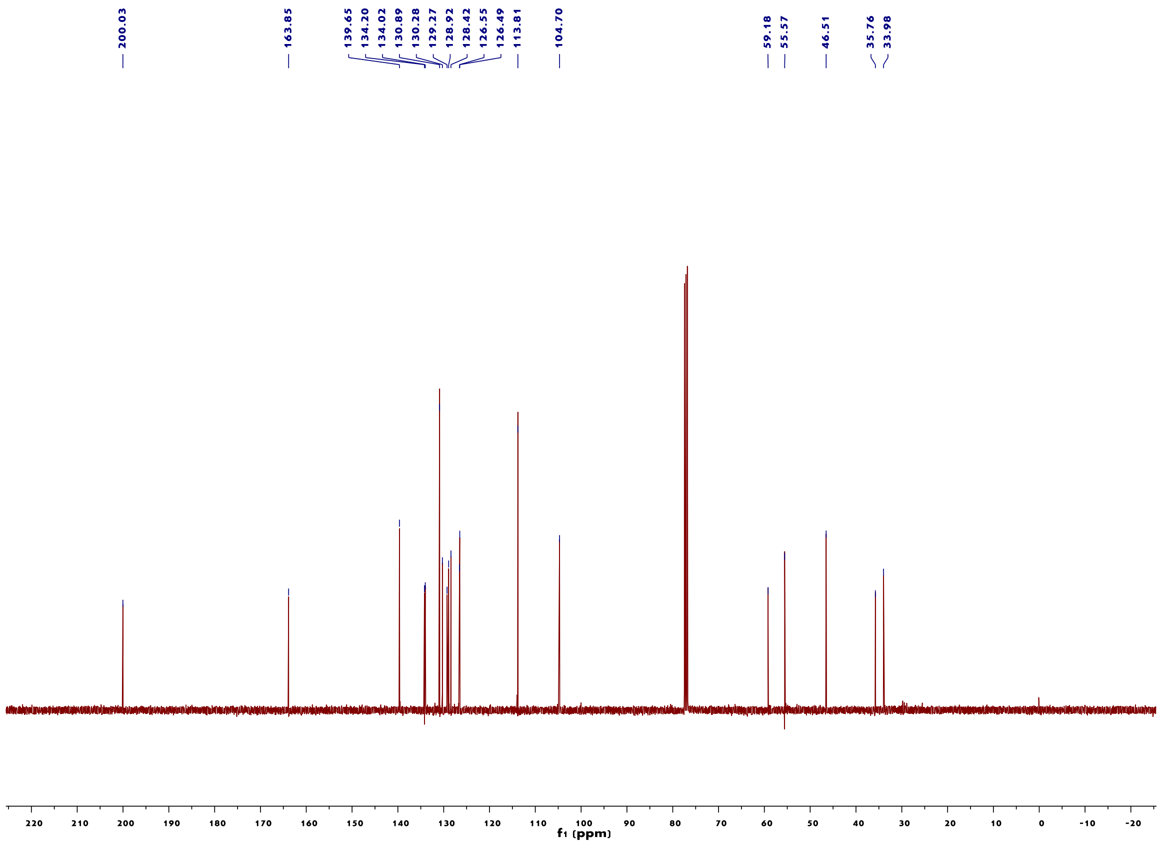

Supplementary Figure 208. ^{13}C NMR (100 MHz, $CDCl_3$) spectrum for 3awa

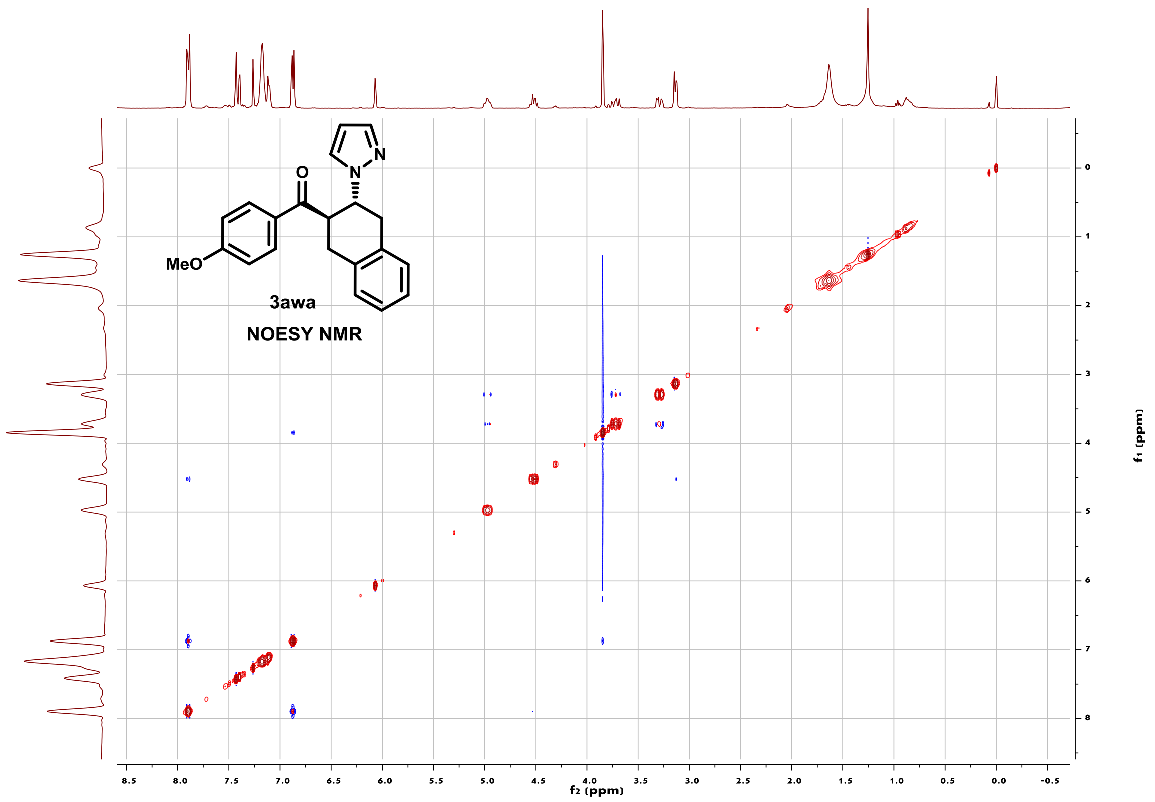

Supplementary Figure 209. NOESY NMR spectrum for 3awa

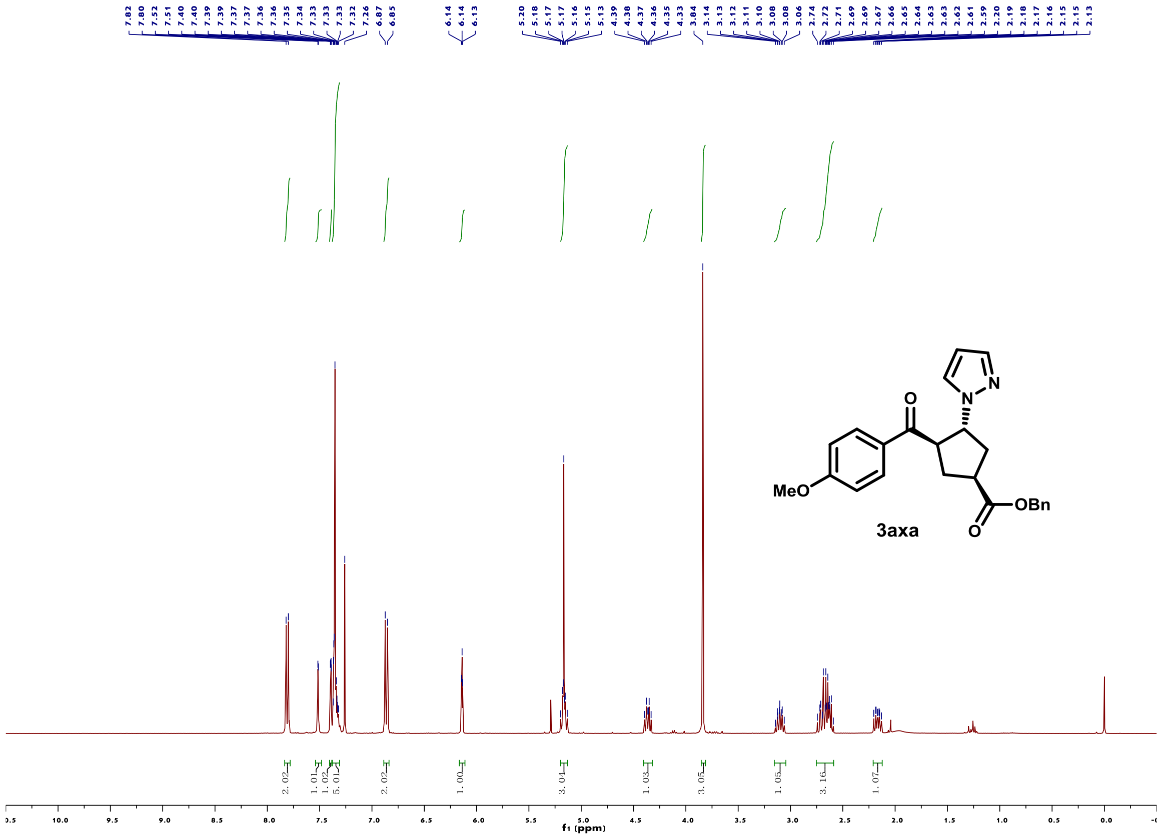

Supplementary Figure 210. ^{1}H NMR (400 MHz, $CDCl_3$) spectrum for 3axa

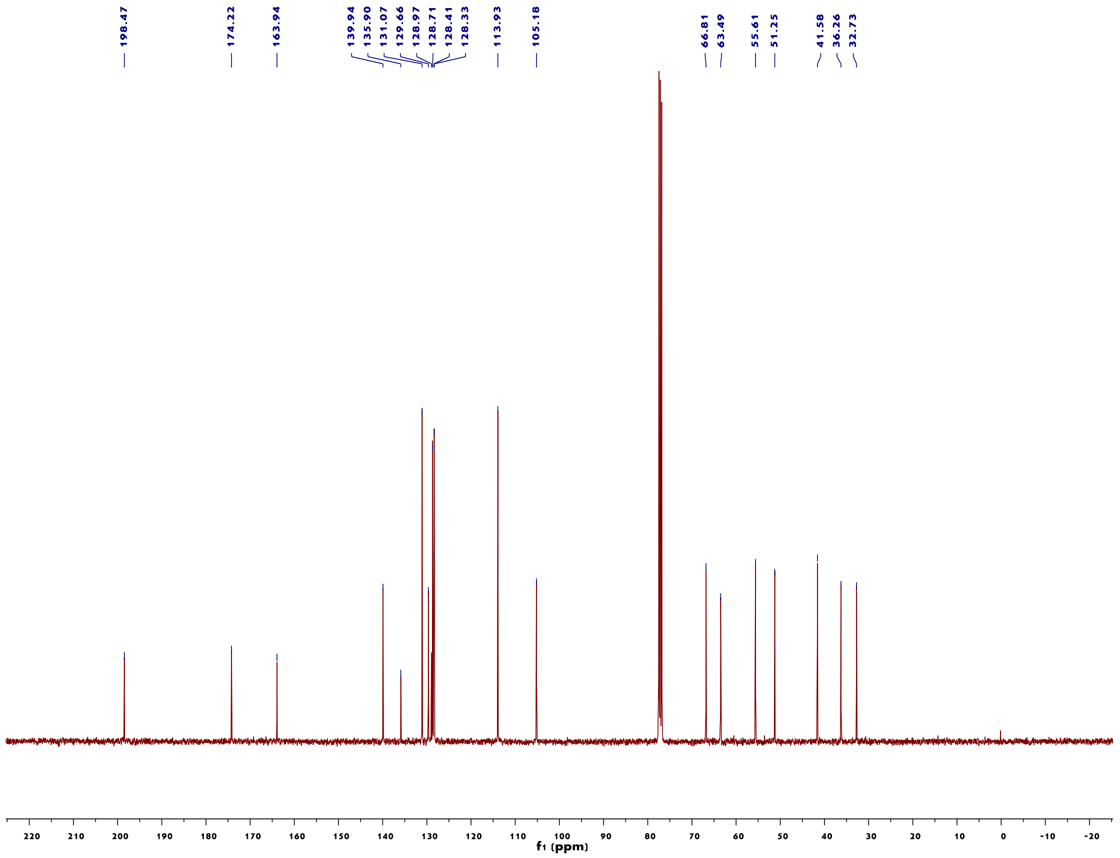

Supplementary Figure 211. ^{13}C NMR (100 MHz, $CDCl_3$) spectrum for 3axa

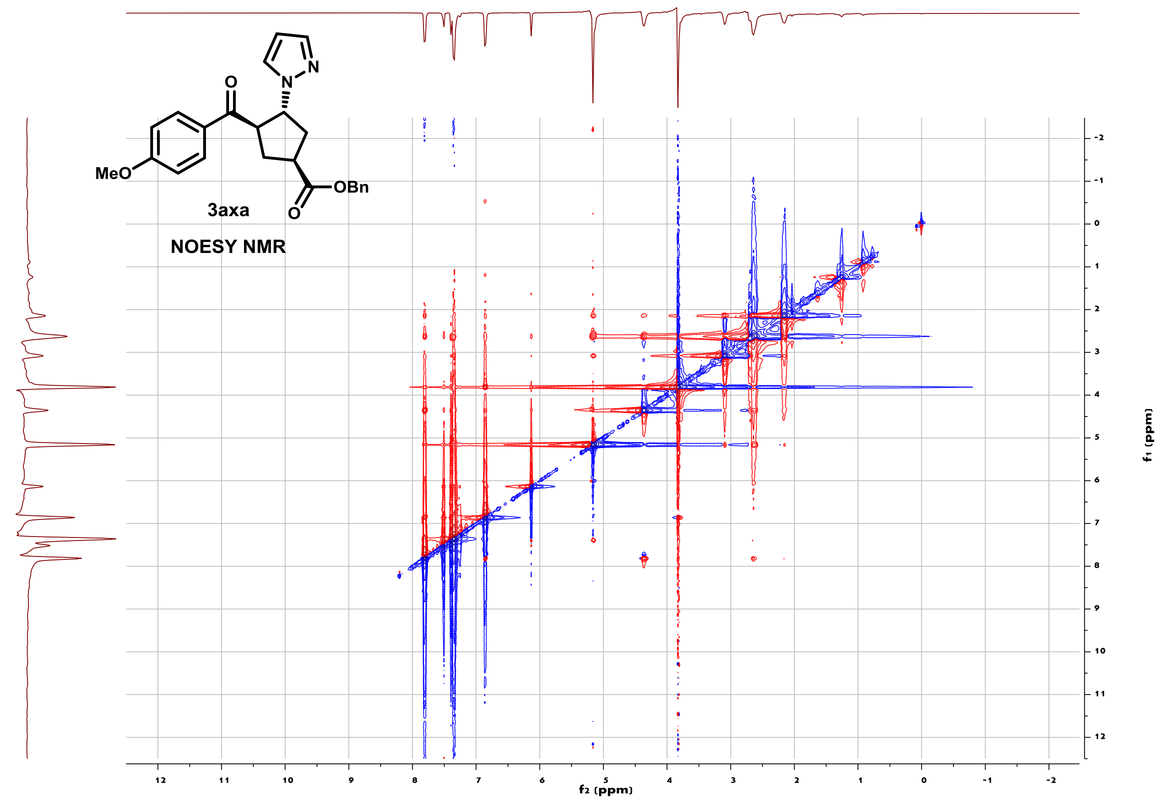

Supplementary Figure 212. NOESY NMR spectrum for 3axa

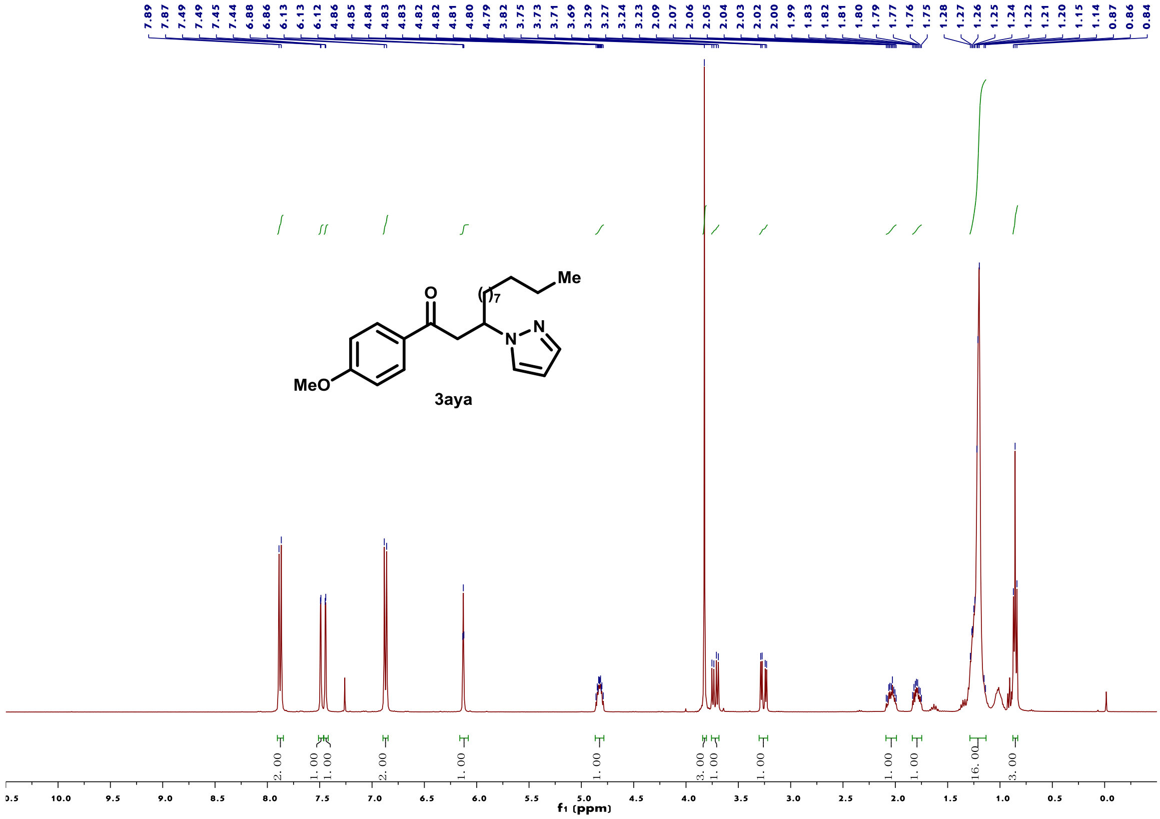

Supplementary Figure 213. ^{1}H NMR (400 MHz, $CDCl_3$) spectrum for 3aya

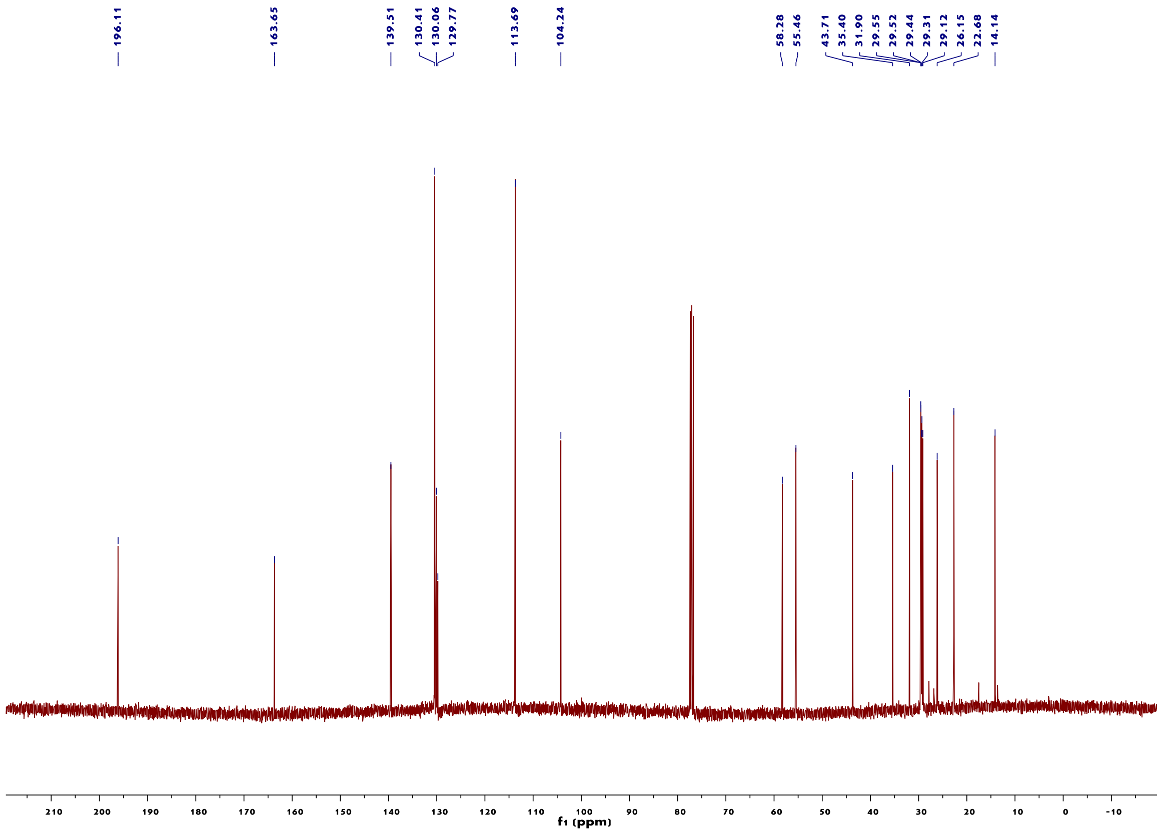

Supplementary Figure 214. ^{13}C NMR (100 MHz, $CDCl_3$) spectrum for 3aya

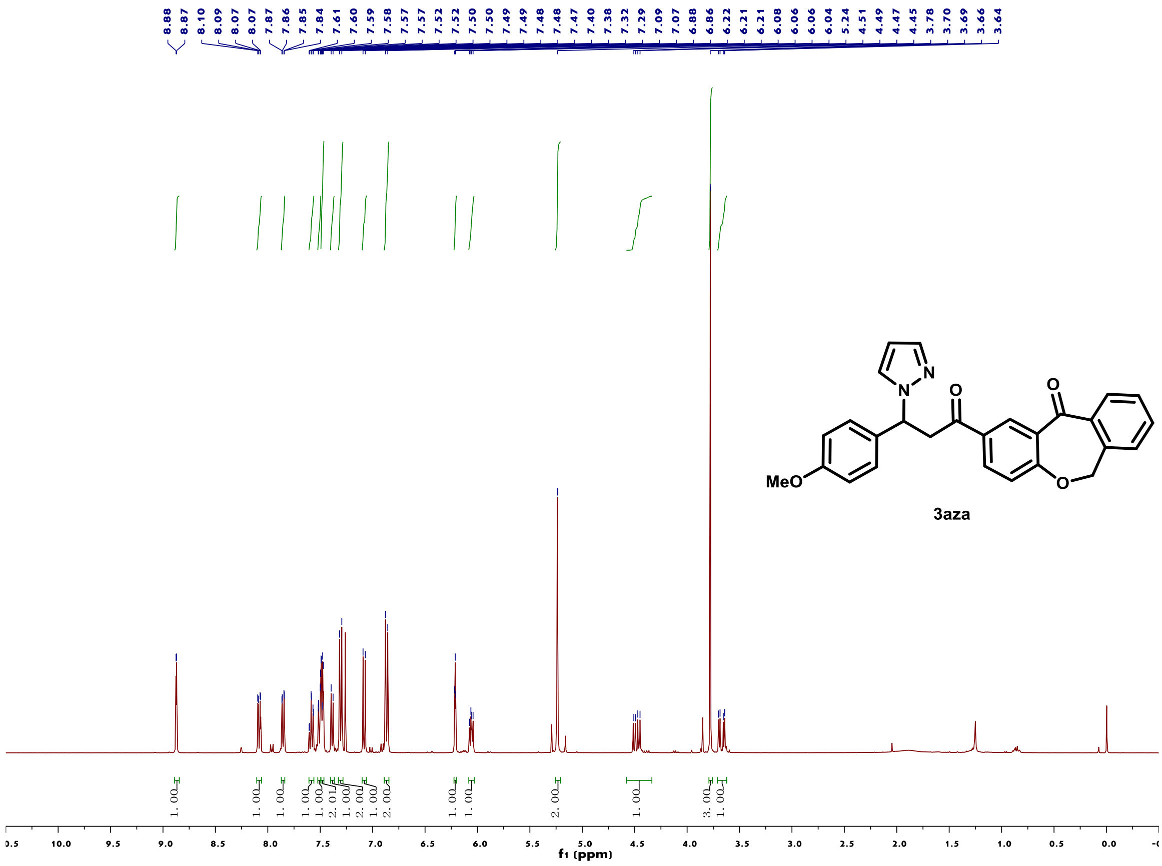

Supplementary Figure 215. ^{1}H NMR (400 MHz, $CDCl_3$) spectrum for 3aza

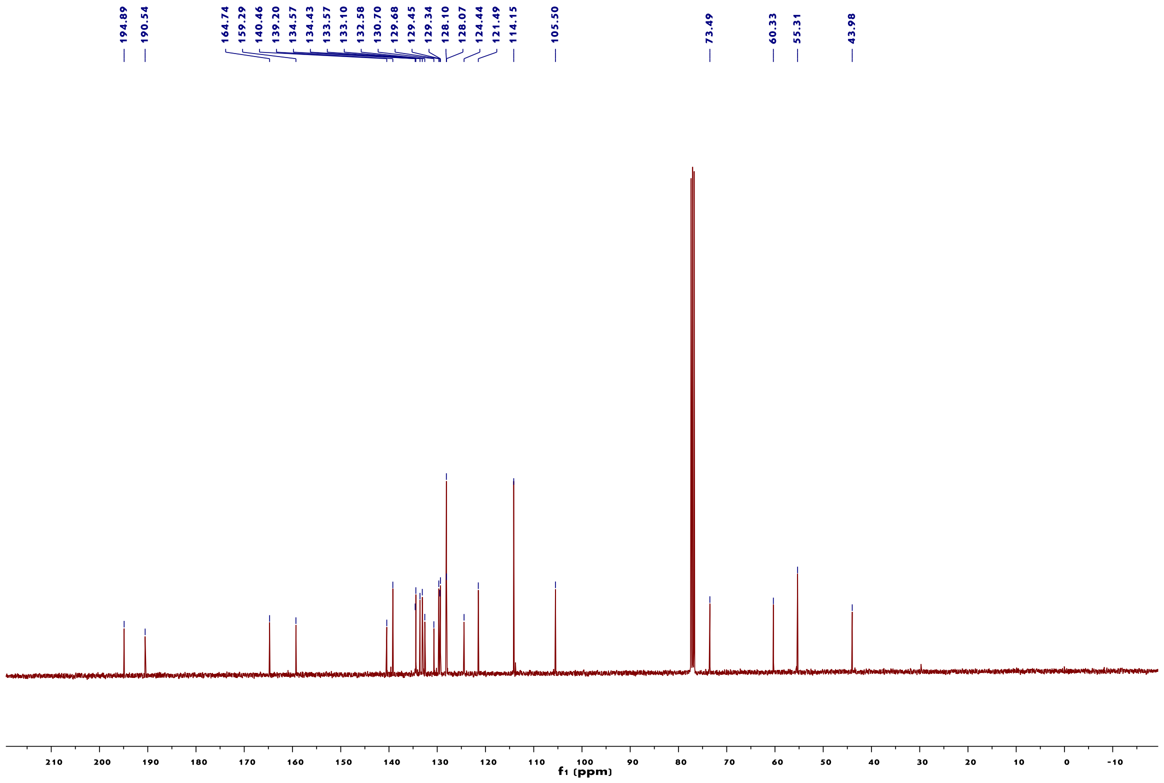

Supplementary Figure 216. ^{13}C NMR (100 MHz, $CDCl_3$) spectrum for 3aza

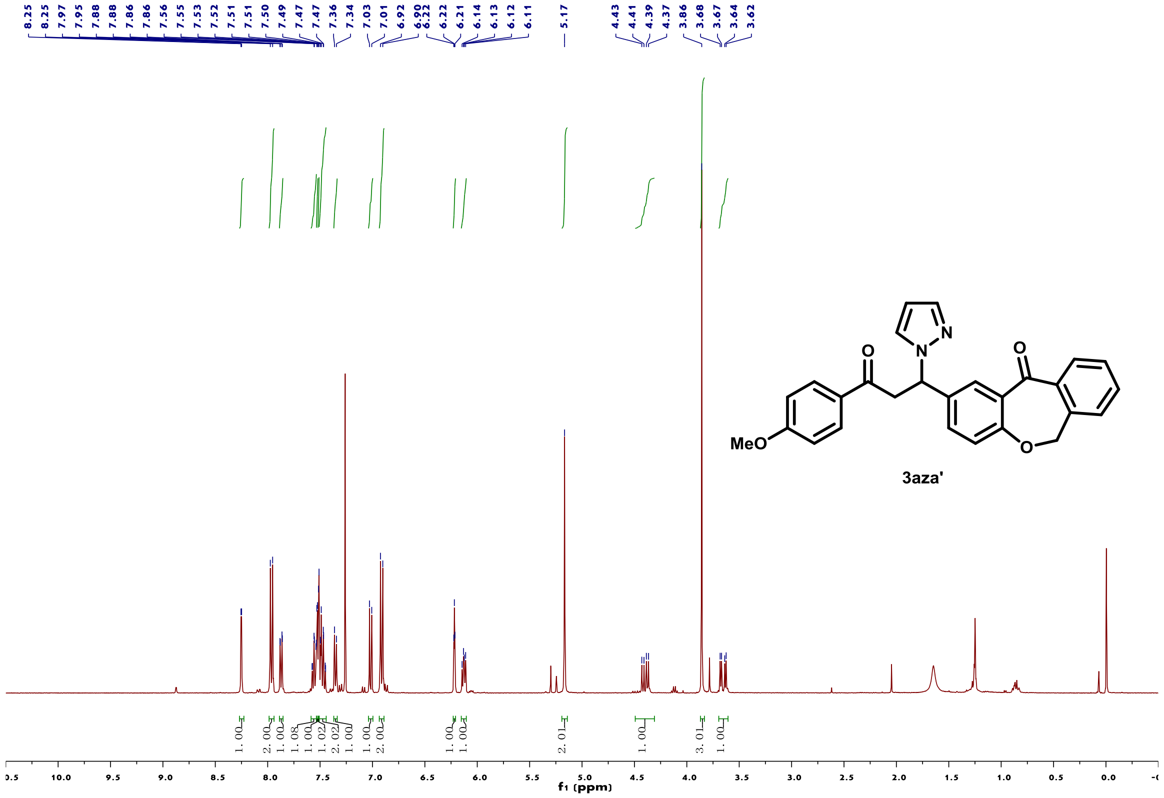

Supplementary Figure 217. ^{1}H NMR (400 MHz, $CDCl_3$) spectrum for 3aza’

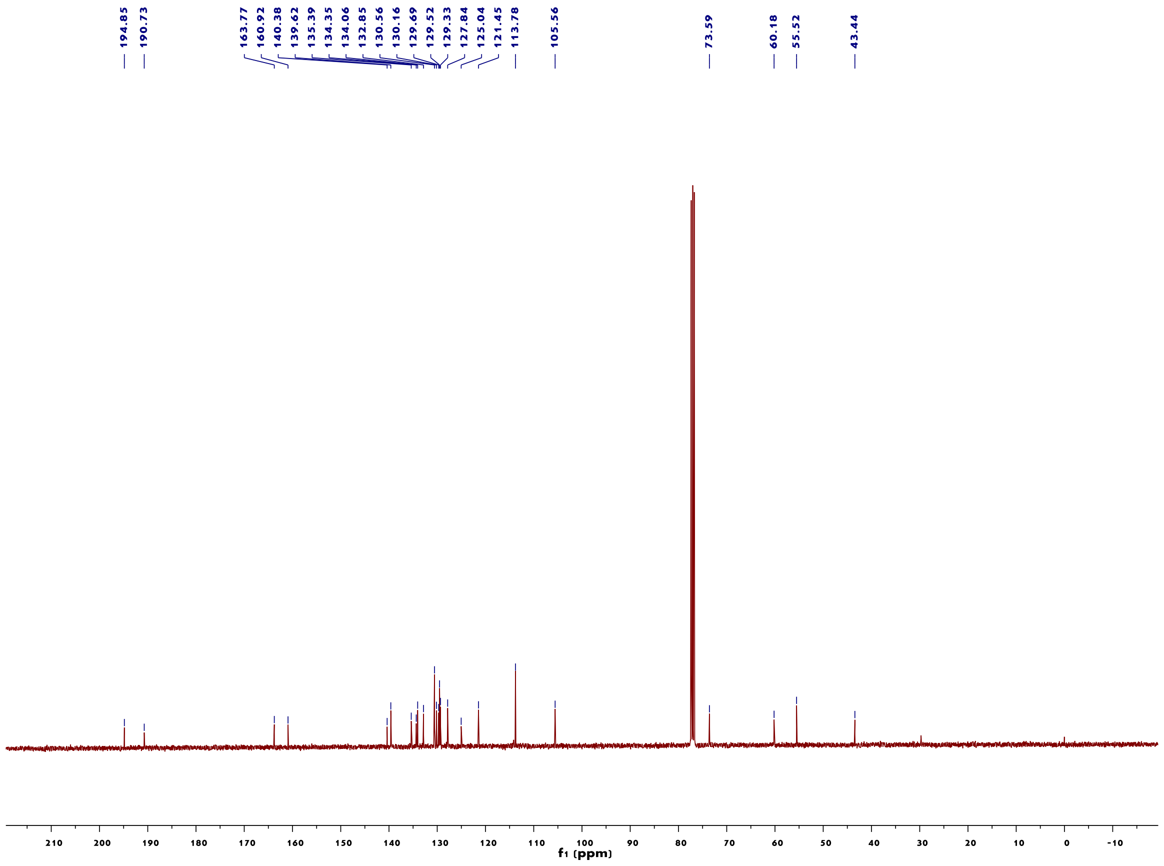

Supplementary Figure 218. ^{13}C NMR (100 MHz, $CDCl_3$) spectrum for 3aza'

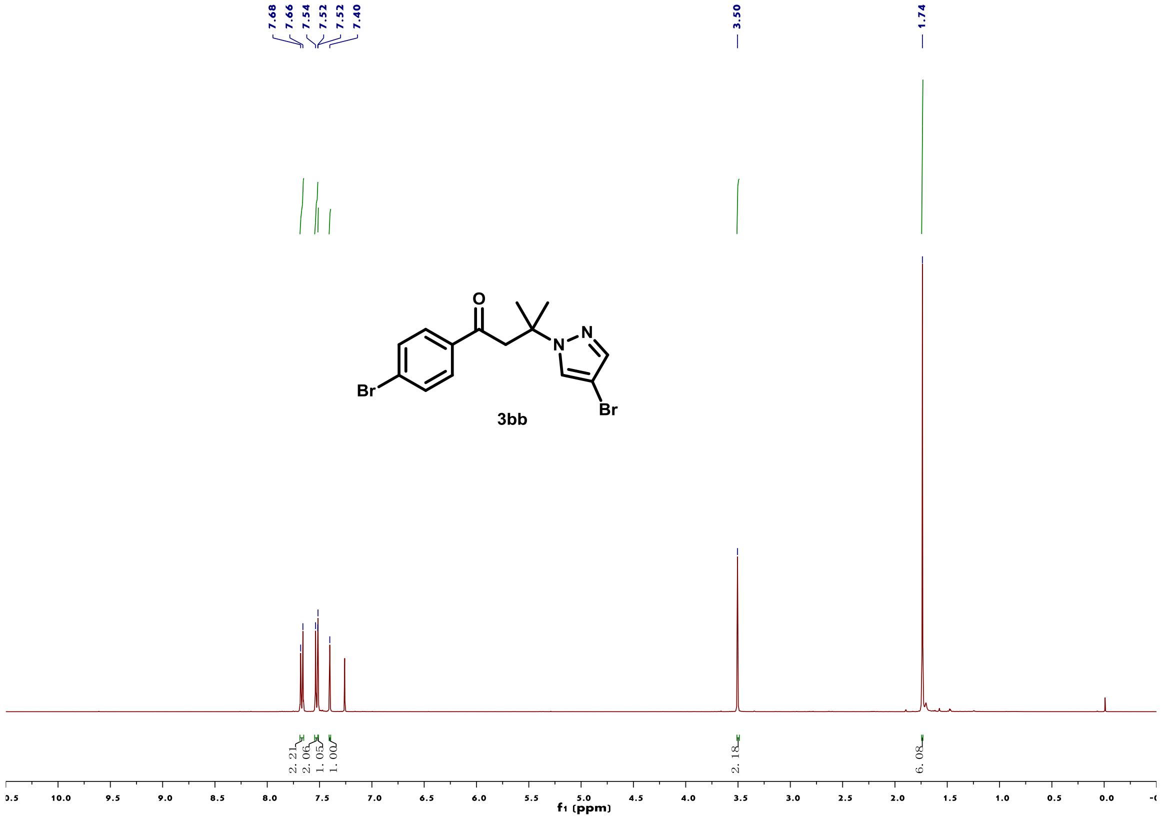

Supplementary Figure 219. ^{1}H NMR (400 MHz, $CDCl_3$) spectrum for 3bb

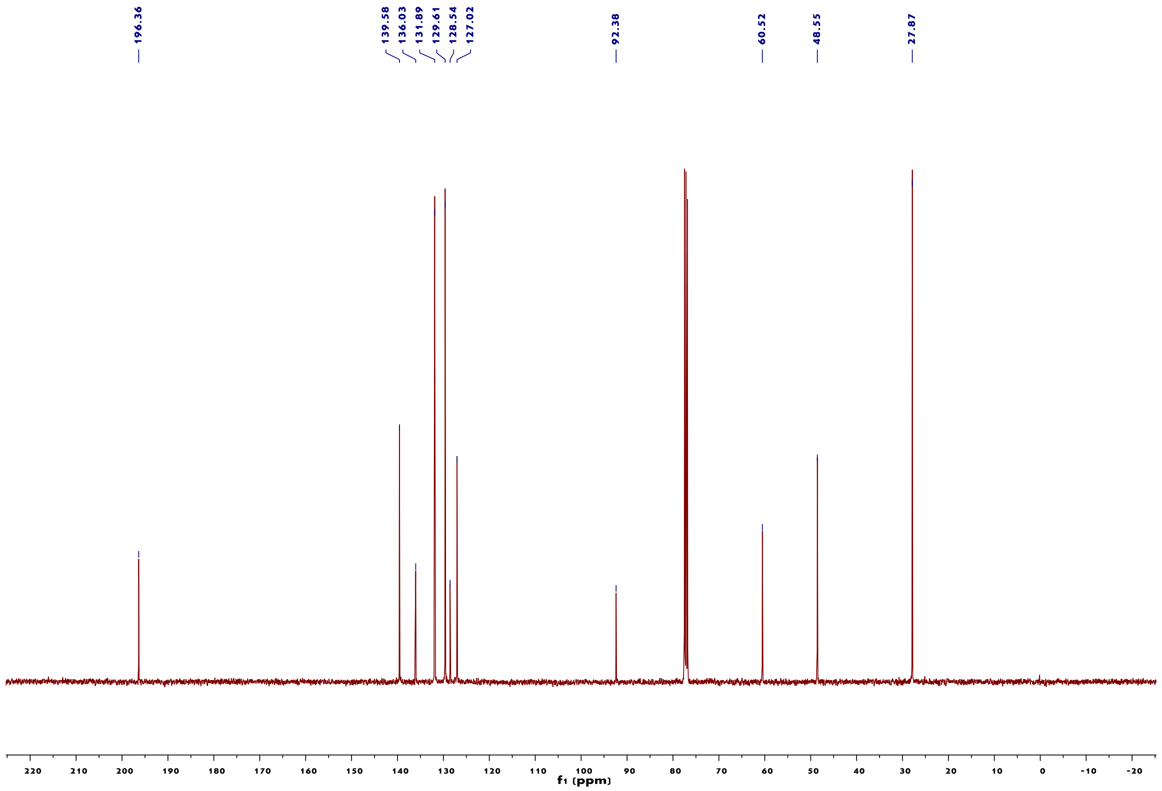

Supplementary Figure 220. ^{13}C NMR (100 MHz, $CDCl_3$) spectrum for 3bb

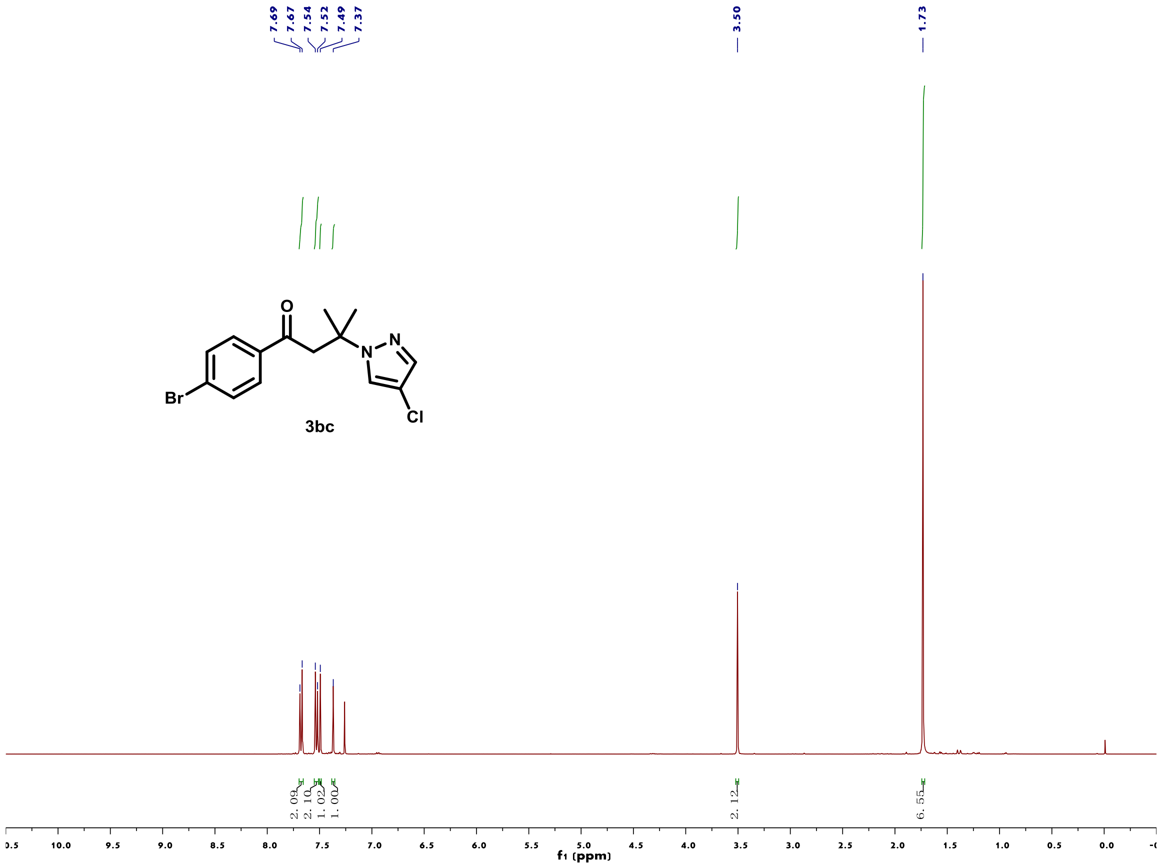

3bc

Supplementary Figure 221. ^{1}H NMR (400 MHz, $CDCl_3$) spectrum for 3bc

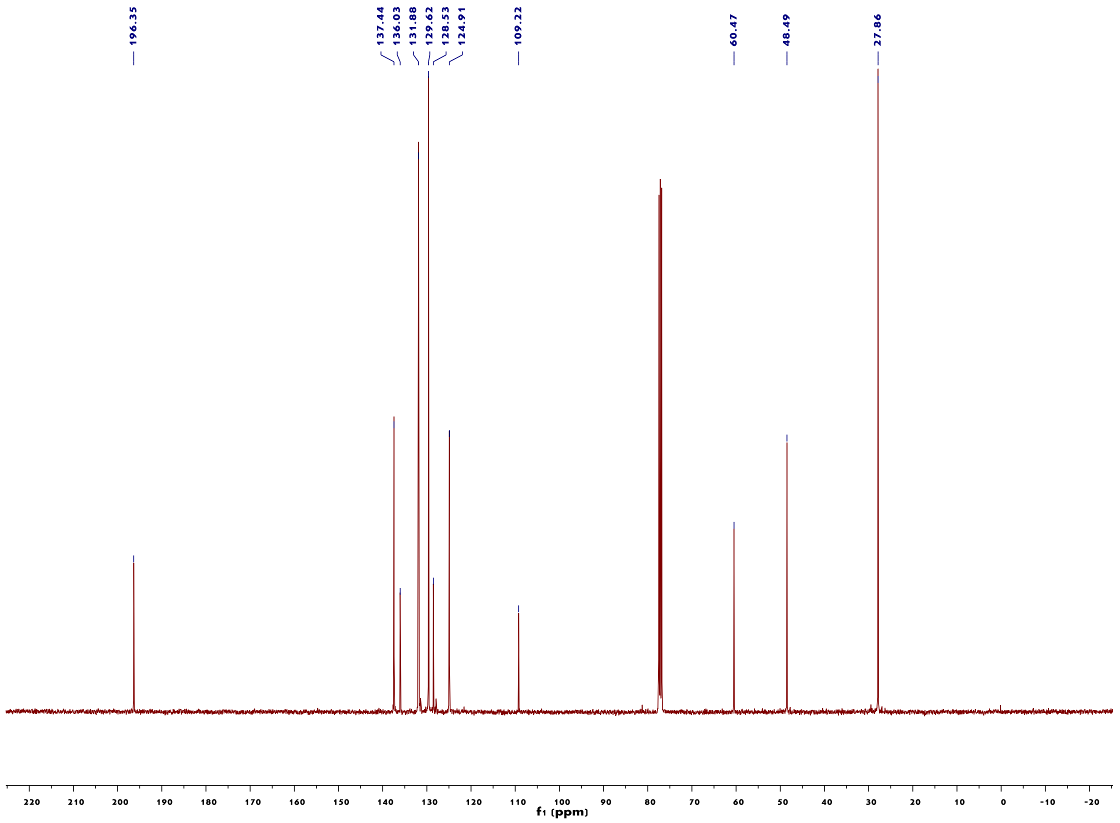

Supplementary Figure 222. ^{13}C NMR (100 MHz, $CDCl_3$) spectrum for 3bc

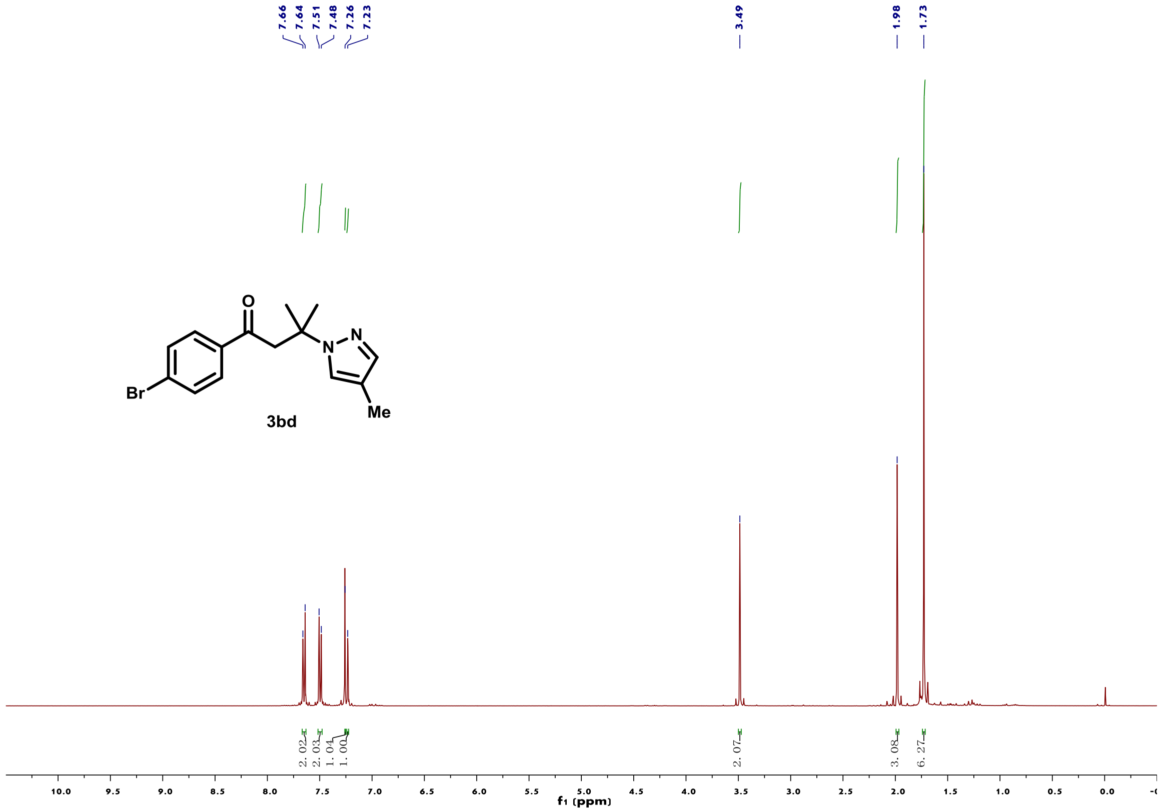

Supplementary Figure 223. ^{1}H NMR (400 MHz, $CDCl_3$) spectrum for 3bd

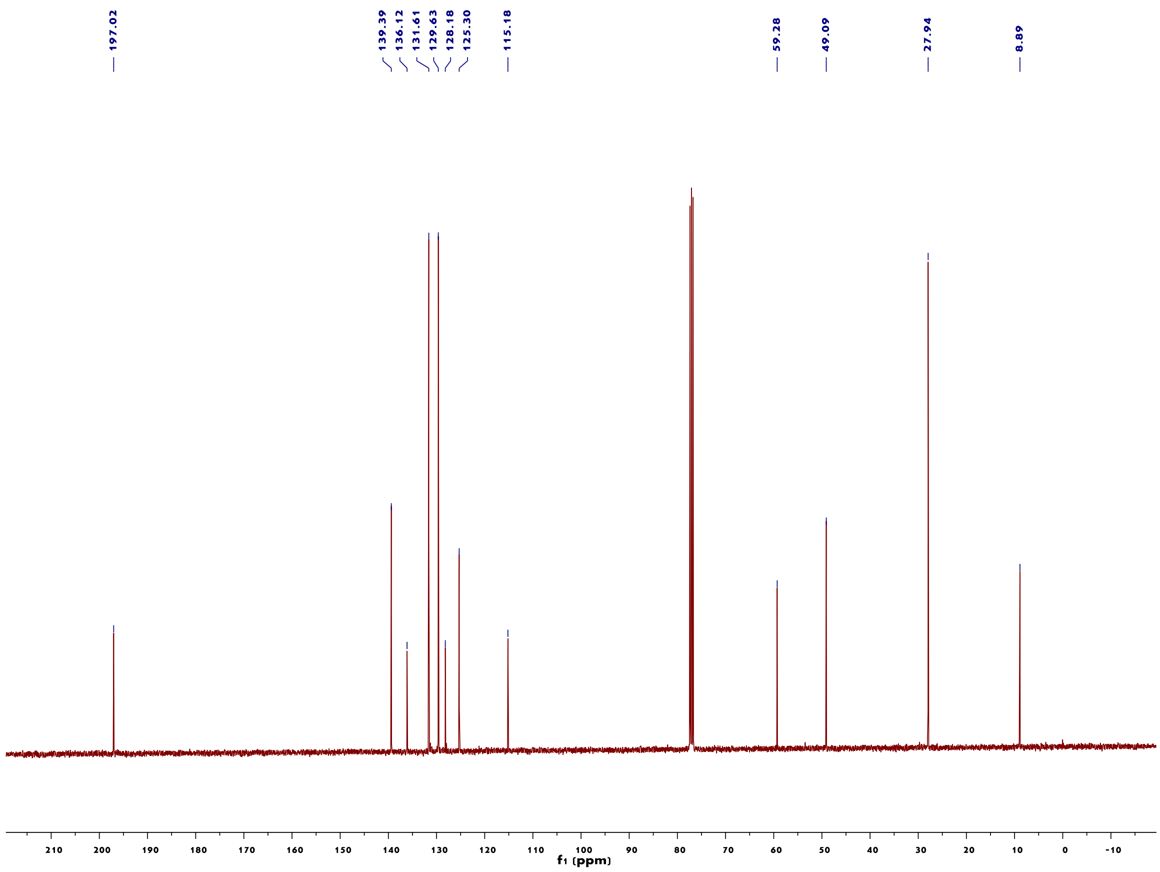

Supplementary Figure 224. ^{13}C NMR (100 MHz, $CDCl_3$) spectrum for 3bd

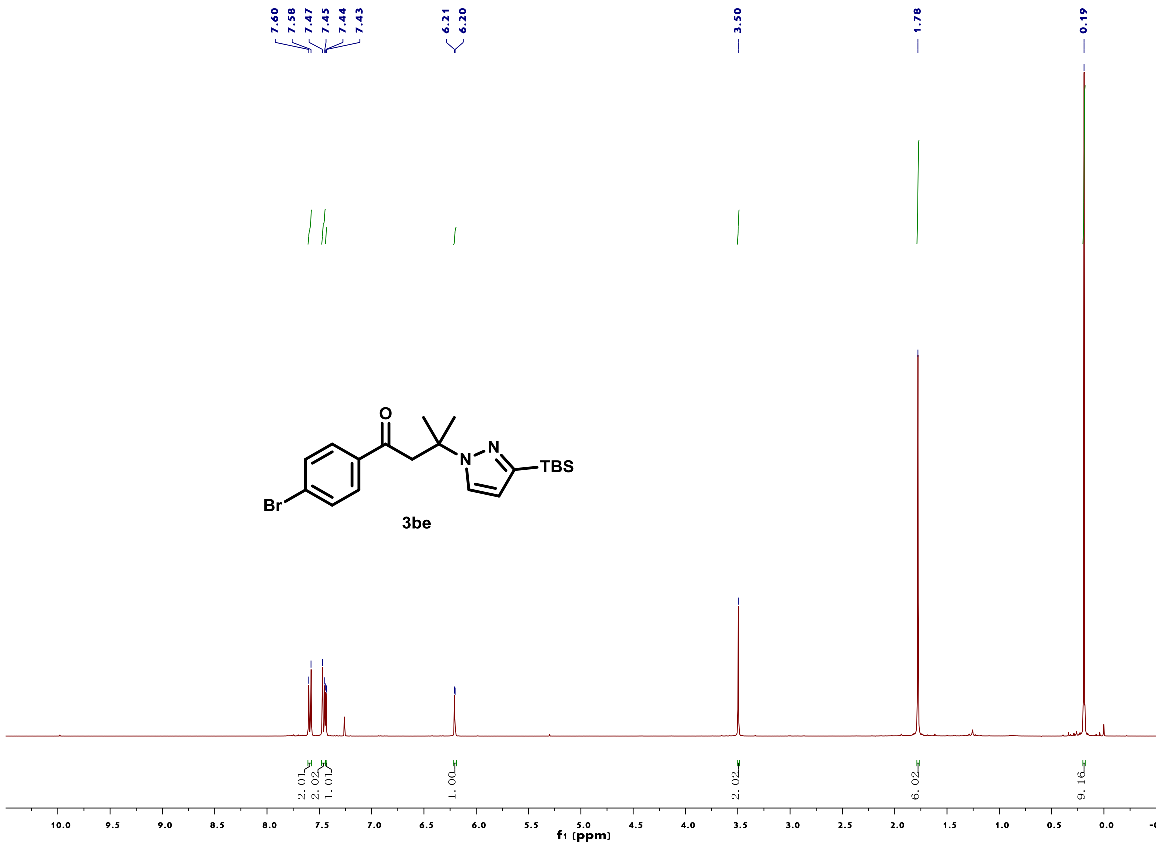

Supplementary Figure 225. ^{1}H NMR (400 MHz, $CDCl_3$) spectrum for 3be

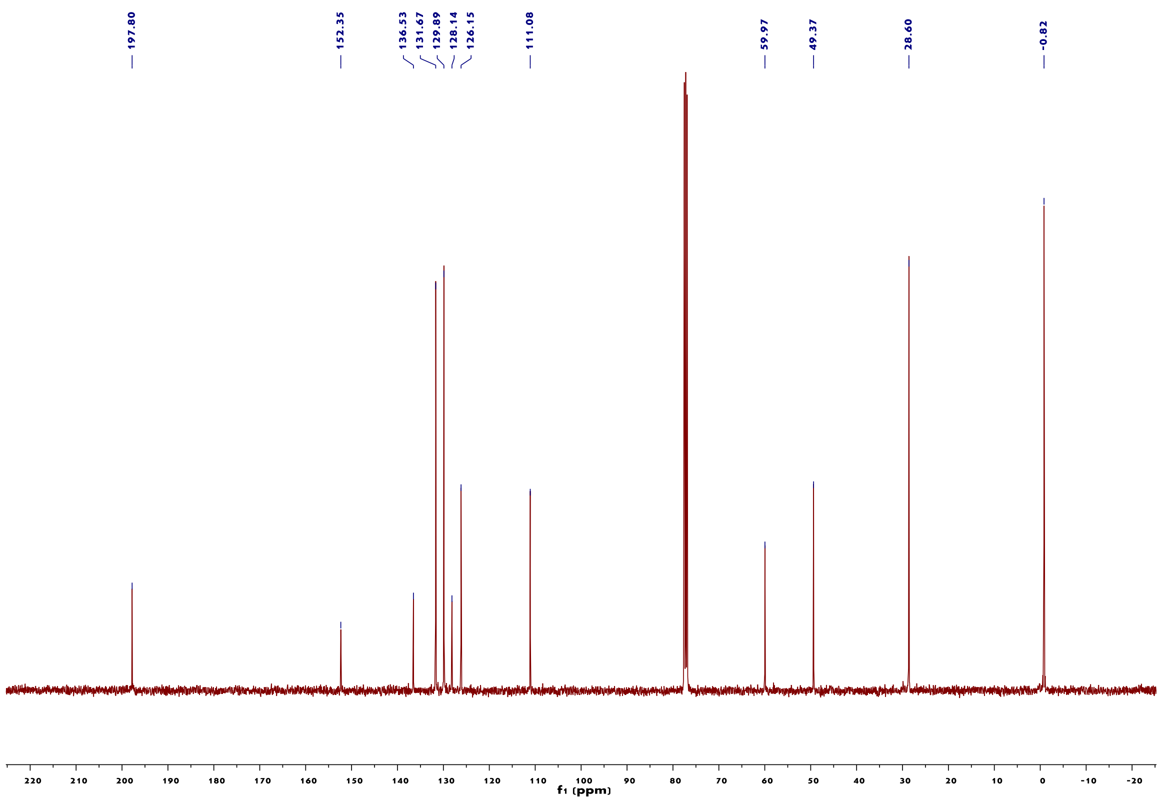

Supplementary Figure 226. ^{13}C NMR (100 MHz, $CDCl_3$) spectrum for 3be

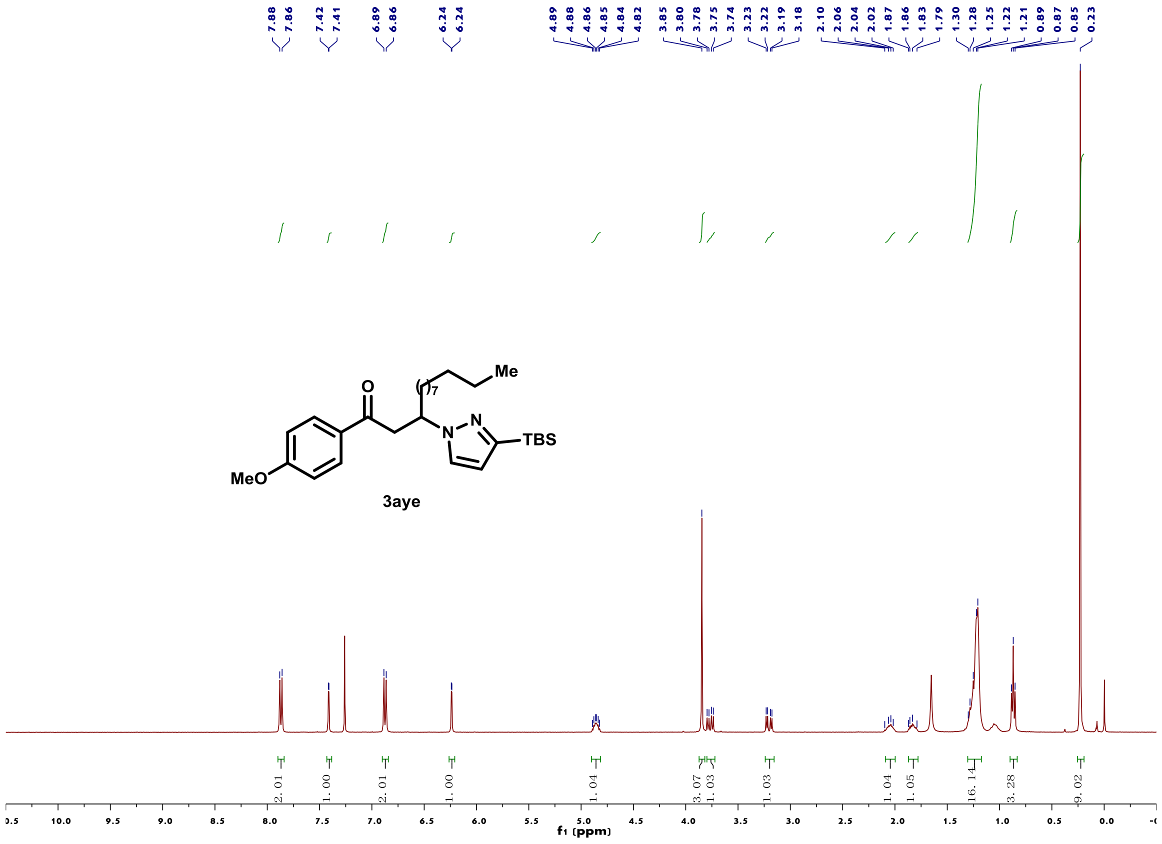

Supplementary Figure 227. ^{1}H NMR (400 MHz, $CDCl_3$) spectrum for 3aye

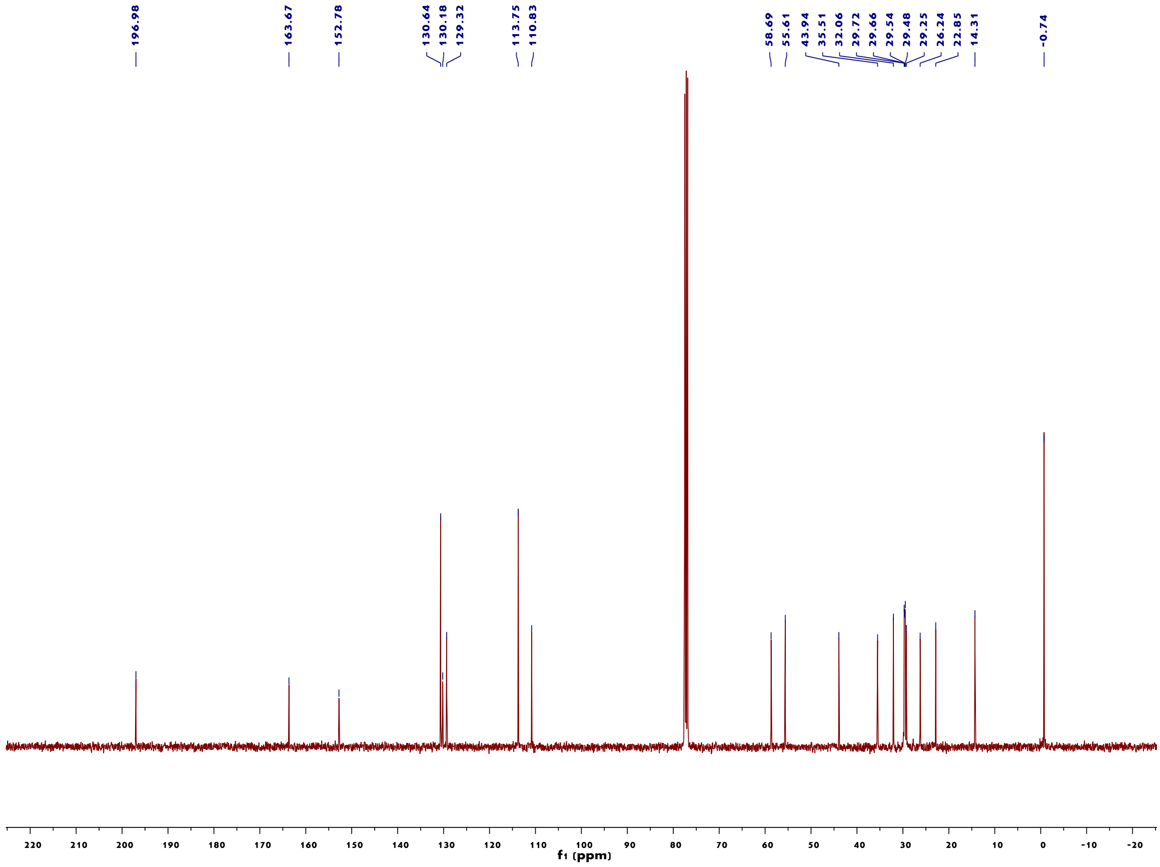

Supplementary Figure 228. ^{13}C NMR (100 MHz, $CDCl_3$) spectrum for 3aye

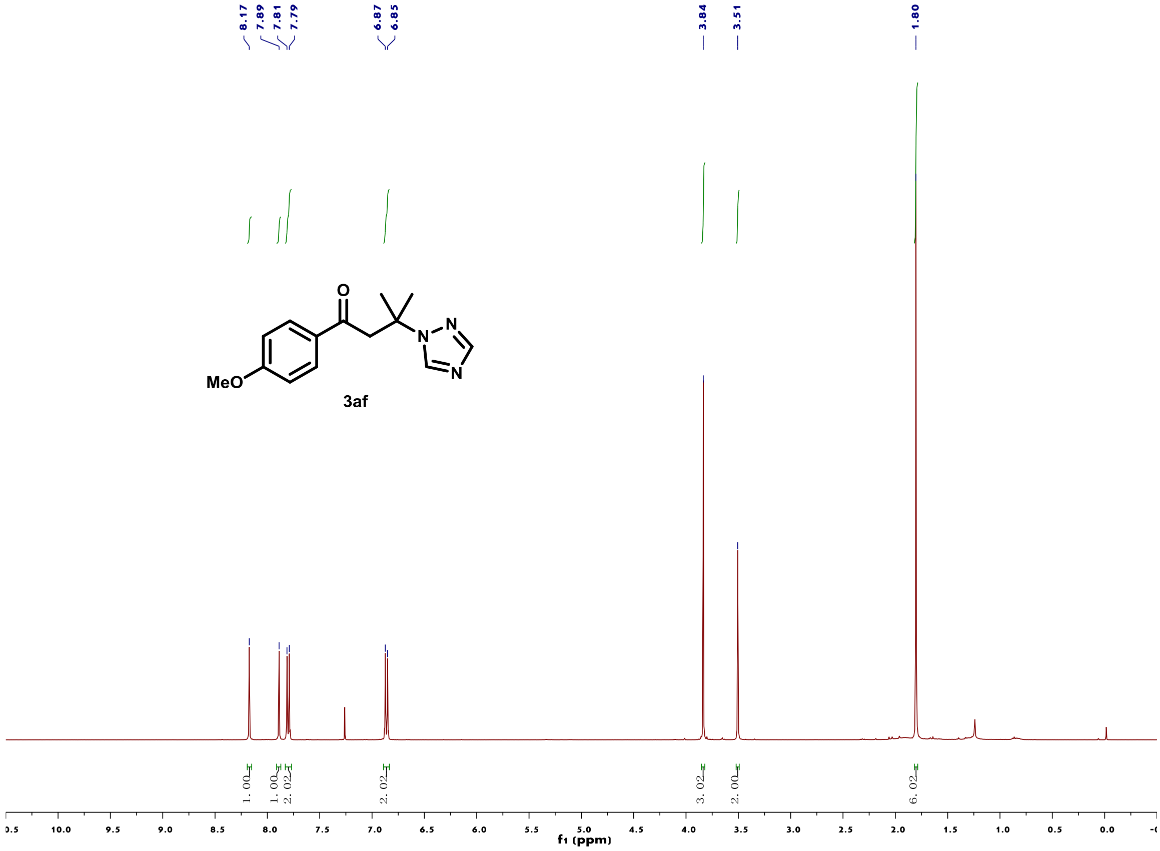

Supplementary Figure 229. ^{1}H NMR (400 MHz, $CDCl_3$) spectrum for 3af

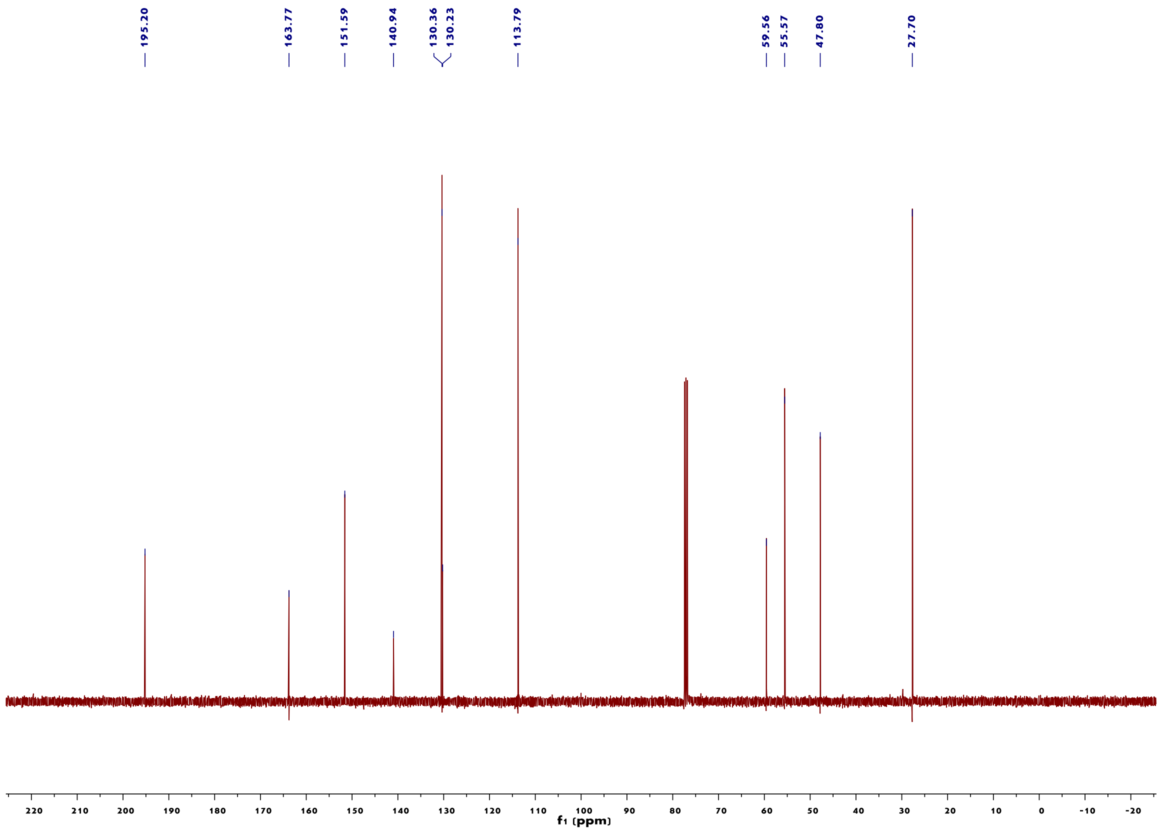

Supplementary Figure 230. ^{13}C NMR (100 MHz, $CDCl_3$) spectrum for 3af

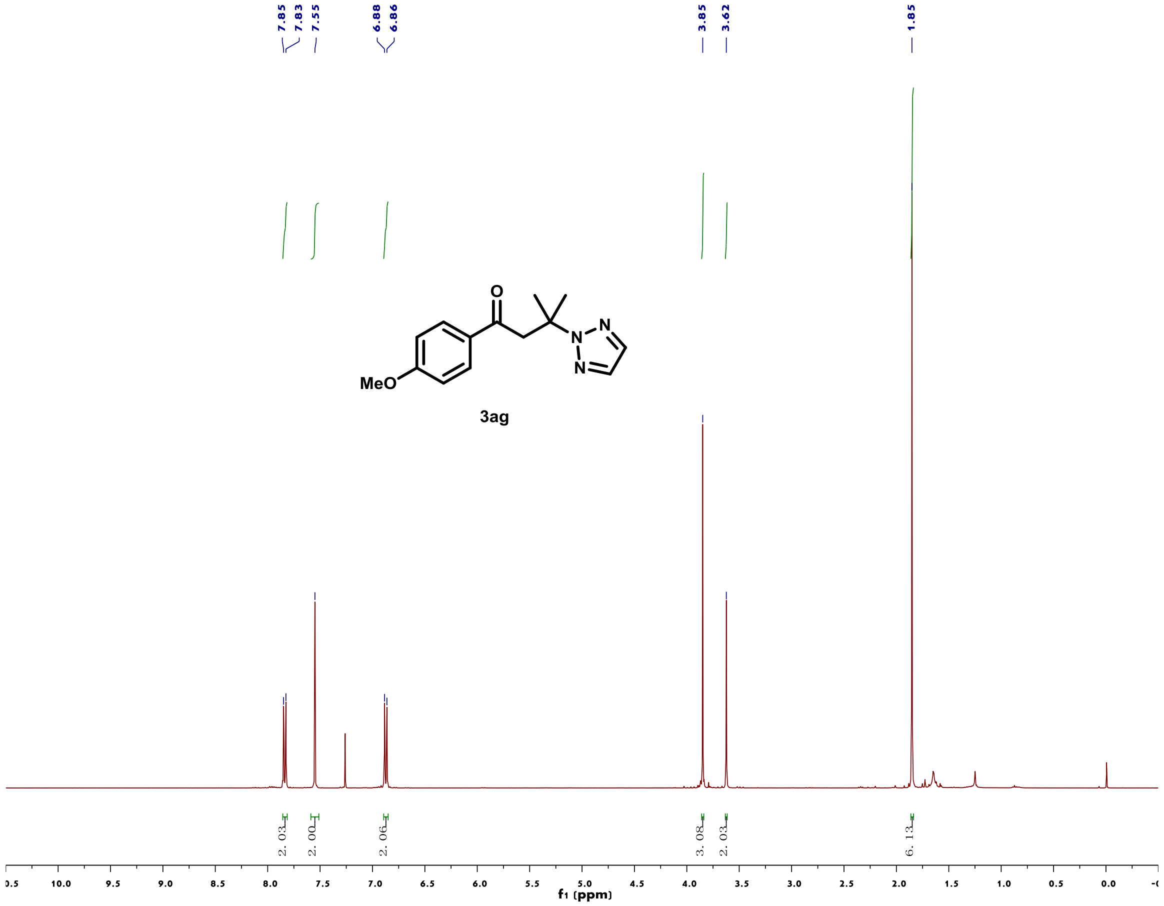

Supplementary Figure 231. ^{1}H NMR (400 MHz, $CDCl_3$) spectrum for 3ag

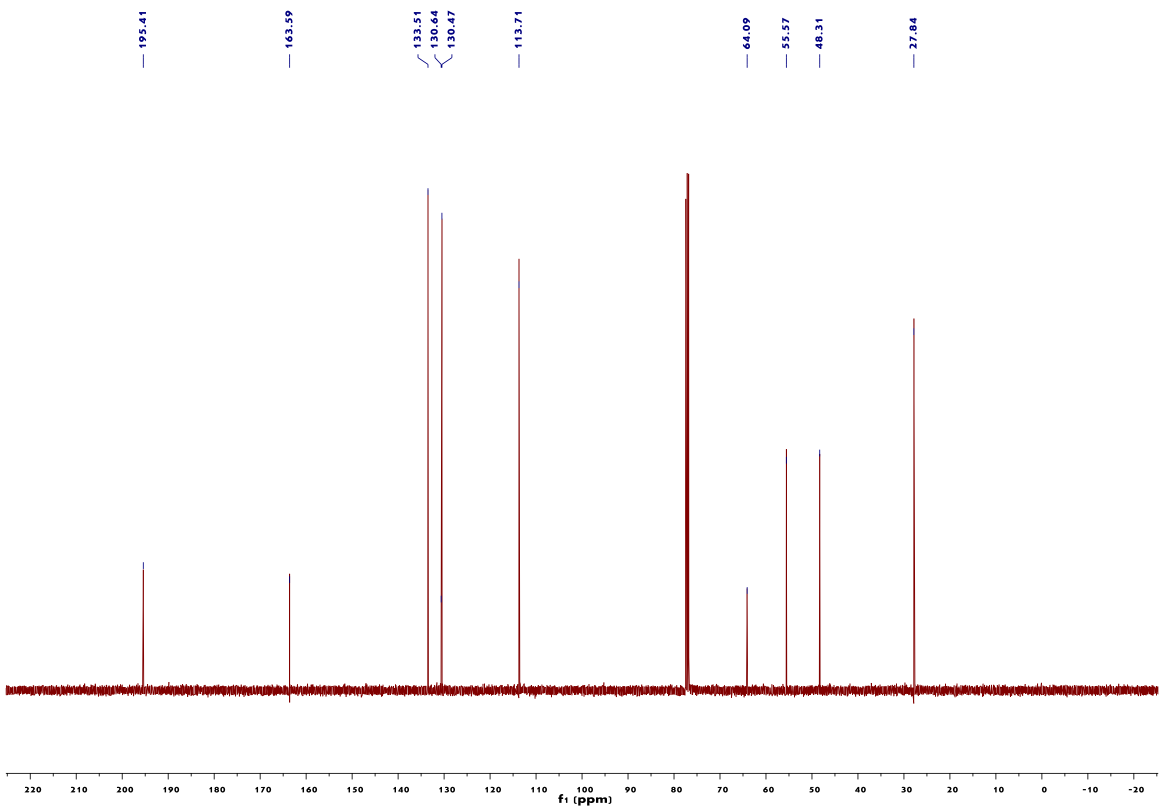

Supplementary Figure 232. ^{13}C NMR (100 MHz, $CDCl_3$) spectrum for 3ag

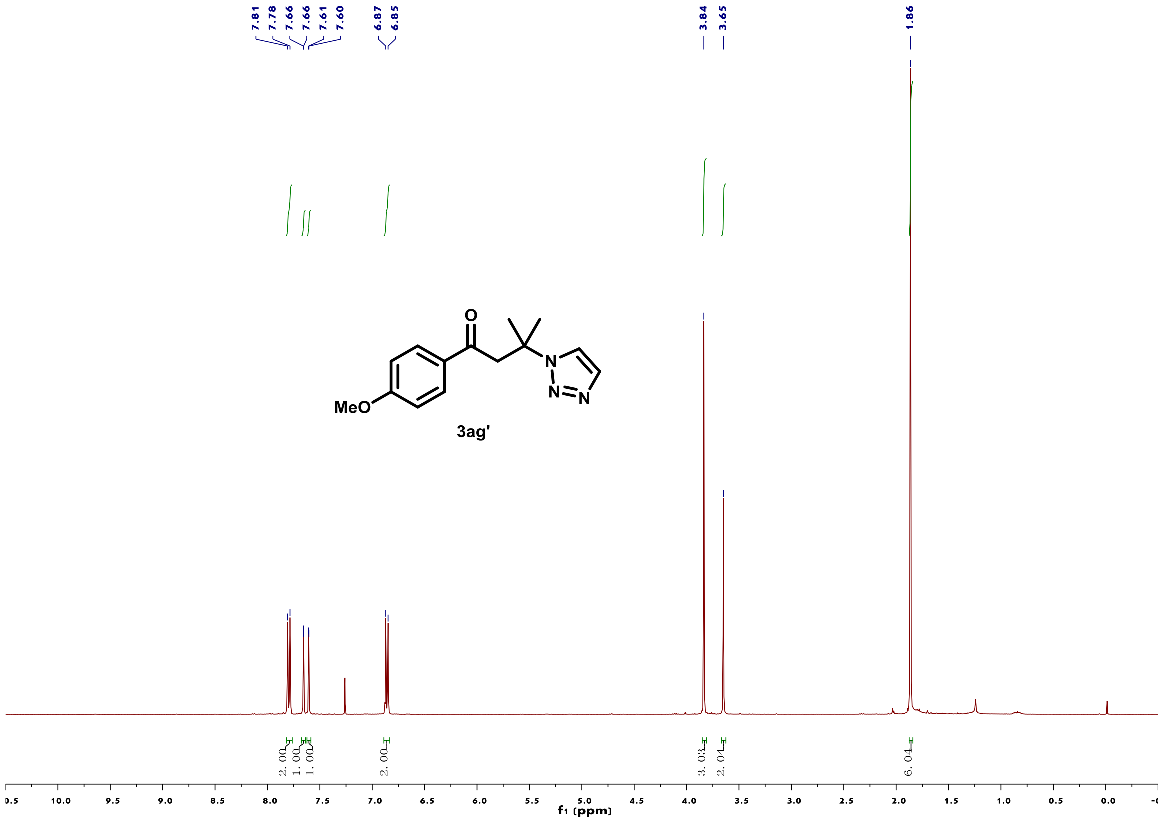

Supplementary Figure 233. ^{1}H NMR (400 MHz, $CDCl_3$) spectrum for 3ag’

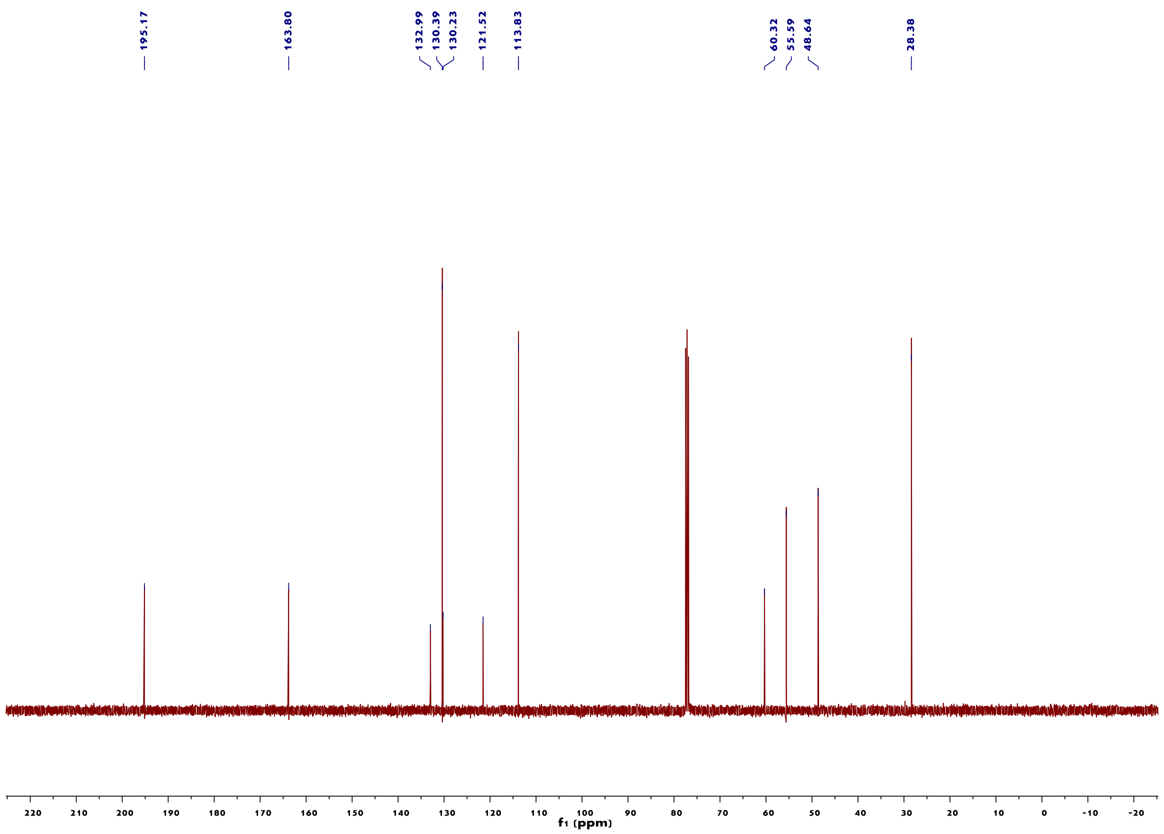

Supplementary Figure 234. ^{13}C **NMR (100 MHz,** $CDCl_3$**) spectrum for 3ag'**

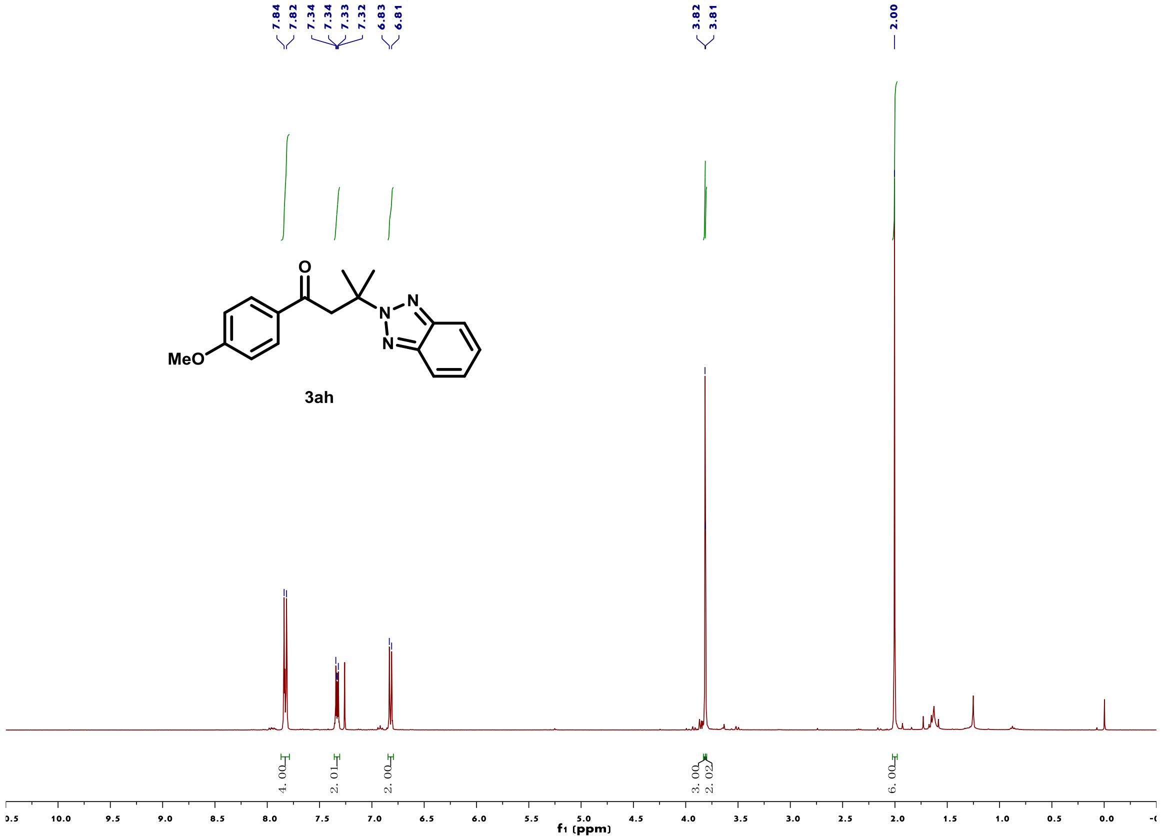

Supplementary Figure 235. ^{1}H **NMR (400 MHz,** $CDCl_3$**) spectrum for 3ah**

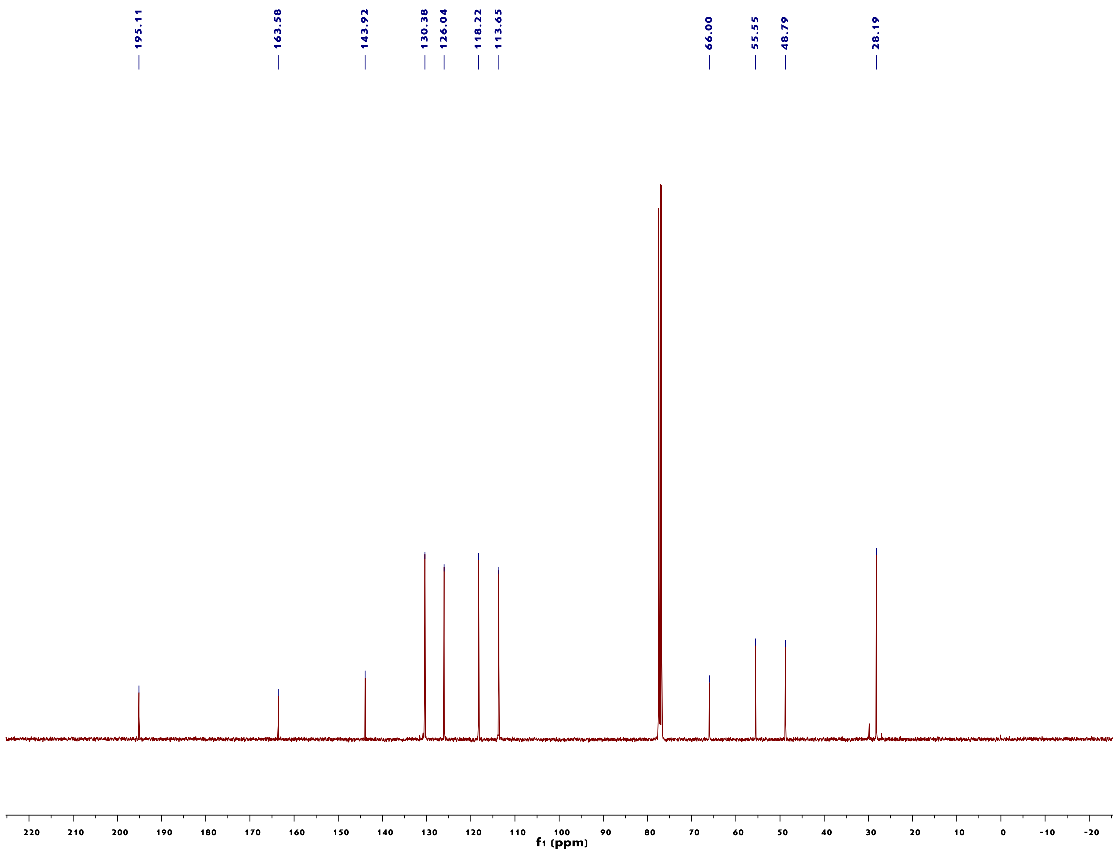

Supplementary Figure 236. ^{13}C NMR (100 MHz, $CDCl_3$) spectrum for 3ah

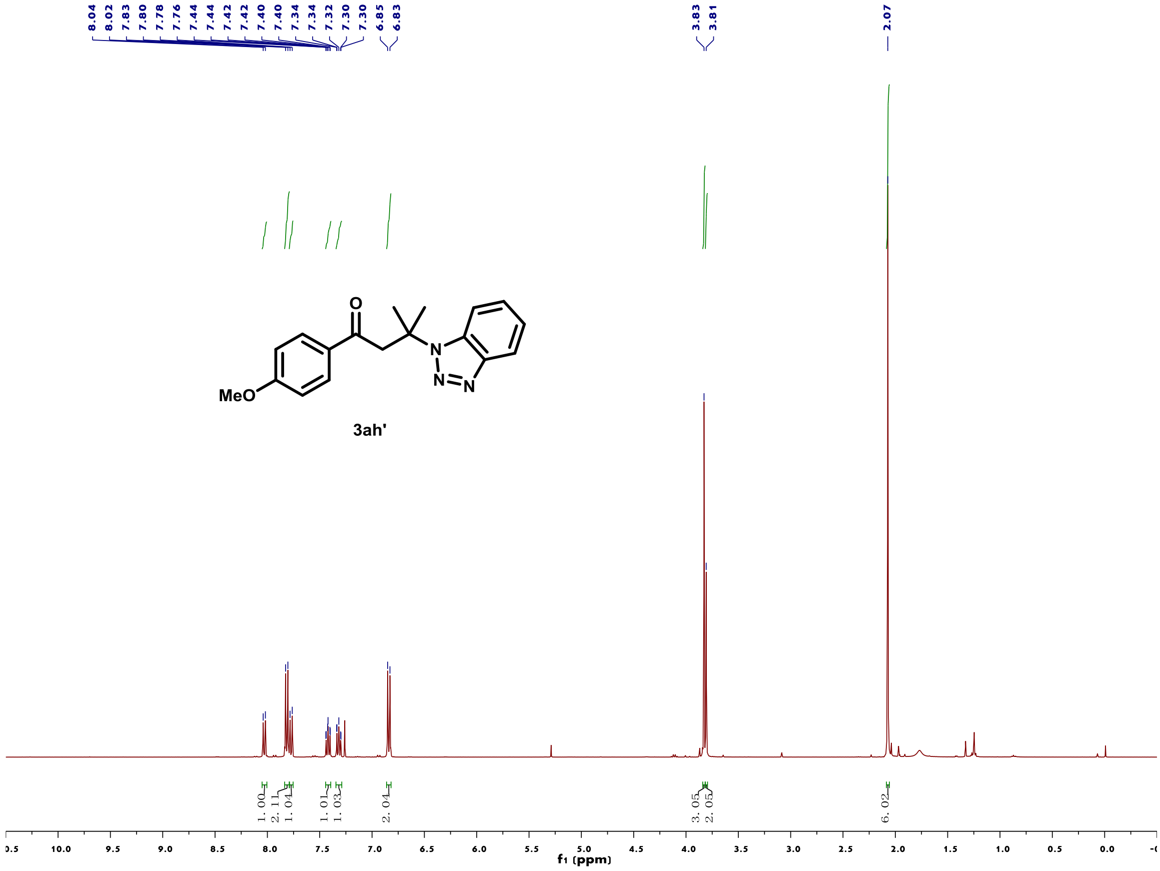

Supplementary Figure 237. ^{1}H NMR (400 MHz, $CDCl_3$) spectrum for 3ah’

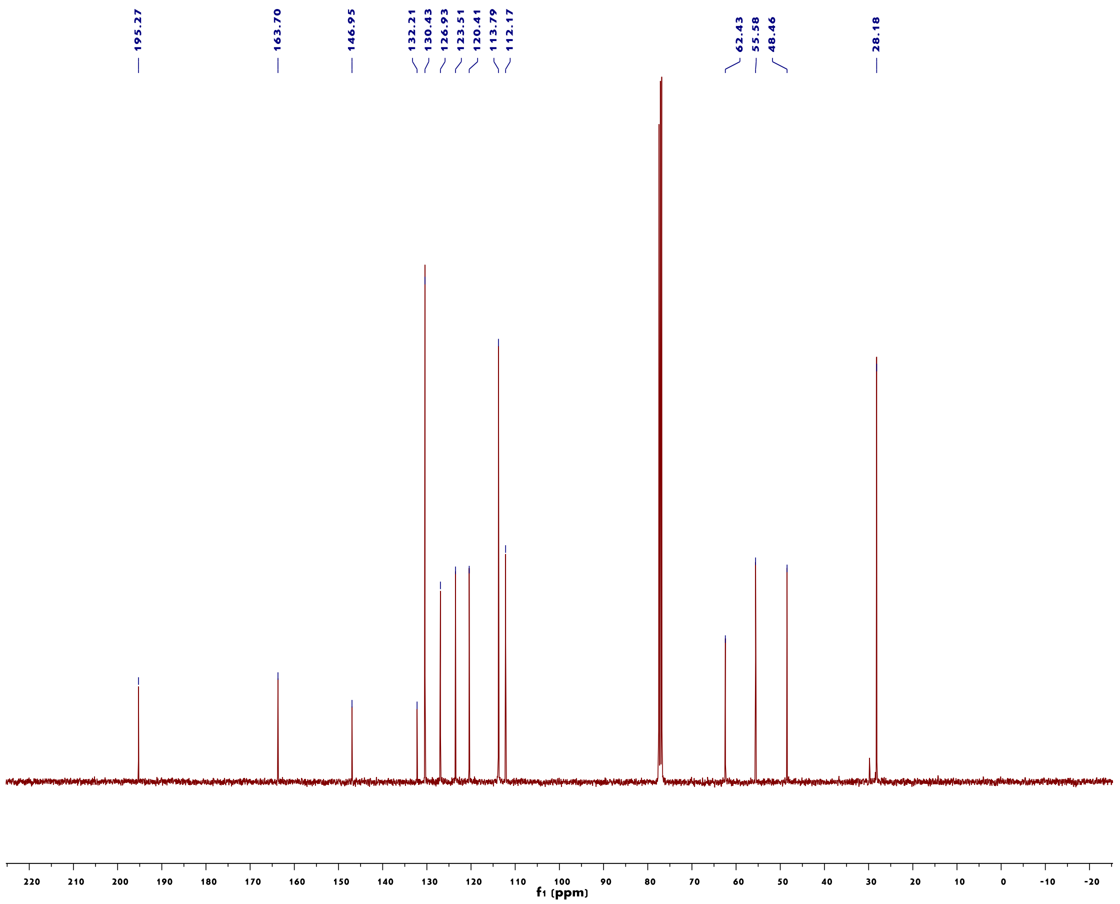

Supplementary Figure 238. ^{13}C NMR (100 MHz, $CDCl_3$) spectrum for 3ah'

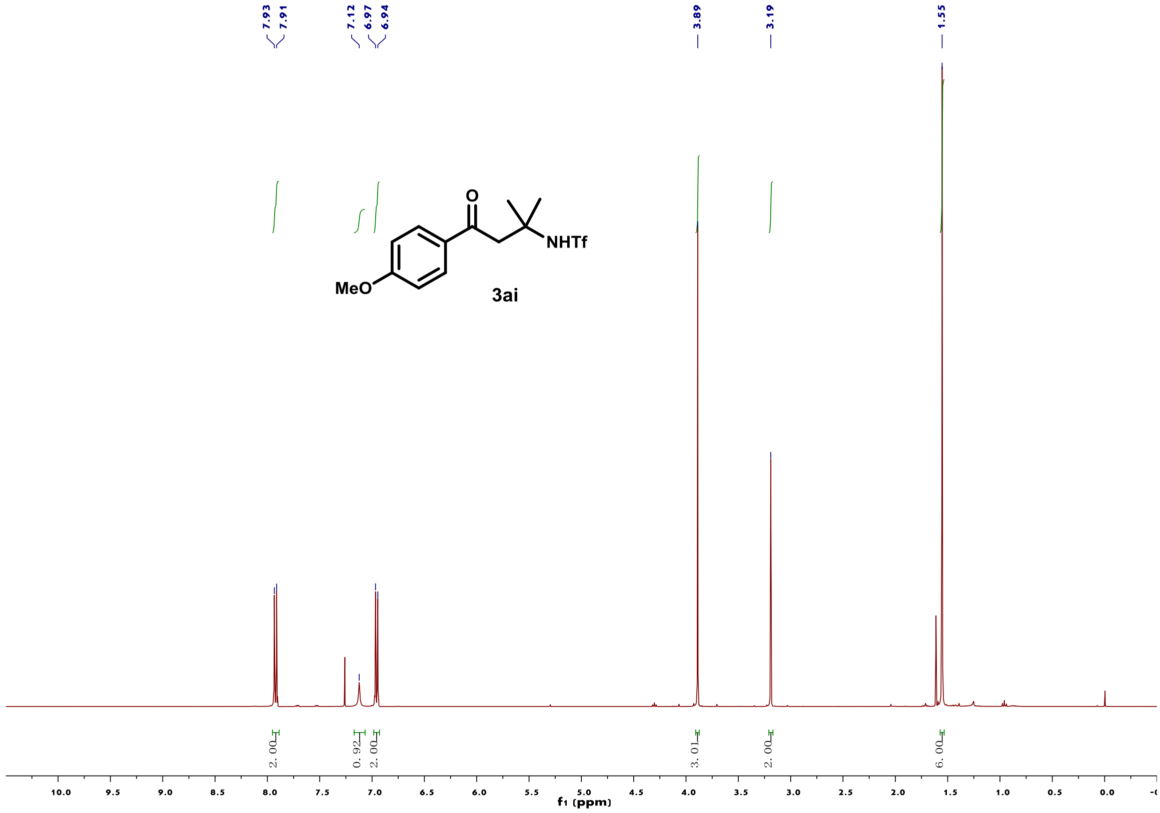

Supplementary Figure 239. ^{1}H NMR (400 MHz, $CDCl_3$) spectrum for 3ai

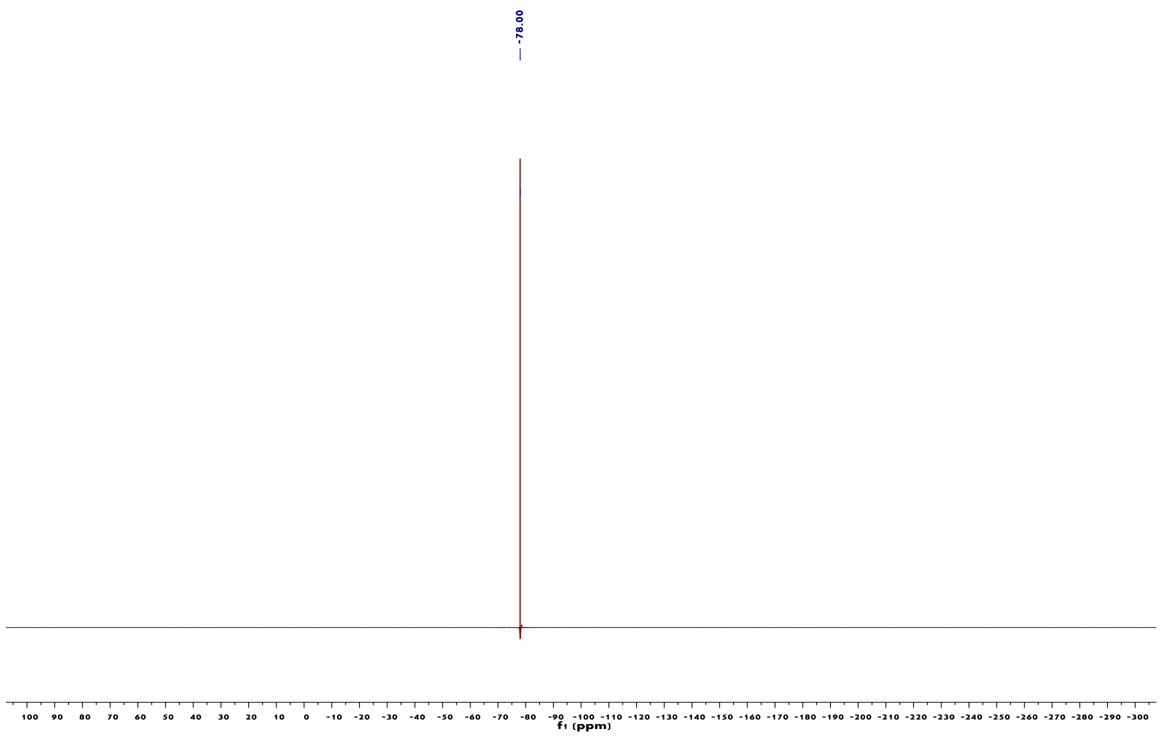

Supplementary Figure 240. ^{19}F NMR (376 MHz, $CDCl_3$) spectrum for 3ai

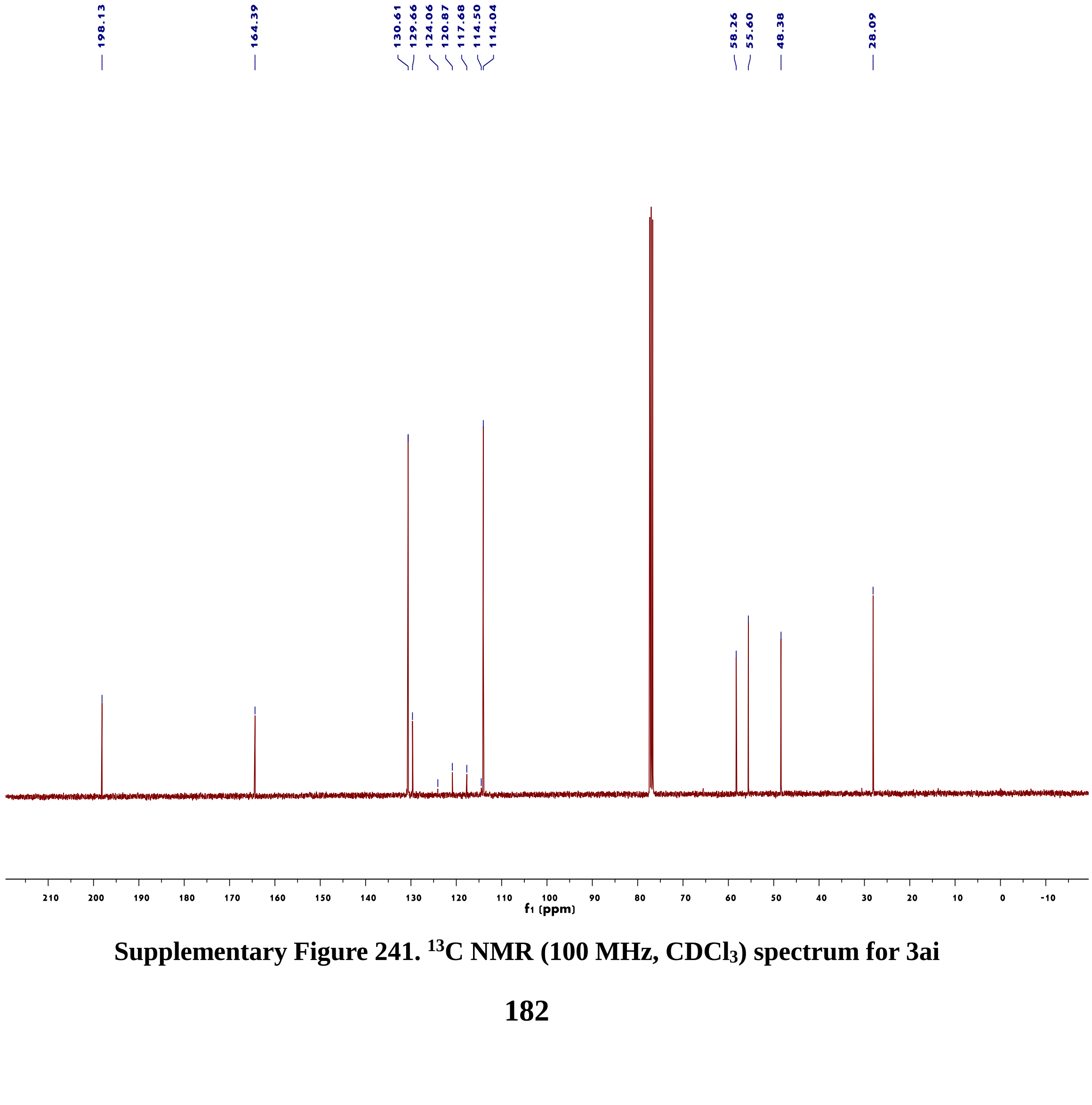

Supplementary Figure 241. ^{13}C NMR (100 MHz, $CDCl_3$) spectrum for 3ai

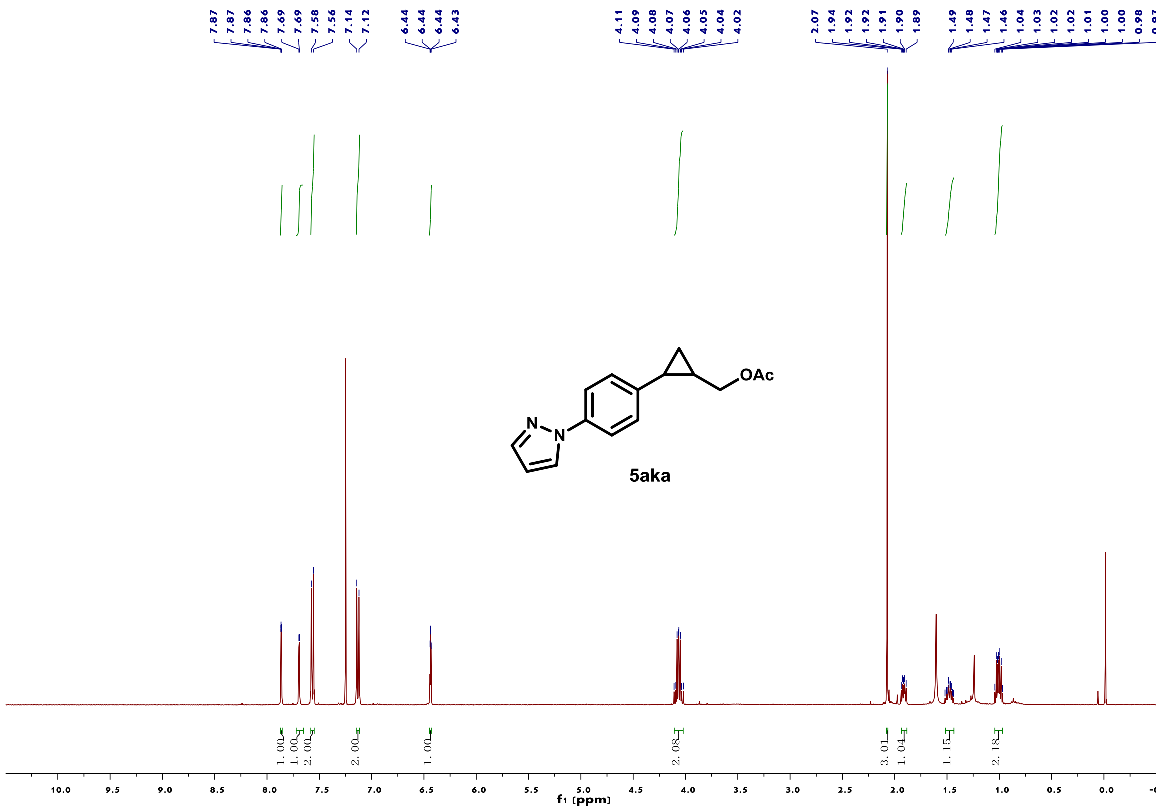

Supplementary Figure 242. ^{1}H NMR (400 MHz, $CDCl_3$) spectrum for 5aka

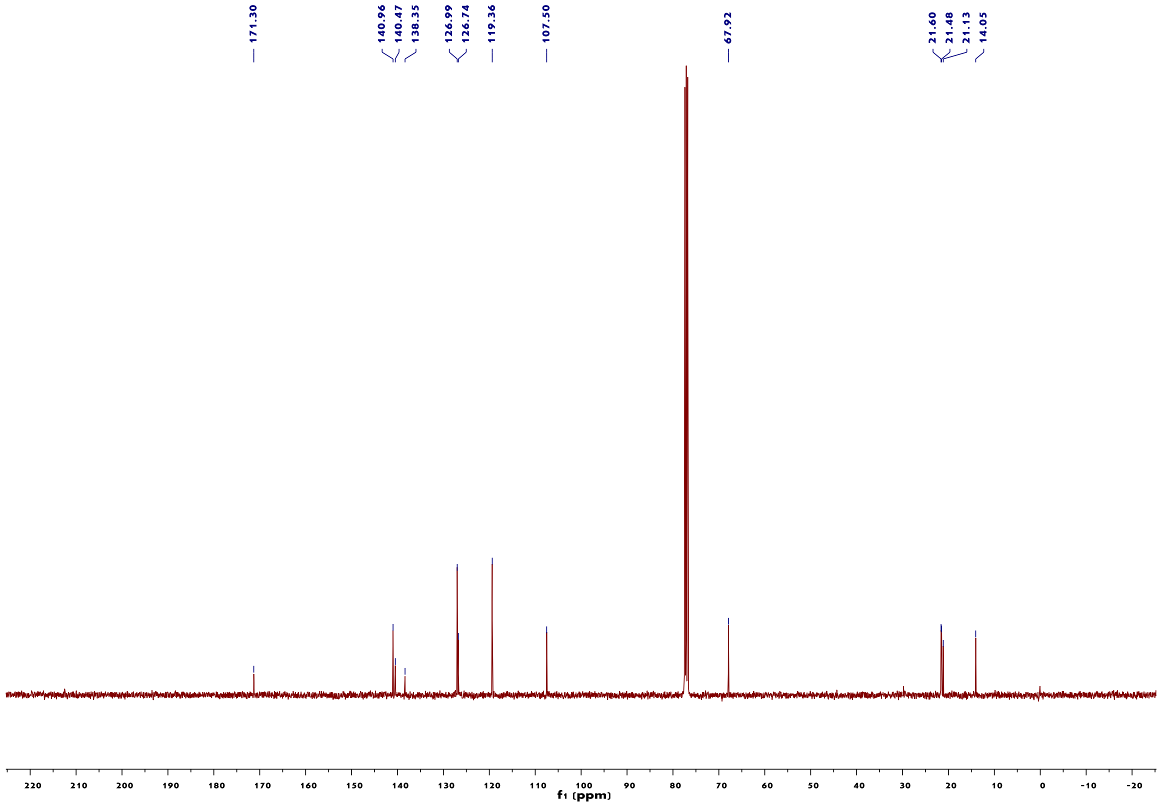

Supplementary Figure 243. ^{13}C NMR (100 MHz, $CDCl_3$) spectrum for 5aka

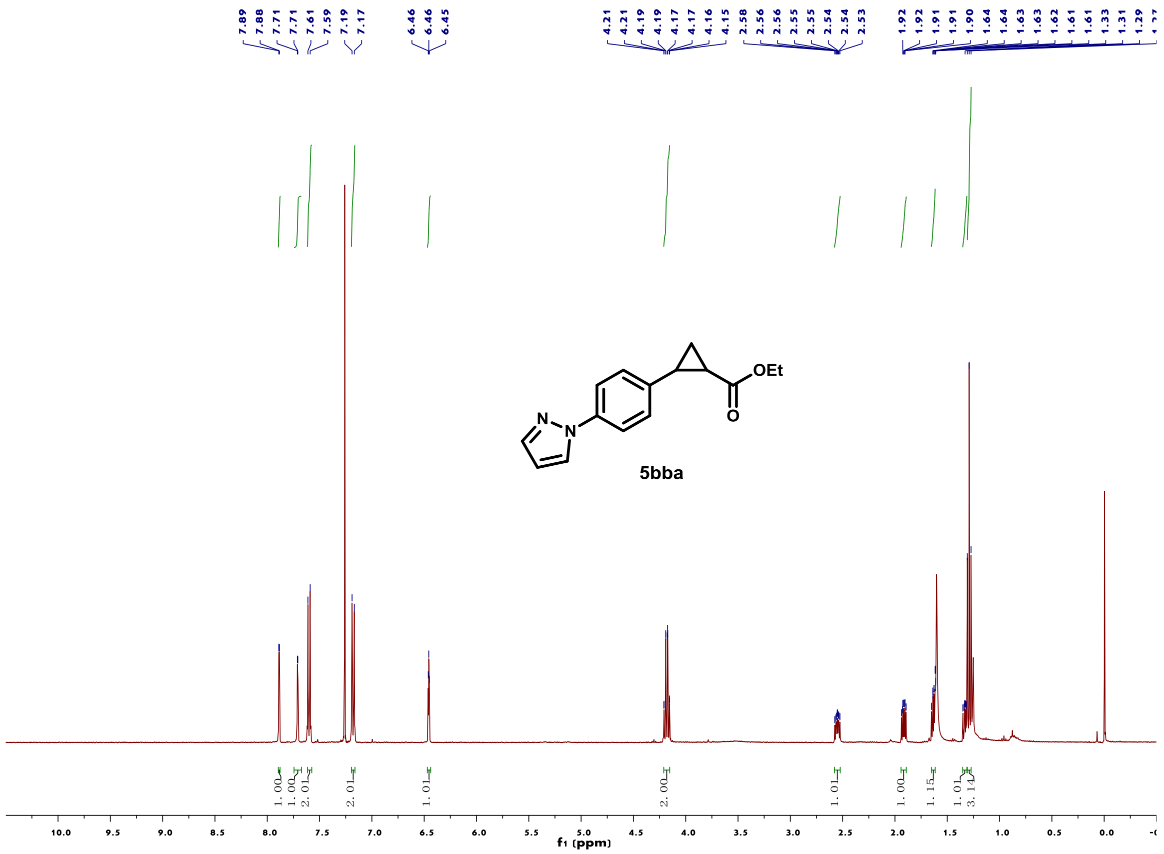

Supplementary Figure 244. ^{1}H NMR (400 MHz, $CDCl_3$) spectrum for 5bba

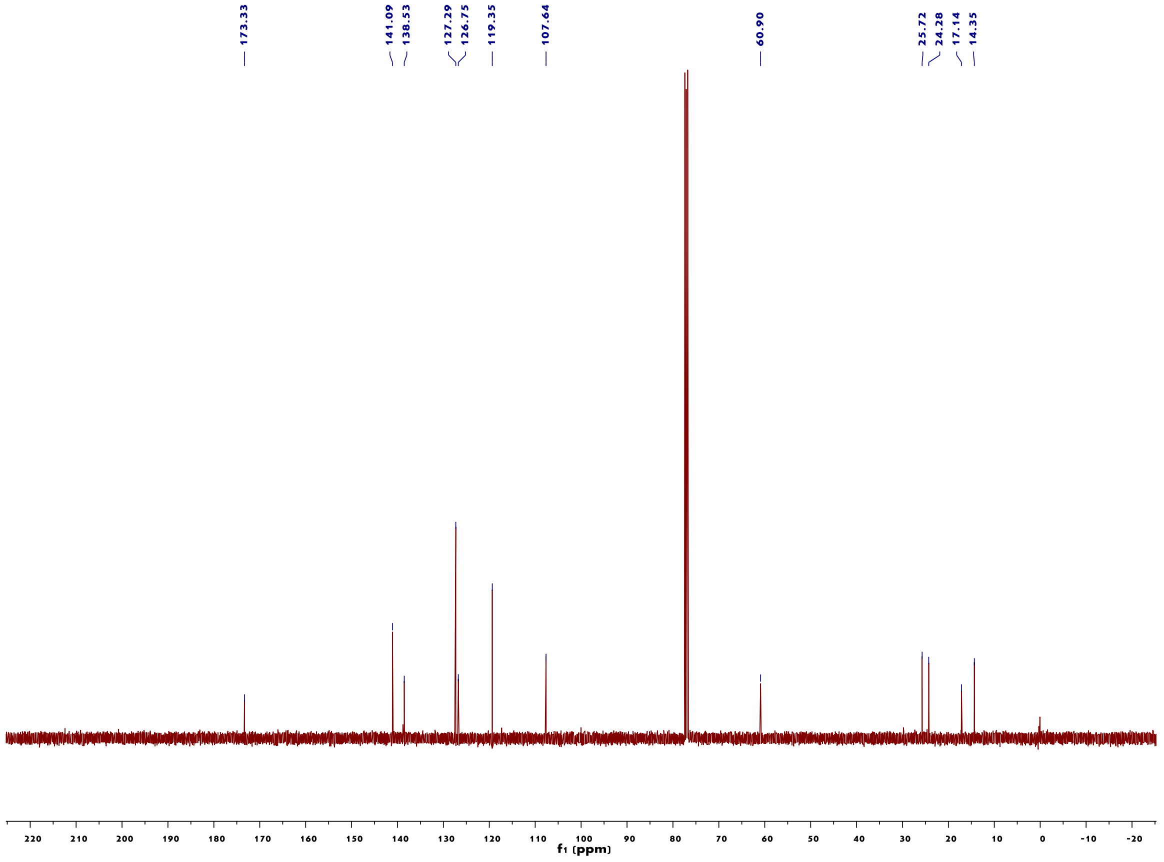

Supplementary Figure 245. ^{13}C NMR (100 MHz, $CDCl_3$) spectrum for 5bba

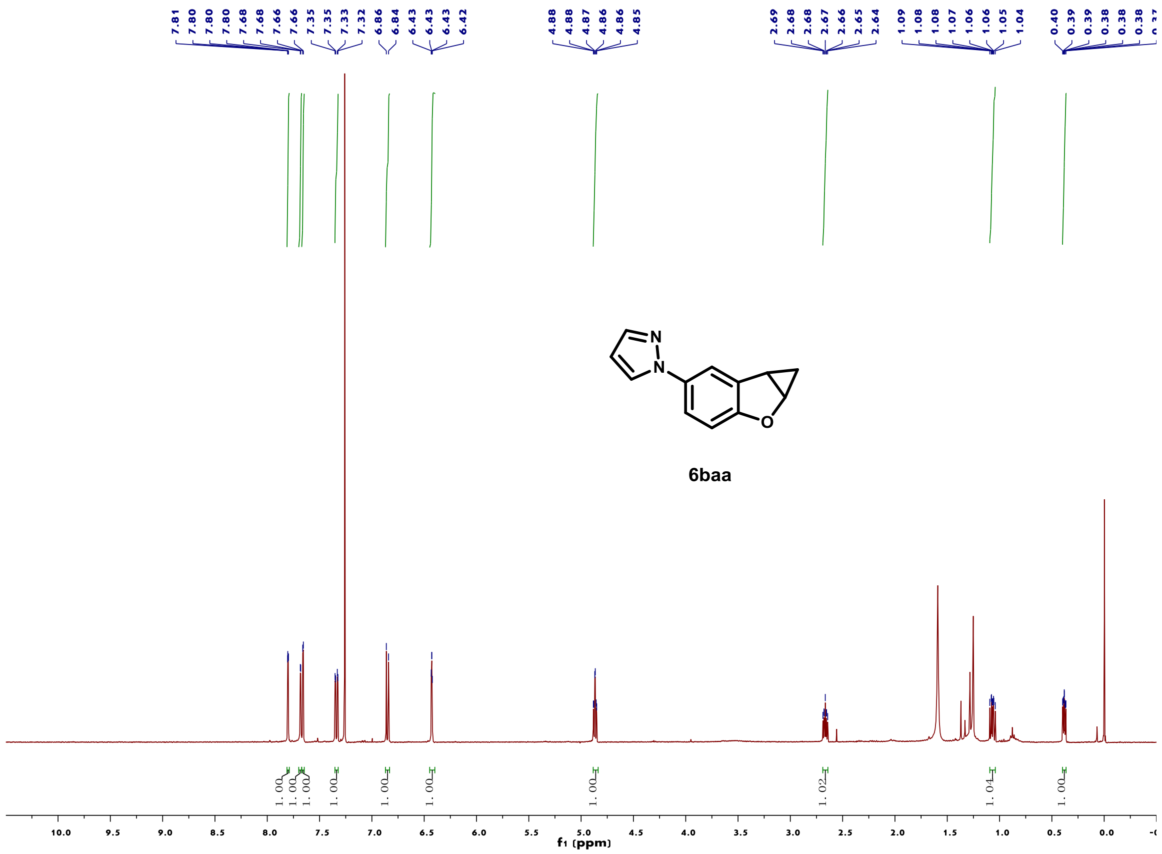

Supplementary Figure 246. ^{1}H NMR (400 MHz, $CDCl_3$) spectrum for 6baa

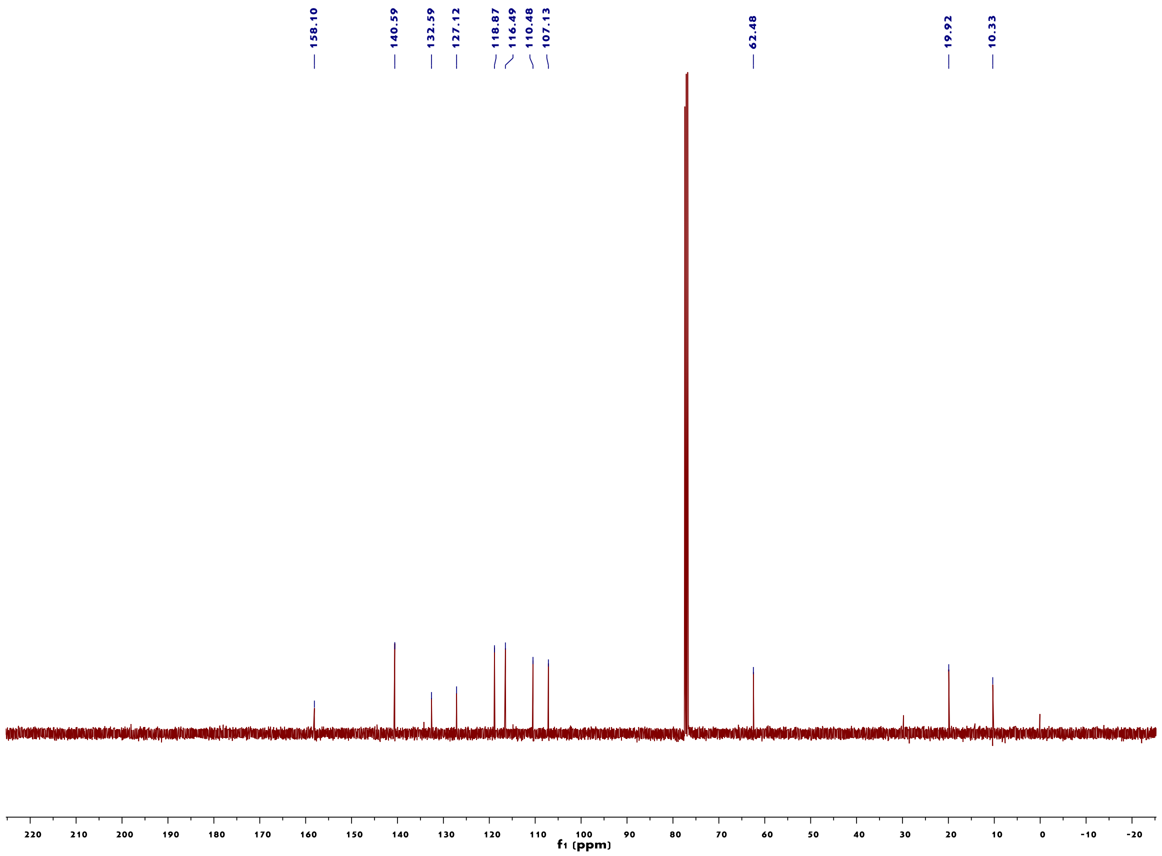

Supplementary Figure 247. ^{13}C NMR (100 MHz, $CDCl_3$) spectrum for 6baa

Supplementary References

1. Wilger, D. J., Grandjean, J. M. M., Lammert, T. R., Nicewicz, D. A. The direct anti-Markovnikov addition of mineral acids to styrenes. *Nat. Chem.* **6**, 720-726 (2014).

2. Choi, G. J., Zhu, Q., Miller, D. C., Gu, C. J., Knowles, R. R. Catalytic alkylation of remote C–H bonds enabled by proton-coupled electron transfer. *Nature* **539**, 268- (2016).

3. Joshi-Pangu, A., Lévesque, F., Roth, H. G., Oliver, S. F., L. C. Campeau, Nicewicz, D., DiRocco, D. A. Acridinium-Based Photocatalysts: A Sustainable Option in Photoredox Catalysis. *J. Org. Chem.* **81**, 7244-7249 (2016).

4. Francoeur, S., Martel, J., Wilb, N., Charette, A.-B. New Family of Cyclopropanating Reagents: Synthesis, Reactivity, and Stability Studies of Iodomethylzinc Phenoxides. *Angew. Chem. Int. Ed.* **39**, 4539-4542 (2000).

5. Chen, D.-H., Huang, D.-S., Shi, Y. Synergistic effect of additives on cyclopropanation of olefins. *Org. Biomol. Chem.* **11**, 5588-5591 (2013).

6. Cahard, E., Schoenebeck, F., Garnier, J., Cutulic, S. P. Y., Zhou, S.-Z., Murphy, J. A. Electron Transfer to Benzenes by Photoactivated Neutral Organic Electron Donor Molecules. *Angew. Chem. Int. Ed.* **51**, 3673-3676 (2012).

7. Wong, Y.-C., Ke, Z.-H., Yeung, Y.-Y. Lewis Basic Sulfide Catalyzed Electrophilic Bromocyclization of Cyclopropylmethyl Amide. *Org. Lett.* **17**, 4944-4947 (2015).

8. Sugawara, M., Yoshida, J.-I. Remarkable γ-Effect of Tin: Acid-Promoted Cyclopropanation Reactions of α-((Alkoxycarbonyl)oxy)stannanes with Alkenes. *J. Am. Chem. Soc.* **119**, 11986-11987 (1997).

9. Dai, W.-P., Zhang, X.-X., Zhang, J., Lin, Y.-Y., Cao, S. Synthesis of Exocyclic Trisubstituted Alkenes via Nickel - Catalyzed Kumada - Type Cross -

Coupling Reaction of gem‐ Difluoroalkenes with Di‐Grignard Reagents. *Advanced. Syn. Catal.* **358**, 183-187 (2016).

10. Neelamegam, R., Ricq, E. L., Malvaez, M., Patnaik, D., Norton, S., Carlin, S. M., Hill, I. T., Wood, M. A., Haggarty, S. J., Hooker, J. M. Brain-Penetrant LSD1 Inhibitors Can Block Memory Consolidation. *ACS Chemical Neuroscience*, **3**(2), 120-128 (2012).
11. Fotsch, C., Bo, Y.-X., Doherty, E. M. Discovery of Potent, Orally Available Vanilloid Receptor-1 Antagonists. Structure−Activity Relationship of N-Aryl Cinnamides. *J. Med. Chem.* **48**, 71-90 (2005).
12. Sekiguchi, Y., Kanuma, K., Sakagami, K., Hayashi, M., Kashiwa, S., Omodera, K. 1-NAPHTHYL ALKYLPIPERIDINE DERIVATIVE. *WO 2008044632 A1* Apr 17, **2008**
13. Feng, Q., Song, Q. Aldehydes and Ketones Formation: Copper-Catalyzed Aerobic Oxidative Decarboxylation of Phenylacetic Acids and α-Hydroxyphenylacetic Acids. *J. Org. Chem*. **79**, 1867-1871 (2014).
14. Onodera, S., Ishikawa, S., Kochi, T., Kakiuchi, F. Direct Alkenylation of Allylbenzenes via Chelation-Assisted C–C Bond Cleavage. *J. Am. Chem. Soc.* **140**, 9788-9792 (2018).
15. Lu, Z., Parrish, J. D., Yoon, T. P. [3+2] Photooxygenation of aryl cyclopropanes via visible light photocatalysis. *Tetrahedron* **70**, 4270-4278 (2014)
16. Wozniak, Ł., Murphy, J. J., Melchiorre, P. Photo-organocatalytic Enantioselective Perfluoroalkylation of β-Ketoesters. *J. Am. Chem. Soc.* ***137***, 5678-5681 (2015).
17. Kandukuri, S. R., Bahamonde, A., Chatterjee, I., Jurberg, I. D., Escudero-Adán, E. C., Melchiorre, P. X‐Ray Characterization of an Electron Donor‐Acceptor Complex that Drives the Photochemical Alkylation of Indoles. *Angew. Chem. Int. Ed.* ***54***, 1485-1489 (2015).
